# Nitrogen‐Doped Starbons®: Methodology Development and Carbon Dioxide Capture Capability

**DOI:** 10.1002/chem.202303436

**Published:** 2023-12-07

**Authors:** Ryan E. Barker, Michael C. Brand, James H. Clark, Michael North

**Affiliations:** ^1^ Green Chemistry Centre of Excellence Department of Chemistry University of York YO10 5DD York UK; ^2^ Department of Chemistry and Materials Innovation Factory and Leverhulme Research Centre for Functional Materials Design University of Liverpool L69 7ZD Liverpool UK

**Keywords:** carbon dioxide adsorption, melamine, microwave, nitrogen-doped, Starbon®

## Abstract

Five nitrogen sources (glycine, β‐alanine, urea, melamine and nicotinamide) and three heating methods (thermal, monomodal microwave and multimodal microwave) are used to prepare nitrogen‐doped Starbons® derived from starch. The materials are initially produced at 250–300 °C (SN_x_300_y_), then heated in vacuo to 800 °C to produce nitrogen‐doped SN_x_800_y_’s. Melamine gives the highest nitrogen incorporation without destroying the Starbon® pore structure and the microwave heating methods give higher nitrogen incorporations than thermal heating. The carbon dioxide adsorption capacities of the nitrogen‐doped Starbons® determined gravimetrically, in many cases exceed those of S300 and S800. The carbon dioxide, nitrogen and methane adsorption isotherms of the most promising materials are measured volumetrically. Most of the nitrogen‐doped materials show higher carbon dioxide adsorption capacities than S800, but lower methane and nitrogen adsorption capacities. As a result, the nitrogen‐doped Starbons® exhibit significantly enhanced carbon dioxide versus nitrogen and methane versus nitrogen selectivities compared to S800.

## Graphical abstract

**Nitrogen doping**: Glycine, β‐alanine, urea, melamine and nicotinamide are used as sustainable nitrogen sources in the preparation of nitrogen‐doped Starbons®. Doping using microwave heating more effectively incorporates nitrogen than thermal heating in vacuo. The nitrogen‐doped materials have superior carbon dioxide adsorption capacities and enhanced carbon dioxide versus nitrogen selectivities compared to undoped Starbon®.

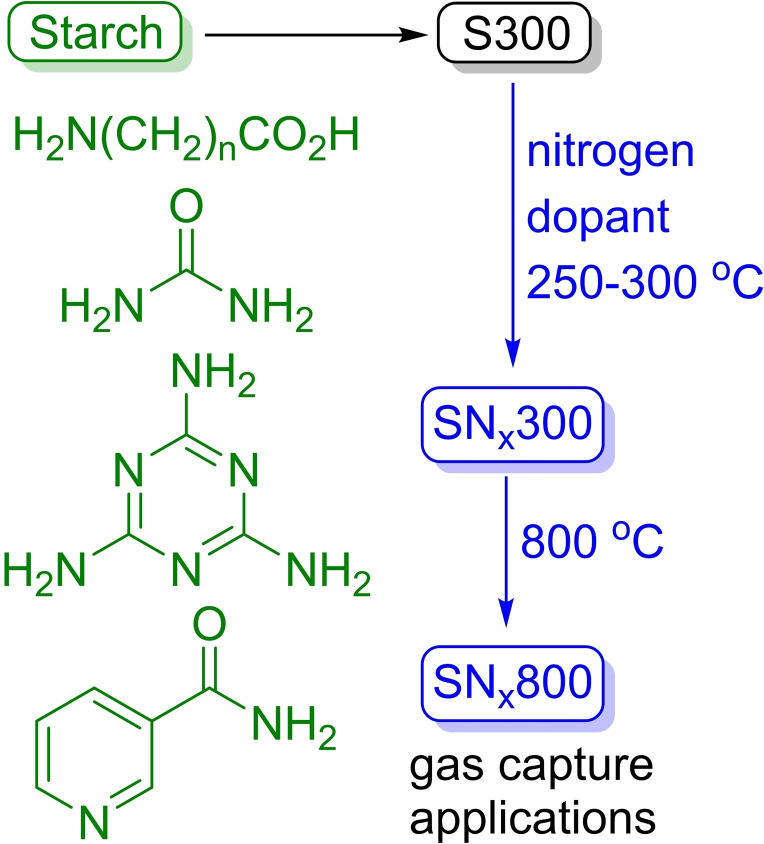

## Introduction

Starbons® are mesoporous carbons produced from waste biomass (starch, alginic acid or pectin) in a three‐step procedure without the use of a template.^[1]^ Since the first report of the synthesis of Starbons® in 2006,^[2]^ their synthesis has been optimised to maximise the sustainability of the reactants and the greenness of the processing.^[3]^ Starbon® materials are now commercially available and have been employed as catalysts;^[4]^ catalyst‐supports;^[5]^ adsorbents for gases,^[6]^ metals^[7]^ and various organic materials;^[8]^ and as electrodes.^[9]^ Recently, the synthesis of Starbons® has been modified to produce hierarchically porous materials possessing both mesopores and micropores which show enhanced adsorption capacities for carbon dioxide^[10]^ and dyes.^[11]^

**Scheme 1:**
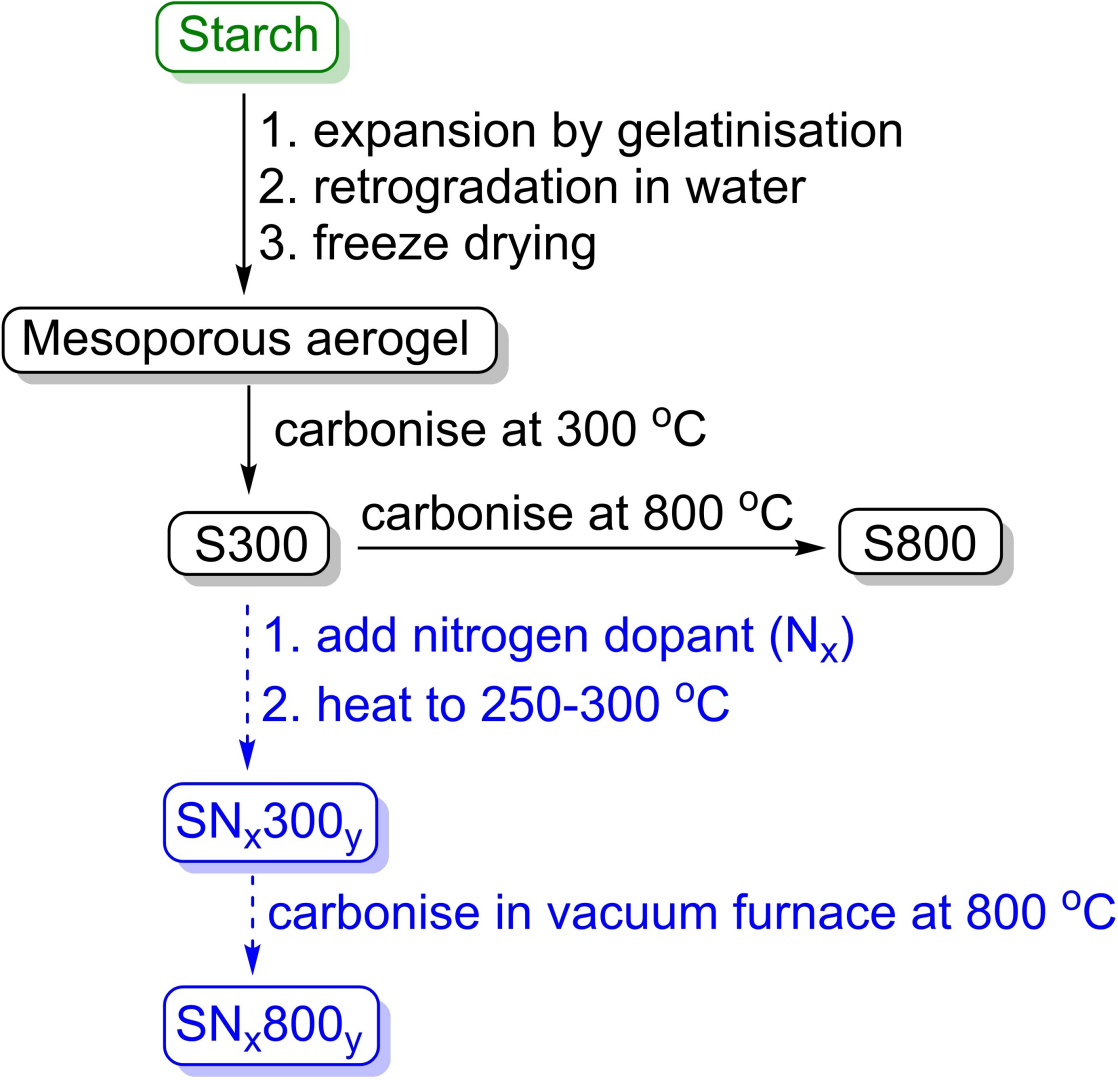
Outline of route used to prepare Starbons® (black solid arrows) and its adaptation to produce nitrogen‐doped Starbons® (blue hashed arrows). S specifies that the materials were produced from starch, N that they were nitrogen‐doped and 300 or 800 gives the maximum temperature they were heated too. X corresponds to the three letter code for a particular dopant (see Figure 1) and Y specifies the heating method used to introduce the nitrogen dopant: Th=thermal heating in a vacuum oven, Mo=microwave heating in a monomodal microwave, Mu=microwave heating in a multimodal microwave.

The synthesis of Starbons® from starch is outlined in Scheme 1. Starch is first subjected to gelatinisation and retrogradation in water to expand its pore structure. This is followed by careful drying[^3^, ^12^] to preserve the pore structure and produce a mesoporous aerogel. The aerogel is then carbonised by heating to 300 °C in a vacuum oven to produce a low temperature Starbon® (S300). Further heating produces higher temperature Starbons® (S600–S1200). The chemical groups present in Starbons® are largely determined by the carbonisation temperature.^[2]^ Carbonisation at 300 °C retains oxygen containing functionalities such as alcohols and carbonyls that were present in the starch. By 600 °C, monocyclic aromatic rings predominate and at higher temperatures, fused polycyclic aromatics are formed. However, the carbohydrate nature of starch means that only carbon, hydrogen and oxygen containing functionalities can be directly introduced. Nitrogen‐containing carbons are known to show superior heavy‐metal binding, gas adsorption, conductivity and catalytic activity compared to purely carbon derived materials.^[13]^ There has only been one previous report of the production of nitrogen‐doped Starbons® and that used ammonia as the nitrogen source to dope starch‐derived Starbons®.^[14]^ However, ammonia is undesirable as a source of nitrogen as it is not sustainably sourced and its production by the Haber‐Bosch process^[15]^ generates 1.4 % of global carbon dioxide emissions and consumes 1 % of the total energy produced globally.^[16]^ Therefore, in this paper we report the synthesis of nitrogen‐doped Starbons® using potentially more sustainably obtainable organic compounds as the nitrogen source and investigate the carbon dioxide, methane and nitrogen adsorption capabilities of the resulting nitrogen‐doped Starbons®.

## Results and Discussion

### Synthesis and characterisation of nitrogen‐doped Starbons® using thermal heating in vacuo

**Figure 1:**
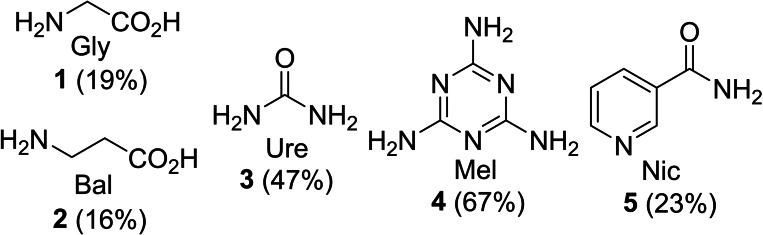
Structures, codes used in Starbon® names and (in brackets) nitrogen weight percentages of dopants **1**–**5**.

Five nitrogen containing organic compounds (Figure 1) were selected as potential dopants. Glycine **1** and β‐alanine **2** are naturally occurring amino acids. Urea **3** is also a natural product produced in mammals by protein metabolism and melamine **4** which contains 67 % nitrogen by mass is manufactured from urea. Nicotinamide **5** (also known as vitamin B_3_) is found in various foods including milk, meat and green vegetables and contains pyridinic as well as amide nitrogen. Thus, compounds **1**–**5** are all potentially available from sustainable feedstocks and contain differing amounts of nitrogen in various functional groups.

To avoid disrupting the formation of the pore network during Starbon® formation, nitrogen doping was carried out after the formation of S300.[^2^, ^3^] Therefore, a 1 : 1 (g/g) mixture of S300 and each of dopants **1**–**5** was first ground in a pestle and mortar, then heated to 300 °C in a vacuum furnace and held at 300 °C for 5 h. After cooling to room temperature, the resulting material was split into two parts. One part was thoroughly washed with deionised water (to remove excess dopant not associated with the Starbon® material) and dried at 80 °C in a vacuum oven to give SN_x_300_Th_ samples where ‘x’ corresponds to the three letter codes for dopants **1**–**5** given in Figure 1 and ‘Th’ specifies that thermal heating was used to produce the materials. The remainder of each sample was further heated to 800 °C in the vacuum furnace, to produce SN_x_800_Th_ samples. All ten samples were characterised to determine their nitrogen contents and pore structures with details given in Table 1 (see Supporting Information for full data).

**Table 1:** Chemical and structural analysis of S300, S800, SN_x_300_Th_ and SN_x_800_Th_ samples.

Entry	Sample	Combustion analysis N [%]	XPS N [%]	Solid‐state ^13^C NMR^[a]^	Powder X‐ray^[a]^	DRIFTS IR^[b]^	BET surface area^[c]^ [m^2^ g^−1^]	Total pore volume^[c]^ [cm^3^ g^−1^]	Micropore^[c]^ [%]	Mesopore^[c]^ [%]
1	S300	0.0	0.2	No	No	No	132	0.16	17	83
2	SN_Gly_300_Th_	1.3	1.4	No	No	Yes (weak)	244	0.22	28	72
3	SN_Bal_300_Th_	4.3	3.7	No	No	Yes (Strong)	63	0.1	0	100
4	SN_Ure_300_Th_	1.5	1.7	No	No	Yes (Strong)	270	0.21	32	68
5	SN_Mel_300_Th_	9.5 (17.2)^[d]^	10.6	Possible	No	Yes (Strong)	78 (45)^[d]^	0.13	0	100
6	SN_Nic_300_Th_	1.1	1.5	No		Yes (weak)	249	0.19	35	65
7	S800	0.0	0.4	^[e]^	No	^[e]^	679	0.33	64	36
8	SN_Gly_800_Th_	1.7	2.0	^[e]^	No	^[e]^	542	0.28	65	35
9	SN_Bal_800_Th_	2.9	2.7	^[e]^	No	^[e]^	504	0.28	66	34
10	SN_Ure_800_Th_	3.0	3.0	^[e]^	No	^[e]^	687	0.38	63	38
11	SN_Mel_800_Th_	7.9 (17.4)^[d]^	8.1	^[e]^	No	^[e]^	531 (622)^[d]^	0.30	61	39
12	SN_Nic_800_Th_	0.8	1.4	^[e]^	No	^[e]^	635	0.31	66	34

[a] Refers to whether unreacted dopant was detected in the sample. [b] Refers to the presence of a nitrile stretch at 2189–2228 cm^−1^. [c] Determined by porosimetry. Total pore volume determined using the HK method at *P/P_0_* =0.99; percentage micropores determined using the t‐plot method; percentage mesopores determined using the BJH method. [d] Figure in brackets was obtained using a 1 : 2 ratio (w/w) of S300 to melamine. [e] DRIFTS IR and solid‐state ^13^C NMR could not be obtained for samples heated to 800 °C.

All of the materials prepared in this work were analysed by porosimetry (data in Supporting Information) and showed type IV adsorption isotherms^17^ with hysteresis present at higher P/P_0_ ratios typical of mesporous materials. Comparison of the porosimetry data for the SN_x_300_Th_ samples (Table 1, entries 2–6) with that of the S300 sample from which they were prepared (Table 1, entry 1) shows that the SN_x_300_Th_ samples fall into two groups. SN_Bal_300_Th_ and SN_Mel_300_Th_ have much lower BET surface areas than S300 and possess no microporosity whilst the other three SN_x_300_Th_ samples all have higher BET surface areas than S300 and possess a greater percentage of micropores. In contrast, all five SN_x_800_Th_ samples have very similar BET surface areas, total pore volumes and microporosities (Table 1, entries 8–12) and these are all comparable to the porosimetry data of an undoped S800 sample (Table 1, entry 7) prepared from S300 as previously reported.^[10]^ These results suggest that the synthesis of SN_Bal_300_Th_ and SN_Mel_300_Th_ has left material within the micropores of the materials, totally blocking them and reducing the BET surface area. On further heating to 800 °C, this physisorbed material is volatilised, restoring the microporosity and BET surface area.

The other three dopants are vaporised out of the micopores below 300 °C and the enhanced BET surface area and microporosities observed for these samples can be attributed to oxygen activation of the Starbon® material^[10]^ by the small amount of air present in the vacuum furnace which operated at a pressure of less than 20 mbar. This oxygen activation is enhanced when the samples are heated to 800 °C in the vacuum furnace, resulting in all the SN_x_800_Th_ samples having similar BET surface areas and microporosities.

**Figure 2:**
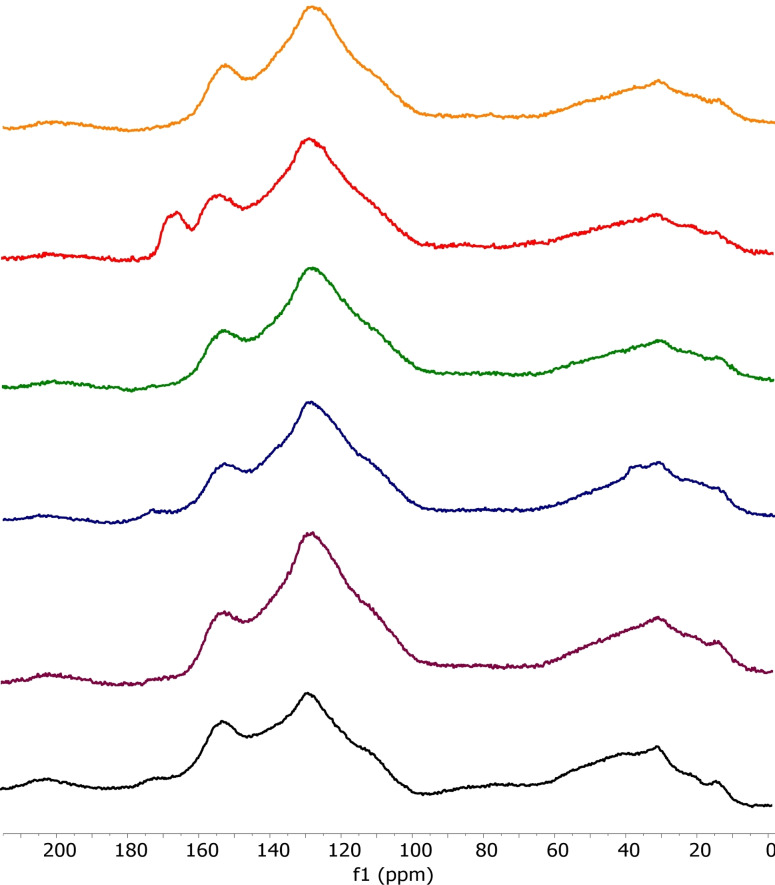
Solid‐state ^13^C NMR spectra of S300 (bottom) and in ascending order: SN_X_300_Th_ where X=Gly, Bal, Ure, Mel and Nic.

Powder X‐ray diffraction and solid‐state ^13^C NMR spectroscopy were used to investigate if any of the SN_x_300_Th_ or SN_x_800_Th_ samples did contain unreacted or decomposed dopant trapped within their pore structure. No crystalline material was detectable by powder X‐ray diffraction of any of the materials even though dopants **1**–**5** were all microcrystalline as determined by powder X‐ray diffraction (data in Supporting Information). This suggests that the material blocking the micropores in SN_Bal_300_Th_ and SN_Mel_300_Th_ was not unreacted dopant, but a non‐crystalline decomposition product (or mixture of decomposition products). Only the SN_x_300_Th_ samples could be analysed by solid‐state ^13^C NMR spectroscopy. Figure 2 shows the spectra obtained for S300 and the five SN_x_300_Th_ samples (larger versions of the spectra are given in the Supporting Information). All but one of the SN_x_300_Th_ spectra are very similar, exhibiting the same aromatic and aliphatic carbon peaks seen in S300 and showing no evidence for the presence of unreacted dopant. The spectrum of SN_Mel_300_Th_ does show an additional peak at 166 ppm which may be consistent with the presence of melamine, however this could also be due to an additional aromatic environment being present in SN_Mel_300_Th_ or in a melamine decomposition product, as powder X‐ray diffraction did not show any evidence for the presence of unreacted melamine. Overall, the porosimetry, powder X‐ray and solid‐state NMR data indicate that all the SN_x_300_Th_ and all the SN_x_800_Th_ samples have similar structures with the exception of decomposed dopant being present in the micropores of SN_Bal_300_Th_ and SN_Mel_300_Th_.

All the SN_x_300_Th_ and SN_x_800_Th_ samples (Table 1, entries 2–6 and 8–12) were found to contain nitrogen when examined both by combustion analysis and by XPS analysis (data in Supporting Information). The two techniques gave very similar values for the nitrogen contents even though combustion analysis investigates the whole sample whilst XPS only analyses the surface of a sample. This suggests that the nitrogen is equally distributed throughout the samples. The two samples which appeared to have material blocking the micropores (SN_Bal_300_Th_ and SN_Mel_300_Th_) were found to contain the most nitrogen by both XPS and combustion analysis (Table 1, entries 3 and 5). In both cases, the nitrogen content dropped significantly on heating to 800 °C (Table 1, entries 9 and 11), though SN_Mel_800_Th_ still contained around 8 % nitrogen which is more the double the nitrogen content of any other SN_X_800_Th_ sample. However, the amount of nitrogen present is, in all cases, lower than the amount that would have been expected based on the use of a 1 : 1 (g/g) mixture of S300 and dopant **1**–**5**. The nitrogen contents of dopants **1**–**5** are given in Figure 1, and the theoretical amounts of nitrogen expected in the SN_x_300_Th_ samples would be half of these (8–33.5 %). This suggests that a large amount of the dopant was either being vaporised during the vacuum heating or remained unreacted and was removed by the aqueous washing.

To investigate the thermal stability of dopants **1**–**5**, each was subjected to combined DSC‐TGA analysis under a stream of nitrogen, at atmospheric pressure (data in Supporting Information). Melamine **4** and nicotinamide **5** showed very simple TGA curves with 97–100 % weight loss in the ranges 240–360 and 170–280 °C respectively. In both cases, the DSC traces showed a broad negative heat‐flow peak (at 330 and 263 °C respectively) corresponding to an endothermic decomposition process. The DSC trace of nicotinamide also showed a sharp negative heat‐flow peak at 131 °C with no corresponding mass change corresponding to its melting point and hence indicating that decomposition occurs after melting. Urea **3** exhibited a multistage mass loss with 95 % mass loss between 149 and 400 °C. The DSC trace of urea was also complex with a sharp negative heat‐flow peak at 136 °C corresponding to the melting point, followed by a broad negative heat‐flow peak at 206 °C correlating with the main decomposition process followed by a second sharp melting peak (of a decomposition product) at 220 °C. The amino acids glycine **1** and β‐alanine **2** were not completely volatilised even at 625 °C. Glycine exhibited a sharp loss of 45 % of its weight between 235 and 280 °C (with a corresponding negative heat‐flow peak in the DSC trace at 243 °C) followed by a slow weight loss at higher temperatures with 29 % of the weight remaining at 625 °C. β‐Alanine displayed a sharp loss of 31 % weight between 202 and 240 °C followed by a second loss of 49 % weight up to 370 °C and then a slow weight loss up to 625 °C with just 11 % of the original weight remaining. The DSC trace of β‐alanine showed a negative heat‐flow peak at 204 °C which was sharp on the low temperature side but broad on the high temperature side, indicating that decomposition occurs at the melting point. A second negative heat‐flow peak at 342 °C corresponded to the second weight loss process. Since the TGA‐DSC analysis of dopants **1**–**5** had to be carried out at atmospheric pressure whilst the nitrogen doping was carried out at less than 20 mbar pressure, the TGA‐DSC data suggest that all of dopants **1**–**5** will be extensively or completely vaporised during the synthesis of nitrogen‐doped Starbons®.

Additional evidence for loss of dopant by vaporisation during the thermal treatment was obtained from an experiment in which the S300 to melamine ratio was increased from 1 : 1 to 1 : 2. This resulted in an approximate doubling of the nitrogen content of the resulting samples of SN_Mel_300_Th_ and SN_Mel_800_Th_ (Table 1, entries 5 and 11). Since melamine is completely vaporised at 350 °C even at atmospheric pressure, these enhanced nitrogen contents cannot be due to unreacted melamine, but rather indicate that much of the melamine is being vaporised before it can be incorporated into the Starbon® structure. Overall, the combustion analysis, XPS and thermal analysis data indicate that dopants **1**–**5** do effectively introduce nitrogen into SN_x_300_Th_ and SN_x_800_Th_. Melamine **4** gives significantly higher nitrogen incorporations than the other dopants. In all cases, significant amounts of dopant are lost during the vacuum heating. This can be circumvented by use of higher ratios of dopant to S300, but this is detrimental to the atom economy, so alternative heating methods were investigated instead see below.

**Figure 3:**
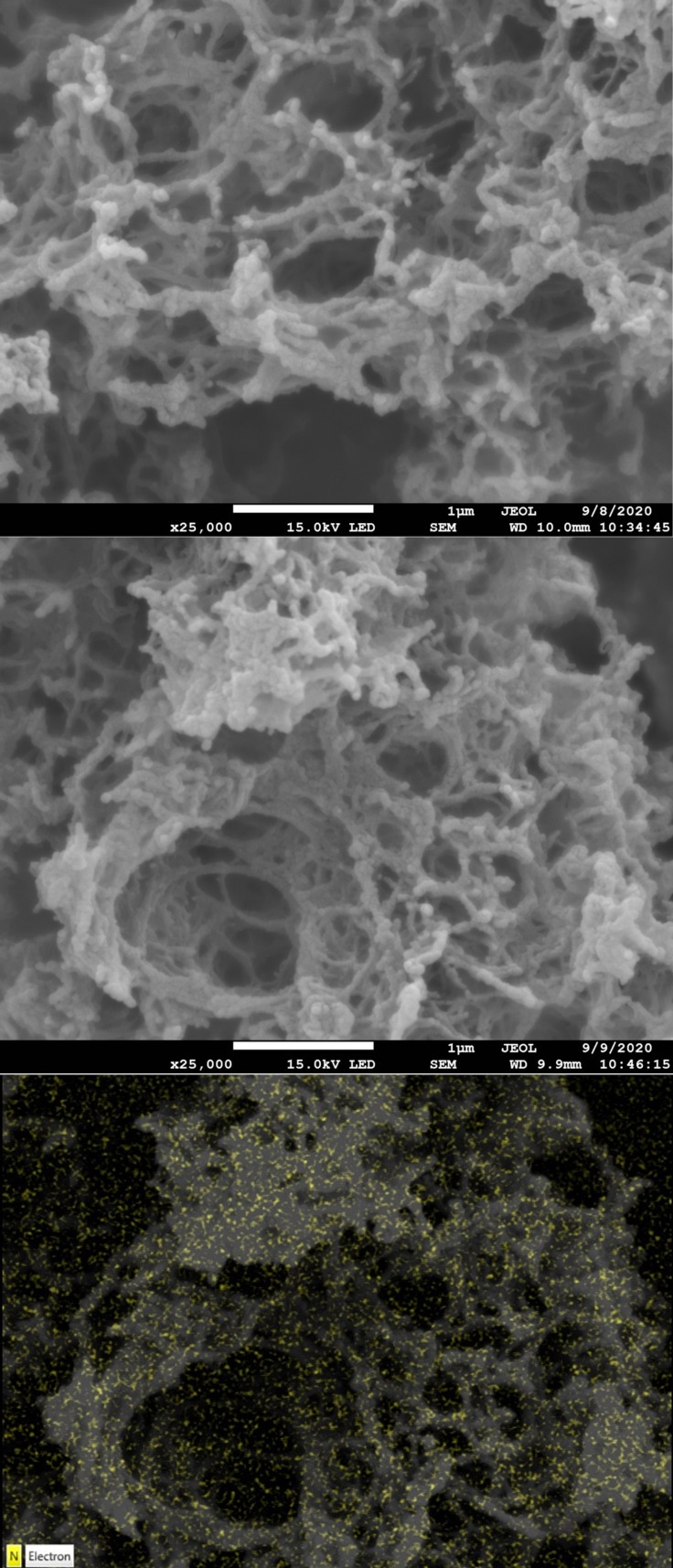
SEM images at 25,000 magnification of S300 (top) and SN_Gly_300_Th_ (middle) along with nitrogen EDX mapping of SN_Gly_300_Th_ (bottom).

SEM imaging with EDX mapping for nitrogen was carried out on S300, S800, all five SN_x_300_Th_ samples and all five SN_x_800_Th_ samples (data in Supporting Information). The undoped S300 and S800 samples had a nodular composition and showed extensive evidence for the presence of macropores and mesopores (Figure 3). All the nitrogen‐doped materials retained the nodular architecture and showed the same evidence for the presence of macropores and mesopores. EDX mapping indicated that the nitrogen was distributed throughout the surface of the samples. TEM imaging was also carried out on the samples to image the micropore structure. All the samples were found to contain micropores (data in Supporting Information), even the SN_Bal_300_Th_ and SN_Mel_300_Th_ samples for which porosimetry suggested no micropores were present. However, it is not possible to quantify the TEM data and the area imaged may not be representative of the sample as a whole. Thus, the SEM and TEM imaging showed that the overall structure of Starbons® was not changed as a result of the nitrogen doping and EDX mapping confirmed that nitrogen was present through the samples. Information on how nitrogen was incorporated in the SN_x_300_Th_ samples was obtained by DRIFTS analysis. All of the spectra (but not that of S300) showed an adsorption at 2189–2228 cm^−1^ corresponding to a nitrile group. Thus, at least some of the nitrogen is incorporated as cyano‐groups, a result that is consistent with previous work using ammonia as the nitrogen dopant.^[14]^ The DRIFTS adsorption data was consistent with the N1s binding energies obtained from the XPS spectra (data in Supporting Information). Thus, the SN_x_300_Th_ samples exhibited two to four separate N1s binding energies and one of these was always in the region of 398.6–399.1 eV. The N1s energy of a nitrile has been reported at 399.0 eV.^[18]^ However, the N1s binding energies of nitrogen containing species are not sufficiently well resolved to allow definitive functional group assignments to be made solely on their basis. Thus, for example the N1s binding energy of the conjugated tripyridine (2,2’:6’2”‐ terpyridine) is reported at 398.5 eV, whilst the N1s binding energy of the fused pyridine, acridine, is reported at 399.2 eV.^[17]^ The SN_x_800_Th_ samples all showed four N1s binding energies and one of these was always at 398.1–398.2 eV, consistent with the presence of pyridine units within in the sample. Comparison of the fingerprint region of the DRIFT spectra of SN_x_300_Th_ with the corresponding region of the spectra of dopants **1**–**5** showed no evidence for the presence of unreacted dopant in the SN_x_300_Th_ samples, supporting the data obtained from powder X‐ray and solid‐state ^13^C NMR analysis. Thus, the DRIFTS and XPS data show that nitrogen doping of S300 to produce SN_x_300_Th_ results in the formation of nitrile groups within the Starbon® material.

### Synthesis and characterisation of nitrogen‐doped Starbons® using microwave heating

The results presented above demonstrated that dopants **1**–**5** could be used to prepare nitrogen‐doped Starbons® containing up to 17 % nitrogen. However, a large amount of the dopant was lost during the vacuum heating. Therefore, two different microwave heating methods were investigated to try to overcome this problem. Initially, a monomodal microwave (CEM Discover CP) was used to heat a 1 : 1 mixture of S300 and dopant **1**–**5** in a sealed system to prevent loss of dopant and produce SN_x_300_Mo_ samples. These were then heated in the vacuum furnace to produce SN_x_800_Mo_ samples. The use of a multimodal microwave (Milestone SynthWAVE) was also investigated. This system was designed to heat liquids and has a maximum temperature of 250 °C, so dopant **1**–**5** (10 equivalents) was first dissolved in water, then S300 was added and the mixture heated in a sealed system to produce SN_x_300_Mu_ samples which were further heated in a vacuum furnace to produce SN_x_800_Mu_ samples. All the microwave prepared samples were characterised to determine their nitrogen contents and pore structures with details given in Table 2 (see Supporting Information for full data).

**Table 2:** Chemical and structural analysis of S300, S800, SN_x_300_Mo,_ SN_x_800_Mo,_ SN_x_300_Mu_ and SN_x_800_Mu_ samples.

Entry	Sample	Combustion analysis N [%]	XPS N [%]	Solid‐state ^13^C NMR^[a]^	Powder X‐ray^[a]^	DRIFTS IR^[b]^	BET surface area^[c]^ [m^2^ g^−1^]	Total pore volume^[c]^ [cm^3^ g^−1^]	Micropore^[c]^ [%]	Mesopore^[c]^ [%]
1	S300	0.0	0.2	No	No	No	132	0.16	17	83
2	SN_Gly_300_Mo_	6.5	5.4	Yes (weak)	No	Yes (weak)	57	0.11	0	100
3	SN_Bal_300_Mo_	4.6	5	Yes (strong)	Modified	Yes (strong)	43	0.10	0	100
4	SN_Ure_300_Mo_	6.9	6.9	Yes (weak)	Modified	Yes (weak)	95	0.15	10	90
5	SN_Mel_300_Mo_	11.3	8.5	Yes (medium)	Yes	Yes (strong, br)	60	0.10	0	100
6	SN_Nic_300_Mo_	2.9	3.8	No	No	Yes (strong)	323	0.23	45	55
7	SN_Gly_300_Mu_	1.3	2.3	No	No	No	127	0.21	12	88
8	SN_Bal_300_Mu_	1.3	1.9	No	No	No	148	0.18	20	80
9	SN_Ure_300_Mu_	2.3	1.6	No	Modified	No	188	0.23	18	82
10	SN_Mel_300_Mu_	9.5	8.7	Yes (medium)	Modified	No	115	0.15	18	82
11	SN_Nic_300_Mu_	0.2	0.7	No	Modified	No	320	0.24	33	67
12	S800	0.0	0.4	^[d]^	No	^[d]^	679	0.33	64	36
13	SN_Gly_800_Mo_	6.1	^[e]^	^[d]^	No	^[d]^	821	0.40	67	33
14	SN_Bal_800_Mo_	4.3	^[e]^	^[d]^	No	^[d]^	697	0.33	71	29
15	SN_Ure_800_Mo_	9.0	^[e]^	^[d]^	No	^[d]^	863	0.42	65	35
16	SN_Mel_800_Mo_	8.4	^[e]^	^[d]^	No	^[d]^	732	0.34	68	32
17	SN_Nic_800_Mo_	3.9	^[e]^	^[ d]^	No	^[ d]^	783	0.39	67	33
18	SN_Gly_800_Mu_	2.7	^[e]^	^[d]^	No	^[d]^	962	0.51	62	38
19	SN_Bal_800_Mu_	3.1	^[e]^	^[d]^	No	^[d]^	1147	0.60	62	38
20	SN_Ure_800_Mu_	5.0	^[e]^	^[d]^	No	^[d]^	1295	0.68	58	42
21	SN_Mel_800_Mu_	11.2	^[e]^	^[d]^	No	^[d]^	1126	0.63	58	42
22	SN_Nic_800_Mu_	0.0	^[e]^	^[d]^	No	^[d]^	1704	0.92	47	53

[a] Refers to whether unreacted or modified dopant was detected in the sample. [b] Refers to the presence of a nitrile stretch at 2193–2232 cm^−1^. [c] Determined by porosimetry. [d] DRIFTS IR and solid‐state ^13^C NMR could not be obtained for samples heated to 800 °C. [e] Not determined.

Comparison of the porosimetry data for the SN_x_300_Mo_ samples (Table 2, entries 2–6) with that of unmodified S300 (Table 2, entry 1), reveals that nicotinamide **5** has behaved differently to dopants **1**–**4**. Thus, whilst dopants **1**–**4** all gave SN_x_300_Mo_ samples with lower BET surface areas and lower total pore volumes than those of the S300 from which they were prepared, SN_Nic_300_Mo_ had a higher BET surface area and higher total pore volume than S300. SN_Gly_300_Mo_, SN_Bal_300_Mo_ and SN_Mel_300_Mo_ (Table 2, entries 2, 3 and 5) had no microporosity and SN_Ure_300_Mo_ (Table 2, entry 4) had lower microporosity than S300. In contrast, SN_Nic_300_Mo_ (Table 2, entry 6) had significantly higher microporosity than S300. These results can be explained on the basis that within SN_Gly_300_Mo_, SN_Bal_300_Mo_, SN_Ure_300_Mo_ and SN_Mel_300_Mo_, material derived from dopant **1**–**4** is completely or partially blocking the micropores in the Starbon® material, thus reducing the total pore volume and BET surface area. In contrast, for SN_Nic_300_Mo_ no dopant derived material is present in the sample and the microwave thermal treatment has resulted in activation of the Starbon® material, increasing its total pore volume, BET surface area and microporosity. This activation is probably induced by steam^[19]^ generated by dehydration of S300 and/or nicotinamide **5**.

SEM images of the SN_x_300_Mo_ materials (data in Supporting Information) revealed that they all had a nodular morphology similar to that of undoped S300 and the SN_x_300_Th_ materials (Figure 3). EDX mapping showed that nitrogen was distributed throughout the SN_x_300_Mo_ samples. TEM images (data in Supporting Information) showed that the SN_x_300_Mo_ materials were hierarchically porous and possessed macropores, mesopores and micropores.

The presence of dopant‐derived material blocking the micropores in SN_Gly_300_Mo_, SN_Bal_300_Mo_, SN_Ure_300_Mo_ and SN_Mel_300_Mo_ but not in SN_Nic_300_Mo_ was supported by powder X‐ray diffraction and solid‐state ^13^C NMR studies of the materials (data in Supporting Information). For SN_Gly_300_Mo_, the solid‐state ^13^C NMR spectrum indicated the presence of glycine **1** and for SN_Mel_300_Mo_, both the solid‐state ^13^C NMR spectrum and the powder X‐ray diffraction plot showed the presence of unreacted melamine **4**. Powder X‐ray diffraction analysis of SN_Bal_300_Mo_ and SN_Ure_300_Mo_ indicated the presence of crystalline material though the X‐ray peaks were at different positions to those of pure β‐alanine **2** and urea **3**. The solid‐state ^13^C NMR spectra of these two materials did exhibit peaks consistent with unreacted monomer, though given the limited resolution of solid‐state NMR these could also have derived from a modified form of the dopant such as an oligomer. Neither powder X‐ray diffraction nor solid‐state ^13^C NMR spectroscopy showed any evidence for dopant derived material being present in the SN_Nic_300_Mo_ sample. Powder X‐ray diffraction of the SN_x_800_Mo_ materials showed no evidence of dopant derived material being present, but did indicate that SN_Mel_800_Mo_ and SN_Nic_800_Mo_ have a different microstructure (more pronounced 100 and 004 planes^[20]^) than the other three samples.

DRIFTS analysis of the SN_x_300_Mo_ samples (data in Supporting Information) indicated that nitrogen doping of the Starbon® had been achieved, even when dopant‐derived material was present, as all the samples showed an adsorption at 2193–2232 cm^−1^ corresponding to a nitrile group as previously reported for ammonia‐doped Starbons®.^[14]^

Combustion analysis and XPS analysis of the SN_x_300_Mo_ samples (data in Supporting Information) confirmed the presence of nitrogen in the samples and the percentage of nitrogen present found by the two techniques were in good agreement (Table 2, entries 2–6). The actual nitrogen content values will be affected by the presence of unreacted or modified dopant (except for SN_Nic_300_Mo_). However, the SN_x_800_Mo_ samples (Table 2, entries 13–17) were found to possess very similar nitrogen contents to the corresponding SN_x_300_Mo_. As discussed above, heating to 800 °C will have vaporised any unreacted dopant in the samples, so the combustion analysis data on the SN_x_800_Mo_ samples is a true reflection of the level of nitrogen doping achieved and also suggests that unreacted dopant only makes a minor contribution to the nitrogen contents determined for the SN_x_300_Mo_ samples.

Porosimetry analysis of the SN_x_800_Mo_ samples supported the removal of all dopant derived material from the pores of the materials. The SN_x_800_Mo_ samples (Table 2, entries 13–17) all had higher surface areas than that of undoped S800 (Table 2, entry 12) and had total pore volumes which were higher than or identical to that of undoped S800. The micropore to mesopore ratios were also similar to those of undoped S800.

Overall, the above results demonstrate that use of a monomodal microwave to nitrogen dope Starbons® is an effective way of increasing the nitrogen content of the resulting materials. Whilst most of the SN_x_300_Mo_ materials contained dopant derived material within their pore structures, this was not the case for SN_Nic_300_Mo_ and its nitrogen content (2.9–3.8 %; Table 2, entry 6) was more than double that of SN_Nic_300_Th_ (1.1–1.5 %; Table 1, entry 6). Conversion of the SN_x_300_Mo_ samples into the corresponding SN_x_800_Mo_ materials removed any unreacted dopant and, in all cases, gave a higher nitrogen incorporation than the corresponding SN_x_800_Th_ sample as determined by combustion analysis. The increase is particularly pronounced for doping using glycine **1** (1.7–6.1 %; compare Table 1, entry 8 and Table 2, entry 13) and nicotinamide **5** (0.8–3.9 %; compare Table 1, entry 12 and Table 2, entry 17). The highest nitrogen incorporation was again obtained using melamine **4** as dopant, which gave SN_Mel_800_Mo_ containing 8.4 % nitrogen (Table 2, entry 16).

Production of SN_x_300_Mu_ samples by heating S300 with aqueous solutions of dopants **1**–**5** at 250 °C gave rather different results to thermal or monomodal microwave heating (Table 2, entries 7–11). As with monomodal microwave heating, nicotinamide **5** was found to behave differently to the other dopants. Thus, the nitrogen contents of SN_Gly_300_Mu_, SN_Bal_300_Mu_, SN_Ure_300_Mu_ and SN_Mel_300_Mu_ as determined by combustion analysis and XPS analysis (data in Supporting Information) were similar to those of the corresponding SN_x_300_Th_ materials produced in a vacuum furnace (Table 1, entries 2–5), but the nitrogen content of SN_Nic_300_Mu_ was very low (0.2–0.7 %) and possibly entirely due to nicotinamide derived material adsorbed onto the S300 rather than to nitrogen doping of the S300. The SN_Nic_300_Mu_ sample was also exceptional when analyzed by porosimetry (data in Supporting Information). The other four SN_x_300_Mu_ samples all had similar BET surface areas, total pore volumes and micropore to mesopore ratios as undoped S300. However, SN_Nic_300_Mu_ had a BET surface area 2.4 times greater than that of S300 and its percentage of micropores was double that of S300 (Table 2, entries 1 and 11). This indicates that under the multimodal microwave conditions and in the presence of nicotinamide **5**, S300 undergoes steam activation^[18]^ rather than nitrogen doping.

Use of the amino acids glycine **1** and β‐alanine **2** as dopants (Table 2, entries 7 and 8) produced SN_x_300_Mu_ materials uncontaminated by dopant or dopant derivatives as assessed by powder X‐ray diffraction and solid‐state ^13^C NMR spectroscopy of the materials (data in Supporting Information). This is probably due to the high solubility of the amino acids in water. SN_x_300_Mu_ samples prepared from dopants **3**–**5** did contain crystalline material derived from the dopant as determined by powder X‐ray diffraction and unreacted melamine was detected in SN_Mel_300_Mu_ by solid‐state ^13^C NMR spectroscopy. Powder X‐ray diffraction of the SN_x_800_Mu_ samples showed no evidence of unreacted dopant remaining in these samples (data in Supporting Information).

SEM images of SN_Gly_300_Mu_, SN_Bal_300_Mu_, SN_Ure_300_Mu_ and SN_Nic_300_Mu_ all showed the same nodular and highly porous morphology seen for S300 and the other nitrogen‐doped S300’s. EDX mapping again showed an even distribution of nitrogen across the samples (data in Supporting Information). Material with the same nodular morphology was also present in SN_Mel_300_Mu_, but in this case an additional crystalline, needle shaped material was also present. EDX mapping showed that the crystals were rich in nitrogen. Based on the powder X‐ray diffraction, solid‐state ^13^C NMR data and SEM images, the crystalline material appears to be melamine‐based^[21]^ and totally separate to the nitrogen‐doped S300 material. TEM images of the SN_x_300_Mu_ samples (data in Supporting Information) were all similar to the TEM images of materials prepared in a vacuum furnace or monomodal microwave, showing the presence of mesopores and micropores within the materials. TEM images were also obtained on the crystalline material present in the SN_Mel_300_Mu_ sample and showed that it did not contain pores.

Unlike the SN_x_300_Th_ and SN_x_300_Mo_ samples, no evidence of nitrile formation was seen in the DRIFTS spectra of the SN_x_300_Mu_ materials (data in Supporting Information). This is likely due to the high temperature aqueous condition preventing the dehydration of other functional groups to nitriles and/or facilitating the hydrolysis of nitriles to amides.

The five SN_x_300_Mu_ samples were converted into the corresponding SN_x_800_Mu_ materials (Table 2, entries 18–22). Combustion analysis on the SN_x_800_Mu_ materials showed that for SN_Gly_800_Mu_, SN_Bal_800_Mu_, SN_Ure_800_Mu_ and SN_Mel_800_Mu_ the nitrogen contents all increased compared to the corresponding SN_x_300_Mu_ materials due to preferential pyrolysis of the carbon, hydrogen and oxygen in the samples. When dopants **1**–**3** were used, the nitrogen contents in the SN_x_300_Mu_ materials were higher than those in the corresponding SN_x_300_Th_ materials, but not as high as those in the SN_x_300_Mo_ samples. However, melamine **4** gave SN_Mel_800_Mu_ containing 11.2 % nitrogen (Table 2, entry 21) which is higher than any other material prepared at 800 °C. SN_Nic_800_Mu_ was found not to contain any nitrogen (Table 2, entry 22), consistent with the very low amount of nitrogen present in SN_Nic_300_Mu_ (Table 2, entry 11) being due to nicotinamide derived material in the sample and not due to nitrogen doping of the Starbon®.

Porosimetry analysis of the SN_x_800_Mu_ materials (Table 2, entries 18–22) revealed that they had the highest BET surface areas and largest total pore volumes of any of the nitrogen‐doped Starbon® samples (data in Supporting Information). The BET surface areas were 1.4–2.5 times that of S800 and the total pore volumes were 1.5–2.8 times that of S800 (Table 2, entries 12 and 18–22). These high BET surface areas and pore volumes are likely caused by steam activation^[18]^ of the SN_x_300_Mu_ materials with the steam coming from dehydration of remaining alcohol groups within the SN_x_300_Mu_ and/or from water adsorbed within the SN_x_300_Mu_ framework. The four nitrogen‐doped SN_x_800_Mu_ materials all had similar micropore to mesopore ratios to undoped S800 (Table 2, entries 12 and 18–21). However, SN_Nic_800_Mu_, which contains no nitrogen, had lower microporosity, despite its exceptionally high BET surface area and pore volume (Table 2, entry 22).

In summary, microwave‐based heating methods are preferable to thermal heating in a vacuum oven for the synthesis of SN_x_800_Y_ materials. The microwave‐produced materials have higher BET surface areas and pore volumes than those produced in a vacuum furnace and contain a higher percentage of nitrogen (except for SN_Nic_800_Mu_). For four of the dopants (**1**–**3,5**), the monomodal microwave method gave the highest nitrogen incorporations (3.9–6.1 %), but for melamine **4**, the multimodal microwave method gave the highest nitrogen incorporation (11.2 %). Melamine **4** gave the high nitrogen incorporations with all three heating methods.

### Carbon dioxide capture abilities of nitrogen‐doped Starbons®

Carbon dioxide capture is a key part of both carbon capture and storage (CCS)^[22]^ and carbon capture and utilisation (CCU),^[23]^ technologies which are vital to prevent further climate change^[24]^ and transition the chemicals industry onto sustainable feedstocks.^[21]^ Reversible adsorption of carbon dioxide onto solids is one of the most promising processes for carbon dioxide capture from waste gas streams even at low carbon dioxide partial pressures.^[25]^ Zeolites,^[26]^ silicas,^[27]^ MOFs,^[28]^ COFs,^[29]^ metal oxides^[30]^ and carbon‐based materials^[31]^ have all been studied for carbon dioxide capture and we have previously shown that Starbons® have high carbon dioxide adsorption capacities and rapid adsorption/desorption kinetics.[^6^, ^10^] Nitrogen‐doping has been reported to increase the interactions between carbon‐based materials and carbon dioxide and hence to result in enhanced carbon dioxide adsorption capacities.^[32]^ Since this work has resulted in the production of thirty nitrogen‐doped Starbons® differing in their nitrogen contents, surface areas, pore volumes and surface functional groups (Tables 1 and 2), it was decided to investigate the use of these materials for carbon dioxide capture.

**Figure 4:**
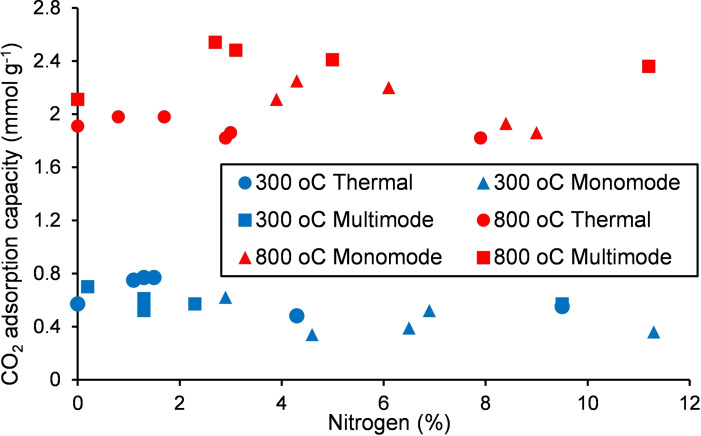
Plot of gravimetrically determined CO_2_ adsorption capacity at 308 K versus nitrogen content for Starbon® materials.

An initial screening of all thirty nitrogen‐doped Starbons®, along with S300 and S800 for comparison, was carried out gravimetrically at 308 K (data in Supporting Information), giving the results shown in Table 3. Figure 4 shows the relationship between carbon dioxide adsorption capacity and the nitrogen content of the materials. From Figure 4 it is apparent that the temperature at which the Starbon® material was prepared has a significant impact on its carbon dioxide adsorption capacity. All the materials prepared at 800 °C (shown in red) have adsorption capacities about four times those of materials prepared at 300 °C (shown in blue), consistent with previous work.^[6]^ There appears to be no dependence of the carbon dioxide adsorption capacity on the nitrogen content for the materials prepared at either temperature. The mode of production of the nitrogen‐doped Starbon® material did however influence the carbon dioxide adsorption capacity as the four highest values 2.36–2.54 mmol g^−1^ were all obtained using nitrogen‐doped materials prepared in the multimode microwave system, then heated to 800 °C (red squares in Figure 4).

**Table 3:** CO_2_ adsorption capacities of nitrogen‐doped Starbons® determined gravimetrically at 308 K.

Material	CO_2_ adsorption capacity [mmol g^−1^] and nitrogen content [%]		
	Y=Th	Y=Mo	Y=Mu
S300	0.57±0.02 (0.0)		
SN_Gly_300_Y_	0.77±0.02 (1.3)	0.39±0.02 (6.5)	0.52±0.05 (1.3)
SN_Bal_300_Y_	0.48±0.02 (4.3)	0.34±0.02 (4.6)	0.61±0.02 (1.3)
SN_Ure_300_Y_	0.77±0.02 (1.5)	0.52±0.02 (6.9)	0.57±0.02 (2.3)
SN_Mel_300_Y_	0.55±0.05 (9.5)	0.36±0.02 (11.3)	0.57±0.07 (9.5)
SN_Nic_300_Y_	0.75±0.02 (1.1)	0.62±0.02 (2.9)	0.70±0.05 (0.2)
S800	1.91±0.02 (0.0)		
SN_Gly_800_Y_	1.98±0.02 (1.7)	2.20±0.07 (6.1)	2.54±0.02 (2.7)
SN_Bal_800_Y_	1.82±0.02 (2.9)	2.25±0.07 (4.3)	2.48±0.02 (3.1)
SN_Ure_800_Y_	1.86±0.02 (3.0)	1.86±0.07 (9.0)	2.41±0.05 (5.0)
SN_Mel_800_Y_	1.82±0.02 (7.9)	1.93±0.05 (8.4)	2.36±0.02 (11.2)
SN_Nic_800_Y_	1.98±0.02 (0.8)	2.11±0.02 (3.9)	2.11±0.07 (0.0)

**Figure 5:**
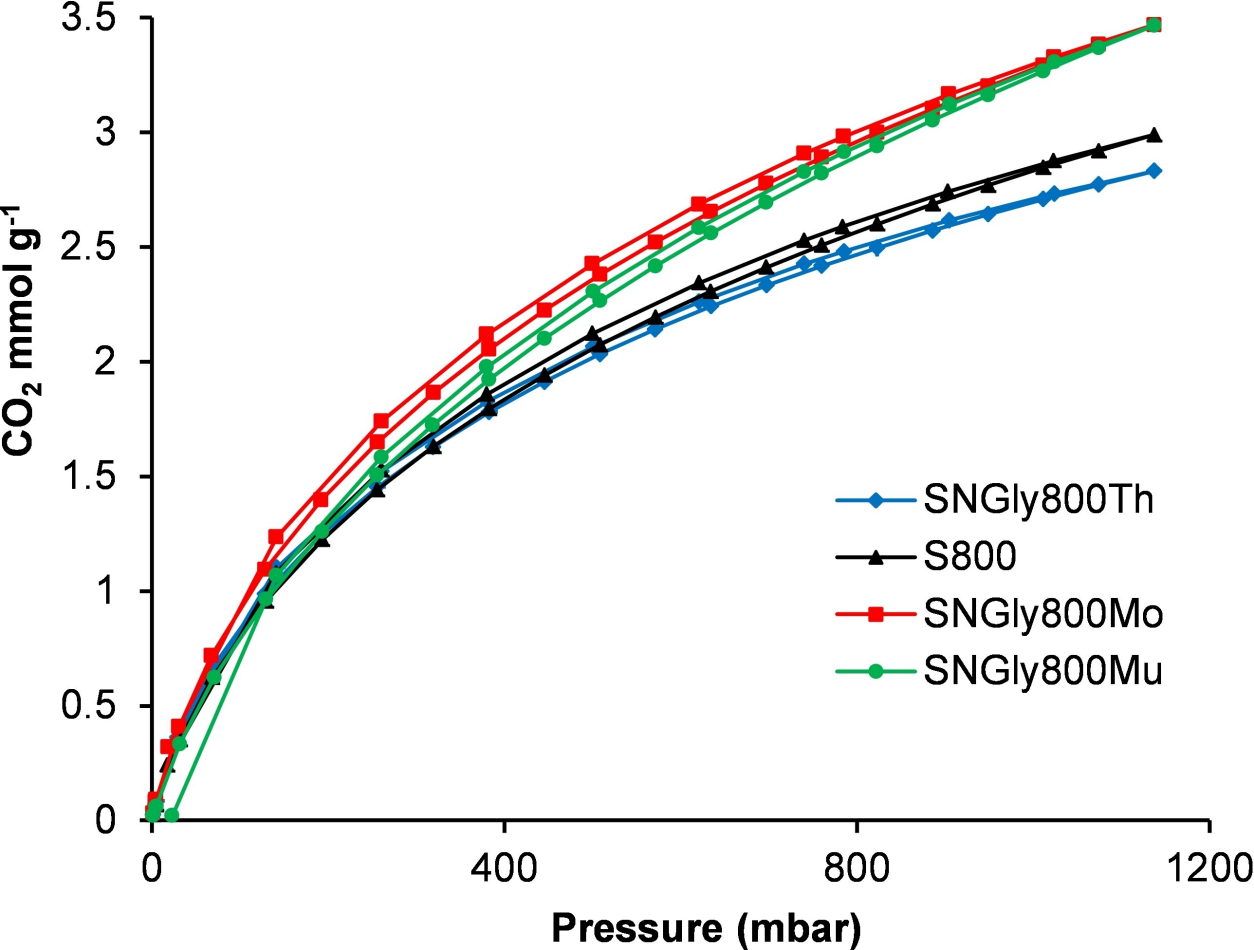
Carbon dioxide adsorption isotherms for S800 and SN_Gly_800_Y_ measured at 298 K.

Whilst the gravimetric method allows a rapid screening of the carbon dioxide adsorption capacities of materials, it is limited to atmospheric pressure. Therefore, the carbon dioxide adsorption of selected materials was also determined volumetrically by measuring their carbon dioxide adsorption isotherms at 1–1137 mbar at 298 K (data in Supporting Information). Initially, the three SN_Gly_800_Y_ materials were studied along with S800. The glycine‐doped materials were selected as they had the best, equal‐best or second‐best carbon dioxide adsorption capacities determined gravimetrically for each heating method (Table 3). Figure 5 shows the carbon dioxide adsorption isotherms obtained and Table 4 tabulates the adsorption capacities at 1 bar pressure.

**Table 4:** Gas adsorption capacities (mmol g^−1^ at 1 bar pressure and 298 K) and selectivities of nitrogen‐doped Starbons® determined volumetrically.

Material (nitrogen content [%])	capacity			Selectivity^[a]^		
	CO_2_	N_2_	CH_4_	CO_2_/N_2_	CO_2_/CH_4_	N_2_/CH_4_
S800 (0)	2.85	0.49	1.34	32.9	12.1	2.1
SN_Gly_800_Th_ (1.7)	2.71	0.46	1.25	33.3	12.2	2.1
SN_Gly_800_Mo_ (6.1)	3.29	0.46	1.27	40.5	14.7	2.0
SN_Gly_800_Mu_ (2.7)	3.27	0.44	1.27	41.7	14.6	2.0
SN_Bal_800_Mo_ (4.3)	3.27	0.47	1.38	39.2	13.4	2.0
SN_Ure_800_Mo_ (6.1)	3.26	0.43	1.25	42.6	14.8	2.0
SN_Mel_800_Mo_ (8.4)	3.35	0.44	1.30	43.2	14.6	1.9
SN_Nic_800_Mo_ (3.9)	3.41	0.47	1.37	40.8	14.0	2.0

[a] Selectivities are calculated at gas 1/gas 2 partial pressures of 0.15 and 0.85 respectively.

It is apparent from Figure 5 and Table 4 that the differences between the carbon dioxide adsorption capacities of S800 and the SN_Gly_800_Y_ materials are relatively small. SN_Gly_800_Th_ had a slightly lower carbon dioxide adsorption capacity than undoped S800 (2.71 versus 2.85 mmol g^−1^ at 1 bar pressure) whilst SN_Gly_800_Mo_ and SN_Gly_800_Mu_ had slightly higher carbon dioxide adsorption capacities than undoped S800 (3.29 and 3.27 versus 2.85 mmol g^−1^ at 1 bar pressure). Figure 5 shows that at pressures below 1 bar, SN_Gly_800_Mo_ slightly outperforms SN_Gly_800_Mu_. Therefore, the volumetric carbon dioxide adsorption study was extended to the other SN_X_800_Mo_ materials. Data for these is given in Table 4 and the isotherms are given in the Supporting Information. All of the SN_X_800_Mo_ materials had very similar carbon dioxide adsorption capacities (3.26–3.41 mmol g^−1^ at 1 bar pressure) and these did not correlate with their nitrogen contents as SN_Nic_800_Mo_ had the lowest nitrogen content (3.9 %), but the highest carbon dioxide adsorption capacity. Attempts were also made to correlate the carbon dioxide adsorption capacities with the structural properties of the Starbon® materials given in Tables 1 and 2. However, only moderate correlations were associated with the micropore volume (*R* ^2^=0.6) and BET surface area (*R* ^2^=0.5) and no correlation was apparent with the total pore volume (*R* ^2^=0.3) or mesopore volume (*R* ^2^=0.1).

### Selectivity of carbon dioxide capture by nitrogen‐doped Starbons®

Once the carbon dioxide adsorption capacity of a material exceeds 3 mmol g^−1^, the selectivity for carbon dioxide adsorption versus other gases present is more important than further enhancement of the carbon dioxide adsorption capacity.^[33]^ The two most important gases that carbon dioxide needs to be separated from are nitrogen (for applications in post‐combustion carbon capture, utilisation and storage[^32^, ^34^]) and methane (for applications in biogas purification^[35]^). Therefore, nitrogen and methane adsorption isotherms were also obtained for each of the Starbon® materials in Table 4 (data in Supporting Information). For all of the materials, the adsorption capacities decreased in the order: carbon dioxide>methane>nitrogen.

The adsorption capacities of the three gases then allowed the selectivity (S) for each pair of gases to be calculated on the basis of ideal adsorbed solution theory (IAST)^[36]^ using Equation (1) where q_1_ and q_2_ are the adsorbed amounts of the two gases at partial pressures of *p*_1_ and *p*_2_, respectively.^[37]^ To determine the selectivity of carbon dioxide versus nitrogen, partial pressures of 0.15 (CO_2_) and 0.85 (N_2_) were used as these correspond to the typical composition of the flue‐gas from a coal burning power station.[^32^, ^33^] The resulting selectivities are given in Table 4. Even though nitrogen is present in a 5–6 fold excess, S800 displayed a selectivity in favour of carbon dioxide of 32.9. All of the nitrogen‐doped Starbons® had higher selectivities than S800 suggesting that nitrogen incorporated into the Starbon® materials has a favourable interaction with carbon dioxide. The highest selectivity (43.2) was shown by SN_Mel_800_Mo_. This, combined with the high carbon dioxide adsorption capacity (3.35 mmol g^−1^) of this material, suggests that SN_Mel_800_Mo_ is a promising material for post‐combustion carbon dioxide capture applications.

(1) $$S=\frac{q_{1}p_{2}}{q_{2}p_{1}}\;$$

**Figure 6:**
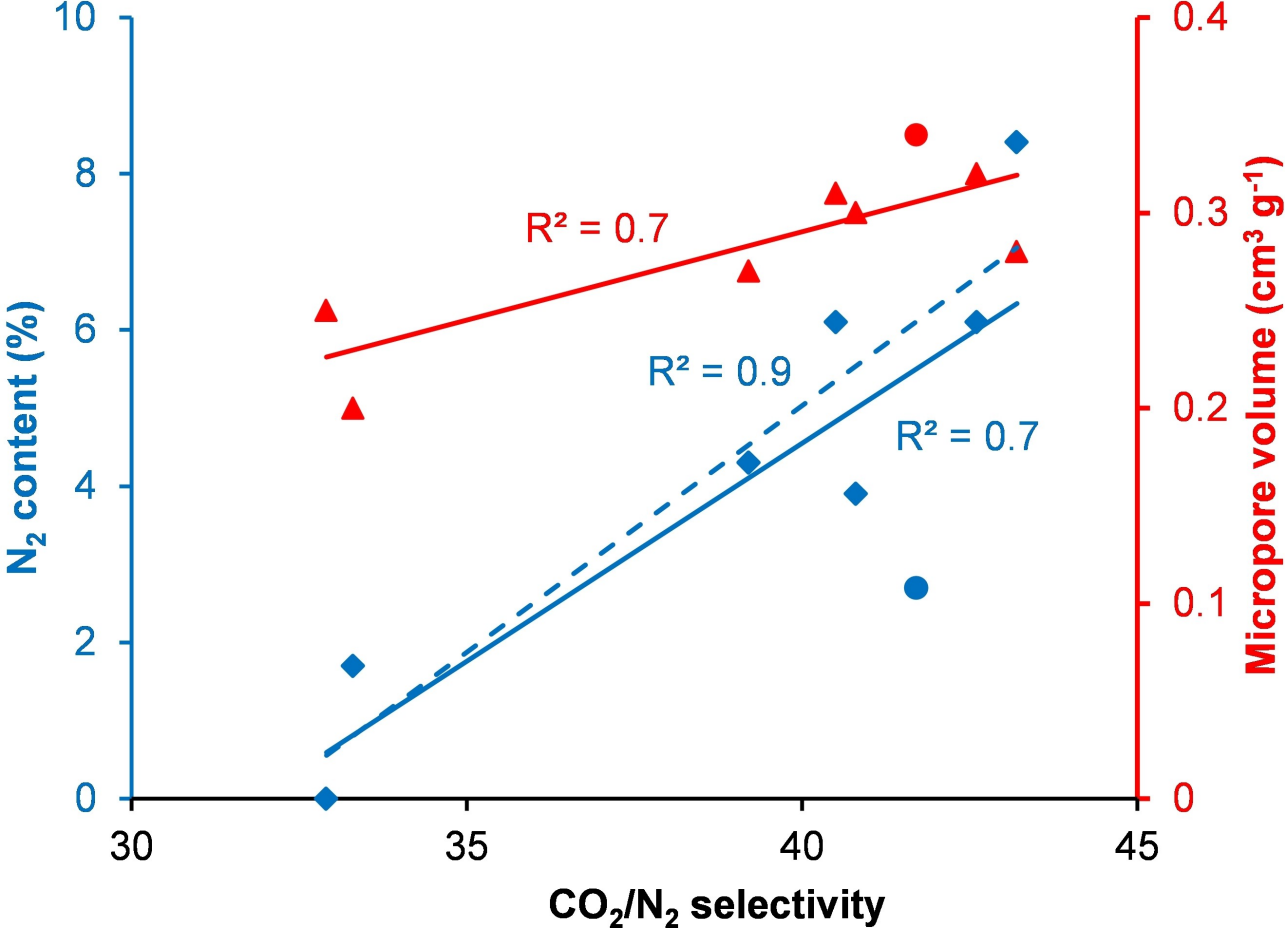
Plots of IAST CO_2_/N_2_ selectivity versus nitrogen content and micropore volumes of Starbon® materials. SN_Gly_800_Mu_ is represented by the blue and red circles. The blue dashed line is the best‐fit line omitting SN_Gly_800_Mu_.

To further investigate the effect of nitrogen incorporation within Starbon® materials on the selectivity for carbon dioxide over nitrogen adsorption, the IAST CO_2_/N_2_ selectivity was plotted against the nitrogen content of the materials (Figure 6). The resulting plot showed a strong correlation between the nitrogen content and the IAST CO_2_/N_2_ selectivity. This is likely due to the ability of lone pairs of electrons associated with the nitrogen atoms to favourably interact with the partially positively charged carbon atom within carbon dioxide.

Relationships between the IAST CO_2_/N_2_ selectivity and the structural properties of the Starbon® materials were also investigated. A strong correlation was found with the micropore volume of the materials (Figure 6), but not with the BET surface area (*R* ^2^=0.5), total pore volume (*R* ^2^=0.4) or mesopore volume (*R* ^2^=0.2). This is consistent with previous results on non‐nitrogen doped Starbons®.^[10]^ Thus, the smallest pores (micropores) provide the most effective discrimination between carbon dioxide and nitrogen. SN_Gly_800_Mu_ is an outlier in Figure 6 (shown by blue and red circles) as it has the third best IAST CO_2_/N_2_ selectivity despite having the third lowest nitrogen content. This is a result of it having the highest micropore volume (0.34 cm^3^ g^−1^) of any of the samples. When SN_Gly_800_Mu_ was omitted from the nitrogen content versus IAST CO_2_/N_2_ selectivity analysis, a very strong correlation (*R* ^2^=0.9) was found for the seven remaining samples (blue dashed line in Figure 6).

**Figure 7:**
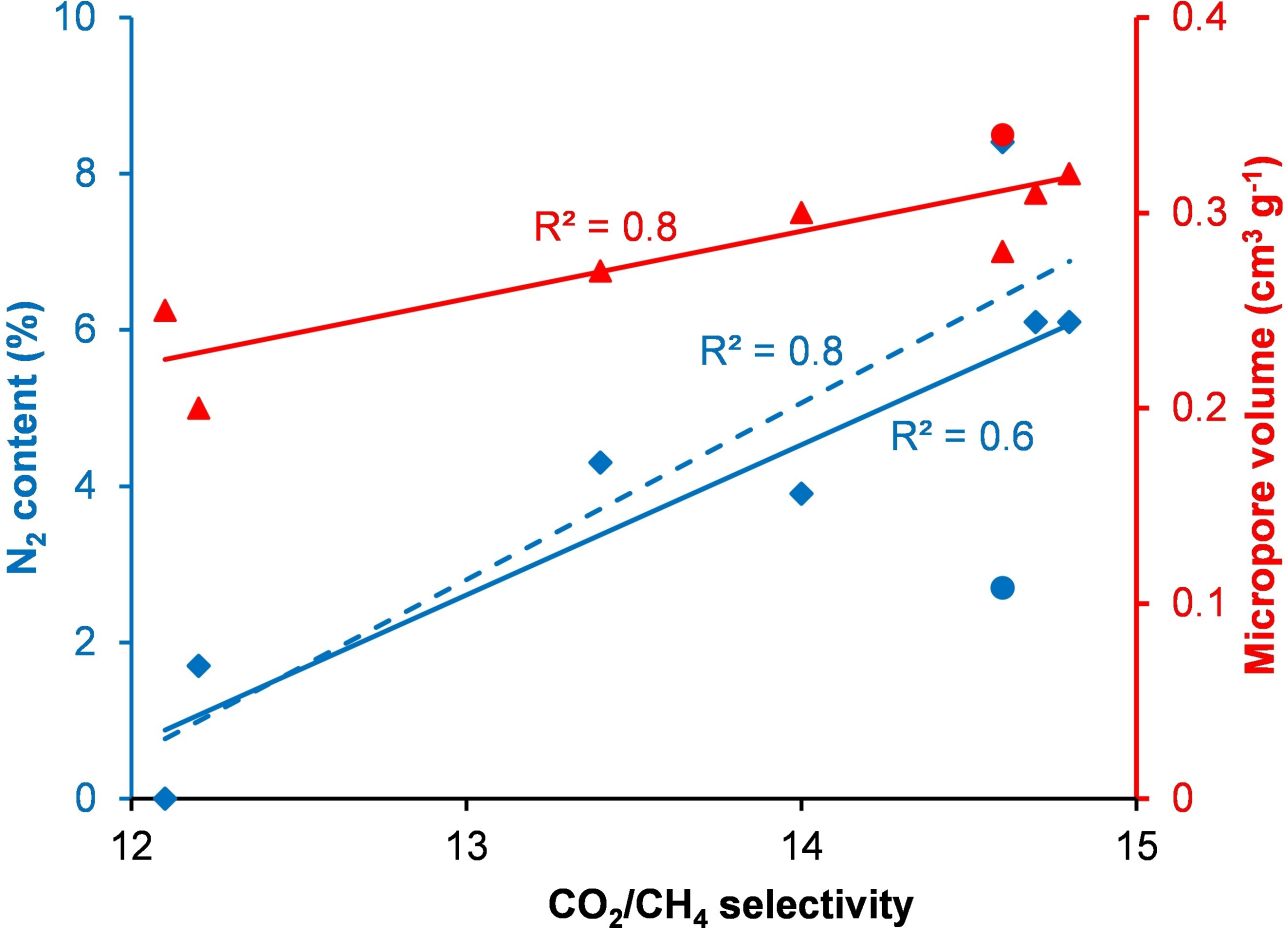
Plots of IAST CO_2_/CH_4_ selectivity versus nitrogen content and micropore volumes of Starbon® materials. SN_Gly_800_Mu_ is represented by the blue and red circles. The blue dashed line is the best‐fit line omitting SN_Gly_800_Mu_.

The IAST selectivities for carbon dioxide over methane (at partial pressures of 0.15 and 0.85 respectively) followed a very similar trend to the carbon dioxide versus nitrogen selectivities, though the absolute values for the selectivities were lower (Table 4). S800 displayed an IAST selectivity of 12.1 and all of the nitrogen‐doped materials had higher selectivities (12.2–14.8). The highest selectivity (14.8) was obtained using SN_Ure_800_Mo_, though this was only marginally higher than the selectivities observed for SN_Gly_800_Mo_ (14.7), SN_Mel_800_Mo_ (14.6) and SN_Gly_800_Mu_ (14.6). The IAST CO_2_/CH_4_ selectivity again showed a strong correlation with the micropore volume of the Starbon® material (Figure 7) along with moderate correlations with the nitrogen content and BET surface area (both *R* ^2^=0.6). There were no significant correlations with the total pore volume (*R* ^2^=0.5) or mesopore volume (*R* ^2^=0.3). Again, the correlation between the nitrogen content of the materials and their IAST CO_2_/CH_4_ selectivity increased (to *R* ^2^=0.8) if SN_Gly_800_Mu_ was omitted as shown by the blue dashed line in Figure 7.

For completeness, the IAST selectivities for nitrogen over methane (at partial pressures of 0.15 and 0.85 respectively) were calculated (Table 4). These showed no significant variation between the Starbon® materials (1.9–2.1), further highlighting the importance of a dipolar interaction between the partially positively charged carbon atom in carbon dioxide and the electron‐rich nitrogen atoms of doped Starbons® in enhancing the carbon dioxide versus nitrogen and carbon dioxide versus methane selectivities.

## Conclusions

Potentially sustainably sourced, nitrogen‐containing solids have been shown to dope S300, producing nitrogen‐doped Starbons®. Traditional thermal heating in vacuo was found to result in loss of much of the nitrogen dopant through vaporisation, so microwave heating methods in sealed systems were investigated. Doping in a monomodal microwave using a mixture of S300 and solid dopant was found to be effective, giving SN_X_800_Mo_ materials containing 3.9–9.0 % nitrogen, compared to the 1.4–7.9 % nitrogen incorporation obtained for the SN_X_800_Th_ materials. Doping in a multimode microwave using solid S300 and an aqueous solution of dopants **1**–**4** was also effective, giving SN_X_800_Mu_ materials containing 2.7–11.2 % nitrogen. However, nicotinamide **5** did not act as a dopant under these conditions. Overall, melamine **4** gave the highest levels of nitrogen incorporation under thermal (7.9 % for SN_Mel_800_Th_) and multimode microwave (11.2 % for SN_Mel_800_Mu_) heating, whilst urea **3** gave the highest level of nitrogen incorporation under monomode microwave heating (9.0 % for SN_Ure_800_Mo_).

The nitrogen‐doped Starbons® were characterised by porosimetry, solid‐state ^13^C NMR spectroscopy, DRIFTS, powder X‐ray diffraction, XPS, SEM/EDX and TEM. These showed that whilst some of the SN_X_300_Y_ materials contained dopant derived material, this was all pyrolysed away during the preparation of the SN_X_800_Y_ materials. The nitrogen‐doped Starbons® were shown to retain the morphology and pore structures associated with undoped Starbons®. For the thermally and monomode microwave produced materials, some of the nitrogen was incorporated as nitrile groups, but no nitriles were detected in materials prepared using the multimode microwave to heat an aqueous suspension of S300 and dopant.

The application of the nitrogen‐doped Starbons® in gas capture applications was investigated using carbon dioxide, methane and nitrogen. The gravimetrically determined carbon dioxide adsorption capacities (at 308 K and 1 bar pressure) of the nitrogen‐doped Starbons® were up to 33 % higher than those of undoped Starbons® (2.54 mmol g^−1^ for SN_Gly_800_Mu_ versus 1.91 mmol g^−1^ for S800). The four highest carbon dioxide adsorption capacities were all observed for materials prepared using the multimode microwave followed by heating to 800 °C. Since the multimode microwave method is the only method of preparation which does not introduce nitrogen in the form of nitriles, this suggests that the chemical nature of the nitrogen dopant does influence the carbon dioxide adsorption capacity of the resulting material.

The seven most promising nitrogen‐doped materials along with S800 were then investigated volumetrically using carbon dioxide, methane and nitrogen to determine their adsorption isotherms. In all cases, the gas adsorption capacities decreased in the order carbon dioxide > methane > nitrogen. This allowed the selectivity of adsorption of pairs of the gases to be determined using ideal adsorbed solution theory and SN_Mel_800_Mo_ was found to have a selectivity for carbon dioxide versus nitrogen of 43.2 (31 % higher than the corresponding selectivity for S800). This, along with the high carbon dioxide adsorption capacity of SN_Mel_800_Mo_ (3.35 mmol g^−1^ determined volumetrically at 298 K and 1 bar pressure) makes SN_Mel_800_Mo_ a promising material to use in post‐combustion carbon dioxide capture applications. A similar trend was seen in the carbon dioxide versus methane selectivities, but all the materials had similar methane versus nitrogen selectivities. This suggests the importance of an interaction between the lone pair of electrons of the nitrogen dopant and the partially positively charged carbon of carbon dioxide in obtaining enhanced selectivities for gas adsorption using these materials.

In summary, this work has produced and characterised a new class of nitrogen‐doped Starbons® using sustainably available nitrogen sources and shown that the materials have potential uses in carbon capture, utilisation and storage applications.

## Experimental Section

For details of instrumentation see Supporting Information. S300 and S800 were prepared as previously reported.^[10]^

### General procedure for the synthesis of SN_x_300_Th_

S300 was ground with one of dopants **1**–**5** (1 : 1 mass ratio unless otherwise stated) for approx. 3 min. The ground material was then heated to 300 °C in a vacuum furnace, at a pressure of less than 20 mbar. From room temperature to 280 °C, the rate of temperature increase was 5 °C per minute and from 280–300 °C the rate of temperature increase was 0.1 °C per minute. The sample was held at 300 °C for 5 h. After cooling to room temperature, part of the sample (approx. 1 g) was thoroughly washed with approx. 2 L of deionised water, dried under suction, then dried further in a vacuum oven at 80 °C for 16 h to give samples of SN_x_300_Th_. Typically, 0.9 g of SN_x_300_Th_ is obtained from each gram of S300 used.

### General procedure for the synthesis of SN_x_800_Th_

Unwashed SN_x_300_Th_ prepared as described above was heated to 800 °C in a vacuum furnace, at a pressure of less than 20 mbar. From room temperature to 280 °C, the rate of temperature increase was 5 °C per minute; from 280–300 °C the rate of temperature increase was 0.1 °C per minute; from 300–400 °C the rate of temperature increase was 0.3 °C per minute; from 400–600 °C the rate of temperature increase was 1 °C per minute and from 600–800 °C the rate of temperature increase was 3 °C per minute. After cooling to room temperature, samples of SN_x_800_Th_ were obtained. Typically, 0.7 g of SN_x_800_Th_ is obtained from each gram of S300 used.

### General procedure for the synthesis of SN_x_300_Mo_ and SN_x_800_Mo_

S300 was ground with one of dopants **1**–**5** (1 : 1 mass ratio unless otherwise stated) for approx. 3 min. The ground material was then sealed in a quartz tube (35 mL) and heated to 300 °C in a CEM Discover CP microwave using a maximum power setting of 300 W and a maximum allowed pressure of 300 PSI. Hold times of 2 min. were programmed when the temperature reached 200 and 250 °C and a final hold time of 20 min. was programmed at 300 °C. After cooling to room temperature, the material was thoroughly washed with approx. 2 L of solvent (deionised water unless otherwise stated) and dried under suction, then dried further in a vacuum oven at 80 °C for 16 h to give samples of SN_x_300_Mo_. Typically, 1.0 g of SN_x_300_Mo_ is obtained from each gram of S300 used. Part of each sample was heated to 800 °C in a vacuum furnace as described above for the synthesis of SN_x_800_Th_ to give samples of SN_x_800_Mo_. Typically, 0.6 g of SN_x_800_Mo_ is obtained from each gram of S300 used.

### General procedure for the synthesis of SN_x_300_Mu_ and SN_x_800_Mu_

Nitrogen dopant **1**–**5** (5.0 g, 10 equiv.) was added to water (200 mL) in a TFM plastic microwave vessel and stirred for approximately 5 min. S300 (0.5 g, 1 equiv.) was then added to the vessel which was sealed and placed in a Milestone SynthWAVE microwave and was heated to 250 °C over a period of 15 min, then held at 250 °C for 30 min. After cooling to room temperature, the material was thoroughly washed with approx. 2 L of deionised water and dried under suction, then dried further in a vacuum oven at 80 °C for 16 h to give samples of SN_x_300_Mu_. Typically, 1.0 g of SN_x_300_Mu_ is obtained from each gram of S300 used. Part of each sample was heated to 800 °C in a vacuum furnace as described above for the synthesis of SN_x_800_Th_ to give samples of SN_x_800_Mu_. Typically, 0.5 g of SN_x_800_Mu_ is obtained from each gram of S300 used.

### CO_2_ adsorption capacities by thermogravimetric analysis

A Starbon® material (ca. 10 mg) was placed in a simultaneous thermal analyser and heated under a flow of N_2_ (60 mL min^−1^) from room temperature to 413 K at 10 °C per minute, then held at 413 K for 60 min to remove any water and other volatiles from the material. The sample was cooled to room temperature, then heated to 308 K at 1 °C per minute still under the flow of N_2_. The gas flow was switched from N_2_ to CO_2_ whilst maintaining the flow rate at (60 mL min^−1^). The resulting heat flow and mass change due to CO_2_ adsorption were recorded. Once the heat flow had returned to a steady value, the gas flow was switched from CO_2_ back to N_2_. The adsorption and desorption cycles were repeated five times to allow average values and standard deviations to be calculated for each Starbon® material. This procedure was repeated using non‐porous alumina (ca. 10 mg) instead of Starbon® to determine the buoyancy effect^[38]^ associated with changing the gas between nitrogen and carbon dioxide. This buoyancy effect was found to be 1.3±0.1 % and all Starbon® mass changes were adjusted by this amount before calculating the carbon dioxide adsorption capacities from the measured mass changes.

### Gas adsorption capacities by volumetric analysis

The Starbon material (0.06–0.09 g) was degassed at 150 °C for 15 h under dynamic vacuum before the carbon dioxide, nitrogen and methane adsorption and desorption isotherms were measured at 298 K over a pressure range of 1–1137 mbar with an equilibration interval of 30–35 s after measurements at each of 31 pressures.

## Supporting Information

Additional references cited within the Supporting Information.[^39^, ^40^]

## Conflict of interest

The authors declare no conflict of interest.

1

## Supporting information

As a service to our authors and readers, this journal provides supporting information supplied by the authors. Such materials are peer reviewed and may be re‐organized for online delivery, but are not copy‐edited or typeset. Technical support issues arising from supporting information (other than missing files) should be addressed to the authors.

Supporting Information

## Data Availability

The data that support the findings of this study are available in the supplementary material of this article.
