# Supplementary material for: Nitrogen‐Doped Starbons®: Methodology Development and Carbon Dioxide Capture Capability
Source: Chemistry. 2023 Dec 7;30(6):e202303436. doi: 10.1002/chem.202303436 (PMC10952171; doi:10.1002/chem.202303436)

Chemistry–A European Journal

Supporting Information

Nitrogen-Doped Starbons®: Methodology Development and Carbon Dioxide Capture Capability

Ryan E. Barker, Michael C. Brand, James H. Clark,* and Michael North*

Contents

General experimental details	3
TGA and DSC traces for dopants **1–5**	5
DRIFTS spectra of dopants **1–5**	10
Powder X-ray diffraction data for dopants **1–5** and nicotinic acid	15
Solid-state ^{13}C NMR spectra of dopants **1–5**	18
Porosimetry data for S300	23
Porosimetry data for SN_x300_{Th}	24
Porosimetry data for SN_x300_{Mo}	29
Porosimetry data for SN_x300_{Mu}	34
Porosimetry data for S800	39
Porosimetry data for SN_x800_{Th}	40
Porosimetry data for SN_x800_{Mo}	46
Porosimetry data for SN_x800_{Mu}	51
Combustion and XPS analysis of S300	56
Combustion and XPS analysis of S800	57
Combustion and XPS analysis of SN_x300_{Th}	58
Combustion and XPS analysis of SN_x800_{Th}	63
Combustion and XPS analysis of SN_x300_{Mo}	68
Combustion analysis of SN_x800_{Mo}	73
Combustion and XPS analysis of SN_x300_{Mu}	74
Combustion analysis of SN_x800_{Mu}	79
Powder X-ray diffraction data on S300 and SN_X300_{Th} samples	80
Powder X-ray diffraction data on S800 and SN_X800_{Th} samples	83
Powder X-ray diffraction data on SN_X300_{Mo} samples	86
Powder X-ray diffraction data on SN_X300_{Mu} samples	89
Powder X-ray diffraction data on SN_X800_{Mo} samples	92
Powder X-ray diffraction data on SN_X800_{Mu} samples	95
Solid-state ^{13}C NMR spectrum of S300	98
Solid-state ^{13}C NMR spectra of SN_x300_{Th}	99
Solid-state ^{13}C NMR spectra of SN_x300_{Mo}	104
Solid-state ^{13}C NMR spectra of SN_x300_{Mu}	109
SEM images of S300	114
SEM images of S800	115
SEM images and nitrogen EDX mapping of SN_x300_{Th}	116
SEM images and nitrogen EDX mapping of SN_x800_{Th}	121
SEM images and nitrogen EDX mapping of SN_x300_{Mo}	126
SEM images and nitrogen EDX mapping of SN_x300_{Mu}	131
TEM images of S300 and S800	137
TEM images of $SN_{Gly}300_{Th}$ and $SN_{Gly}800_{Th}$	138
TEM images of $SN_{Bal}300_{Th}$ and $SN_{Bal}800_{Th}$	139
TEM images of $SN_{Ure}300_{Th}$ and $SN_{Ure}800_{Th}$	140
TEM images of $SN_{Mel}300_{Th}$ and $SN_{Mel}800_{Th}$	141

TEM images of $SN_{Nic}300_{Th}$ and $SN_{Nic}800_{Th}$ 142
TEM images of SN_X300_{Mo} 143
TEM images of SN_X300_{Mu} 146

DRIFTS spectra of SN_x300_{Th} 149
DRIFTS spectra of SN_x300_{Mo} 154
DRIFTS spectra of SN_x300_{Mu} 159

Thermogravimetric analysis of CO_2 adsorption onto S300 and S800 164
Thermogravimetric analysis of CO_2 adsorption onto SN_X300_y 166
Thermogravimetric analysis of CO_2 adsorption onto SN_X800_y 181

CO_2 adsorption isotherms onto S800 and SN_X800_y 196
N_2 adsorption isotherms onto S800 and SN_X800_y 204
CH_4 adsorption isotherms onto S800 and SN_X800_y 212

General experimental details

All chemicals were purchased and used without modification. Starch (Hylon VII, approx. 70% amylose) was purchased from Ingredion. Glycine (≥99%) and nicotinamide (≥99.5%) were purchased from Sigma. β-Alanine (98%) was purchased from Aldrich. Urea (analytical reagent grade) was purchased from Fisher Scientific. Melamine (99%) was purchased from Acros Organics. Deionised water was obtained from a SUEZ water purification system.

For thermal analysis, the compound (approx. 10 mg) was placed in a Stanton Redcroft STA 625 simultaneous thermal analyser and was heated at a rate of 5 °C min^{-1} from room temperature to 625 °C under N_2 gas, with the mass change and heat flow data being recorded. The same STA 625 instrument was used to thermogravimetrically determine CO_2 adsorption capacities. Combustion analysis for carbon, hydrogen and nitrogen was performed using an Exeter Analytical Inc. CE-440 analyser in conjunction with a Sartorius SE2 analytical balance. XPS was performed by the EPSRC national service at the University of Wales, Cardiff on a Thermo Fisher Scientific K-alpha+ spectrometer. Samples were analysed using a micro-focused monochromatic Al X-ray source operating at 6 mA x 12 kV (72 W). Data was recorded at pass energies of 150 eV for survey scans and 40 eV for high resolution scan with 1 eV and 0.1 eV step sizes respectively. Charge neutralisation, where required, was achieved using a combination of both low energy electrons and argon ions. Data analysis was performed in CasaXPS v2.3.25,[39] using a Shirley type background and Scofield cross sections, with an electron energy dependence derived by the TPP-2M equation.[40]

Powder X-ray crystallography was performed on one of two instruments. Most samples were analysed on a Bruker D8 Advance X-ray diffractometer equipped with a 2 kW copper source and a LynxEye detector. The instrument was operated in reflectance mode. Scan range was 2θ from 5.00 to 89.98°, with a 0.01° step and a step time of 264.5 s. SN_x800_{Mo} and SN_x800_{Mu} samples were analysed on a Panalytical Aeris X-ray diffractometer equipped with a 600 W copper source and a PIXcel1D-Medipix3 detector. The instrument was operated in reflectance mode. Scan range was 2θ from 5.00 to 70.00°, with a scan rate of 0.005° s^{-1}.

Solid state NMR was run on a Bruker AVIII HD 400 operating at 100 MHz with magic angle spinning at 54.74° at a spin rate of 12,000 Hz. Up to 32,000 scans of each sample were recorded with an 8 s delay between each scan. Diffuse reflectance infrared Fourier transform spectroscopy (DRIFTS) was performed on a Bruker Equinox 55 spectrometer with a scanning range of 500 to 4000 cm^{-1}.

Porosimetry analysis was carried out on a Micromeritics ASAP 2020 volumetric adsorption analyser. Samples (approx. 120 mg) were first degassed under vacuum whilst being heated at 150 °C over period of at least 6 hours. The nitrogen adsorption-desorption isotherm was then collected at 77 K. Volumetric gas analysis was carried out using a Micromeritics 2050 volumetric adsorption analyser at 298 K, stabilised using a circulating water chiller/heater.

SEM and EDX images were obtained on a JEOL 7800F prime electron microscope with an Oxford Instruments UltiMAX 100 (square-millimetre chip) EDS system at the York JEOL Nanocentre of the University of York. The Starbon® material was attached to an aluminium stub using a double sided adhesive pad which was subsequently loaded into the microscope and subjected to a 15 keV electron beam at a 10 mm working distance. TEM images were obtained on a JEOL 2010+ transmission electron microscope at the York JEOL Nanocentre of the

University of York. The Starbon® material was sonicated in an ethanol solution until a suspension was formed. A few drops of the suspension were pipetted onto a lacey carbon copper TEM grid which was then loaded into the microscope and analysed.

TGA and DSC traces for glycine 1

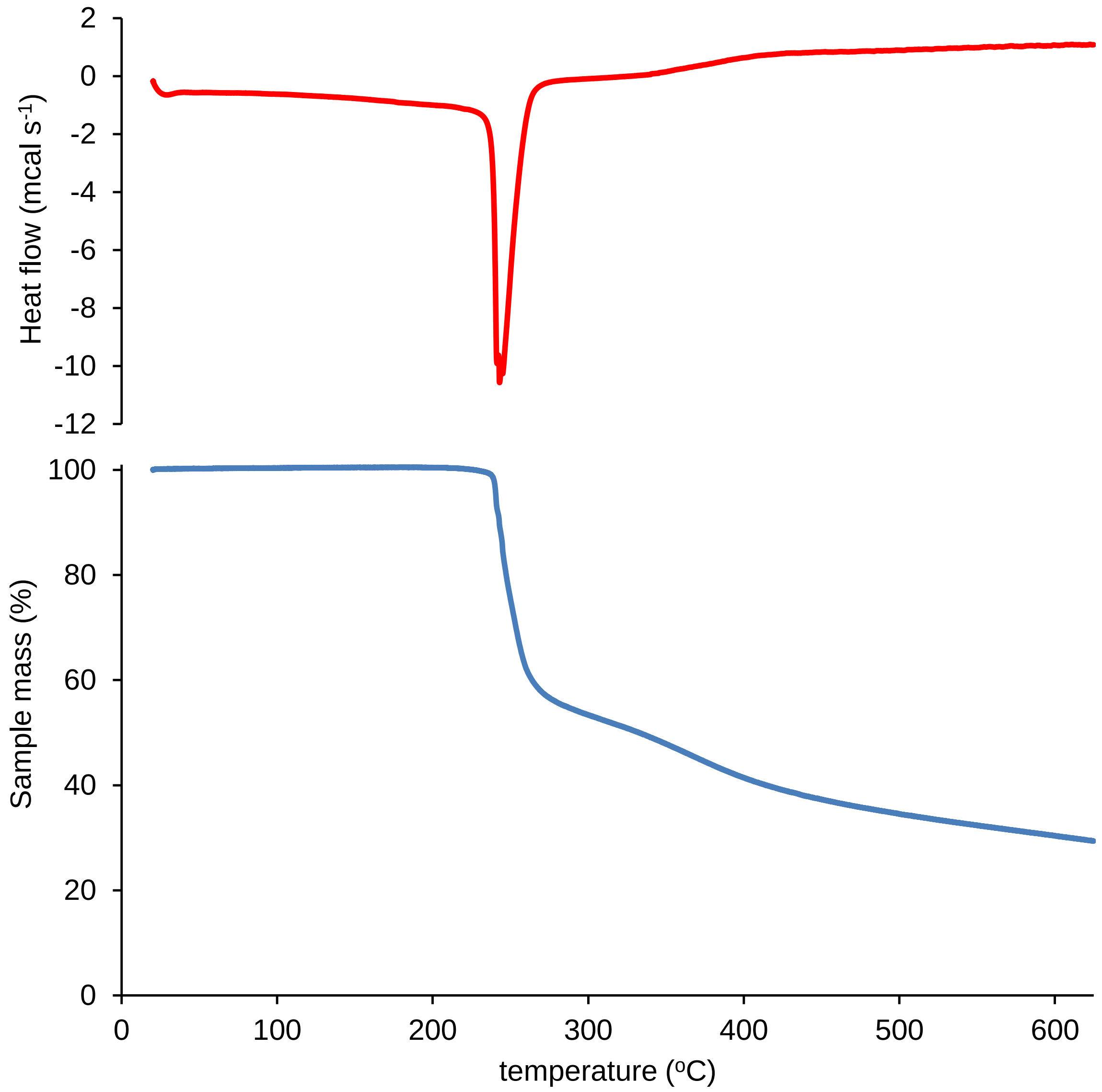

TGA and DSC traces for β-alanine 2

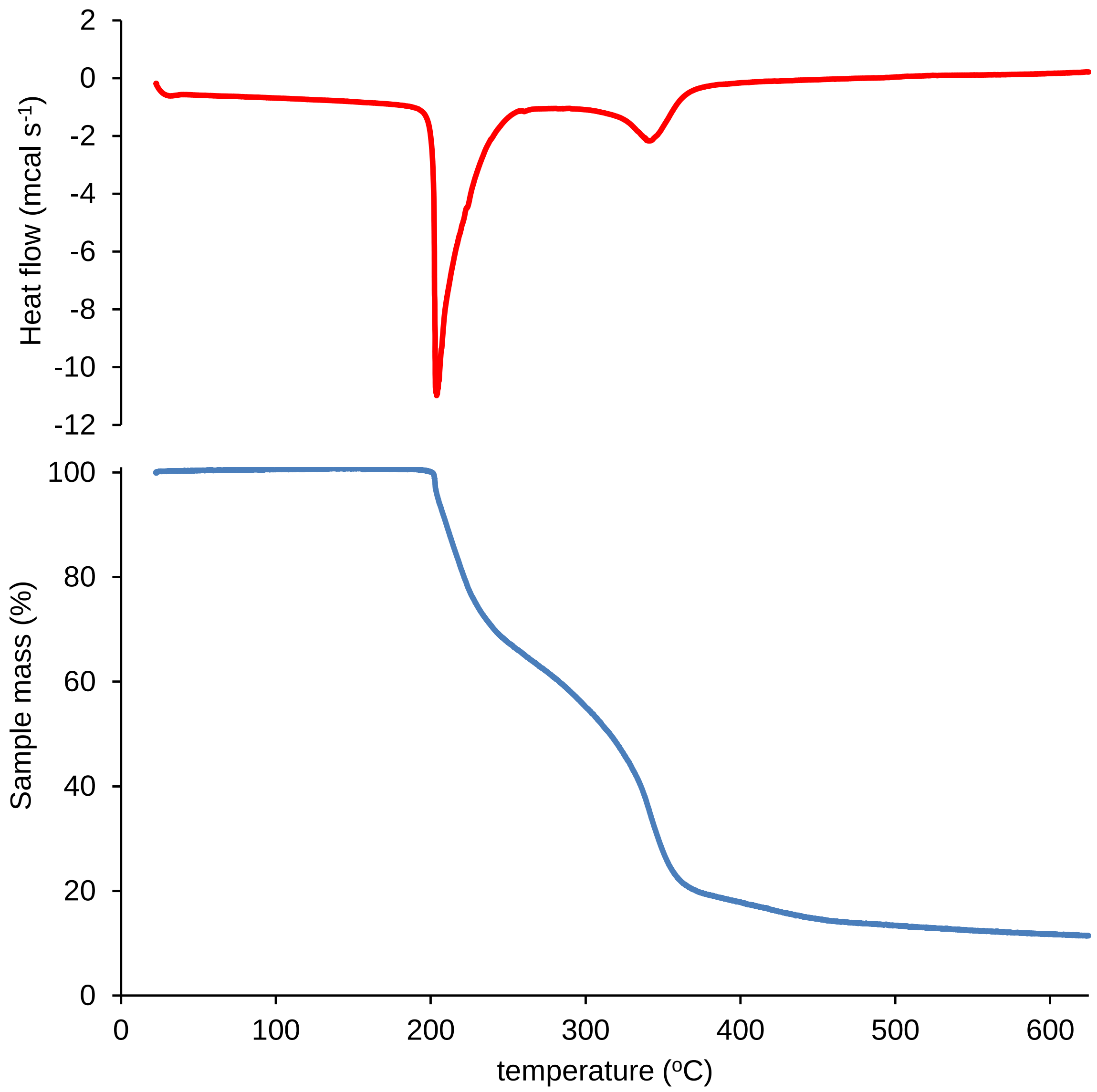

TGA and DSC traces for urea 3

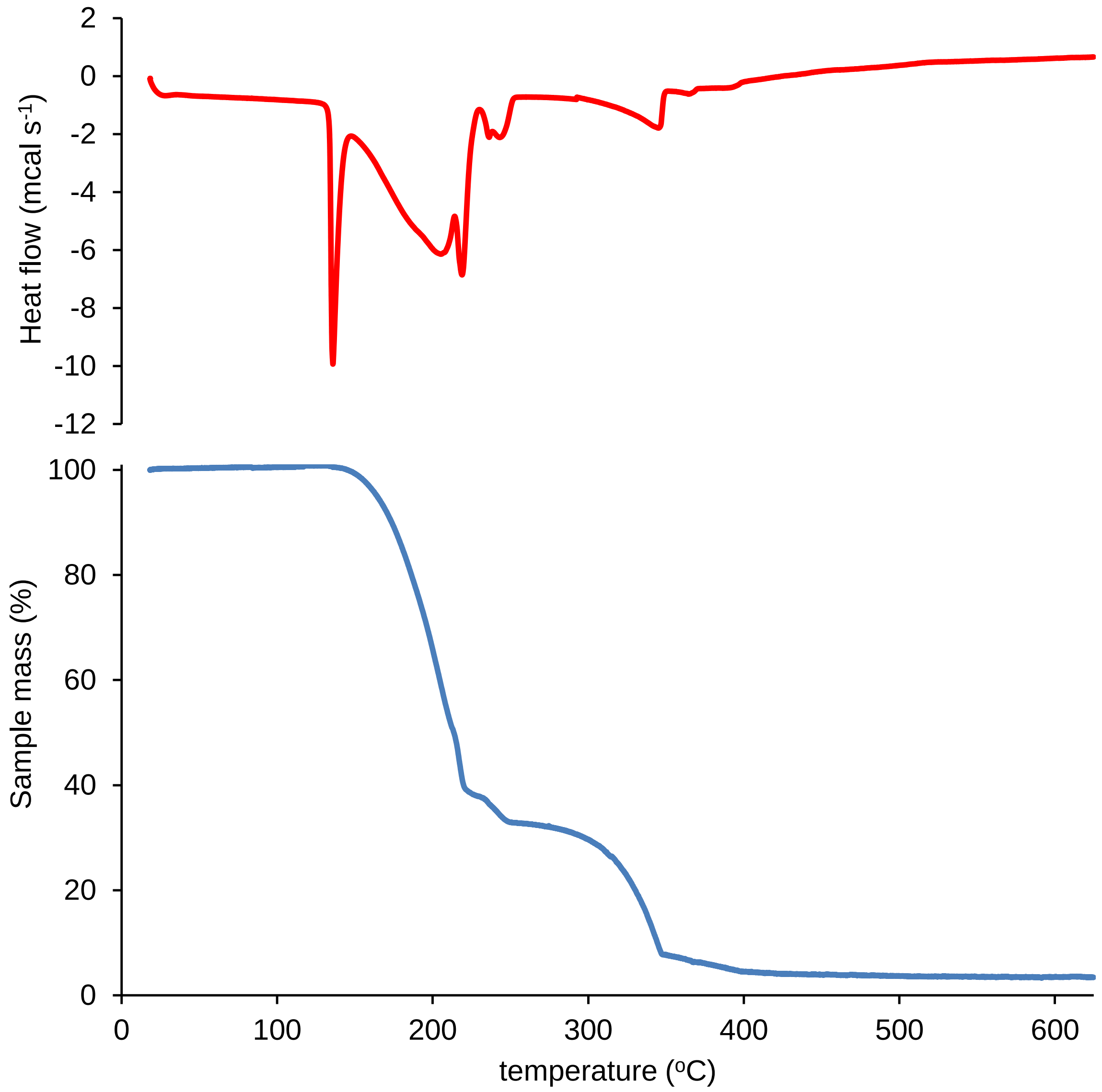

TGA and DSC traces for melamine 4

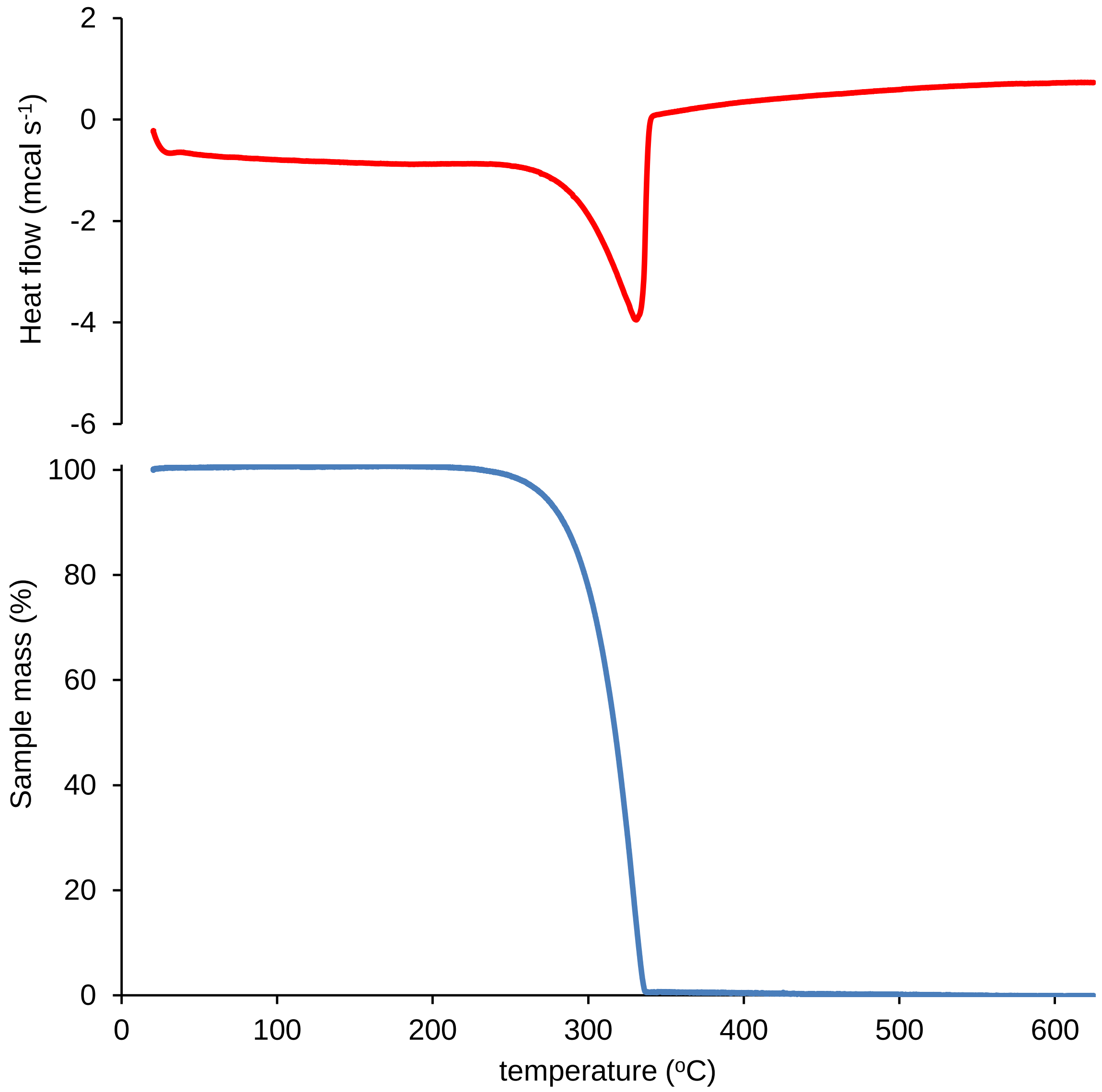

TGA and DSC traces for nicotinamide 5

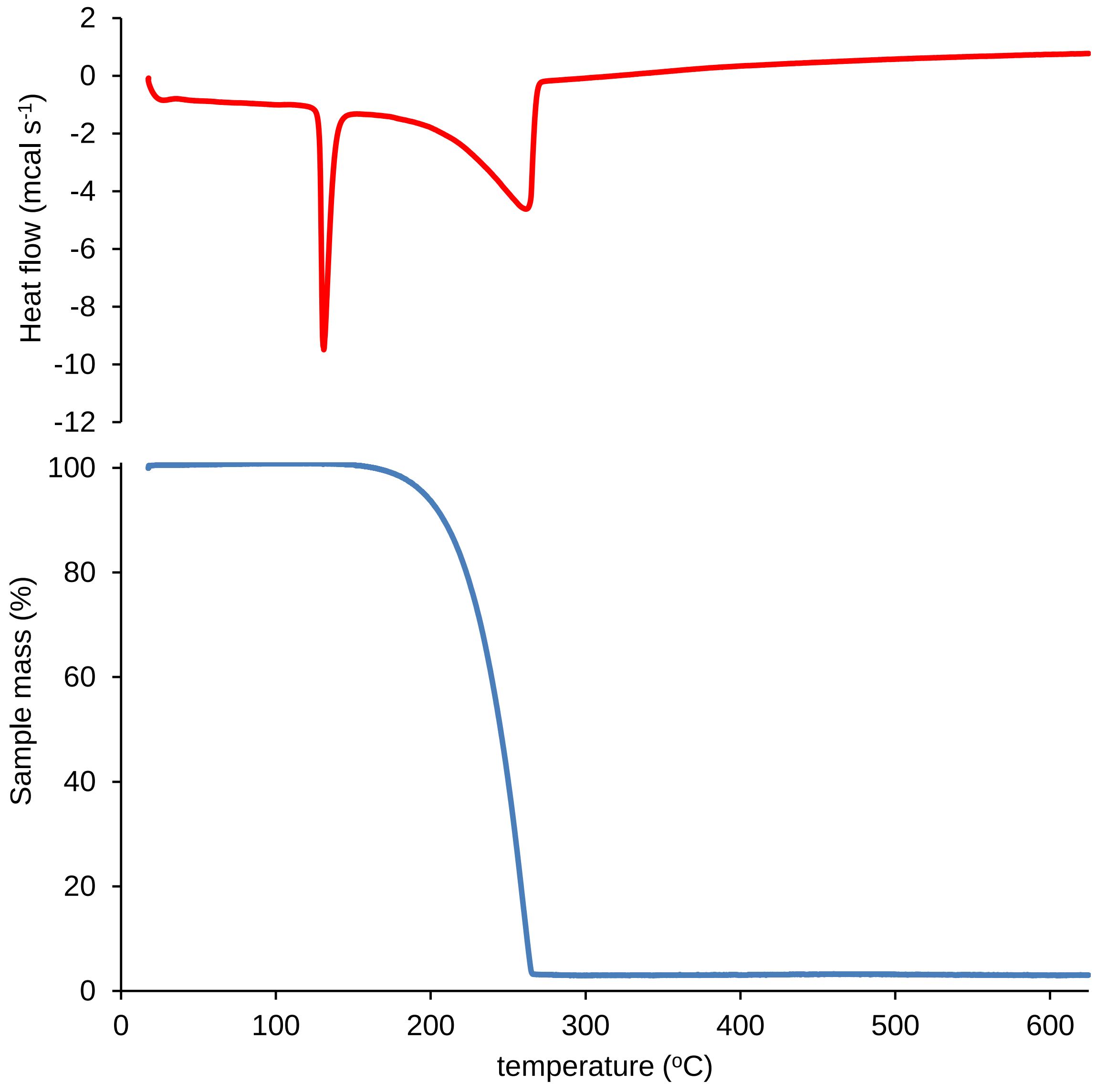

DRIFTS spectrum of glycine 1

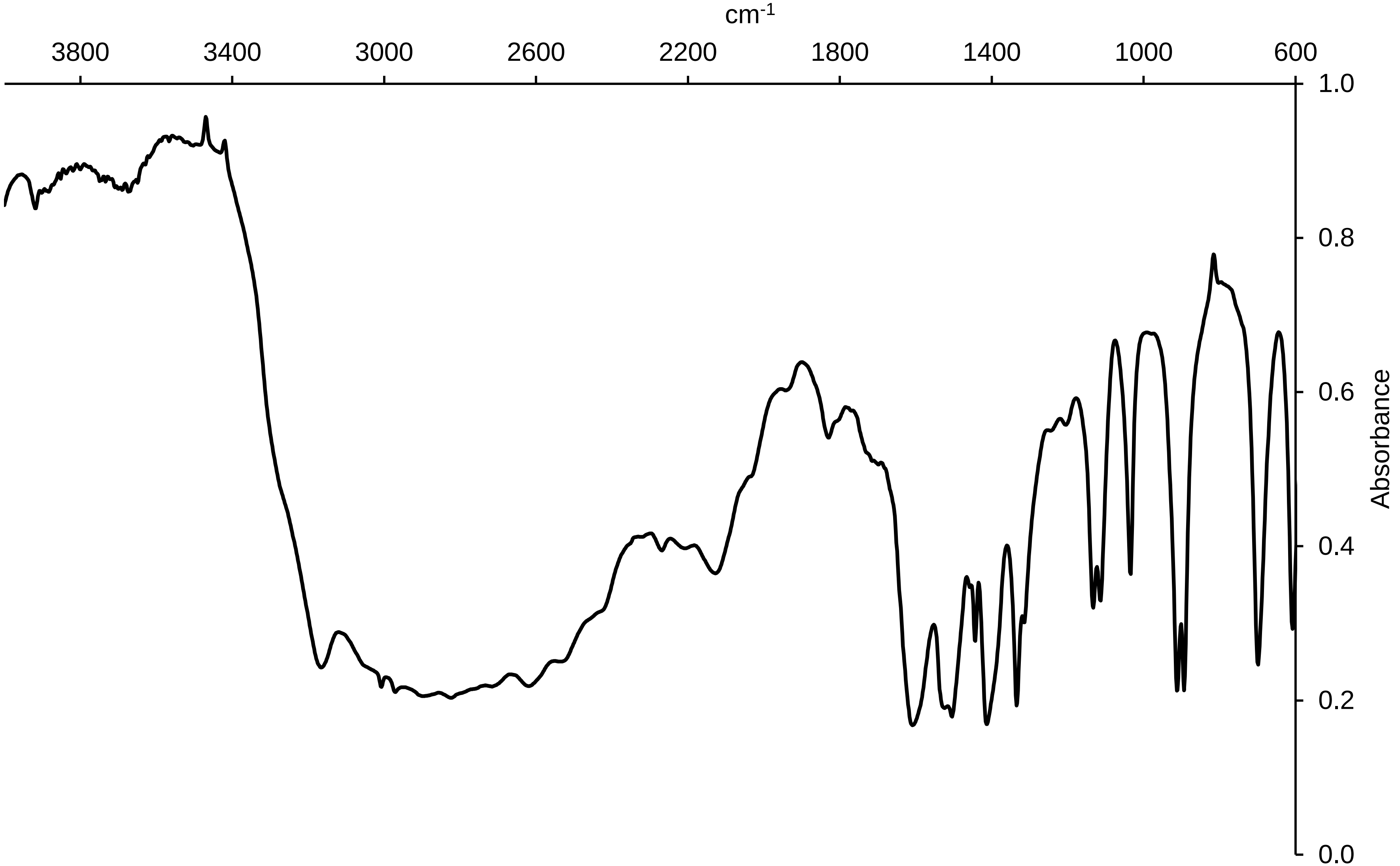

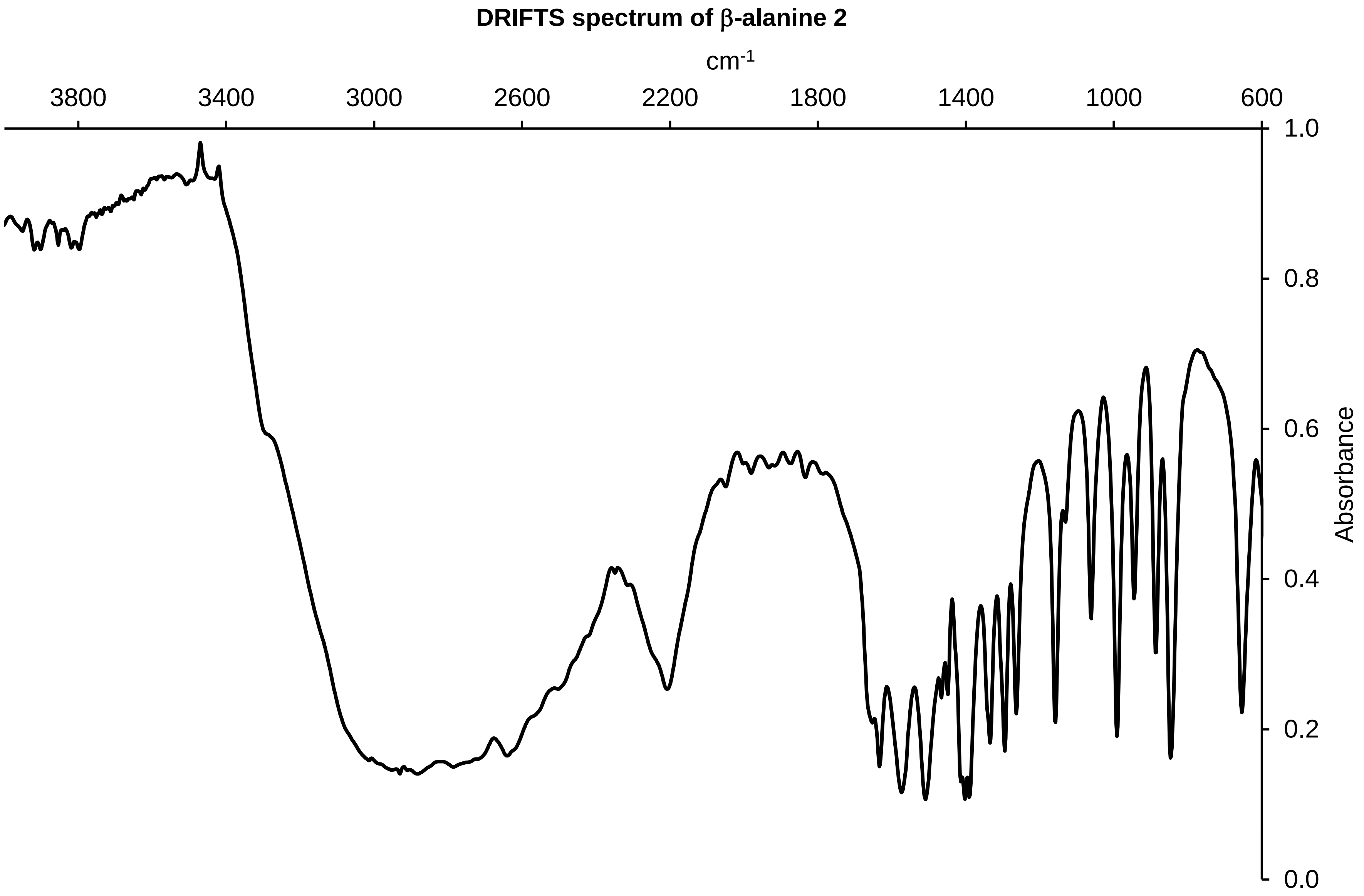
DRIFTS spectrum of β-alanine 2
cm-1
3800
3400
3000
2600
2200
1800
1400
1000
600
1.0
0.8
0.6
0.4
0.2
0.0
Absorbance

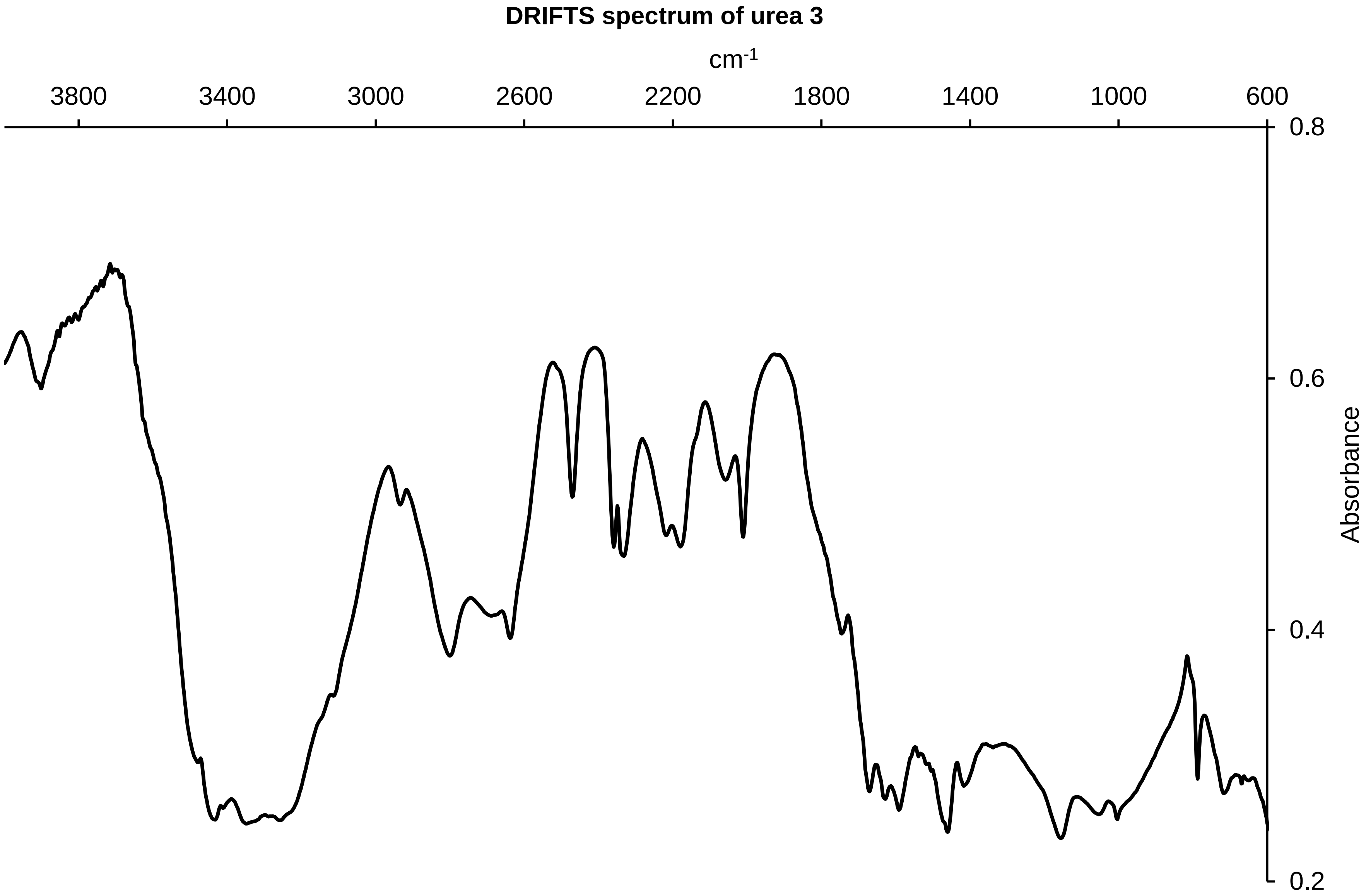

DRIFTS spectrum of urea 3
cm-1
3800
3400
3000
2600
2200
1800
1400
1000
600
0.8
0.6
0.4
0.2
Absorbance

DRIFTS spectrum of melamine 4

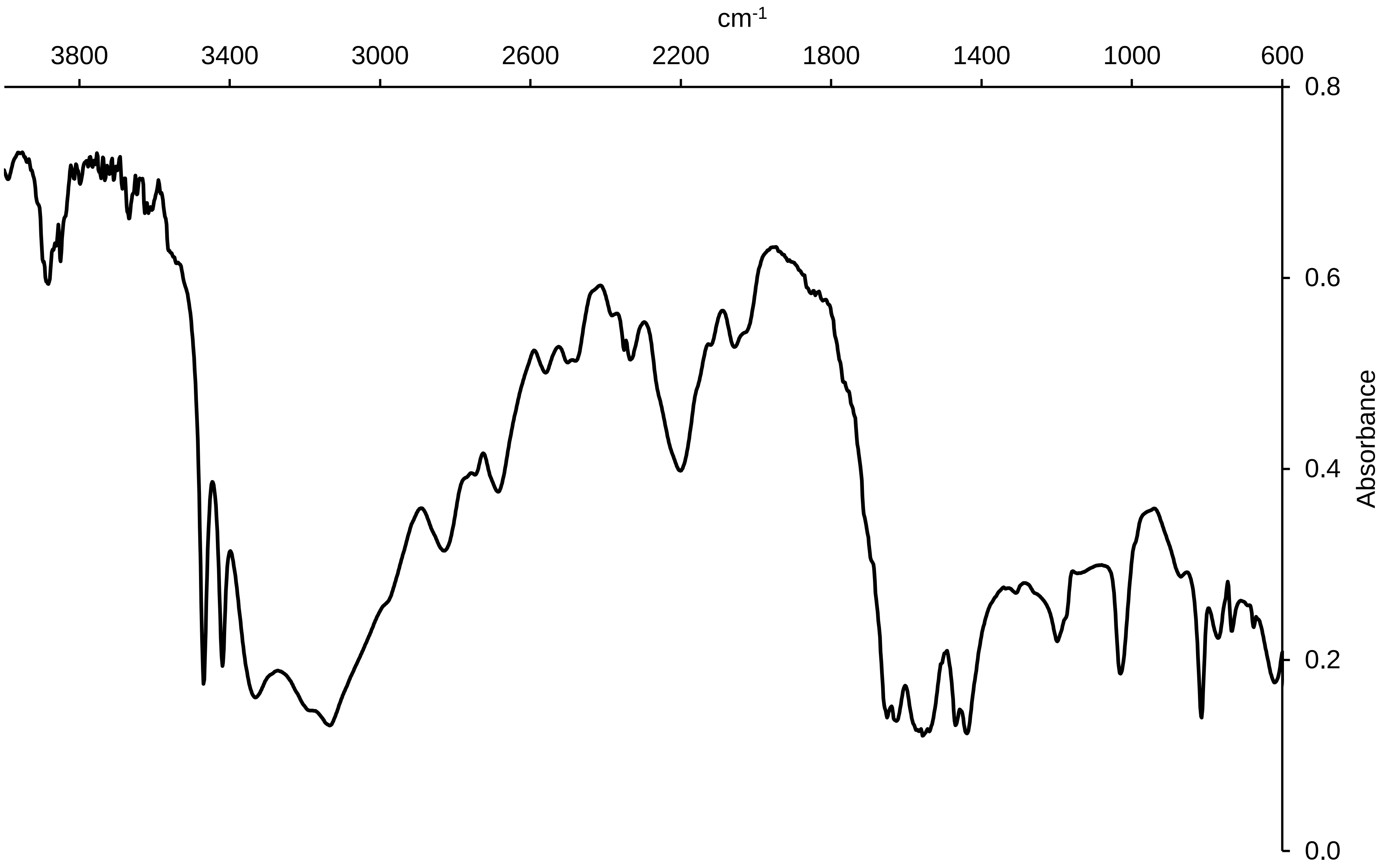

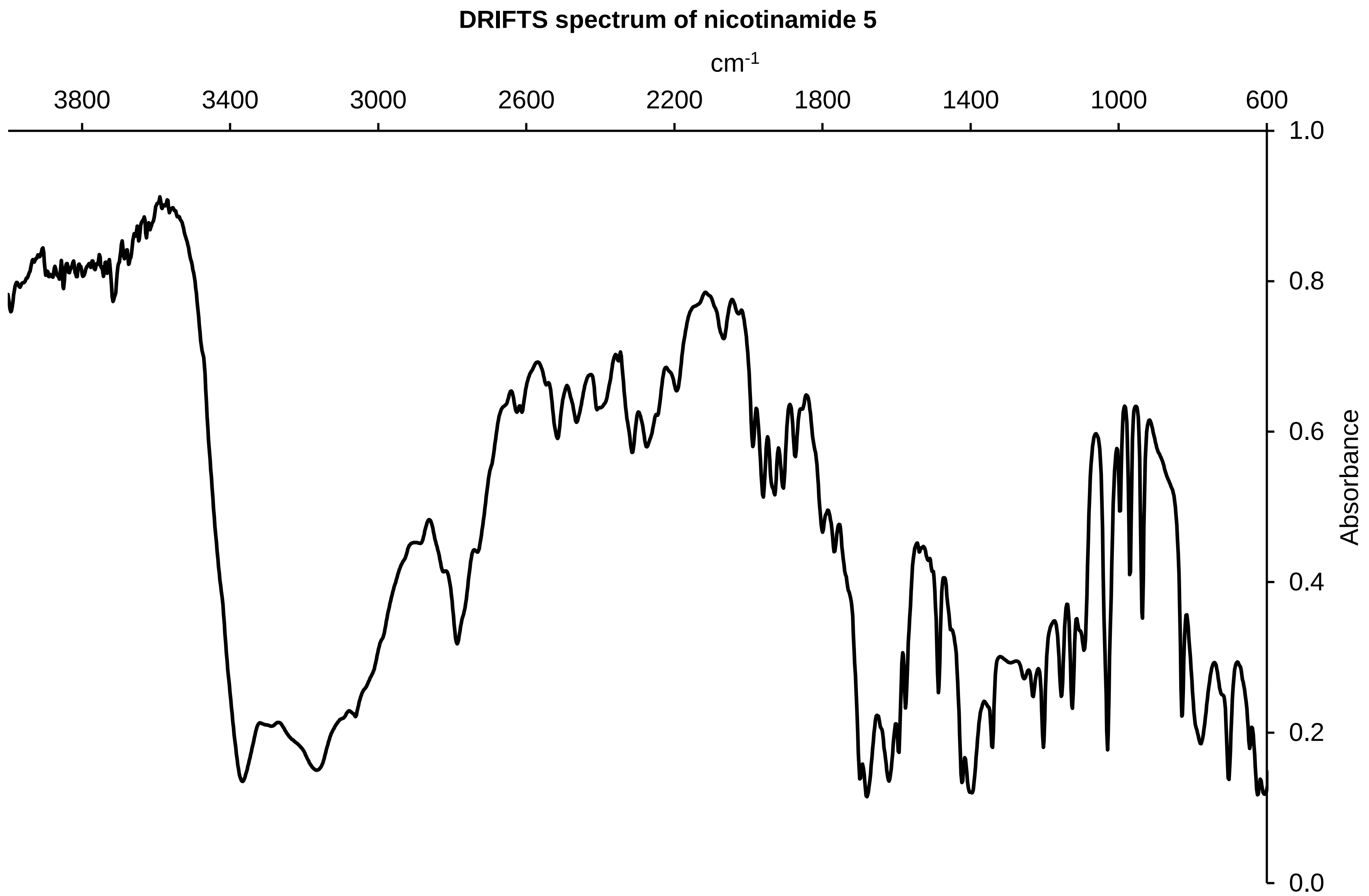
DRIFTS spectrum of nicotinamide 5
cm-1
3800
3400
3000
2600
2200
1800
1400
1000
600
1.0
0.8
0.6
0.4
0.2
0.0
Absorbance

Powder X-ray diffraction data for dopants 1–5 and nicotinic acid

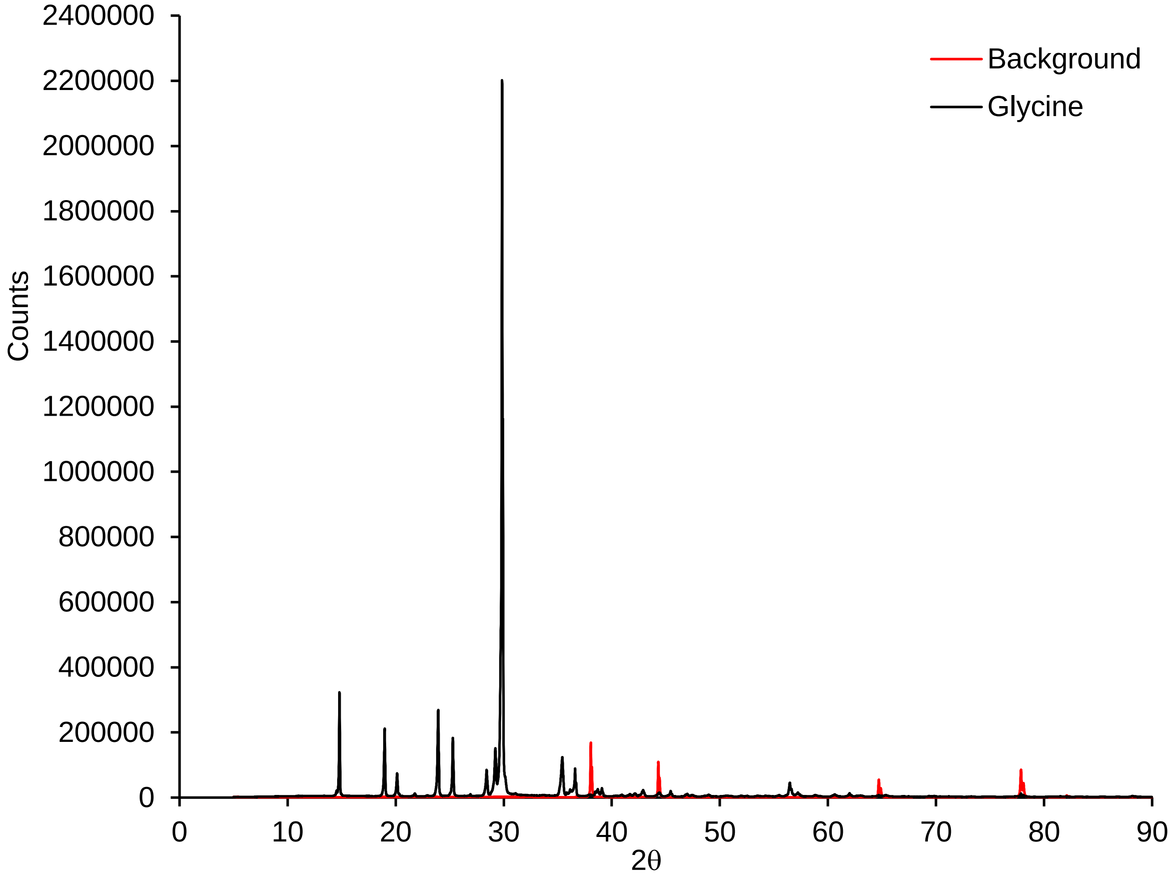

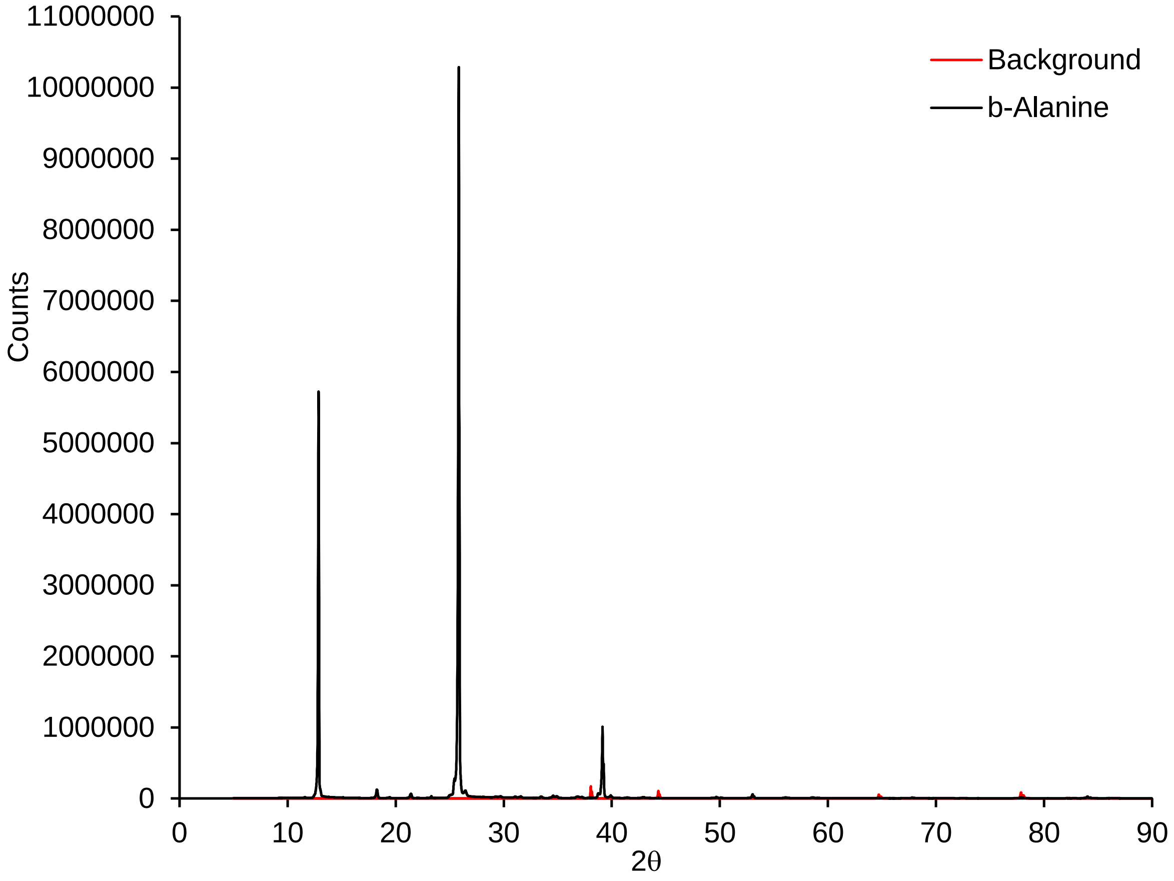

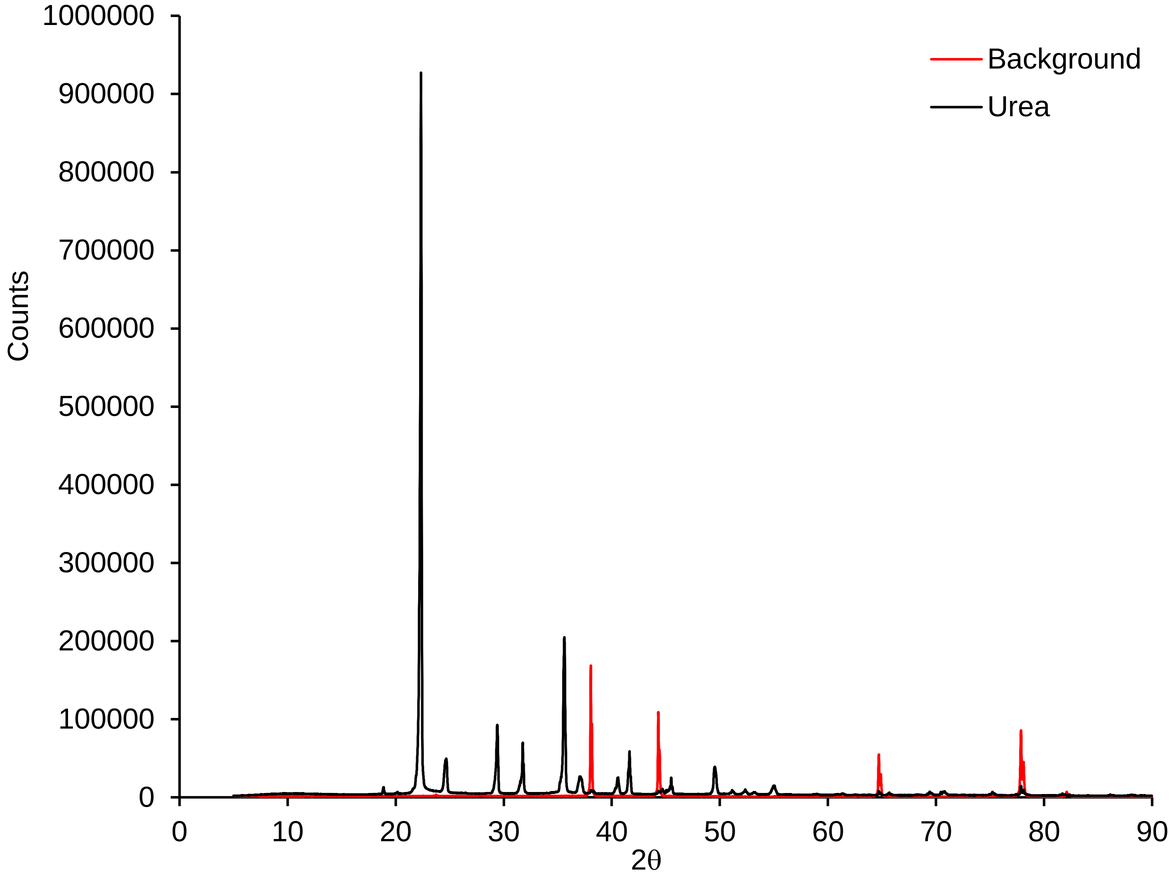
Background
Urea
Counts
2θ
1000000
900000
800000
700000
600000
500000
400000
300000
200000
100000
0
0
10
20
30
40
50
60
70
80
90

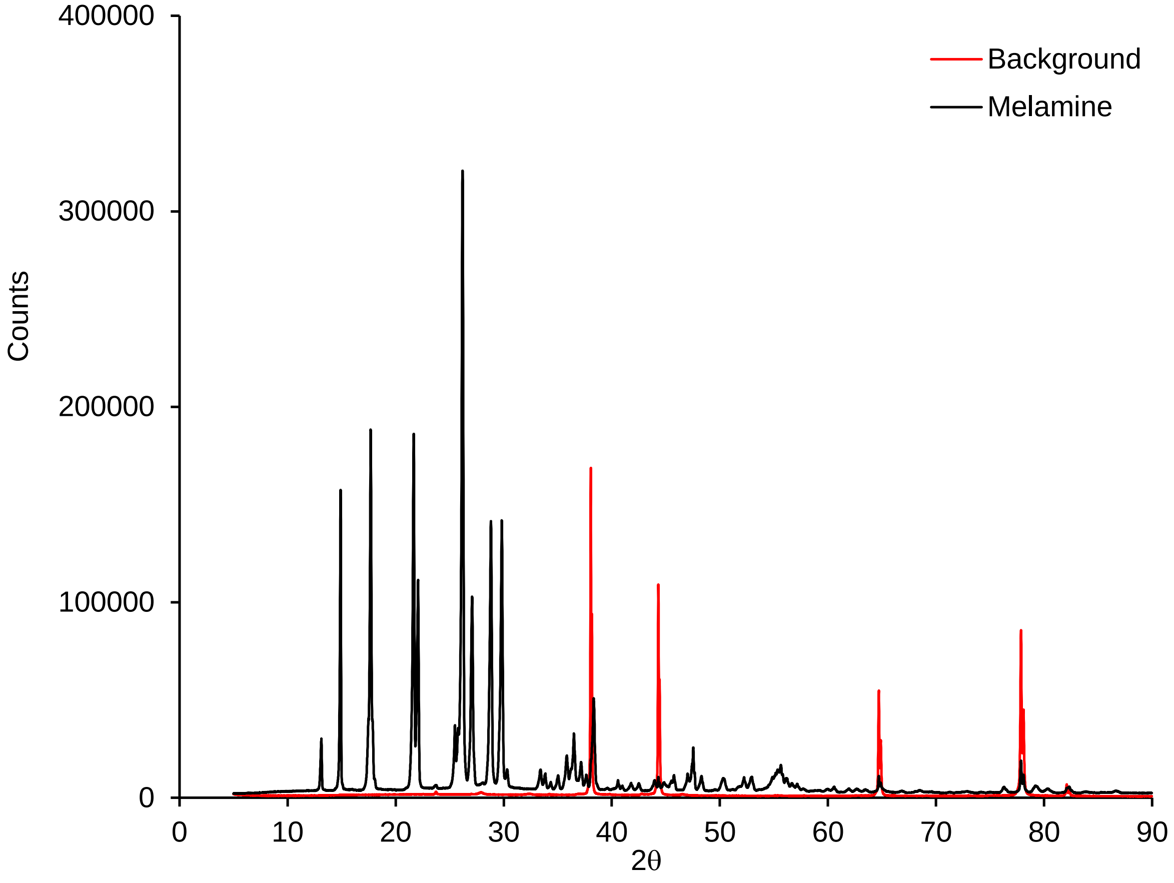
Background
Melamine
Counts
2θ
400000
300000
200000
100000
0
0
10
20
30
40
50
60
70
80
90

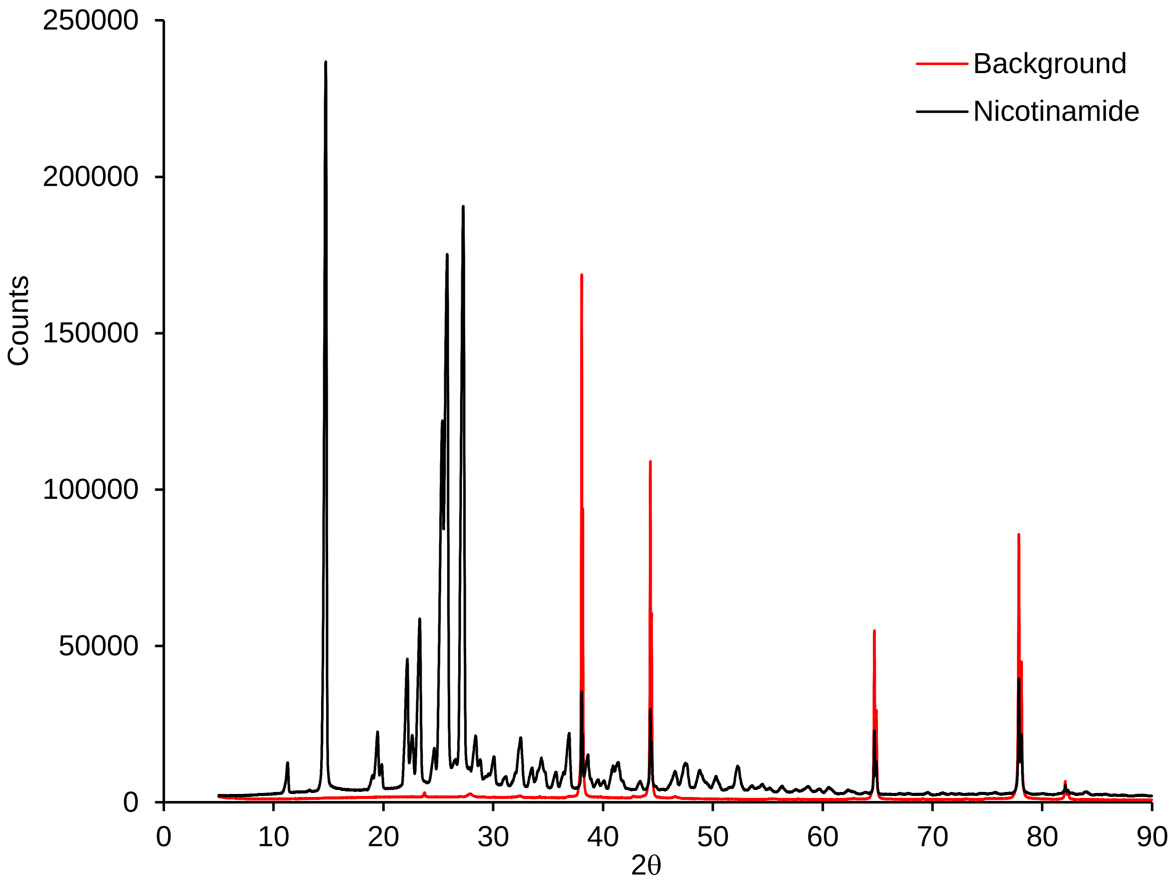
Background
Nicotinamide
Counts
250000
200000
150000
100000
50000
0
0
10
20
30
40
50
60
70
80
90
2θ

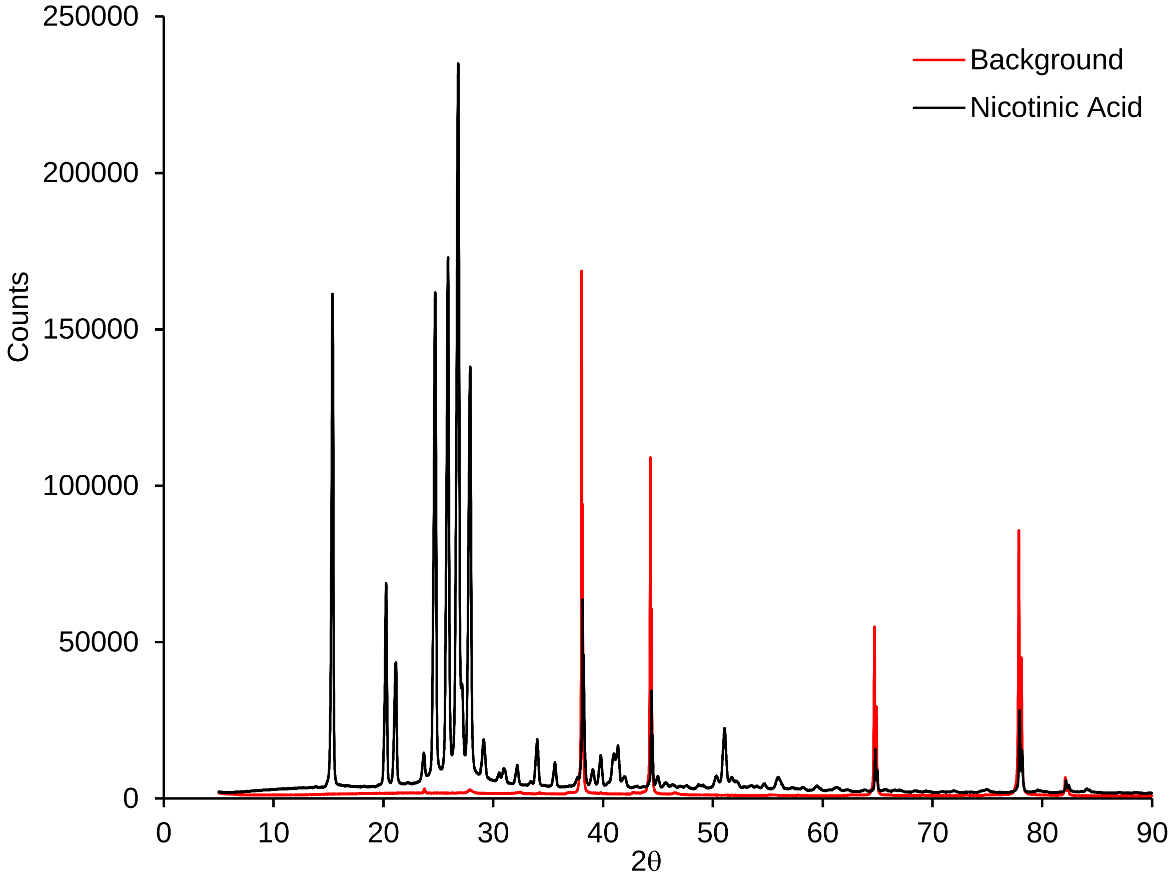
Background
Nicotinic Acid
Counts
250000
200000
150000
100000
50000
0
0
10
20
30
40
50
60
70
80
90
2θ

Solid-state ^{13}C NMR spectrum of glycine 1

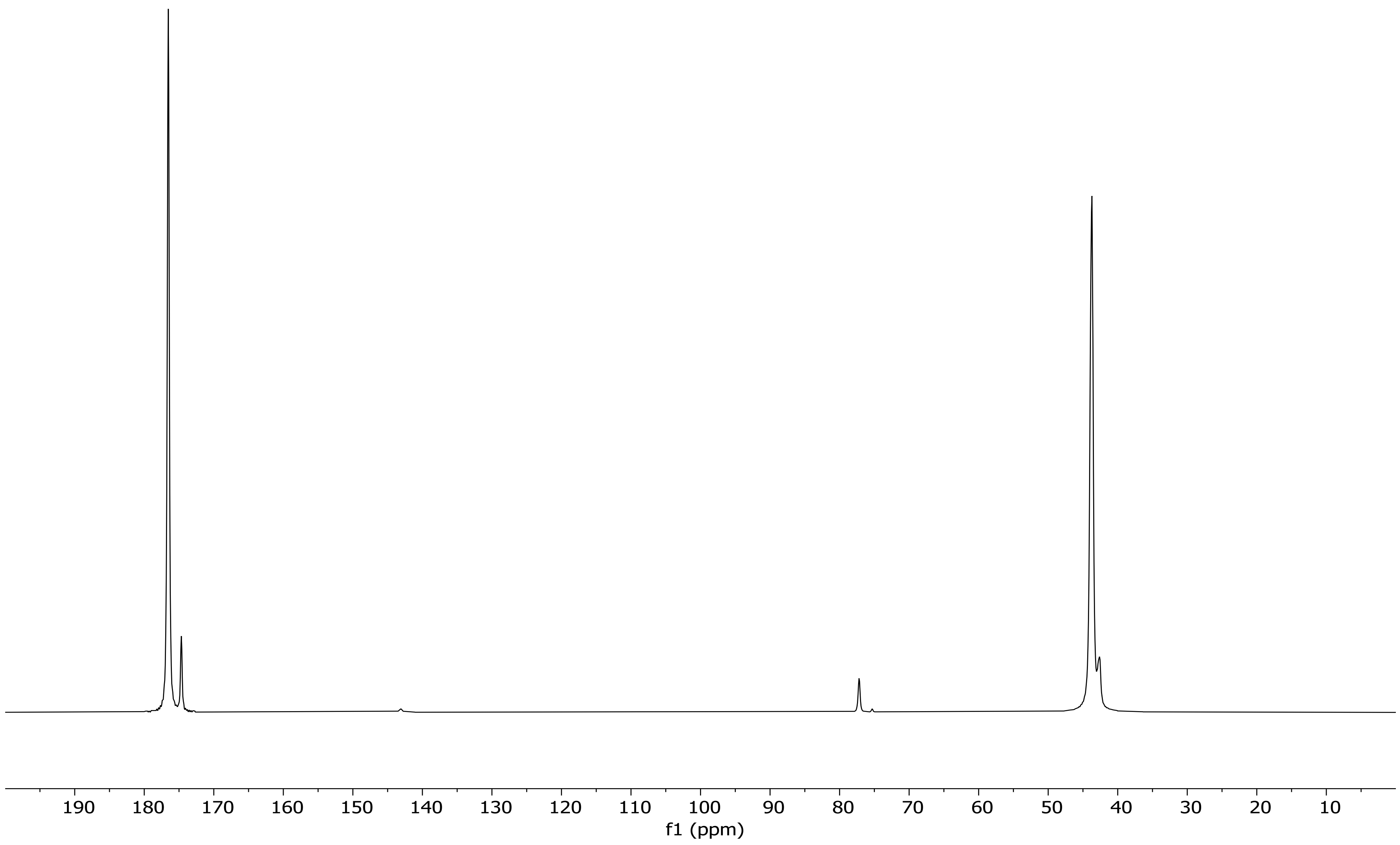

Solid-state ^{13}C NMR spectrum of β-alanine 2

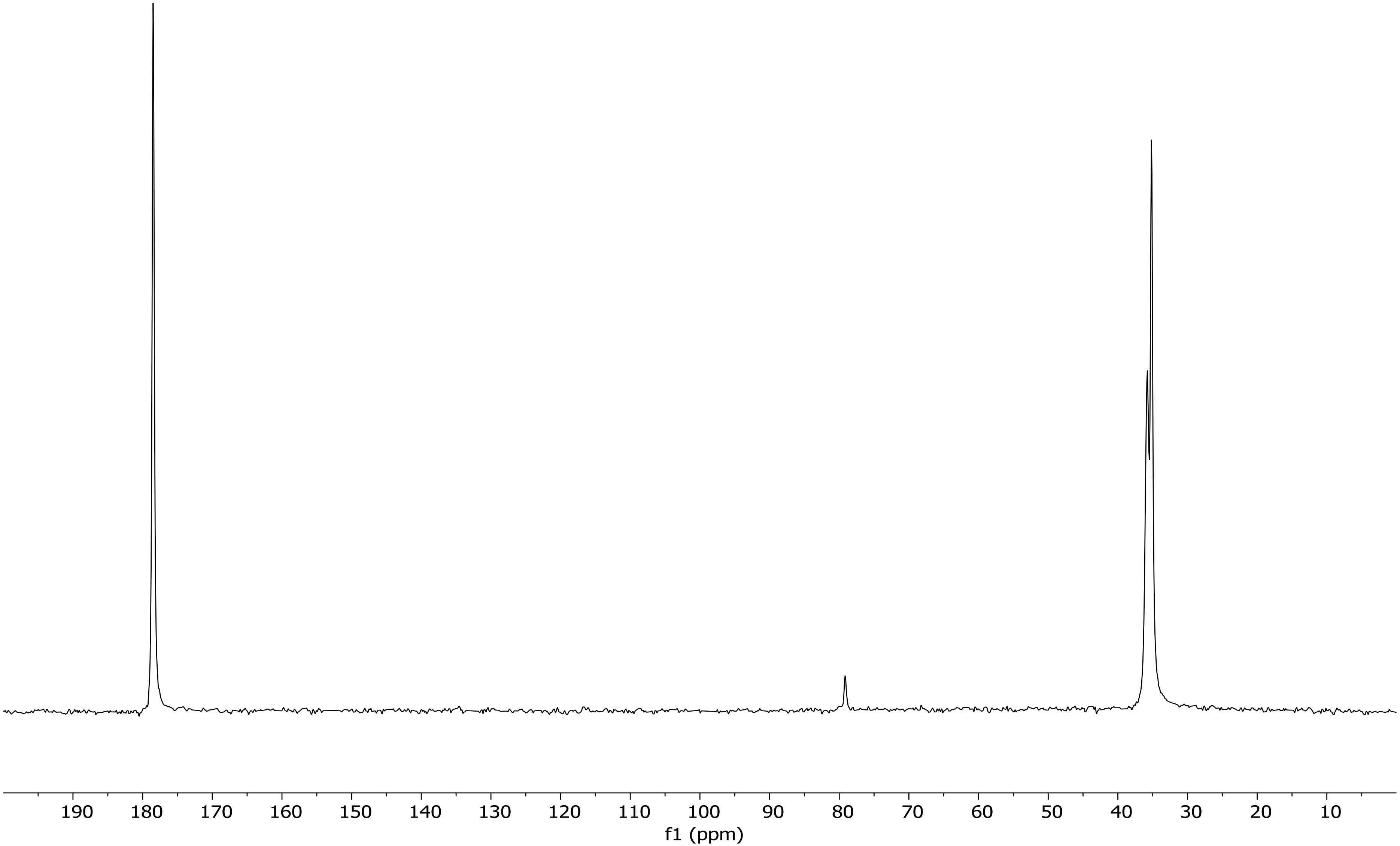

Solid-state ^{13}C NMR spectrum of urea 3

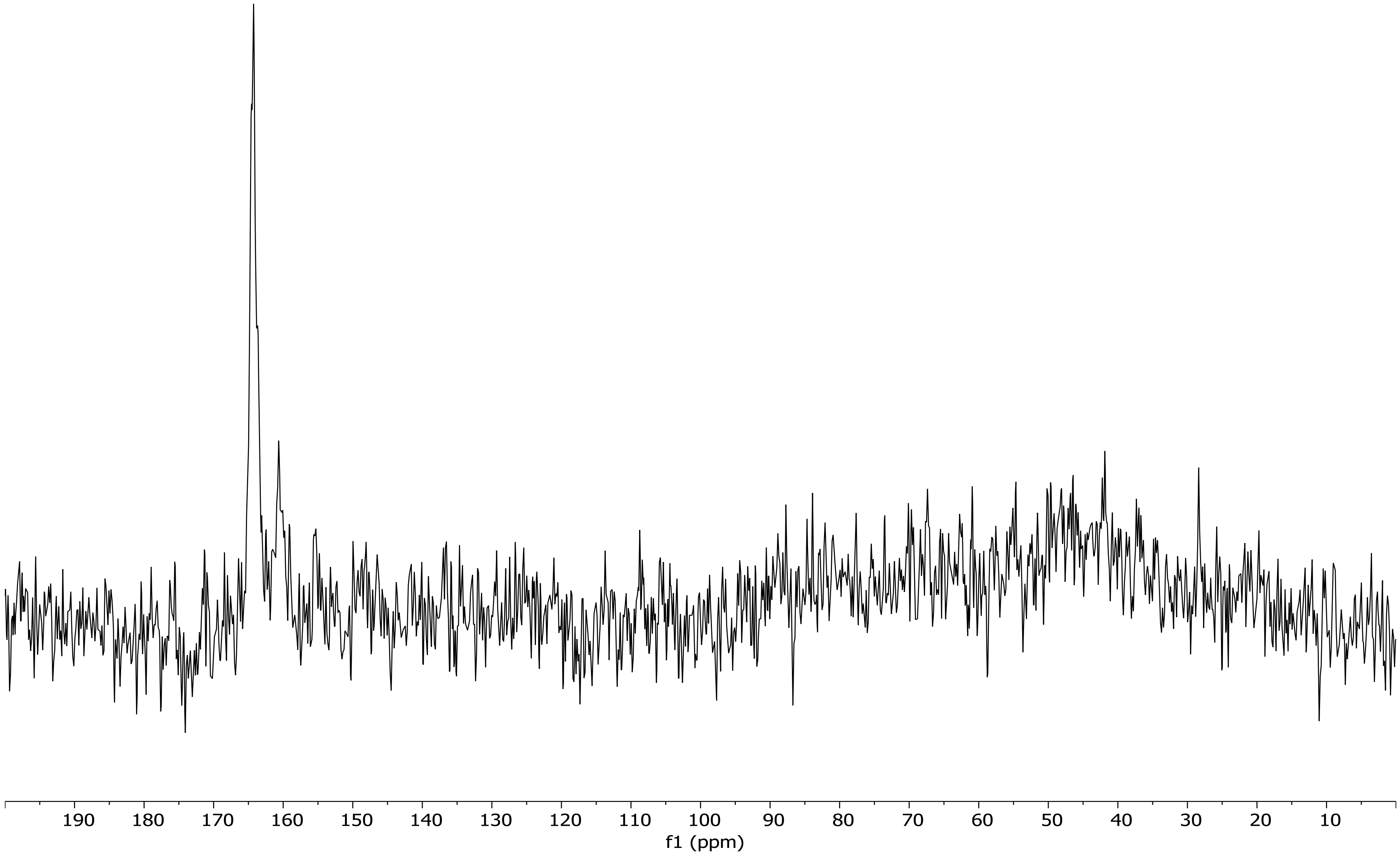

Solid-state ^{13}C NMR spectrum of melamine 4

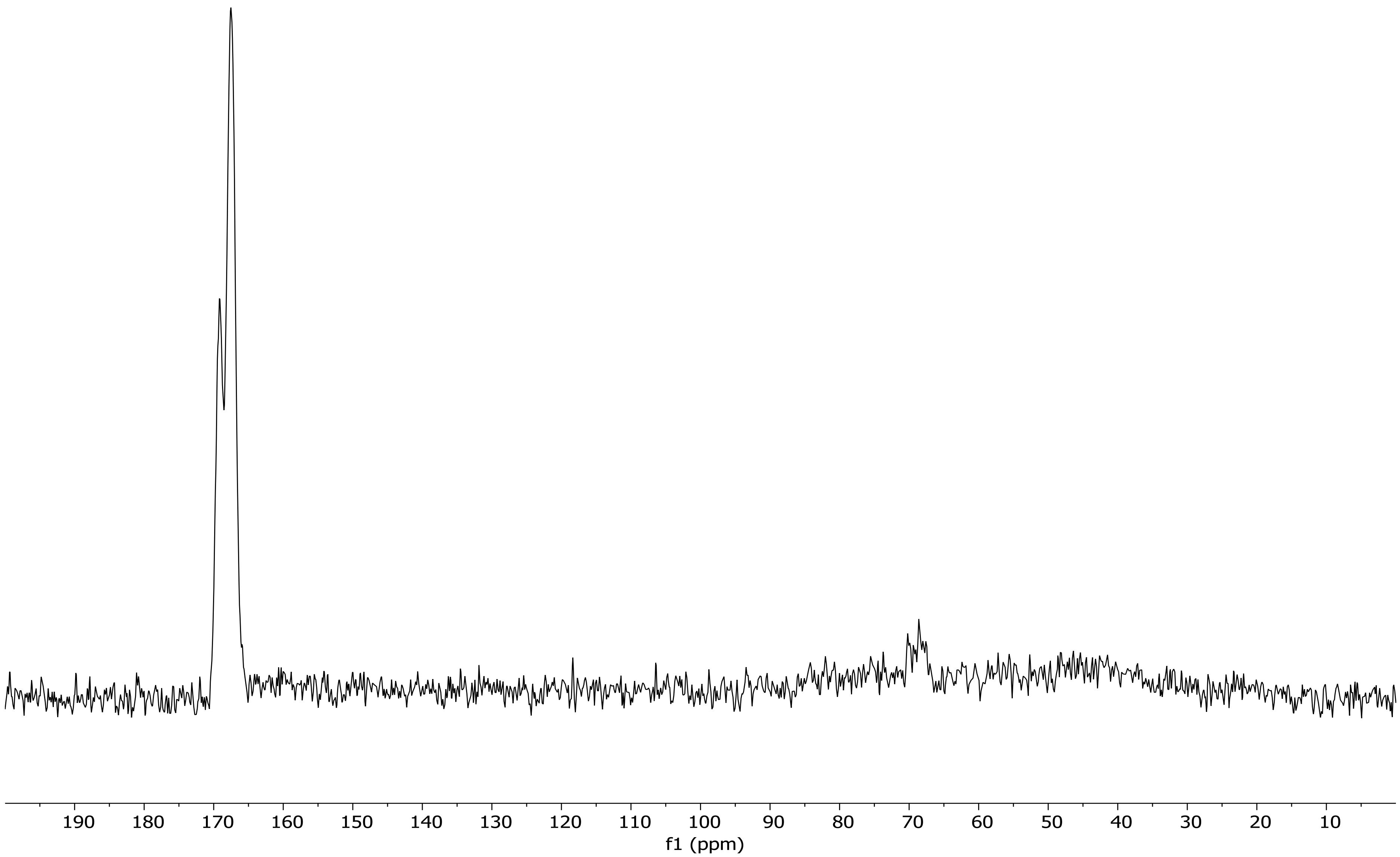

Solid-state ^{13}C NMR spectrum of nicotinamide 5

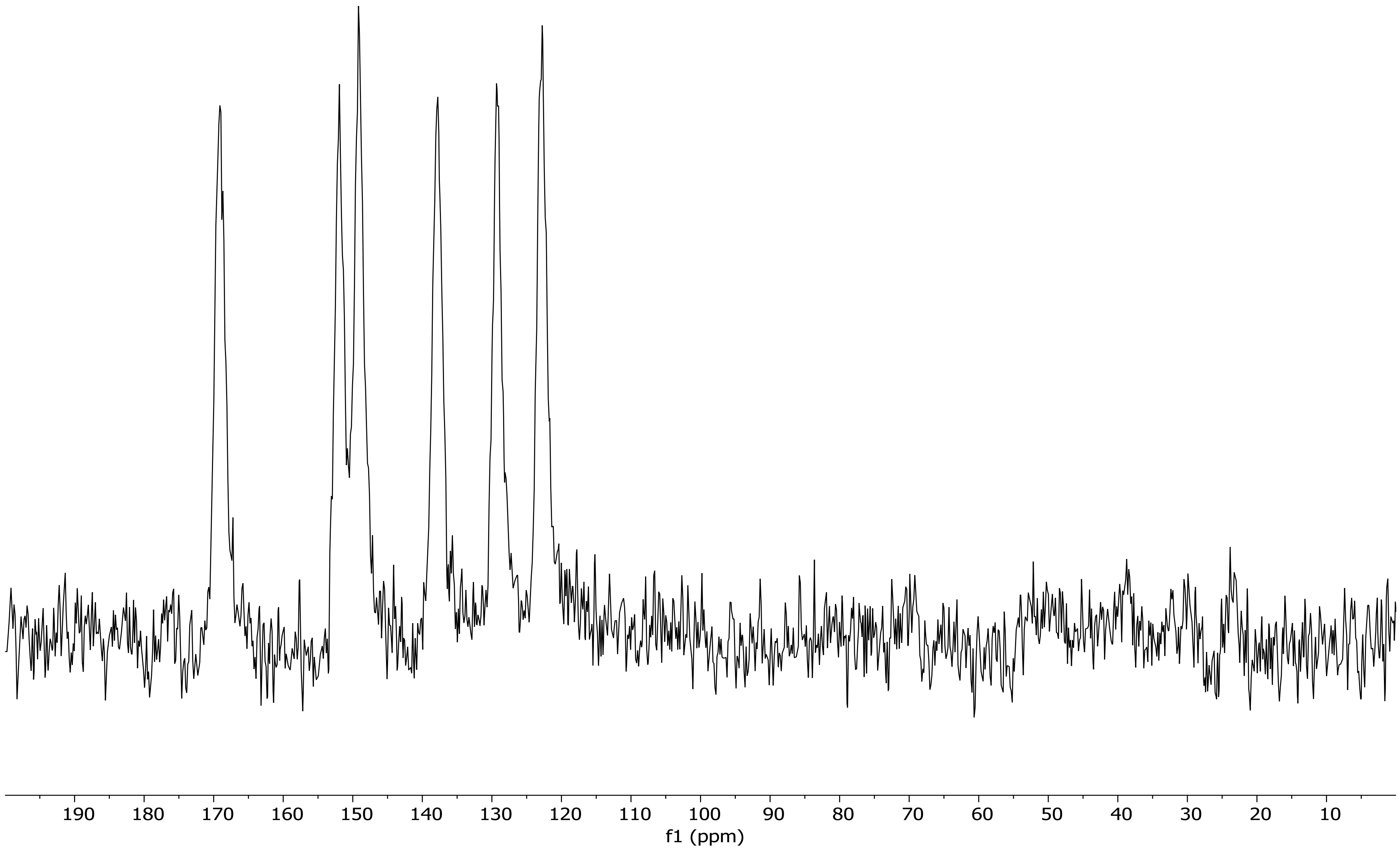

Porosimetry data for S300

Nitrogen Isotherm

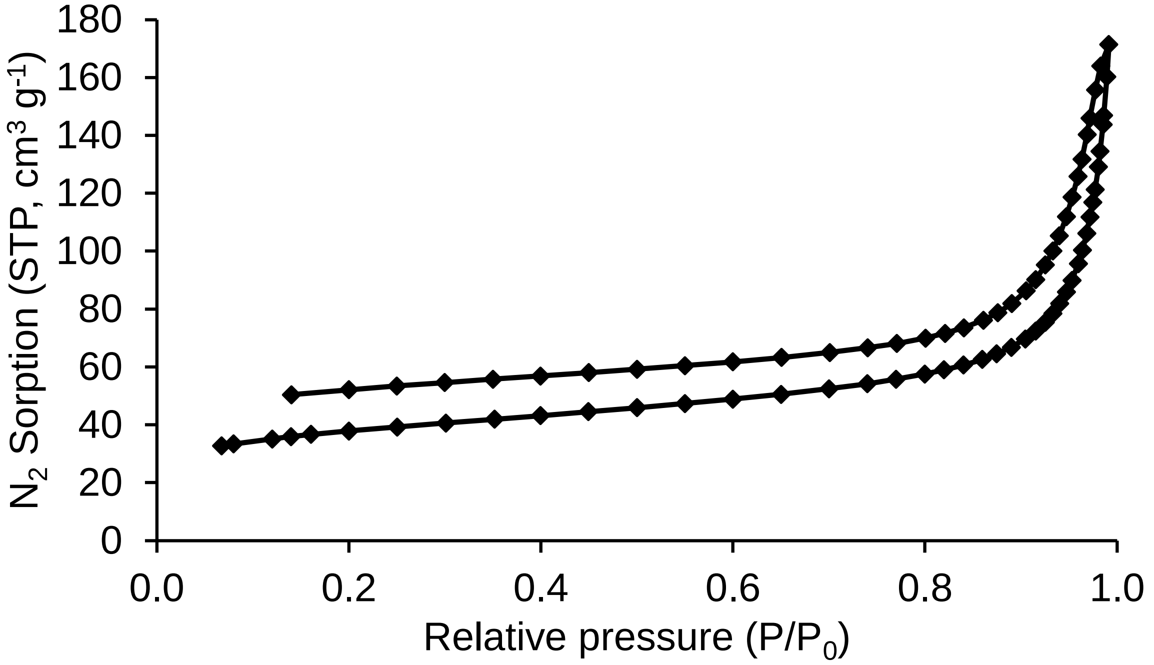

BJH Pore Size Distribution

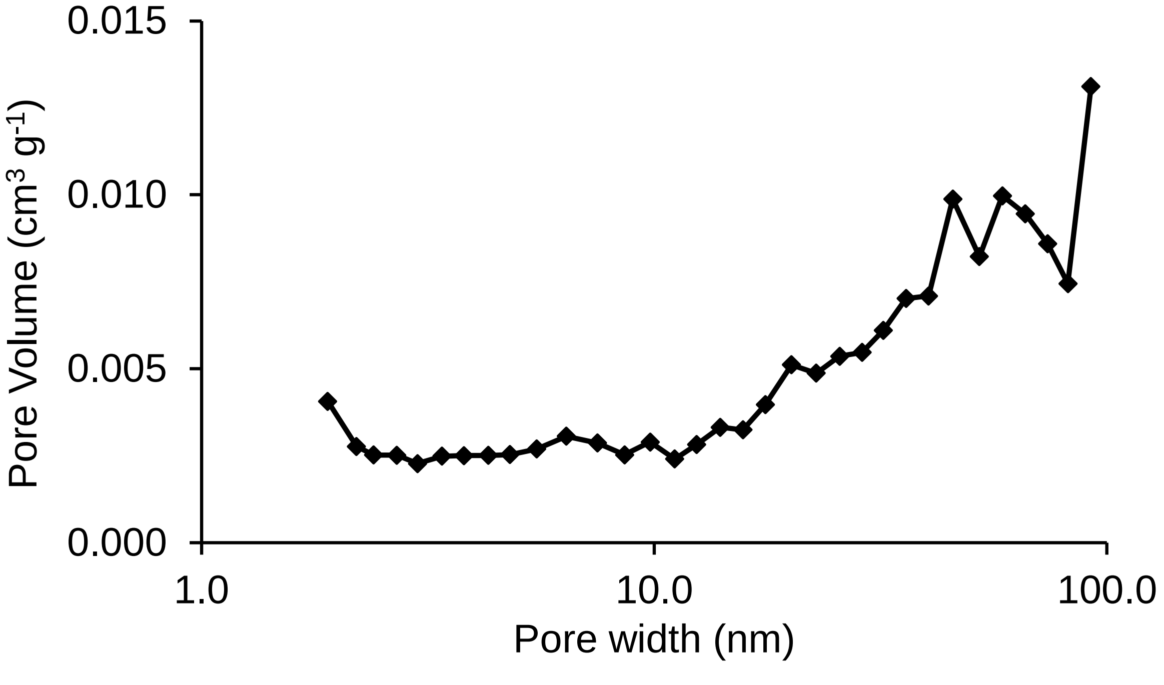

Porosimetry data for $SN_{Gly}300_{Th}$

Nitrogen Isotherm

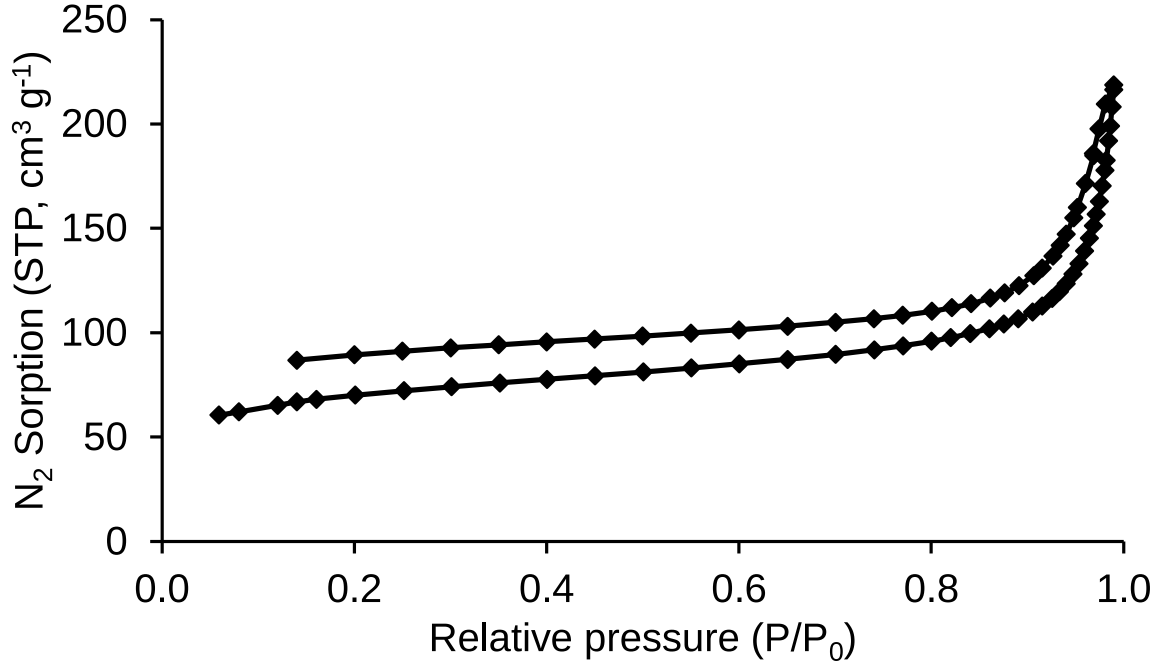

BJH Pore Size Distribution

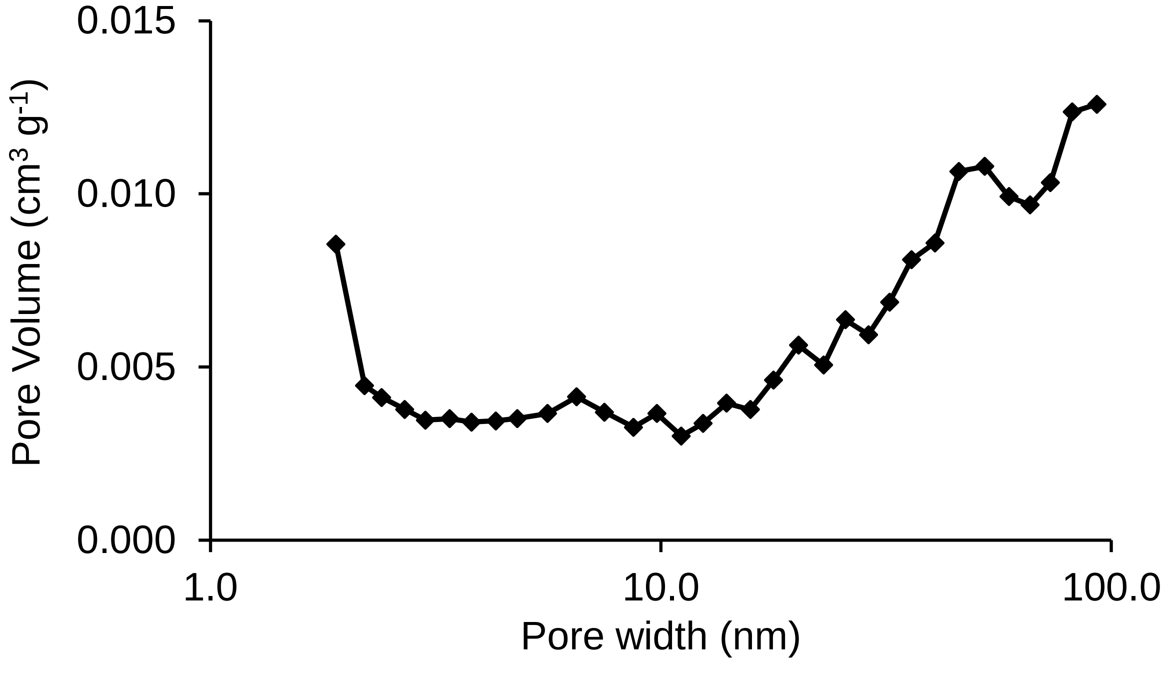

Porosimetry data for $SN_{Bal}300_{Th}$

Nitrogen Isotherm

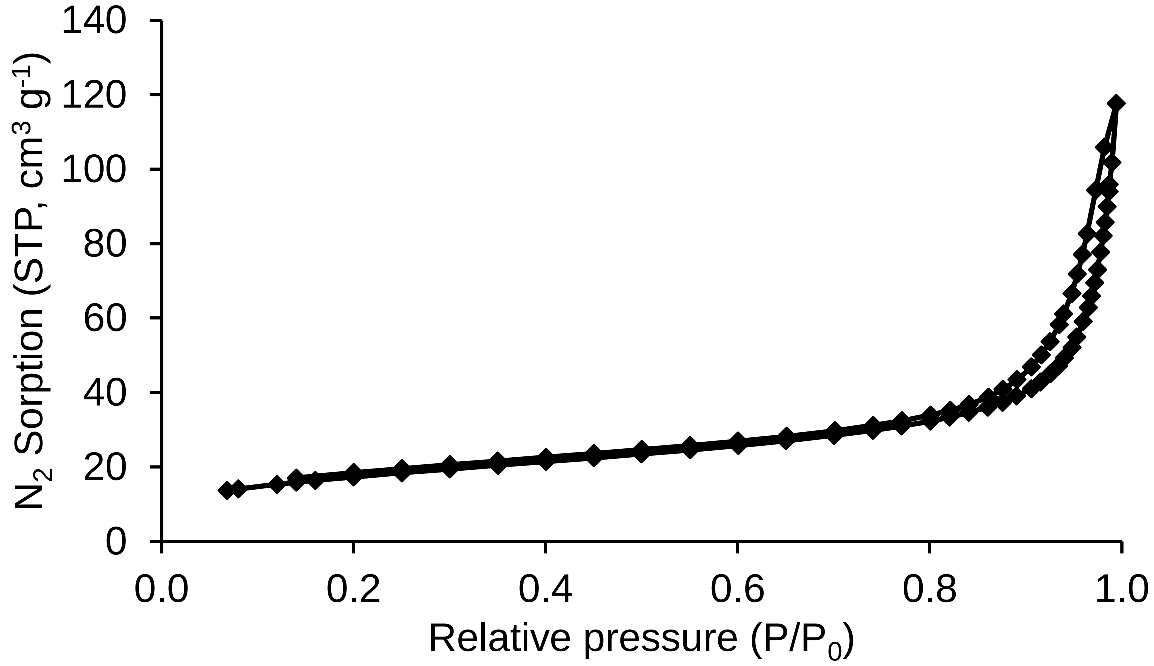

BJH Pore Size Distribution

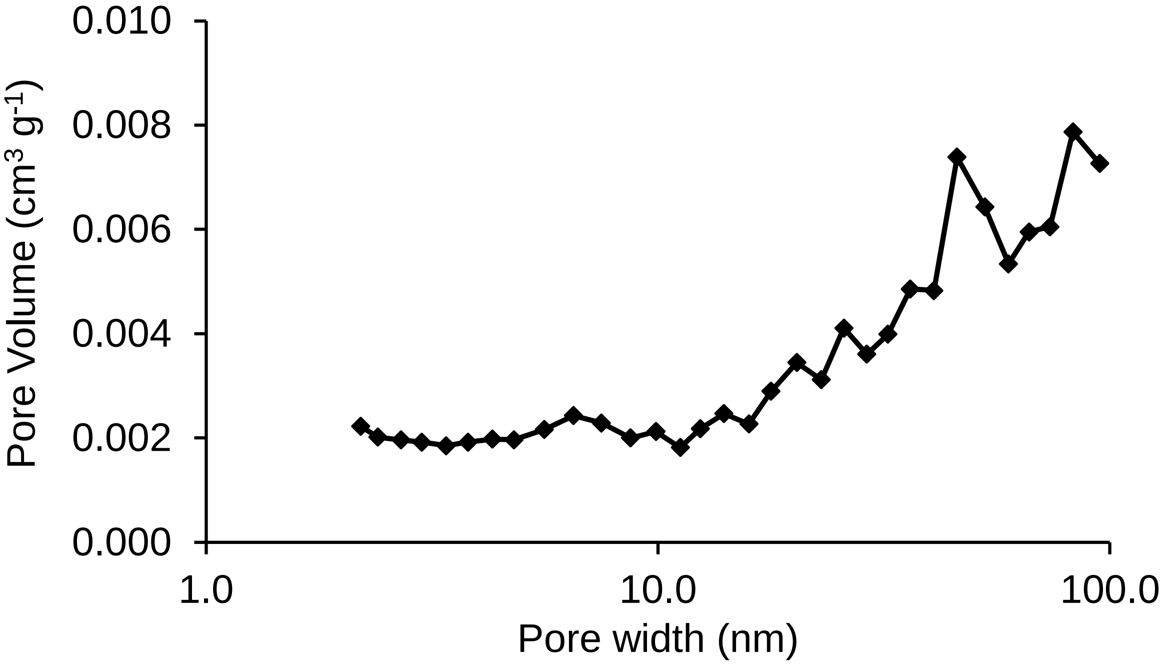

Porosimetry data for $SN_{Ure}300_{Th}$

Nitrogen Isotherm

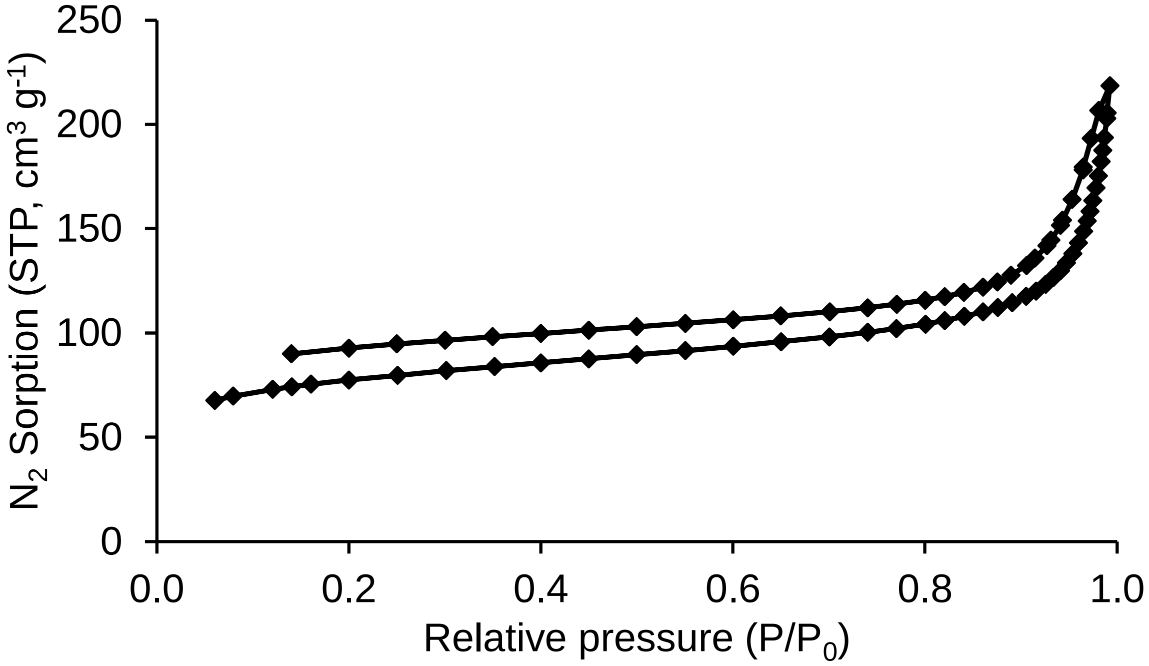

BJH Pore Size Distribution

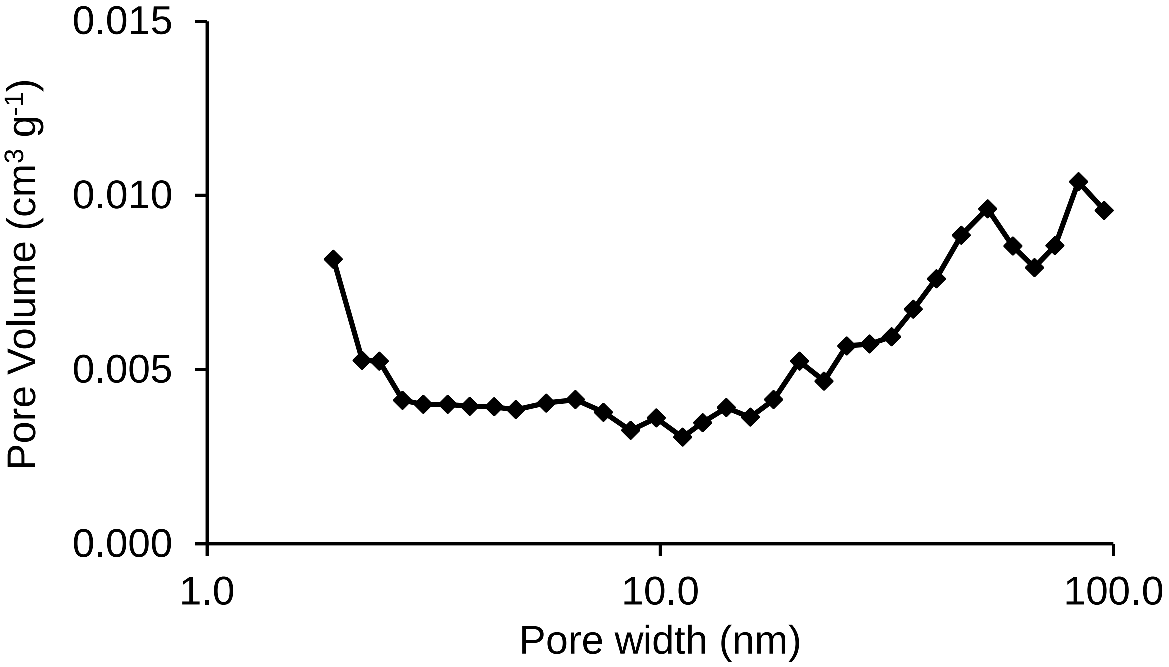

Porosimetry data for $SN_{Mel}300_{Th}$

Nitrogen Isotherm

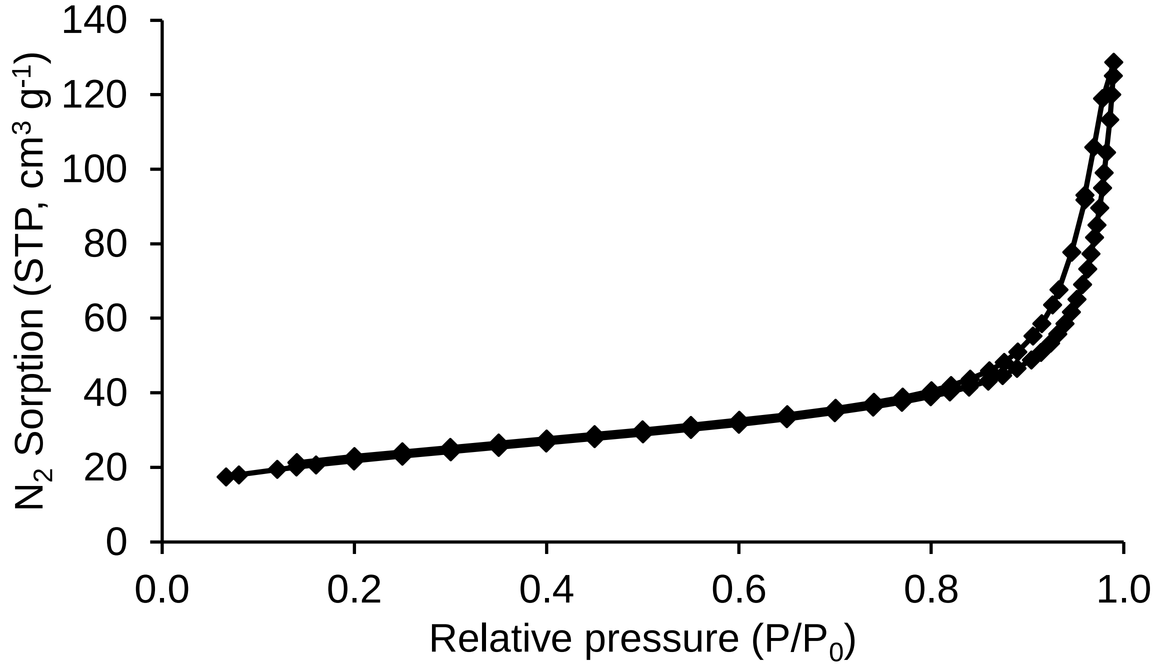

BJH Pore Size Distribution

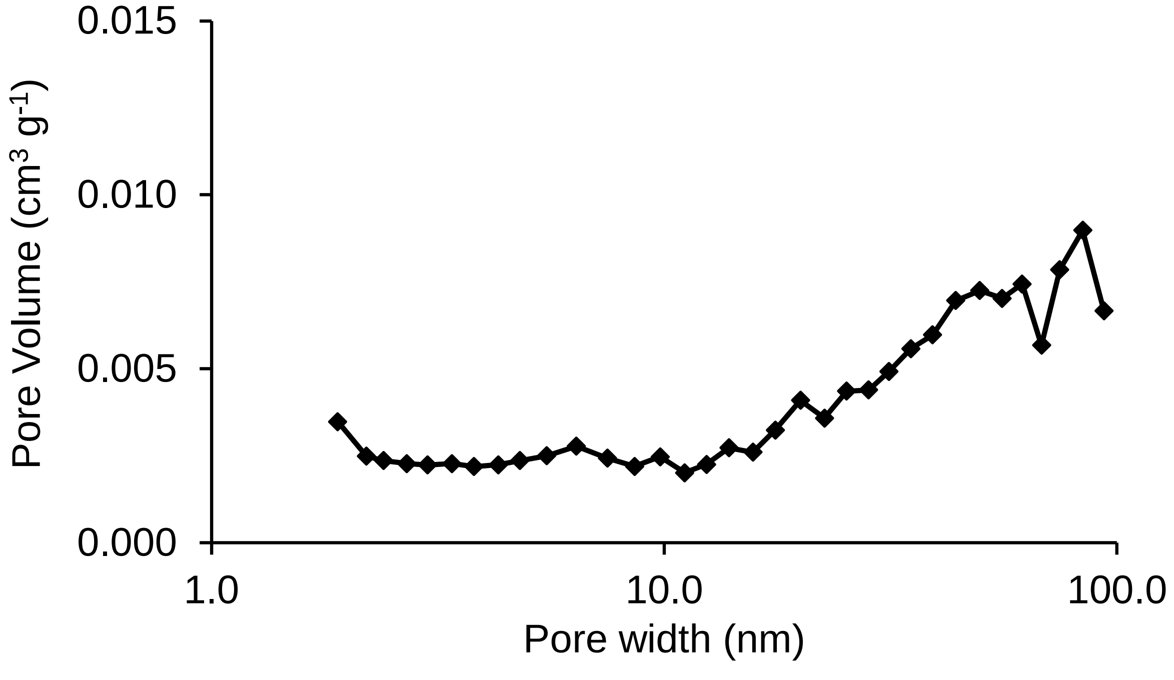

Porosimetry data for $SN_{Nic}300_{Th}$

Nitrogen Isotherm

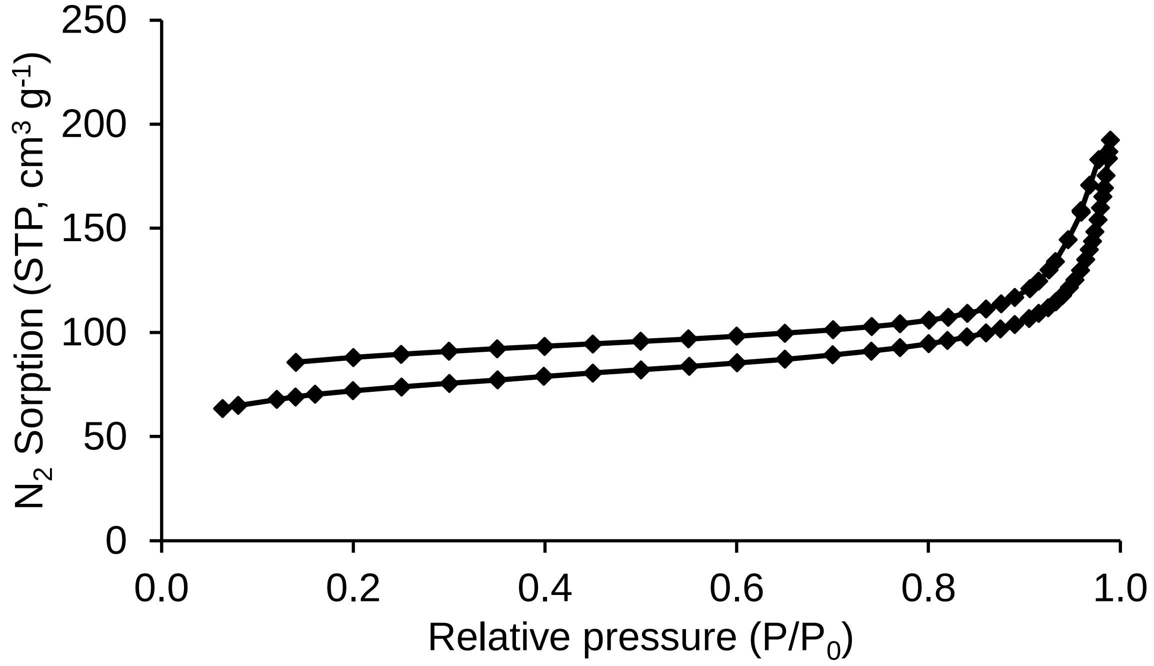

BJH Pore Size Distribution

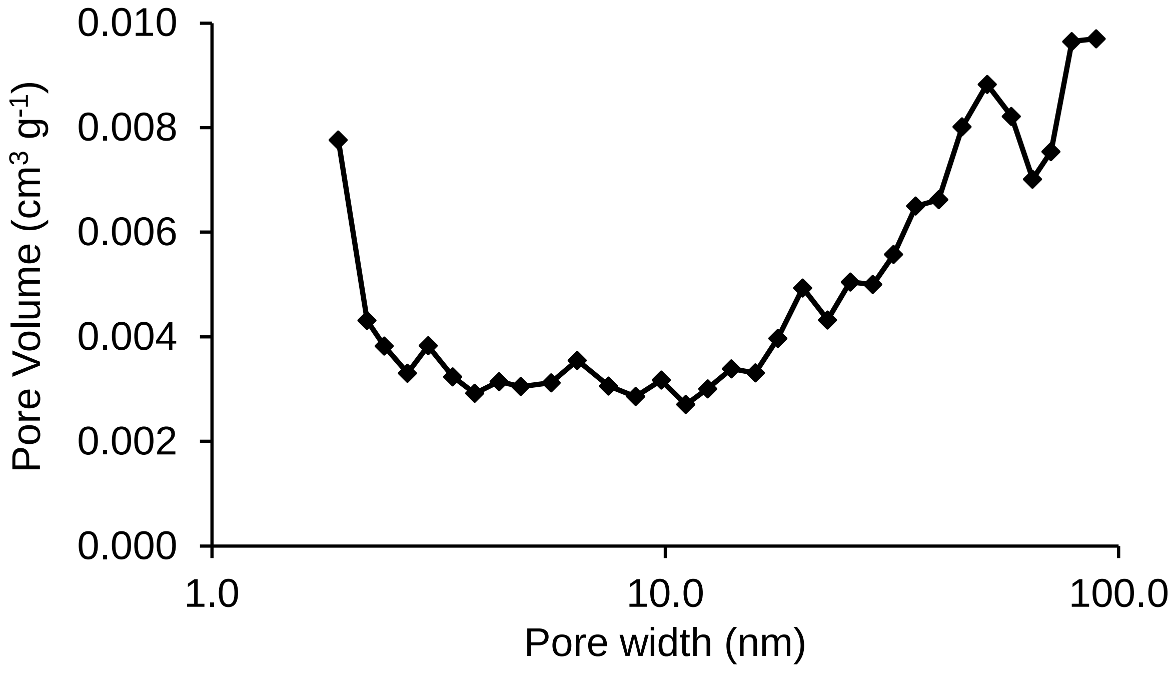

Porosimetry data for $SN_{Gly}300_{Mo}$

Nitrogen Isotherm

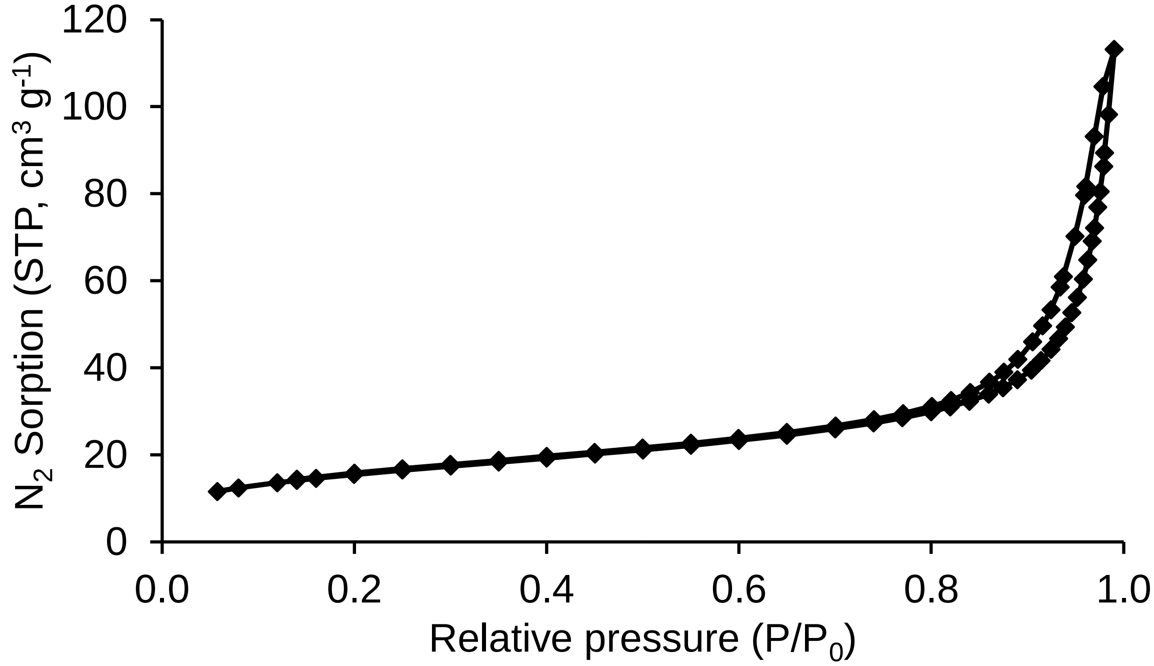

BJH Pore Size Distribution

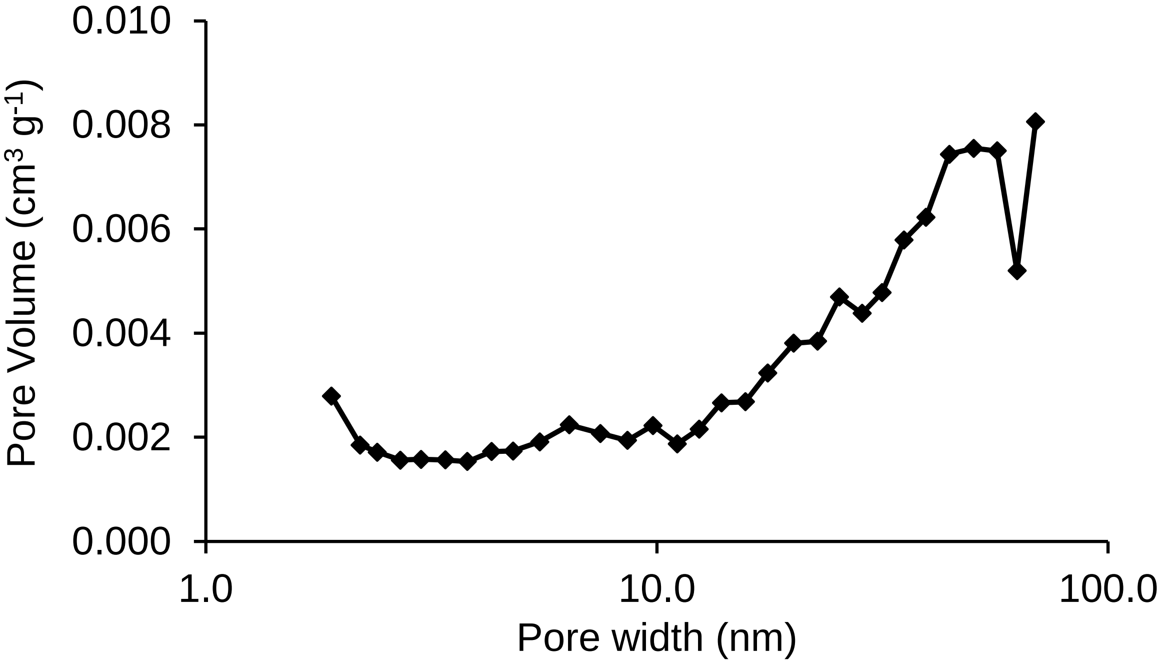

Porosimetry data for $SN_{Bal}300_{Mo}$

Nitrogen Isotherm

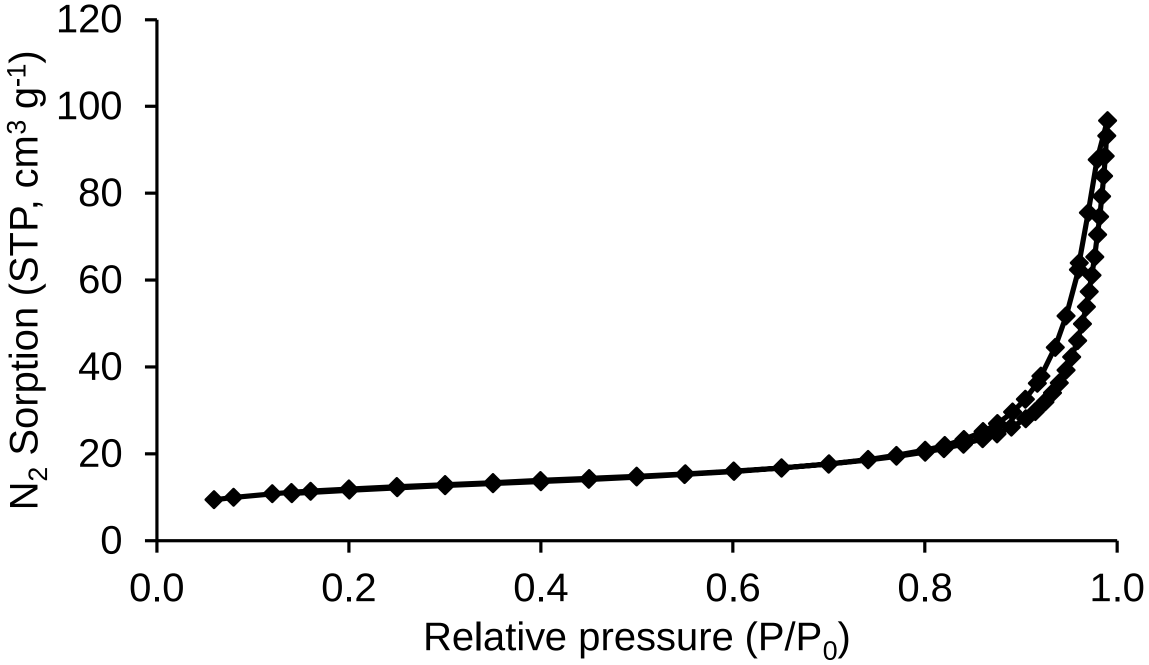

BJH Pore Size Distribution

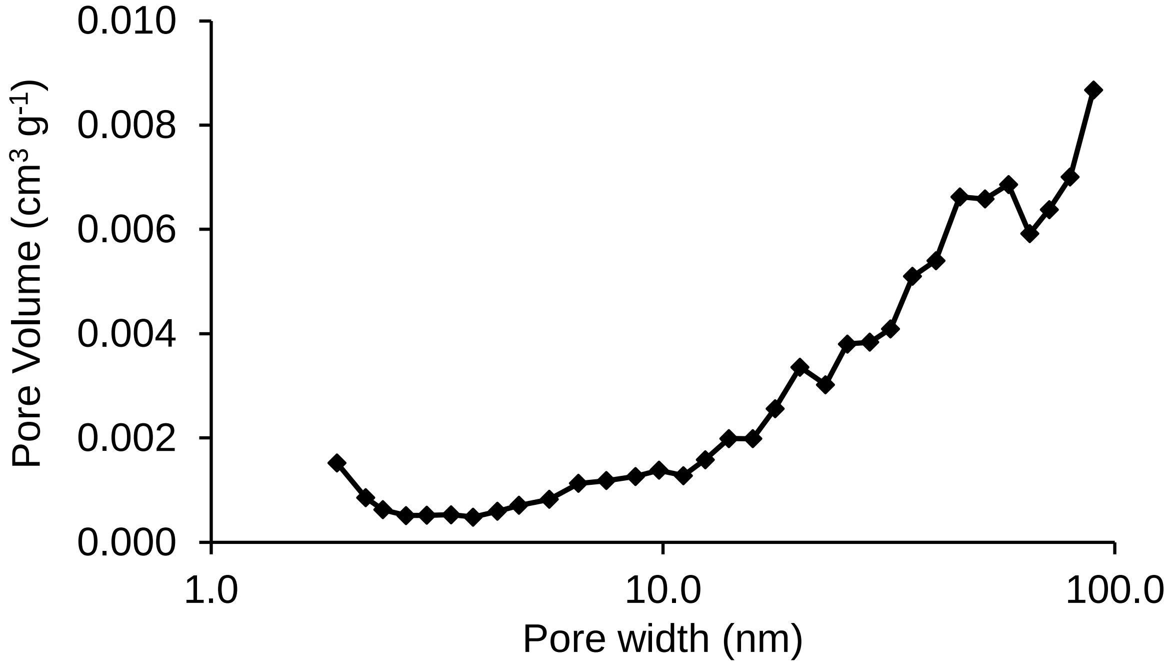

Porosimetry data for $SN_{Ure}300_{Mo}$

Nitrogen Isotherm

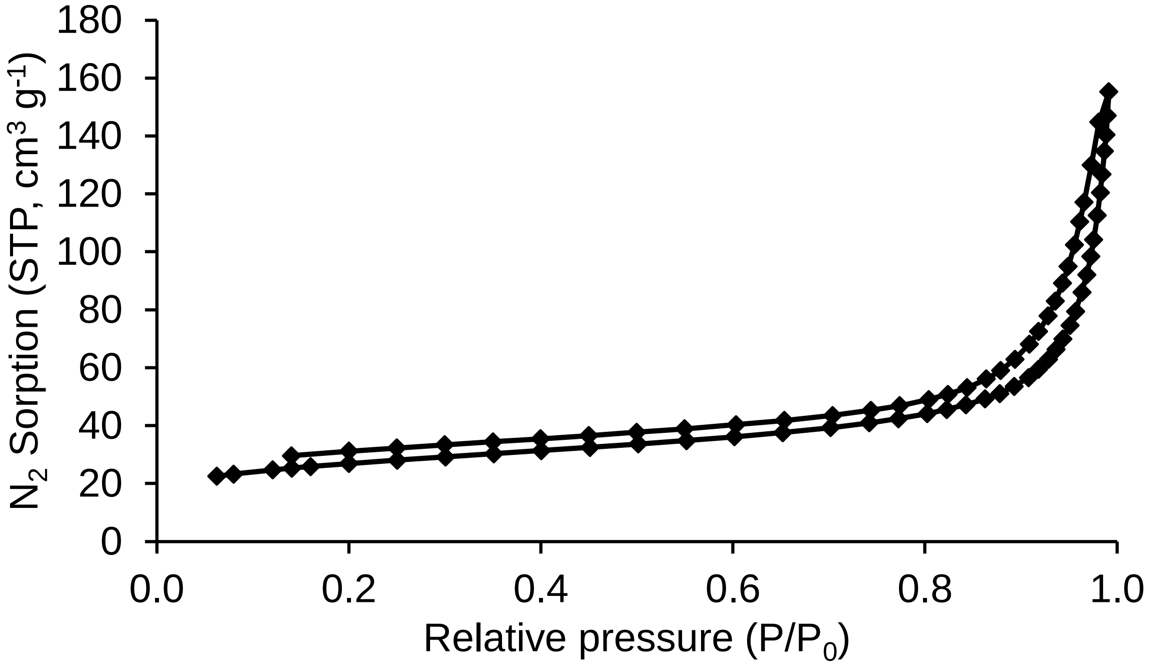

BJH Pore Size Distribution

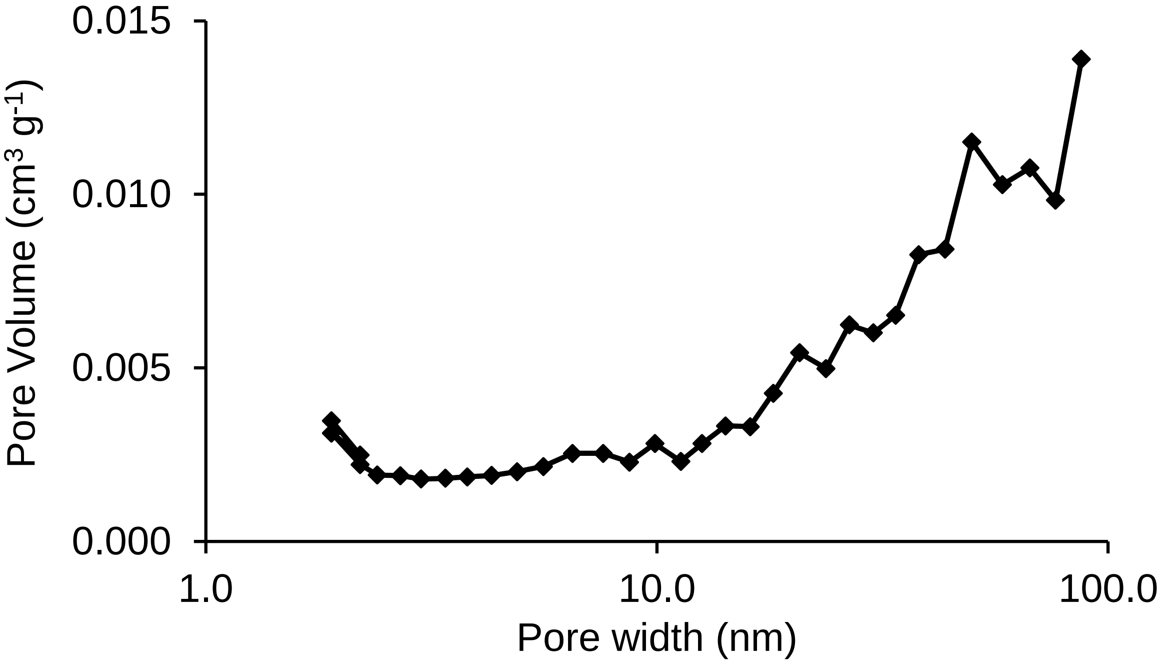

Porosimetry data for $SN_{Mel}300_{Mo}$

Nitrogen Isotherm

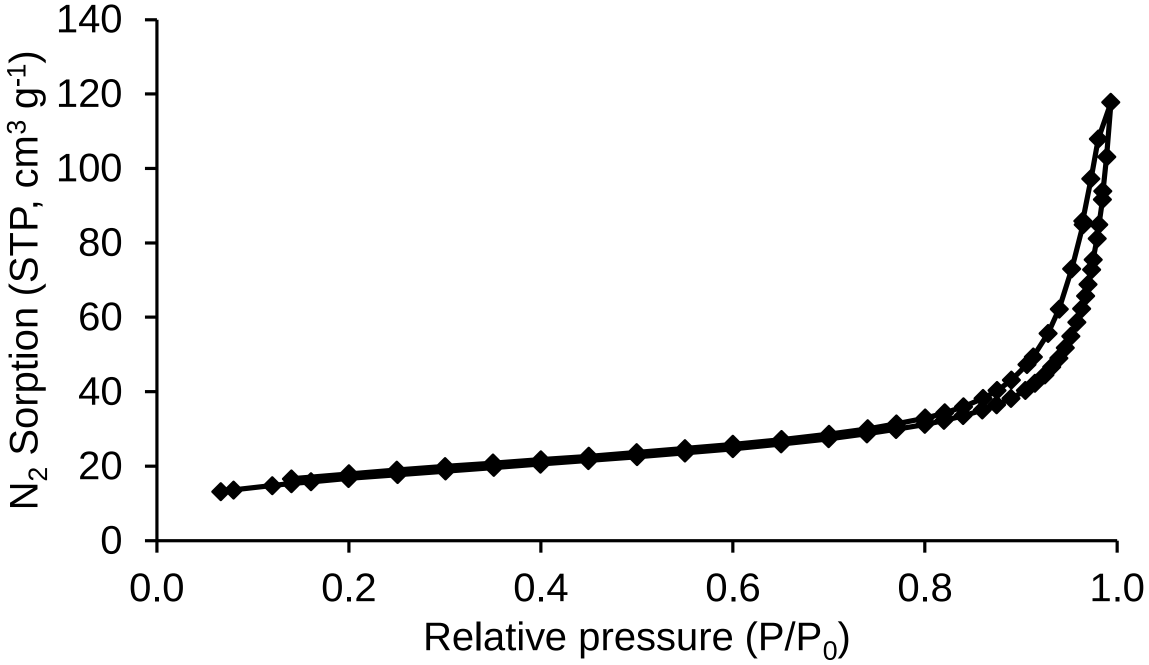

BJH Pore Size Distribution

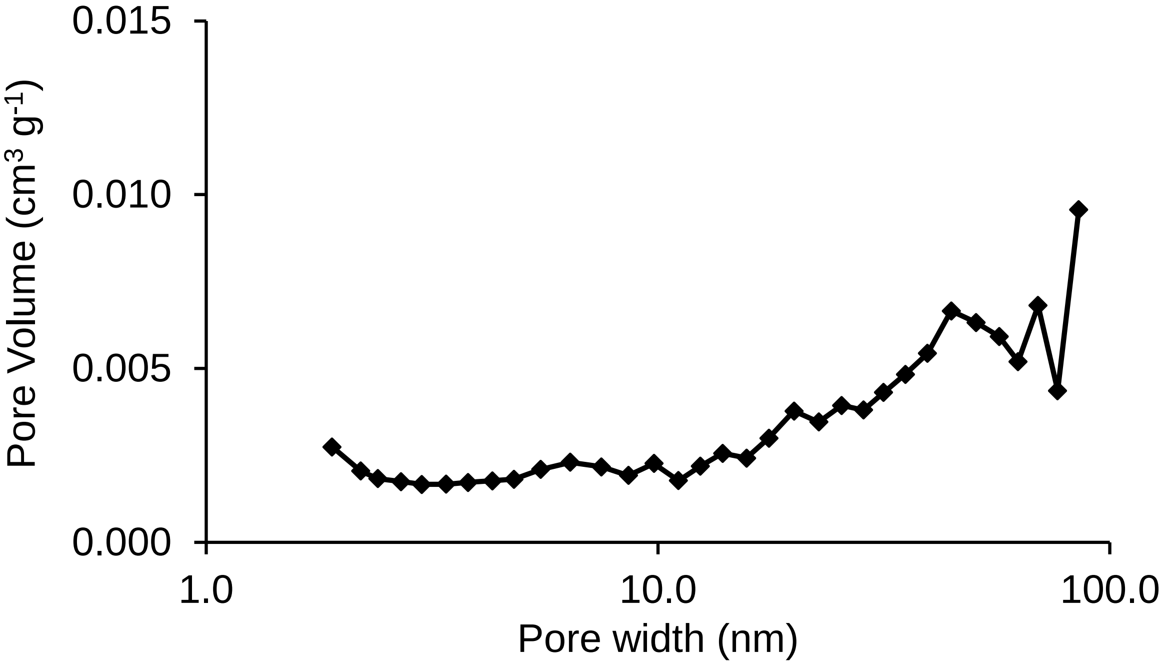

Porosimetry data for $SN_{Nic}300_{Mo}$

Nitrogen Isotherm

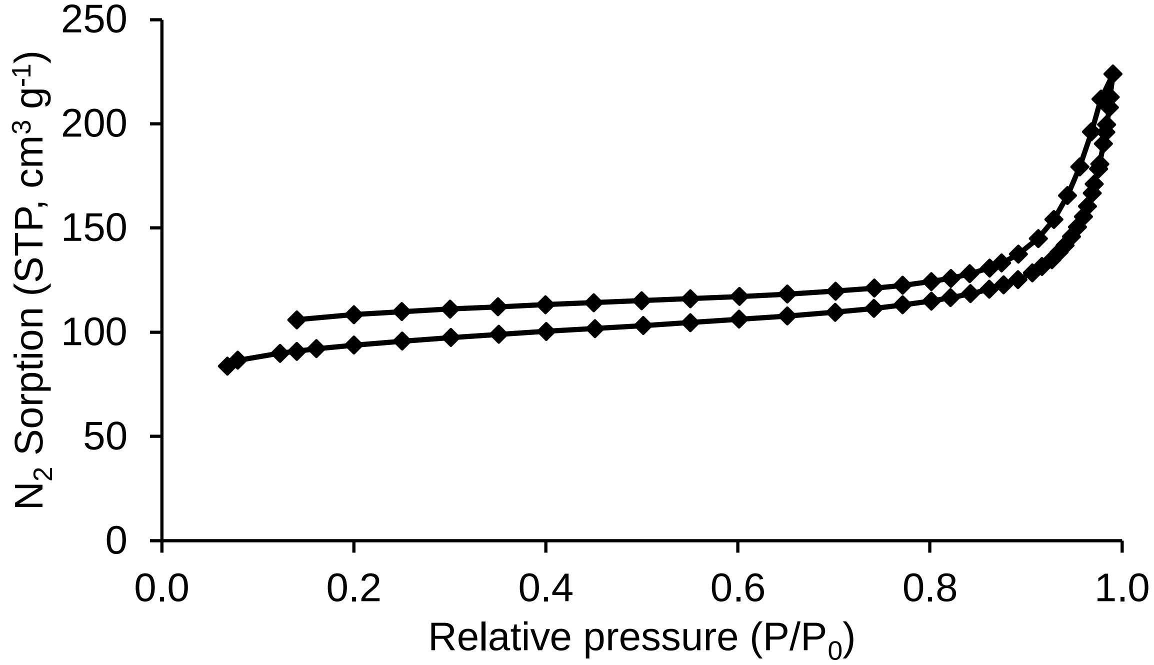

BJH Pore Size Distribution

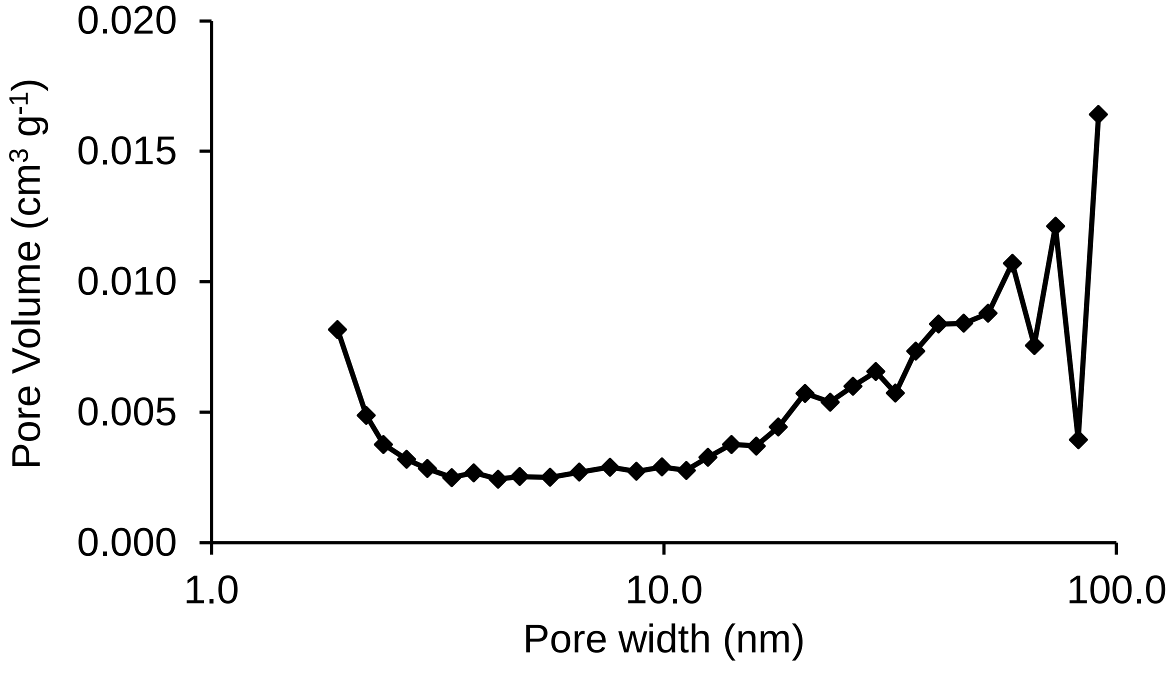

Porosimetry data for $SN_{Gly}300_{Mu}$

Nitrogen Isotherm

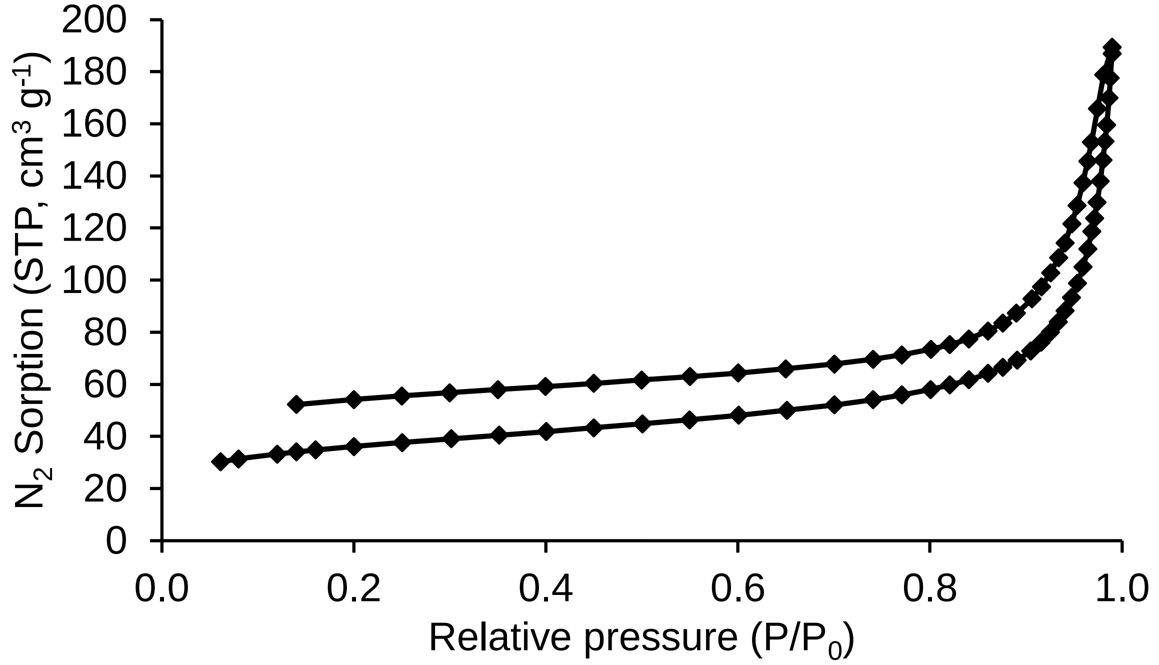

BJH Pore Size Distribution

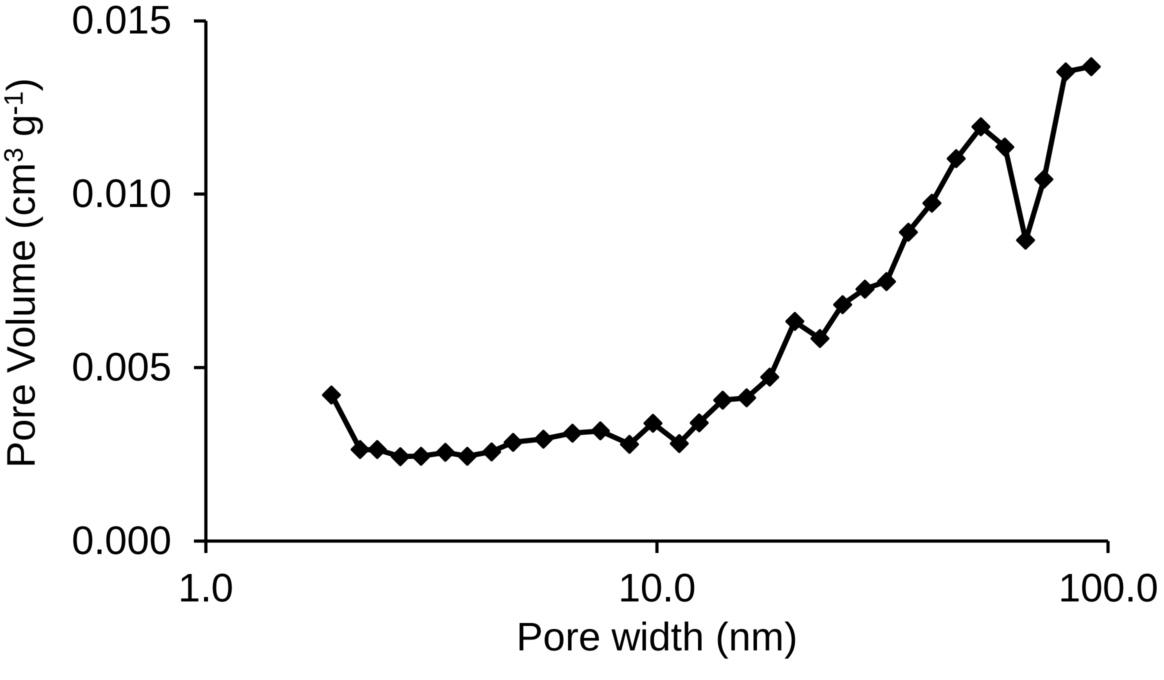

Porosimetry data for $SN_{Bal}300_{Mu}$

Nitrogen Isotherm

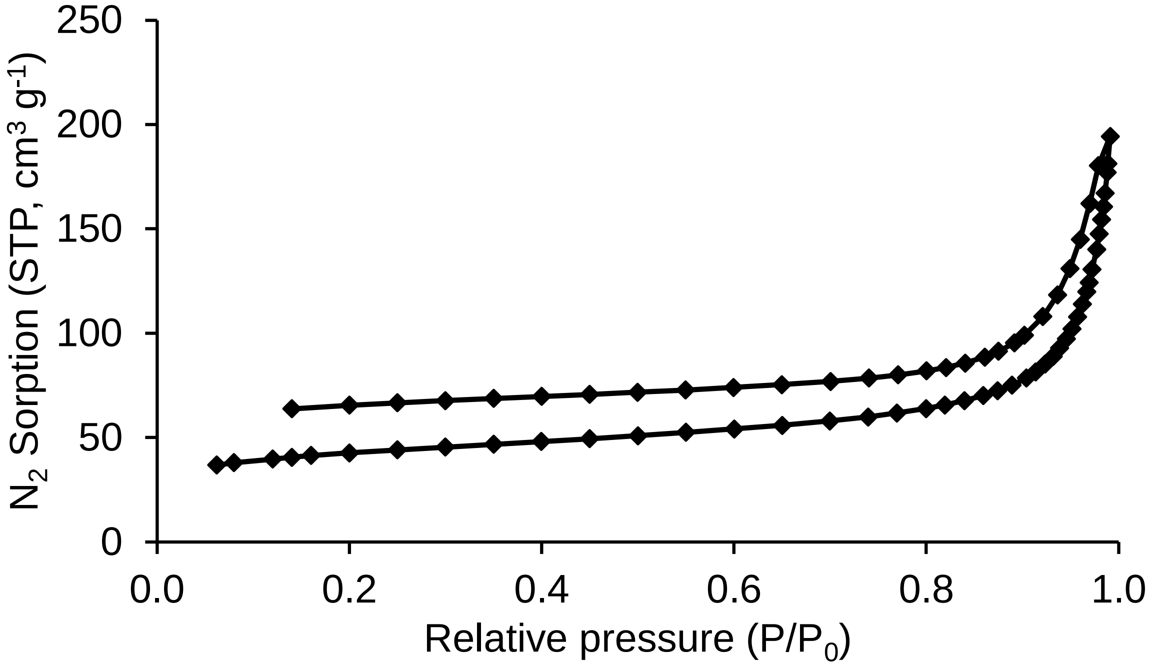

BJH Pore Size Distribution

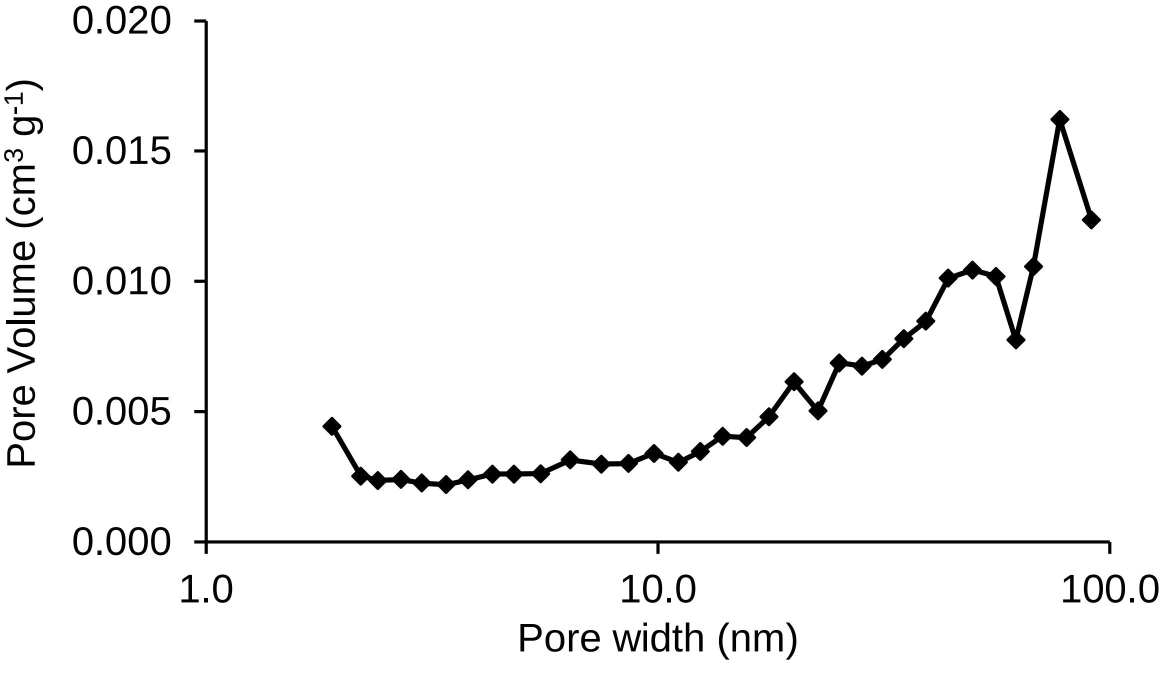

Porosimetry data for $SN_{Ure}300_{Mu}$

Nitrogen Isotherm

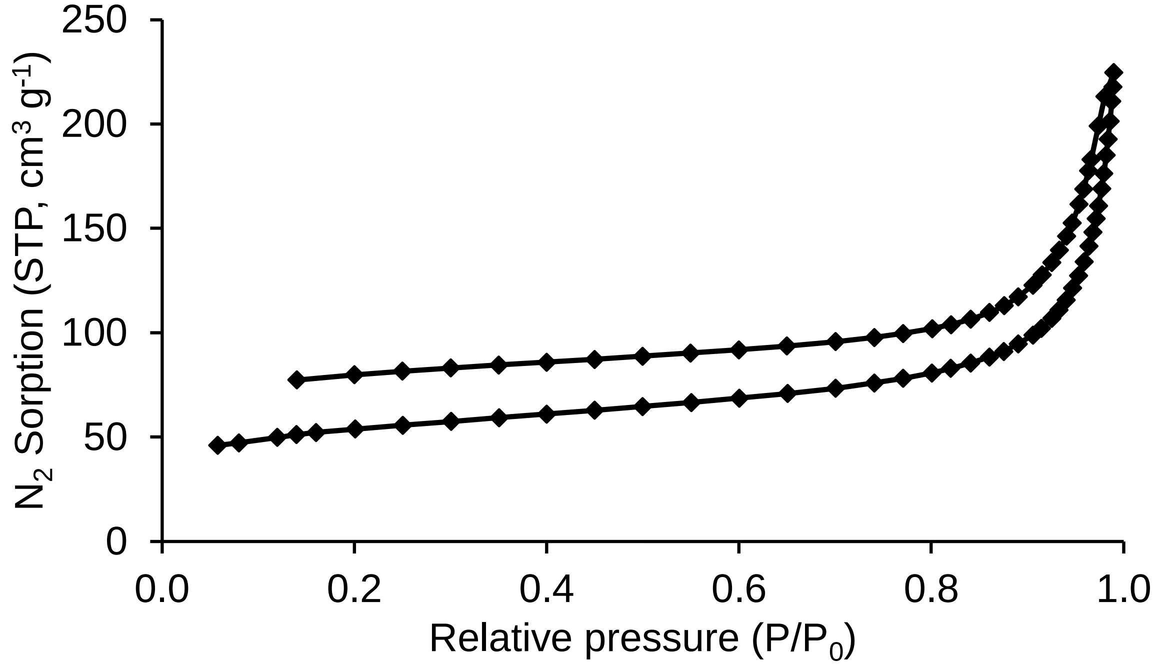

BJH Pore Size Distribution

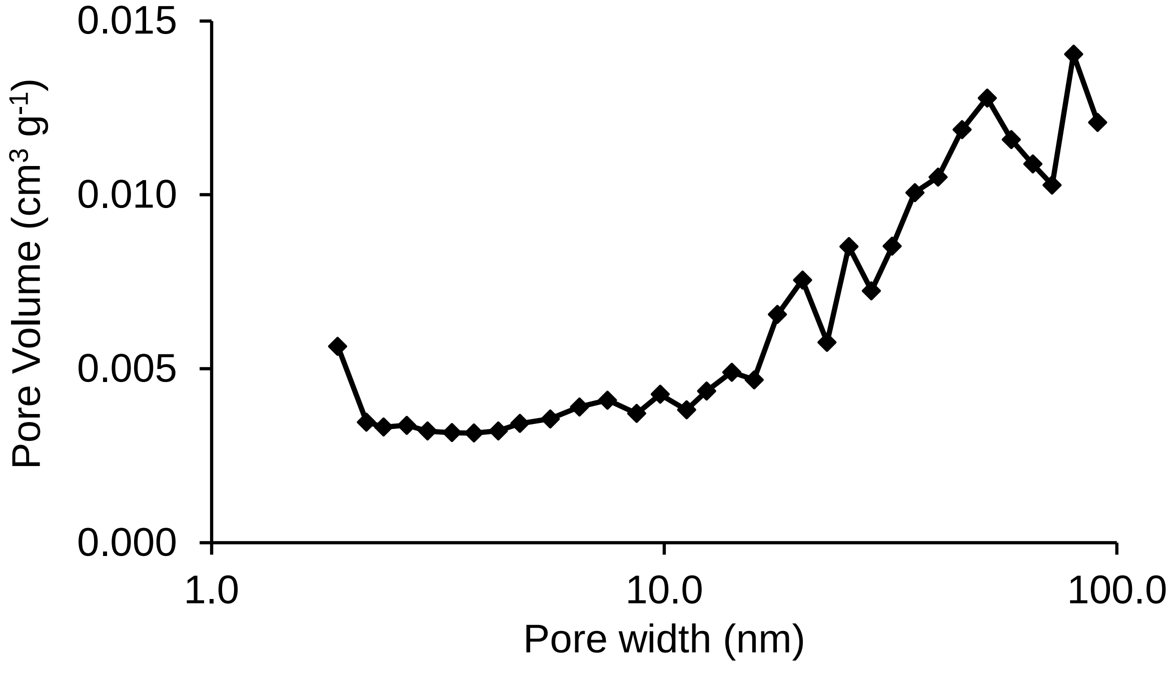

Porosimetry data for $SN_{Mel}300_{Mu}$

Nitrogen Isotherm

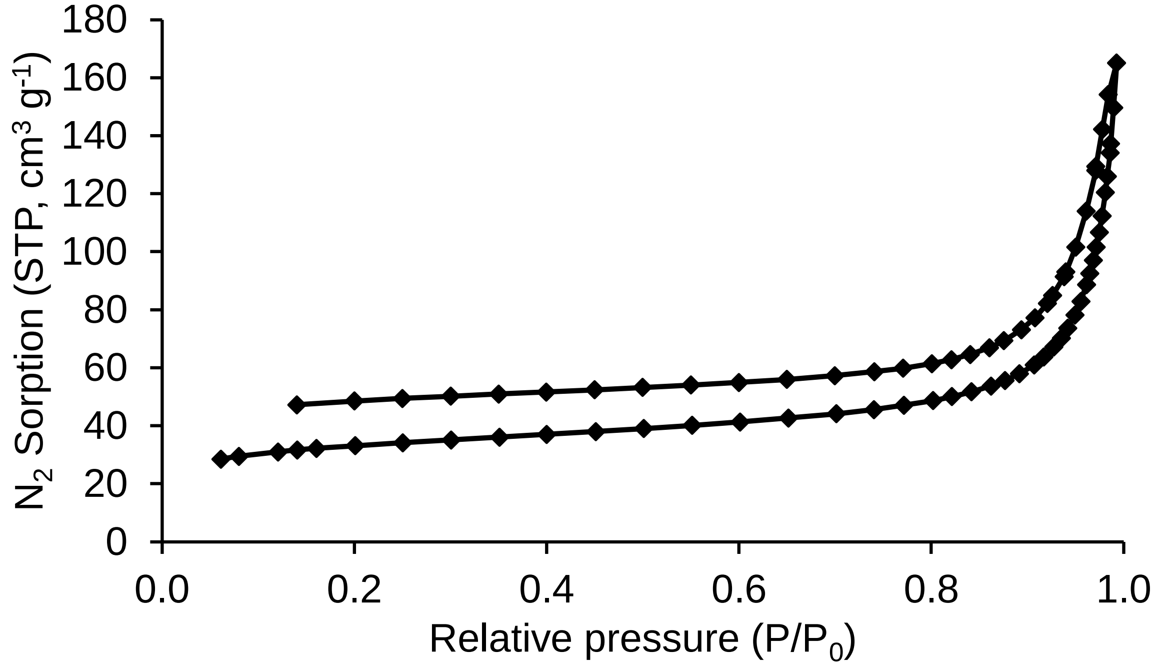

BJH Pore Size Distribution

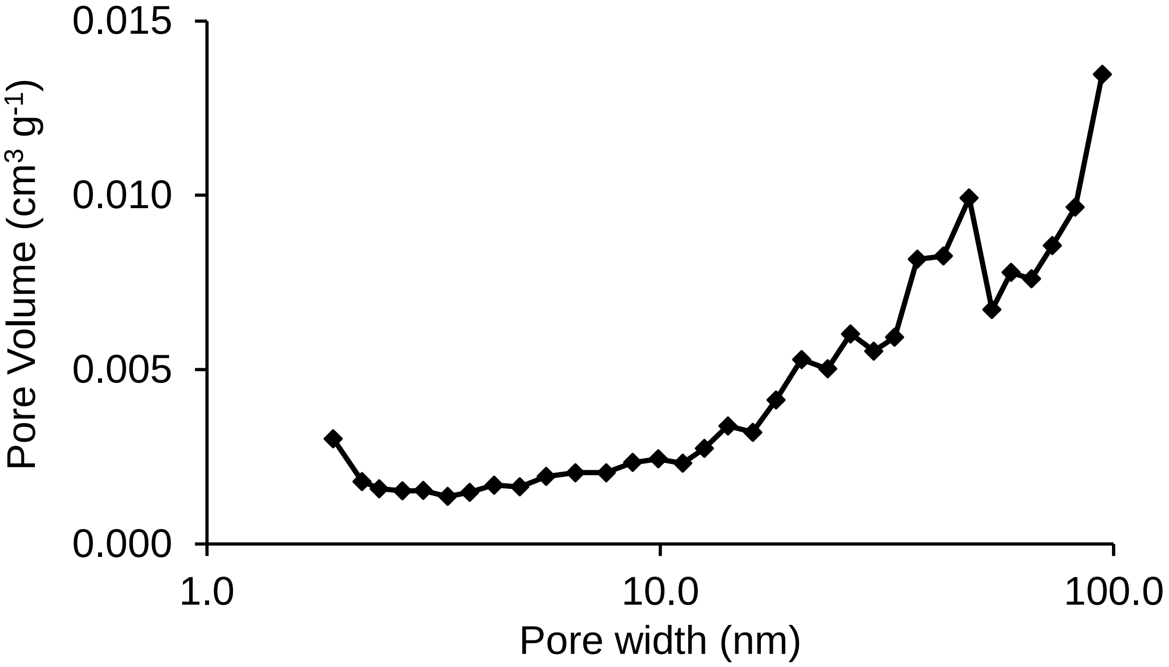

Porosimetry data for $SN_{Nic}300_{Mu}$

Nitrogen Isotherm

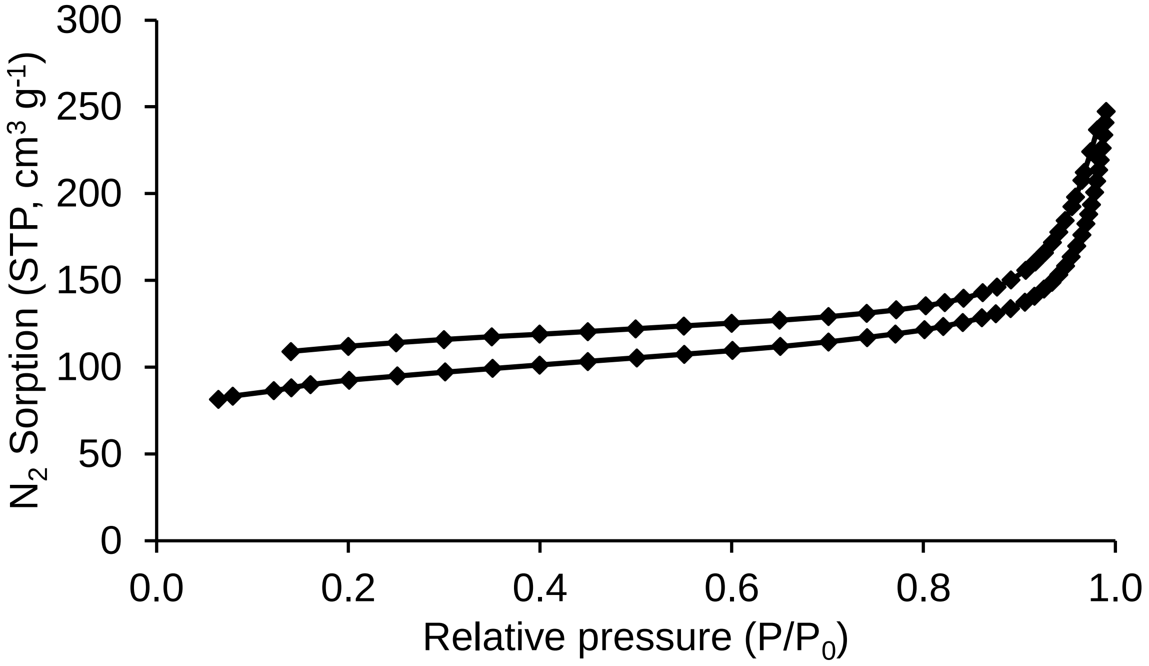

BJH Pore Size Distribution

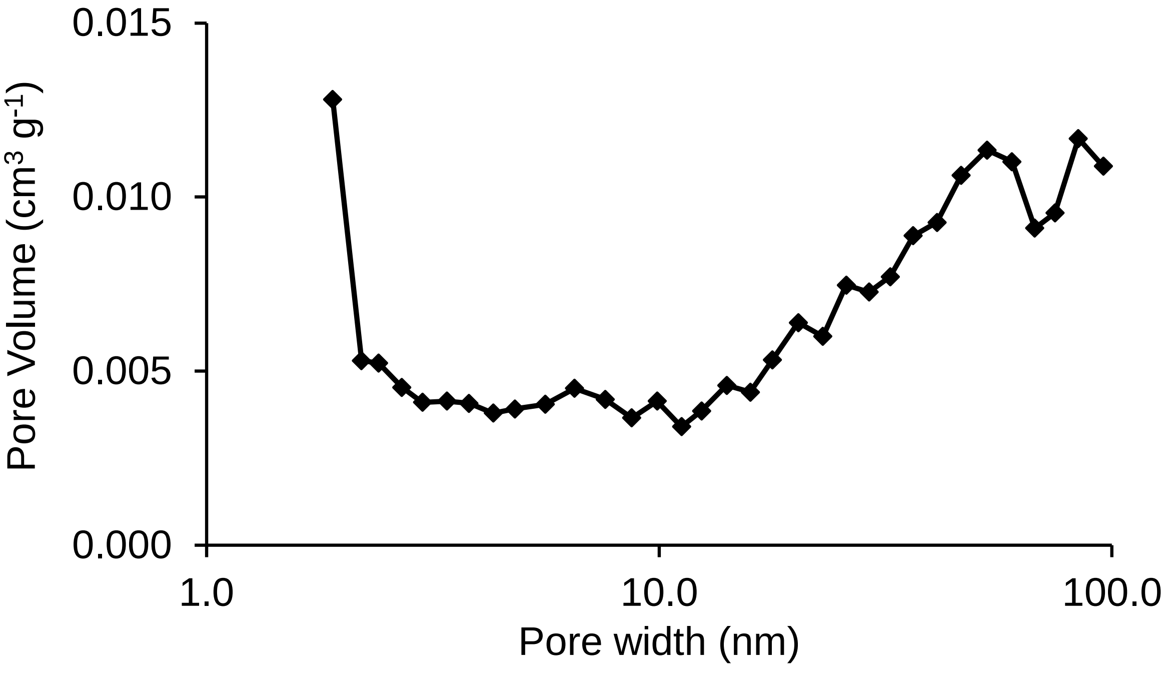

Porosimetry data for S800

Nitrogen Isotherm

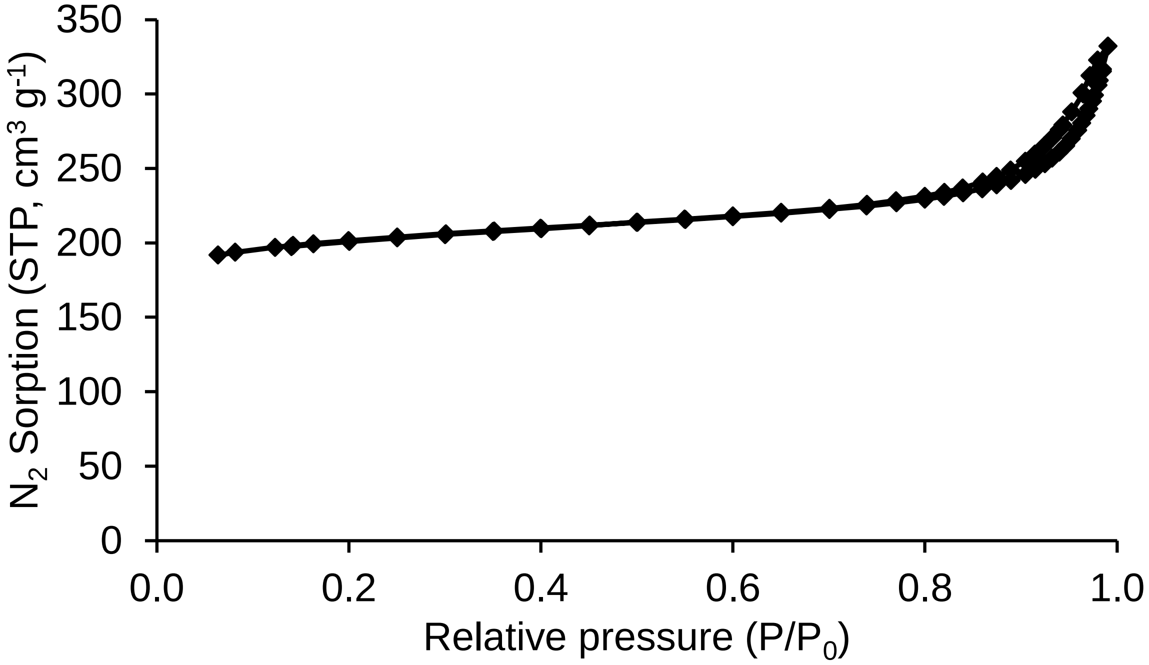

BJH Pore Size Distribution

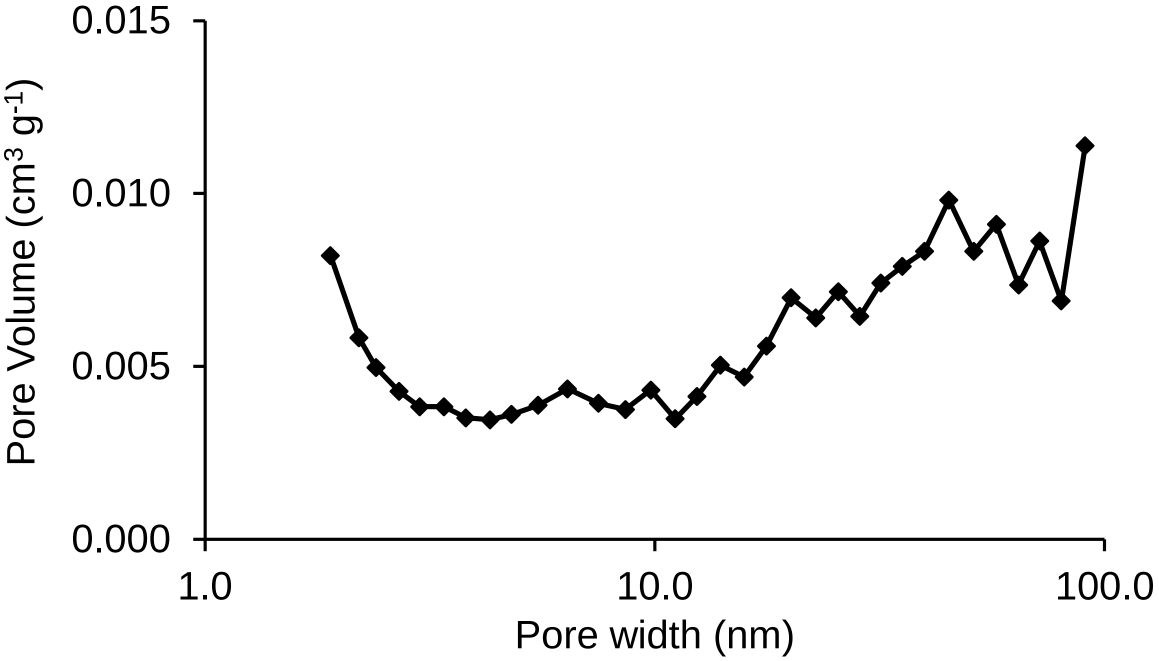

Porosimetry data for $SN_{Gly}800_{Th}$

Nitrogen Isotherm

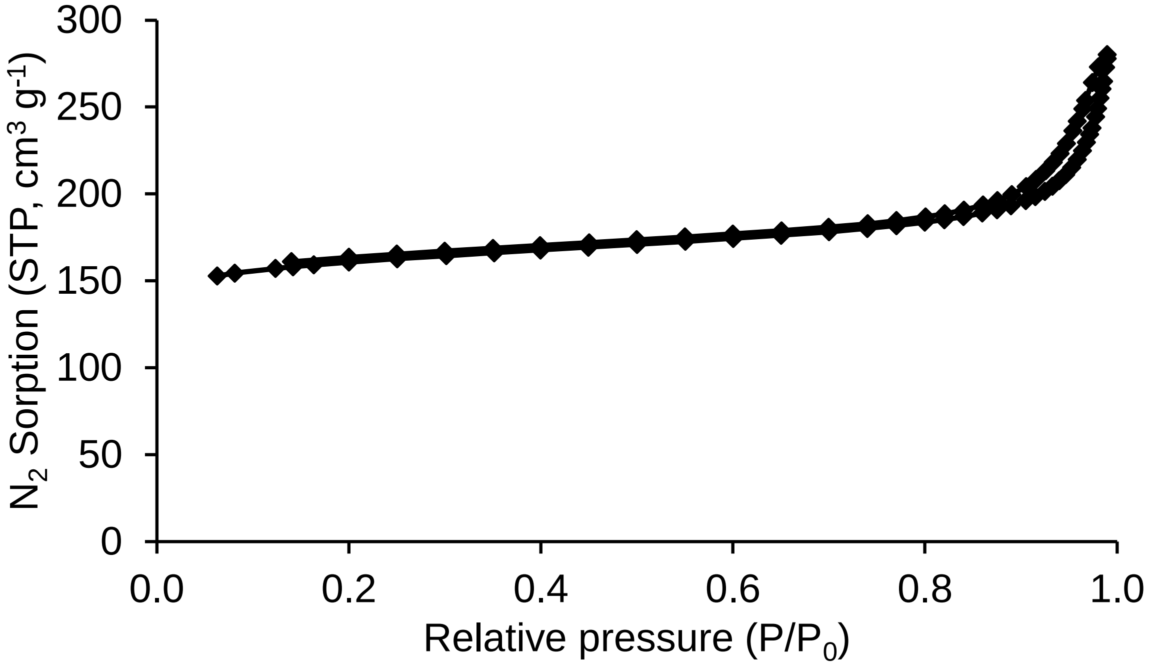

BJH Pore Size Distribution

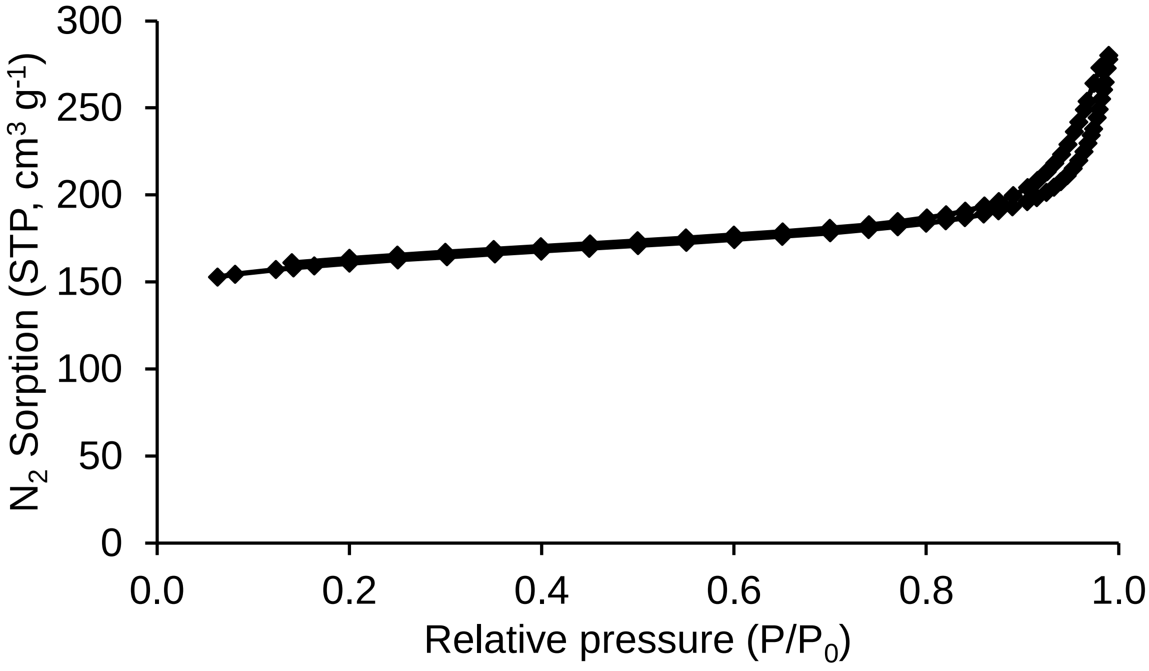

Porosimetry data for $SN_{Bal}800_{Th}$

Nitrogen Isotherm

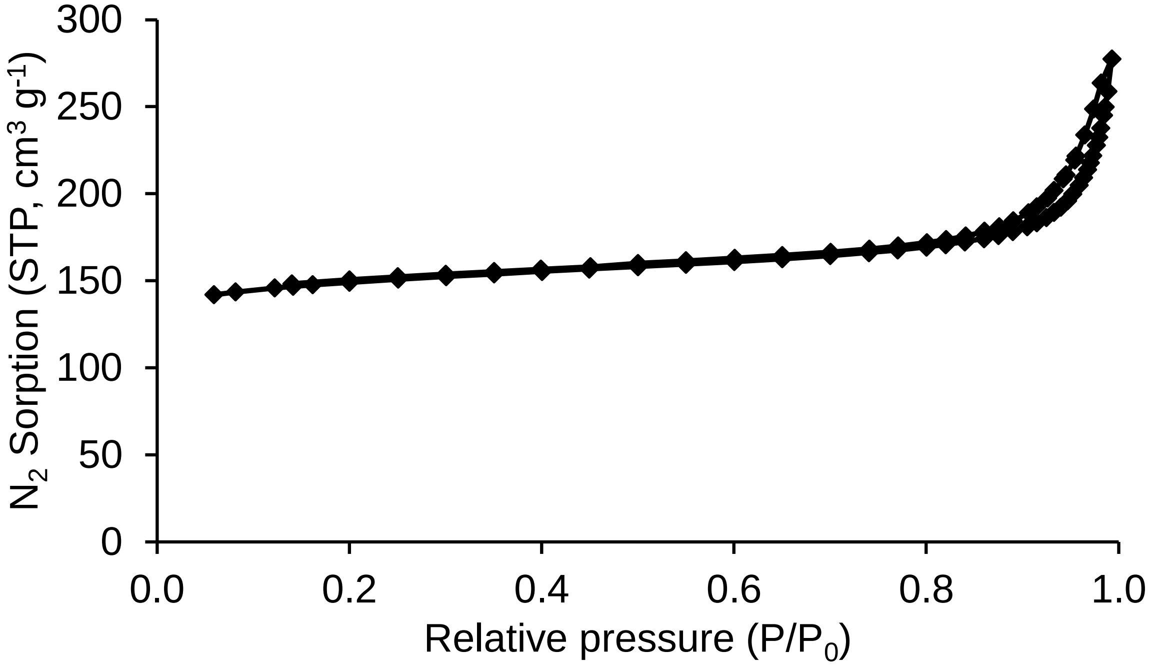

BJH Pore Size Distribution

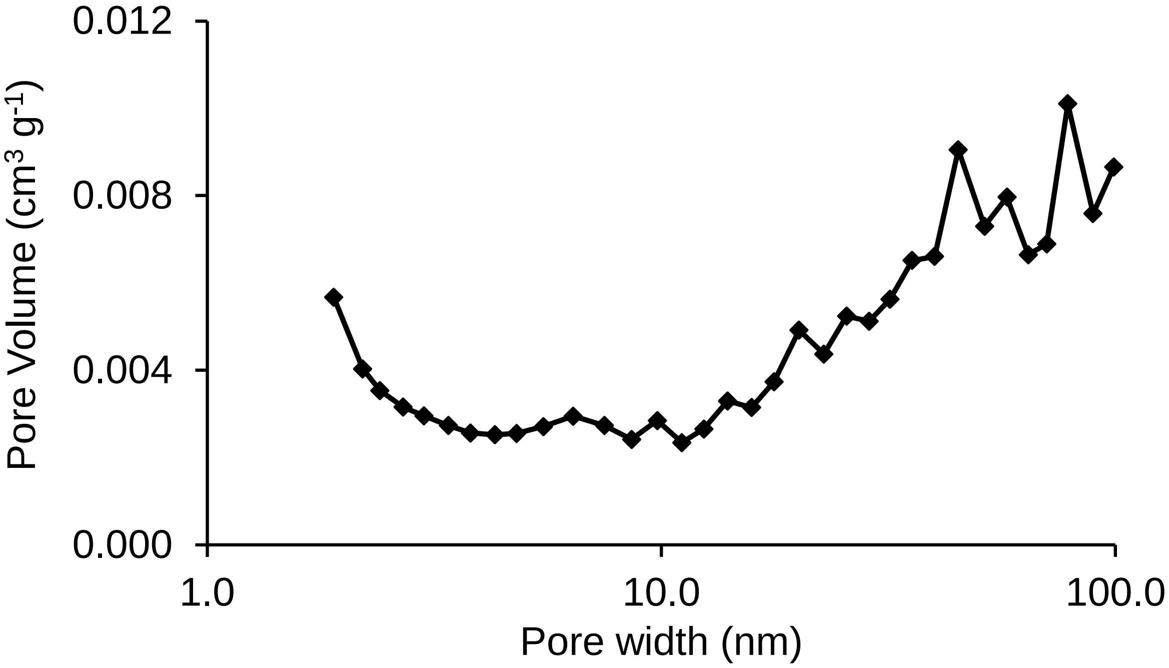

Porosimetry data for $SN_{Ure}800_{Th}$

Nitrogen Isotherm

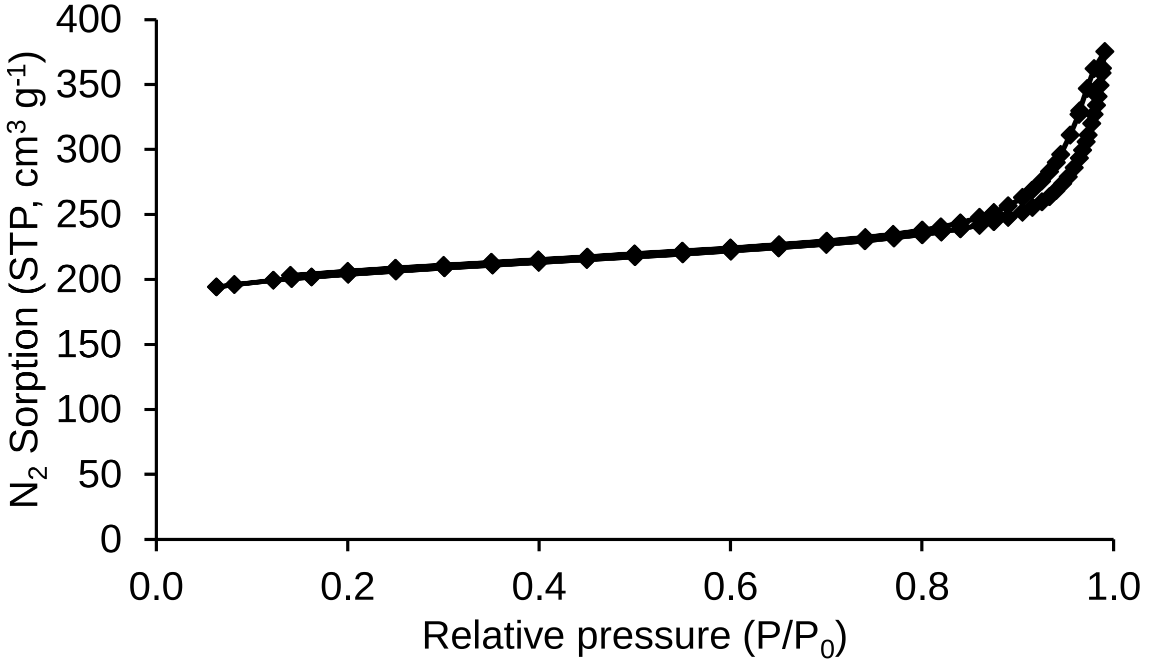

BJH Pore Size Distribution

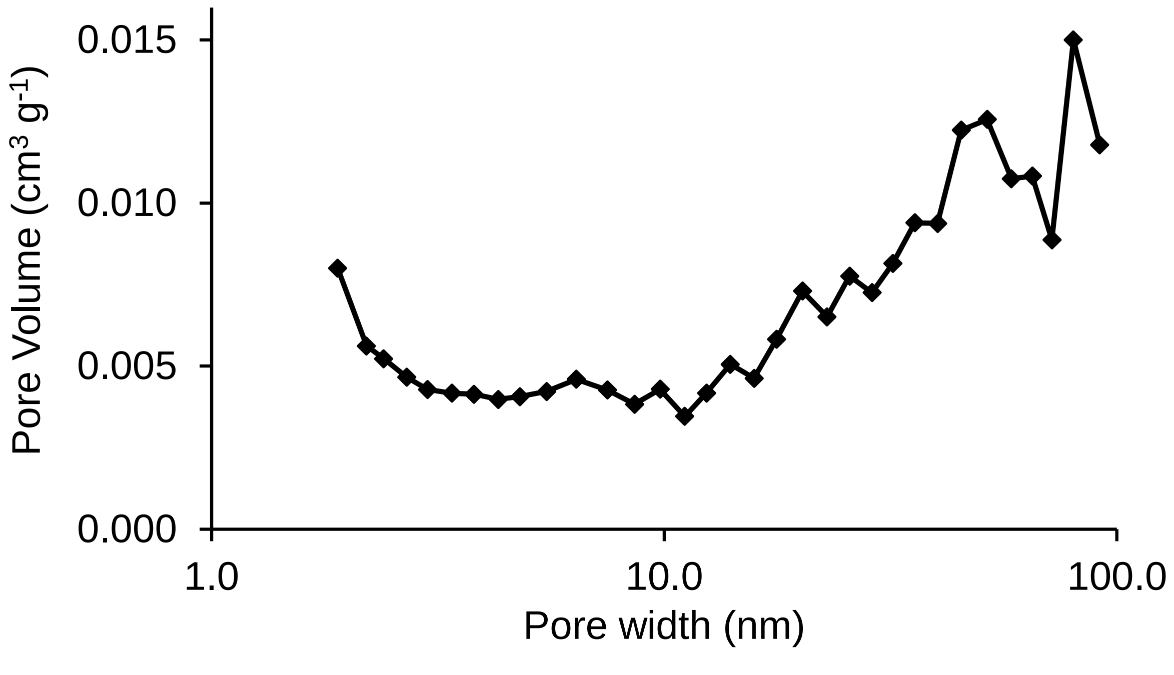

Porosimetry data for $SN_{Mel}800_{Th}$

Nitrogen Isotherm

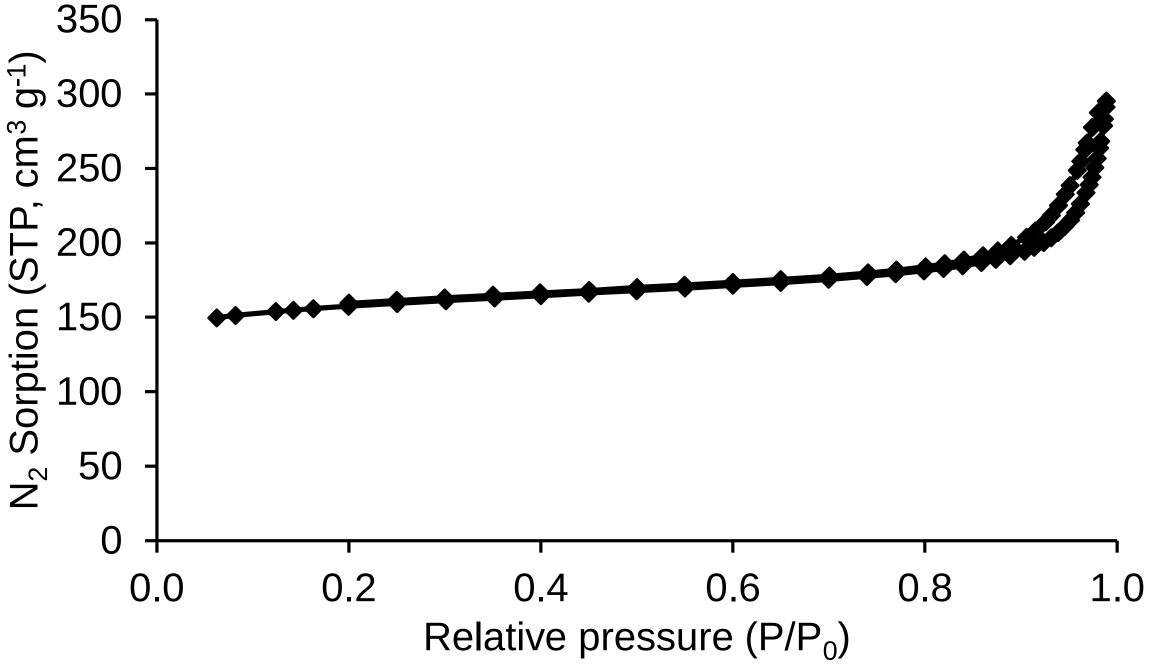

BJH Pore Size Distribution

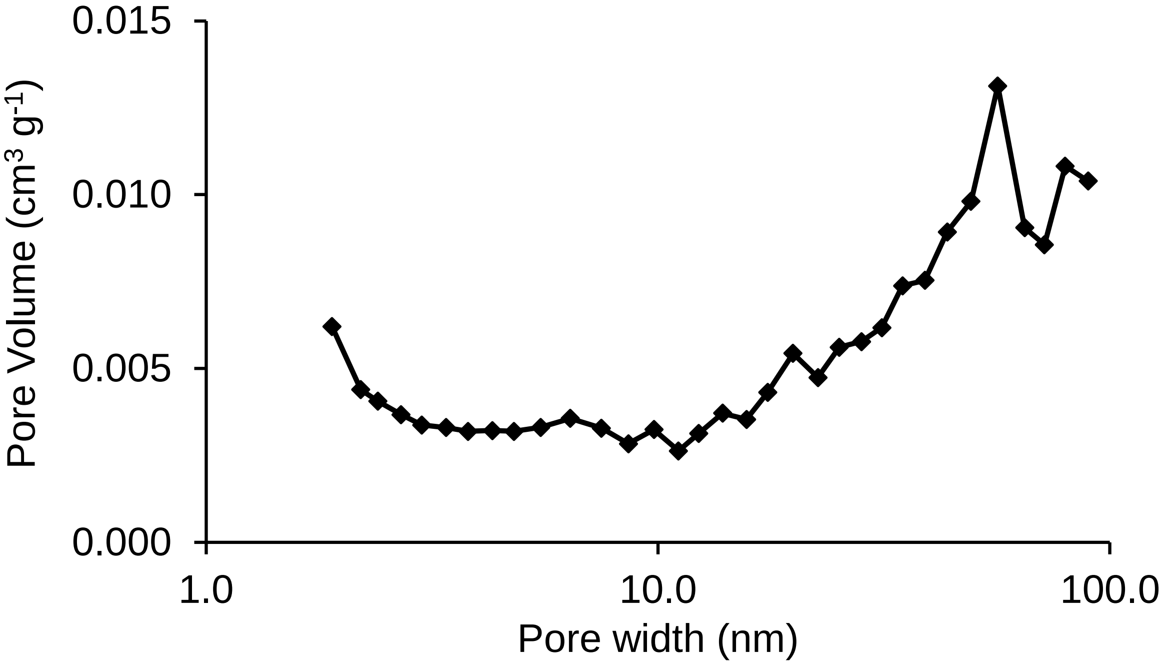

Porosimetry data for $SN_{Mel}800_{Th}$ (using 2 equivalents of melamine)

Nitrogen Isotherm

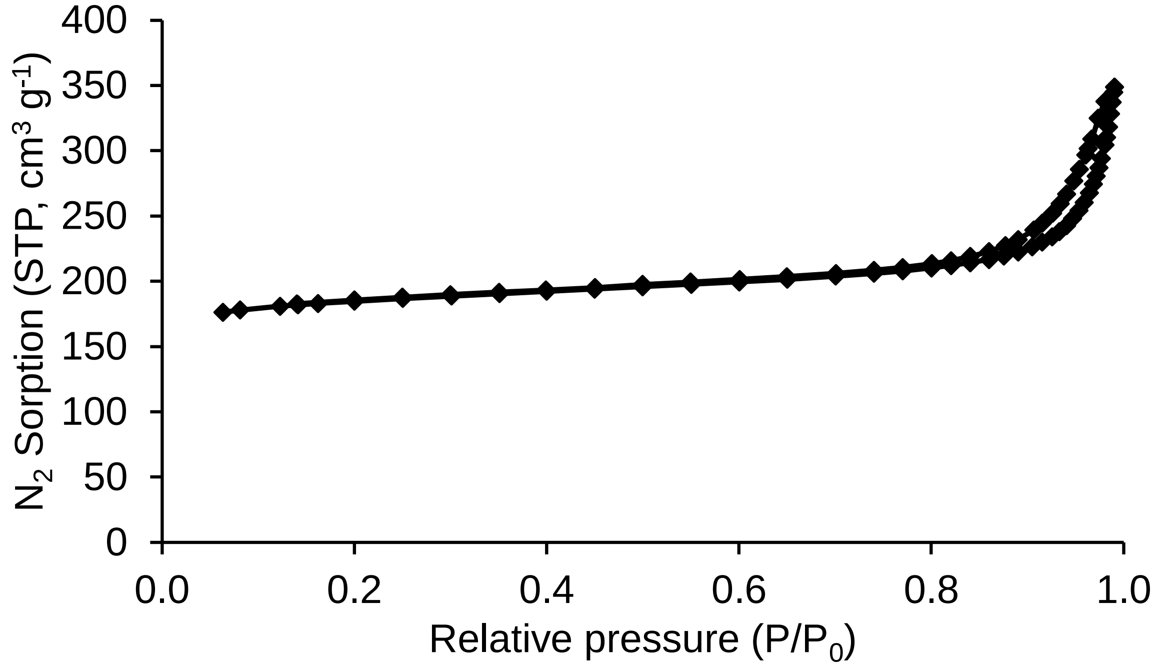

BJH Pore Size Distribution

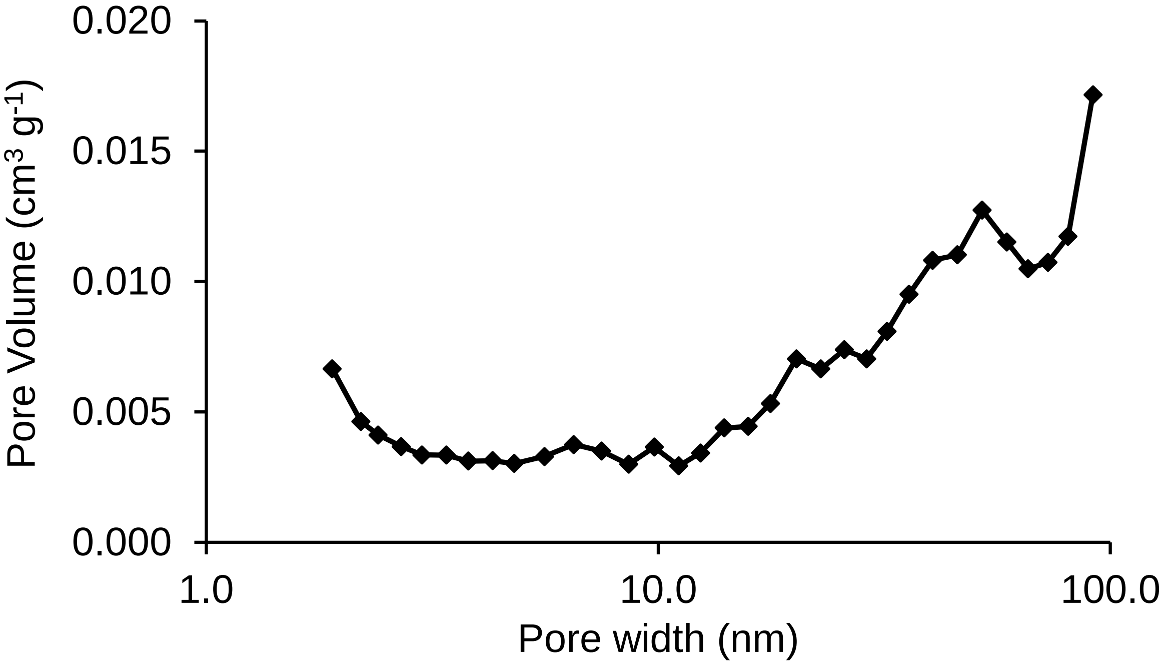

Porosimetry data for $SN_{Nic}800_{Th}$

Nitrogen Isotherm

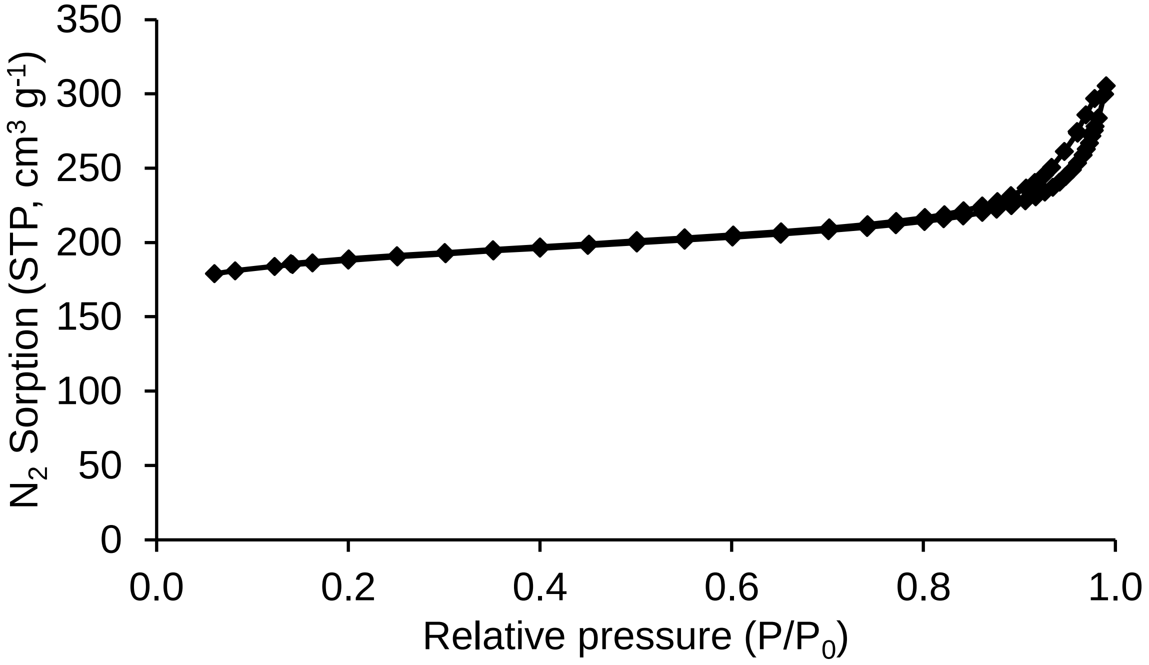

BJH Pore Size Distribution

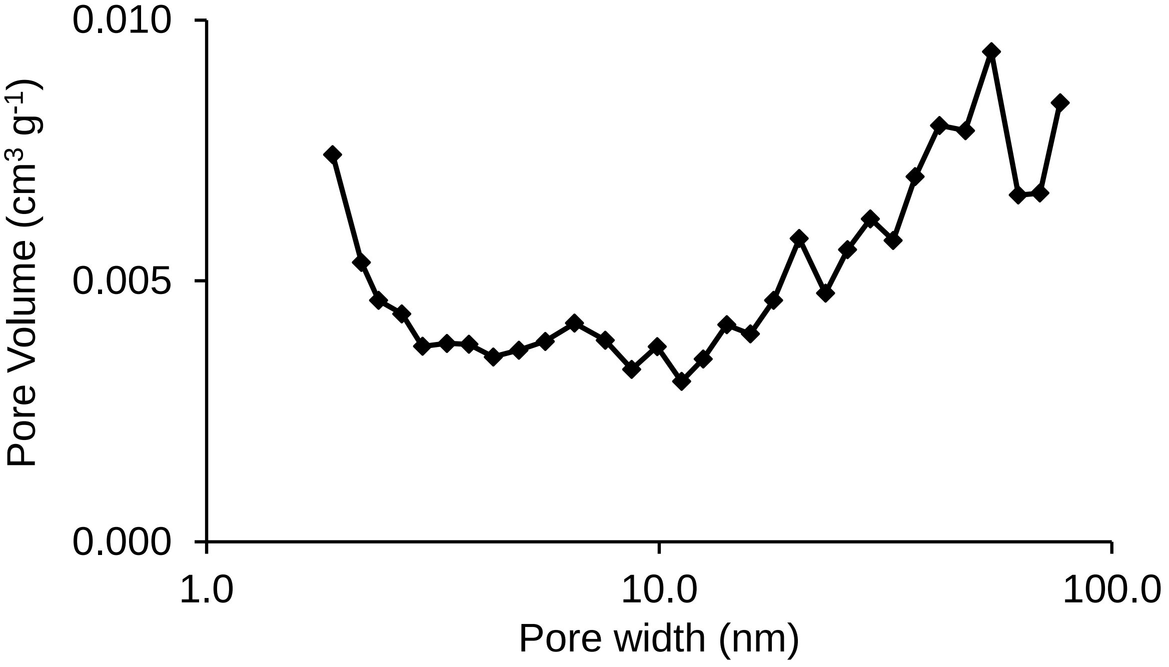

Porosimetry data for $SN_{Gly}800_{Mo}$

Nitrogen Isotherm

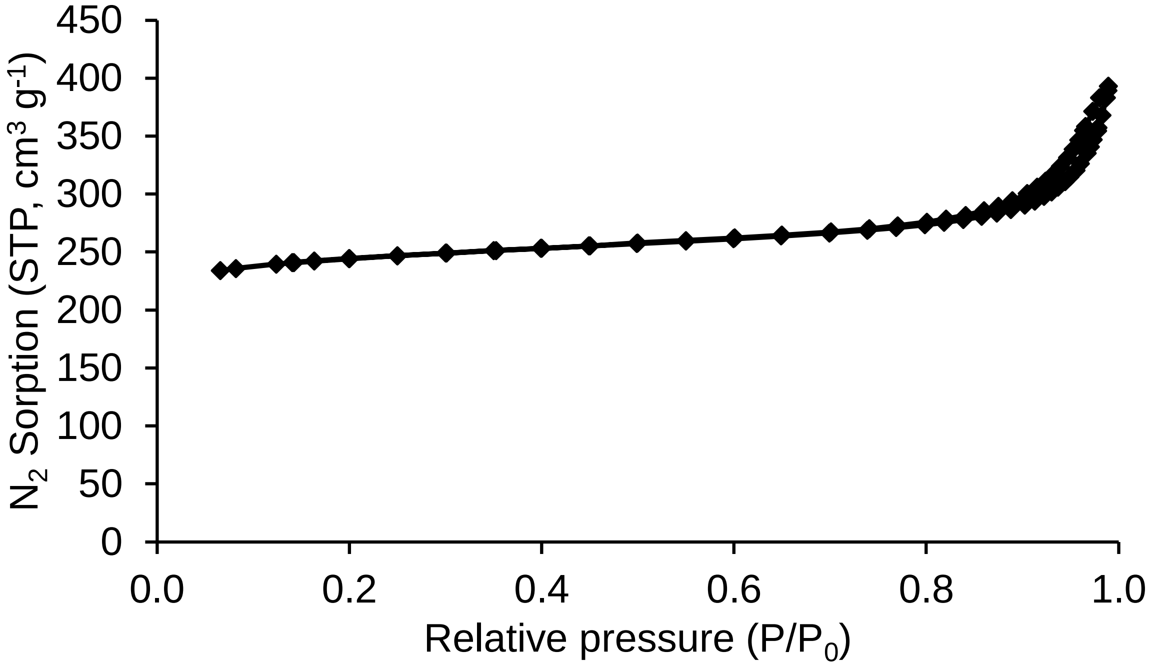

BJH Pore Size Distribution

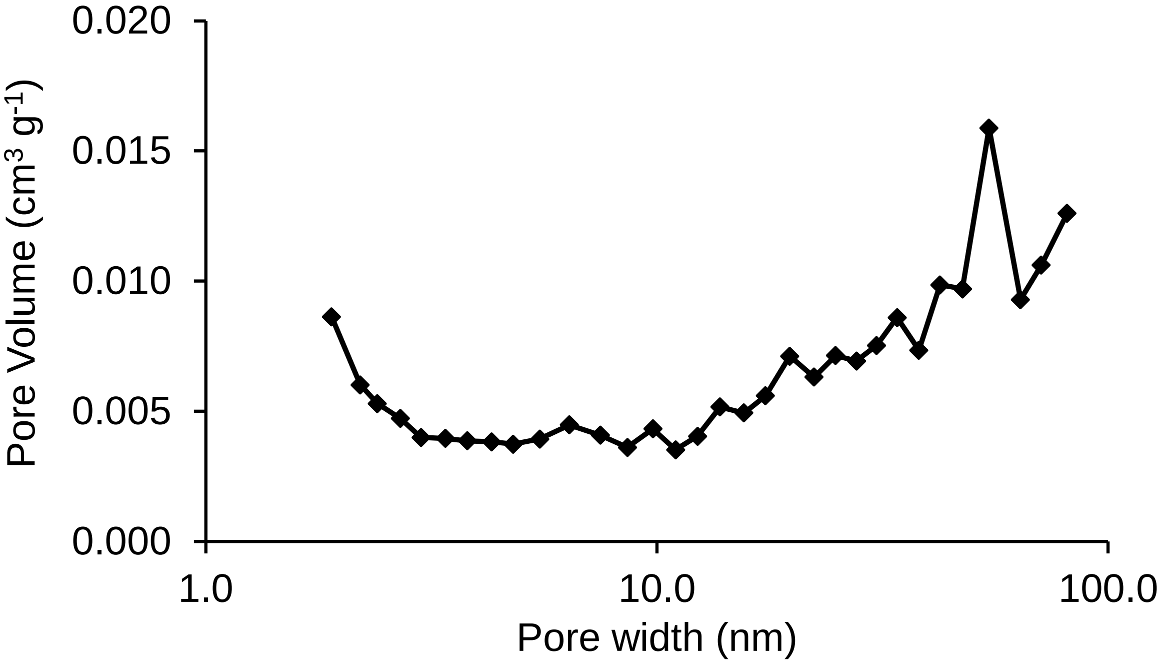

Porosimetry data for $SN_{Bal}800_{Mo}$

Nitrogen Isotherm

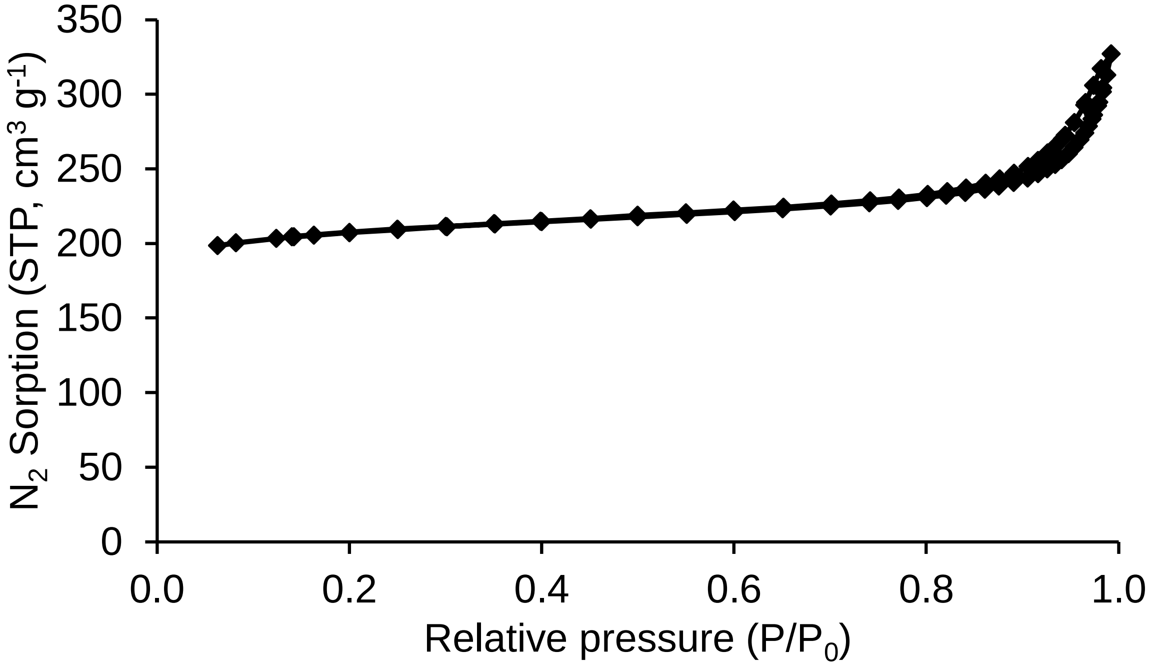

BJH Pore Size Distribution

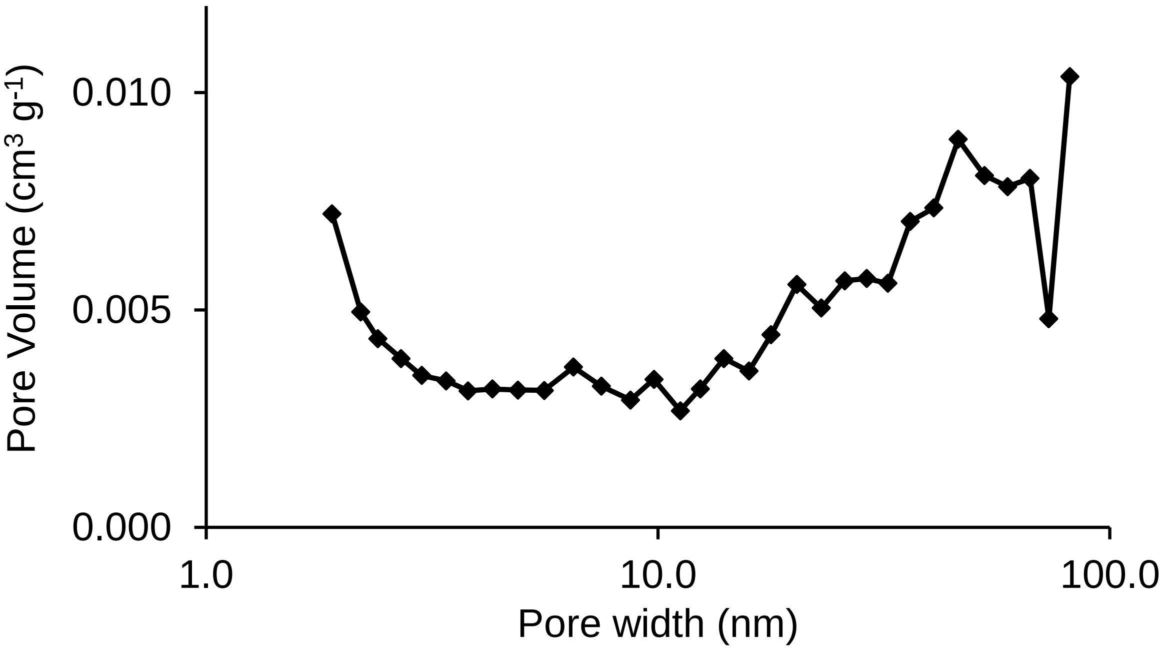

Porosimetry data for $SN_{Ure}800_{Mo}$

Nitrogen Isotherm

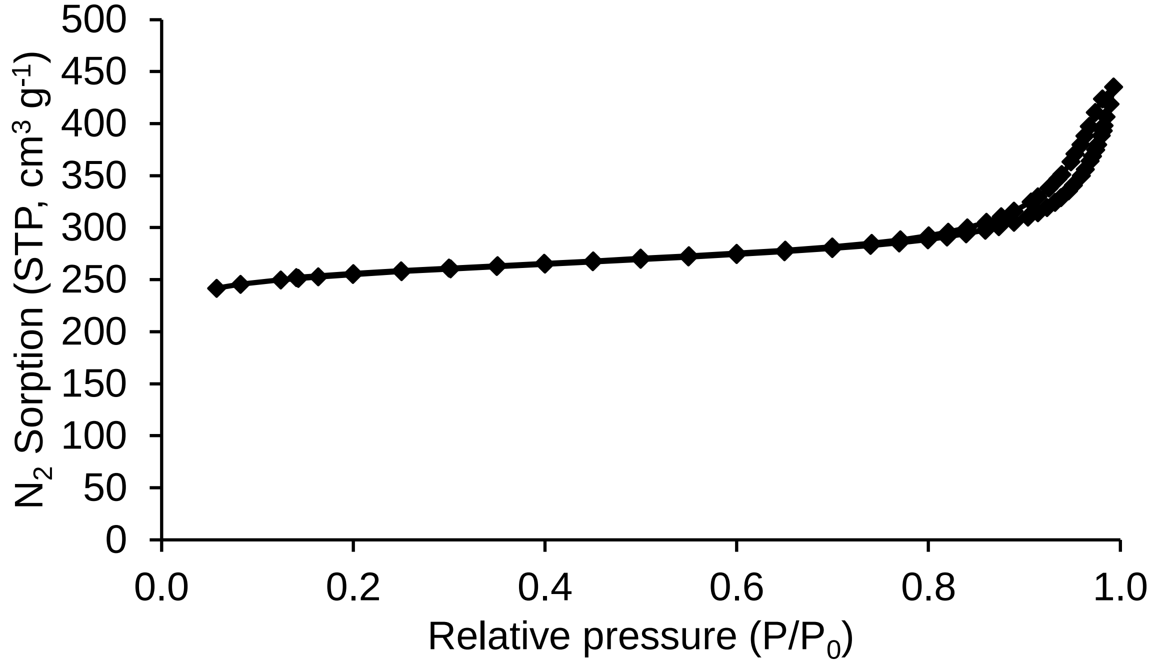

BJH Pore Size Distribution

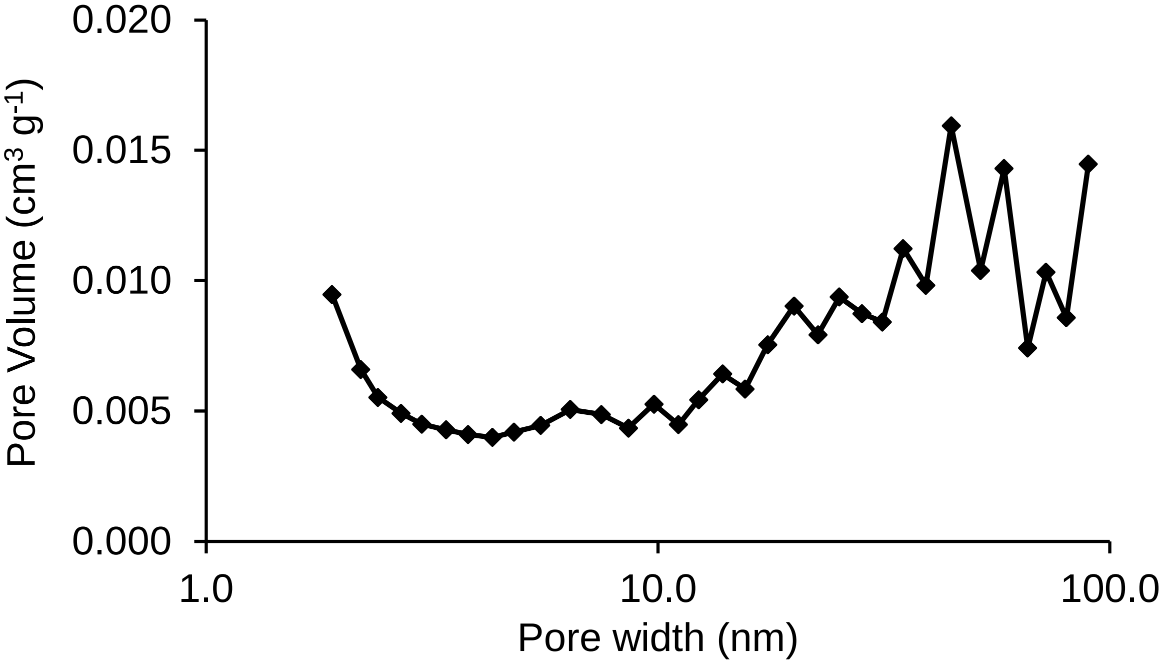

Porosimetry data for $SN_{Mel}800_{Mo}$

Nitrogen Isotherm

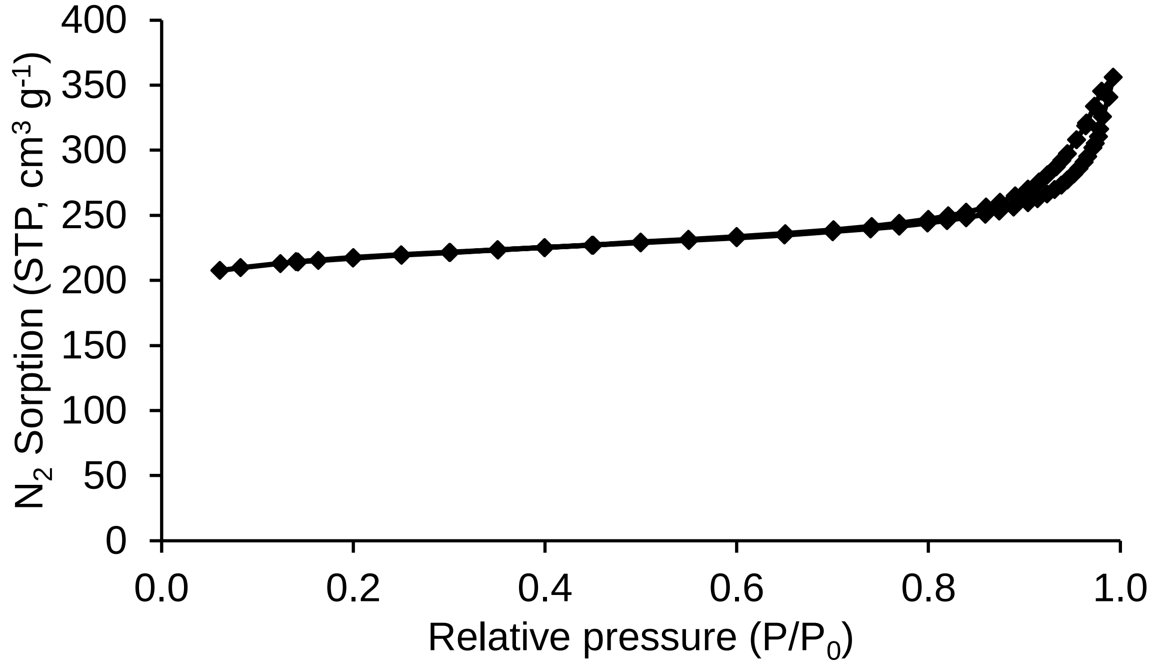

BJH Pore Size Distribution

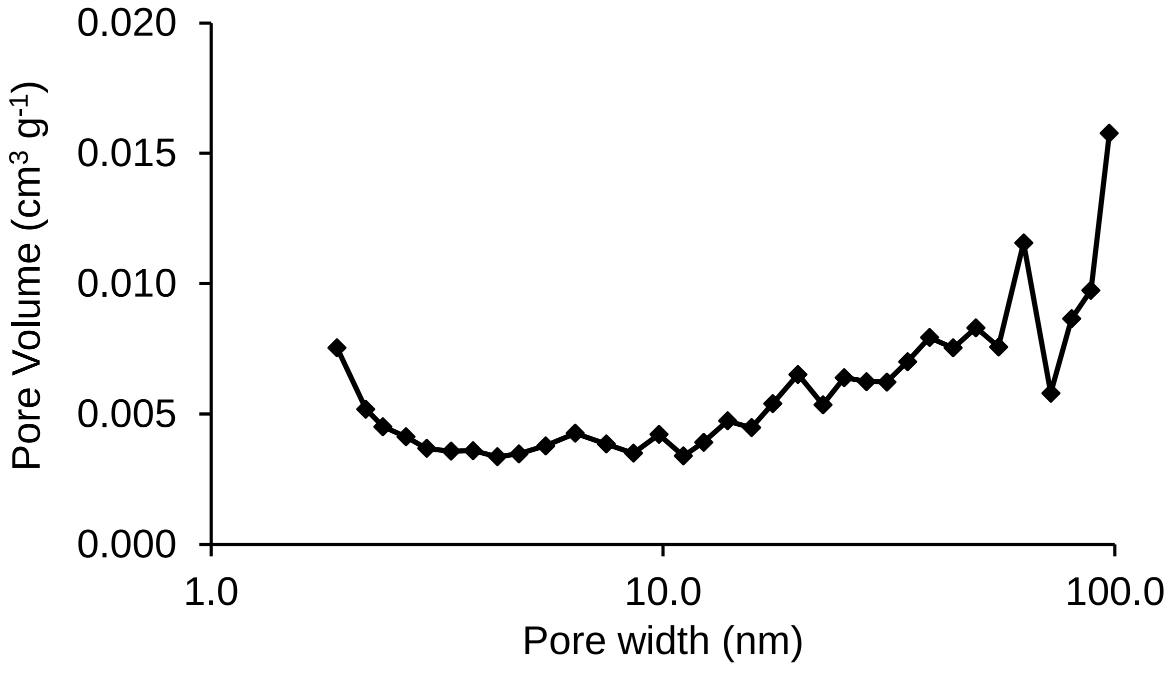

Porosimetry data for $SN_{Nic}800_{Mo}$

Nitrogen Isotherm

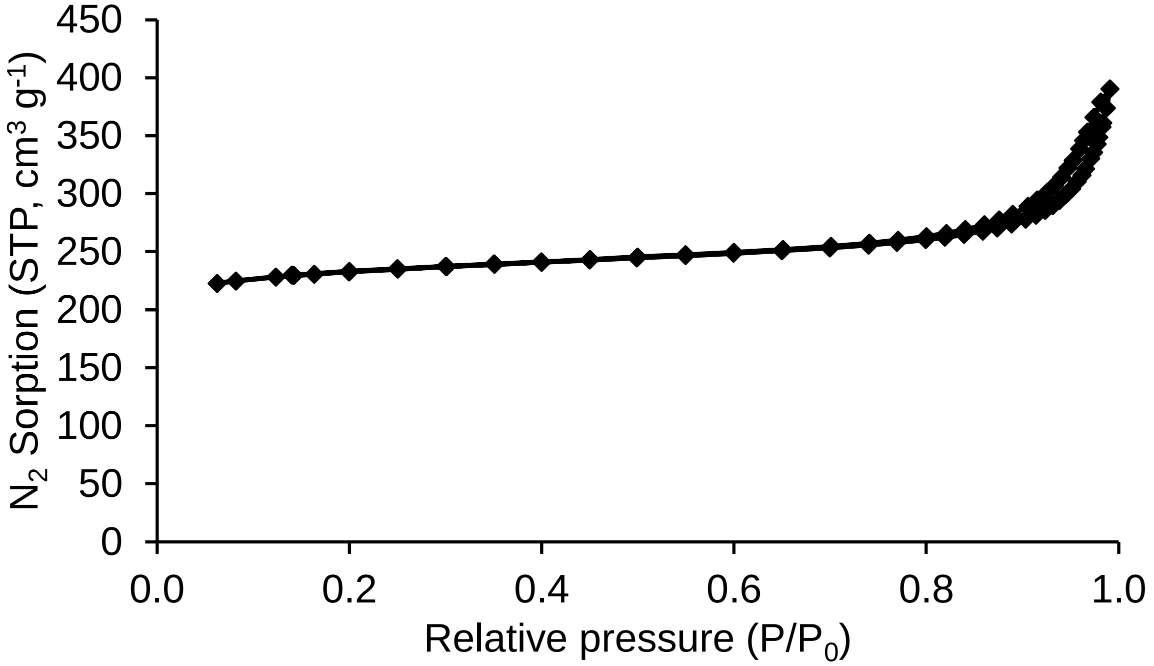

BJH Pore Size Distribution

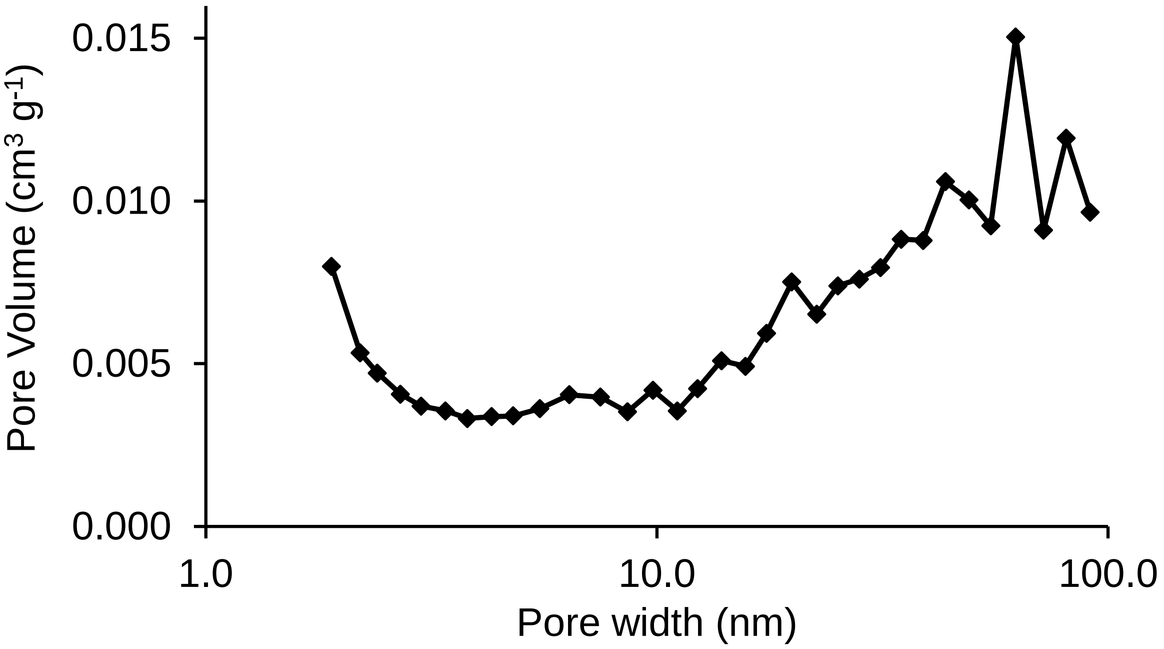

Porosimetry data for $SN_{Gly}800_{Mu}$

Nitrogen Isotherm

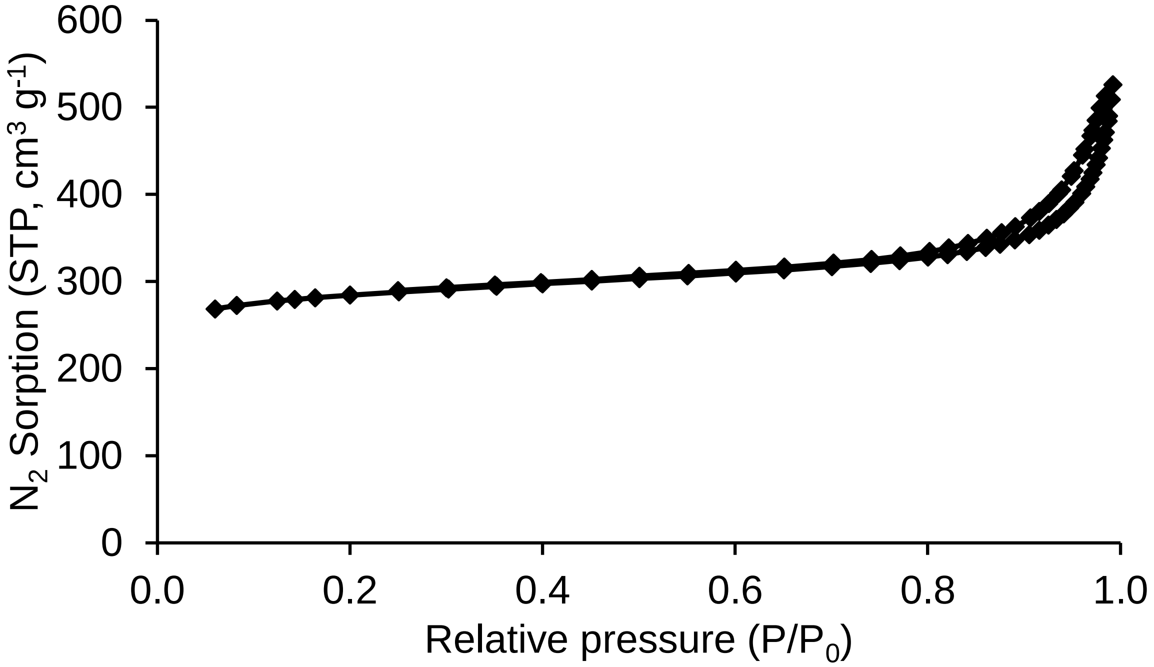

BJH Pore Size Distribution

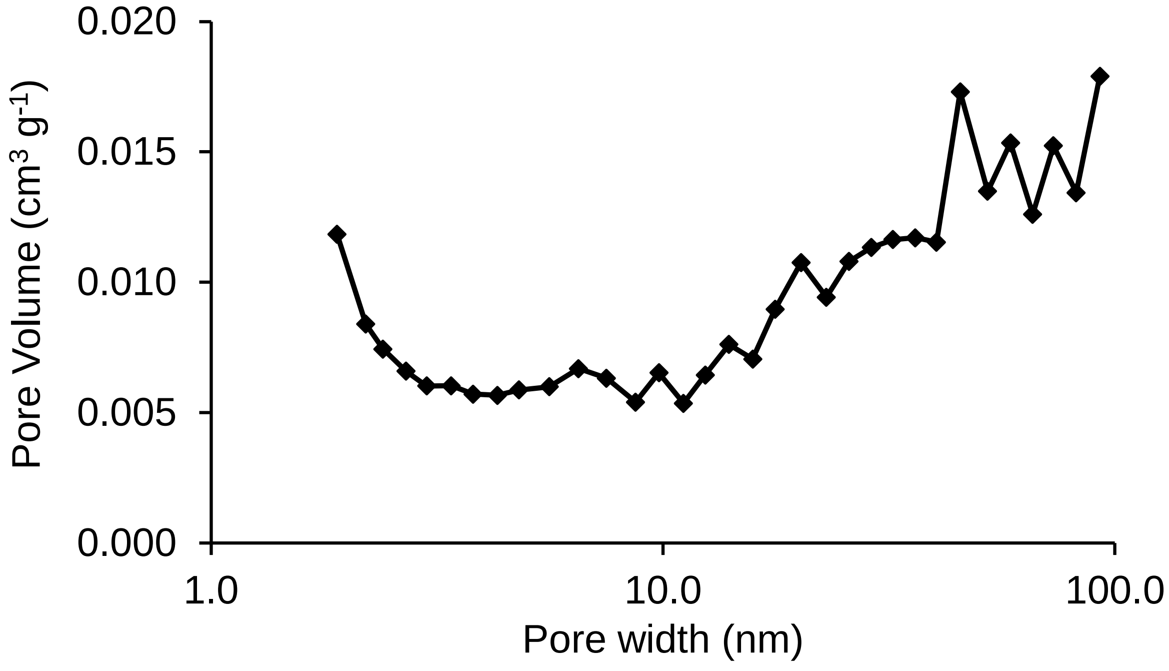

Porosimetry data for $SN_{Bal}800_{Mu}$

Nitrogen Isotherm

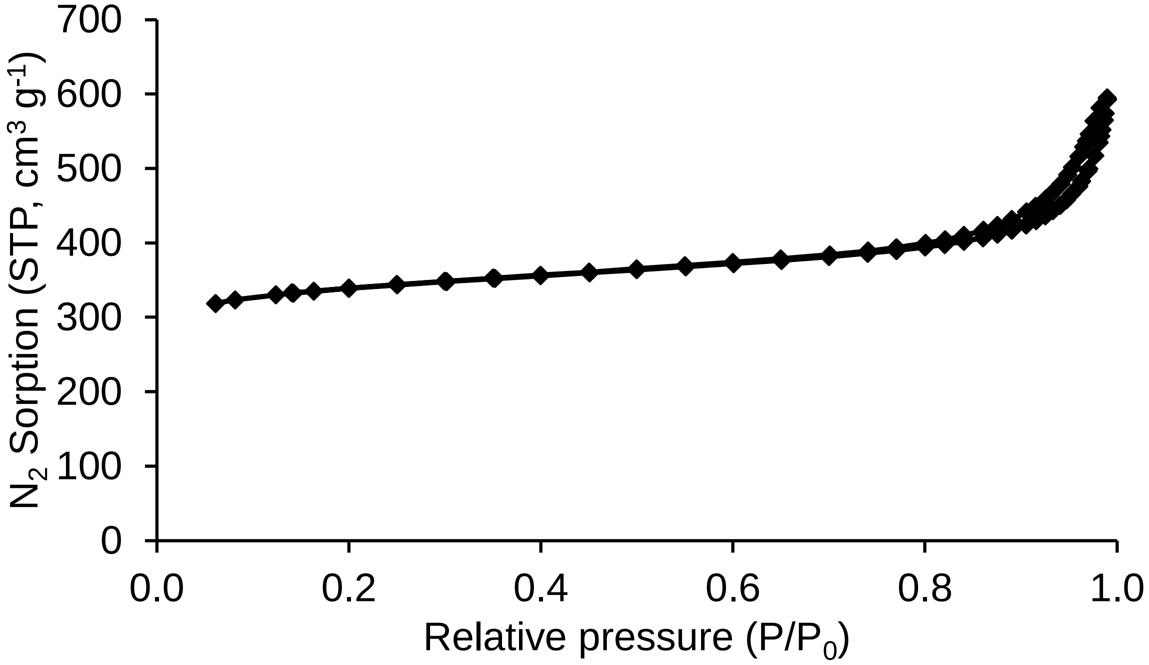

BJH Pore Size Distribution

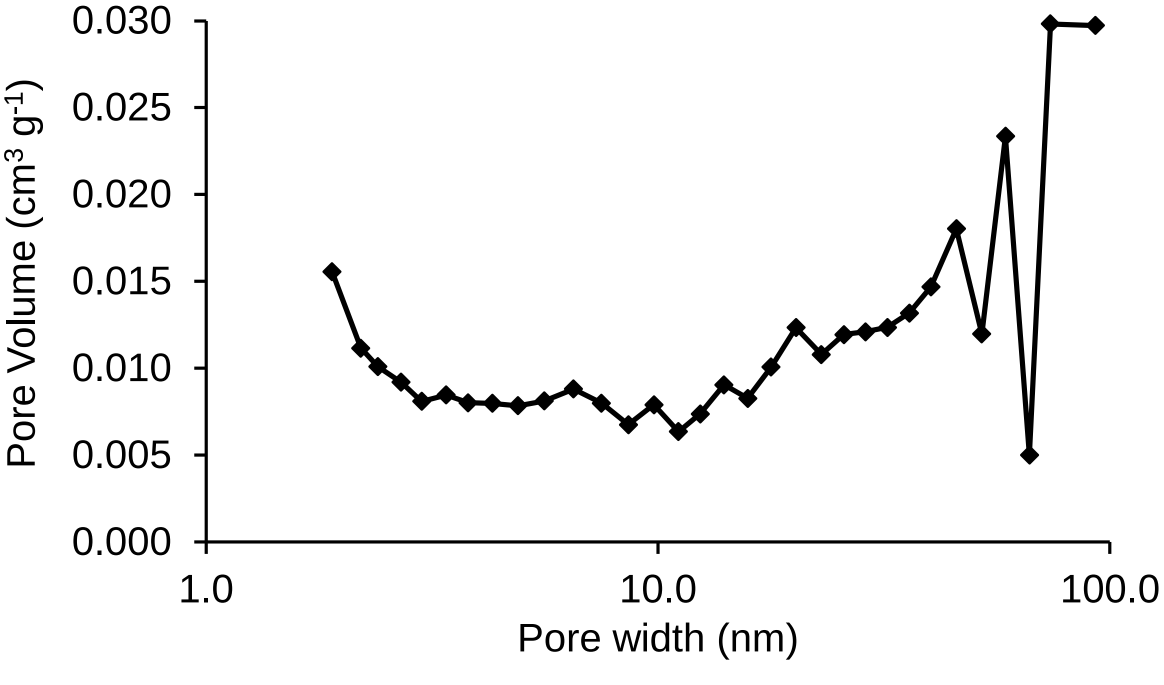

Porosimetry data for $SN_{Ure}800_{Mu}$

Nitrogen Isotherm

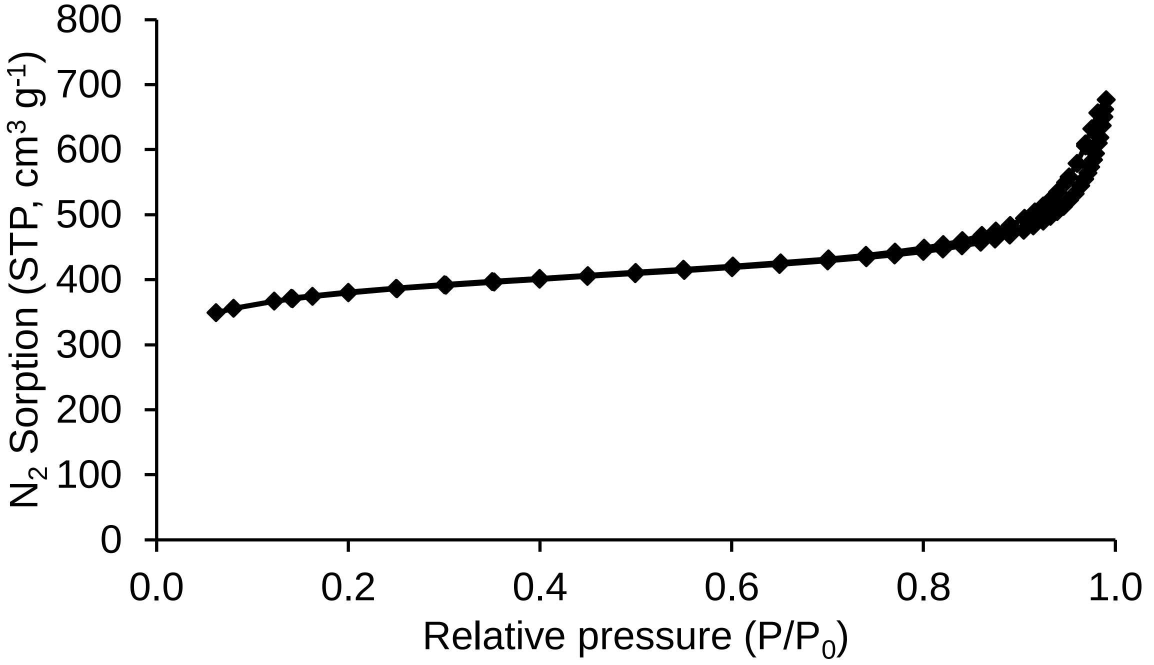

BJH Pore Size Distribution

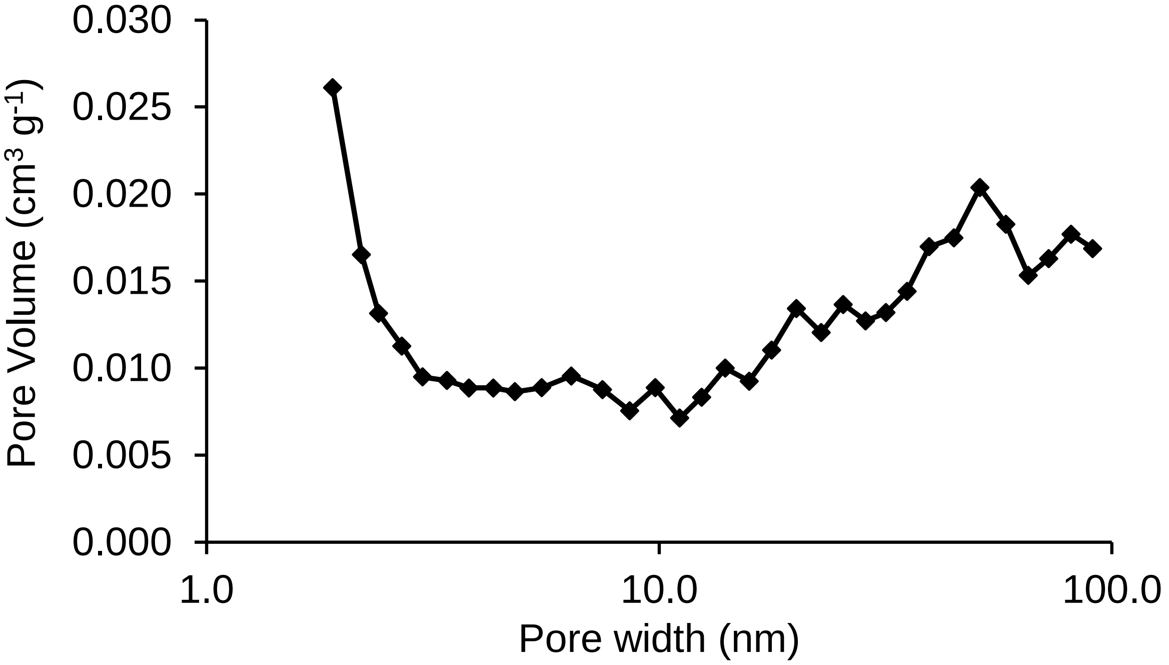

Porosimetry data for $SN_{Mel}800_{Mu}$

Nitrogen Isotherm

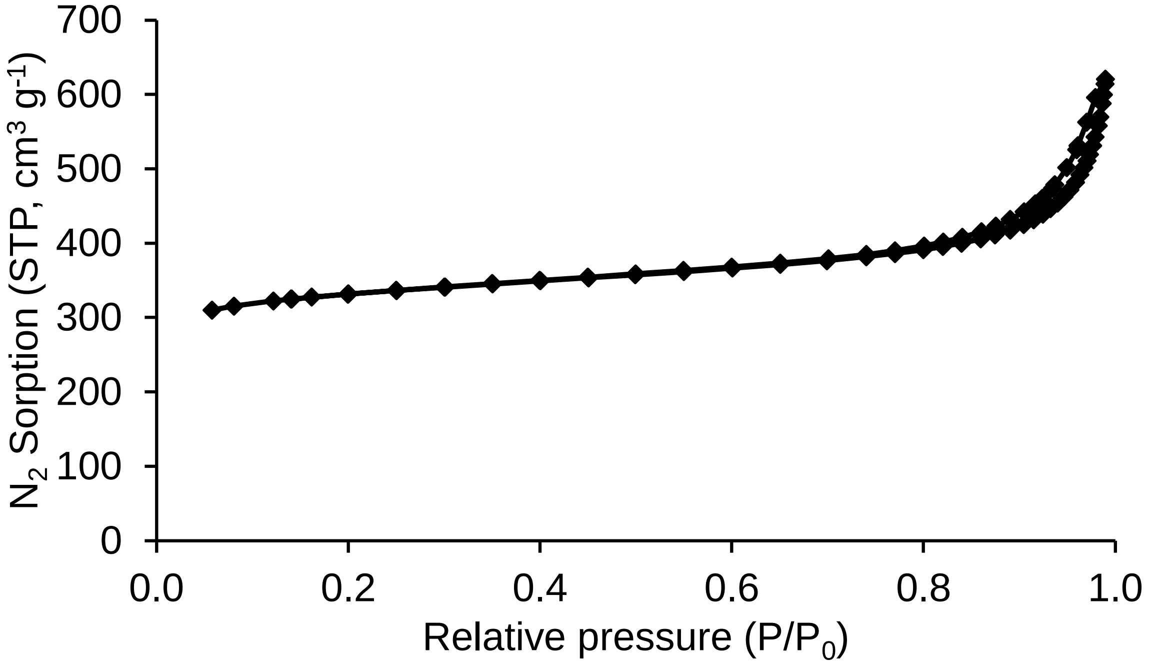

BJH Pore Size Distribution

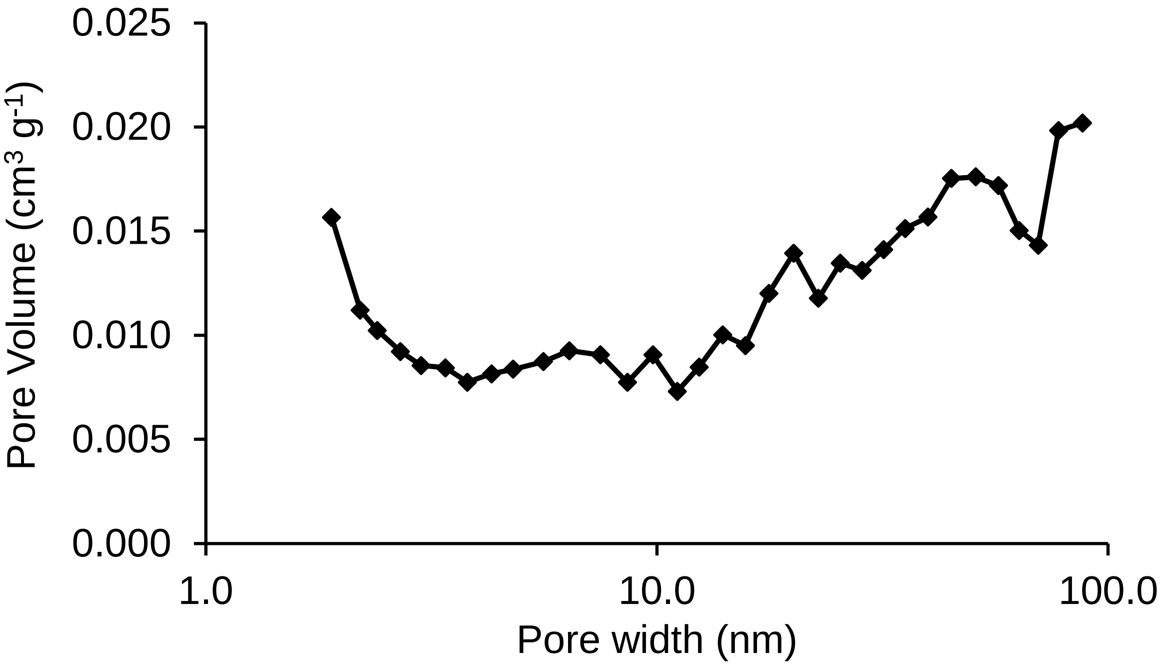

Porosimetry data for $SN_{Nic}800_{Mu}$

Nitrogen Isotherm

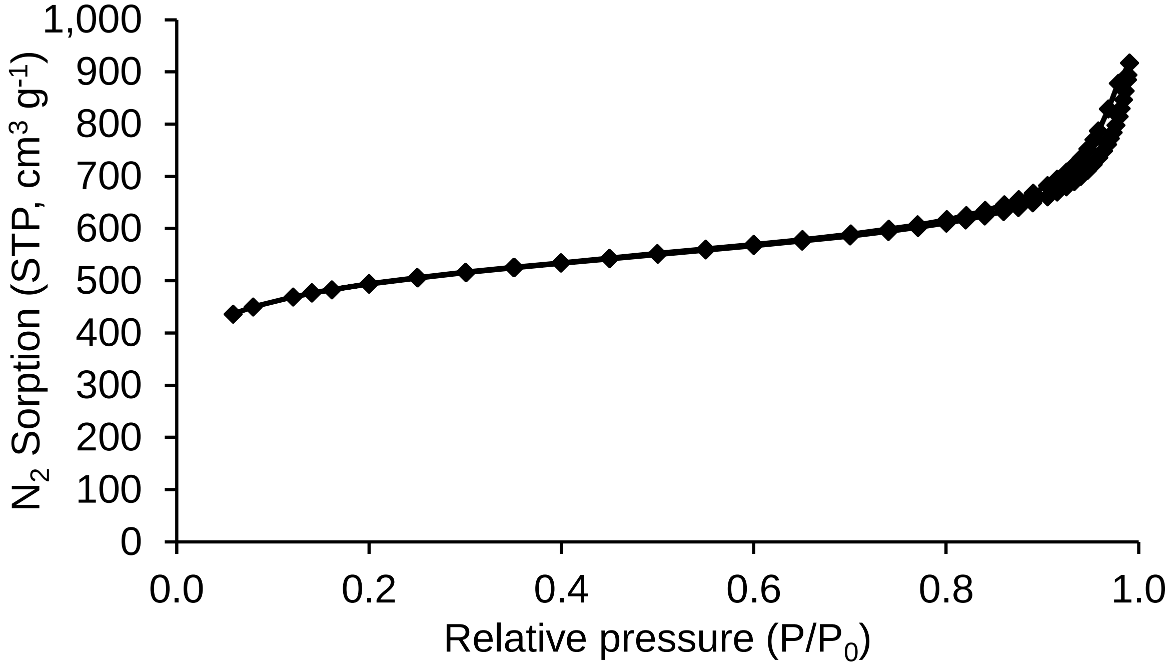

BJH Pore Size Distribution

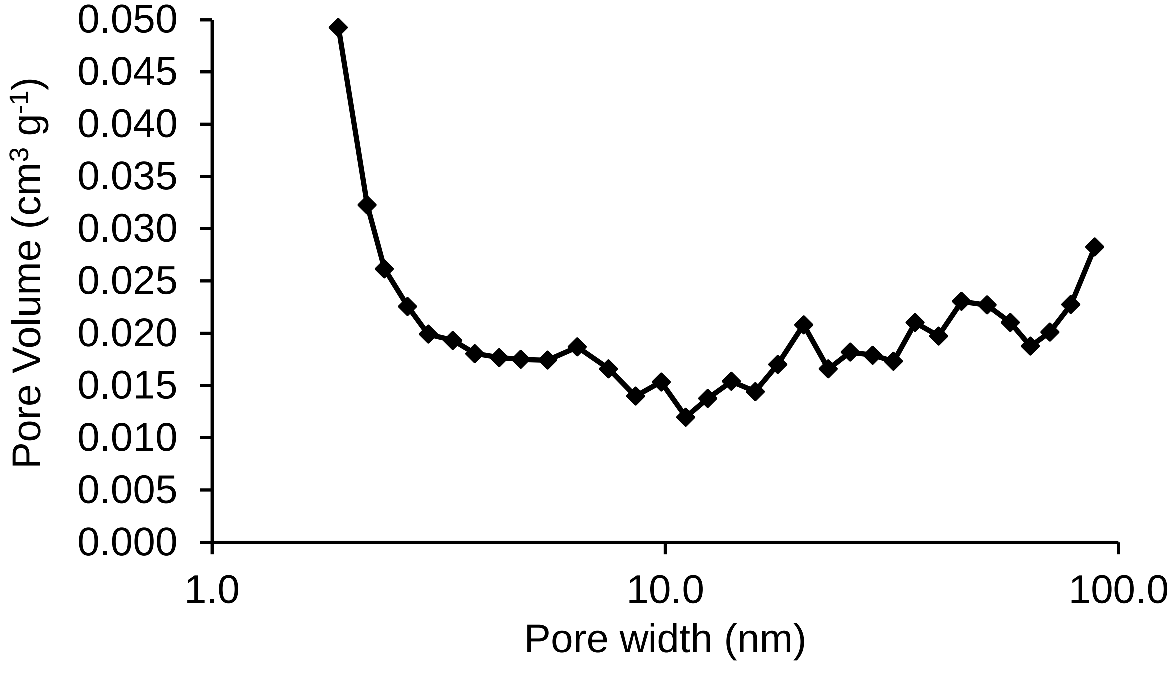

Combustion and XPS analysis of S300

Combustion analysis: C, 71.9%; H, 3.0%; N, 0.0%.

XPS analysis: C, 82.1%; N, 0.2%; O, 17.5%; S, 0.18%.

XPS Energies

Energy (eV)	Assignment	Atomic Concentration (%)
286.16	C-O-C / C-OH / C-OCO	14.72
287.7	O-C-O	3.95
289.1	COOH/Anhydride	1.77
284.32	C sp^2	21.1
290.68	π-π*	2.9
293.77	π-π*	0.66
284.71	Defective / sp^3	37.02
399.88	N 1s	0.16
531.45	O 1s	5.87
533.27	O 1s	11.14
536.02	O 1s	0.38
538.19	O 1s	0.13
167.08	S 2p	0.18

XPS carbon deconvolution

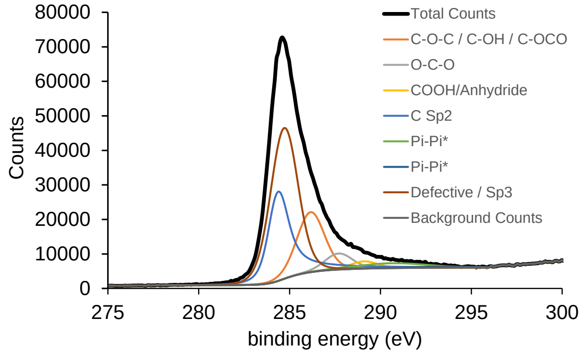

XPS oxygen deconvolution

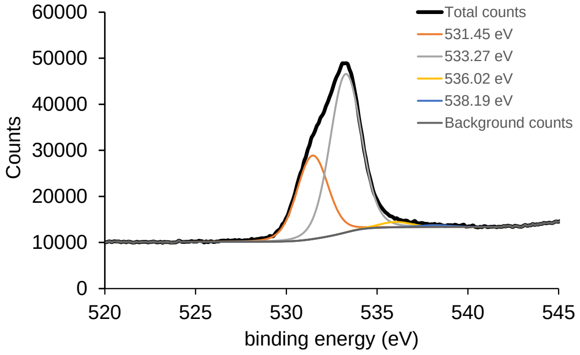

Combustion and XPS analysis of S800

Combustion analysis: C, 85.4%; H, 0.3%; N, 0.0%.

XPS analysis: C, 94.6%; N, 0.4%; O, 4.8%; S, 0.2%.

XPS Energies

Energy (eV)	Assignment	Atomic Concentration (%)
286.66	C-O-C / C-OH / C-OCO	5.49
288.1	O-C-O	2.35
289.1	COOH/Anhydride	1.77
284.39	C sp^2	63.89
290.74	π-π*	8.78
293.83	π-π*	2
284.7	Defective / sp^3	10.28
399.98	N 1s	0.44
530.64	O 1s	1.12
533.34	O 1s	2
535.73	O 1s	0.43
537.9	O 1s	0.24
531.79	O 1s	1.05
163.98	S 2p	0.17

XPS carbon deconvolution

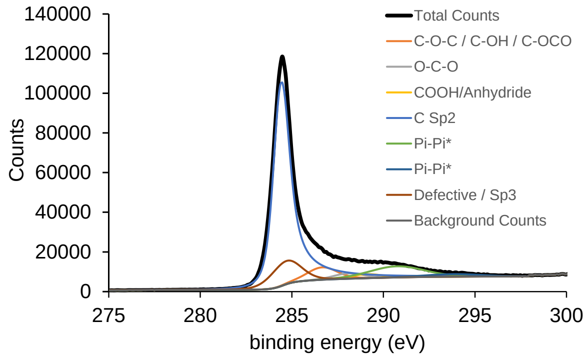

XPS oxygen deconvolution

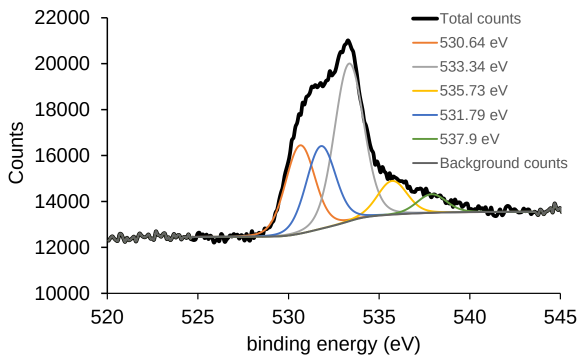

Combustion and XPS analysis of $SN_{Gly}300_{Th}$

Combustion analysis: C, 70.4%; H, 2.9%; N, 1.3%.

XPS analysis: C, 83.5%; N, 1.4%; O, 15.0%; S, 0.1%.

XPS Energies

Energy (eV)	Assignment	Atomic Concentration (%)
286.13	C-O-C / C-OH / C-OCO	12.76
287.7	O-C-O	4.11
289.14	COOH/Anhydride	1.66
284.3	C sp^2	26.48
290.66	π-π*	3.64
293.75	π-π*	0.83
284.73	Defective / sp^3	34.01
398.69	N 1s	0.46
400.02	N 1s	0.85
401.86	N 1s	0.11
531.38	O 1s	5.28
533.32	O 1s	9.03
536	O 1s	0.45
538.25	O 1s	0.22
167.58	S 2p	0.11

XPS carbon deconvolution

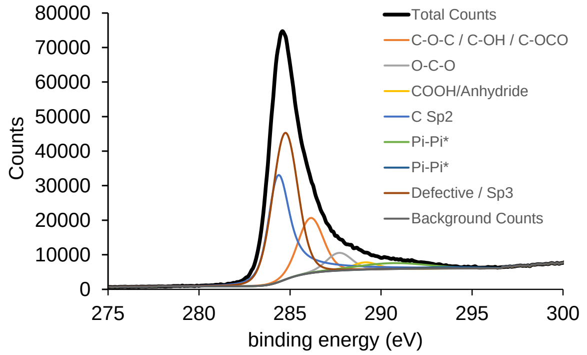

XPS oxygen deconvolution

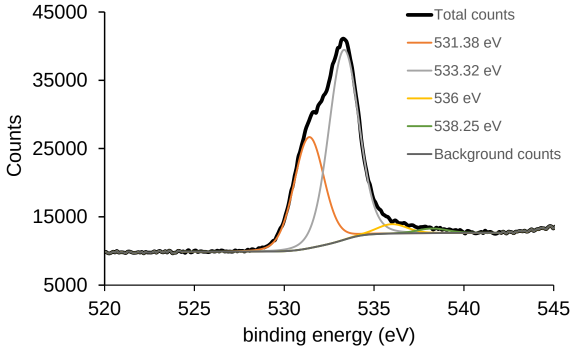

XPS nitrogen deconvolution

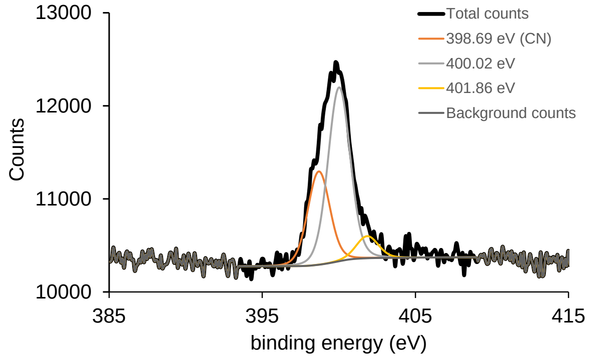

Combustion and XPS analysis of $SN_{Bal}300_{Th}$

Combustion analysis: C, 71.7%; H, 3.0%; N, 4.3%.

XPS analysis: C, 82.4%; N, 3.7%; O, 13.8%; S, 0.1%.

XPS Energies

**Energy (eV)**	**Assignment**	**Atomic Concentration (%)**
286.07	C-O-C / C-OH / C-OCO	12.49
287.7	O-C-O	3.86
289.11	COOH/Anhydride	1.24
284.23	C sp^2	26.8
290.59	π-π*	3.68
293.68	π-π*	0.84
284.7	Defective / sp^3	33.53
398.89	N 1s	1.66
400.06	N 1s	1.93
402.86	N 1s	0.13
531.21	O 1s	5.43
533.24	O 1s	7.85
535.98	O 1s	0.28
538.14	O 1s	0.19
167.58	S 2p	0.08

XPS carbon deconvolution

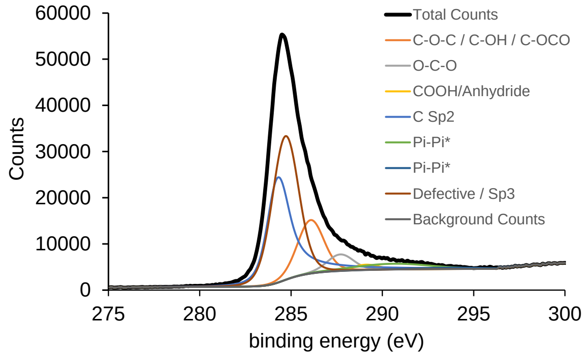

XPS oxygen deconvolution

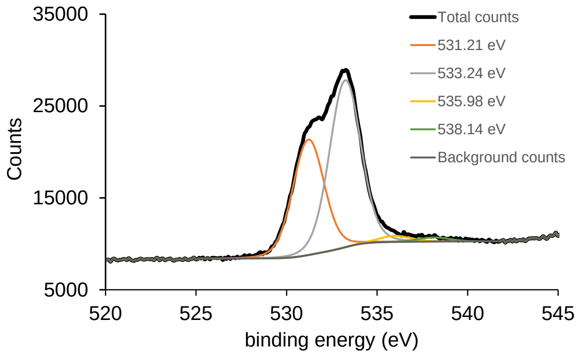

XPS nitrogen deconvolution

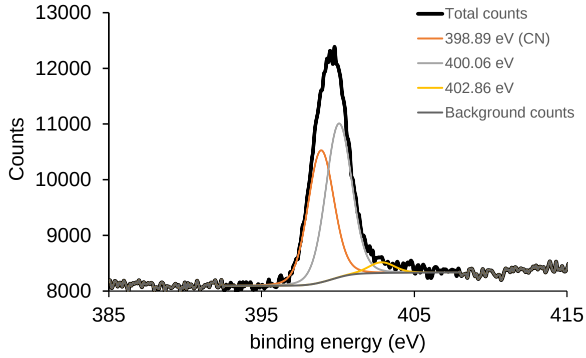

Combustion and XPS analysis of SN$_{Ure}$300$_{Th}$

Combustion analysis: C, 69.9%; H, 2.4%; N, 1.5%.

XPS analysis: C, 82.5%; N, 1.7%; O, 15.8%; S, 0.1%.

XPS Energies

Energy (eV)	Assignment	Atomic Concentration (%)
286.2	C-O-C / C-OH / C-OCO	12.81
287.86	O-C-O	4.02
289.32	COOH/Anhydride	1.74
284.47	C sp^2	29.8
290.83	π-π*	4.09
293.92	π-π*	0.93
284.77	Defective / sp^3	29.1
400.26	N 1s	0.93
399.07	N 1s	0.77
531.41	O 1s	5.65
533.4	O 1s	9.36
536.15	O 1s	0.5
538.53	O 1s	0.25
168.38	S 2p	0.05

XPS carbon deconvolution

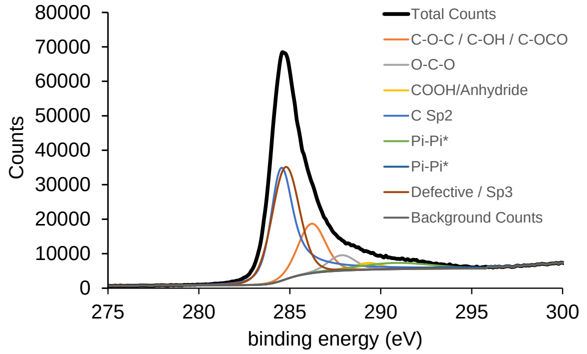

XPS oxygen deconvolution

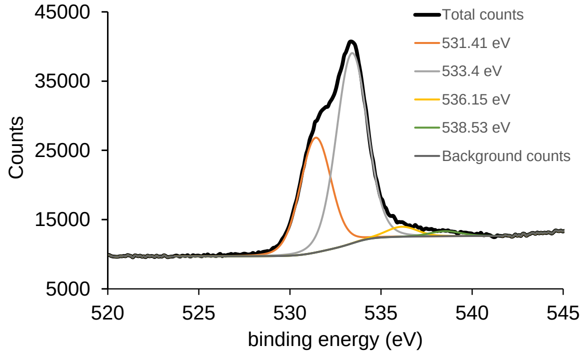

XPS nitrogen deconvolution

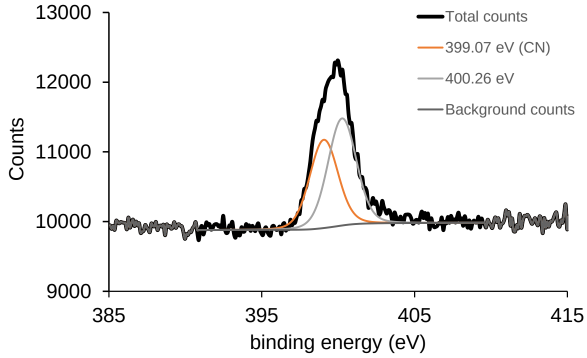

Combustion and XPS analysis of $SN_{Mel}300_{Th}$

Combustion analysis: C, 65.6%; H, 2.0%; N, 9.5%.

XPS analysis: C, 76.6%; N, 10.6%; O, 12.7%; S, 0.1%.

XPS Energies

Energy (eV)	Assignment	Atomic Concentration (%)
286.1	C-O-C / C-OH / C-OCO	10.61
287.85	O-C-O	7.97
289.26	COOH/Anhydride	1.22
284.36	C sp^2	27.66
290.71	π-π*	3.8
293.8	π-π*	0.87
284.7	Defective / sp^3	24.45
398.63	N 1s	6.58
400.11	N 1s	3.73
404.42	N 1s	0.2
406.39	N 1s	0.11
531.28	O 1s	4.7
533.35	O 1s	7.51
536.34	O 1s	0.27
538.6	O 1s	0.18
167.58	S 2p	0.14

XPS carbon deconvolution

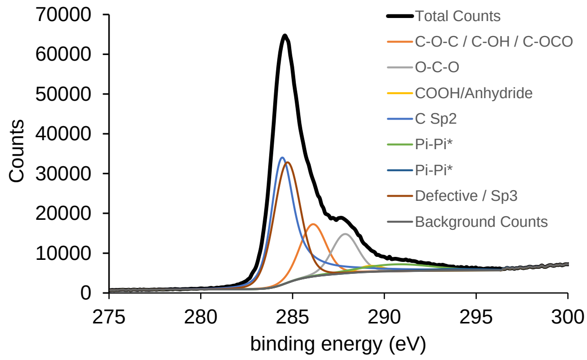

XPS oxygen deconvolution

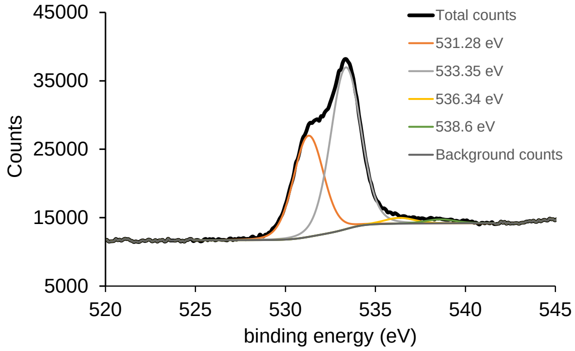

XPS nitrogen deconvolution

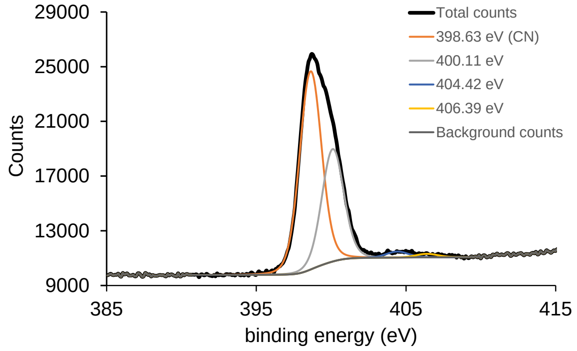

Combustion and XPS analysis of $SN_{Nic}300_{Th}$

Combustion analysis: C, 73.1%; H, 2.2%; N, 1.1%.

XPS analysis: C, 83.7%; N, 1.5%; O, 14.7%; S, 0.1%.

XPS Energies

Energy (eV)	Assignment	Atomic Concentration (%)
286.15	C-O-C / C-OH / C-OCO	13.33
287.7	O-C-O	4.19
289.16	COOH/Anhydride	2
284.42	C sp^2	28.59
290.78	π-π*	3.93
293.87	π-π*	0.89
284.75	Defective / sp^3	30.79
399.07	N 1s	0.95
400.48	N 1s	0.41
402.67	N 1s	0.12
531.45	O 1s	4.85
533.42	O 1s	8.79
535.97	O 1s	0.76
538.4	O 1s	0.28
167.48	S 2p	0.13

XPS carbon deconvolution

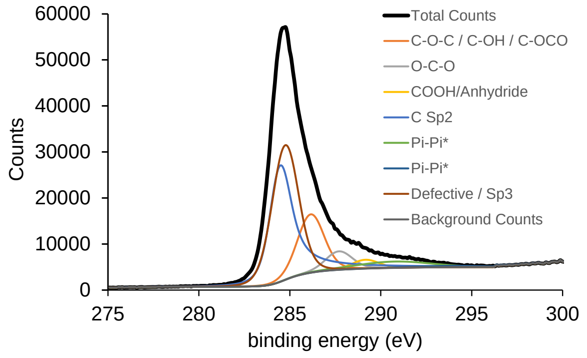

XPS oxygen deconvolution

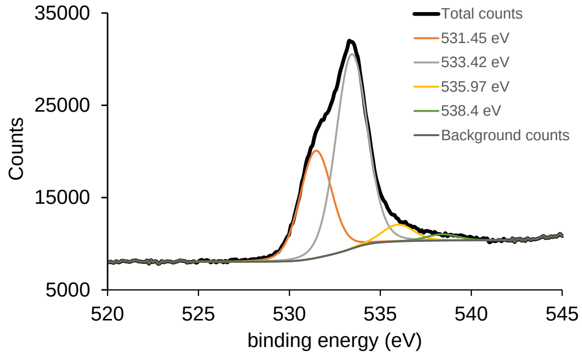

XPS nitrogen deconvolution

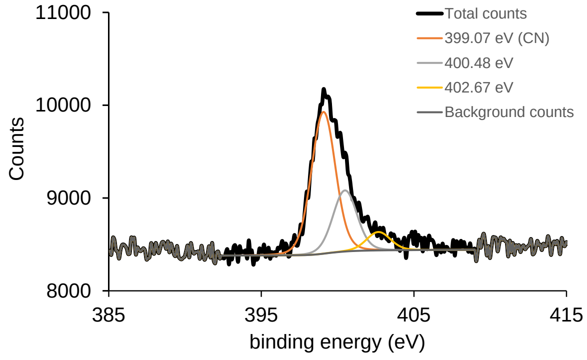

Combustion and XPS analysis of $SN_{Gly}800_{Th}$

Combustion analysis: C, 87.6%; H, 0.6%; N, 1.7%.

XPS analysis: C, 95.8%; N, 2.0%; O, 2.2%.

XPS Energies

Energy (eV)	Assignment	Atomic Concentration (%)
286.54	C-O-C / C-OH / C-OCO	5.45
288.1	O-C-O	2.29
289.1	COOH/Anhydride	1.41
284.38	C sp^2	62.75
290.74	π-π*	8.62
293.83	π-π*	1.96
284.7	Defective / sp^3	13.33
398.14	N 1s	0.65
400.63	N 1s	1.03
405.75	N 1s	0.1
403.16	N 1s	0.2
530.41	O 1s	0.56
533.22	O 1s	0.93
535.71	O 1s	0.17
538.01	O 1s	0.09
531.74	O 1s	0.46

XPS carbon deconvolution

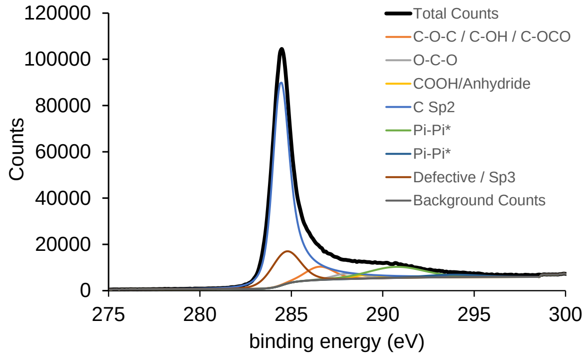

XPS oxygen deconvolution

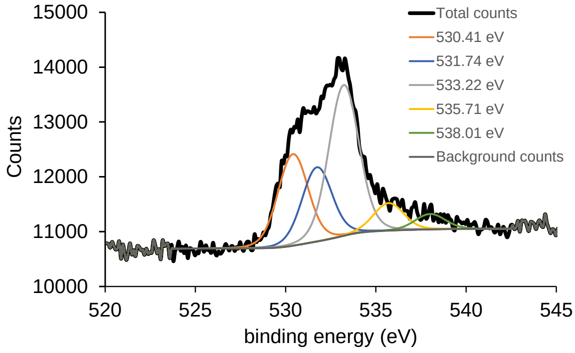

XPS nitrogen deconvolution

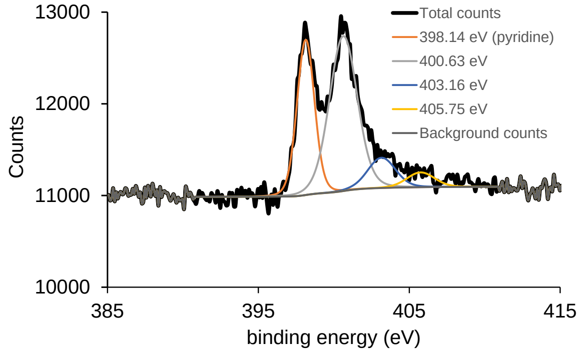

Combustion and XPS analysis of $SN_{Bal}800_{Th}$

Combustion analysis: C, 89.5%; H, 0.0%; N, 2.9%.

XPS analysis: C, 95.3%; N, 2.7%; O, 2.0%.

XPS Energies

Energy (eV)	Assignment	Atomic Concentration (%)
286.52	C-O-C / C-OH / C-OCO	5.55
288.1	O-C-O	2.71
289.38	COOH/Anhydride	1.35
284.4	C sp^2	57.41
290.76	π-π*	7.89
293.85	π-π*	1.8
284.7	Defective / sp^3	18.6
398.15	N 1s	0.86
400.67	N 1s	1.38
405.84	N 1s	0.18
403.18	N 1s	0.29
530.56	O 1s	0.68
533.35	O 1s	0.71
536.35	O 1s	0.15
539.39	O 1s	0.08
532.4	O 1s	0.35

XPS carbon deconvolution

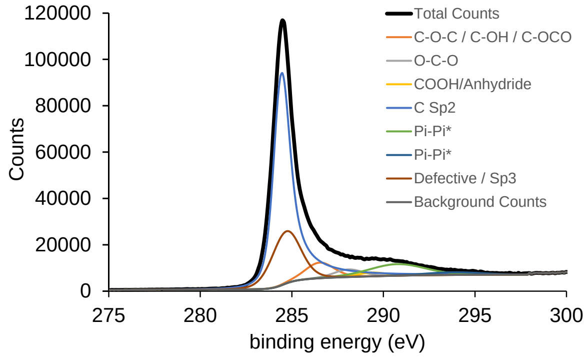

XPS oxygen deconvolution

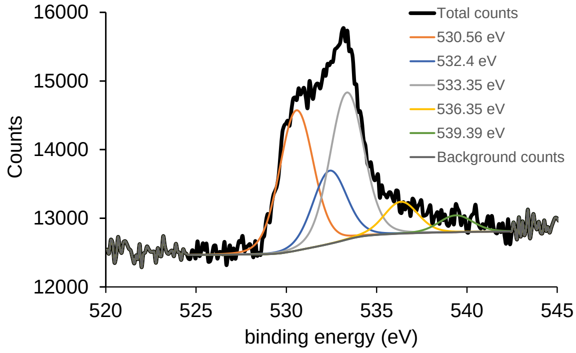

XPS nitrogen deconvolution

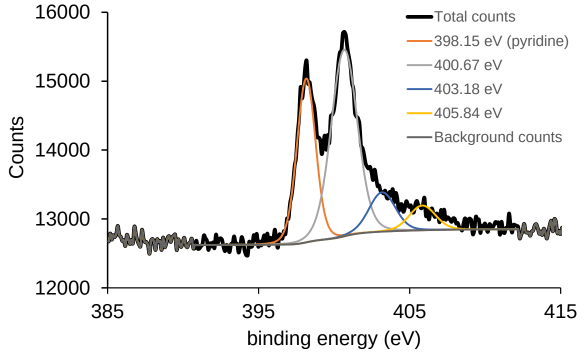

Combustion and XPS analysis of $SN_{Ure}800_{Th}$

Combustion analysis: C, 83.9%; H, 0.2%; N, 3.0%.

XPS analysis: C, 95.0%; N, 3.0%; O, 1.9%.

XPS Energies

Energy (eV)	Assignment	Atomic Concentration (%)
286.44	C-O-C / C-OH / C-OCO	5.57
288.1	O-C-O	2.88
289.38	COOH/Anhydride	1.22
284.37	C sp^2	60.29
290.73	π-π*	8.28
293.82	π-π*	1.89
284.7	Defective / sp^3	14.9
398.1	N 1s	1.02
400.42	N 1s	1.56
406	N 1s	0.15
403.44	N 1s	0.3
530.4	O 1s	0.6
533.27	O 1s	0.72
535.91	O 1s	0.14
538	O 1s	0.08
531.85	O 1s	0.38

XPS carbon deconvolution

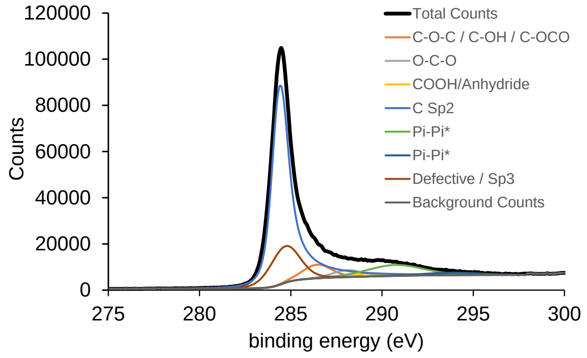

XPS oxygen deconvolution

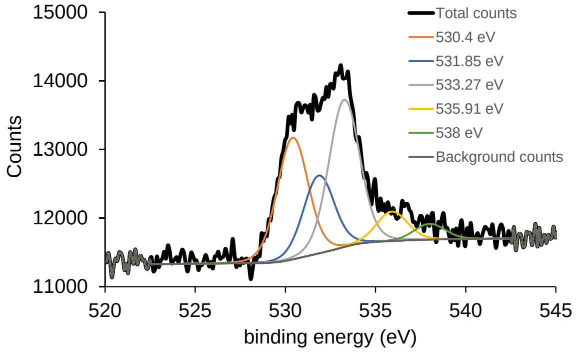

XPS nitrogen deconvolution

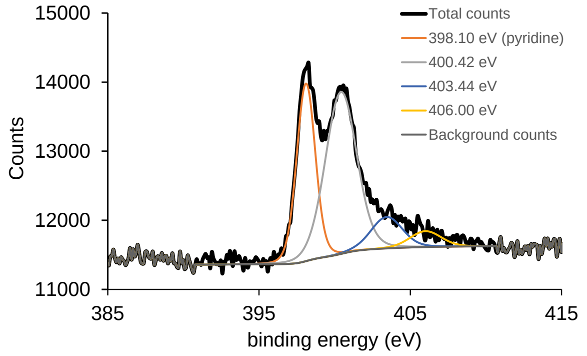

Combustion and XPS analysis of $SN_{Mel}800_{Th}$

Combustion analysis: C, 80.8%; H, 0.3%; N, 7.9%.

XPS analysis: C, 89.7%; N, 8.1%; O, 2.1%.

<u>XPS Energies</u>

Energy (eV)	Assignment	Atomic Concentration (%)
286.26	C-O-C / C-OH / C-OCO	8.53
287.9	O-C-O	3.75
289.6	COOH/Anhydride	2.04
284.5	C sp^2	46.45
290.86	π-π*	6.38
293.95	π-π*	1.45
284.86	Defective / sp^3	21.13
398.16	N 1s	2.96
400.54	N 1s	4
406.1	N 1s	0.4
403.49	N 1s	0.77
530.45	O 1s	0.72
533.46	O 1s	0.63
535.88	O 1s	0.18
538.54	O 1s	0.09
532.14	O 1s	0.5

<u>XPS carbon deconvolution</u>

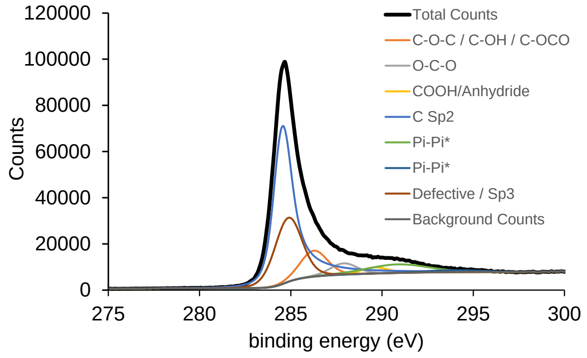

<u>XPS oxygen deconvolution</u>

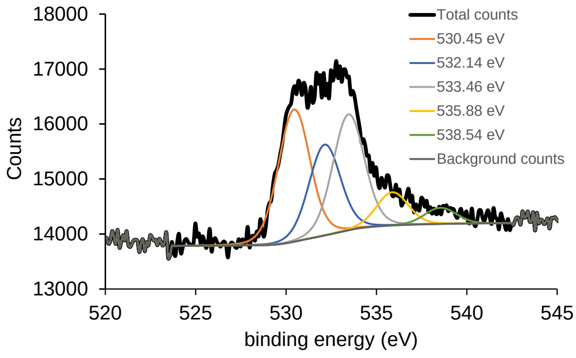

<u>XPS nitrogen deconvolution</u>

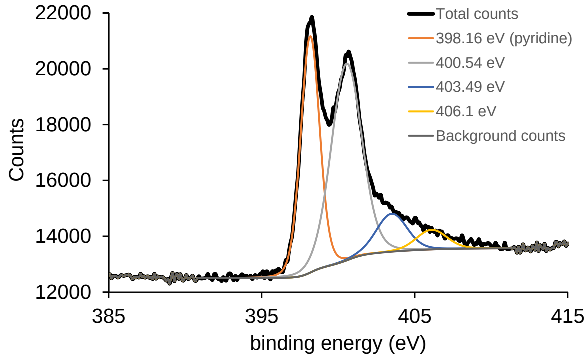

Combustion and XPS analysis of $SN_{Nic}800_{Th}$

Combustion analysis: C, 87.4%; H, 0.0%; N, 0.8%.

XPS analysis: C, 96.0%; N, 1.4%; O, 2.5%.

XPS Energies

Energy (eV)	Assignment	Atomic Concentration (%)
286.5	C-O-C / C-OH / C-OCO	5.27
288.09	O-C-O	2.59
289.2	COOH/Anhydride	1.33
284.35	C sp^2	68.33
290.7	π-π*	9.39
293.8	π-π*	2.14
284.7	Defective / sp^3	6.97
398.09	N 1s	0.4
400.58	N 1s	0.77
406.27	N 1s	0.09
403.38	N 1s	0.18
530.74	O 1s	0.98
533.12	O 1s	1.28
535.97	O 1s	0.2
538.21	O 1s	0.08

XPS carbon deconvolution

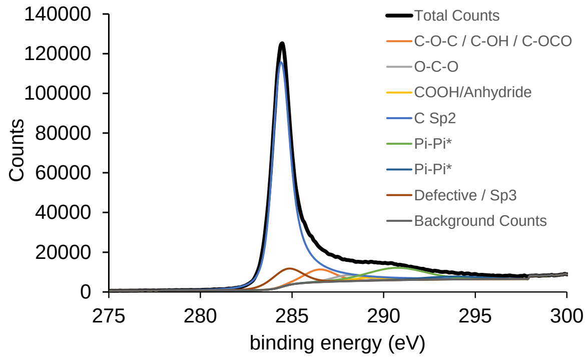

XPS oxygen deconvolution

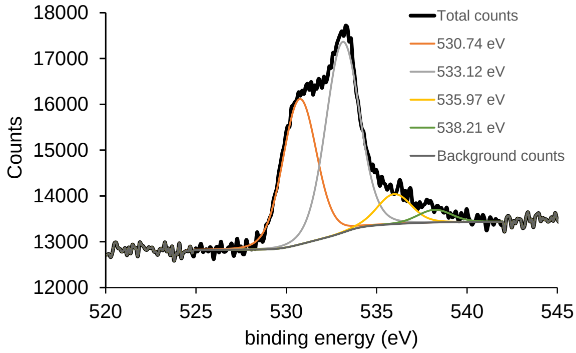

XPS nitrogen deconvolution

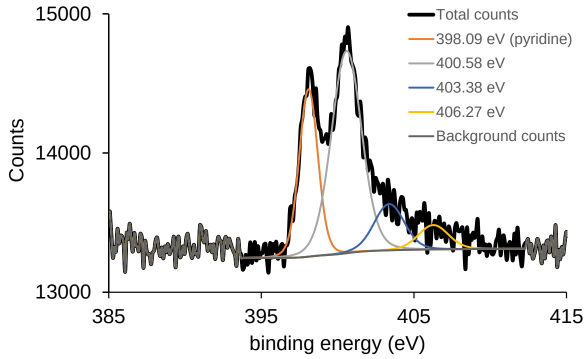

Combustion and XPS analysis of $SN_{Gly}300_{Mo}$

Combustion analysis: C, 69.2%; H, 4.1%; N, 6.5%.

XPS analysis: C, 81.0%; N, 5.4%; O, 13.5%; S, 0.1%.

<u>XPS Energies</u>

Energy (eV)	Assignment	Atomic Concentration (%)
286.05	C-O-C / C-OH / C-OCO	15.57
287.81	O-C-O	4.76
289.14	COOH/Anhydride	1.10
284.40	C sp^2	21.02
290.75	π-π*	2.89
293.84	π-π*	0.66
284.79	Defective / sp^3	34.97
398.87	N 1s	1.50
400.10	N 1s	3.93
531.16	O 1s	4.34
533.52	O 1s	5.49
536.60	O 1s	0.16
538.66	O 1s	0.15
532.27	O 1s	3.33
167.43	S 2p	0.14

<u>XPS carbon deconvolution</u>

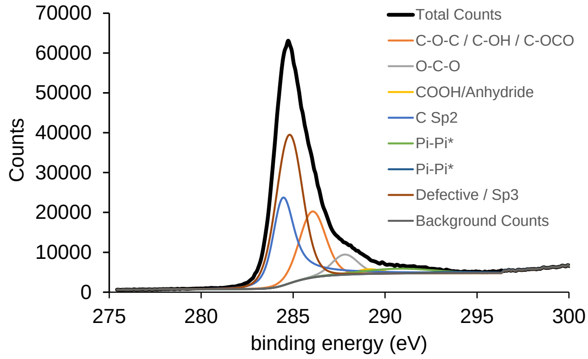

<u>XPS oxygen deconvolution</u>

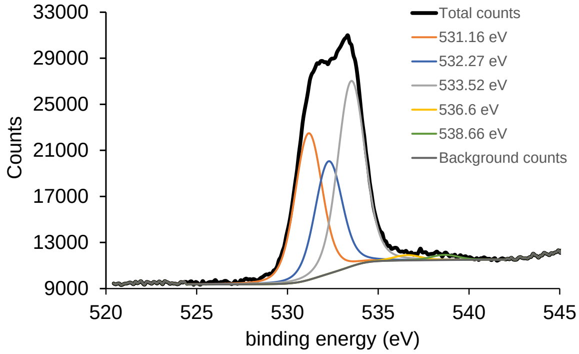

<u>XPS nitrogen deconvolution</u>

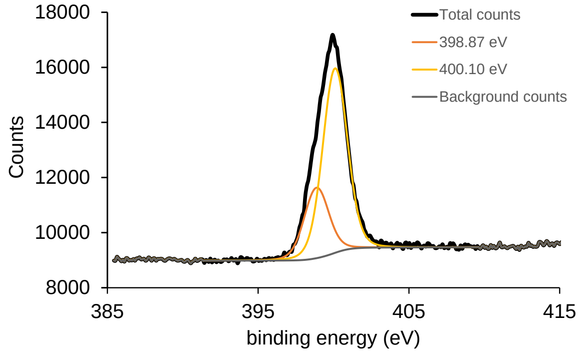

Combustion and XPS analysis of $SN_{Bal}300_{Mo}$

Combustion analysis: C, 70.1%; H, 4.2%; N, 4.6%.

XPS analysis: C, 82.3%; N, 5.0%; O, 12.5%; S, 0.2%.

XPS Energies

Energy (eV)	Assignment	Atomic Concentration (%)
284.50	C sp^2	11.58
291.01	$\pi-\pi^*$	2.73
284.75	C 1s	46.51
287.61	C 1s	3.96
286.03	C 1s	14.95
288.71	C 1s	2.60
400.00	N 1s	3.78
398.95	N 1s	1.23
531.52	O 1s	5.74
533.47	O 1s	6.51
538.44	O 1s	0.15
536.62	O 1s	0.10
167.60	S 2p	0.16

XPS carbon deconvolution

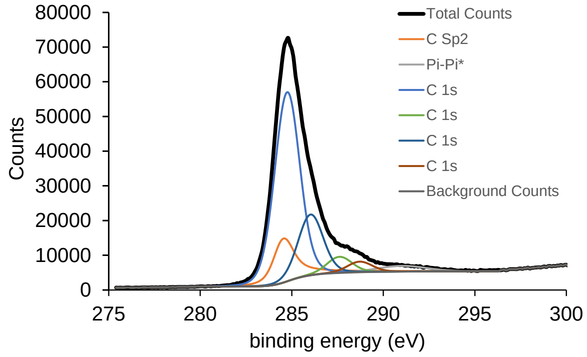

XPS oxygen deconvolution

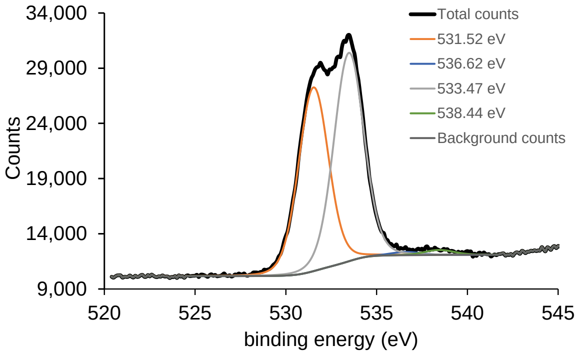

XPS nitrogen deconvolution

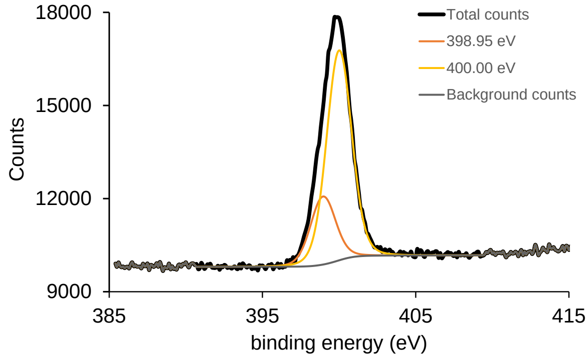

Combustion and XPS analysis of $SN_{Ure}300_{Mo}$

Combustion analysis: C, 65.1%; H, 2.1%; N, 6.9%.

XPS analysis: C, 78.1%; N, 6.9%; O, 14.8%; S, 0.2%.

XPS Energies

Energy (eV)	Assignment	Atomic Concentration (%)
286.55	C-O-C / C-OH / C-OCO	11.63
288.42	O-C-O	4.73
289.93	COOH/Anhydride	1.30
284.55	C sp^2	20.70
290.90	π-π*	2.84
294.00	π-π*	0.65
285.08	Defective / sp^3	36.24
399.02	N 1s	2.18
400.27	N 1s	4.56
406.05	N 1s (Satellite)	0.19
531.33	O 1s	4.63
533.68	O 1s	6.31
536.77	O 1s	0.19
538.82	O 1s	0.14
532.43	O 1s	3.52
167.93	S 2p	0.18

XPS carbon deconvolution

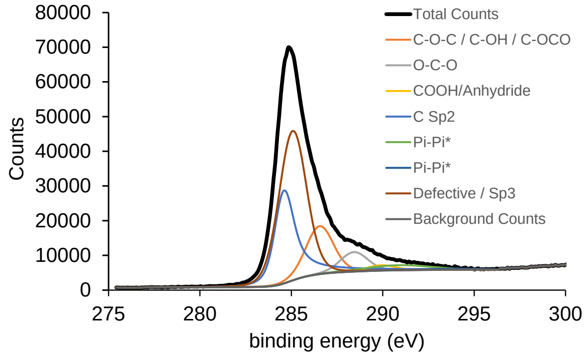

XPS oxygen deconvolution

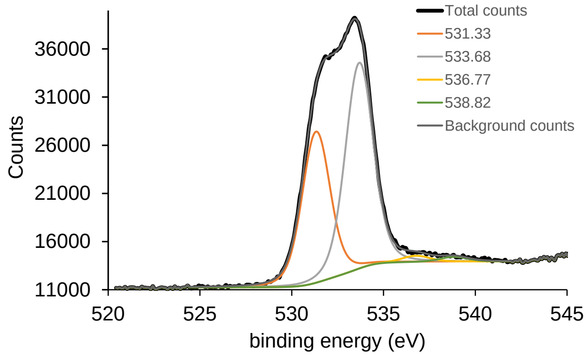

XPS nitrogen deconvolution

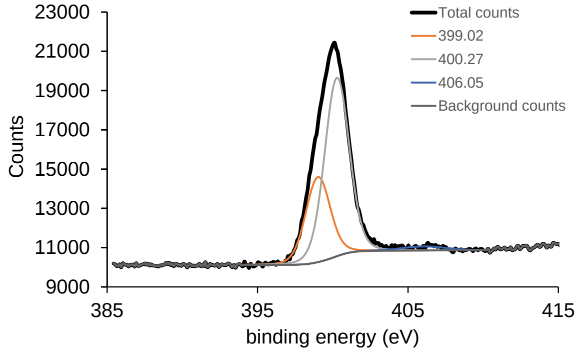

Combustion and XPS analysis of $SN_{Mel}300_{Mo}$

Combustion analysis: C, 65.4%; H, 3.4%; N, 11.3%.

XPS analysis: C, 76.8%; N, 8.5%; O, 14.6%; S, 0.1%.

XPS Energies

Energy (eV)	Assignment	Atomic Concentration (%)
286.32	C-O-C / C-OH / C-OCO	11.85
287.89	O-C-O	6.71
289.06	COOH/Anhydride	2.33
284.48	C sp^2	20.17
290.84	π-π*	2.77
293.93	π-π*	0.63
285.00	Defective / sp^3	32.32
398.86	N 1s	4.89
400.17	N 1s	3.25
406.16	N 1s (Satellite)	0.39
531.37	O 1s	3.39
533.72	O 1s	7.19
536.81	O 1s	0.19
538.86	O 1s	0.13
532.47	O 1s	3.67
168.03	S 2p	0.12

XPS carbon deconvolution

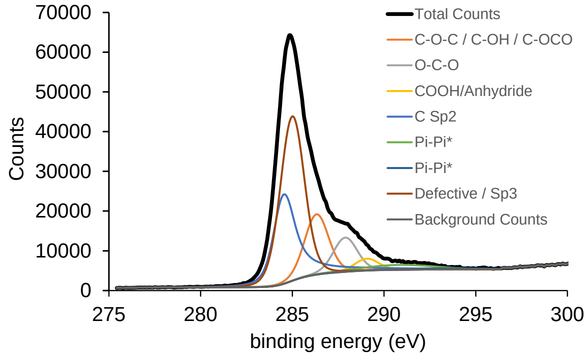

XPS oxygen deconvolution

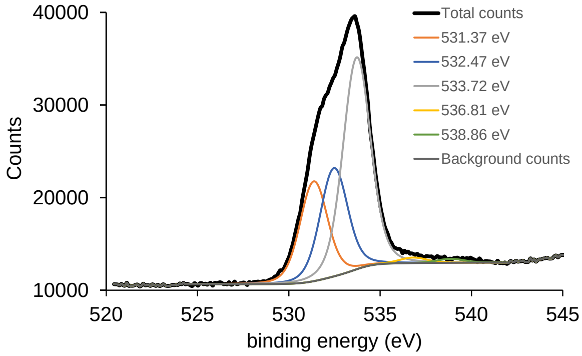

XPS nitrogen deconvolution

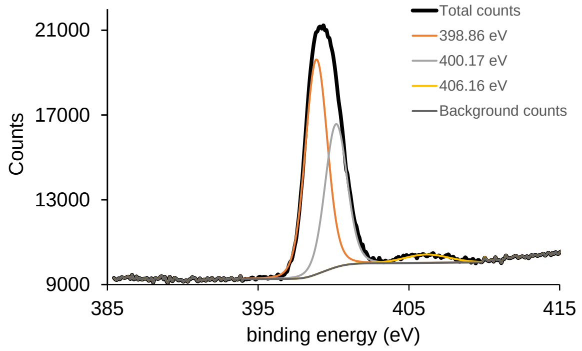

Combustion and XPS analysis of $SN_{Nic}300_{Mo}$

Combustion analysis: C, 74.0%; H, 3.1%; N, 2.9%.

XPS analysis: C, 83.9%; N, 3.8%; O, 12.3%; S, 0.1%.

XPS Energies

Energy (eV)	Assignment	Atomic Concentration (%)
284.5	C sp^2	13.99
291.5	π-π*	3.32
284.8	C 1s	44.49
287.74	C 1s	4.22
286.11	C 1s	15.7
289.23	C 1s	2.16
399.15	N 1s	2.36
400.41	N 1s	1.16
405.57	N 1s	0.14
402.62	N 1s	0.14
531.55	O 1s	4.08
533.58	O 1s	7.73
538.94	O 1s	0.16
536.7	O 1s	0.29
168.55	S 2p	0.07

XPS carbon deconvolution

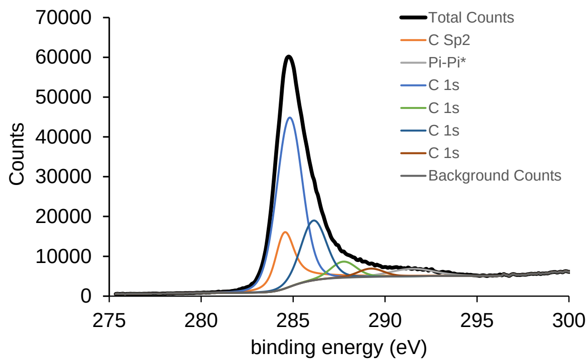

XPS oxygen deconvolution

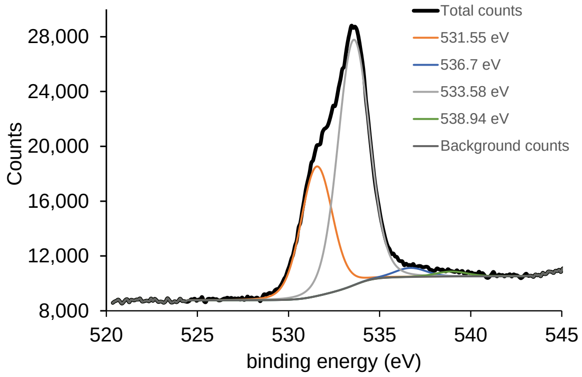

XPS nitrogen deconvolution

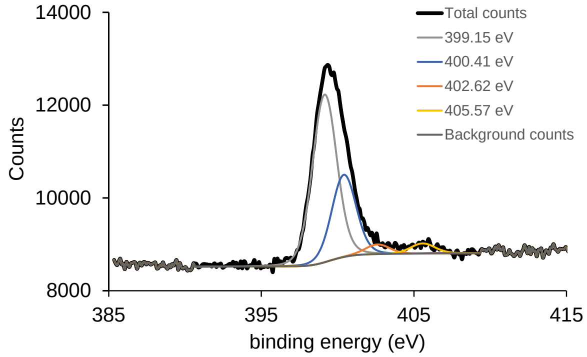

Combustion analysis of SN_X800_{Mo}

$SN_{Gly}800_{Mo}$
Combustion analysis: C, 82.1%; H, 0.4%; N, 6.1%.

$SN_{Bal}800_{Mo}$
Combustion analysis: C, 84.8%; H, 1.4%; N, 4.3%.

$SN_{Ure}800_{Mo}$
Combustion analysis: C, 79.8%; H, 0.4%; N, 9.0%.

$SN_{Mel}800_{Mo}$
Combustion analysis: C, 75.1%; H, 1.0%; N, 8.4%.

$SN_{Nic}800_{Mo}$
Combustion analysis: C, 84.3%; H, 0.6%; N, 3.9%.

Combustion and XPS analysis of $SN_{Gly}300_{Mu}$

Combustion analysis: C, 71.7%; H, 4.3%; N, 1.5%.

XPS analysis: C, 81.8%; N, 2.3%; O, 15.3%; S, 0.1%.

XPS Energies

Energy (eV)	Assignment	Atomic Concentration (%)
286.35	C-O-C / C-OH / C-OCO	13.06
287.96	O-C-O	3.52
289.44	COOH/Anhydride	0.89
284.51	C sp^2	22.15
290.87	$\pi-\pi^*$	3.04
293.96	$\pi-\pi^*$	0.69
284.95	Defective / sp^3	38.43
399.95	N 1s	1.9
402.14	N 1s	0.17
406.45	N 1s (Satellite)	0.18
531.18	O 1s	2.87
533.53	O 1s	7.57
536.62	O 1s	0.1
538.68	O 1s	0.09
532.28	O 1s	4.67
167.53	S 2p	0.11

XPS carbon deconvolution

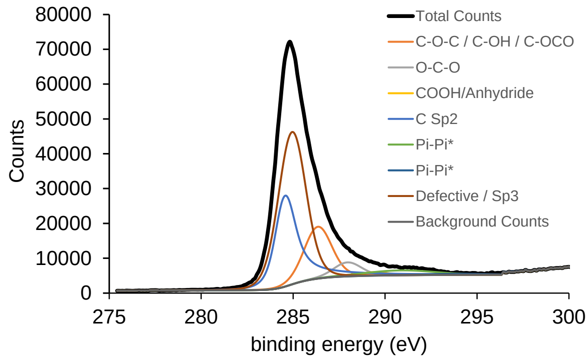

XPS oxygen deconvolution

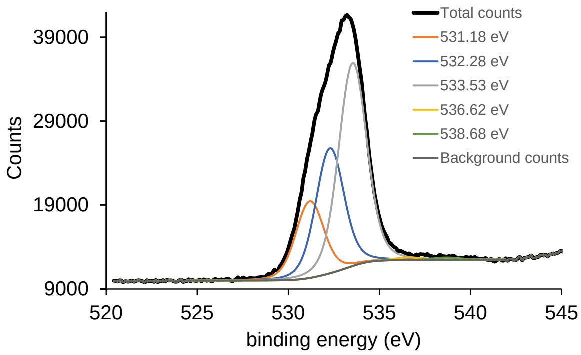

XPS nitrogen deconvolution

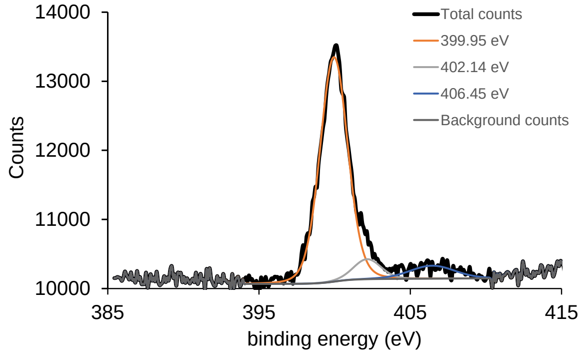

Combustion and XPS analysis of $SN_{Bal}300_{Mu}$

Combustion analysis: C, 72.0%; H, 4.1%; N, 1.3%.

XPS analysis: C, 82.0%; N, 1.9%; O, 16.1%.

XPS Energies

Energy (eV)	Assignment	Atomic Concentration (%)
284.5	C sp^2	14.51
291.08	π-π*	2.37
284.78	C 1s	44.43
287.52	C 1s	4.29
286.11	C 1s	13.4
289.02	C 1s	3.02
400.03	N 1s	1.85
531.66	O 1s	6.1
533.44	O 1s	9.6
536.58	O 1s	0.27
538.97	O 1s	0.16

XPS carbon deconvolution

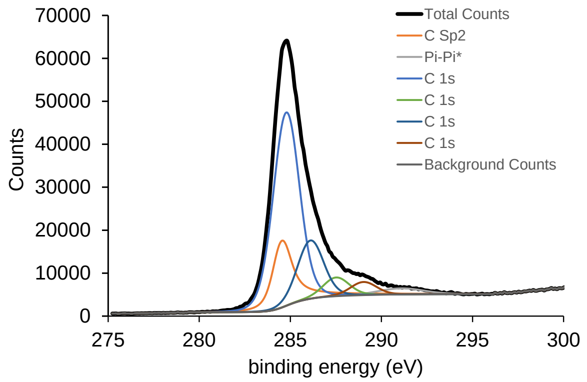

XPS oxygen deconvolution

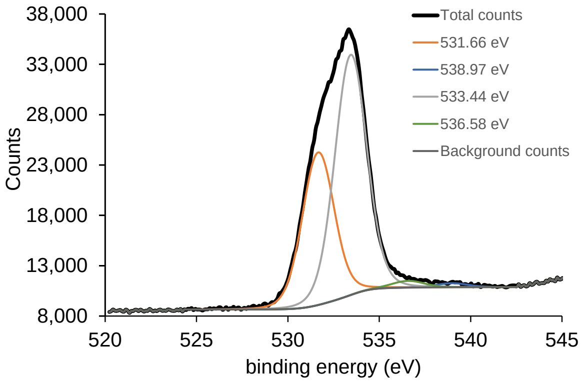

XPS nitrogen deconvolution

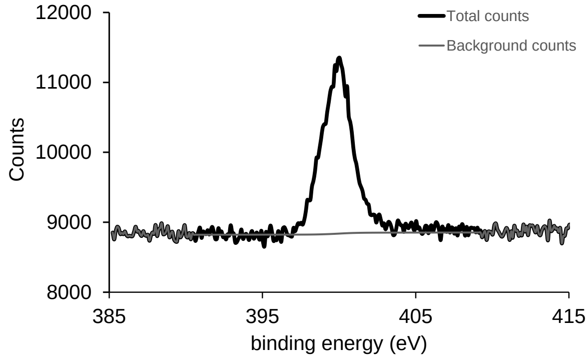

Combustion and XPS analysis of $SN_{Ure}300_{Mu}$

Combustion analysis: C, 71.0%; H, 3.8%; N, 2.3%.

XPS analysis: C, 81.7%; N, 1.6%; O, 16.7%.

XPS Energies

Energy (eV)	Assignment	Atomic Concentration (%)
284.5	C sp^2	9.44
291.02	π-π*	2.76
284.83	C 1s	48.47
287.71	C 1s	4.71
286.24	C 1s	14.17
289.12	C 1s	2.15
400.24	N 1s	0.99
402.18	N 1s	0.1
399.13	N 1s	0.5
531.74	O 1s	5.99
533.55	O 1s	10.22
538.88	O 1s	0.16
536.56	O 1s	0.35

XPS carbon deconvolution

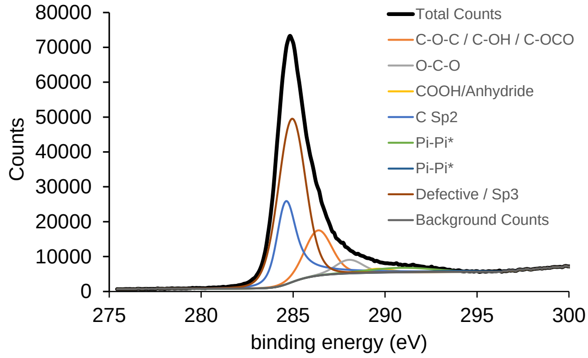

XPS oxygen deconvolution

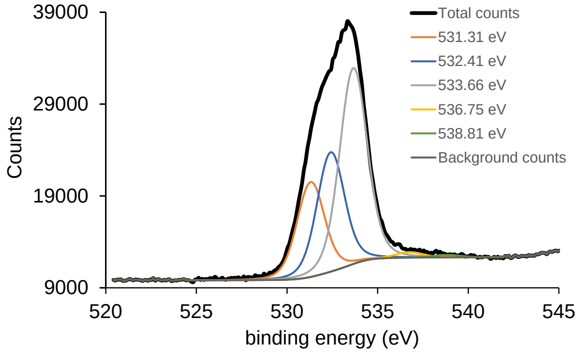

XPS nitrogen deconvolution

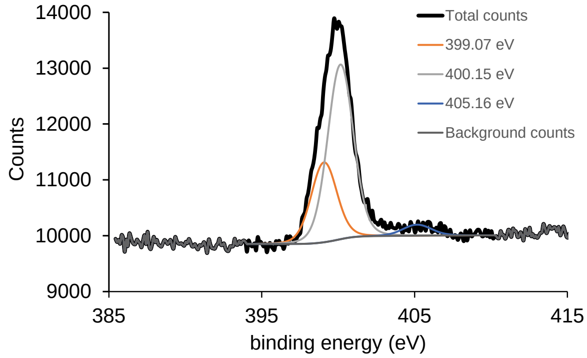

Combustion and XPS analysis of $SN_{Mel}300_{Mu}$

Combustion analysis: C, 62.2%; H, 3.0%; N, 9.5%.

XPS analysis: C, 76.2%; N, 8.7%; O, 15.2%.

XPS Energies

Energy (eV)	Assignment	Atomic Concentration (%)
284.5	C sp^2	9.4
291.06	π-π*	2.66
284.82	C 1s	42.81
287.92	C 1s	5.32
286.24	C 1s	11.78
289.23	C 1s	4.19
398.94	N 1s	3.39
400.11	N 1s	3.92
407.54	N 1s (Satellite)	0.63
401.21	N 1s	0.72
531.7	O 1s	6.74
533.48	O 1s	8.1
539.32	O 1s	0.19
536.92	O 1s	0.15

XPS carbon deconvolution

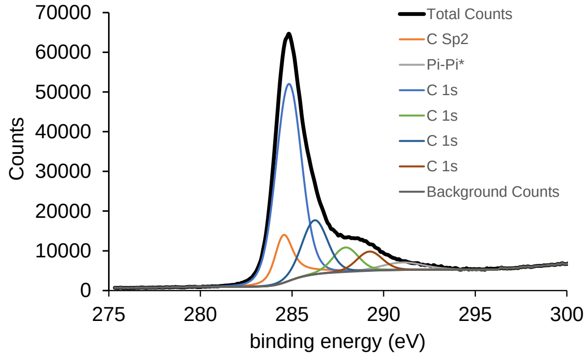

XPS oxygen deconvolution

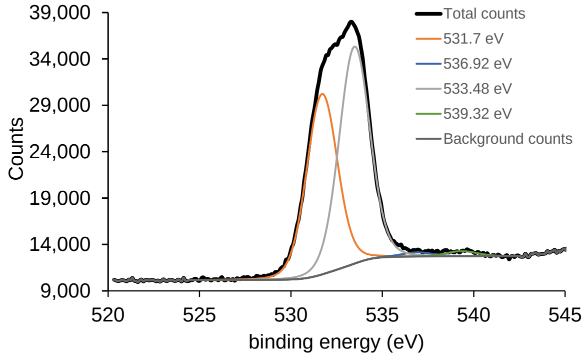

XPS nitrogen deconvolution

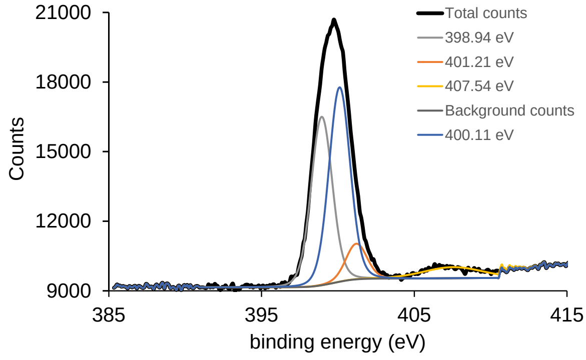

Combustion and XPS analysis of $SN_{Nic}300_{Mu}$

Combustion analysis: C, 68.9%; H, 3.5%; N, 0.2%.

XPS analysis: C, 82.9%; N, 0.7%; O, 16.4%.

XPS Energies

Energy (eV)	Assignment	Atomic Concentration (%)
284.5	C sp^2	12.41
291.28	π-π*	3.08
284.79	C 1s	44.44
287.63	C 1s	5.26
286.19	C 1s	14.94
289.13	C 1s	2.81
400.08	N 1s	0.68
531.67	O 1s	5.69
533.49	O 1s	10.15
538.43	O 1s	0.14
536.2	O 1s	0.39

XPS carbon deconvolution

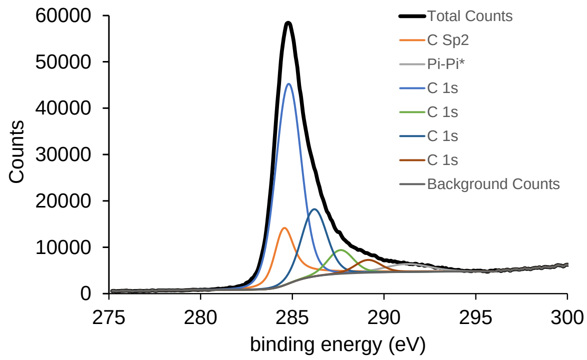

XPS oxygen deconvolution

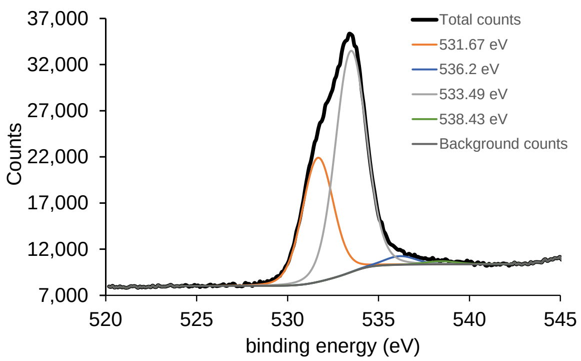

XPS nitrogen deconvolution

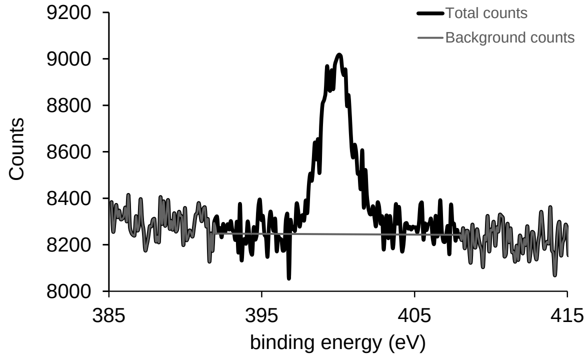

Combustion analysis of SN_X800_{Mu}

$SN_{Gly}800_{Mu}$
Combustion analysis: C, 83.5%; H, 1.0%; N, 2.7%.

$SN_{Bal}800_{Mu}$
Combustion analysis: C, 83.1%; H, 0.2%; N, 3.1%.

$SN_{Ure}800_{Mu}$
Combustion analysis: C, 83.3%; H, 0.9%; N, 5.0%.

$SN_{Mel}800_{Mu}$
Combustion analysis: C, 72.7%; H, 0.8%; N, 11.2%.

$SN_{Nic}800_{Mu}$
Combustion analysis: C, 86.1%; H, 0.0%; N, 0.0%.

Powder X-ray diffraction data on S300 and SN_X300_{Th} samples

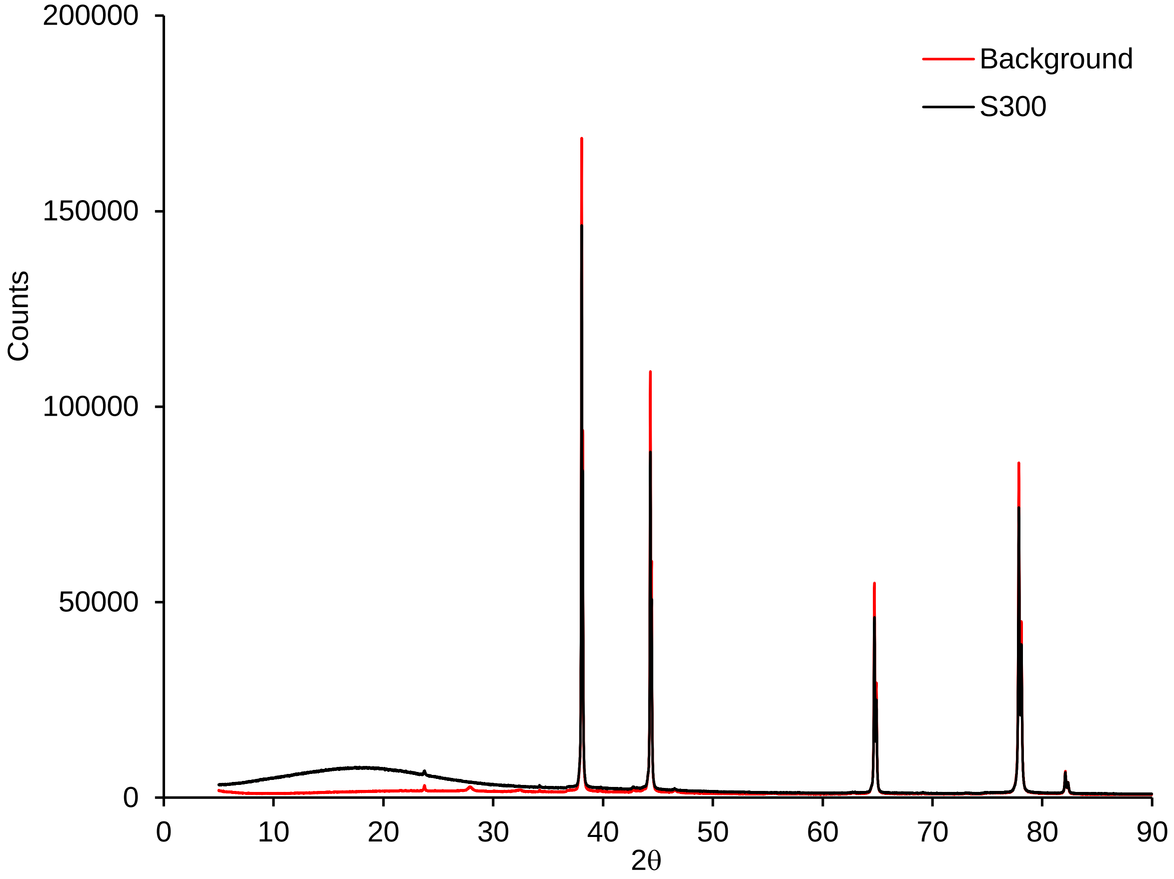

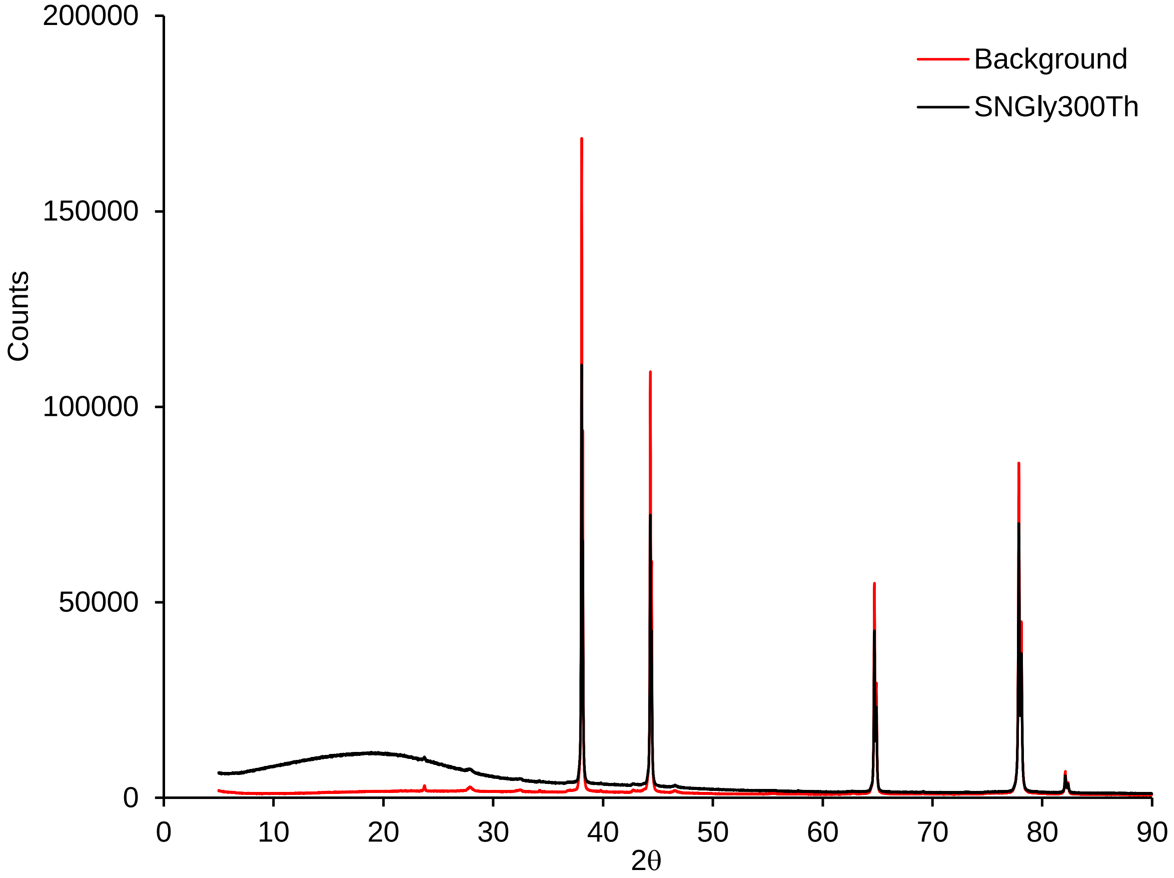

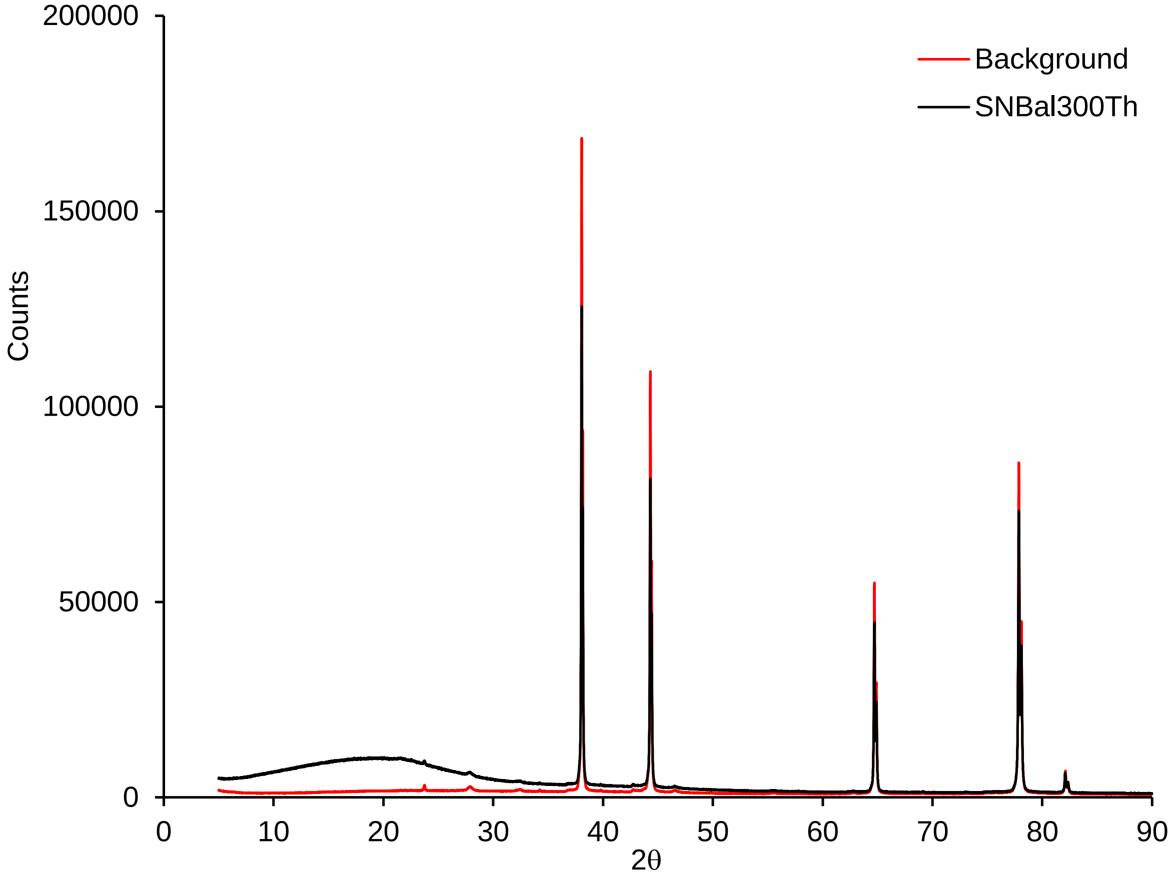

200000
150000
100000
50000
0
Counts
0
10
20
30
40
50
60
70
80
90
2θ
Background
SNBal300Th

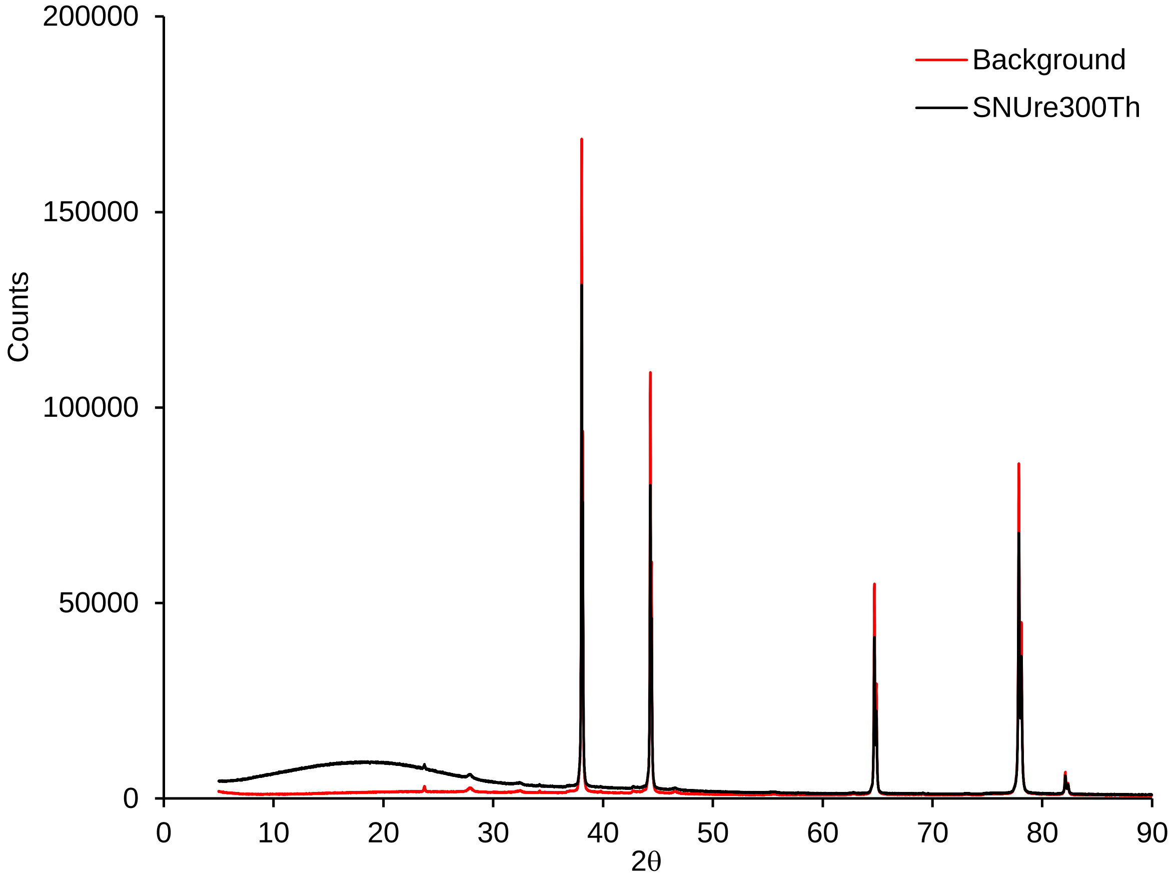

200000
150000
100000
50000
0
Counts
0
10
20
30
40
50
60
70
80
90
2θ
Background
SNUre300Th

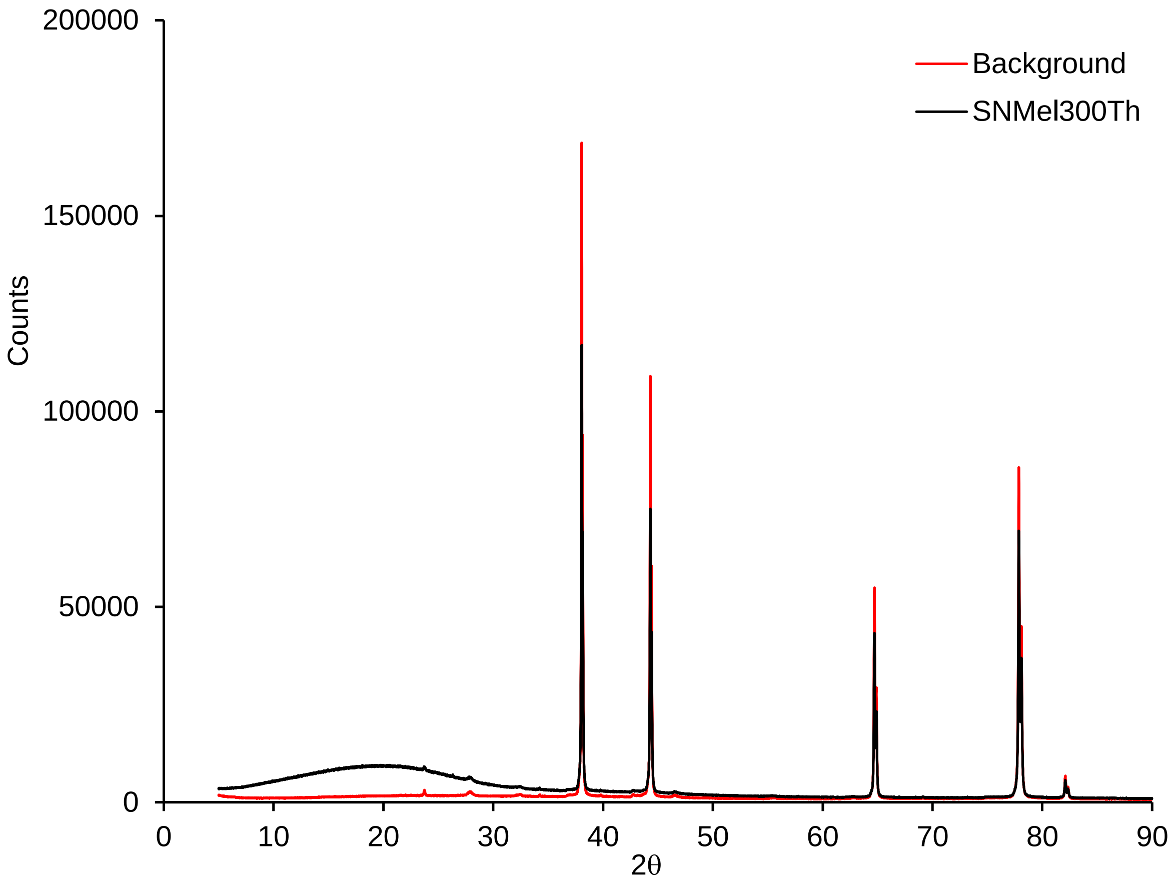
Background
SNMel300Th
Counts
2θ
200000
150000
100000
50000
0
0
10
20
30
40
50
60
70
80
90

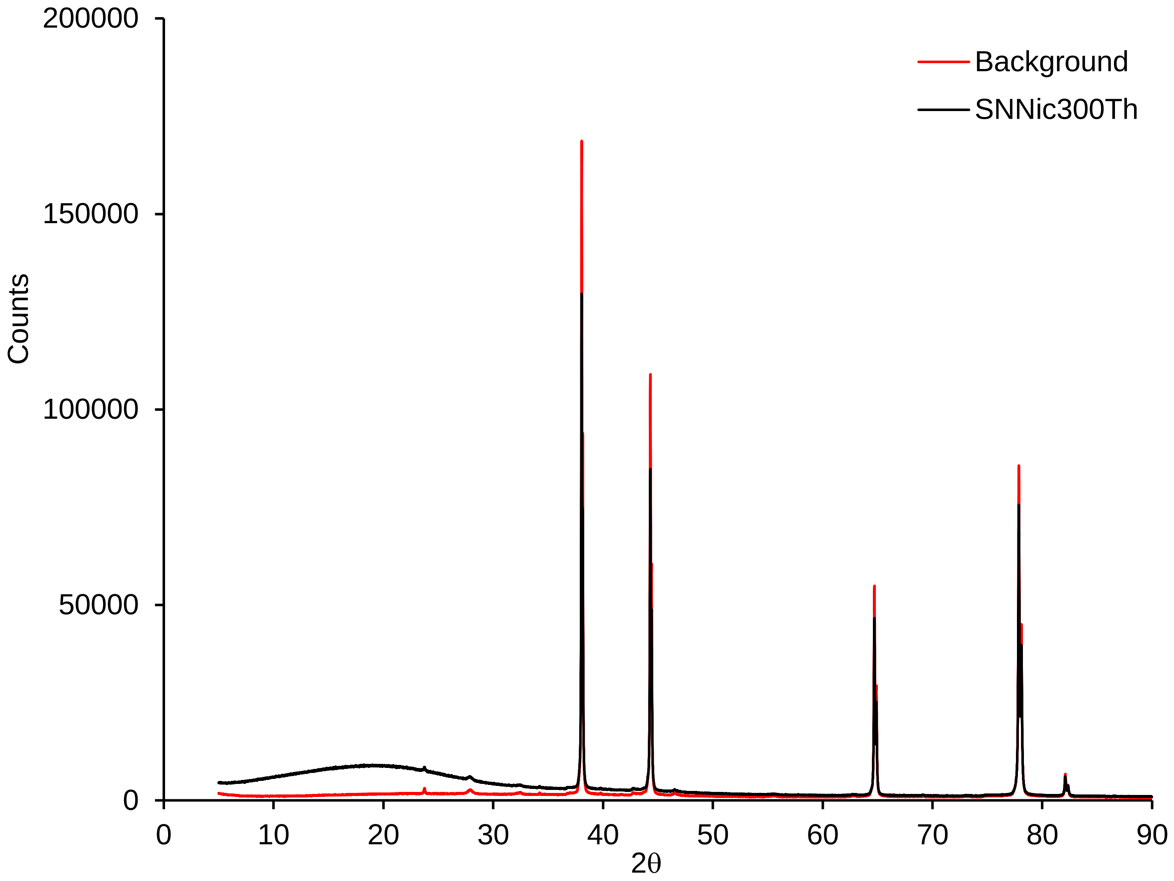
Background
SNNic300Th
Counts
2θ
200000
150000
100000
50000
0
0
10
20
30
40
50
60
70
80
90

Powder X-ray diffraction data on S800 and SN_X800_{Th} samples

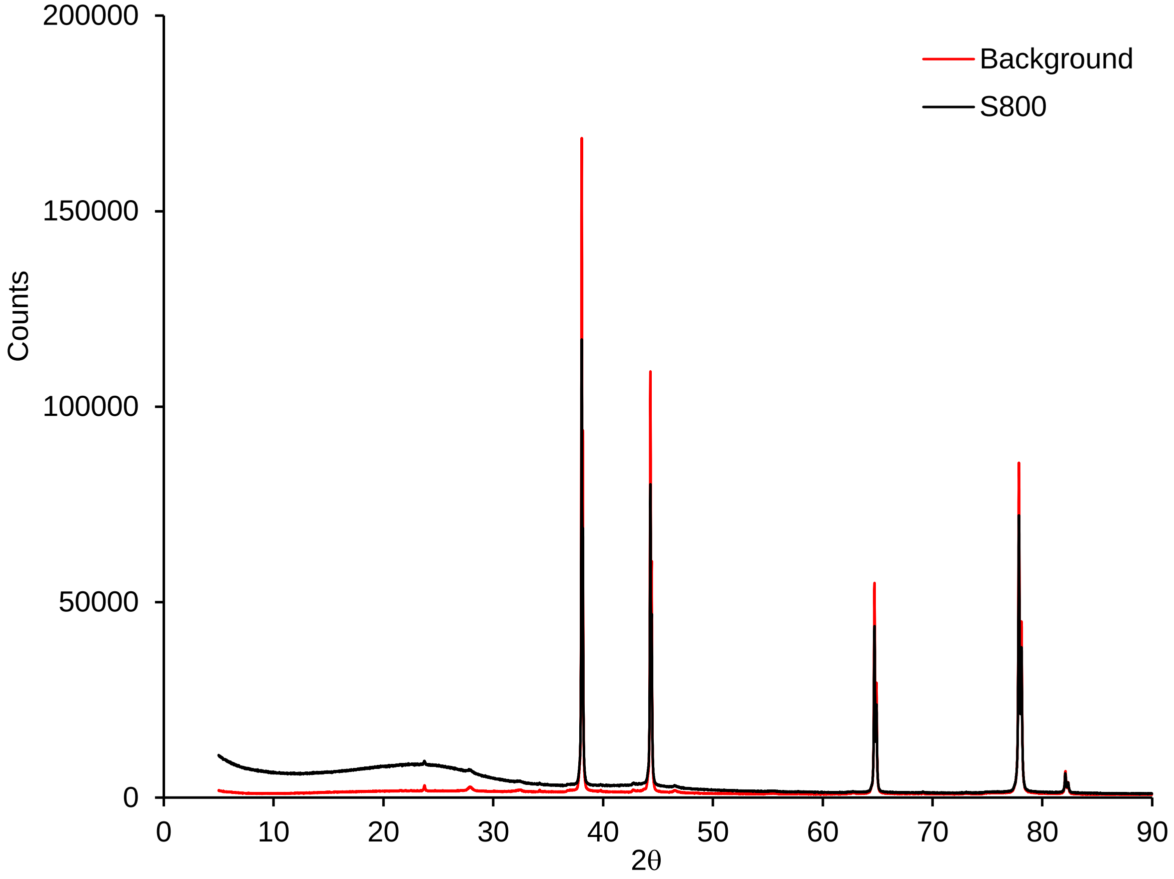

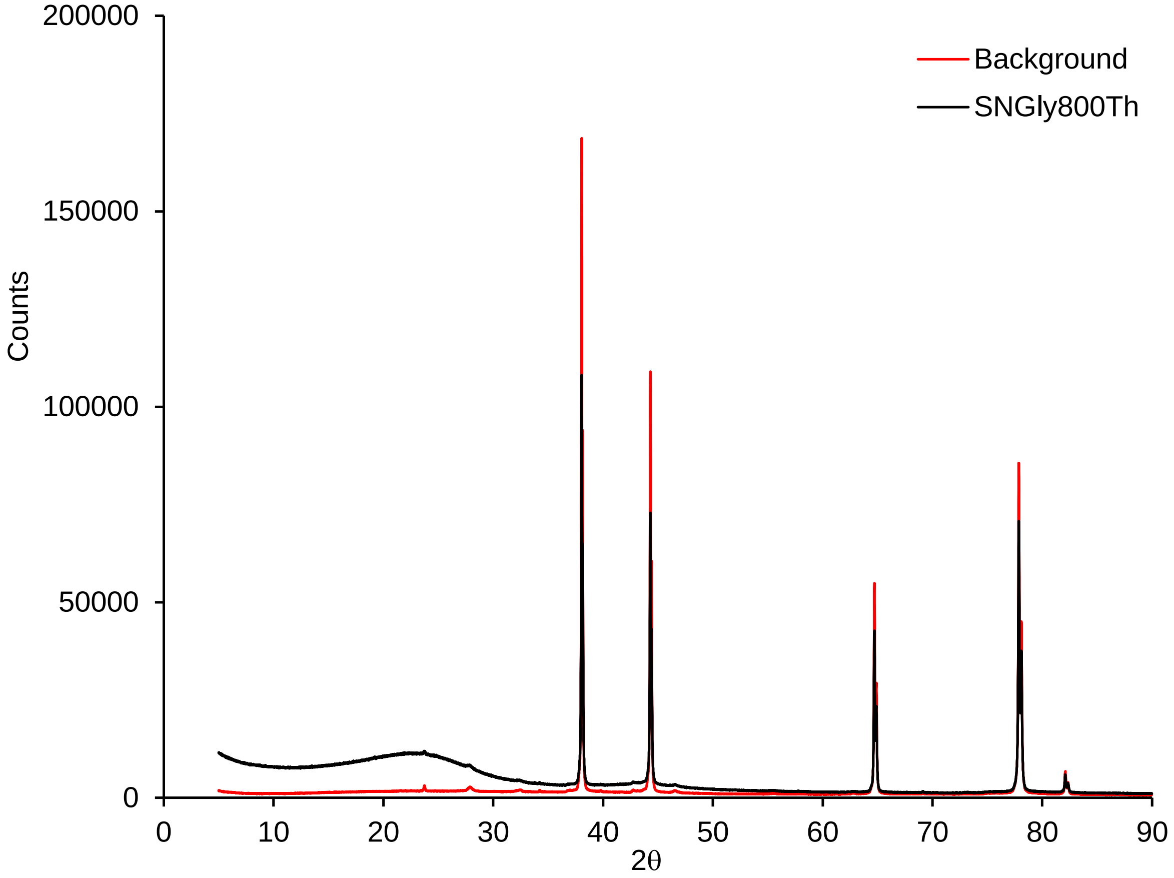

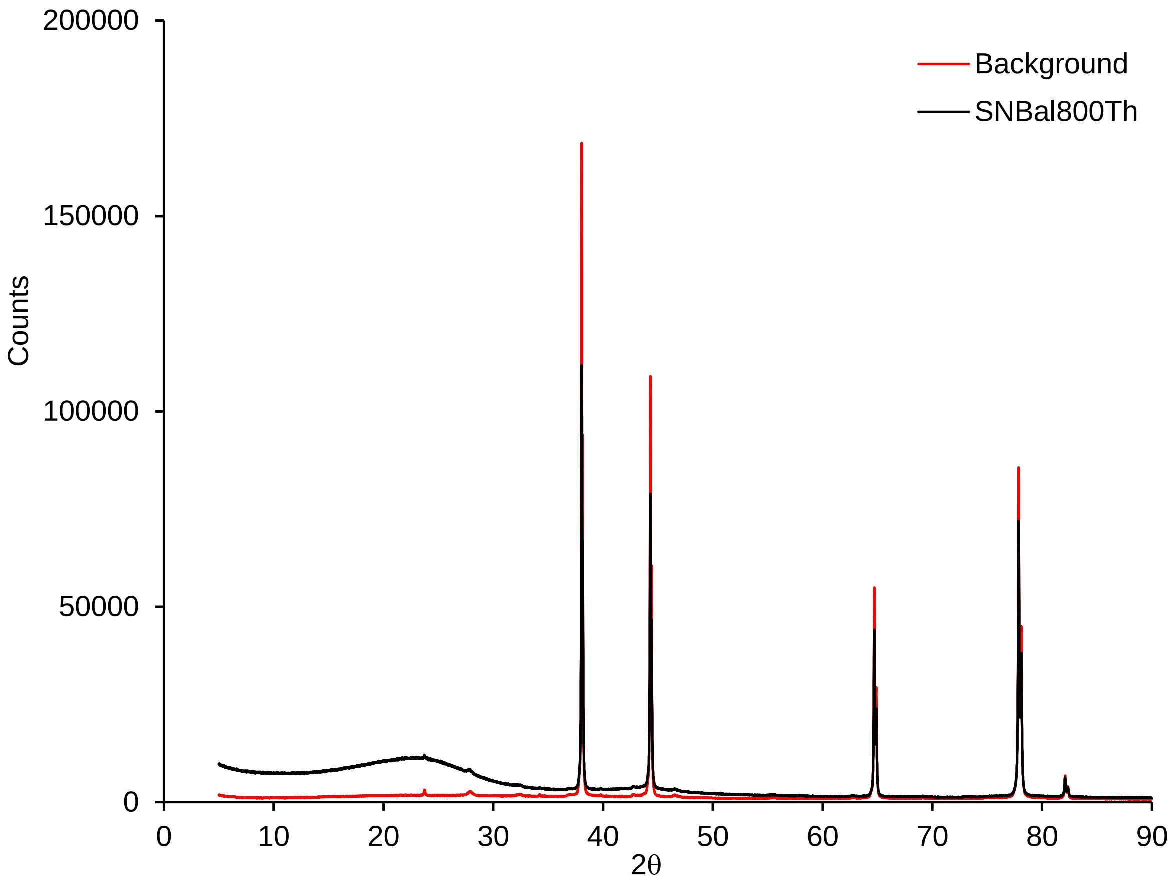

200000
150000
100000
50000
0
Counts
0
10
20
30
40
50
60
70
80
90
2θ
Background
SNBal800Th

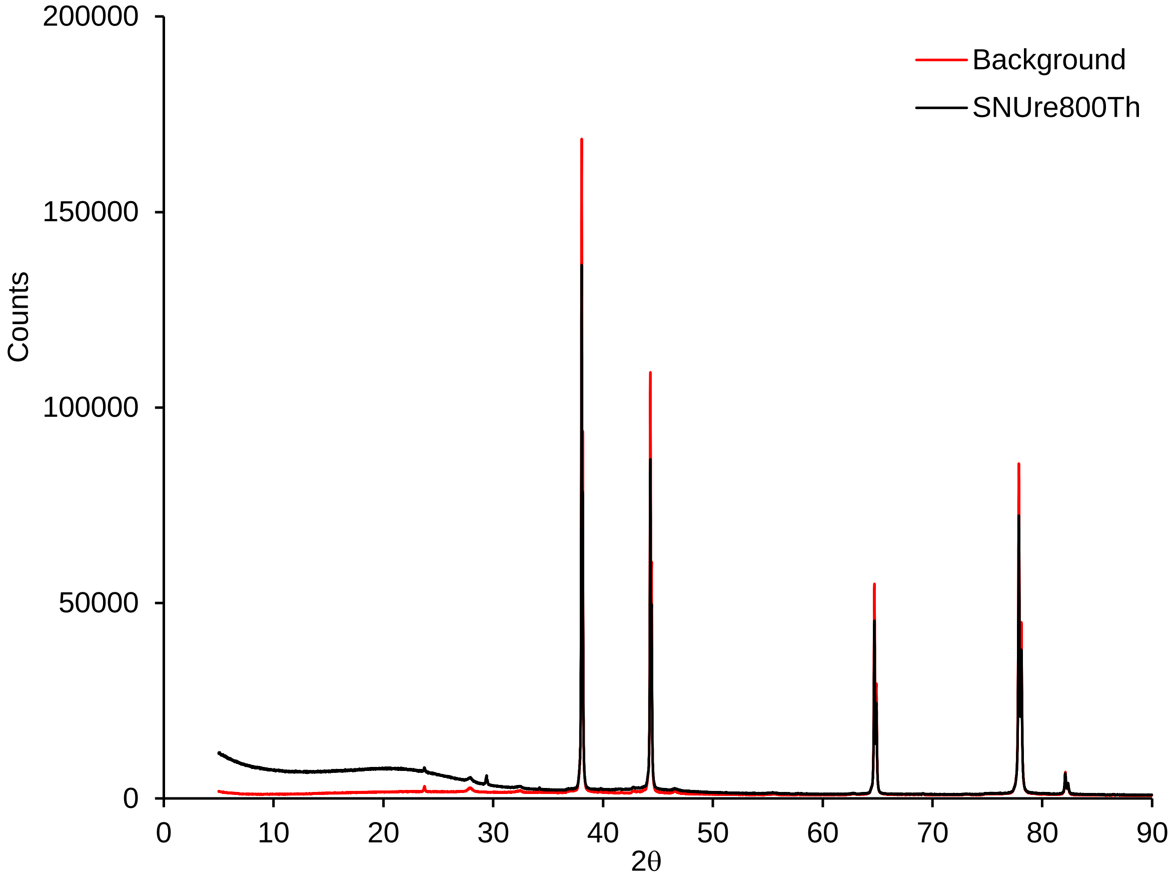

200000
150000
100000
50000
0
Counts
0
10
20
30
40
50
60
70
80
90
2θ
Background
SNUre800Th

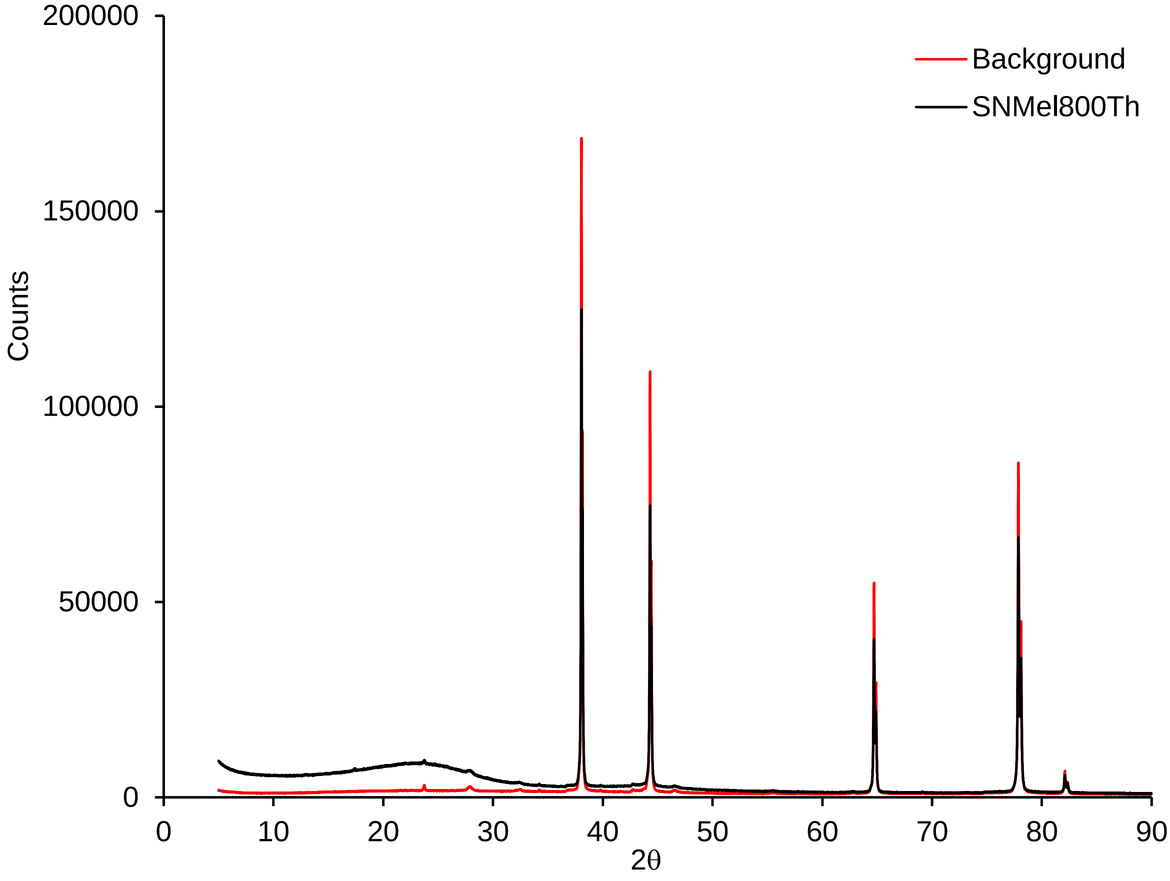

Background
SNMel800Th
Counts
200000
150000
100000
50000
0
0
10
20
30
40
50
60
70
80
90
2θ

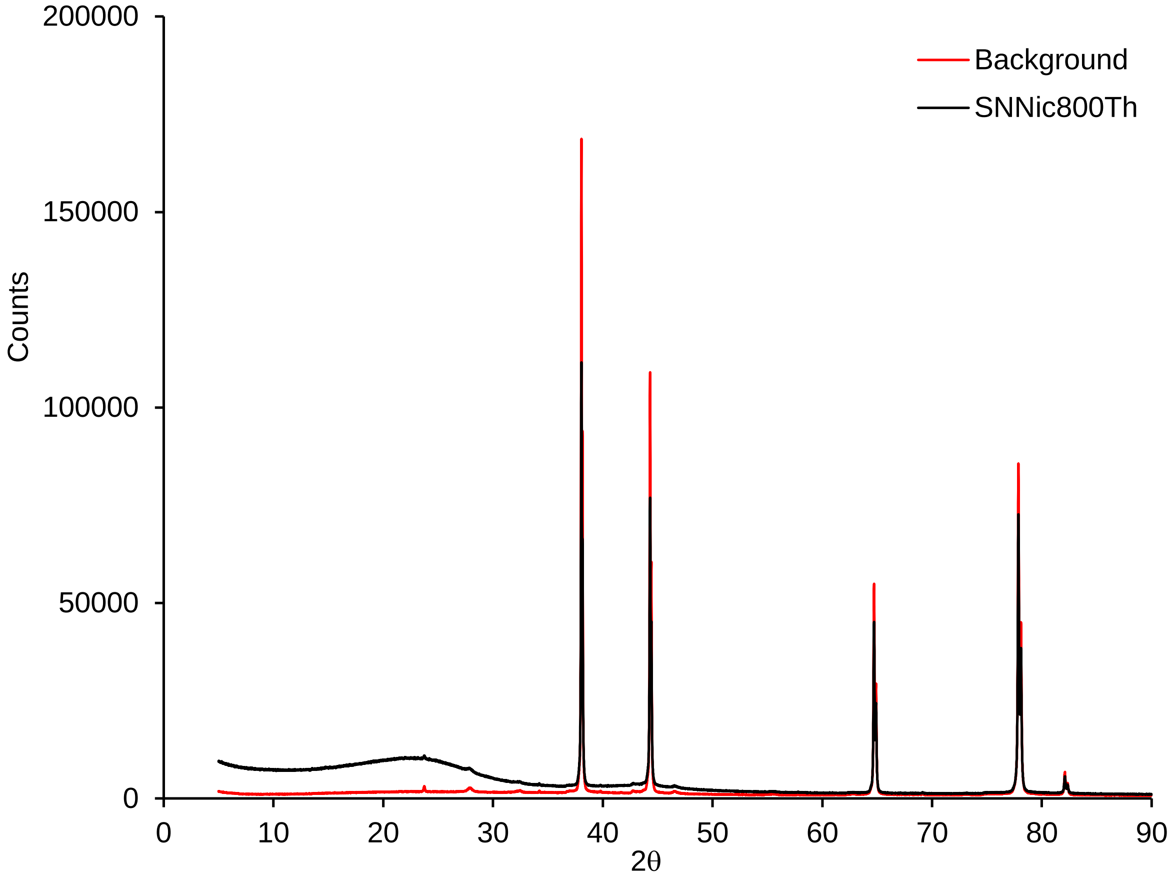

Background
SNNic800Th
Counts
200000
150000
100000
50000
0
0
10
20
30
40
50
60
70
80
90
2θ

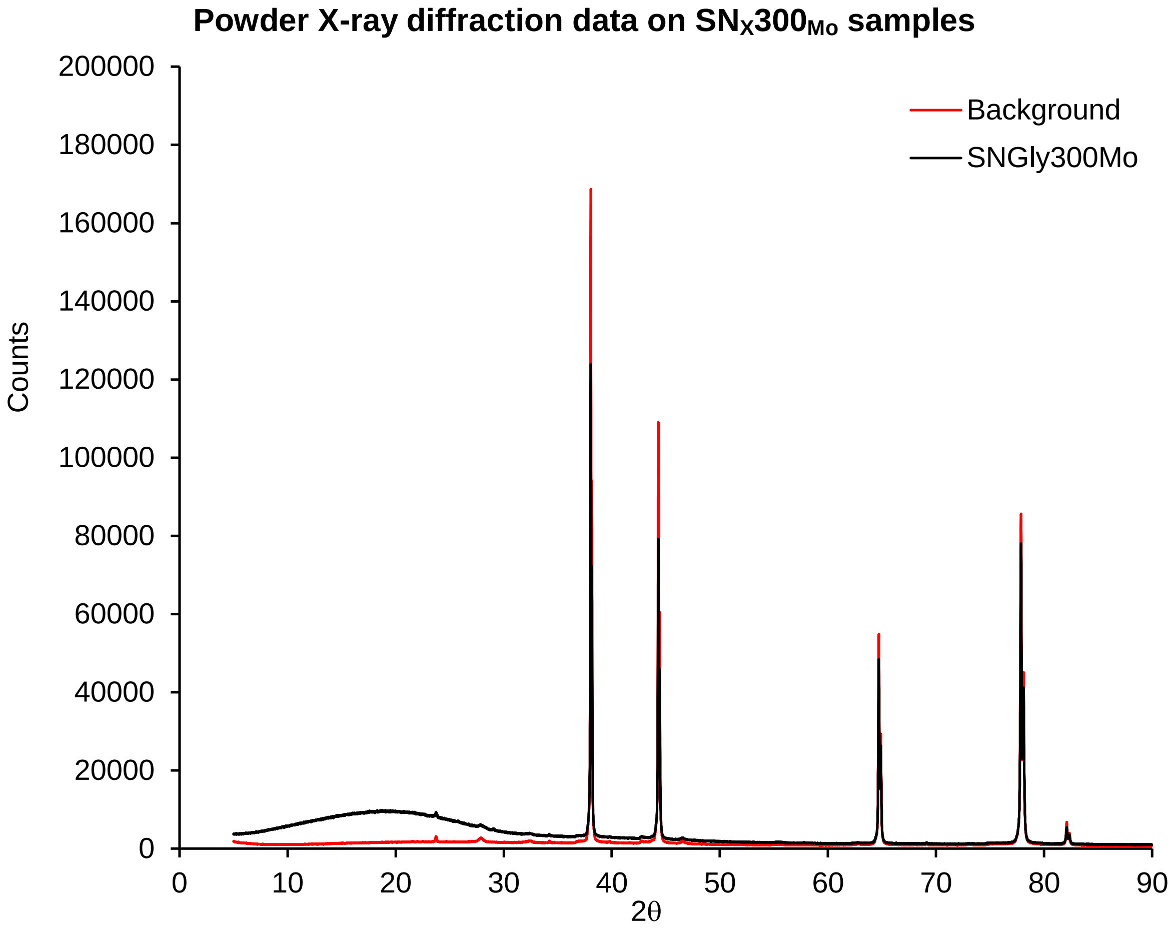
Powder X-ray diffraction data on SN_X300_{Mo} samples
Counts
2θ
Background
SNGly300Mo
0
20000
40000
60000
80000
100000
120000
140000
160000
180000
200000
0
10
20
30
40
50
60
70
80
90

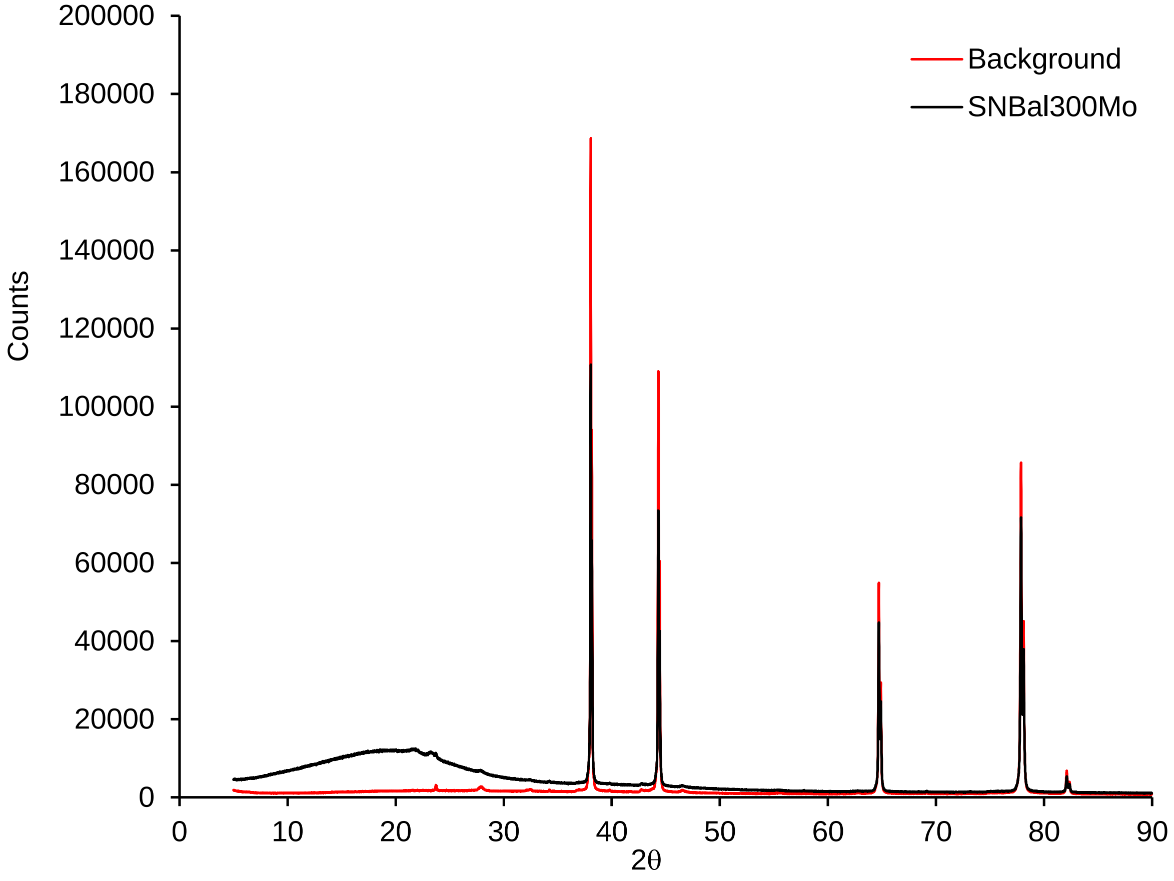
Background
SNBal300Mo
Counts
2θ
200000
180000
160000
140000
120000
100000
80000
60000
40000
20000
0
0
10
20
30
40
50
60
70
80
90

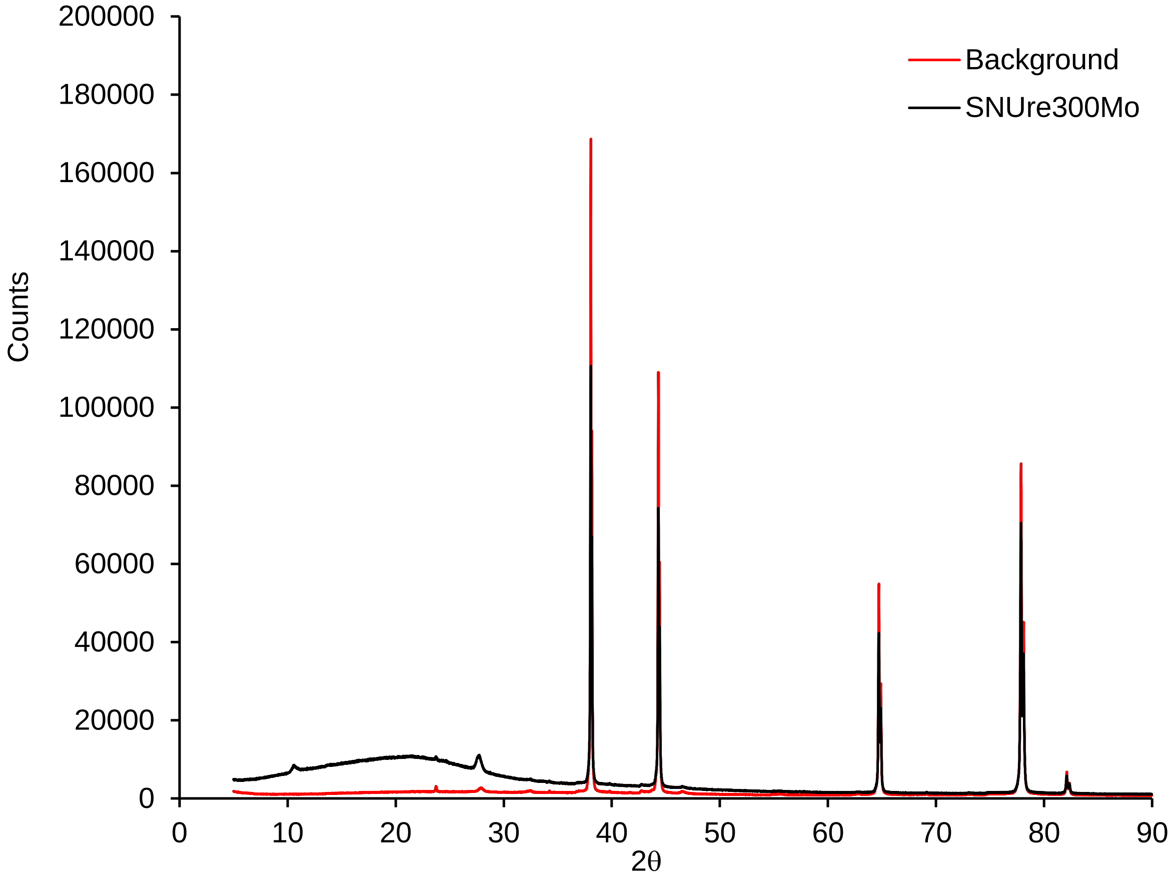
Background
SNUre300Mo
Counts
2θ
200000
180000
160000
140000
120000
100000
80000
60000
40000
20000
0
0
10
20
30
40
50
60
70
80
90

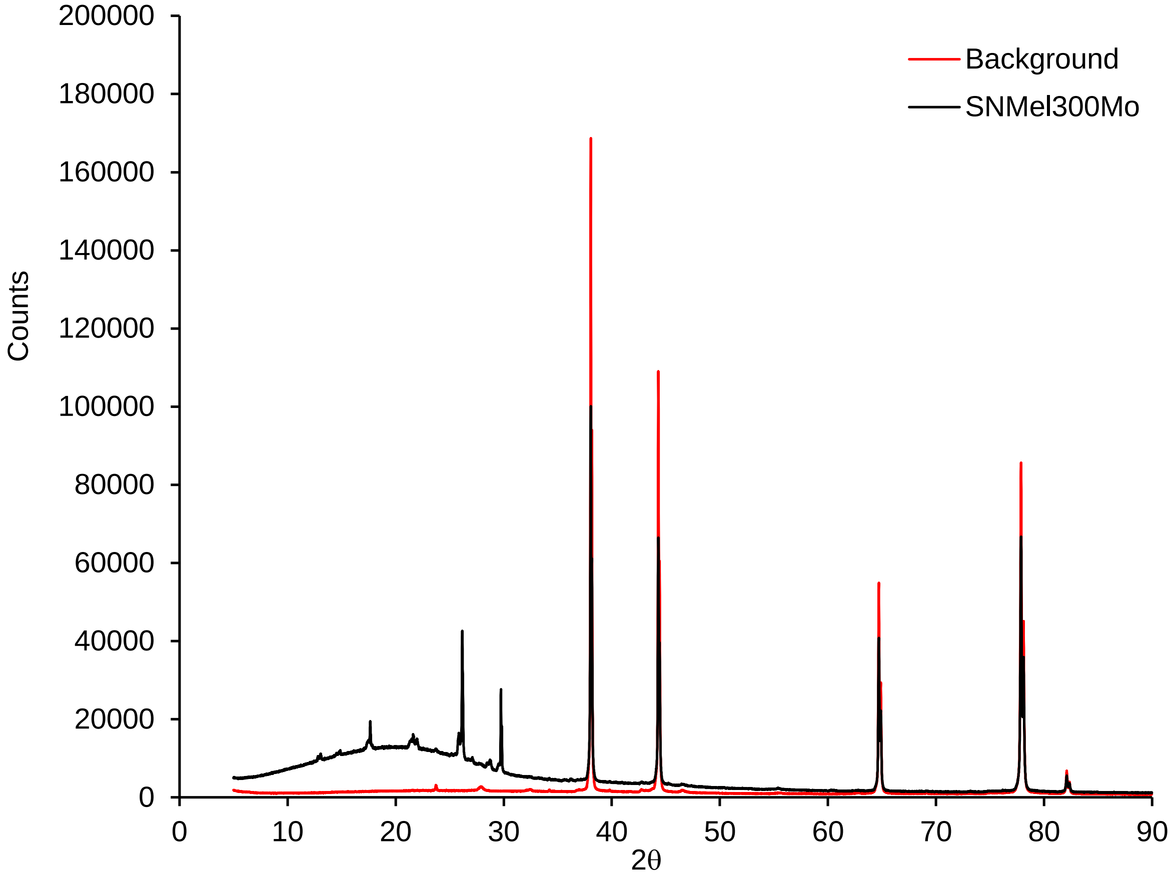
Background
SNMel300Mo
Counts
2θ
0
20000
40000
60000
80000
100000
120000
140000
160000
180000
200000
0
10
20
30
40
50
60
70
80
90

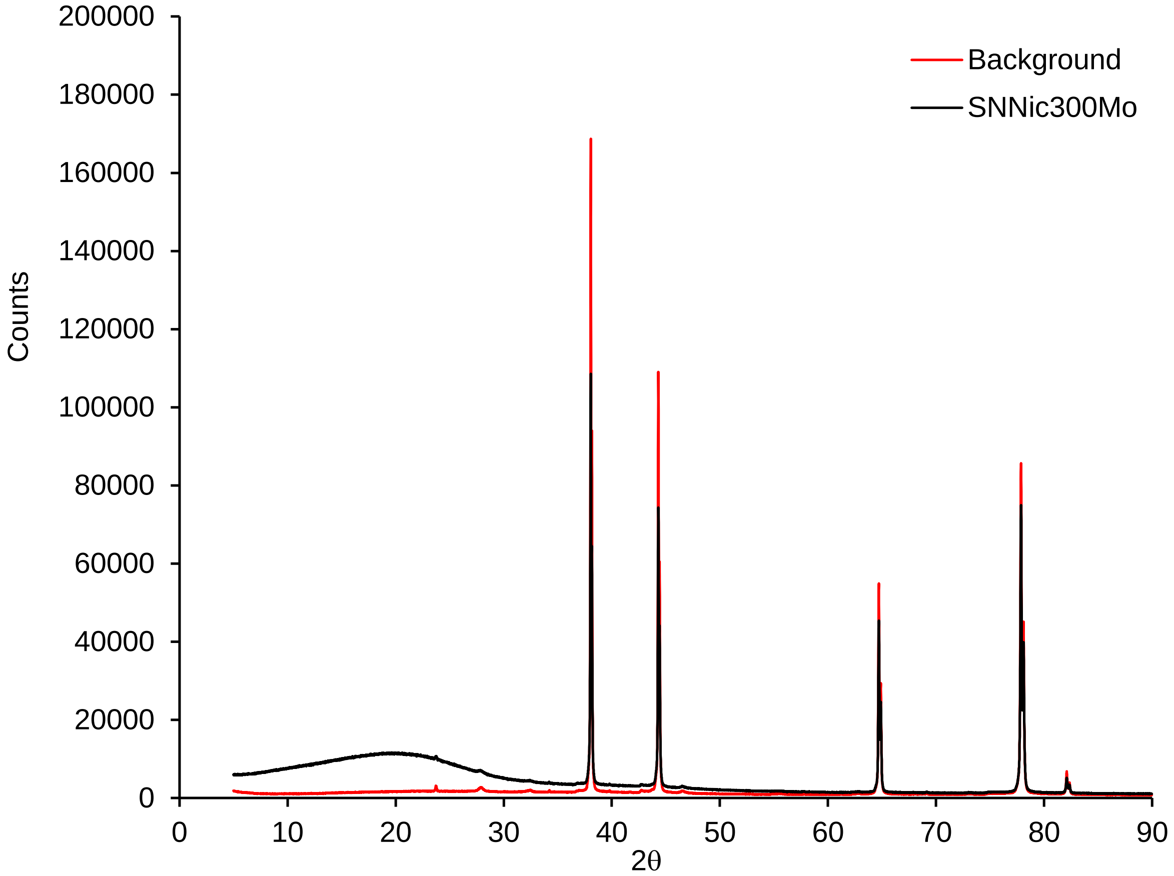
Background
SNNic300Mo
Counts
2θ
0
20000
40000
60000
80000
100000
120000
140000
160000
180000
200000
0
10
20
30
40
50
60
70
80
90

Powder X-ray diffraction data on SN$_{X}$300$_{Mu}$ samples

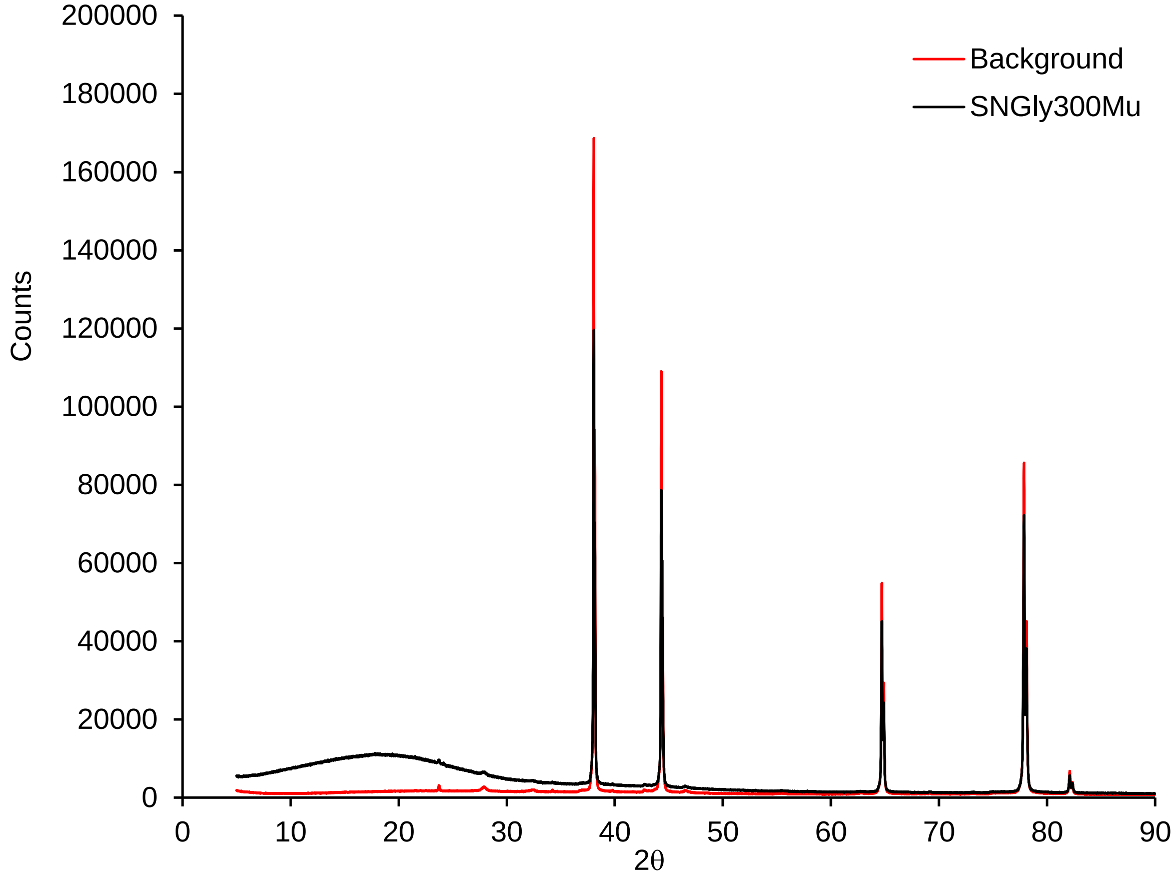

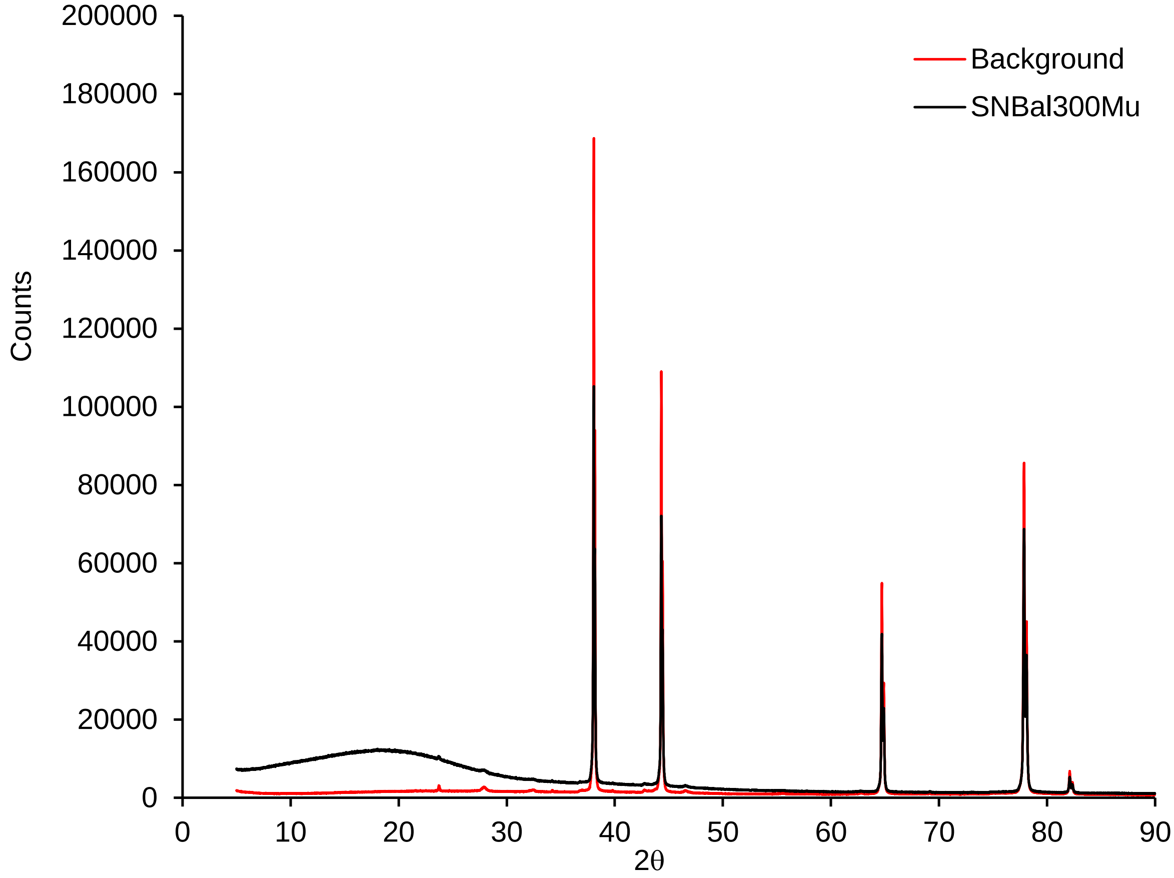

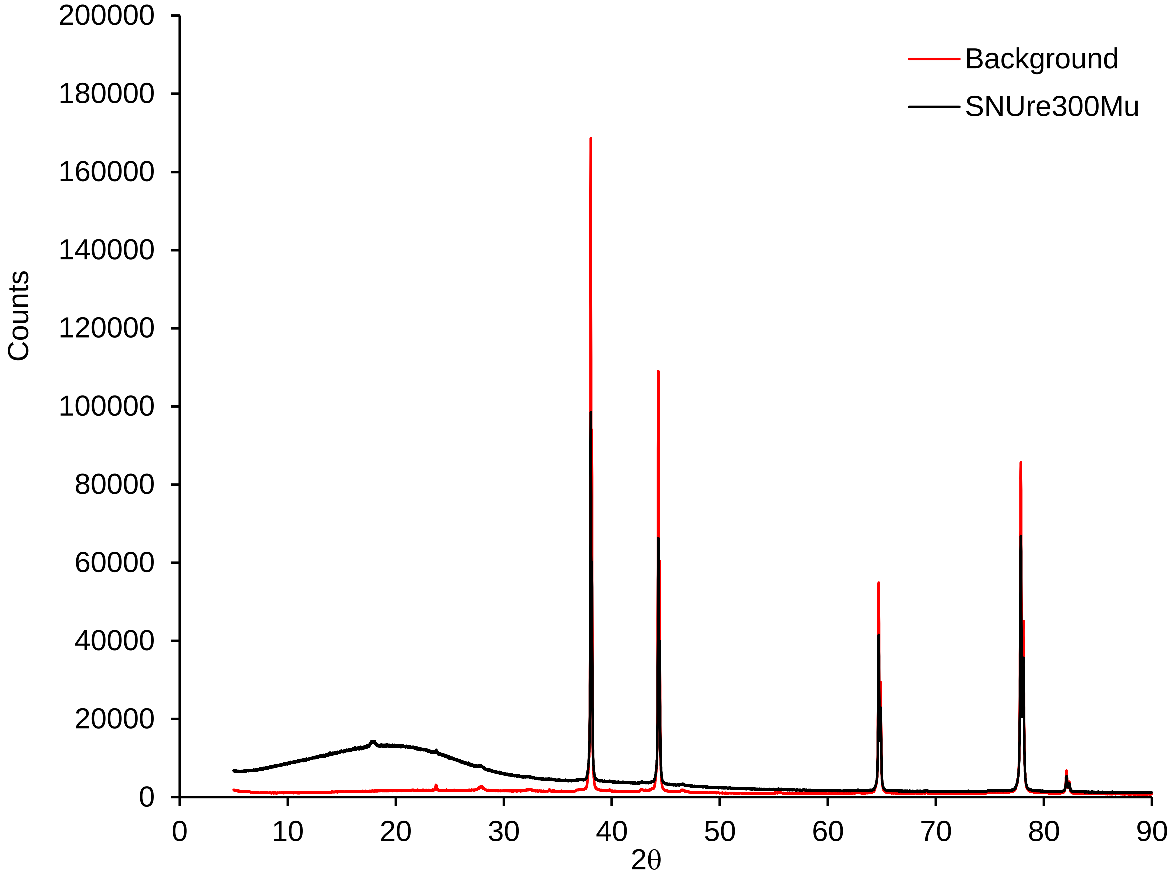
200000
180000
160000
140000
120000
100000
80000
60000
40000
20000
0
Counts
0
10
20
30
40
50
60
70
80
90
2θ
Background
SNUre300Mu

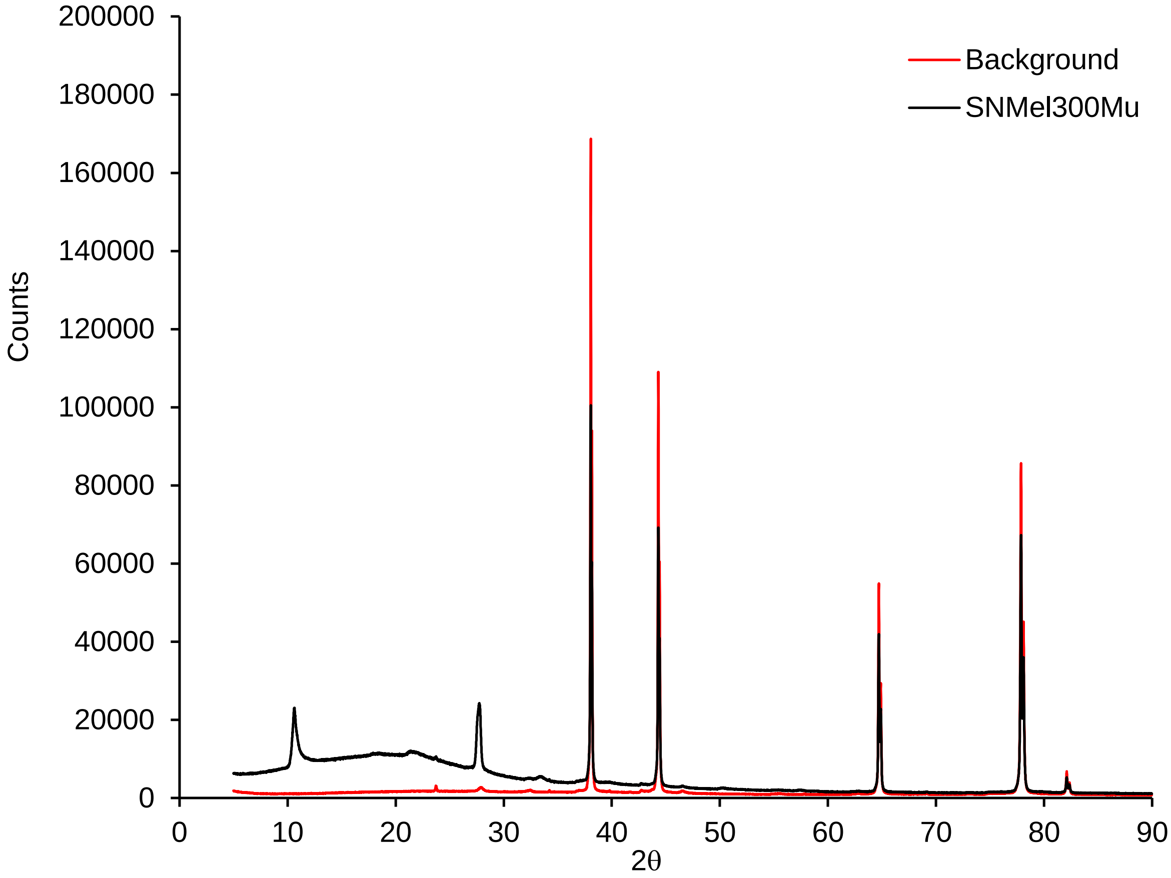
200000
180000
160000
140000
120000
100000
80000
60000
40000
20000
0
Counts
0
10
20
30
40
50
60
70
80
90
2θ
Background
SNMel300Mu

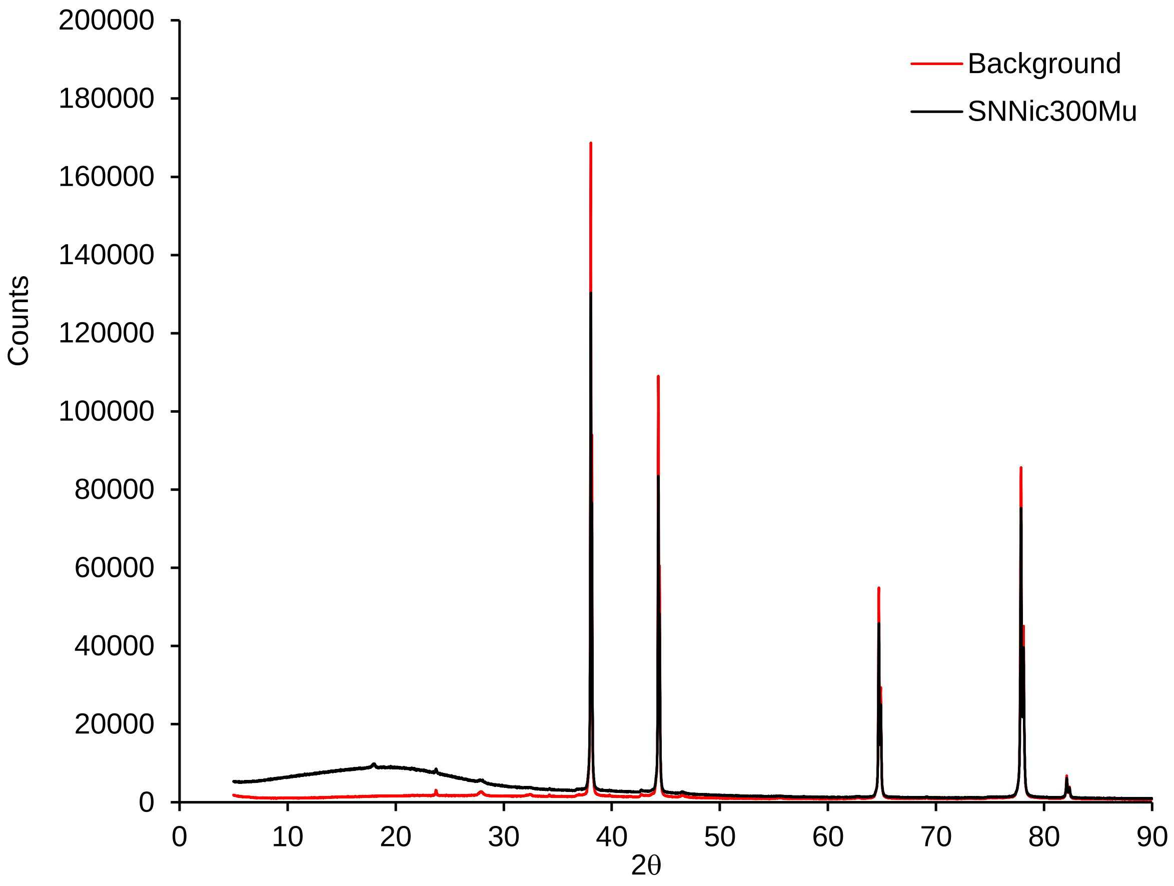
Background
SNNic300Mu
Counts
2θ
200000
180000
160000
140000
120000
100000
80000
60000
40000
20000
0
0
10
20
30
40
50
60
70
80
90

Powder X-ray diffraction data on SN_X800_{Mo} samples

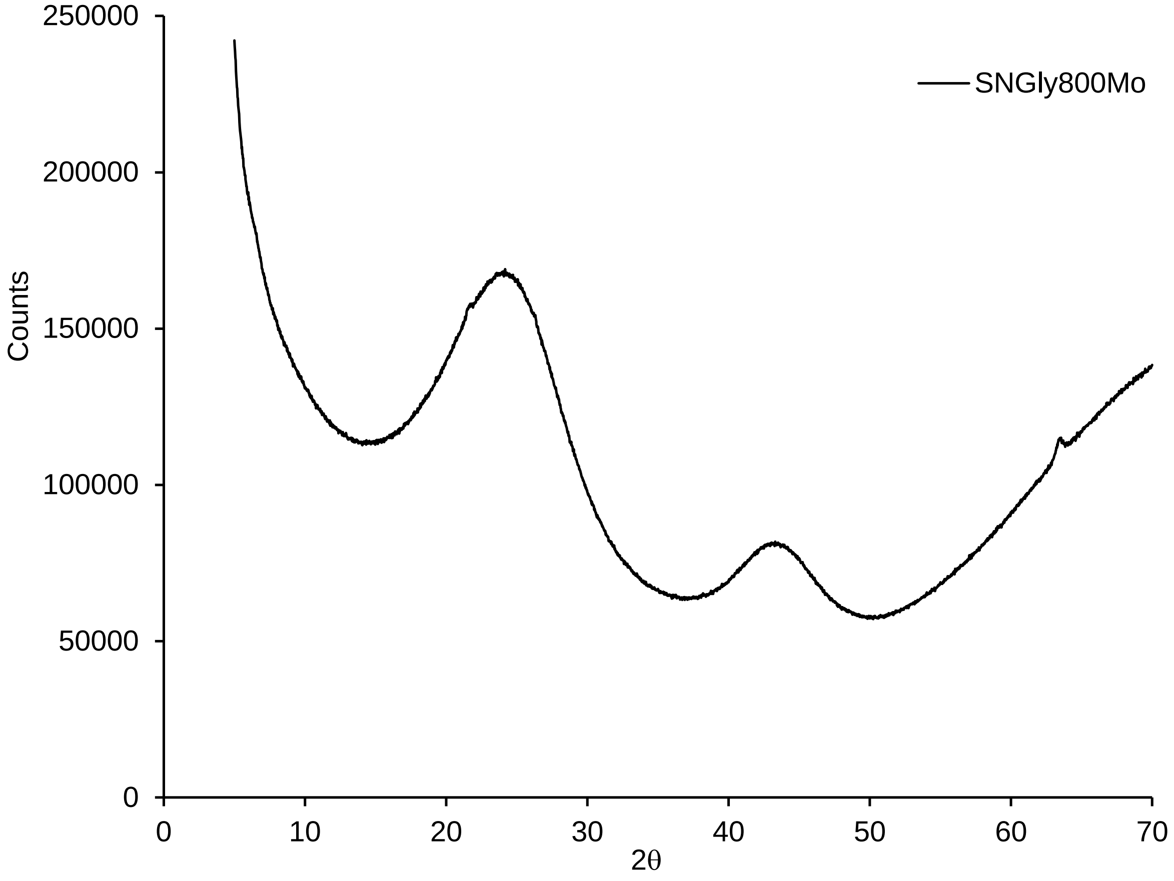

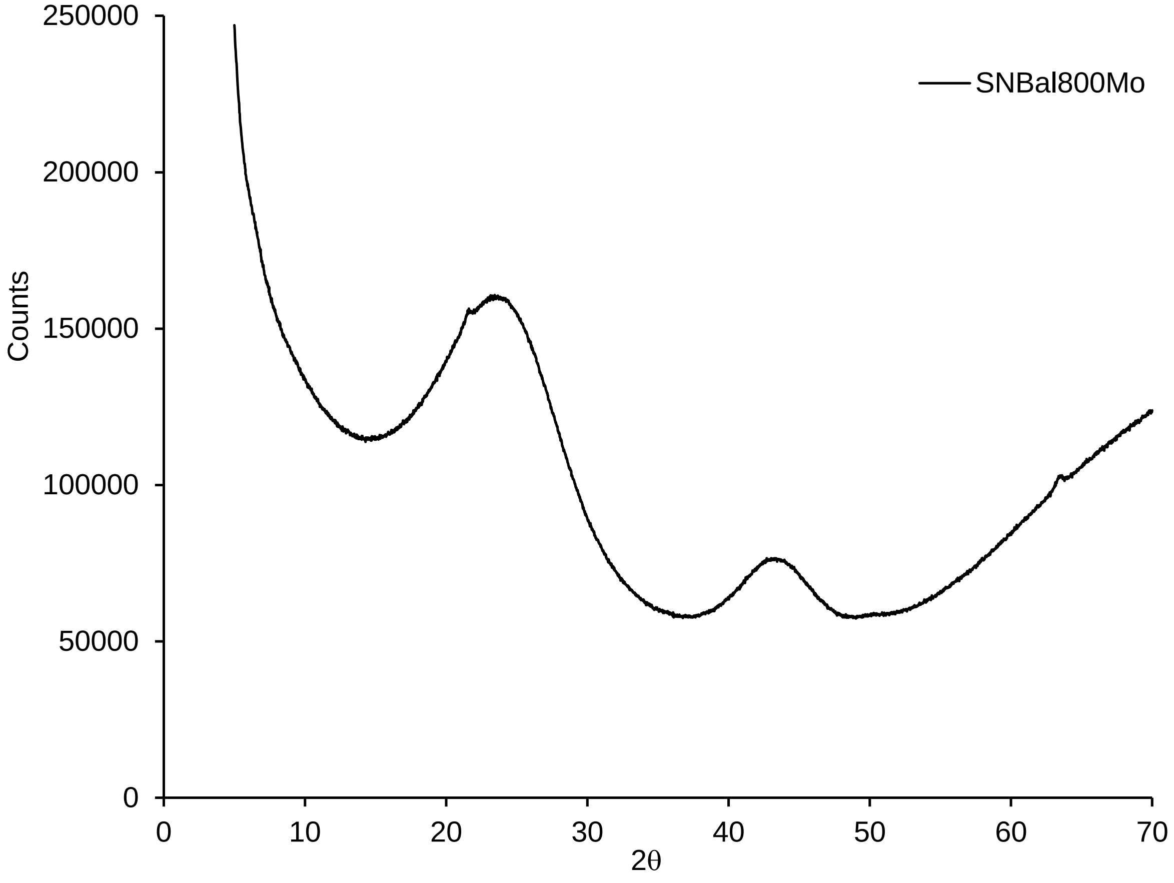

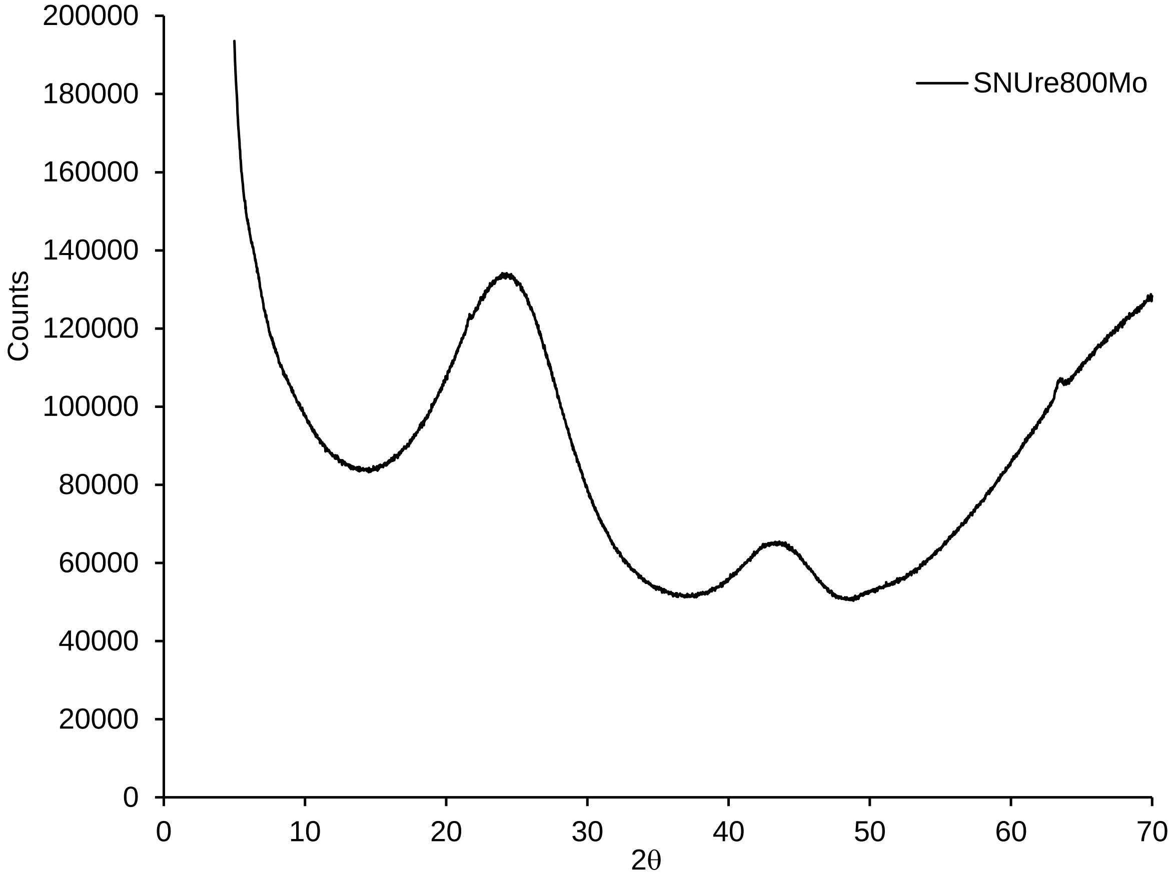

SNUre800Mo
Counts
2θ

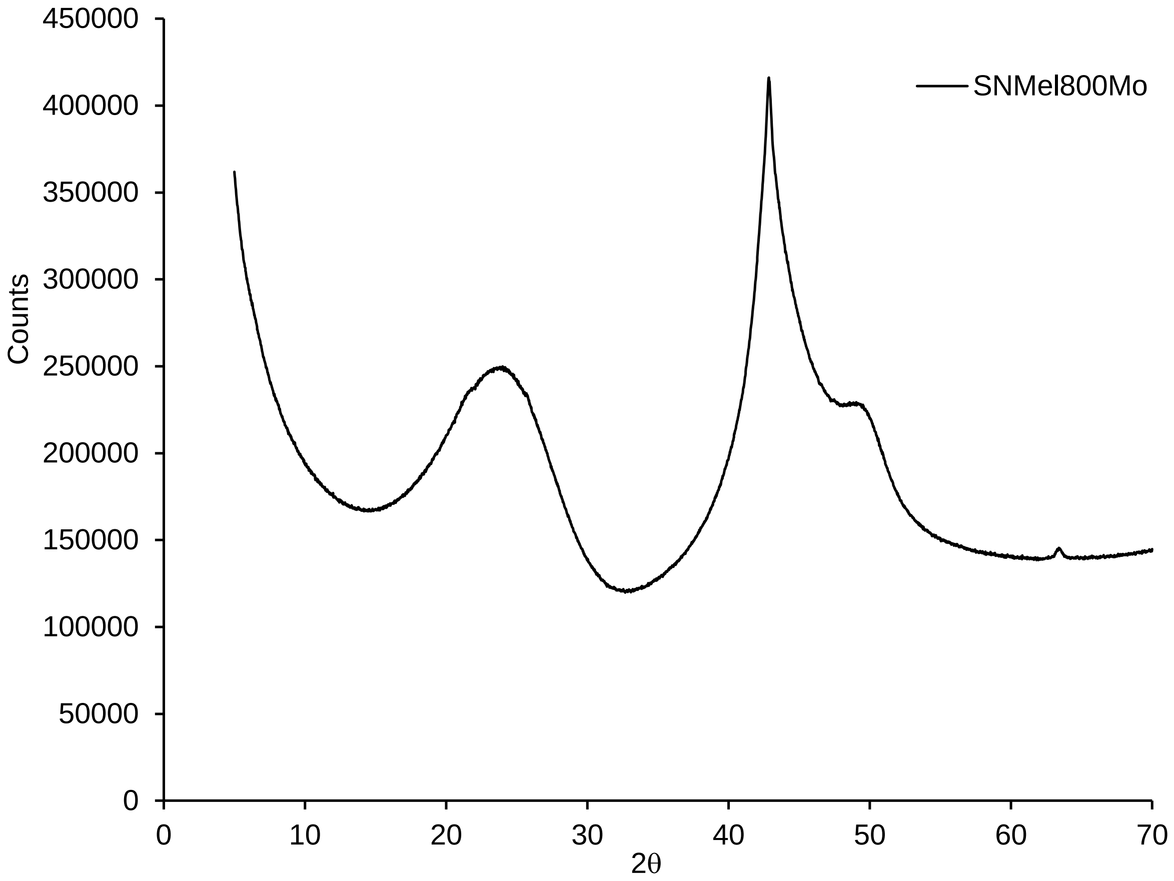

SNMel800Mo
Counts
2θ

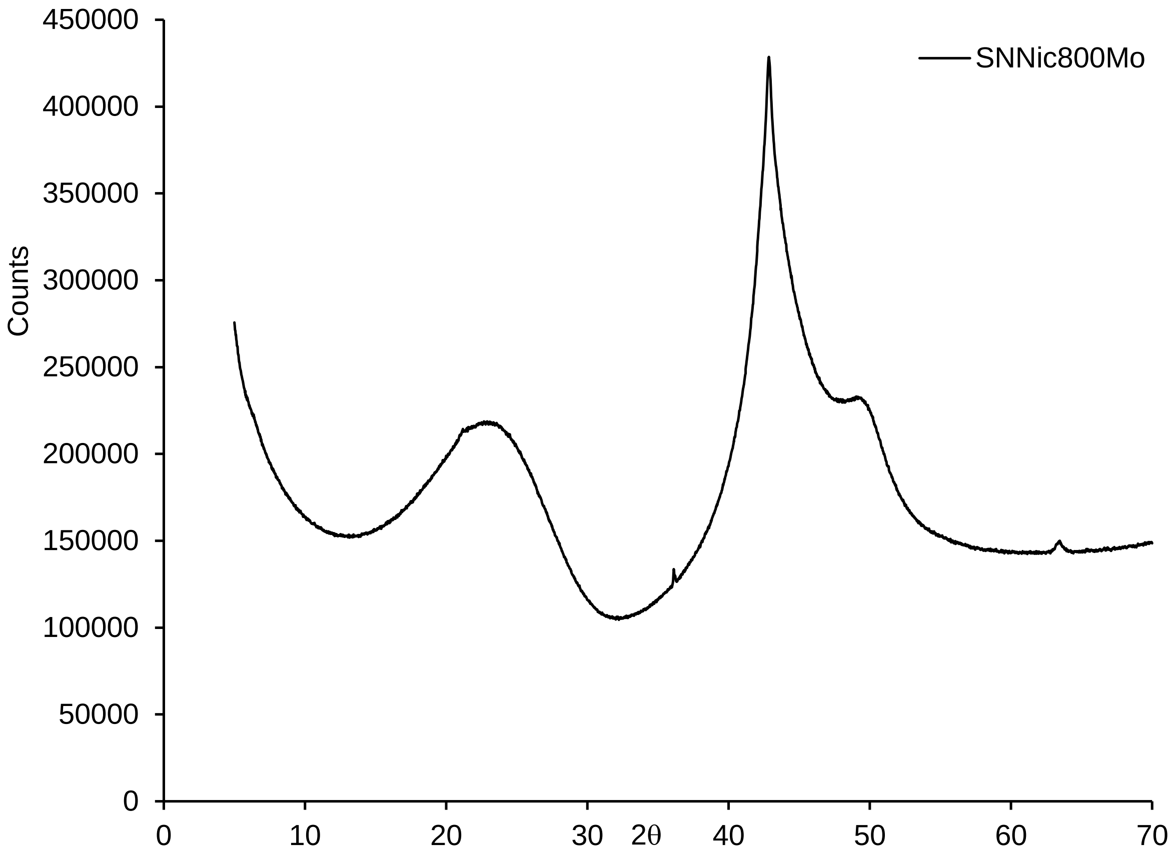
SNNic800Mo
Counts
450000
400000
350000
300000
250000
200000
150000
100000
50000
0
0
10
20
30
2θ
40
50
60
70

Powder X-ray diffraction data on SN_X800_{Mu} samples

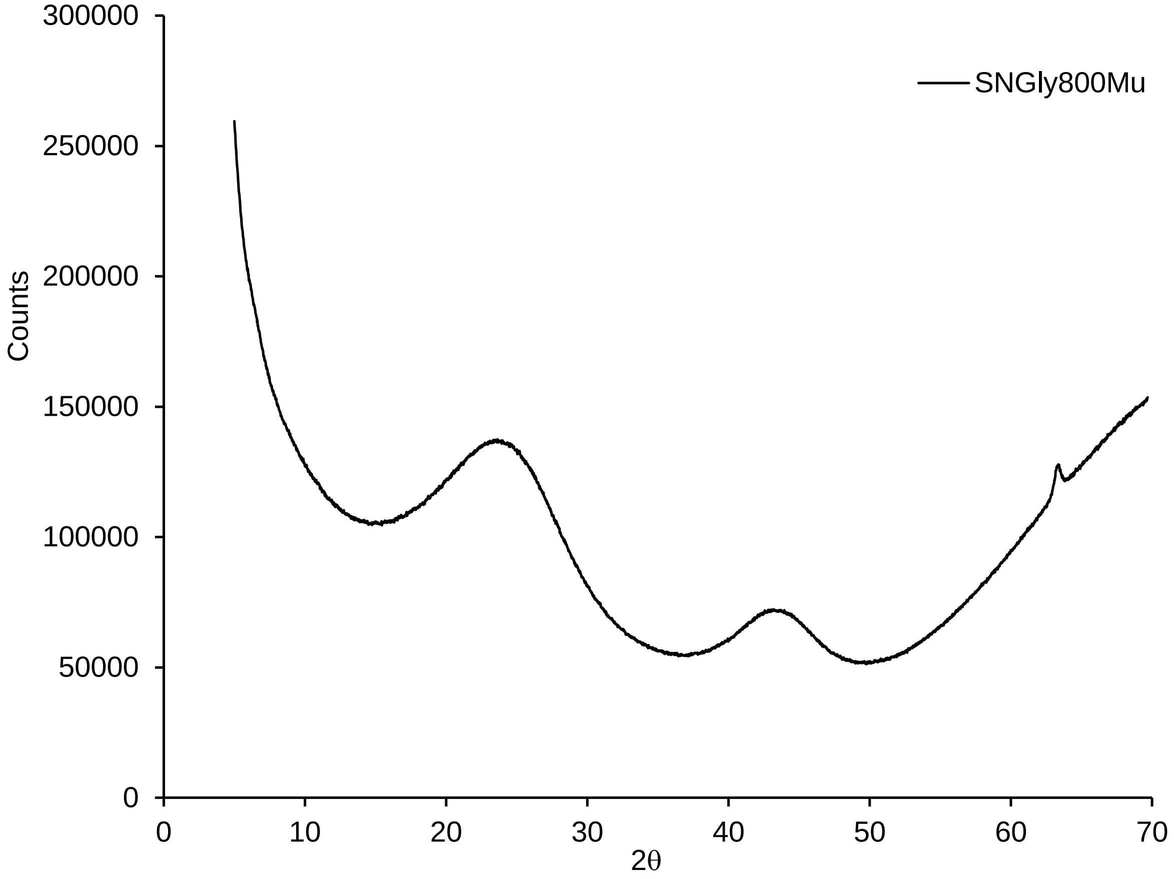

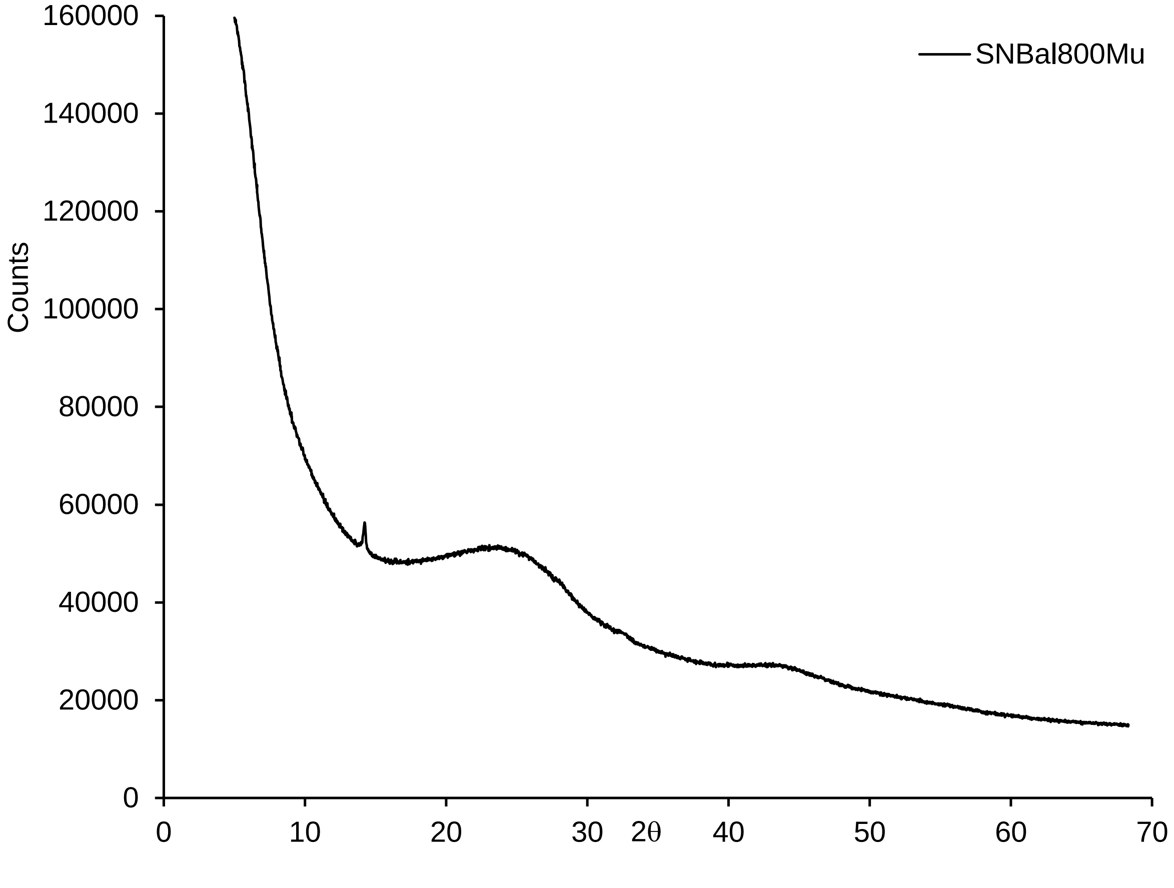

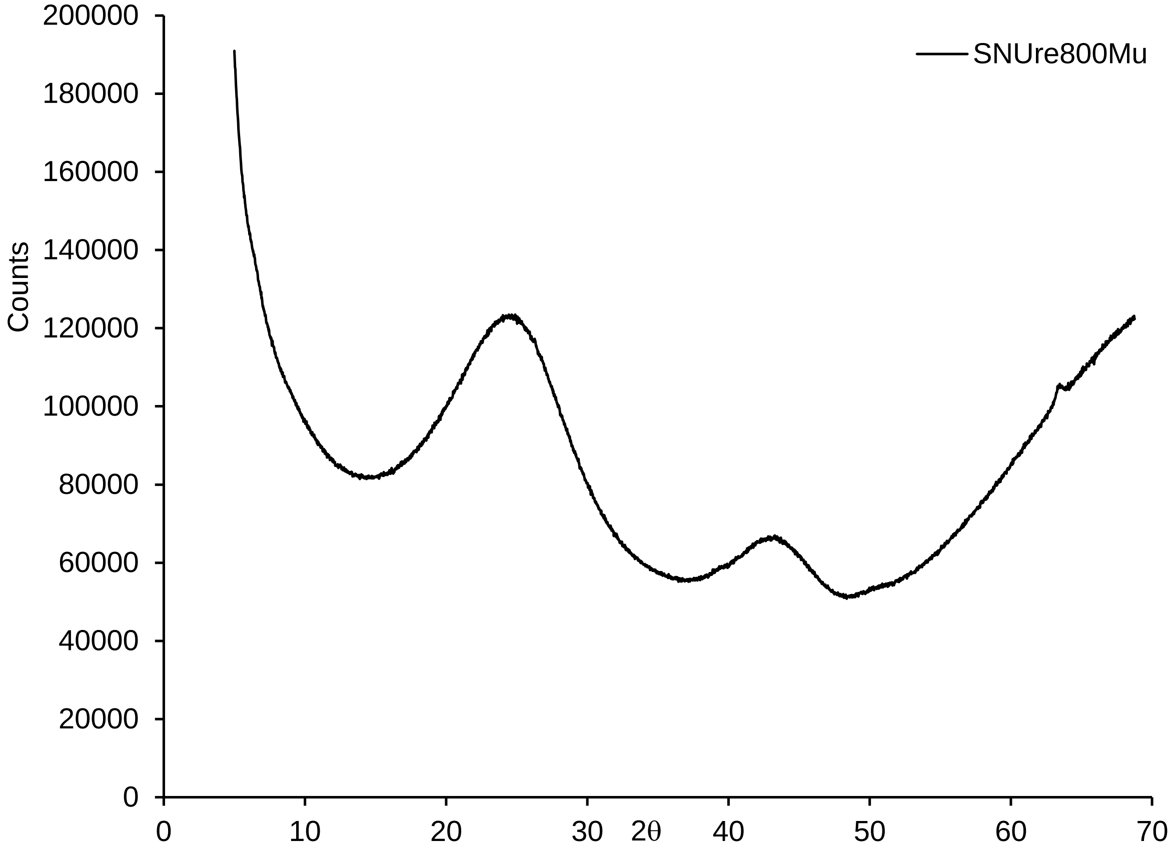
SNUre800Mu
Counts
2θ
200000
180000
160000
140000
120000
100000
80000
60000
40000
20000
0
0
10
20
30
40
50
60
70

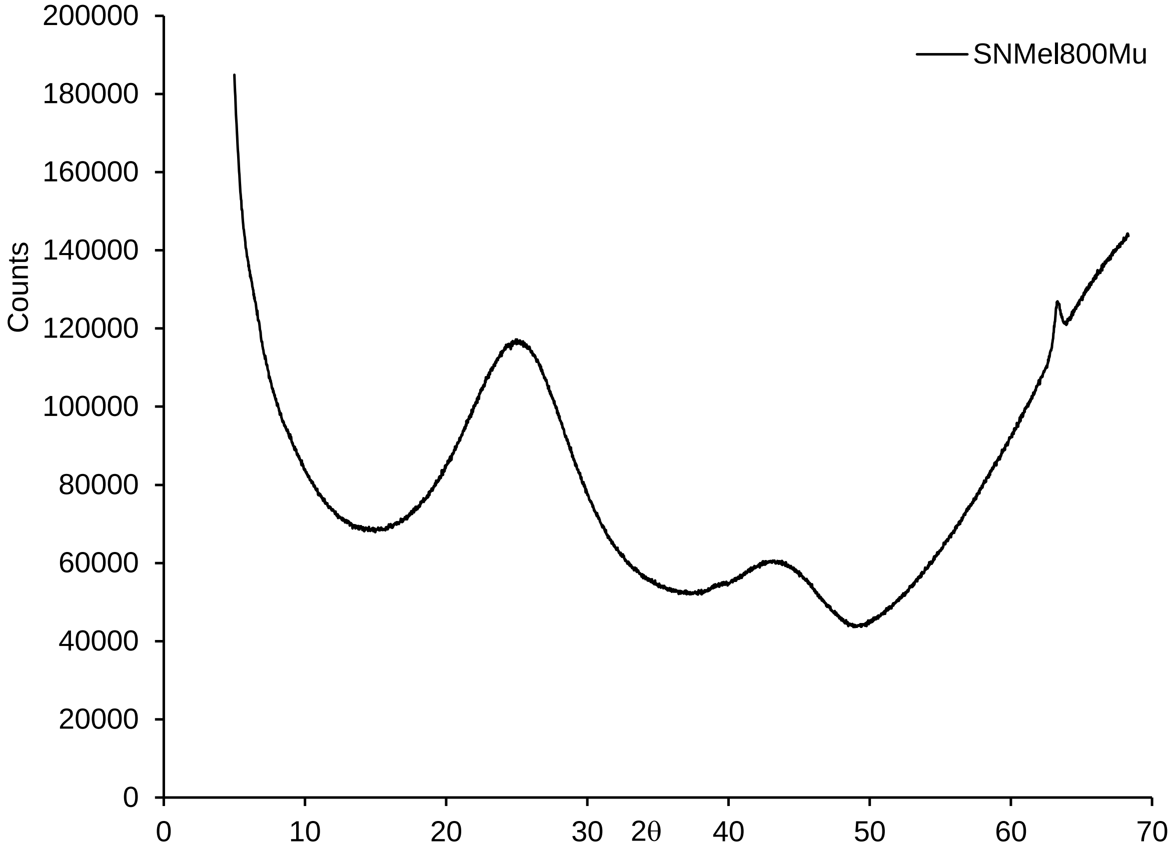
SNMel800Mu
Counts
2θ
200000
180000
160000
140000
120000
100000
80000
60000
40000
20000
0
0
10
20
30
40
50
60
70

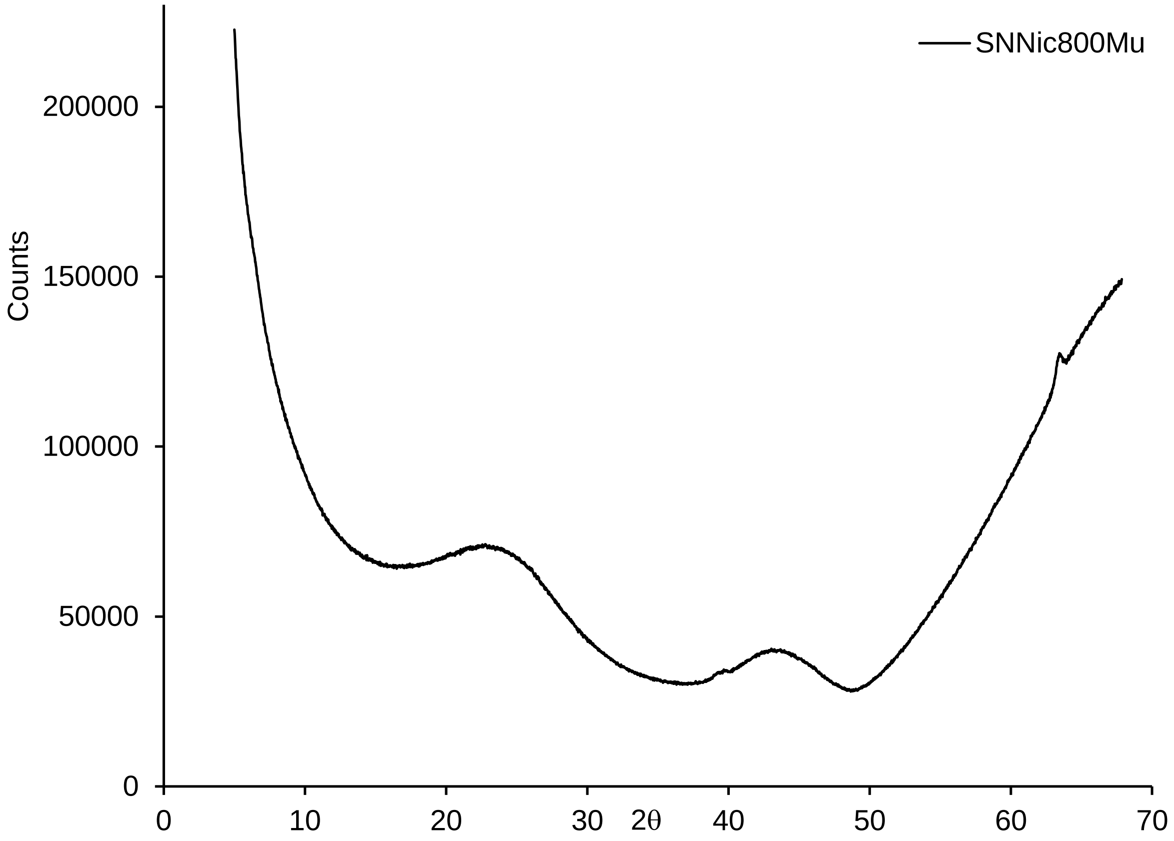
SNNic800Mu
Counts
200000
150000
100000
50000
0
0
10
20
30
2θ
40
50
60
70

Solid-state ^{13}C NMR spectrum of S300

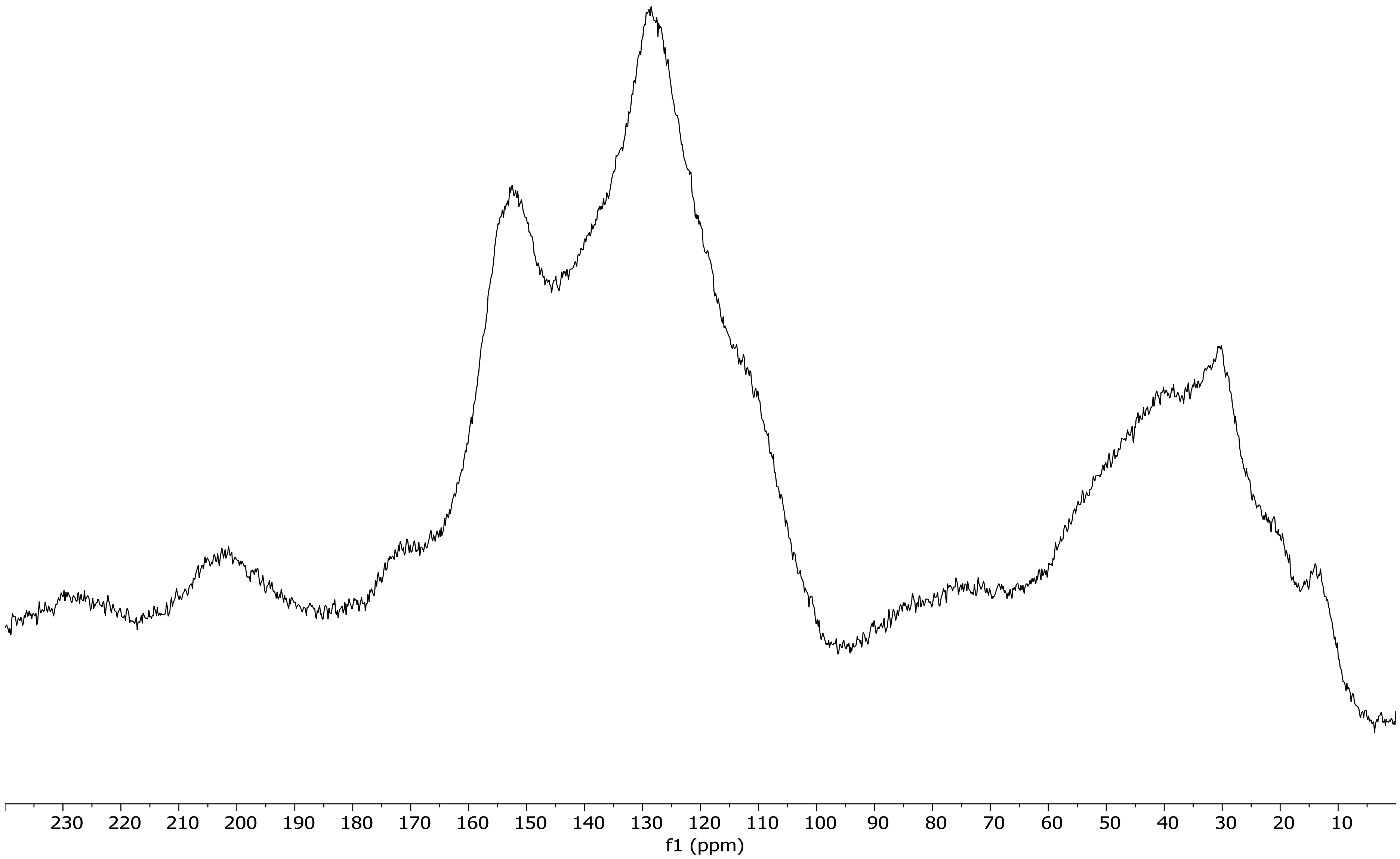

Solid-state ^{13}C NMR spectrum of $SN_{Gly}300_{Th}$

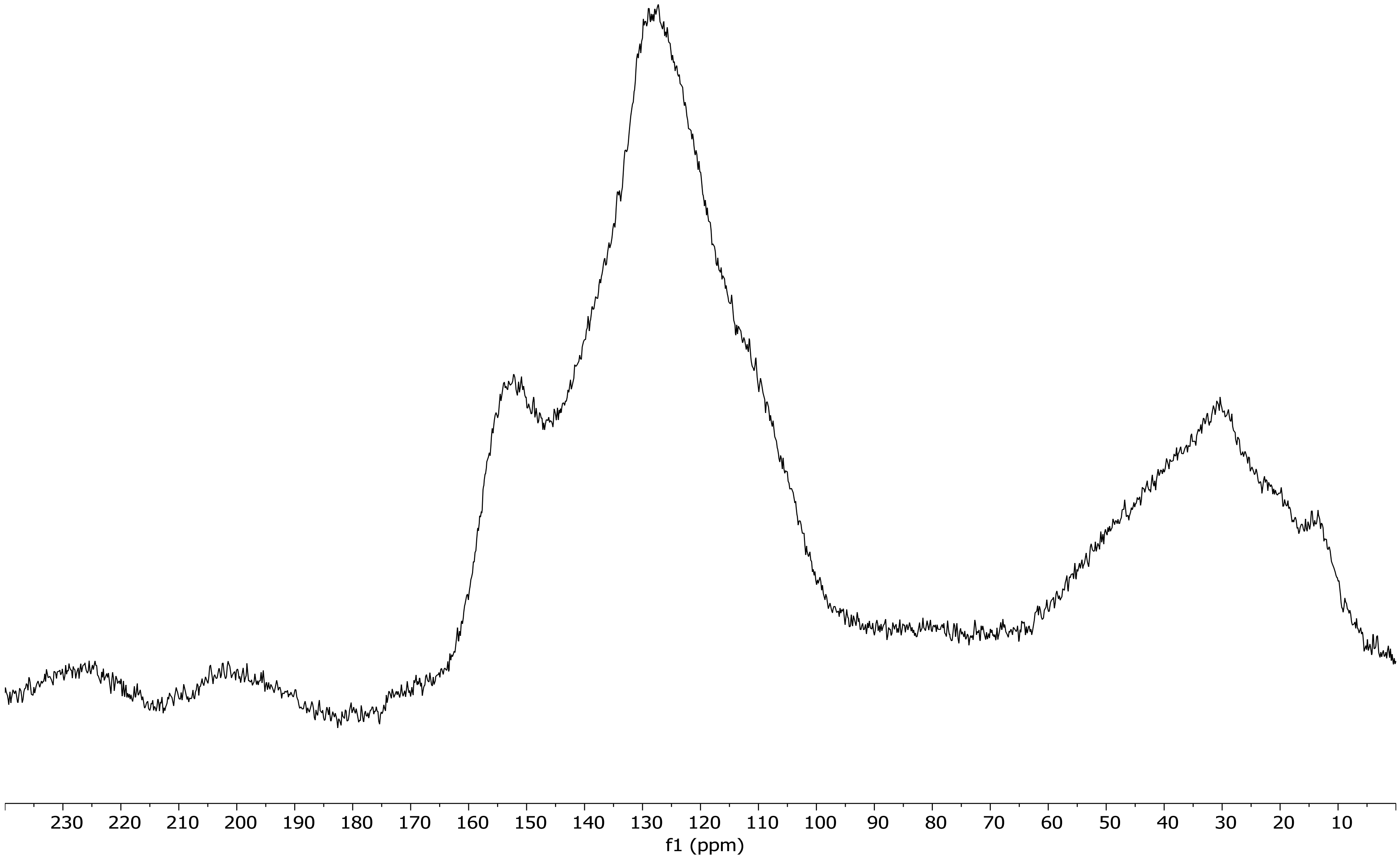

Solid-state ^{13}C NMR spectrum of $SN_{Bal}300_{Th}$

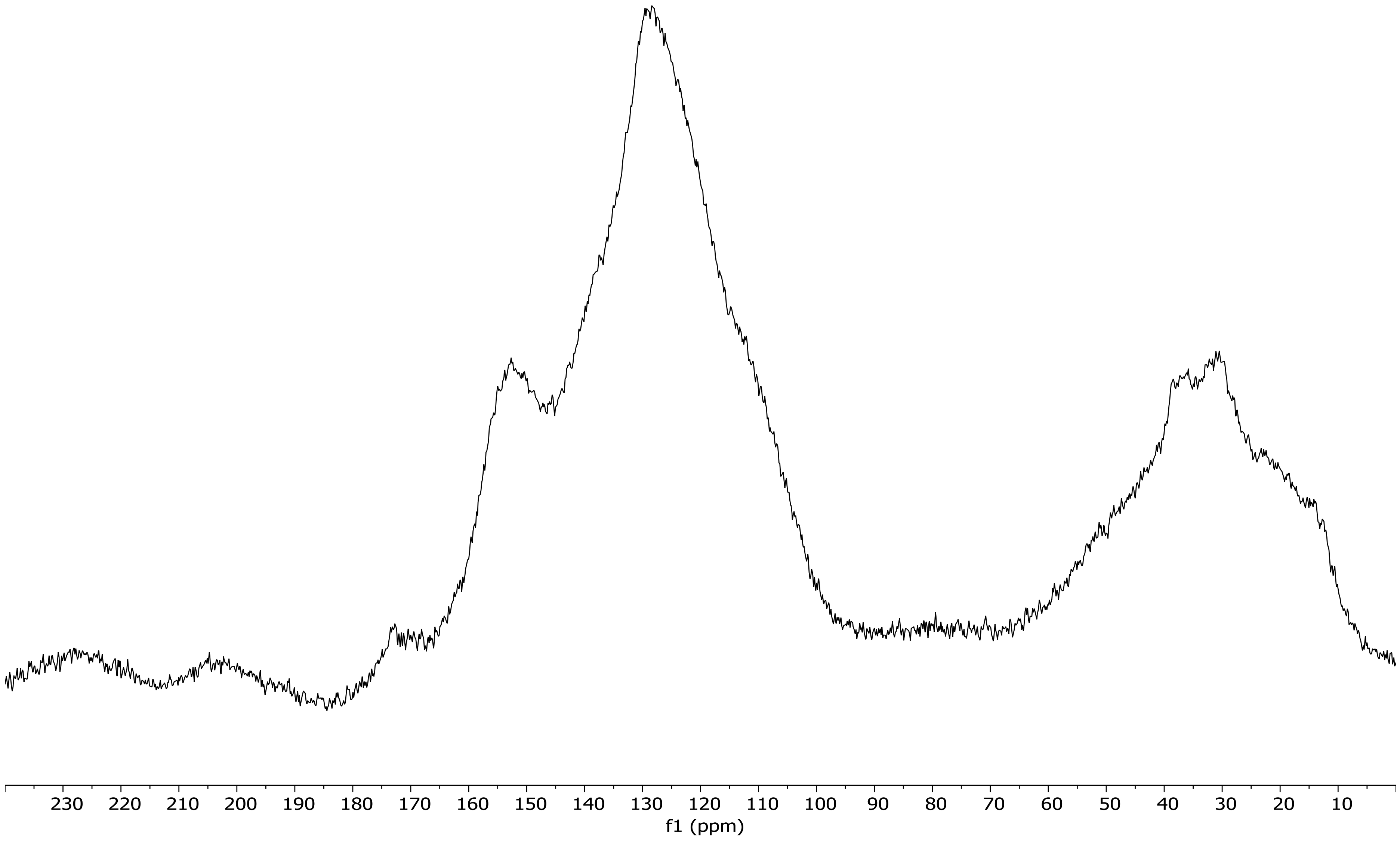

Solid-state ^{13}C NMR spectrum of $SN_{Ure}300_{Th}$

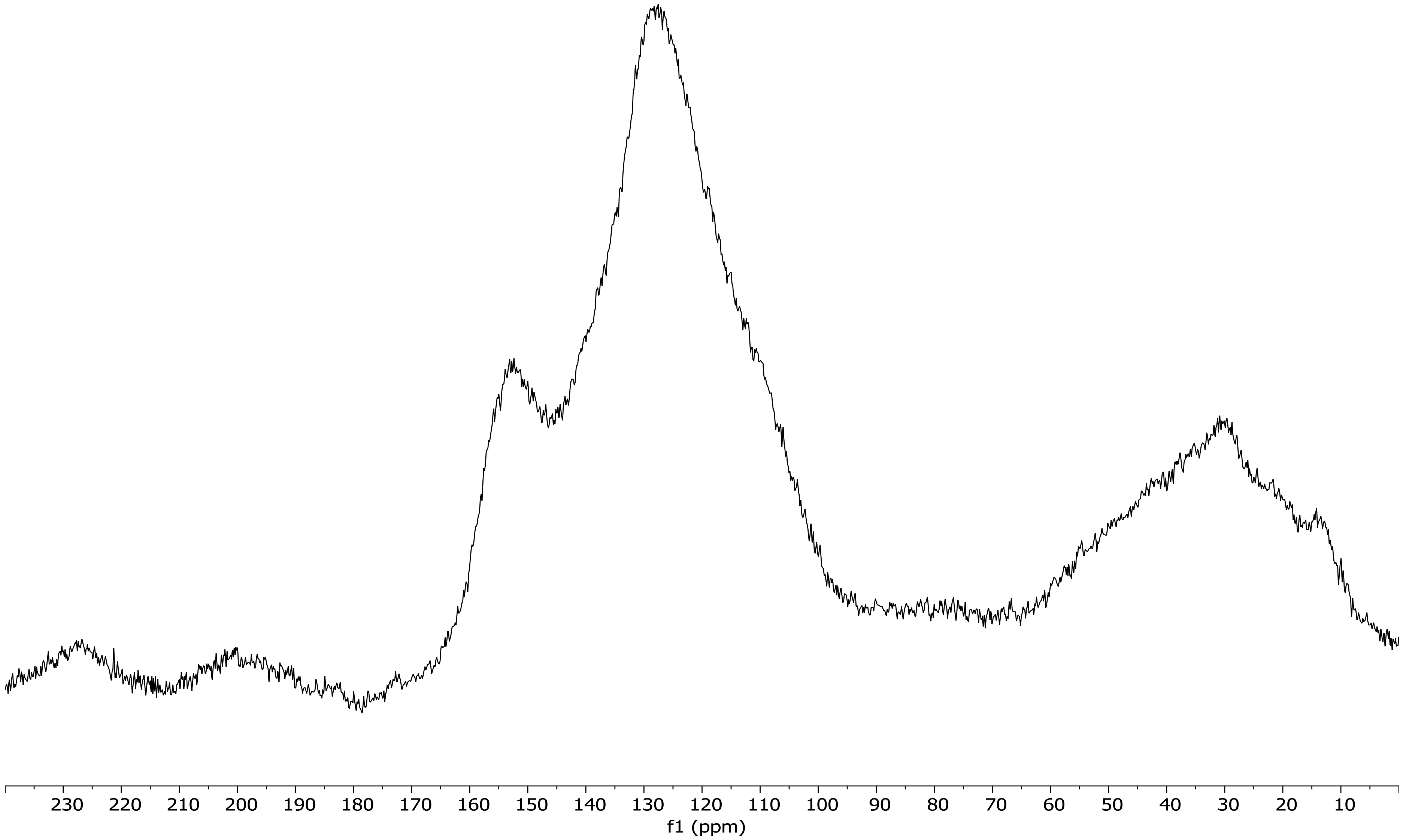

Solid-state ^{13}C NMR spectrum of $SN_{Mel}300_{Th}$

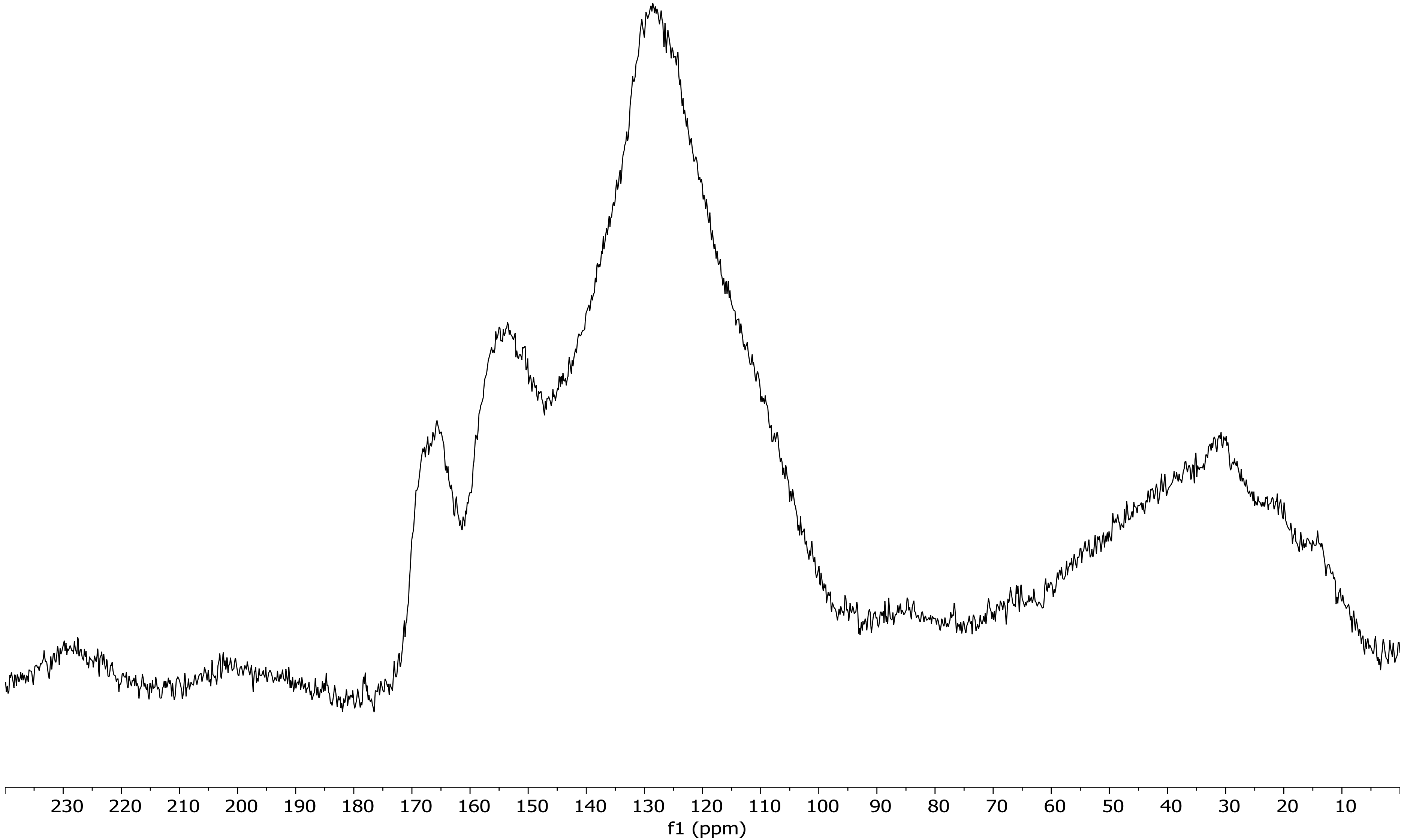

Solid-state ^{13}C NMR spectrum of $SN_{Nic}300_{Th}$

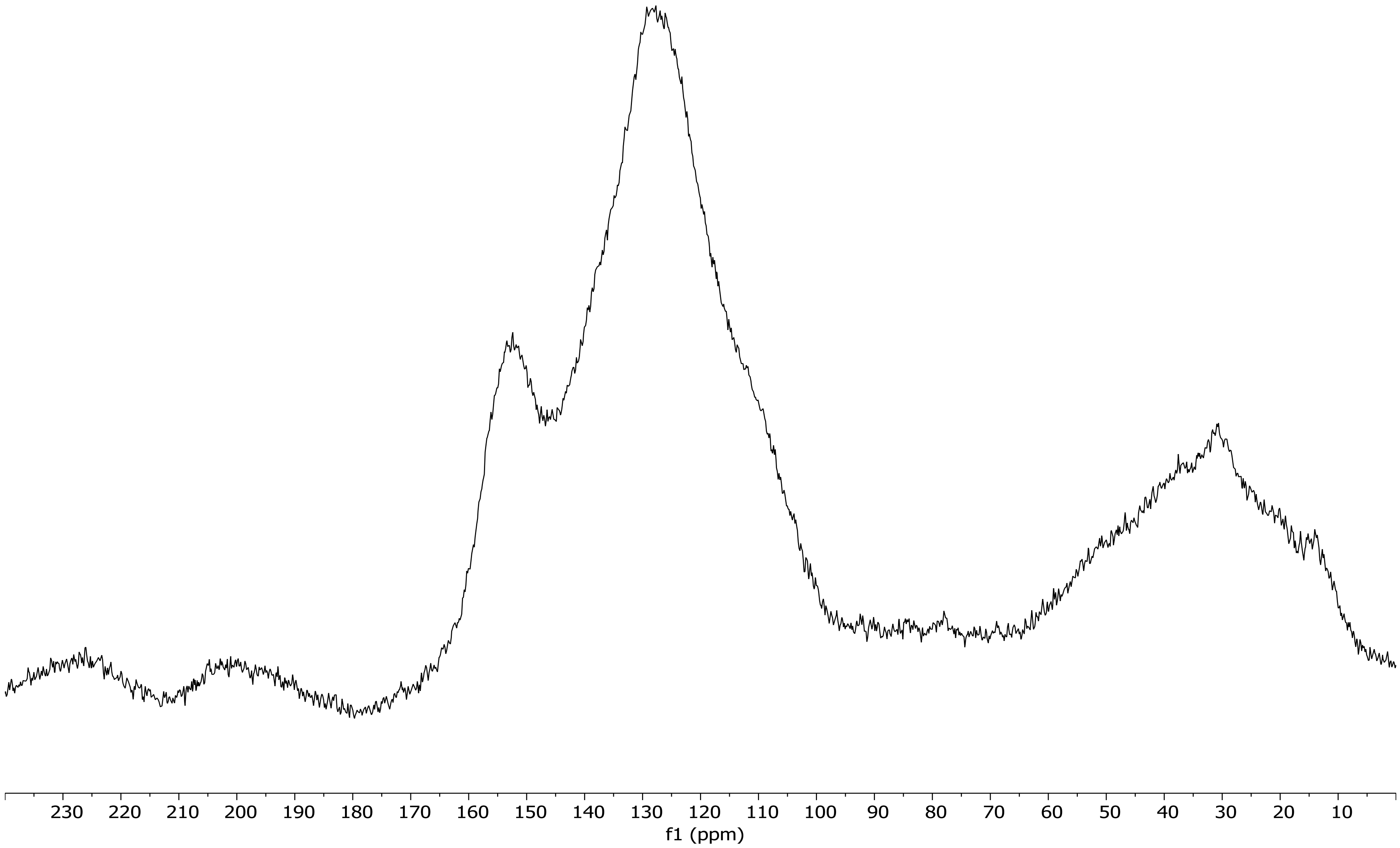

Solid-state ^{13}C NMR spectrum of $SN_{Gly}300_{Mo}$

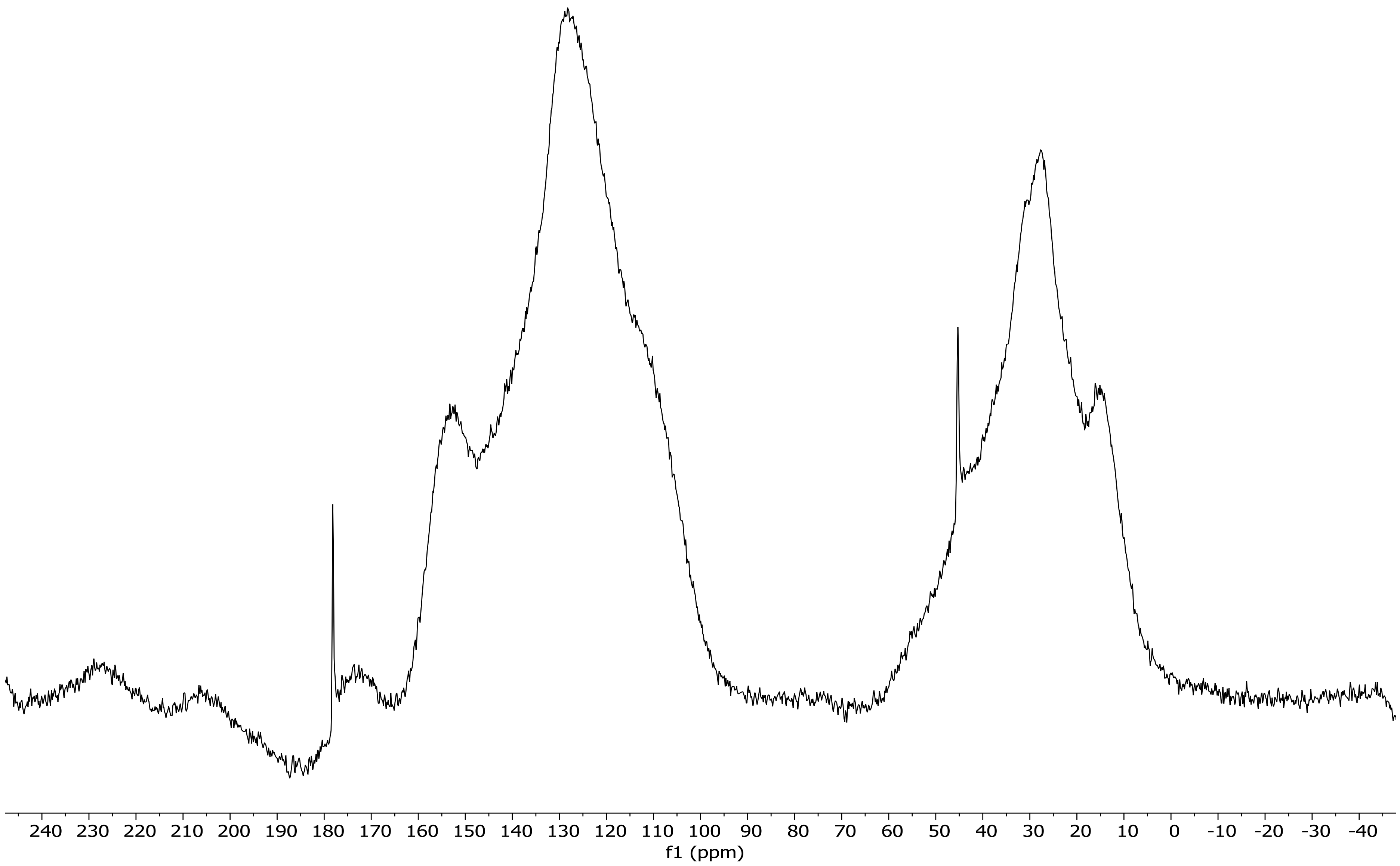

Solid-state ^{13}C NMR spectrum of $SN_{Bal}300_{Mo}$

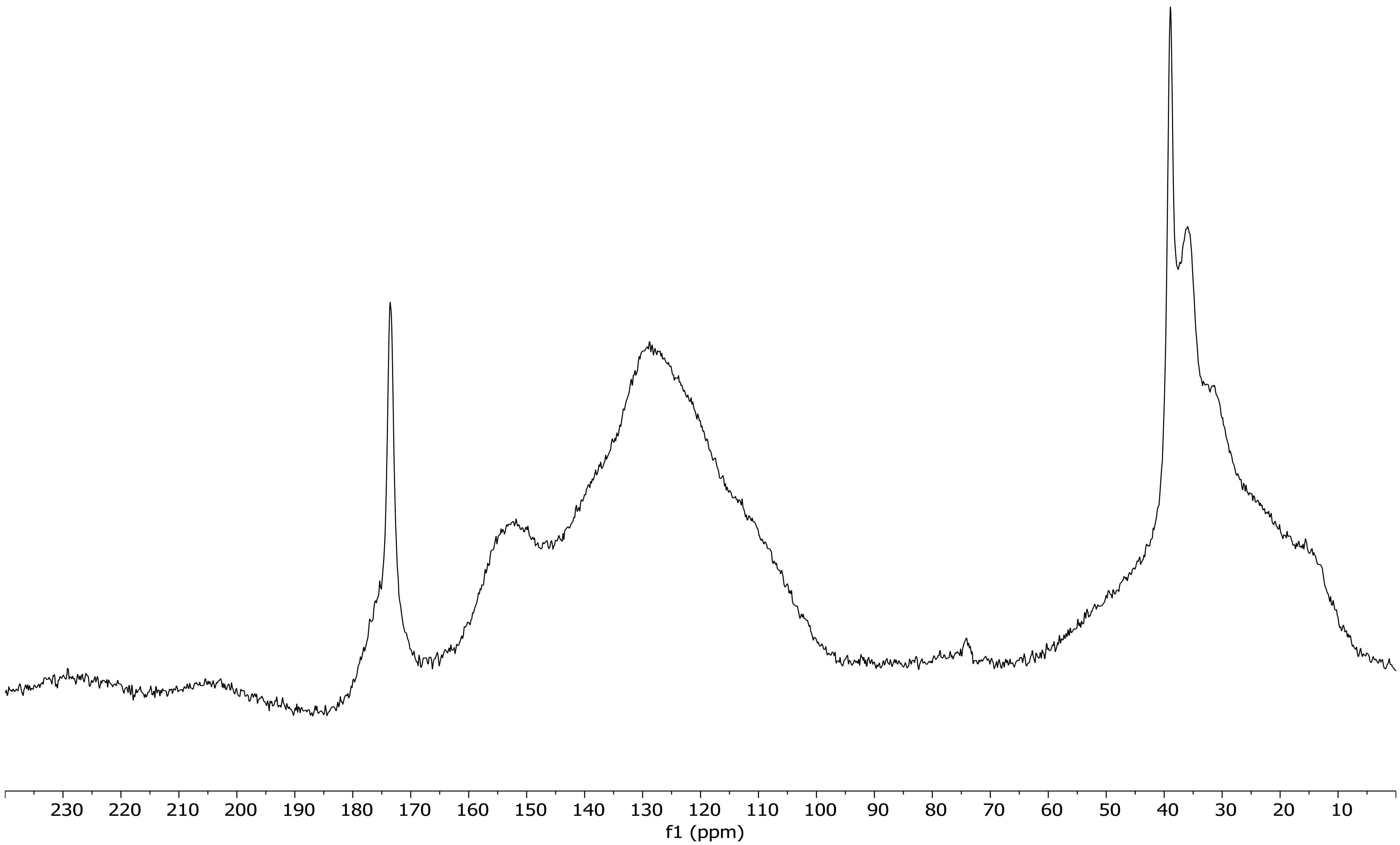

Solid-state ^{13}C NMR spectrum of $SN_{Ure}300_{Mo}$

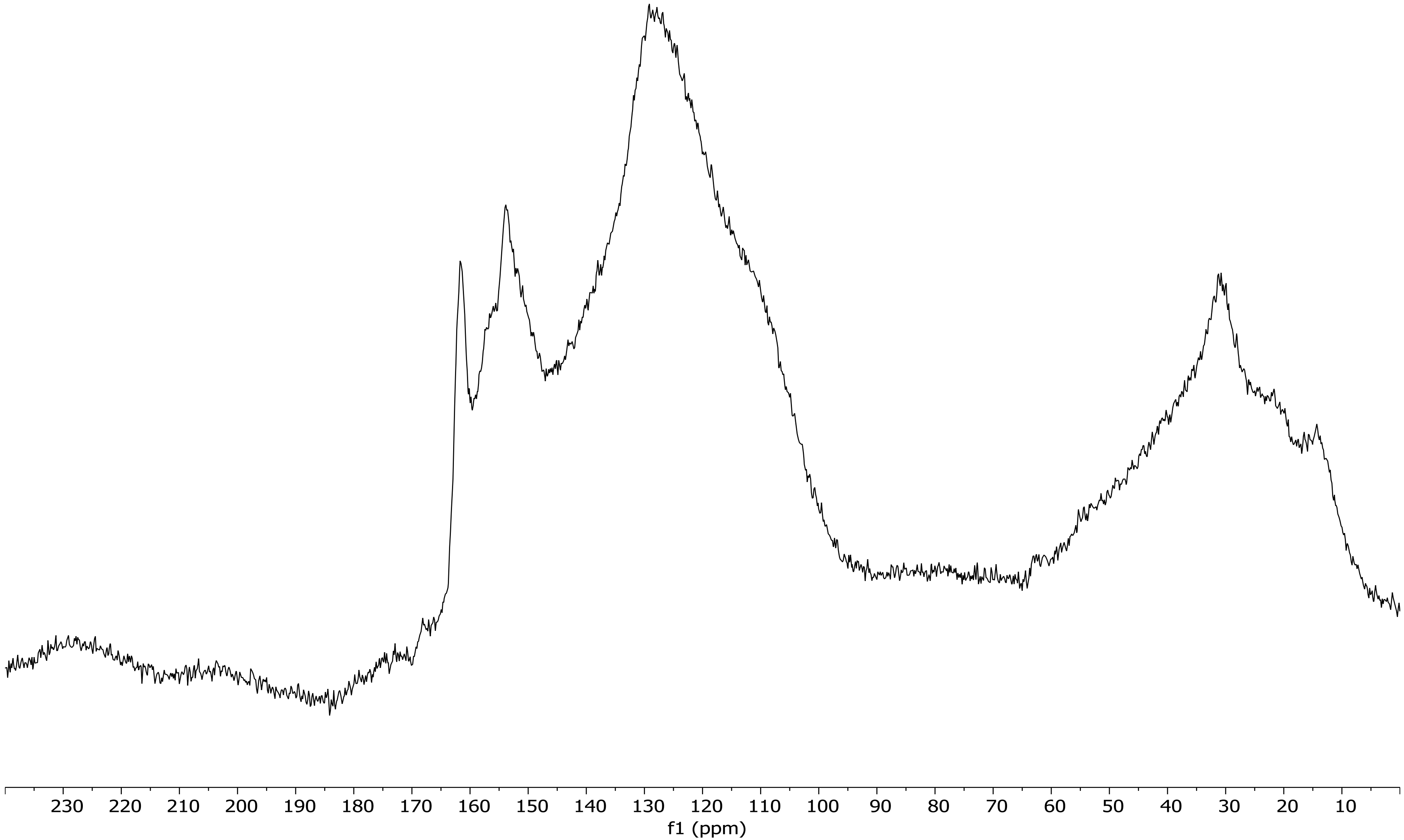

Solid-state ^{13}C NMR spectrum of $SN_{Mel}300_{Mo}$

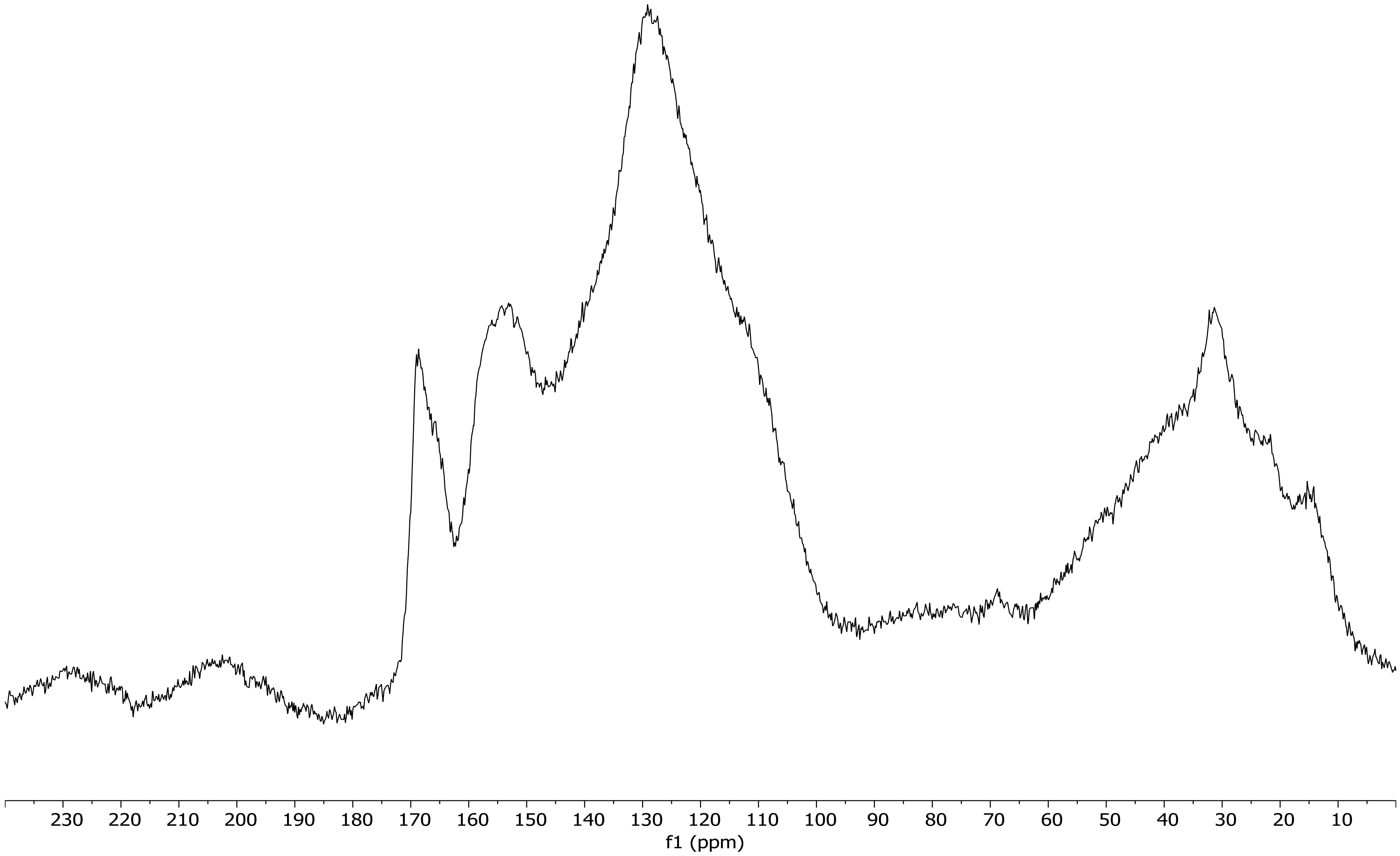

Solid-state ^{13}C NMR spectrum of $SN_{Nic}300_{Mo}$

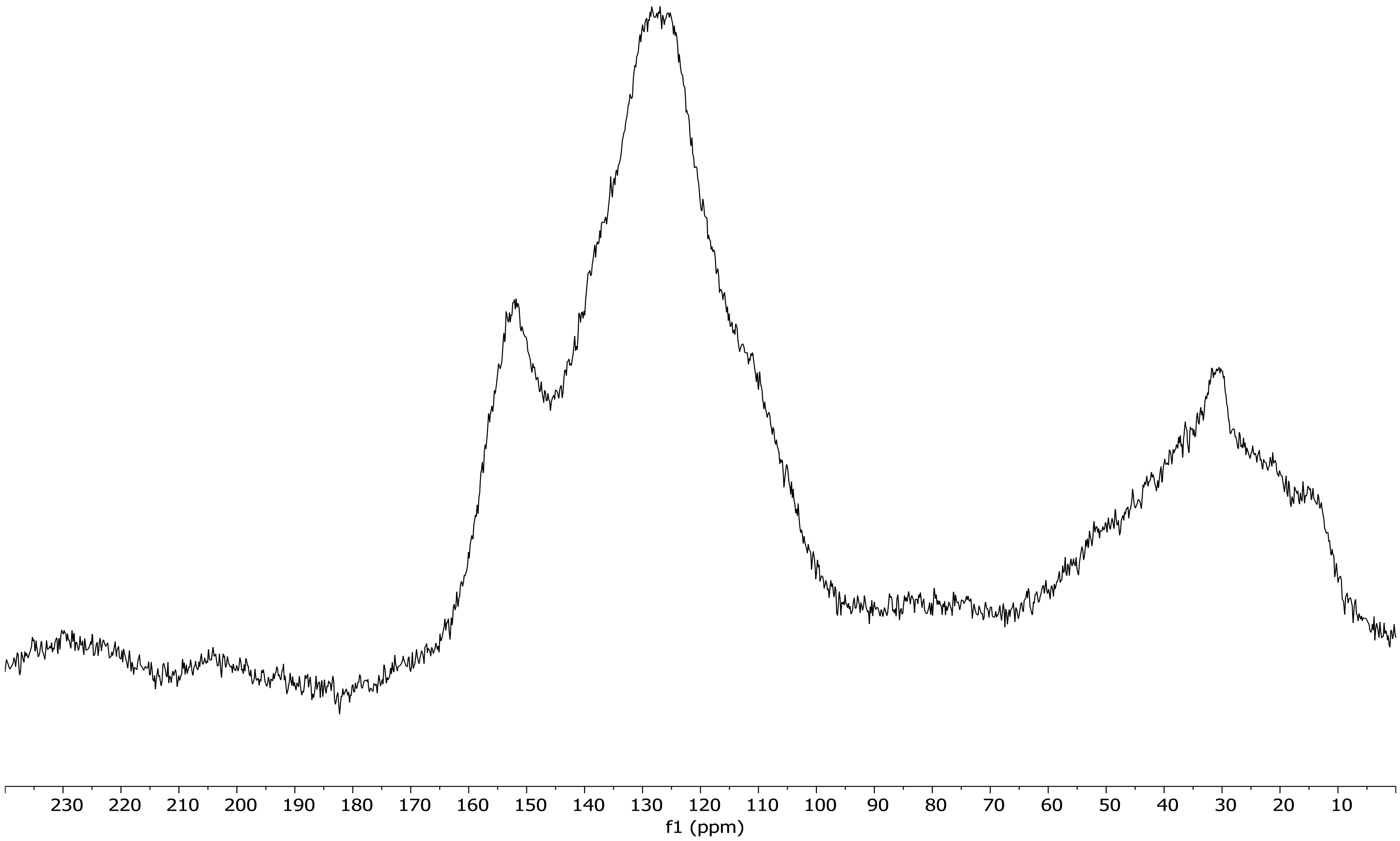

Solid-state ^{13}C NMR spectrum of $SN_{Gly}300_{Mu}$

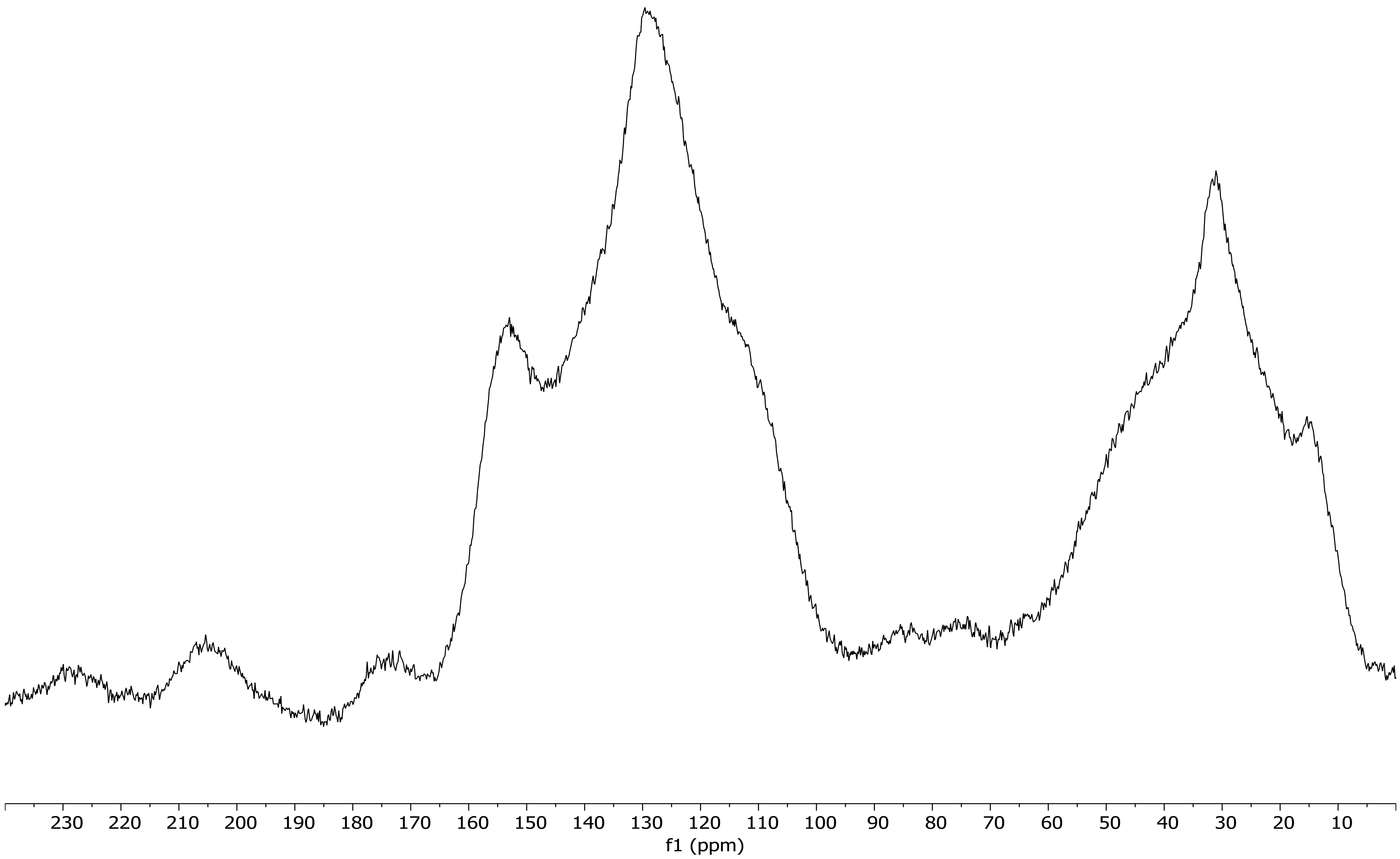

Solid-state ^{13}C NMR spectrum of $SN_{Bal}300_{Mu}$

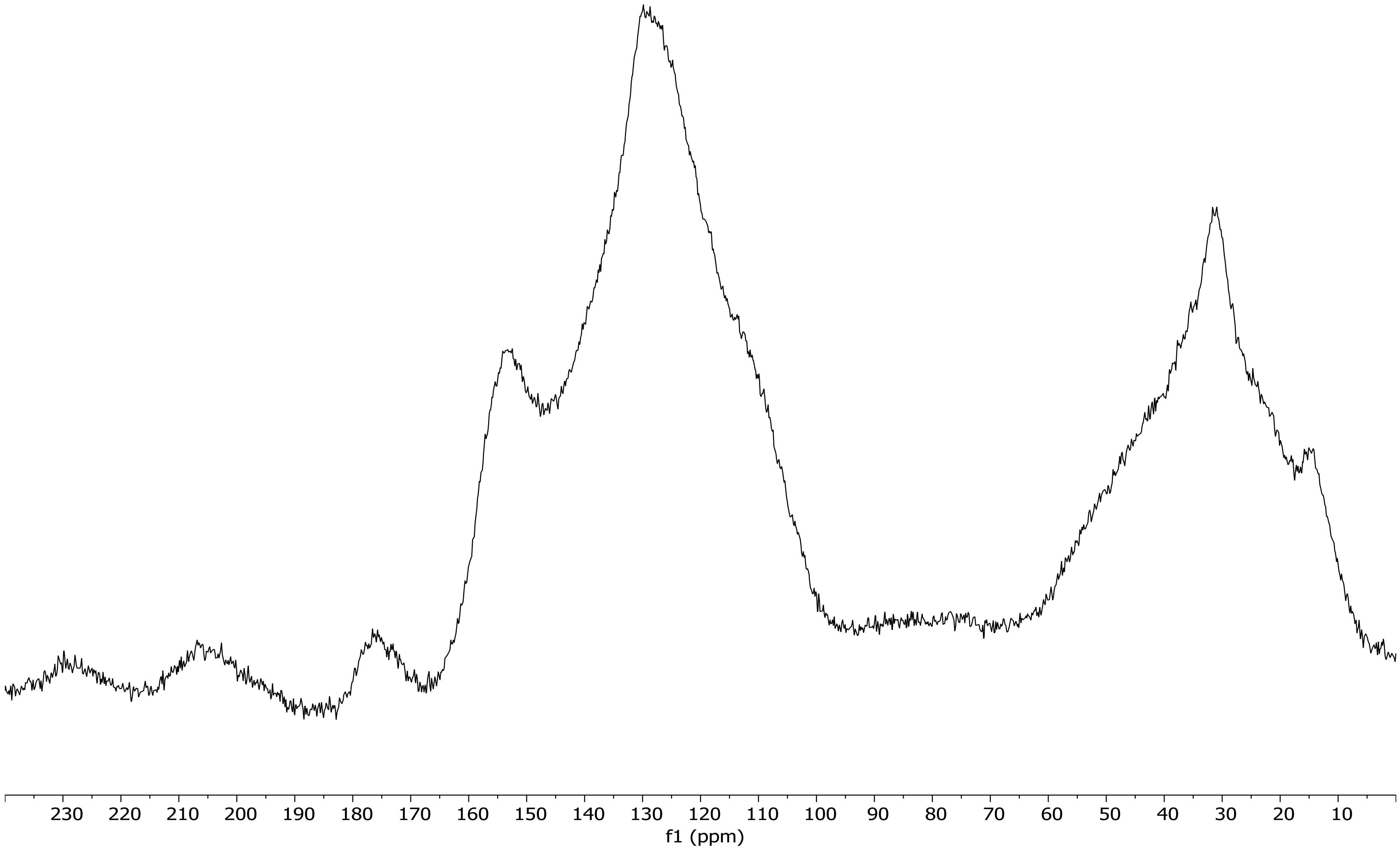

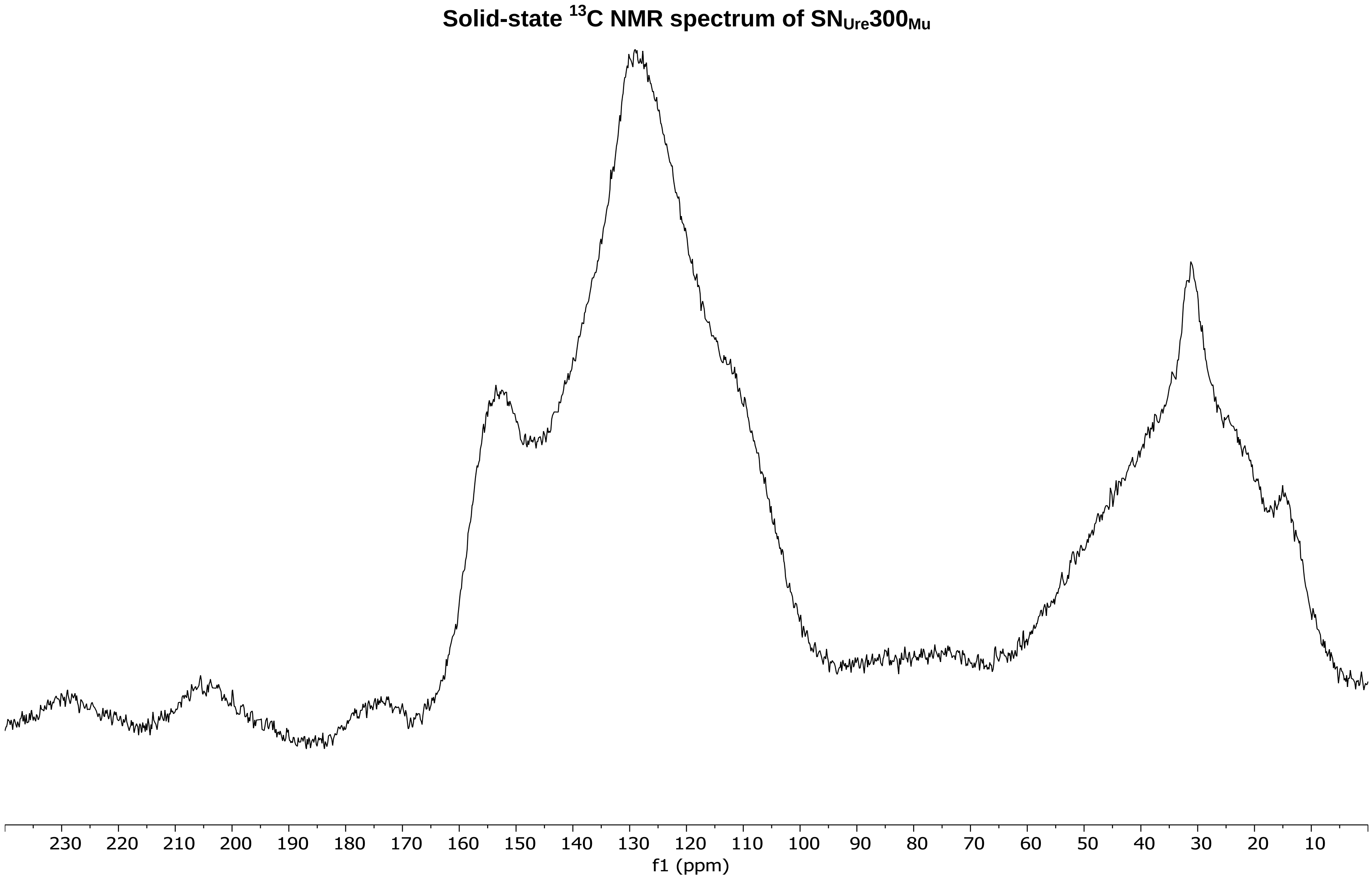
Solid-state ^{13}C NMR spectrum of $SN_{Ure}300_{Mu}$
230
220
210
200
190
180
170
160
150
140
130
120
110
100
90
80
70
60
50
40
30
20
10
f1 (ppm)

Solid-state ^{13}C NMR spectrum of $SN_{Mel}300_{Mu}$

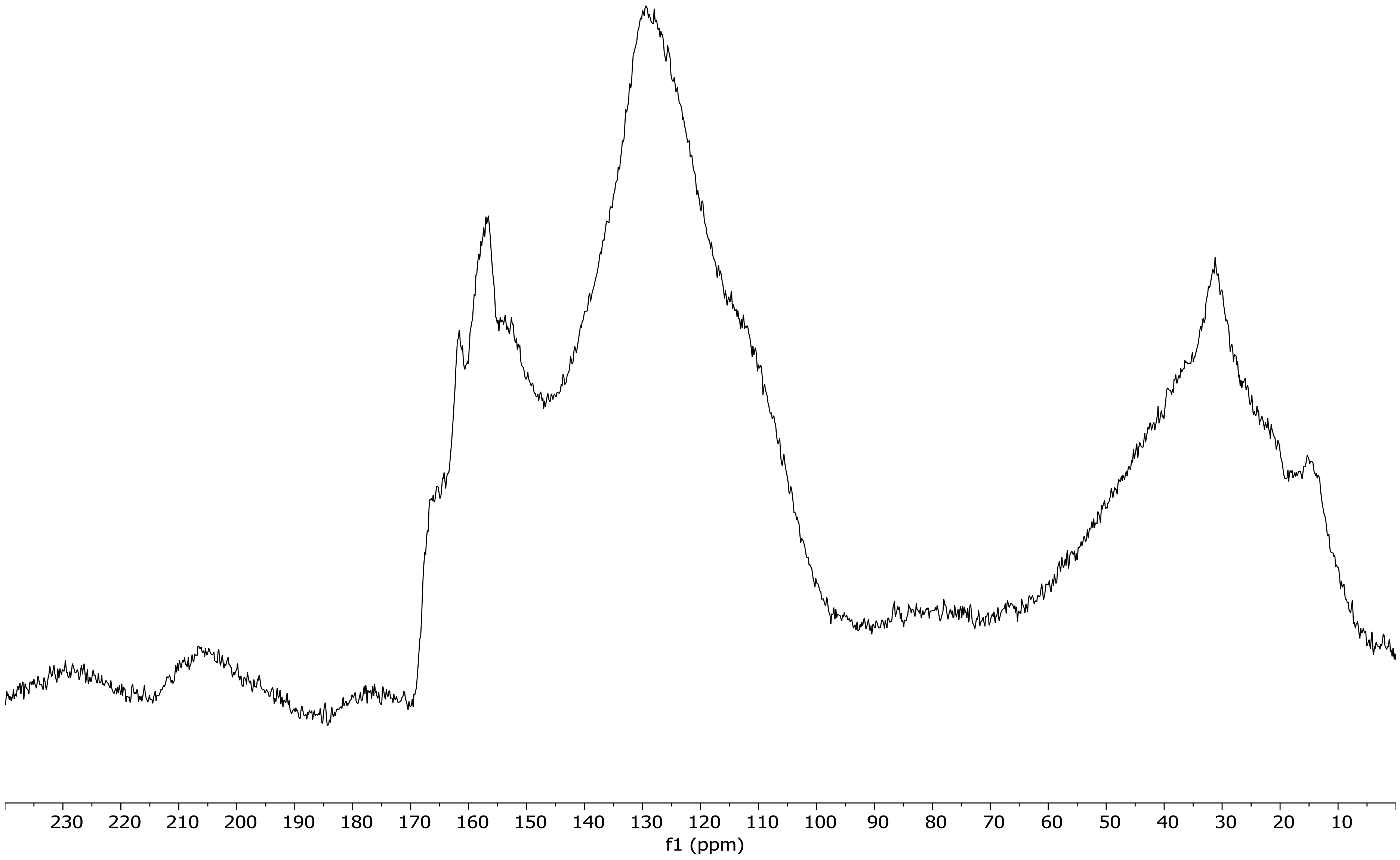

Solid-state ^{13}C NMR spectrum of $SN_{Nic}300_{Mu}$

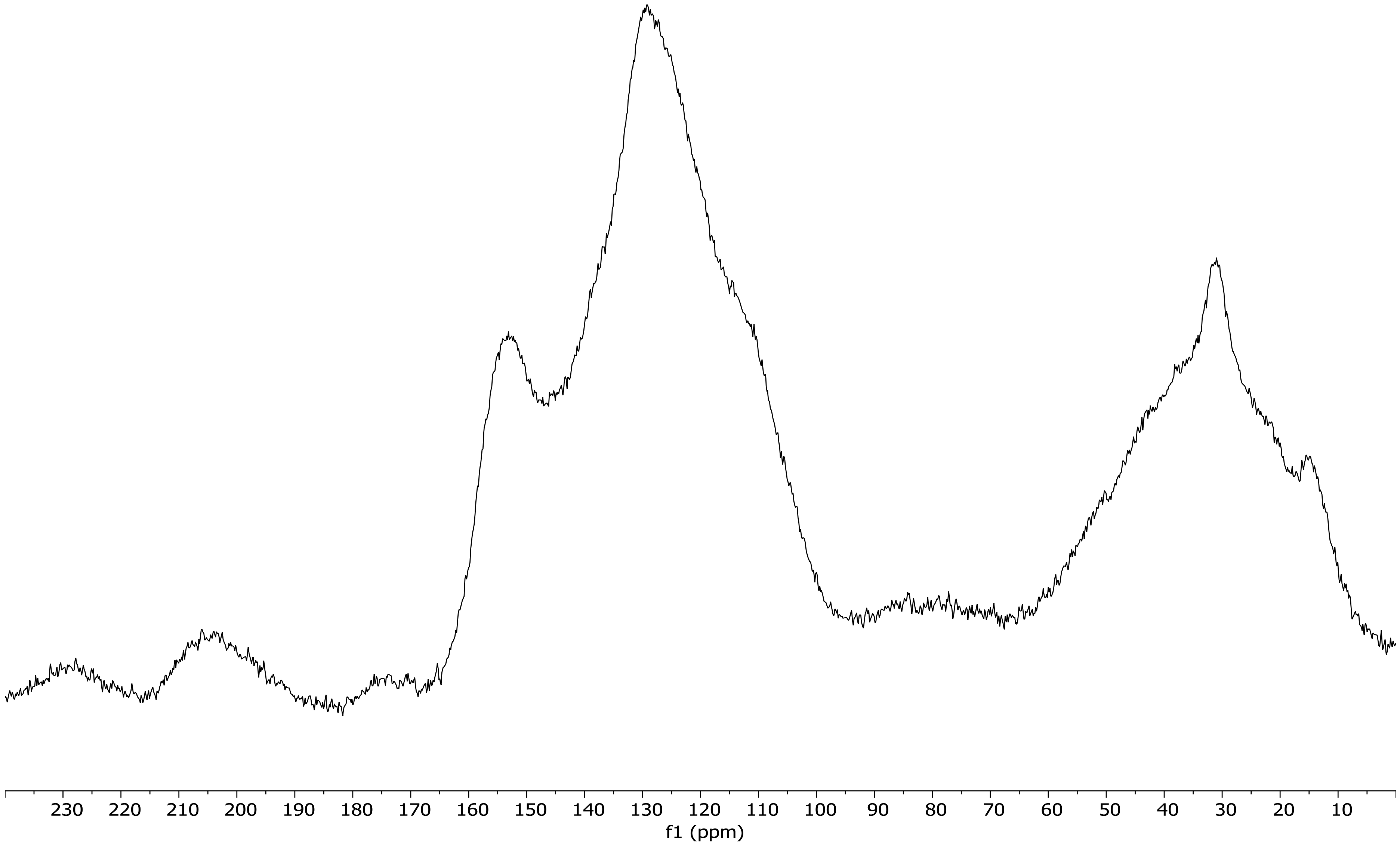

SEM images of S300 and S800

S300

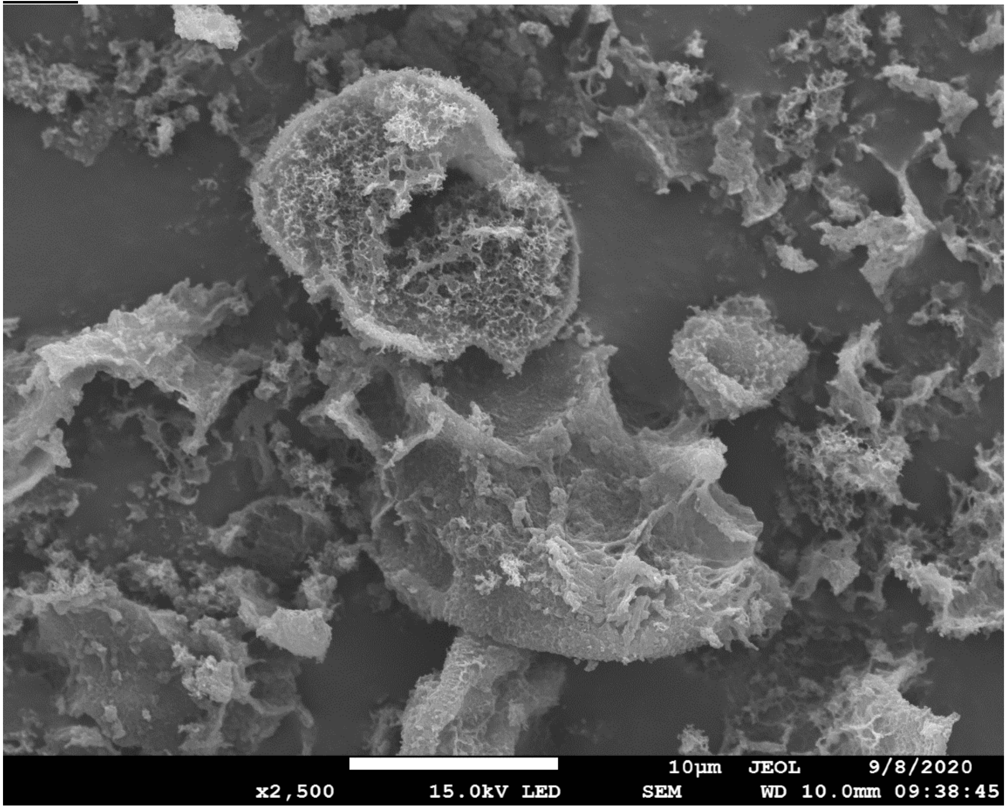

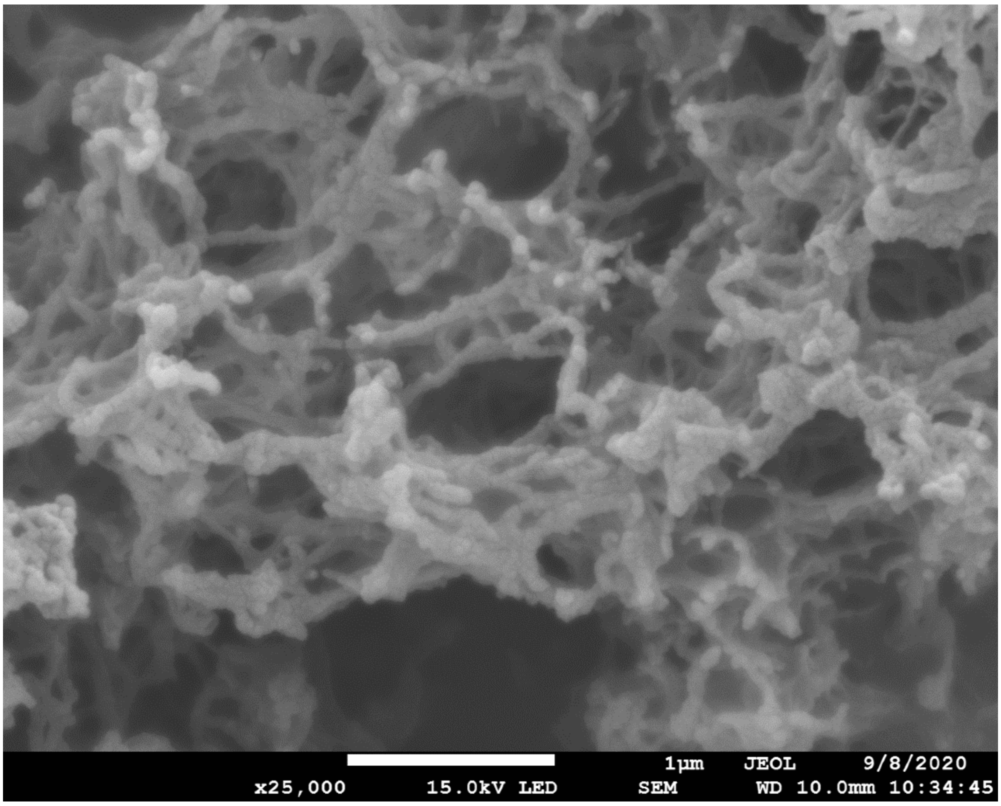

S800

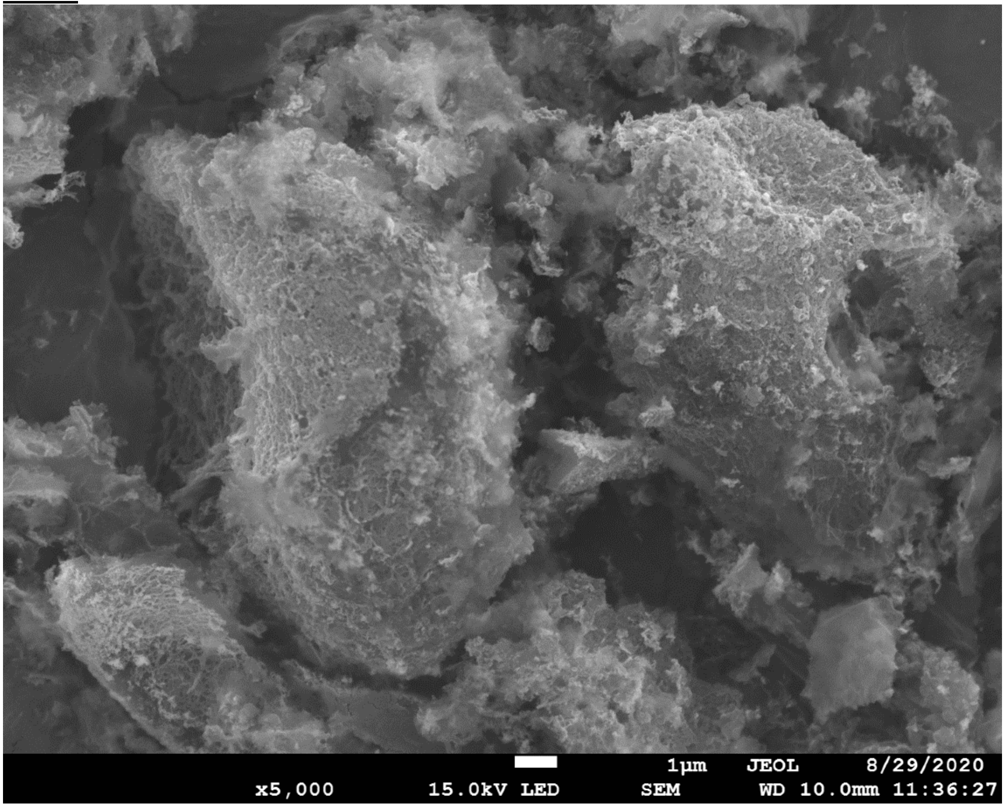
1µm JEOL 8/29/2020
x5,000 15.0kV LED SEM WD 10.0mm 11:36:27

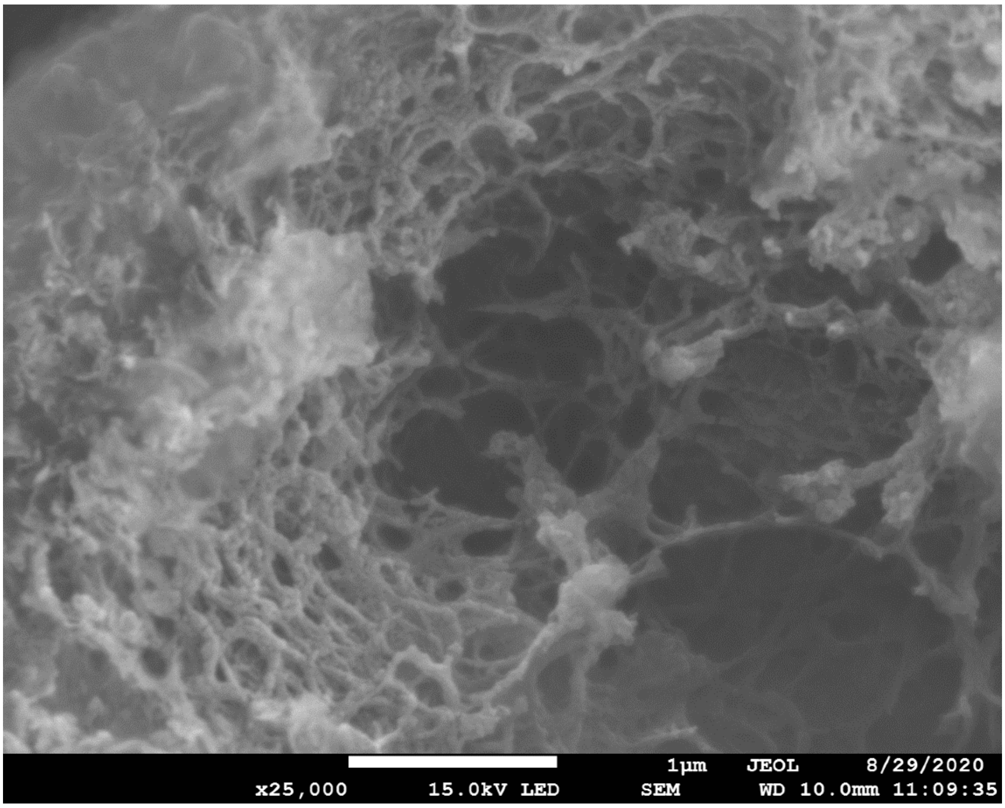
1µm JEOL 8/29/2020
x25,000 15.0kV LED SEM WD 10.0mm 11:09:35

SEM image and nitrogen EDX mapping of $SN_{Gly}300_{Th}$

<u>SEM</u>

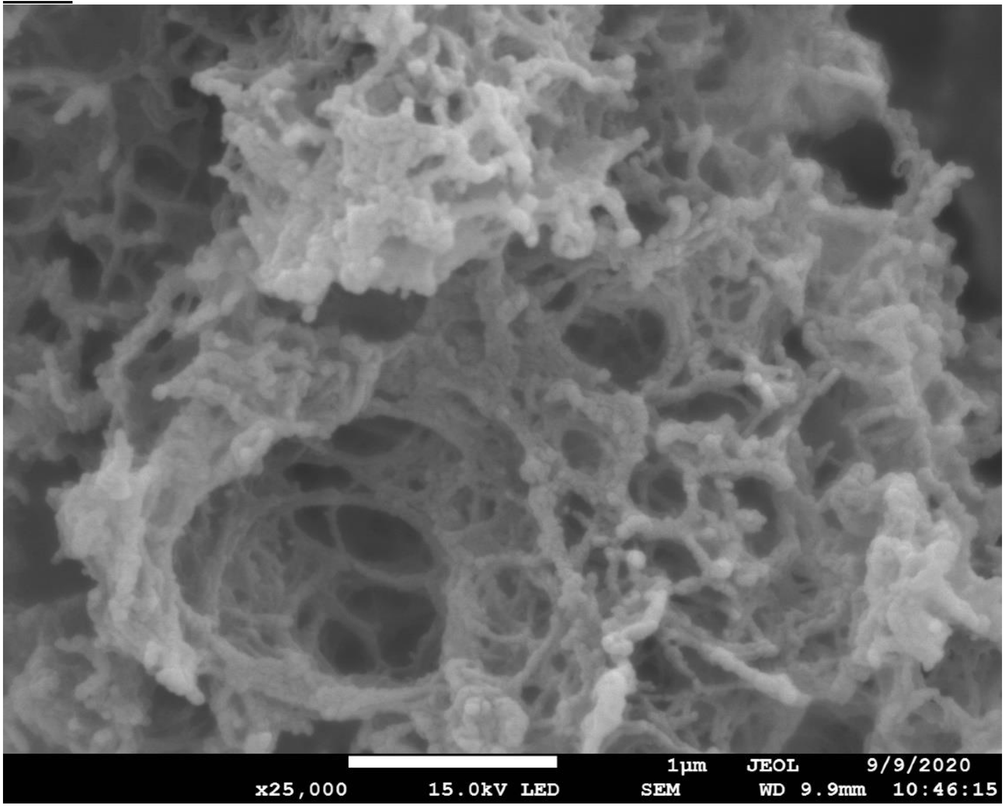

<u>EDX</u>

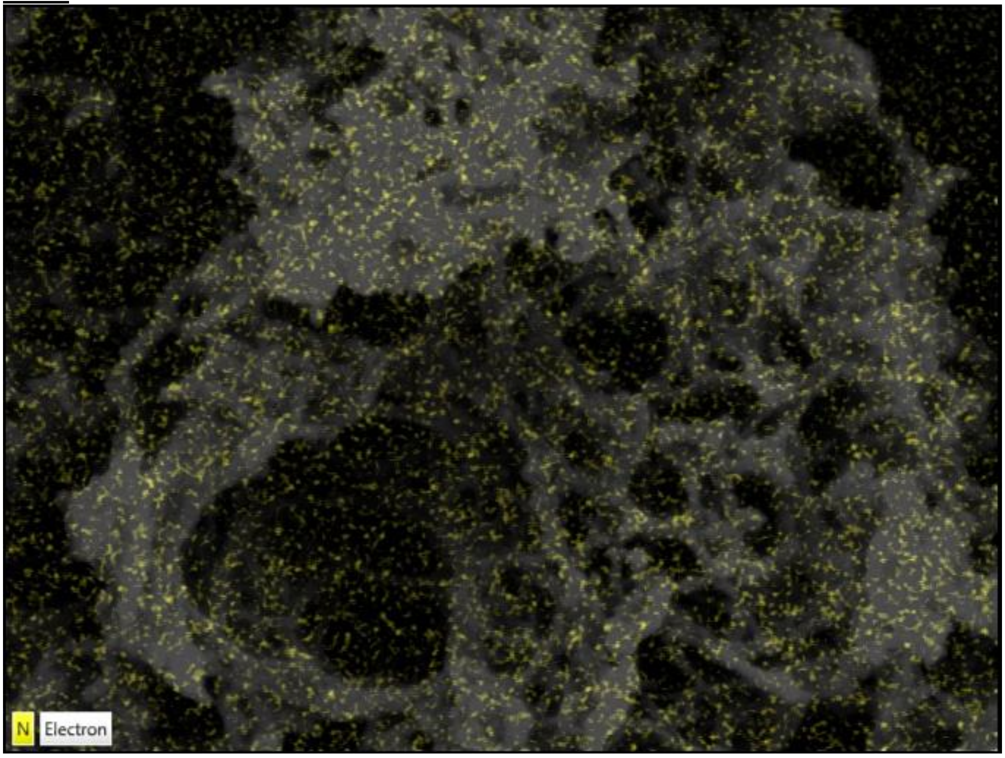

SEM image and nitrogen EDX mapping of $SN_{Bal}300_{Th}$

SEM

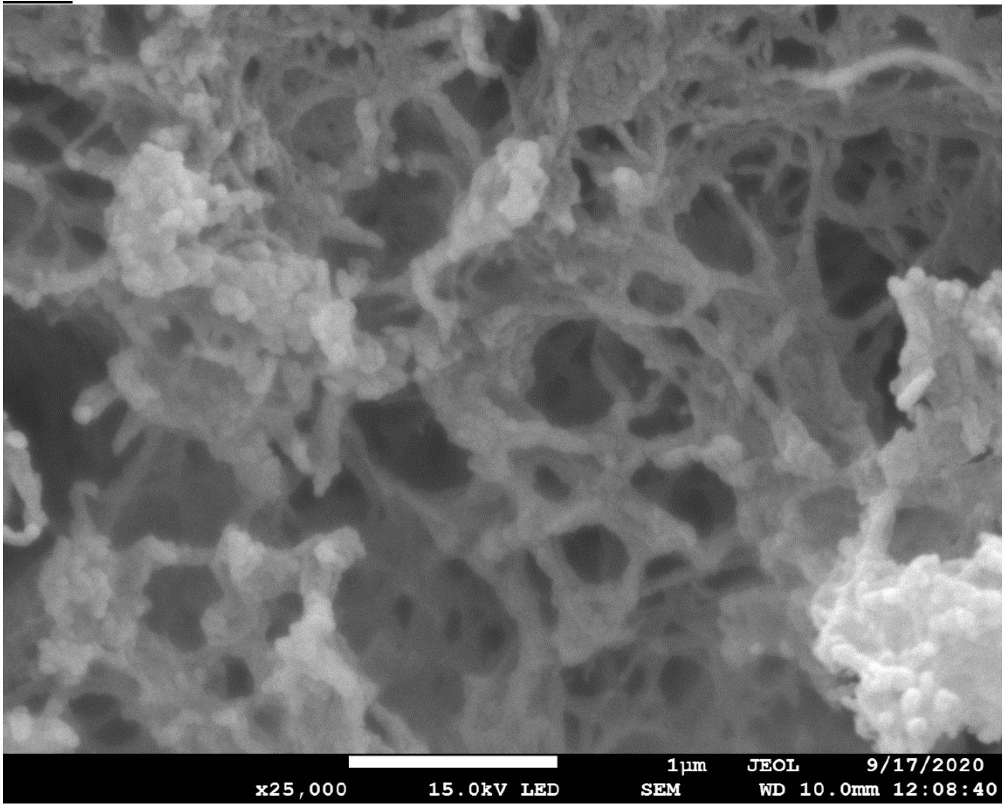

EDX

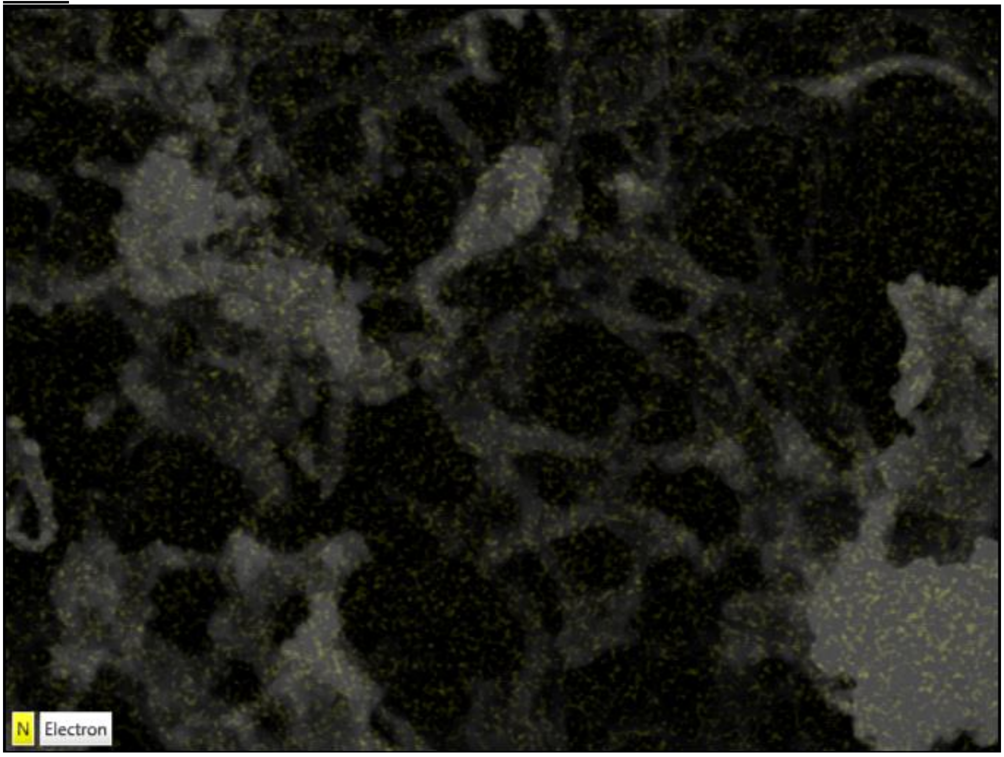

SEM image and nitrogen EDX mapping of $SN_{Ure}300_{Th}$

<u>SEM</u>

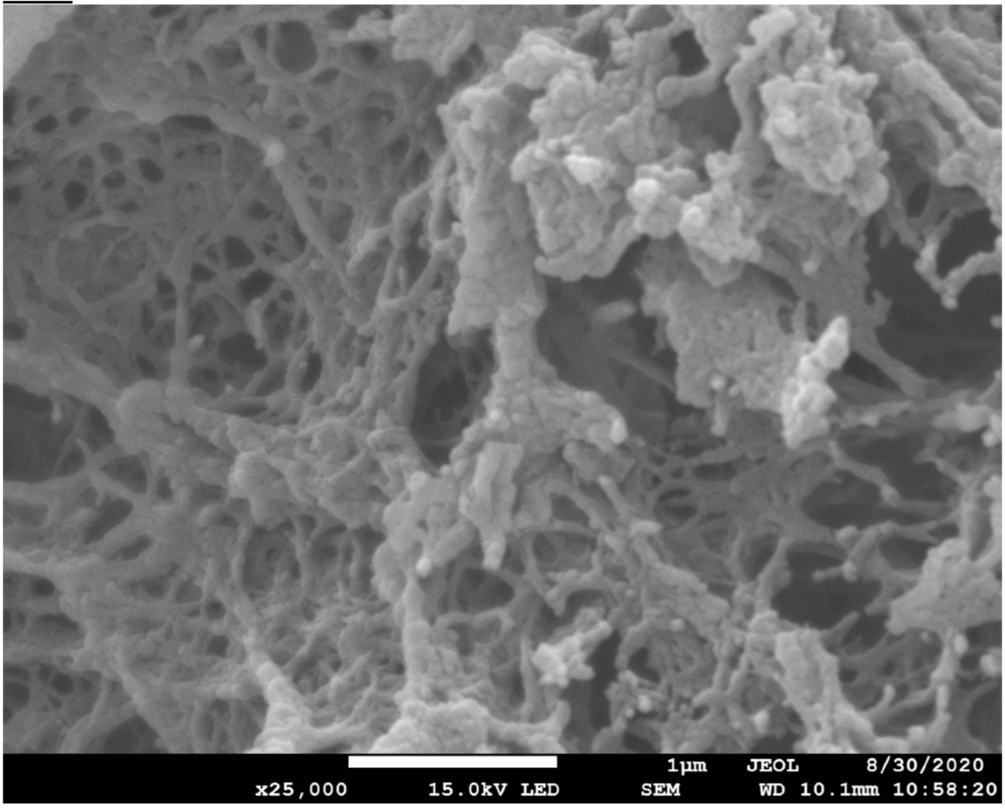

<u>EDX</u>

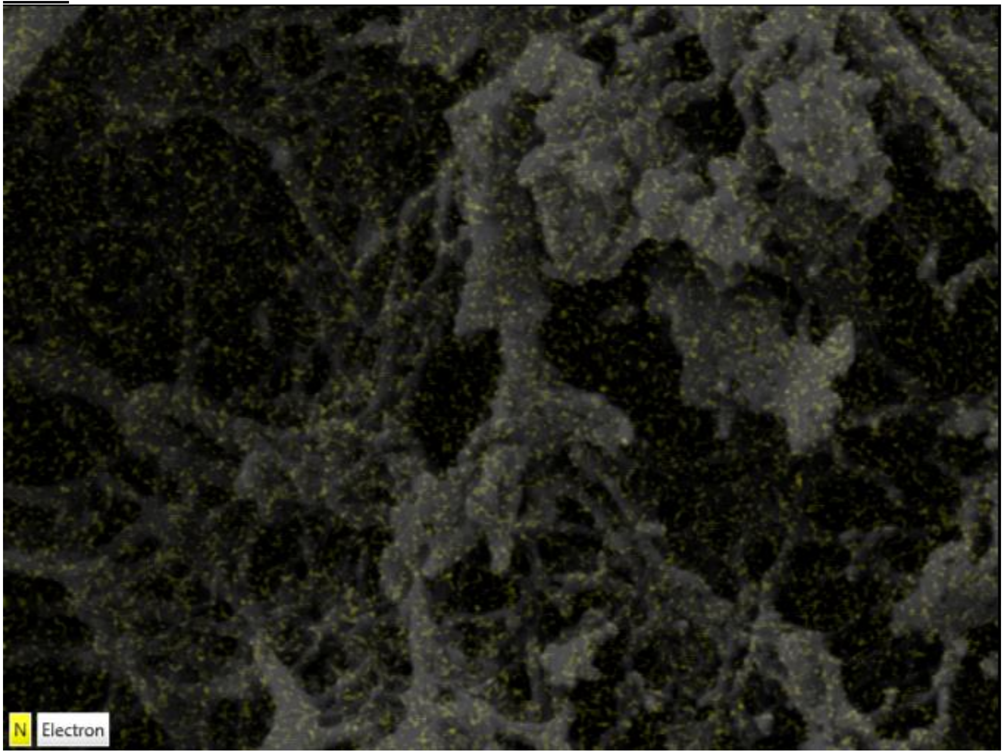

SEM image and nitrogen EDX mapping of $SN_{Mel}300_{Th}$

SEM

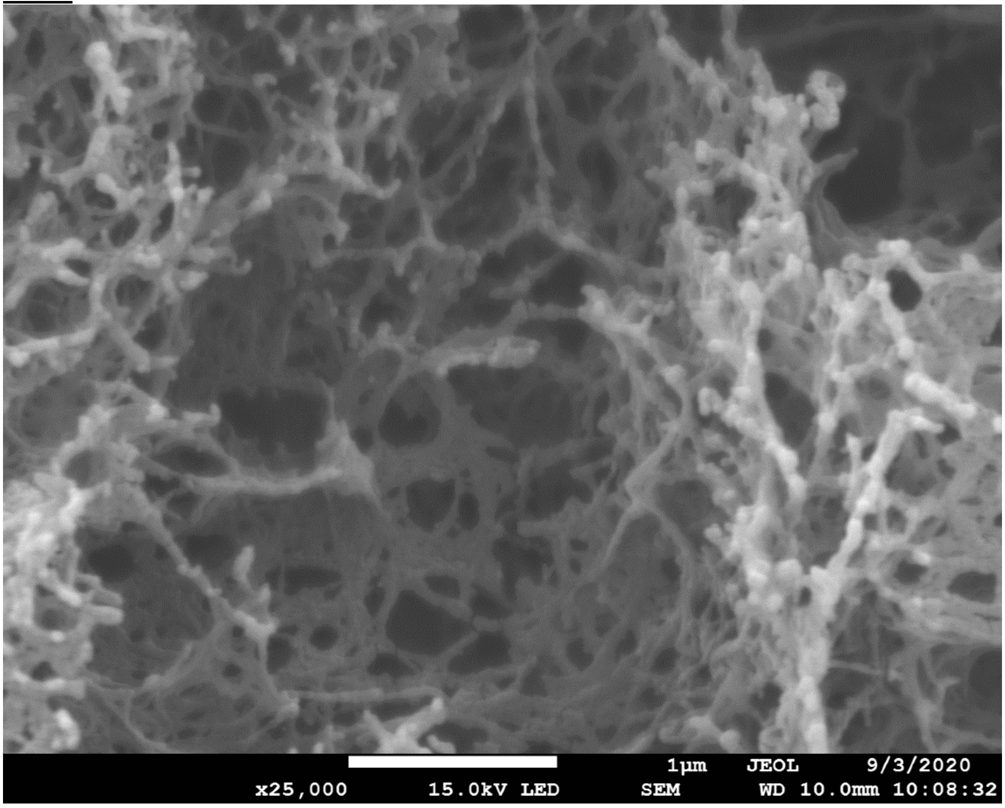

EDX

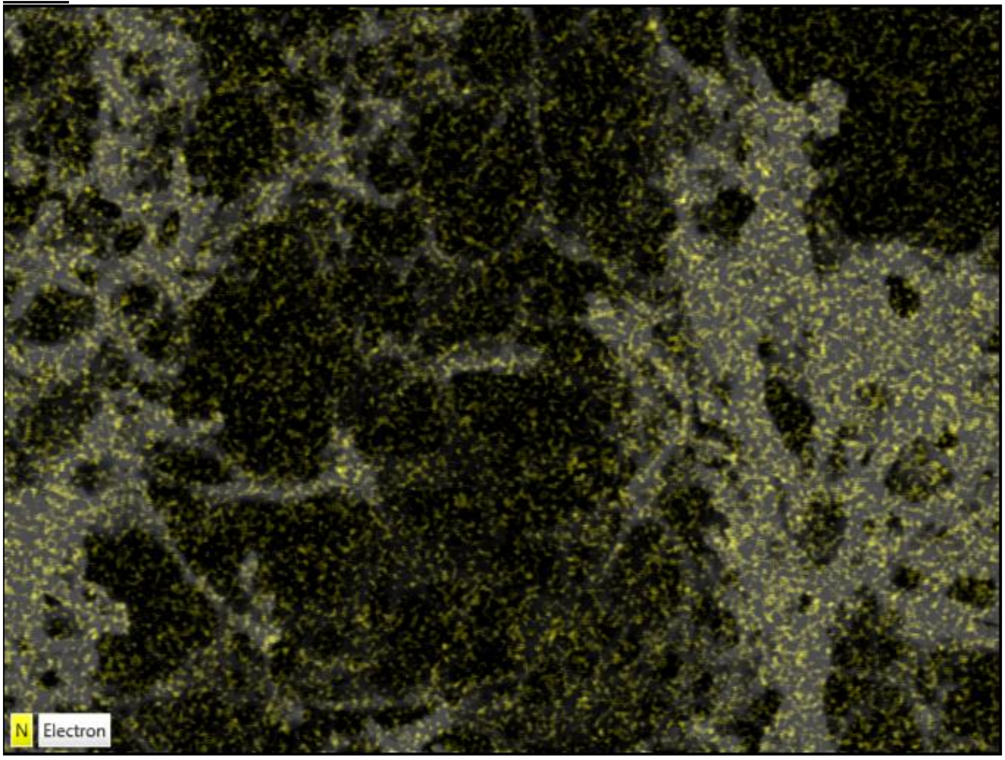

SEM image and nitrogen EDX mapping of $SN_{Nic}300_{Th}$

SEM

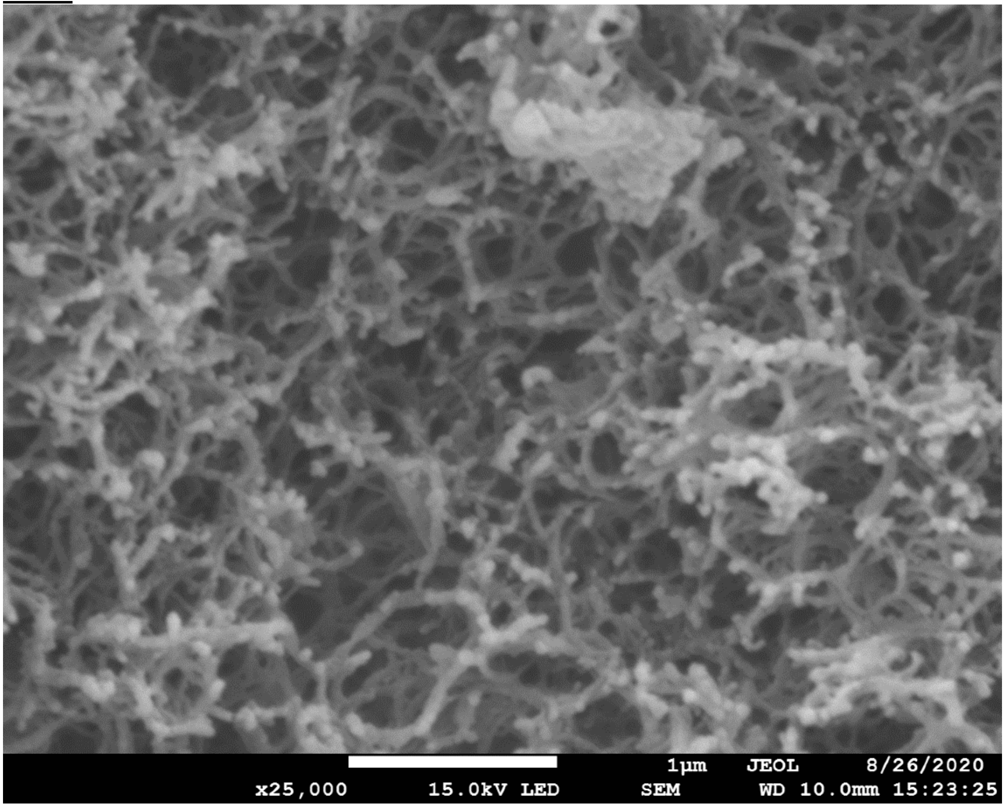

EDX

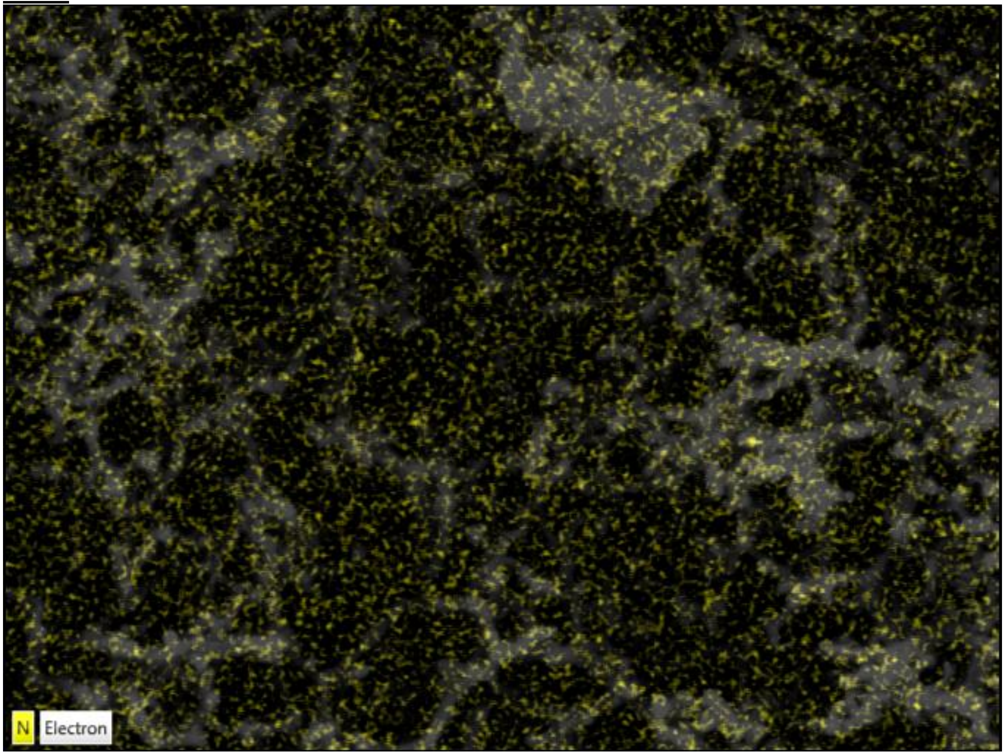

SEM image and nitrogen EDX mapping of $SN_{Gly}800_{Th}$

SEM

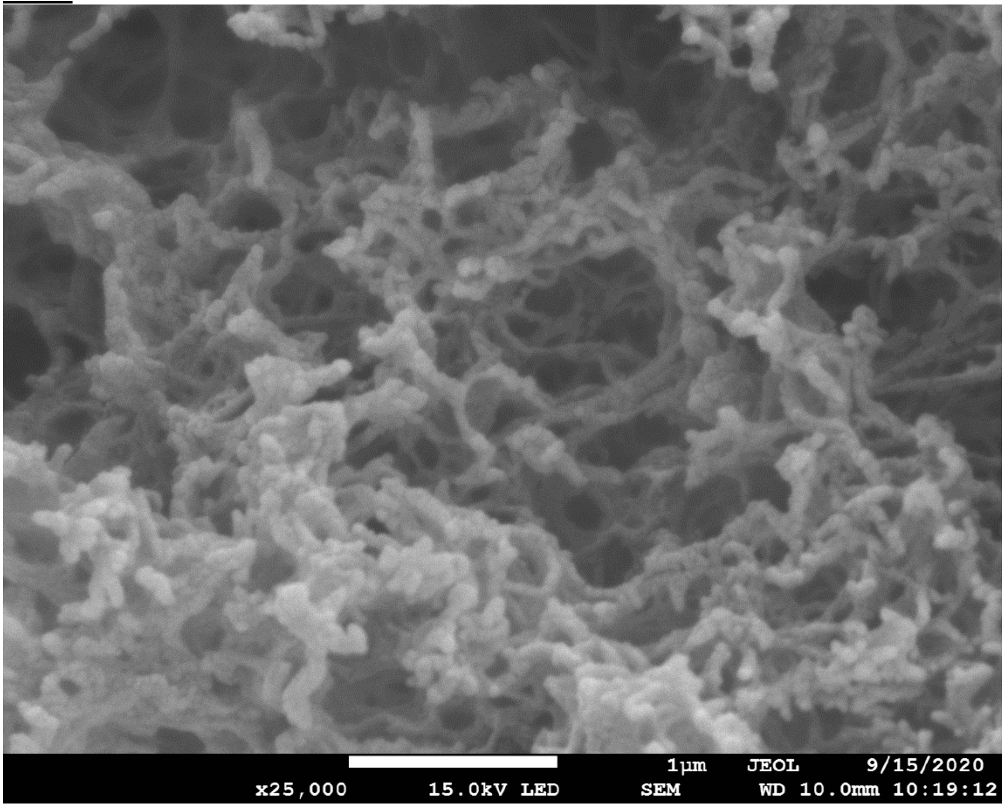

EDX

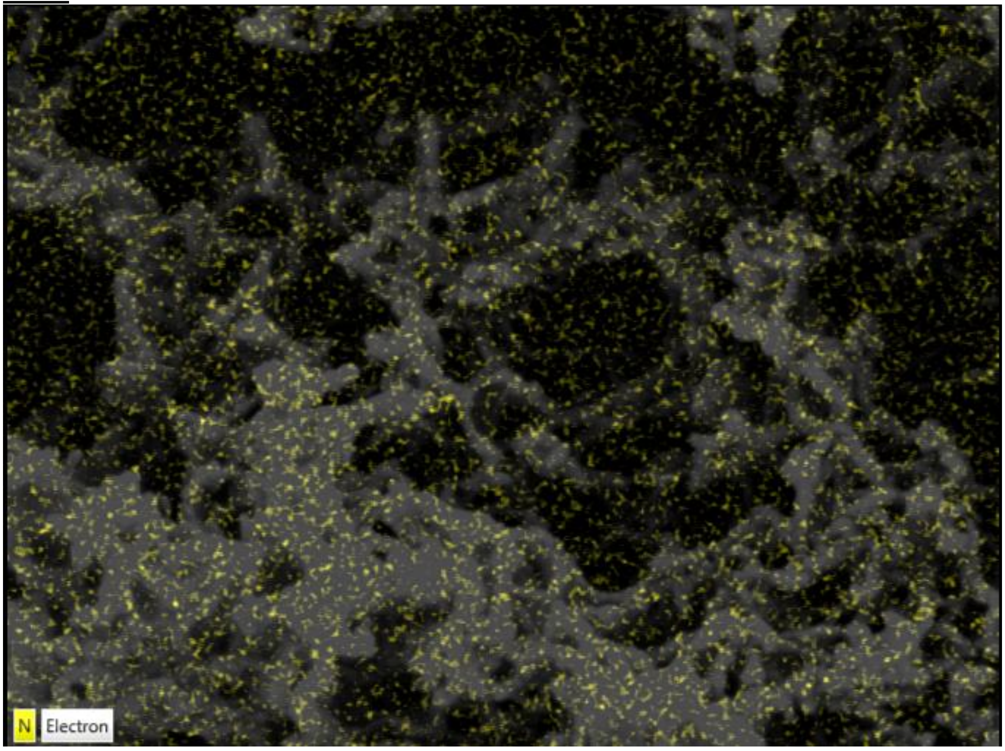

SEM image and nitrogen EDX mapping of $SN_{Bal}800_{Th}$

SEM

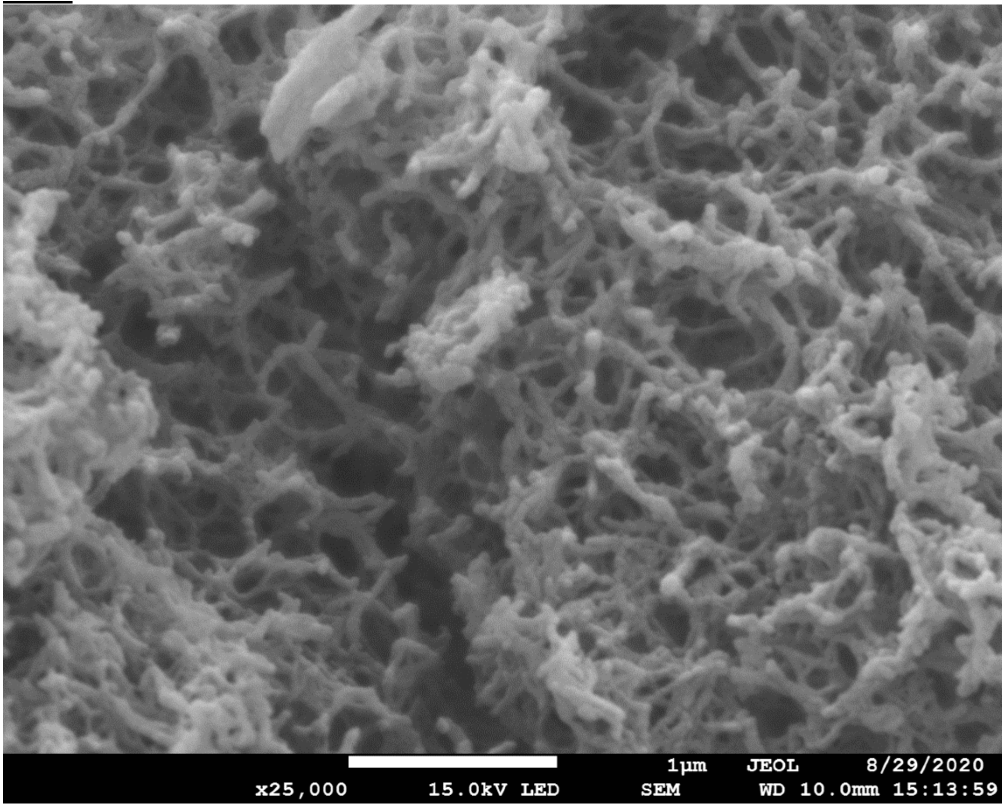

EDX

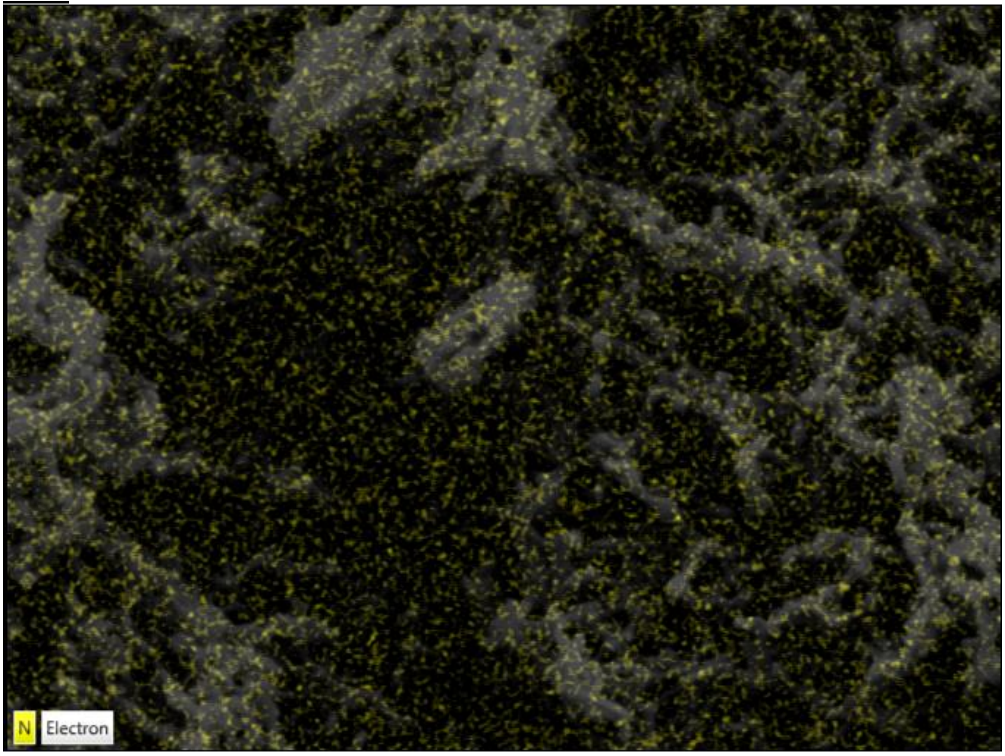

SEM image and nitrogen EDX mapping of $SN_{Ure}800_{Th}$

SEM

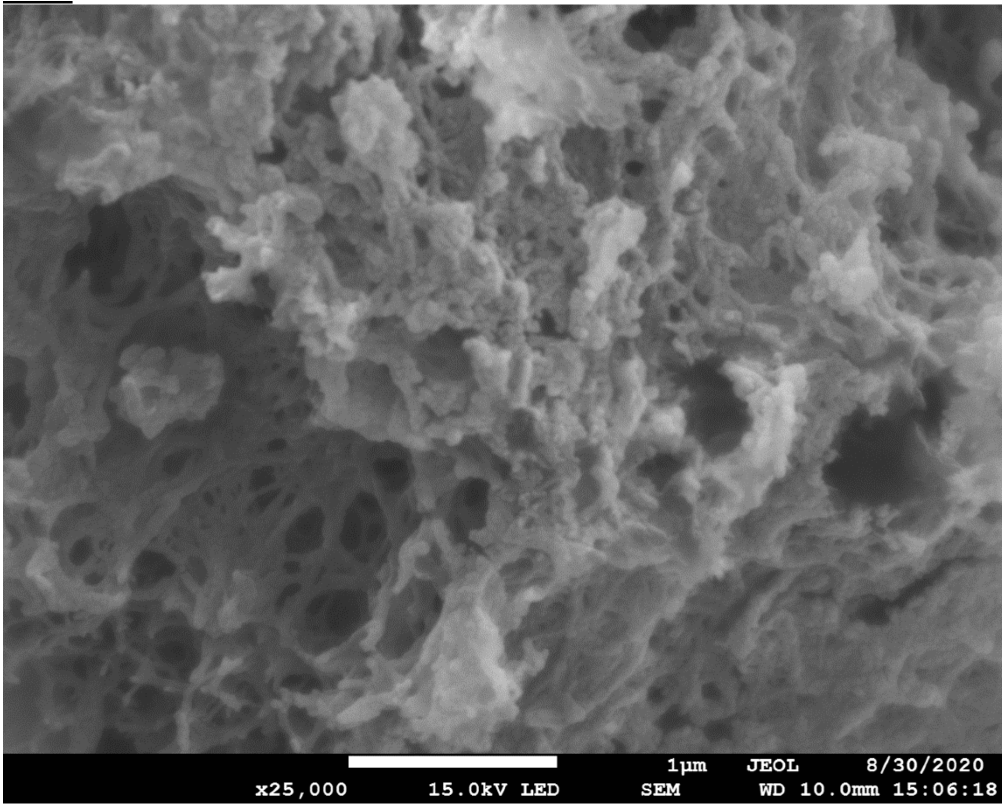

EDX

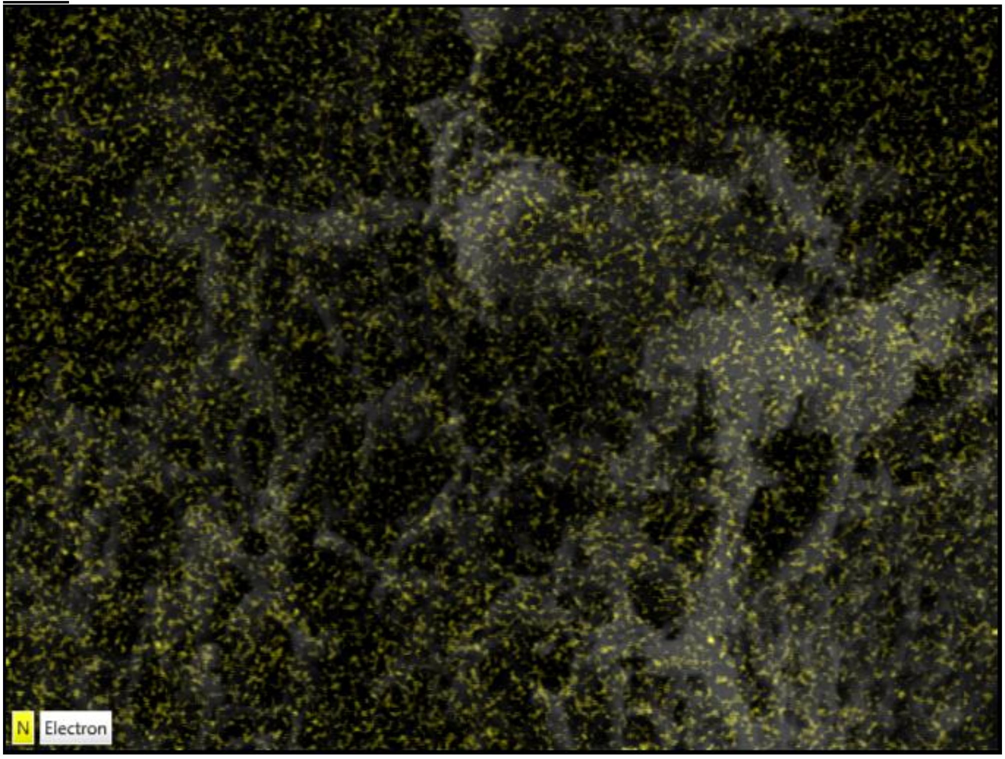

SEM image and nitrogen EDX mapping of $SN_{Mel}800_{Th}$

SEM

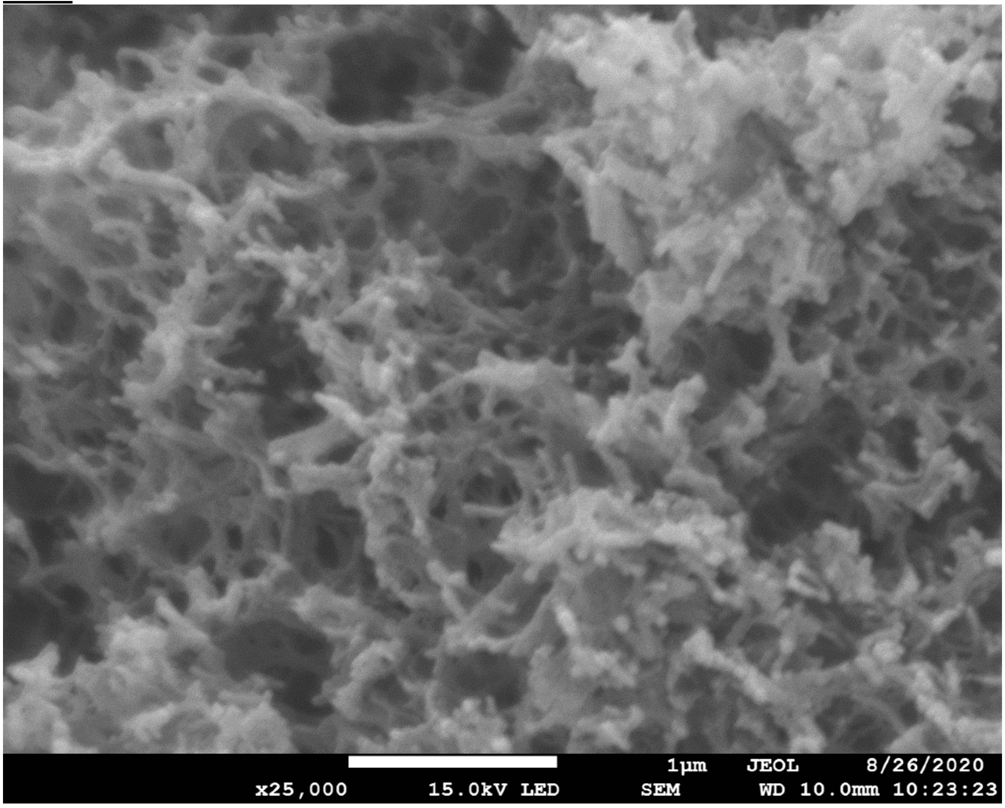

EDX

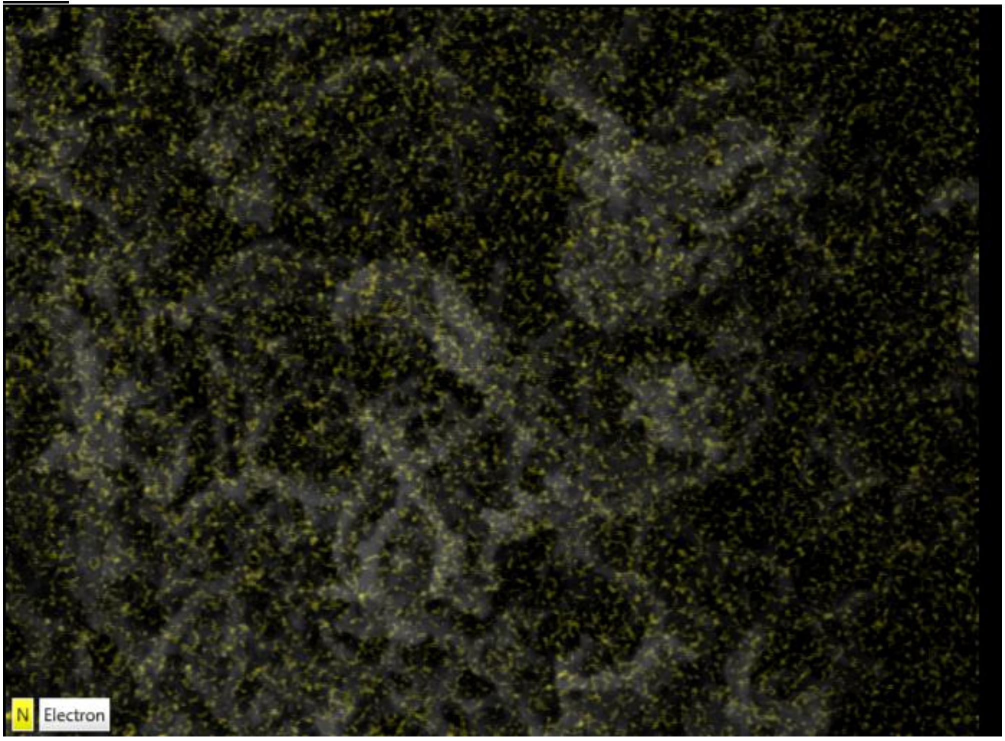

SEM image and nitrogen EDX mapping of $SN_{Nic}800_{Th}$

SEM

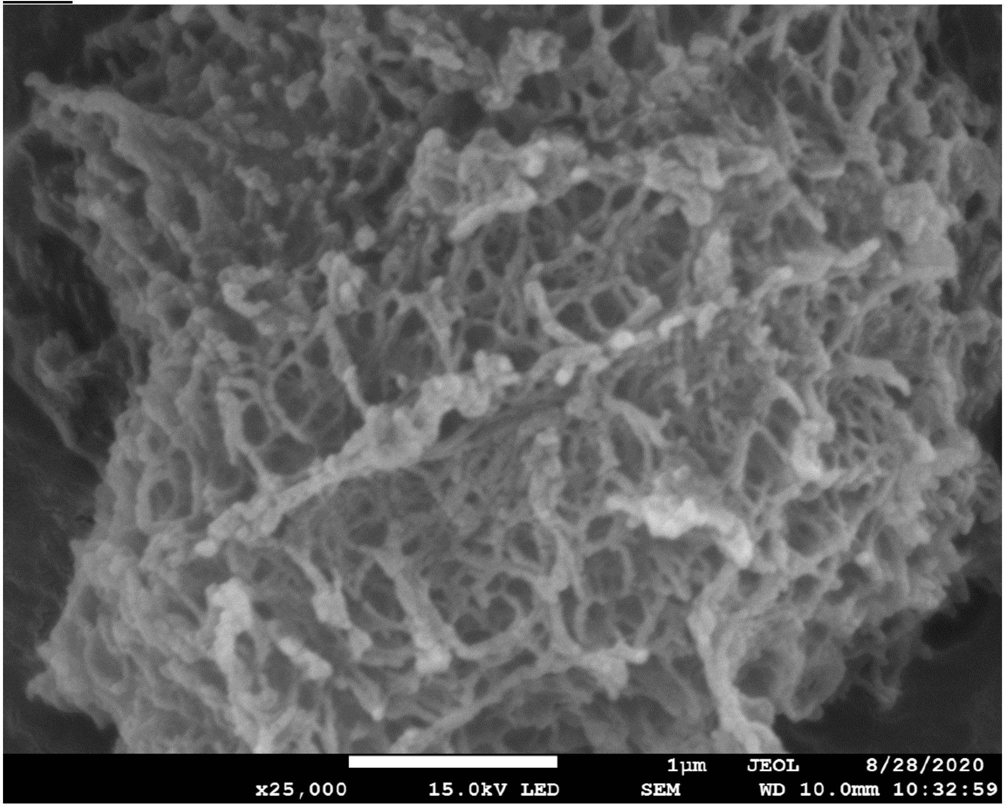

EDX

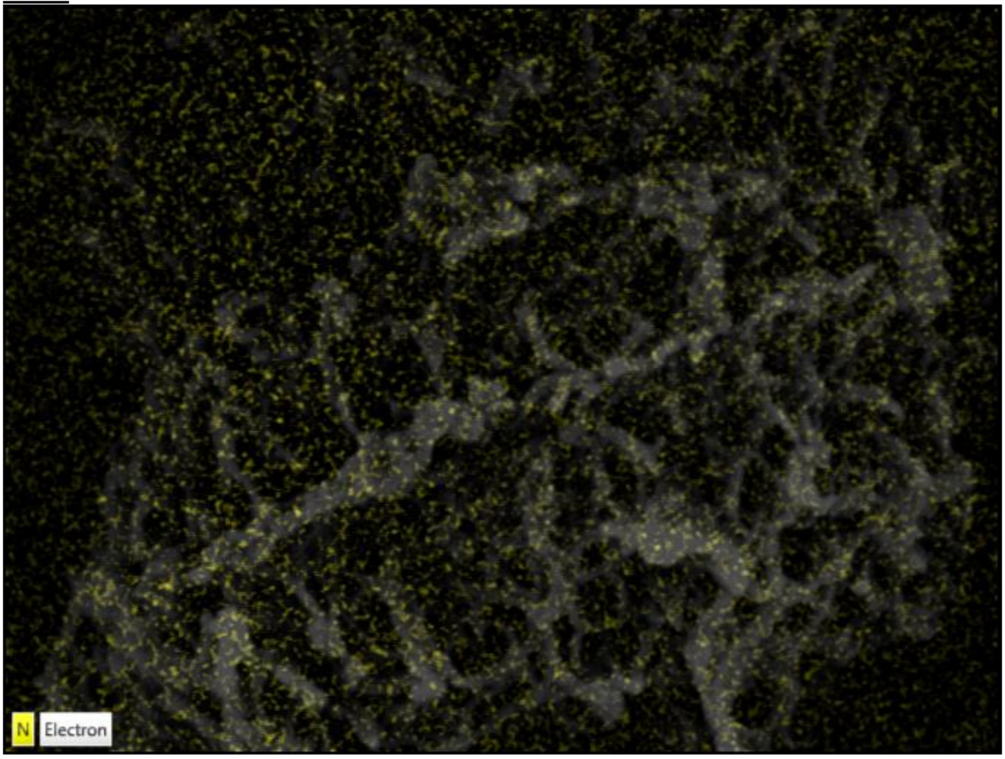

SEM image and nitrogen EDX mapping of $SN_{Gly}300_{Mo}$

SEM

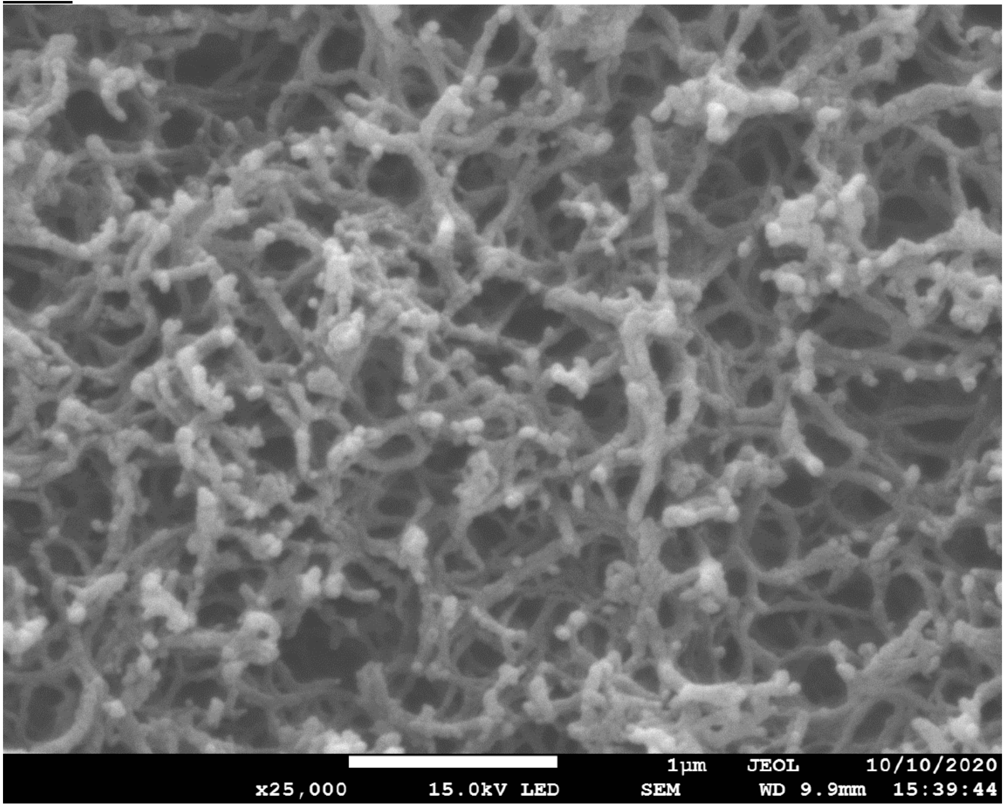

EDX

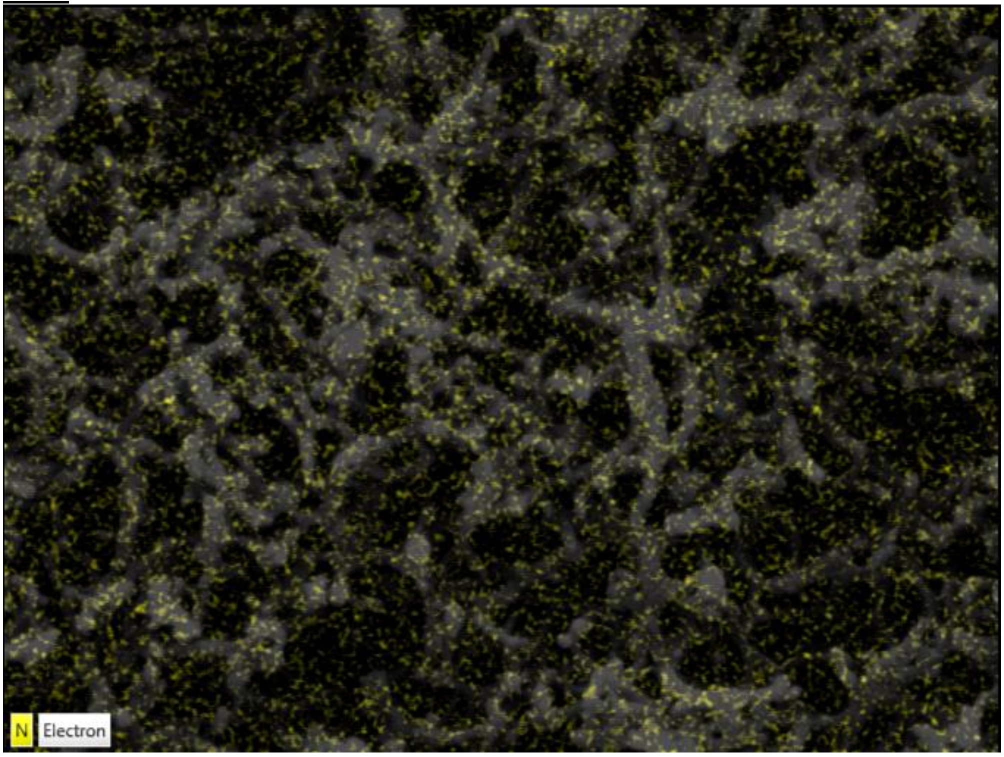

SEM image and nitrogen EDX mapping of $SN_{Bal}300_{Mo}$

SEM

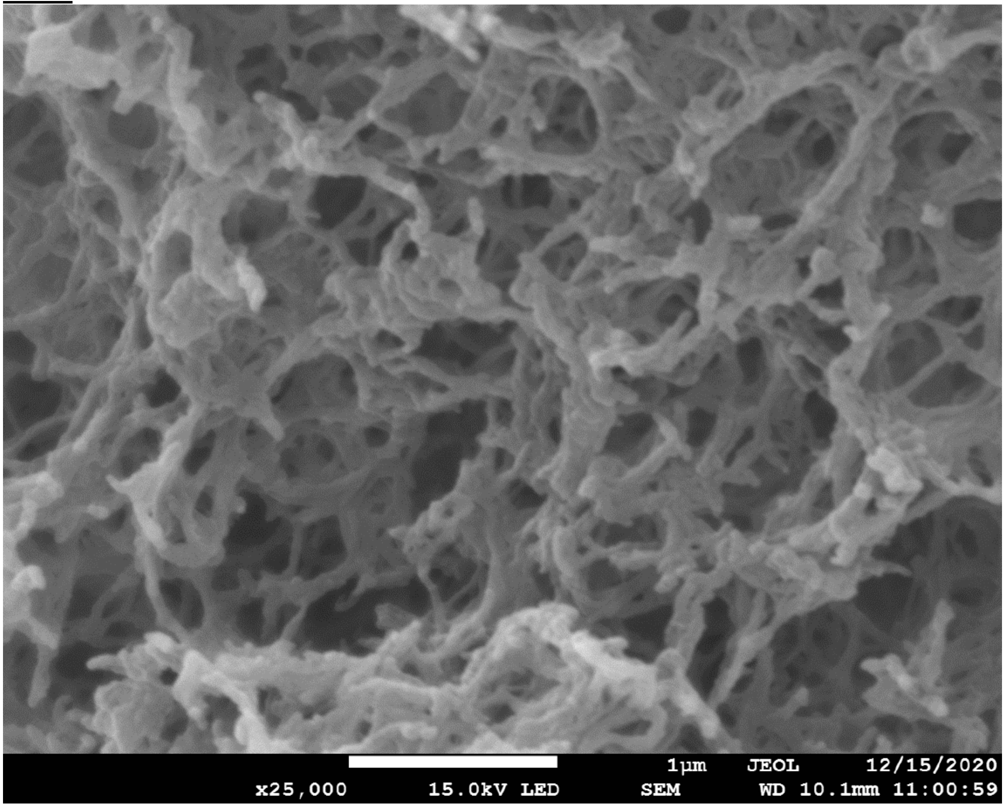

EDX

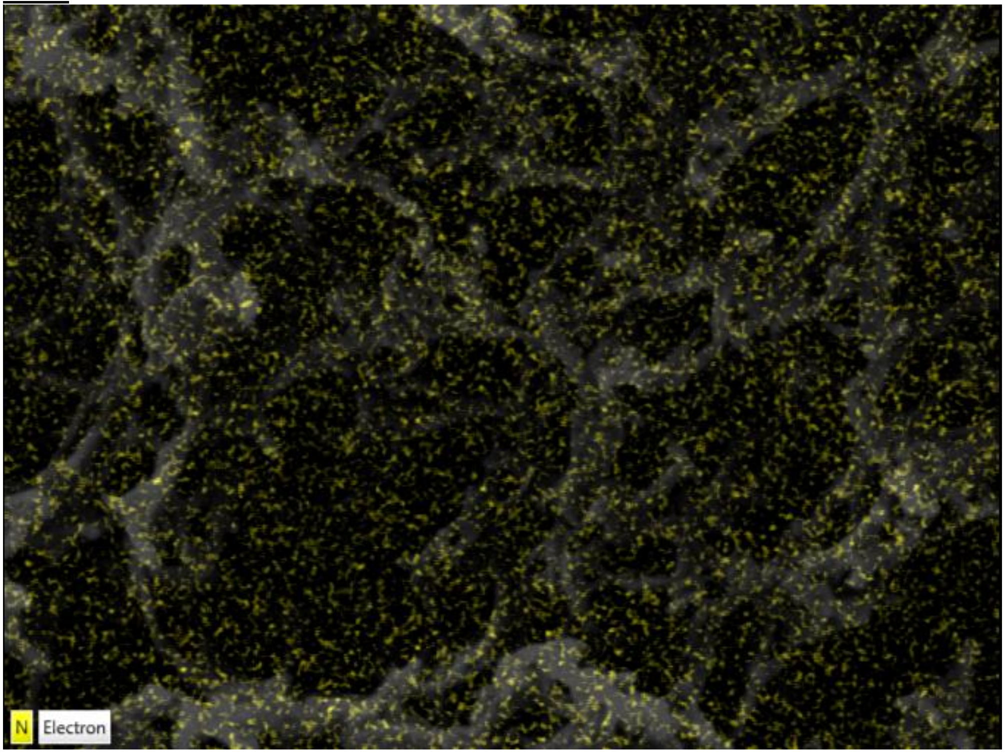

SEM image and nitrogen EDX mapping of $SN_{Ure}300_{Mo}$

SEM

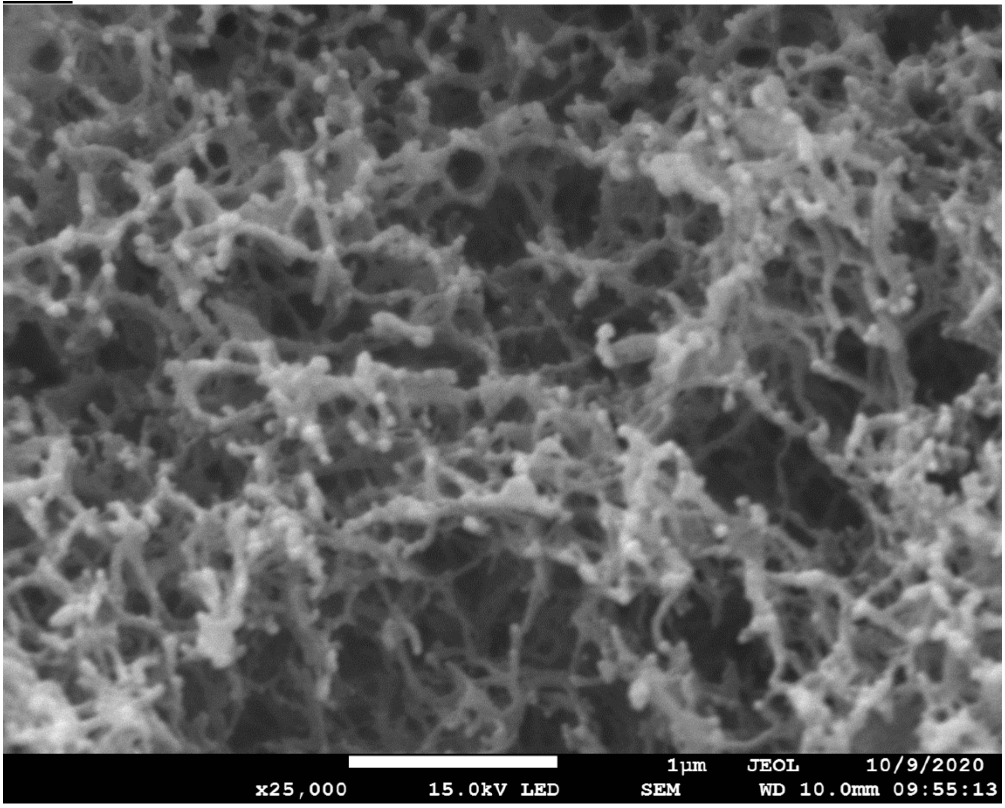

EDX

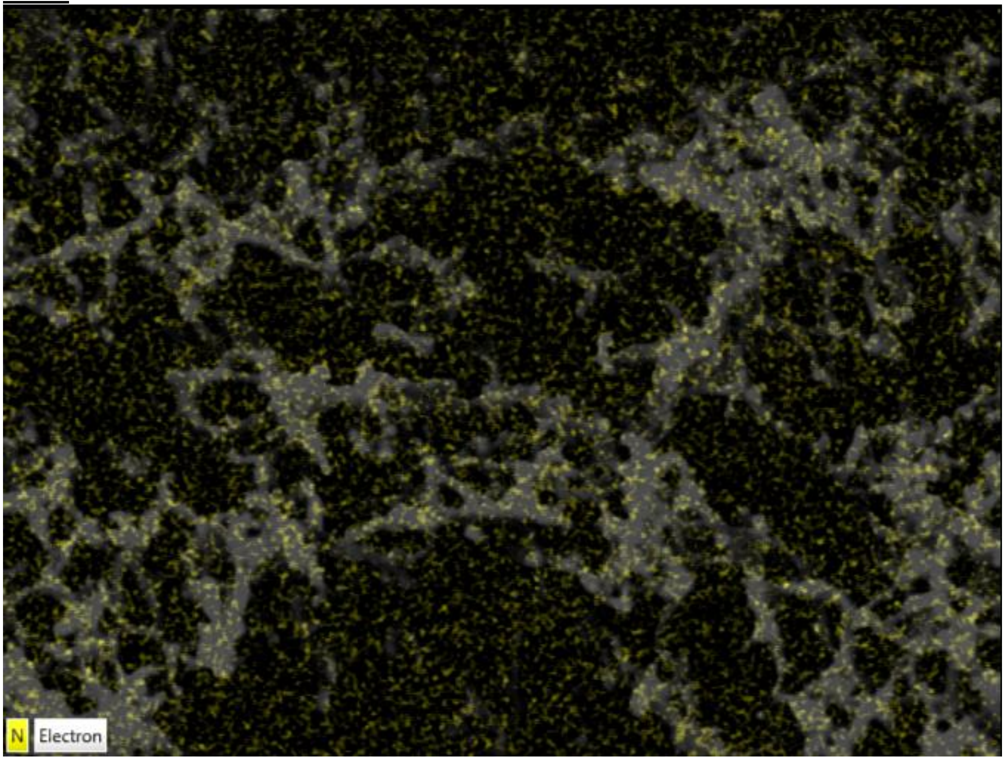

SEM image and nitrogen EDX mapping of $SN_{Mel}300_{Mo}$

SEM

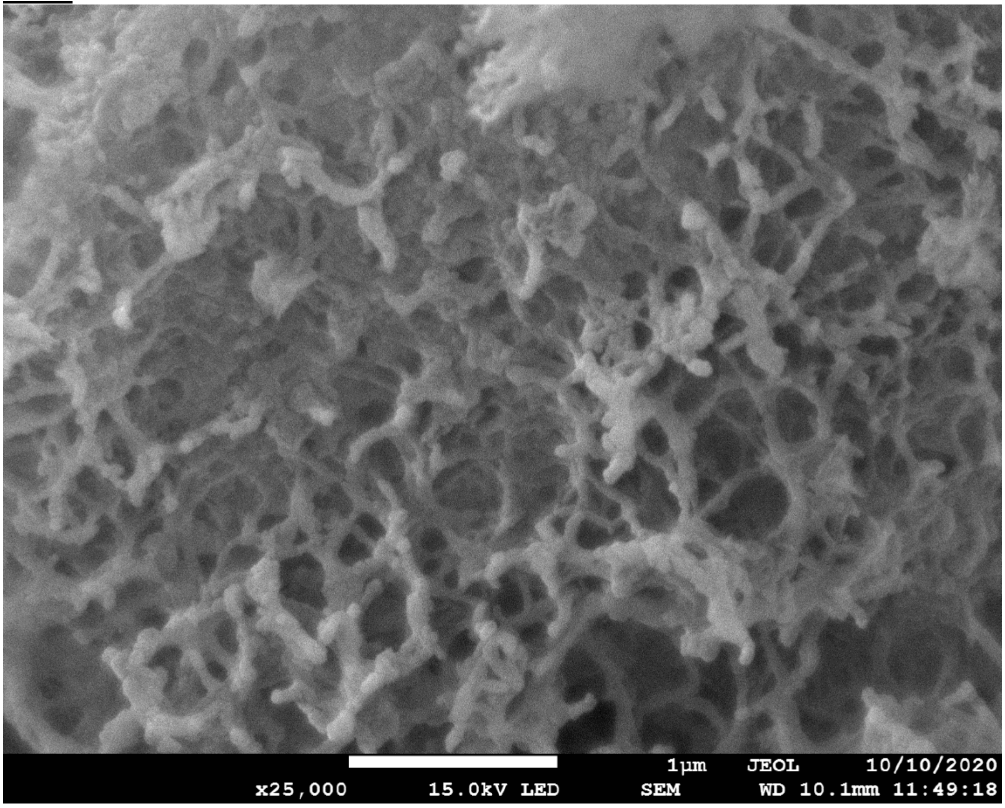

EDX

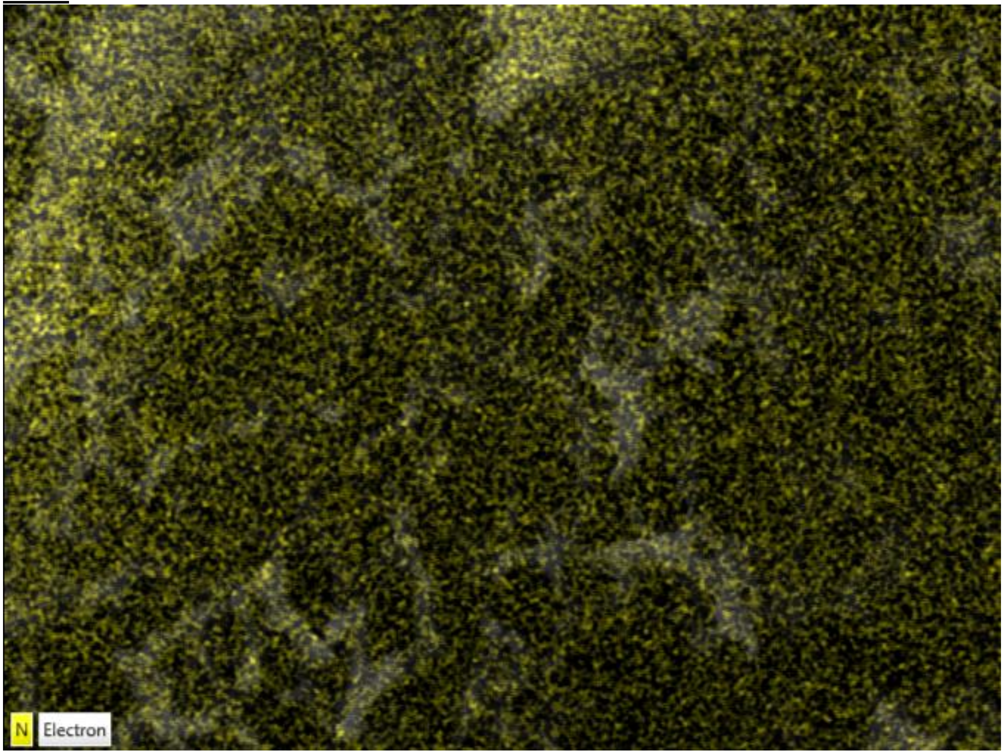

SEM image and nitrogen EDX mapping of $SN_{Nic}300_{Mo}$

<u>SEM</u>

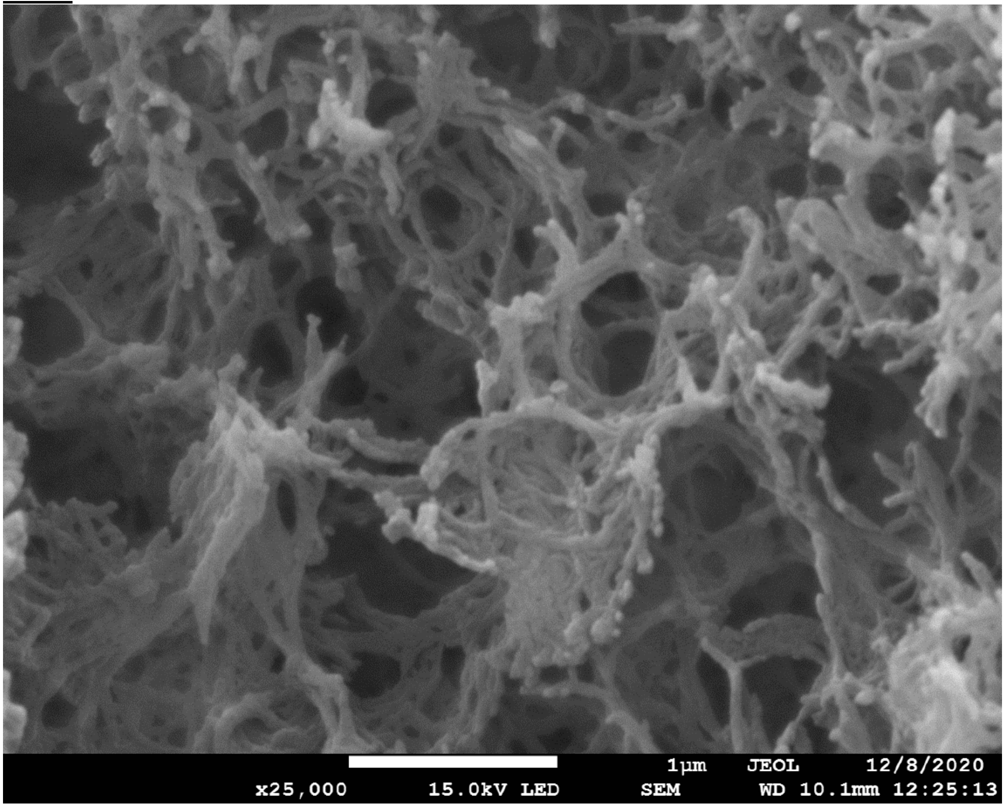

<u>EDX</u>

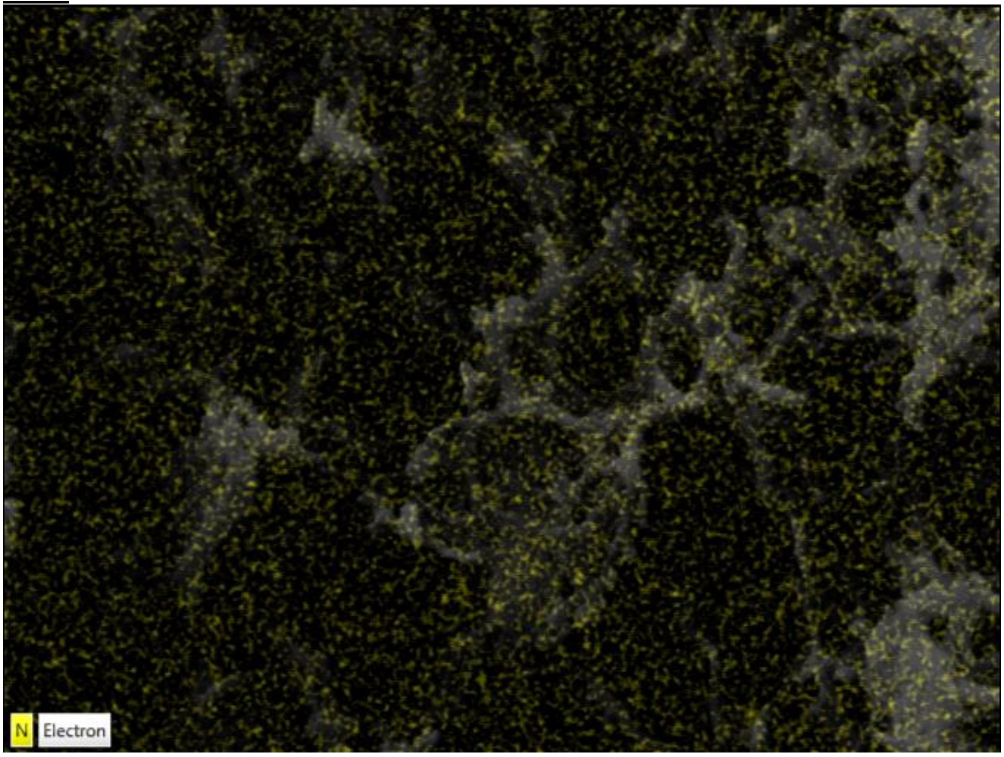

SEM image and nitrogen EDX mapping of $SN_{Gly}300_{Mu}$

SEM

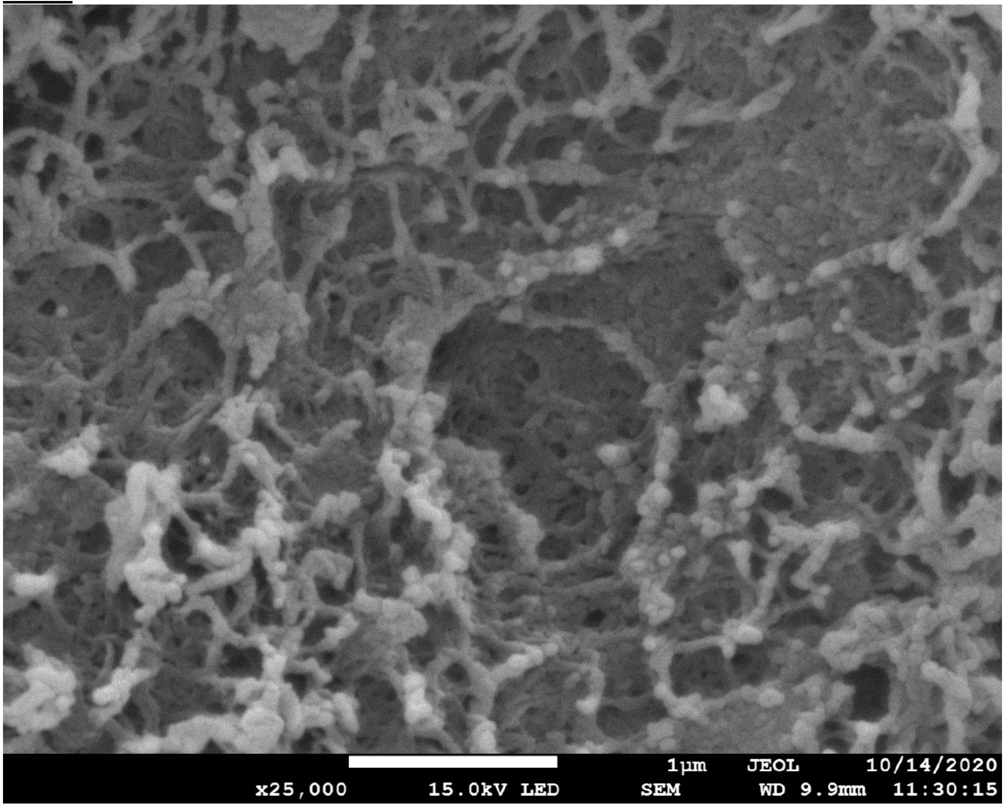

EDX

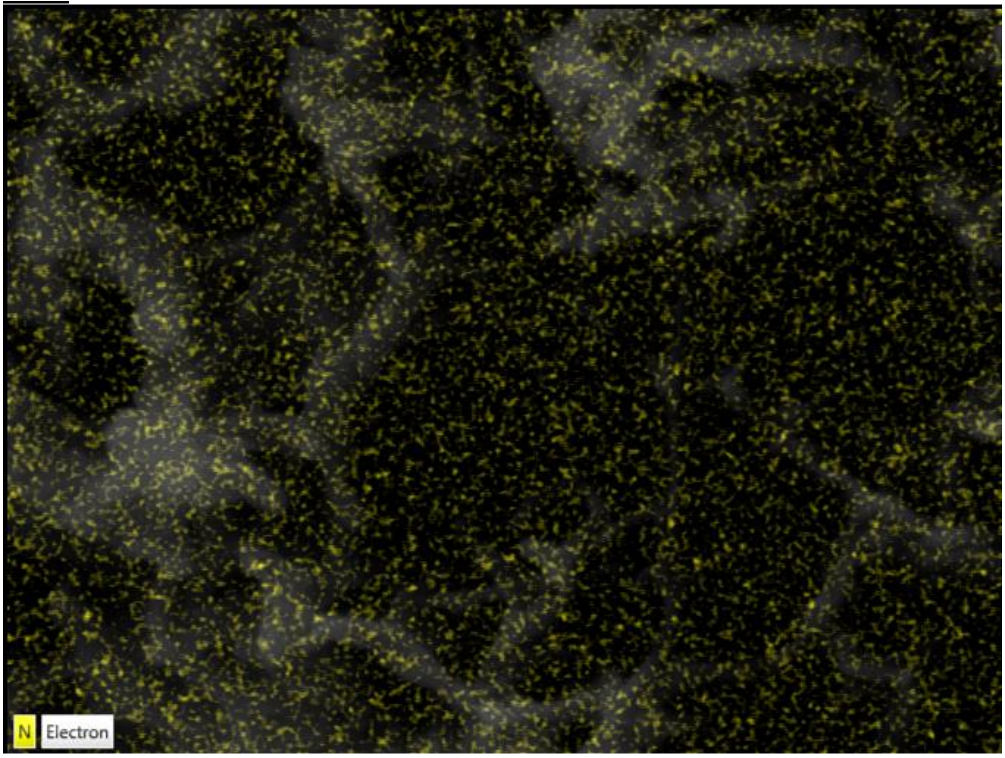

SEM image and nitrogen EDX mapping of $SN_{Bal}300_{Mu}$

SEM

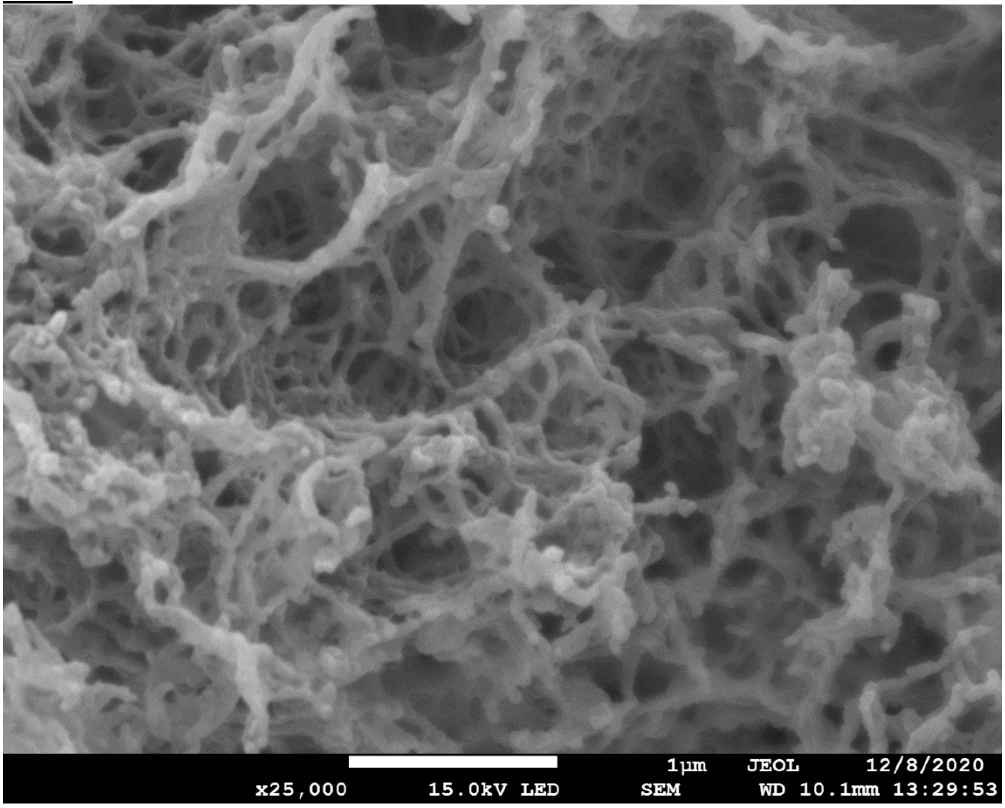

EDX

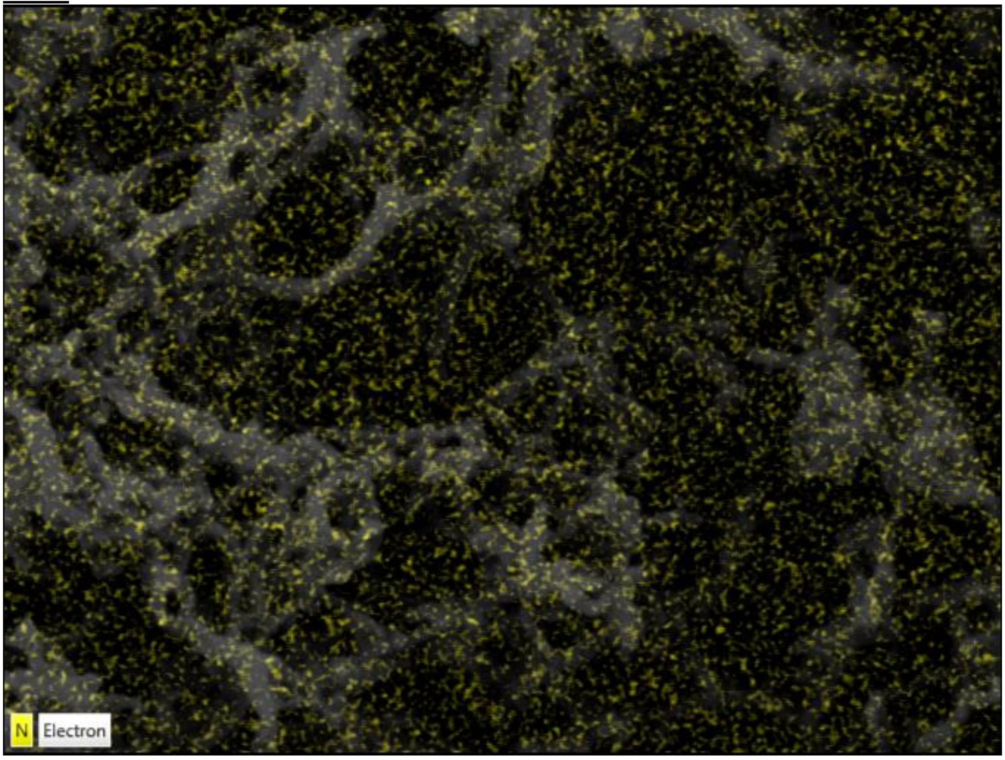

SEM image and nitrogen EDX mapping of $SN_{Ure}300_{Mu}$

SEM

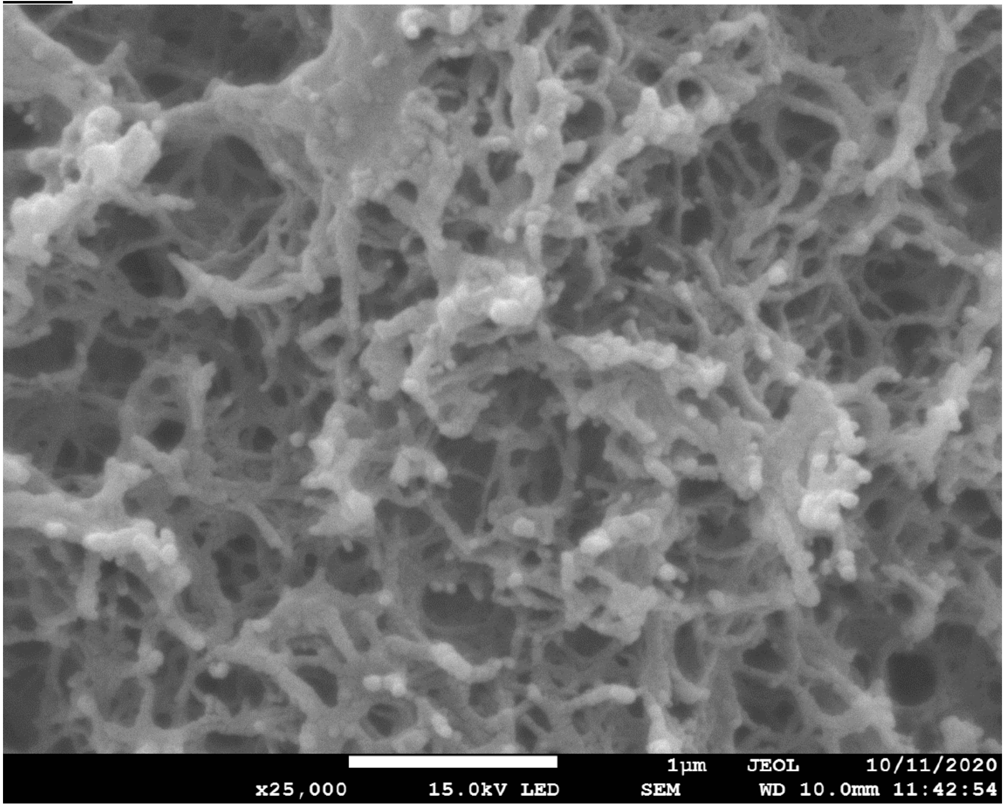

EDX

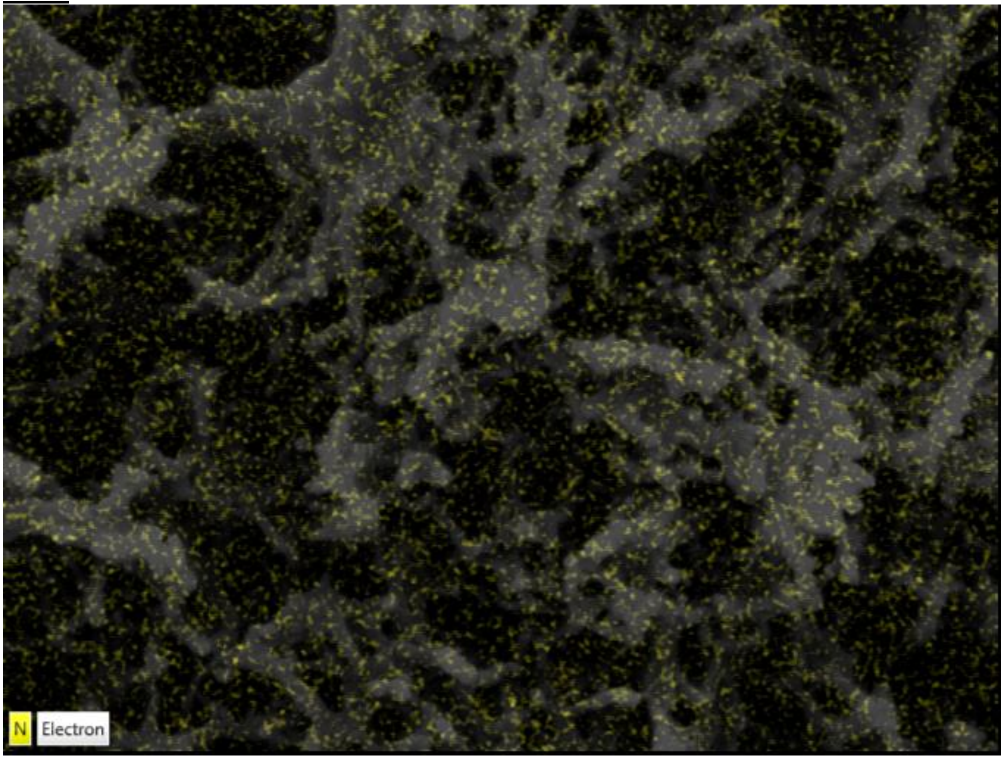

SEM image and nitrogen EDX mapping of $SN_{Mel}300_{Mu}$

SEM image of Starbon® derived material

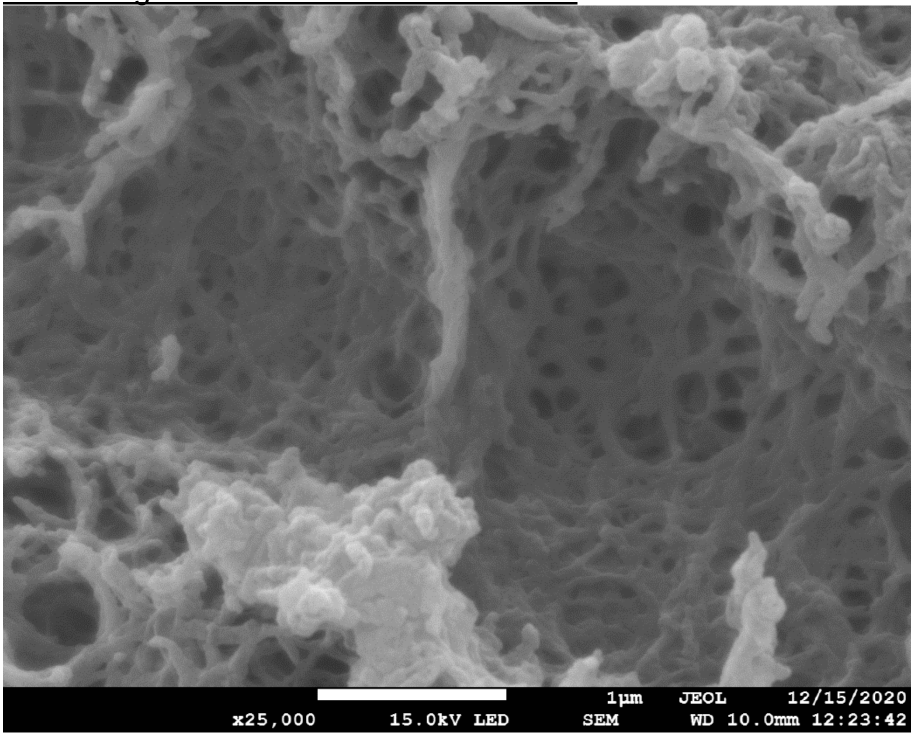

Lower magnification SEM image showing the needles as well as Starbon® derived material

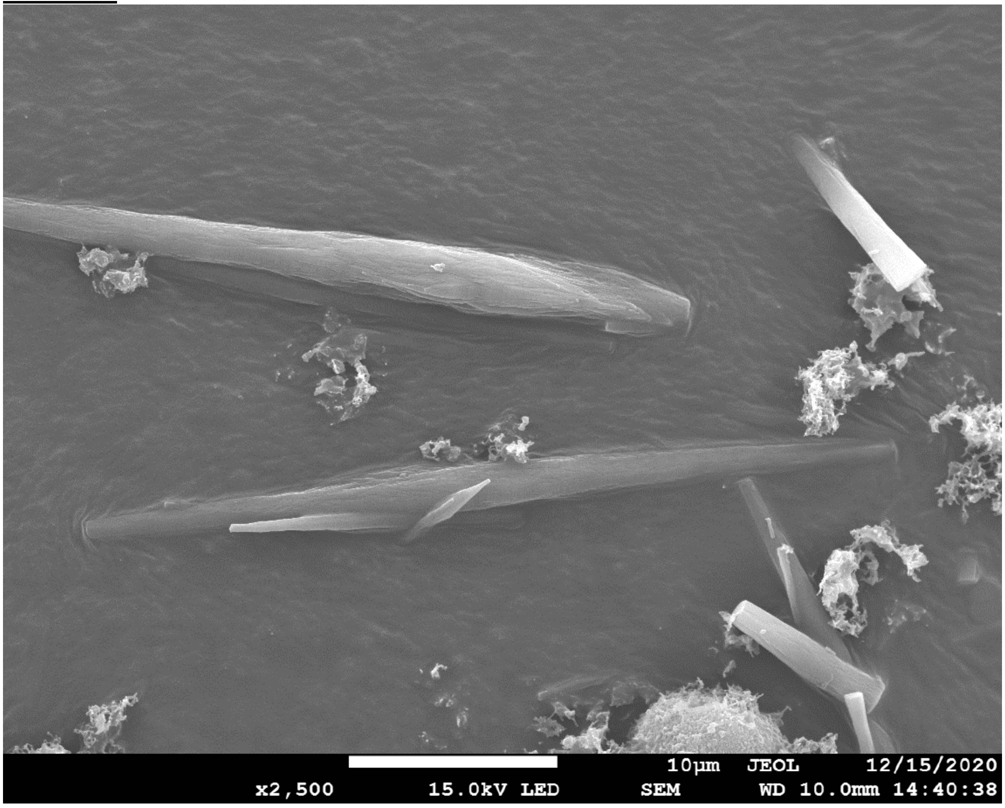

EDX of Starbon® derived material

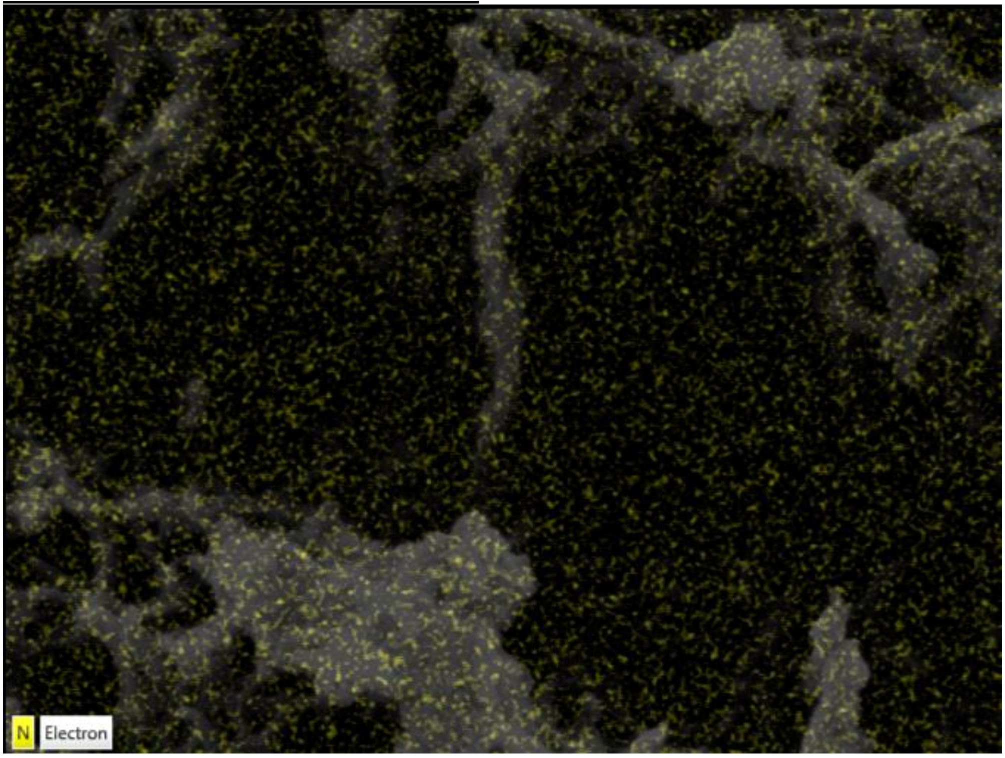

EDX of needles as well as Starbon® derived material

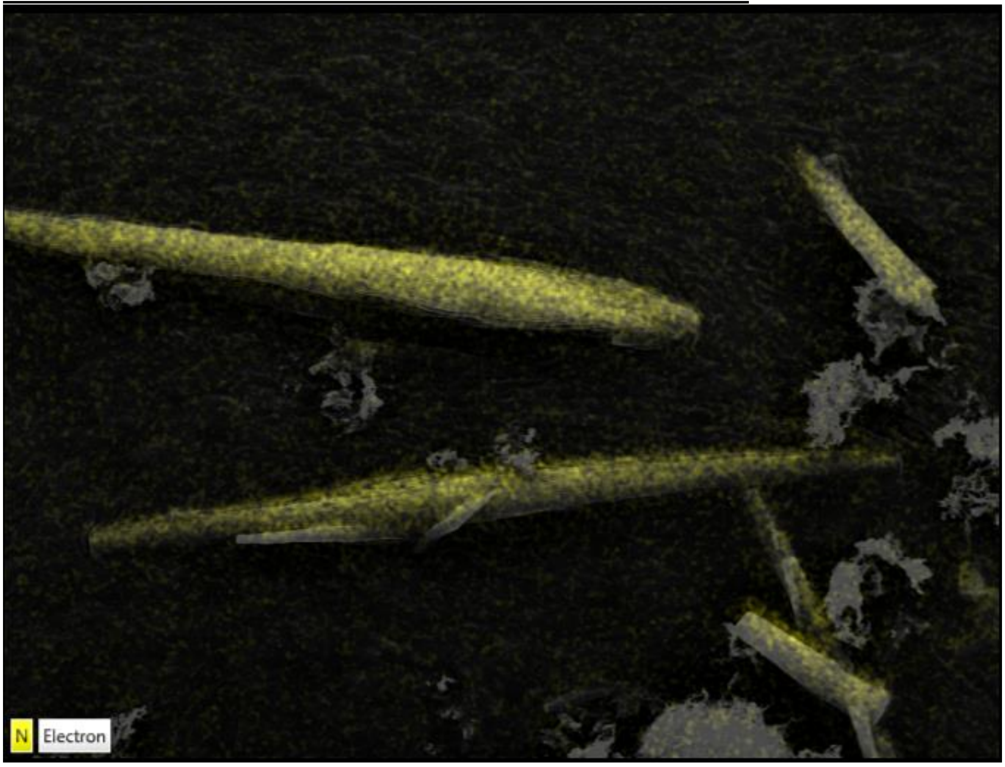

SEM image and nitrogen EDX mapping of $SN_{Nic}300_{Mu}$

SEM

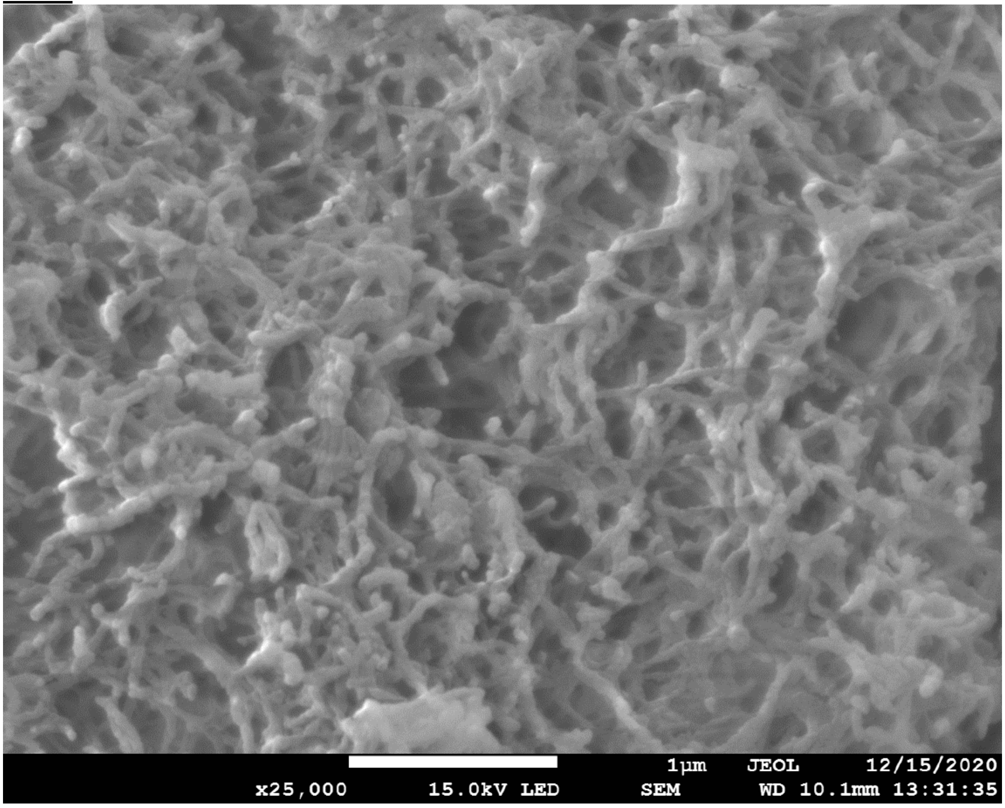

EDX

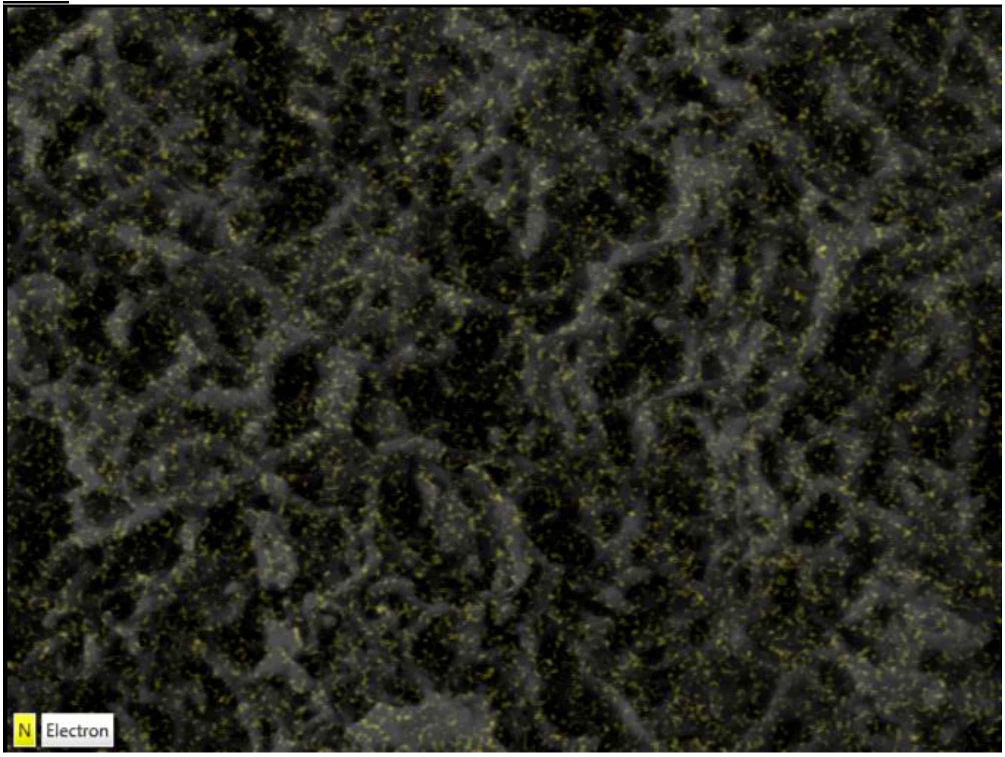

TEM images of S300 and S800

S300

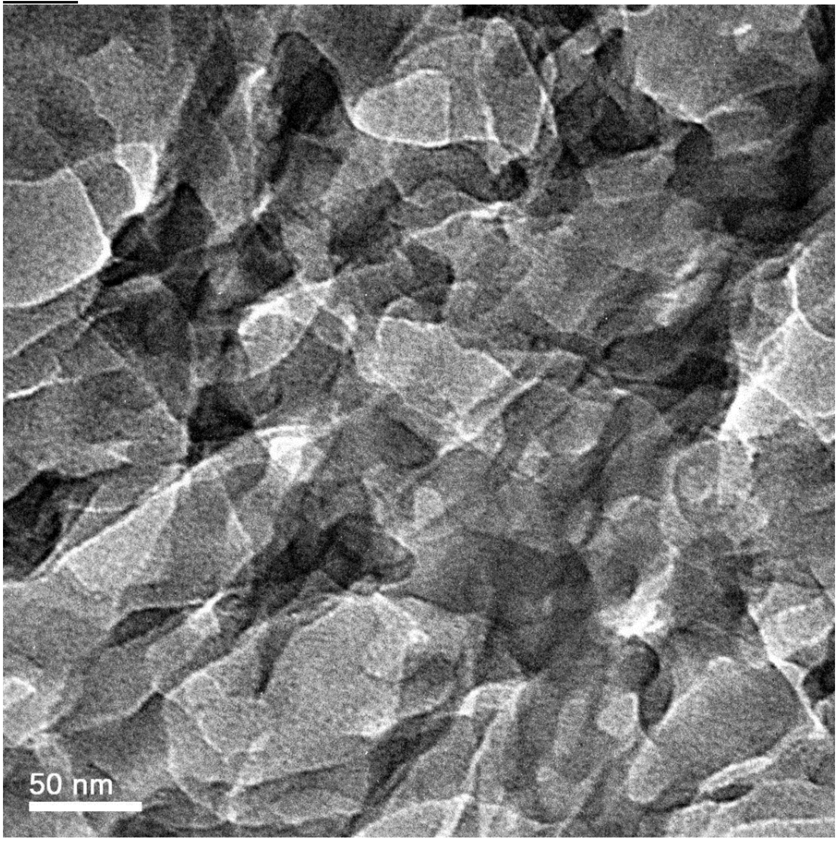

S800

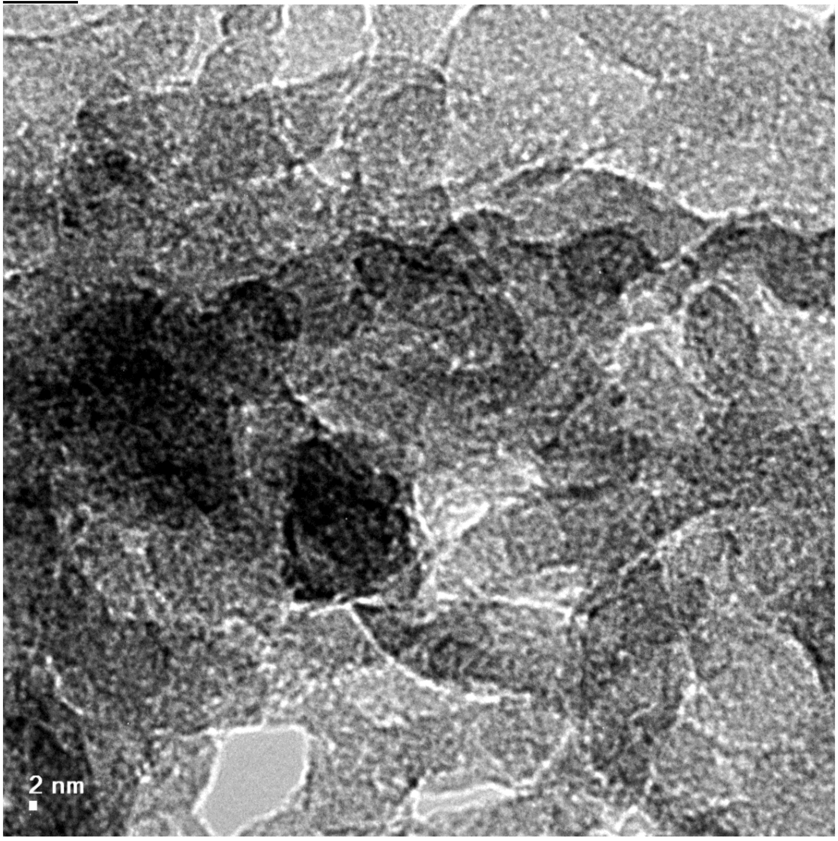

TEM images of $SN_{Gly}300_{Th}$ and $SN_{Gly}800_{Th}$

$SN_{Gly}300_{Th}$

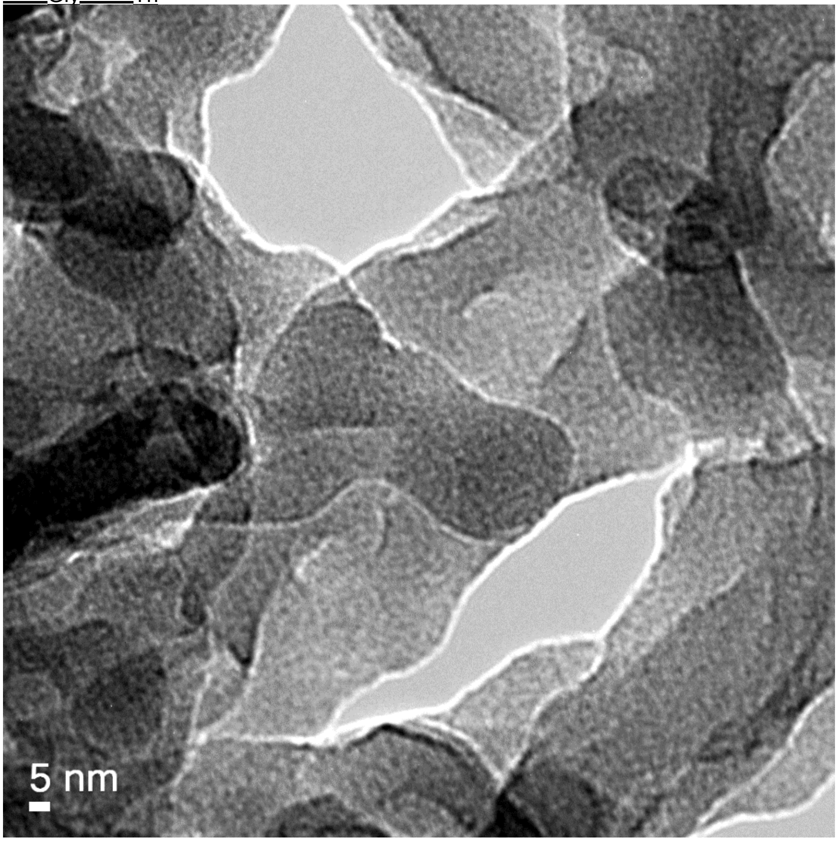

$SN_{Gly}800_{Th}$

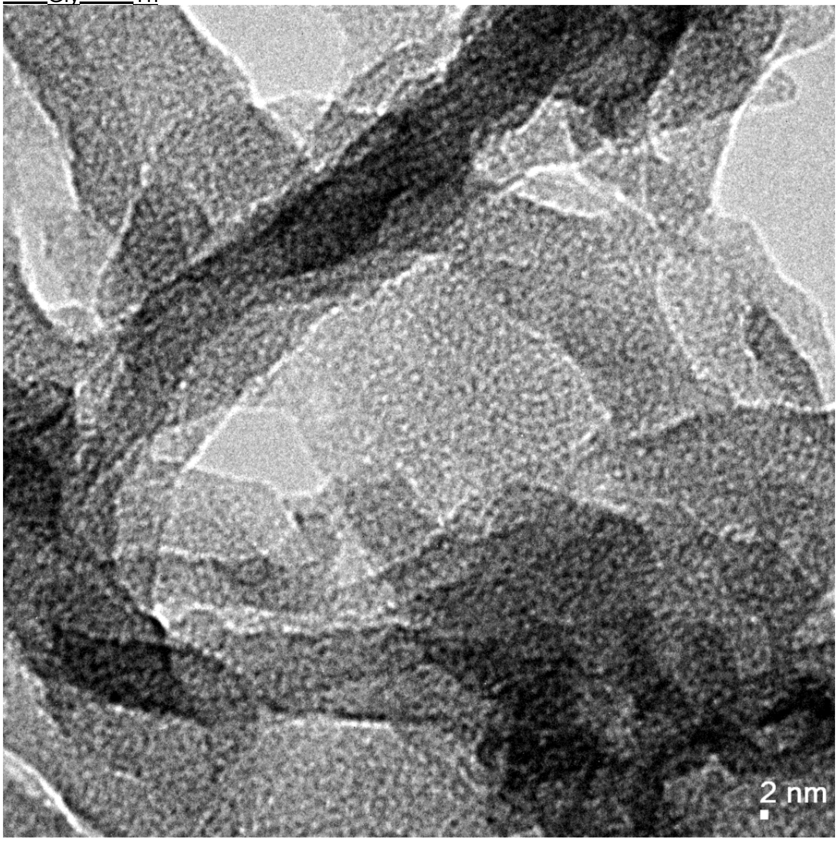

TEM images of $SN_{Bal}300_{Th}$ and $SN_{Bal}800_{Th}$

$SN_{Bal}300_{Th}$

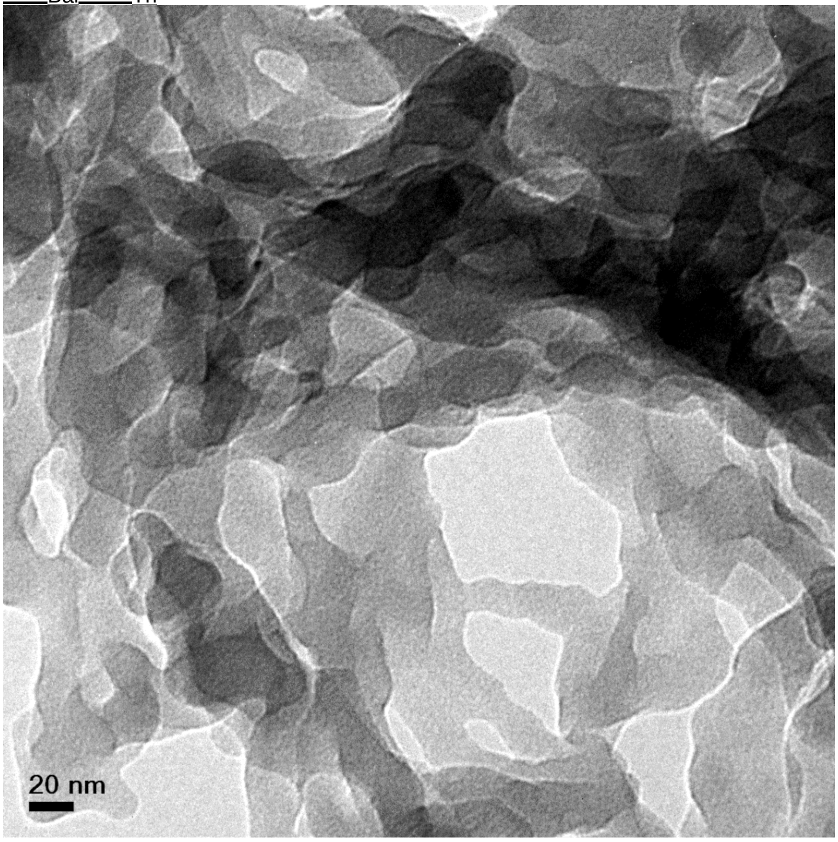

$SN_{Bal}800_{Th}$

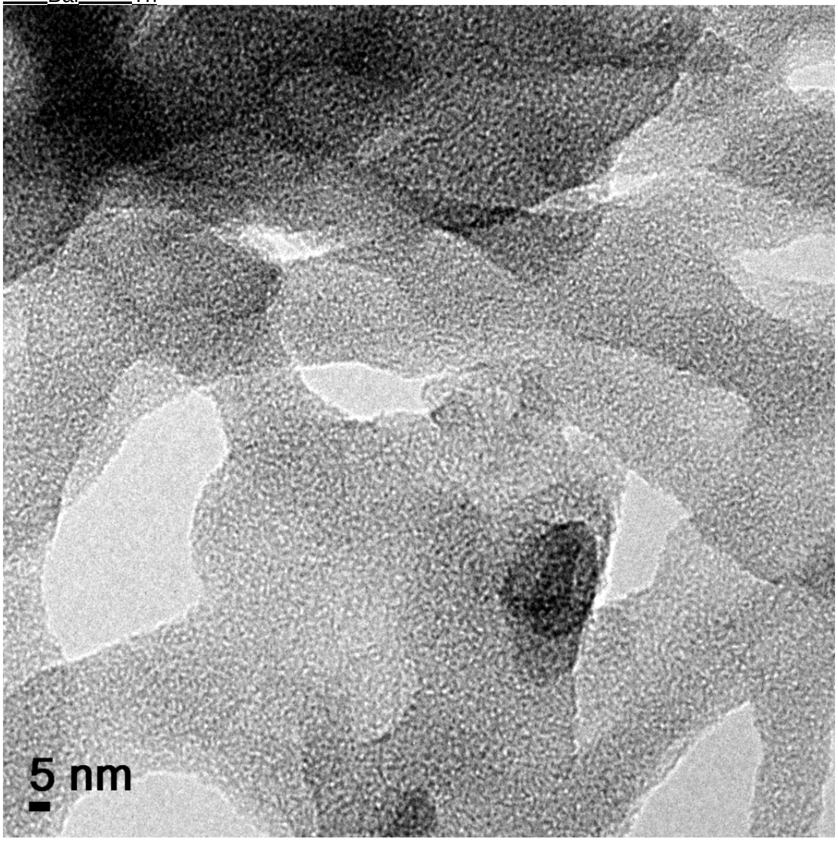

TEM images of $SN_{Ure}300_{Th}$ and $SN_{Ure}800_{Th}$

$SN_{Ure}300_{Th}$

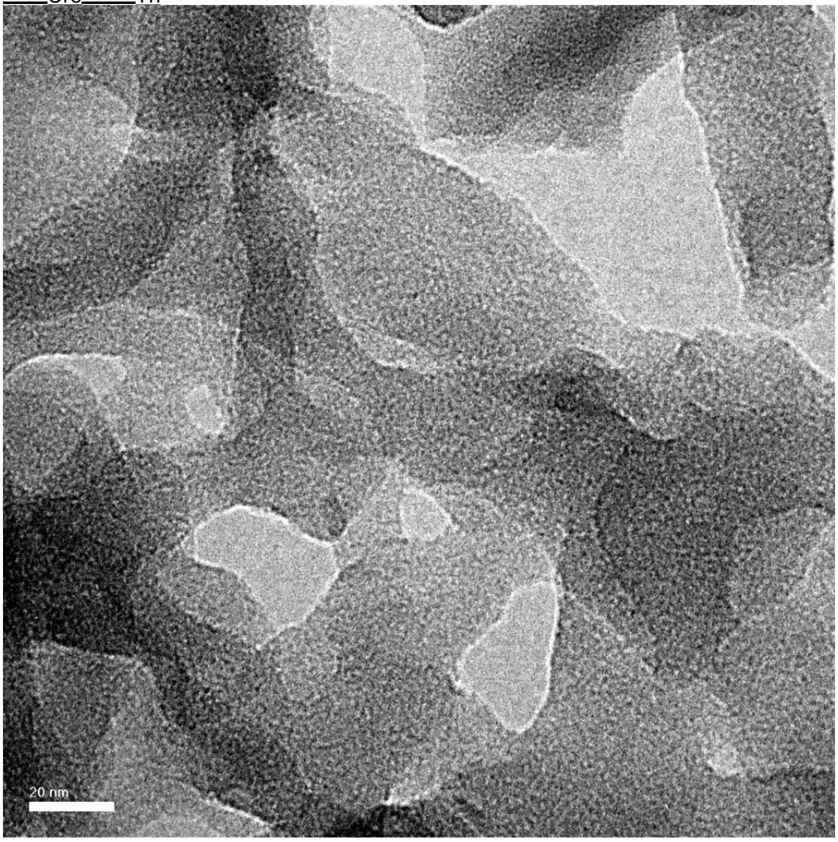

$SN_{Ure}800_{Th}$

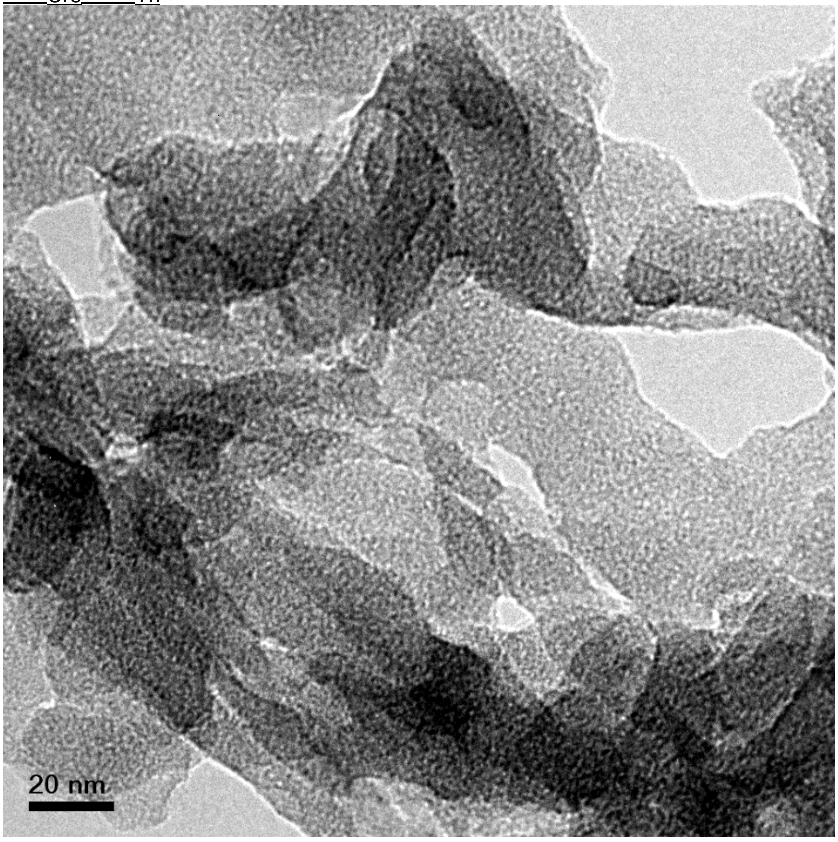

TEM images of $SN_{Mel}300_{Th}$ and $SN_{Mel}800_{Th}$

$SN_{Mel}300_{Th}$

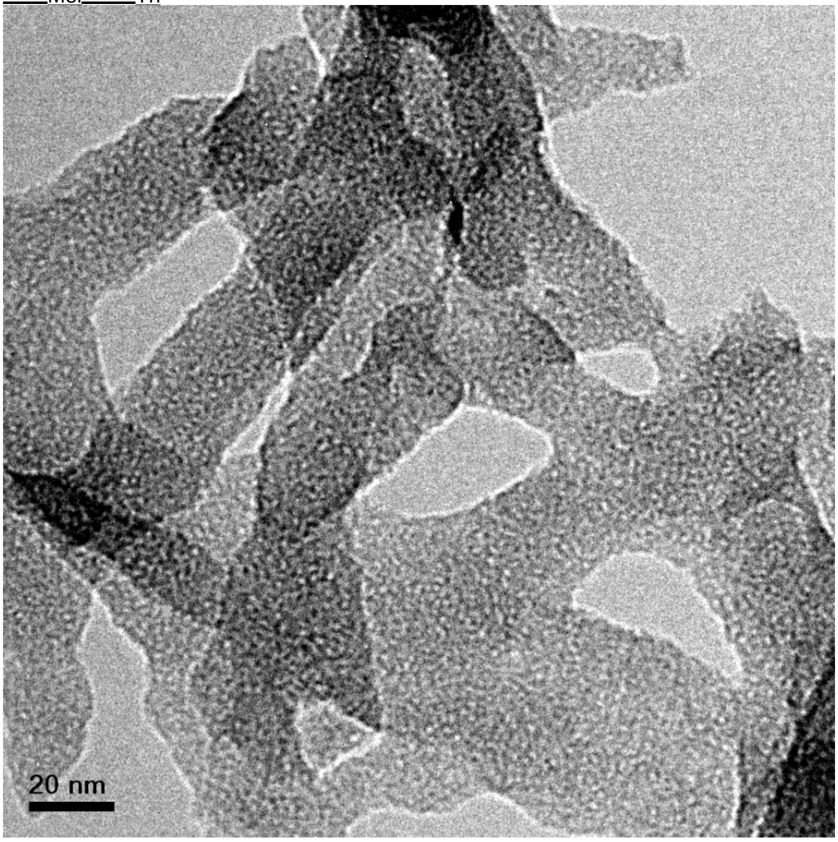

$SN_{Mel}800_{Th}$

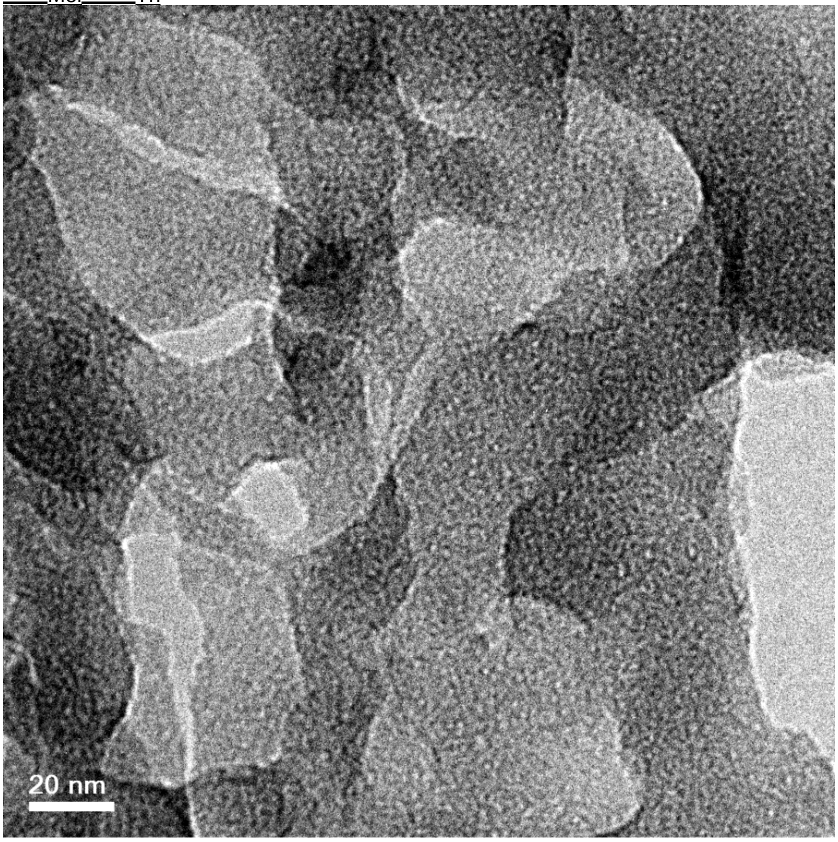

TEM images of $SN_{Nic}300_{Th}$ and $SN_{Nic}800_{Th}$

$SN_{Nic}300_{Th}$

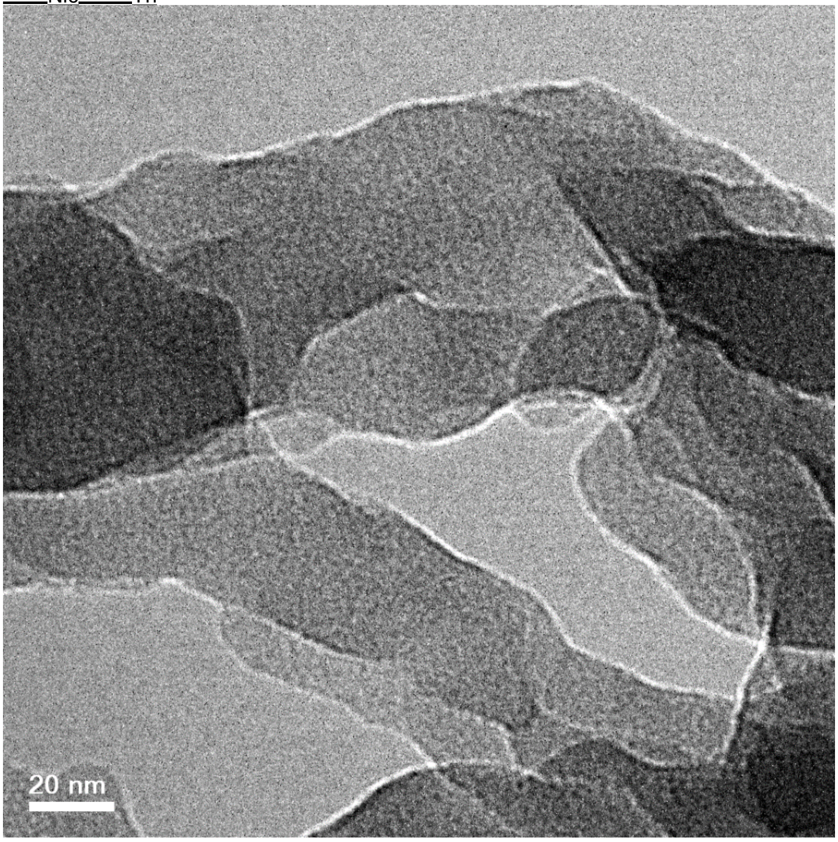

$SN_{Nic}800_{Th}$

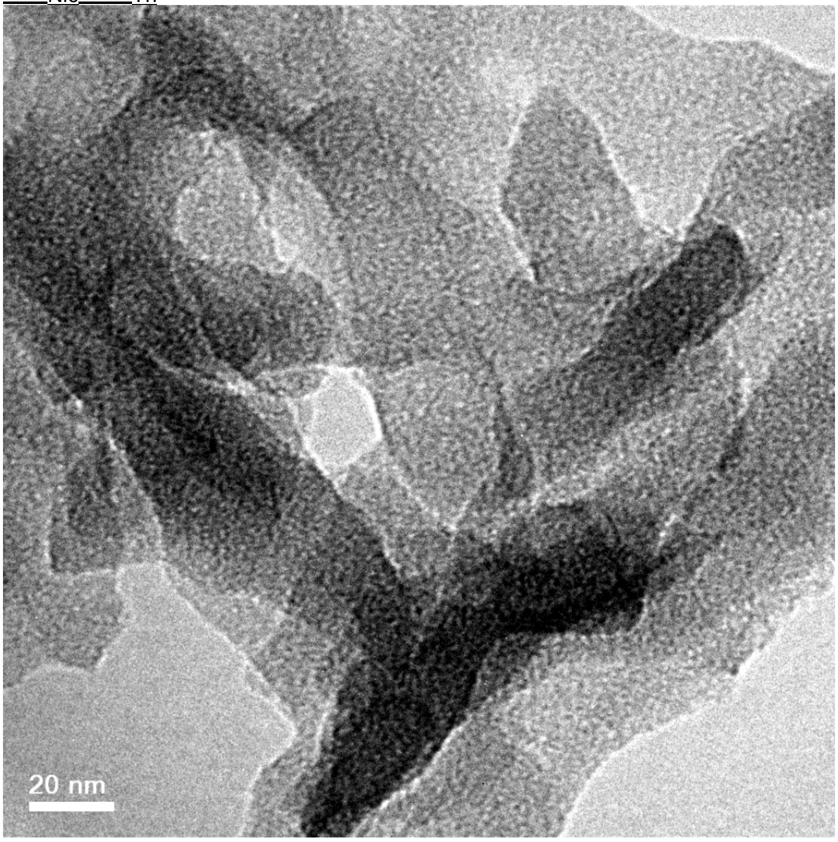

TEM images of SN_X300_{Mo}

$SN_{Gly}300_{Mo}$

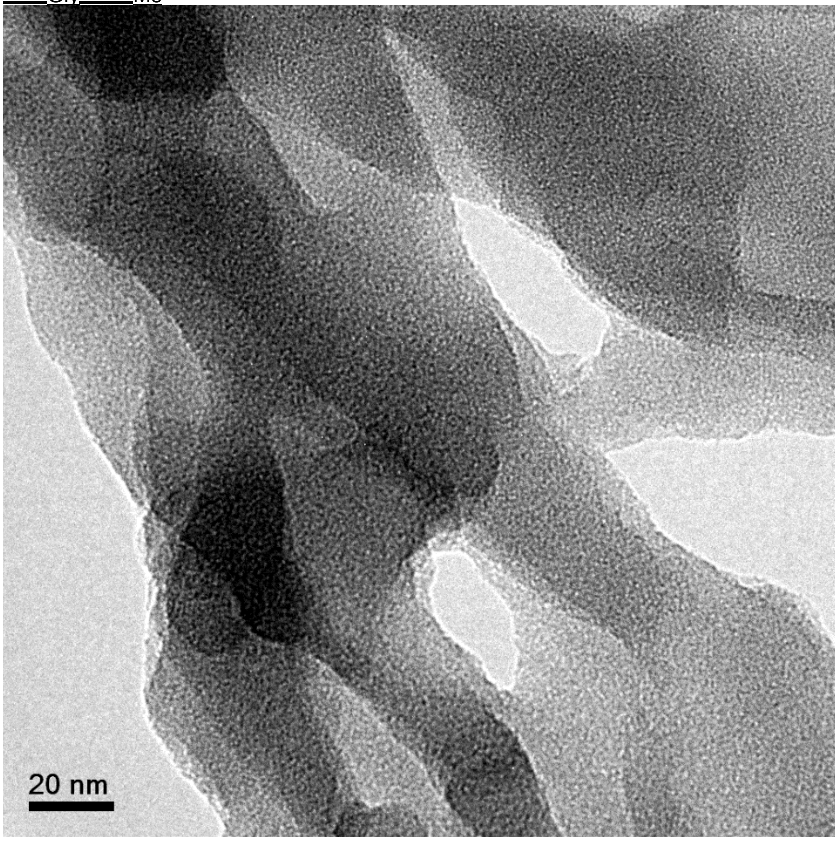

$SN_{Bal}300_{Mo}$

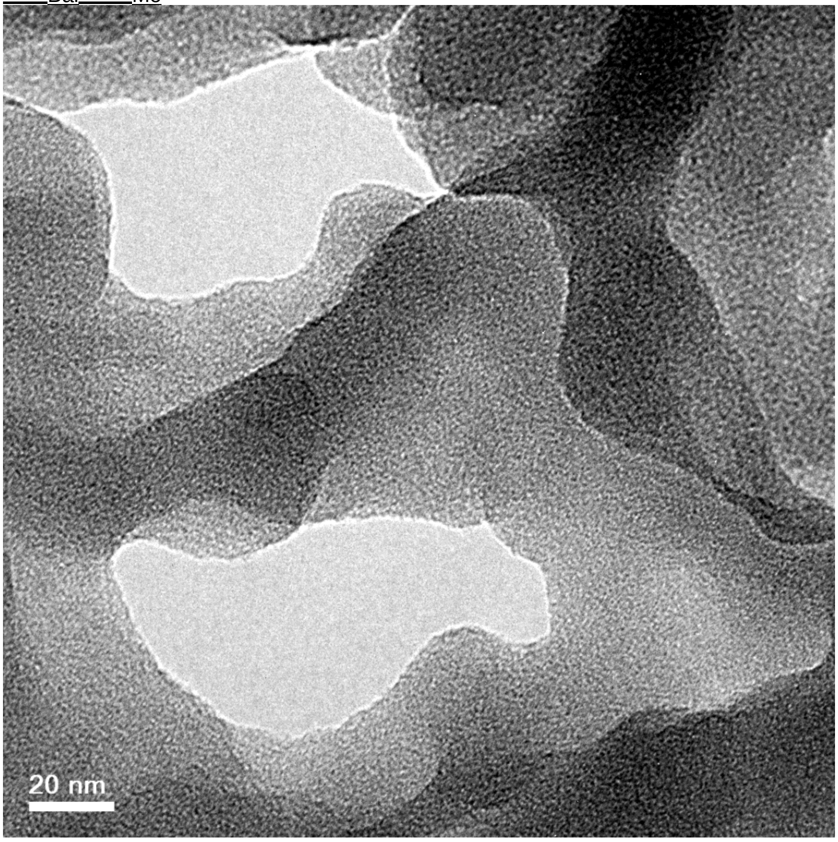

$\underline{SN_{Ure}300_{Mo}}$

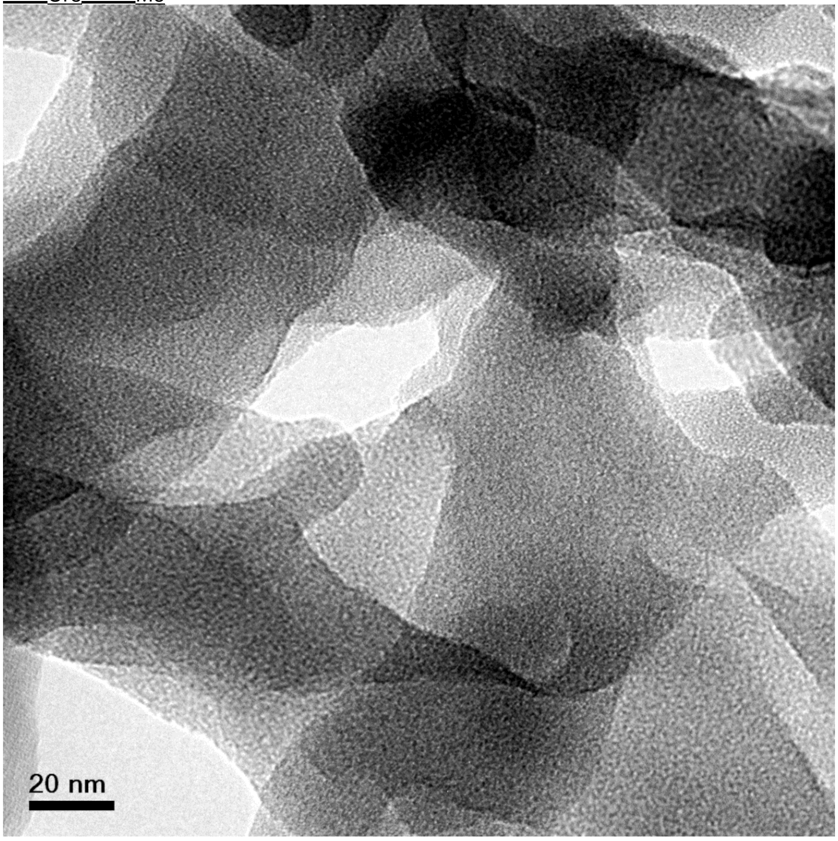

$\underline{SN_{Mel}300_{Mo}}$

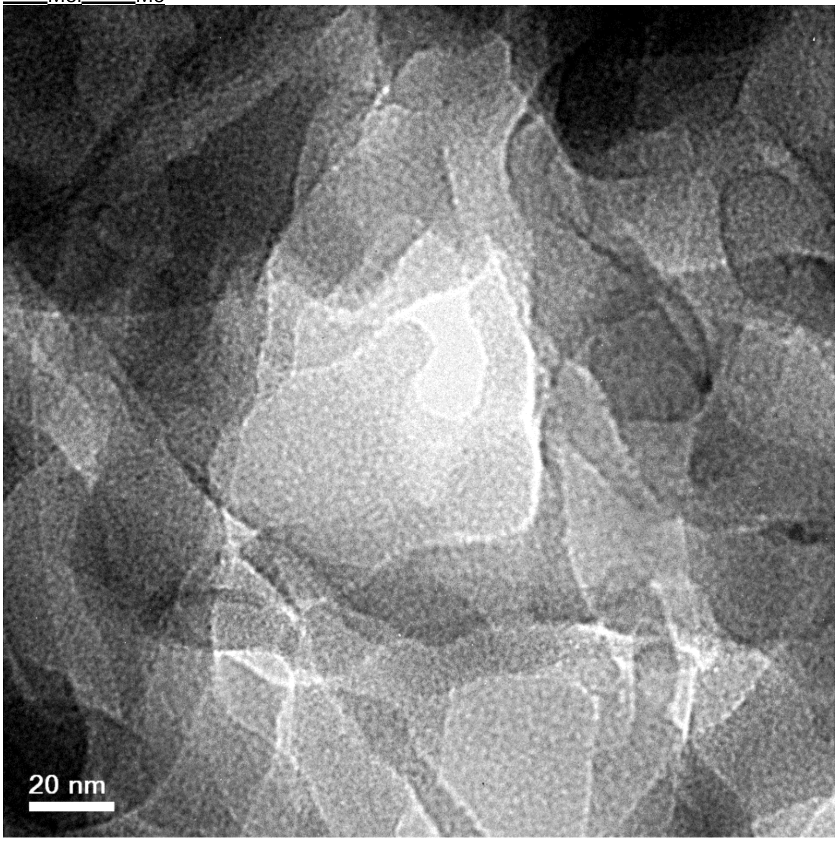

SN$_{Nic}$300$_{Mo}$

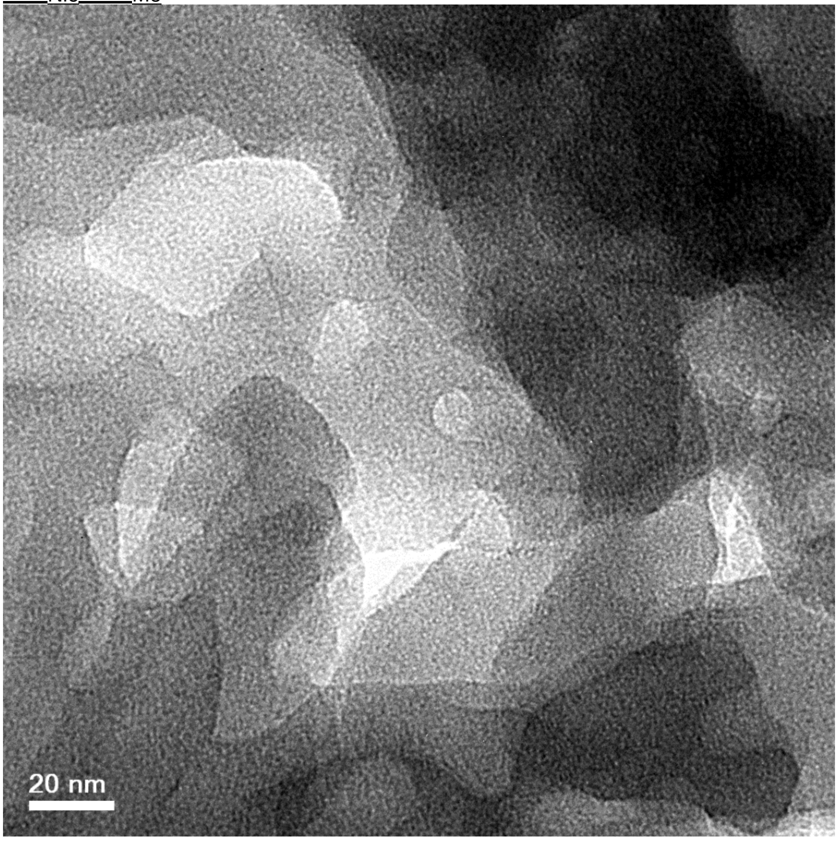

TEM images of SN_X300_{Mu}

$SN_{Gly}300_{Mu}$

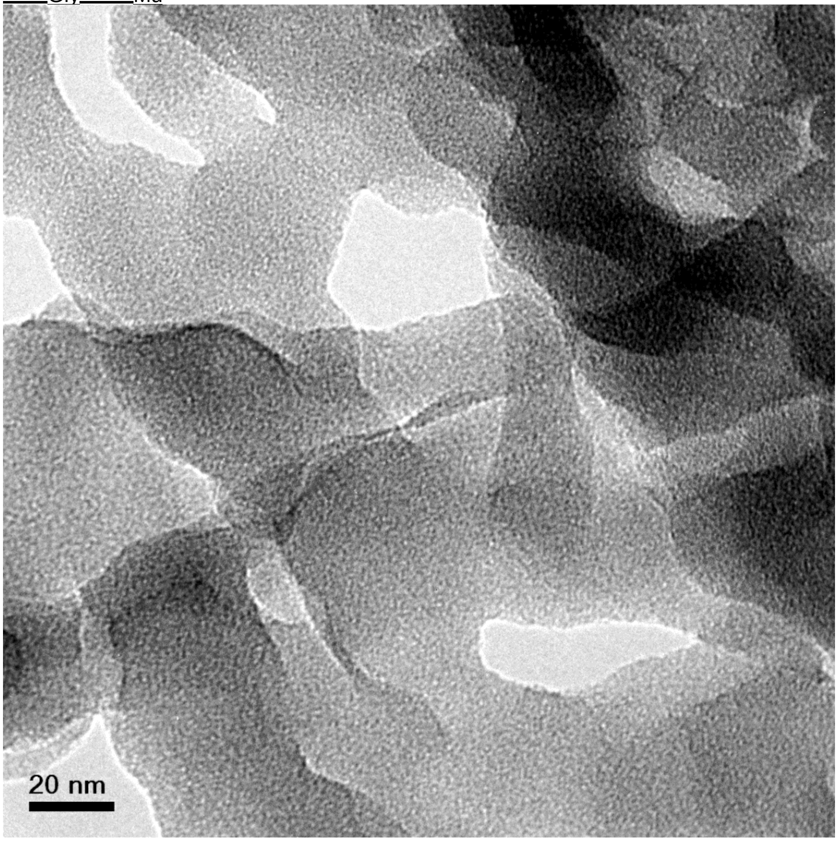

$SN_{Bal}300_{Mu}$

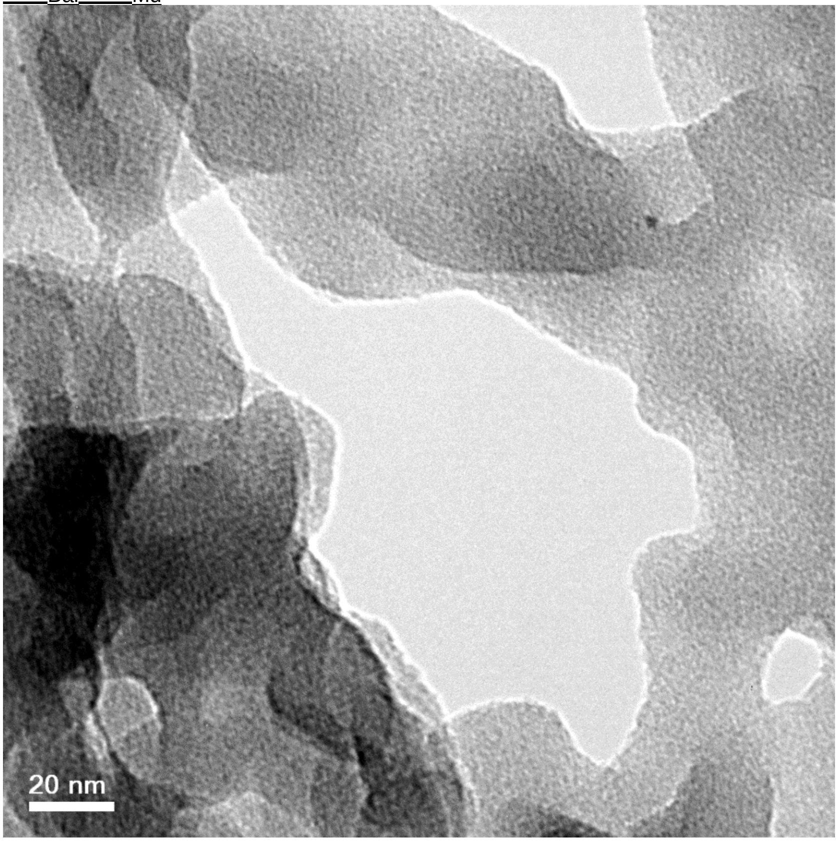

$\underline{SN_{Ure}300_{Mu}}$

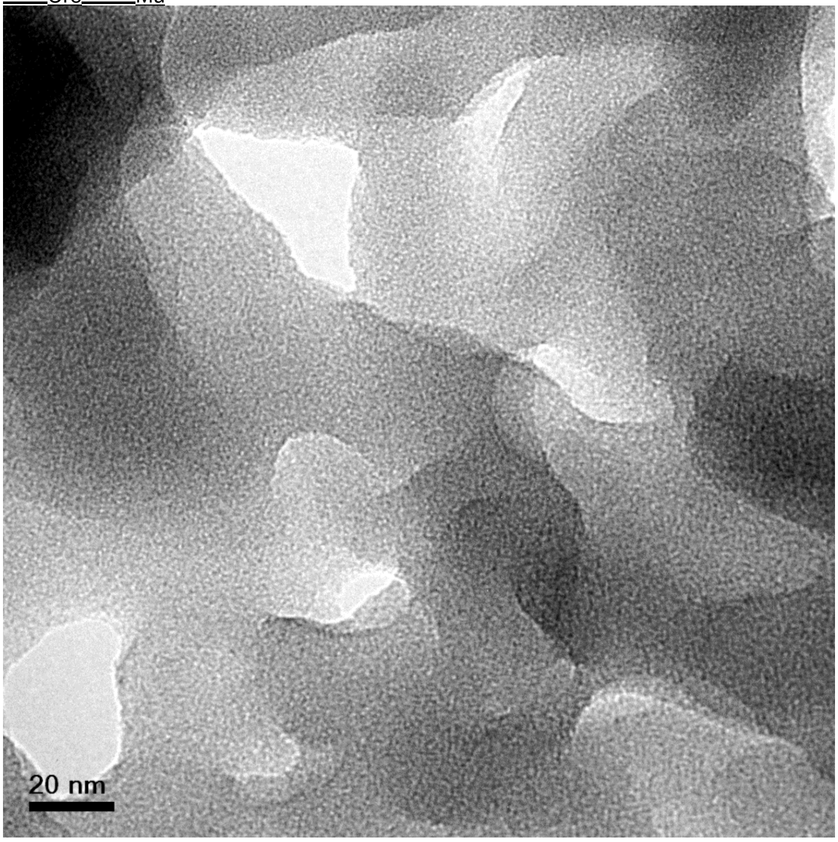

$\underline{SN_{Mel}300_{Mu}}$

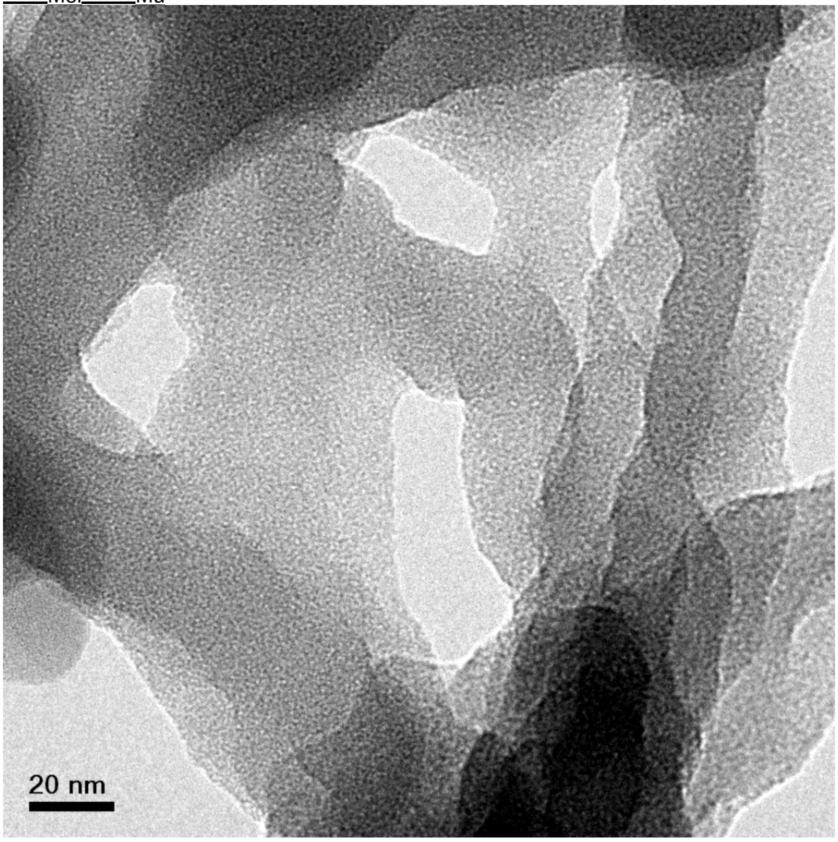

$SN_{Nic}300_{Mu}$

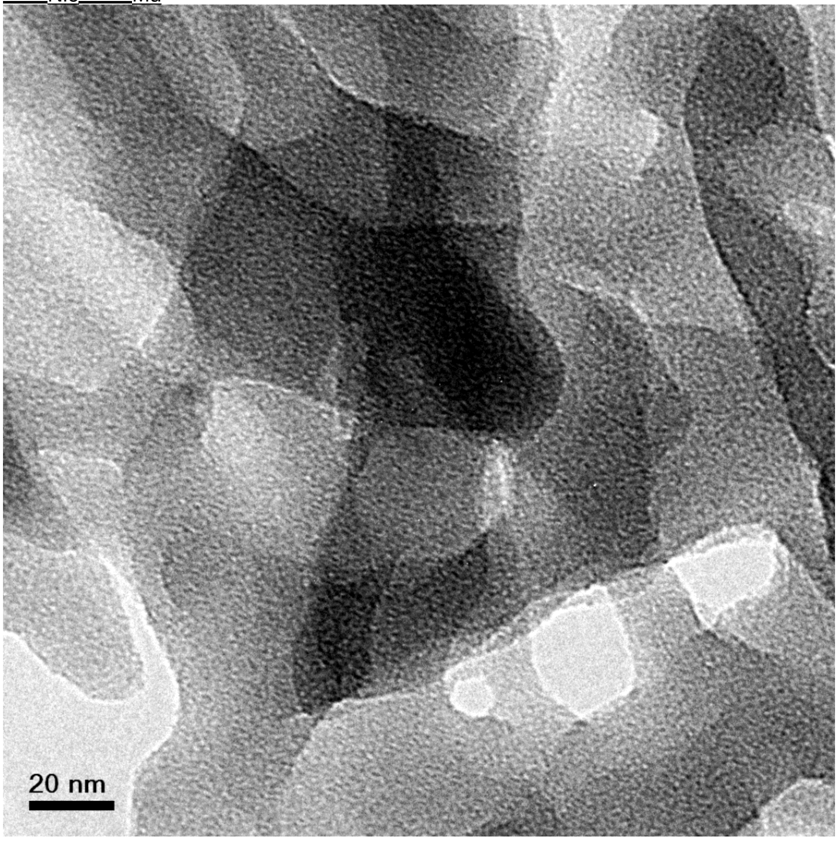

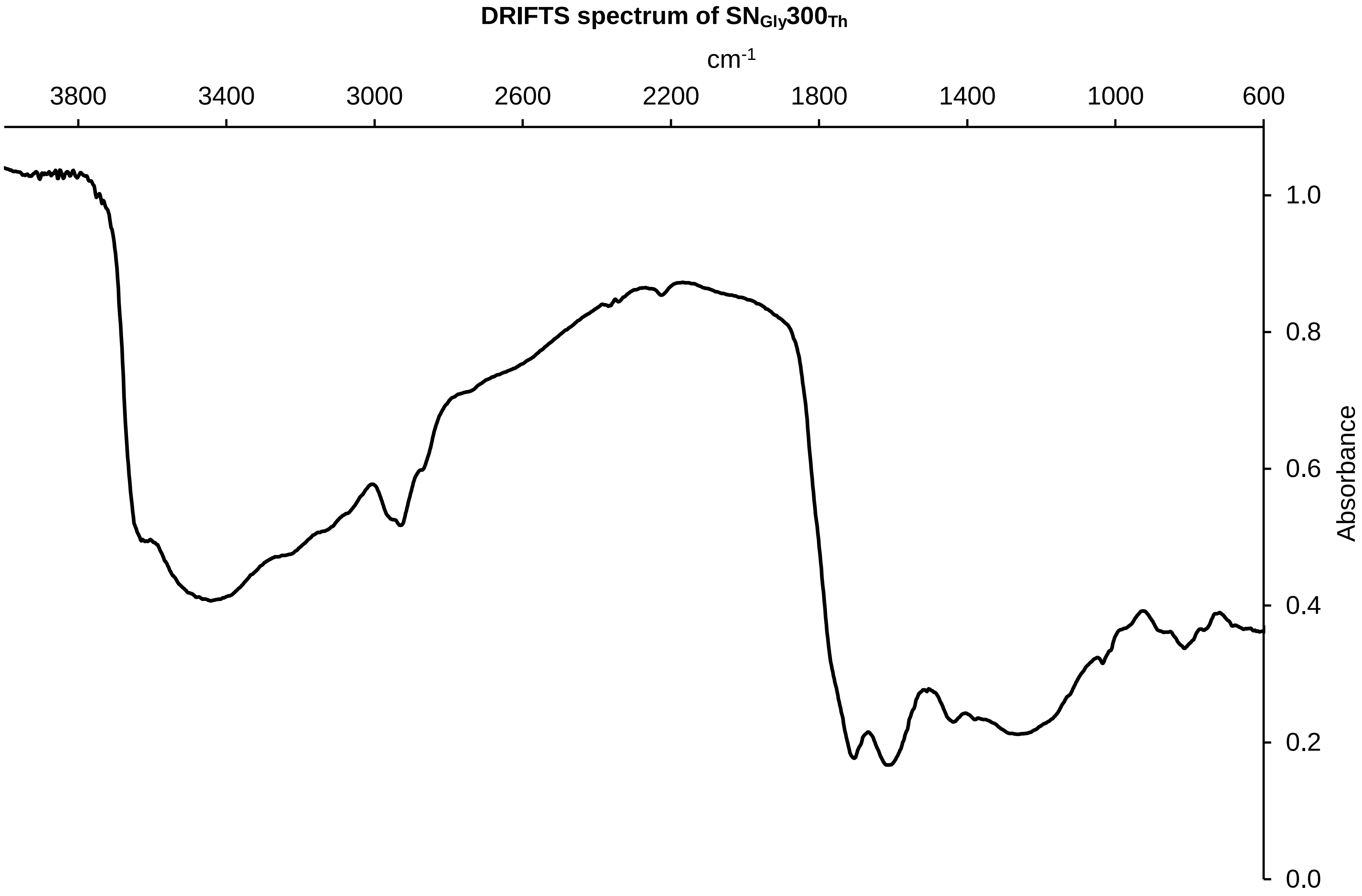
DRIFTS spectrum of SNGly300Th
cm-1
3800
3400
3000
2600
2200
1800
1400
1000
600
1.0
0.8
0.6
0.4
0.2
0.0
Absorbance

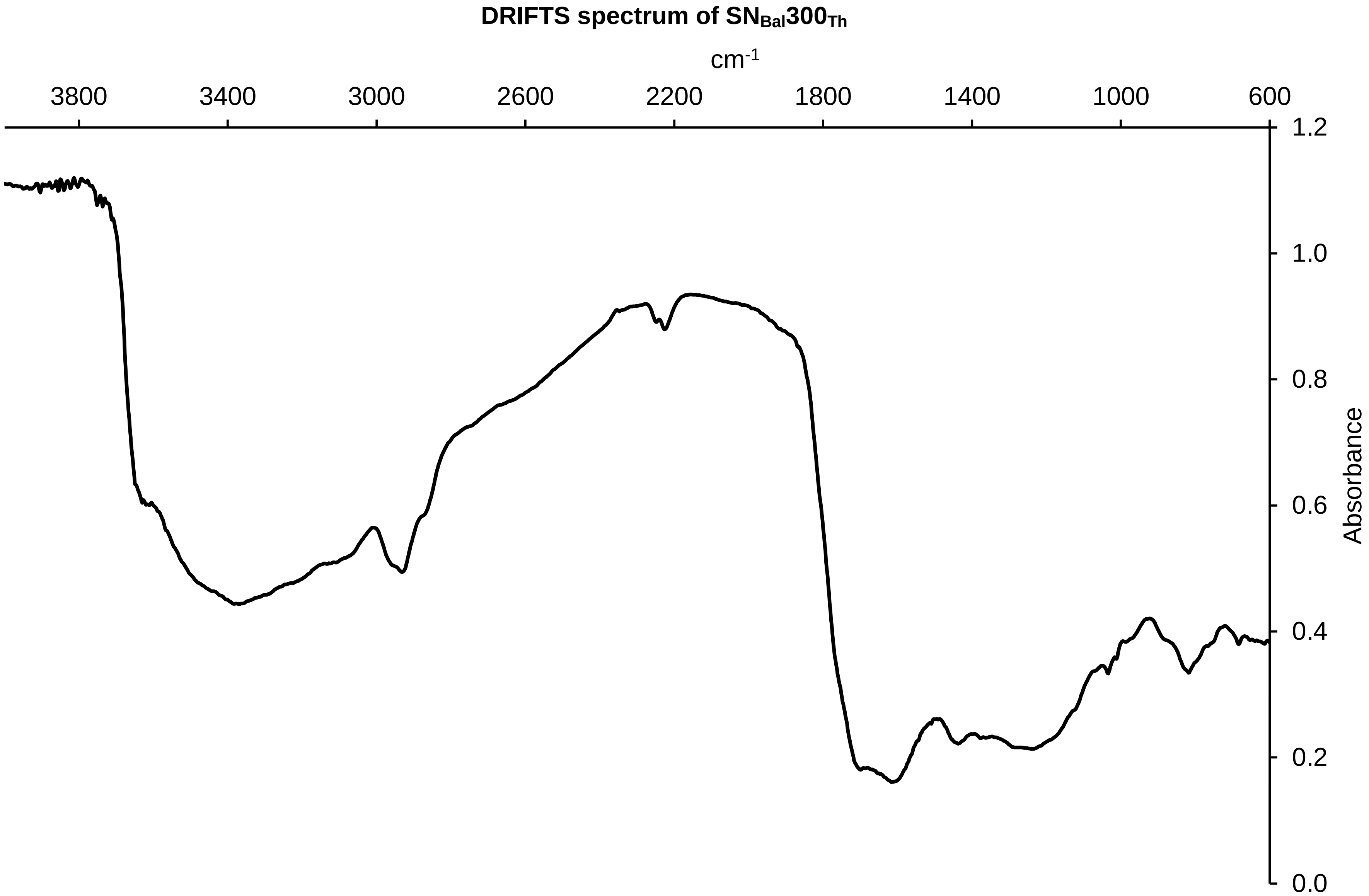
DRIFTS spectrum of SNBal300Th
cm-1
3800
3400
3000
2600
2200
1800
1400
1000
600
1.2
1.0
0.8
0.6
0.4
0.2
0.0
Absorbance

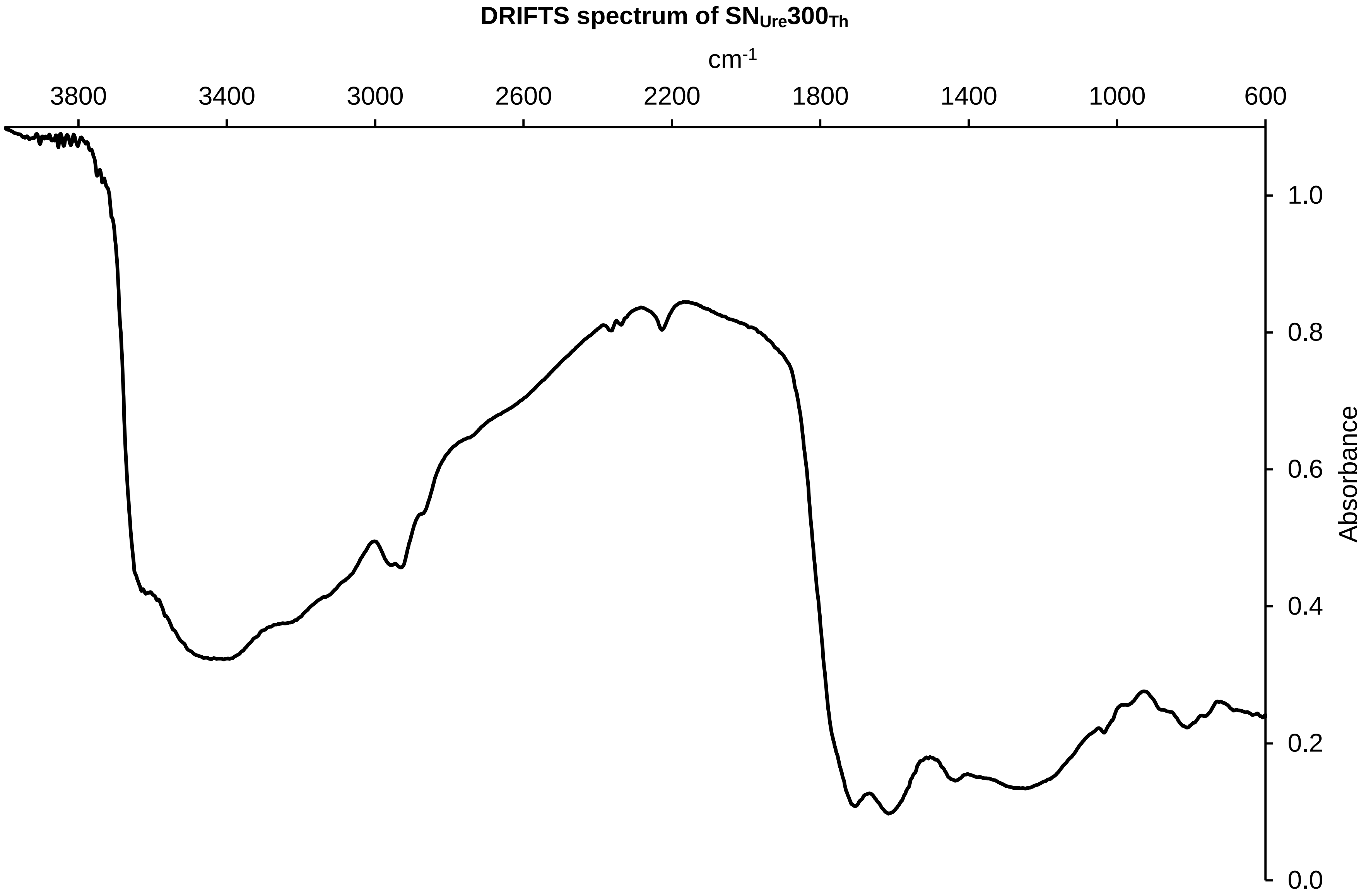
DRIFTS spectrum of SN$_{Ure}$300$_{Th}$
cm^{-1}
3800
3400
3000
2600
2200
1800
1400
1000
600
1.0
0.8
0.6
0.4
0.2
0.0
Absorbance

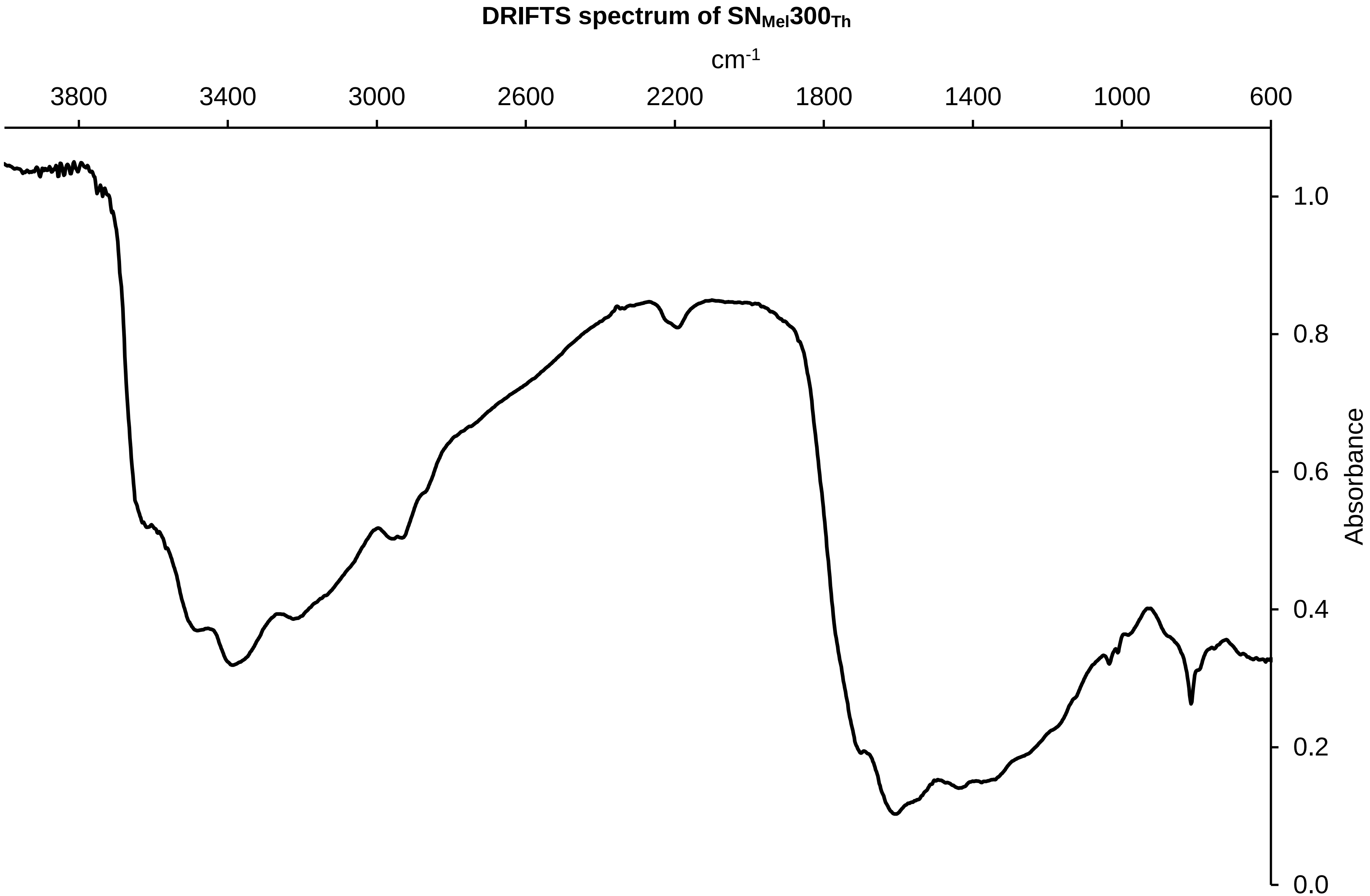

DRIFTS spectrum of SN$_{Mel}$300$_{Th}$
cm^{-1}
3800
3400
3000
2600
2200
1800
1400
1000
600
1.0
0.8
0.6
0.4
0.2
0.0
Absorbance

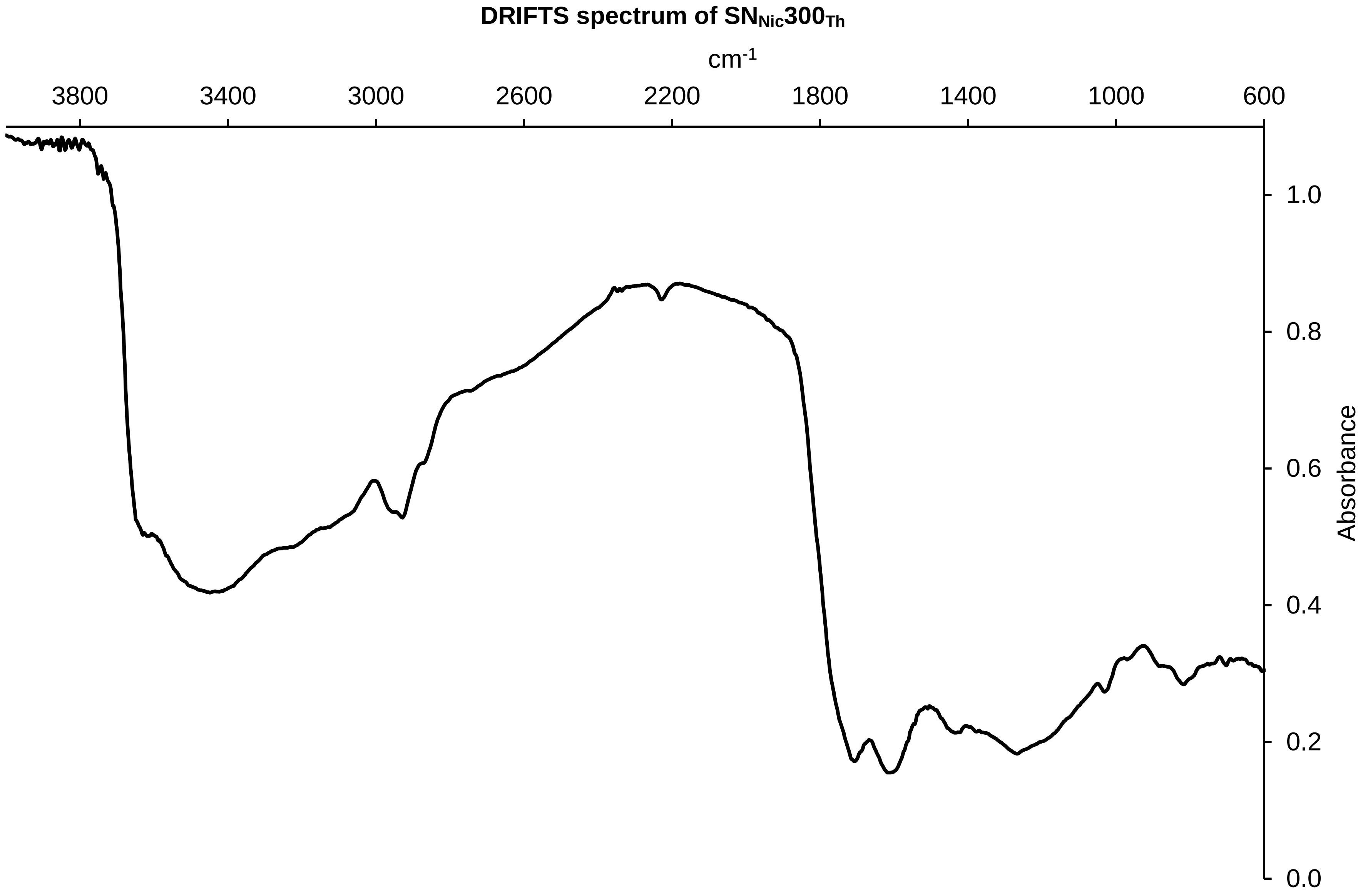
DRIFTS spectrum of SNNic300Th
cm-1
3800
3400
3000
2600
2200
1800
1400
1000
600
1.0
0.8
0.6
0.4
0.2
0.0
Absorbance

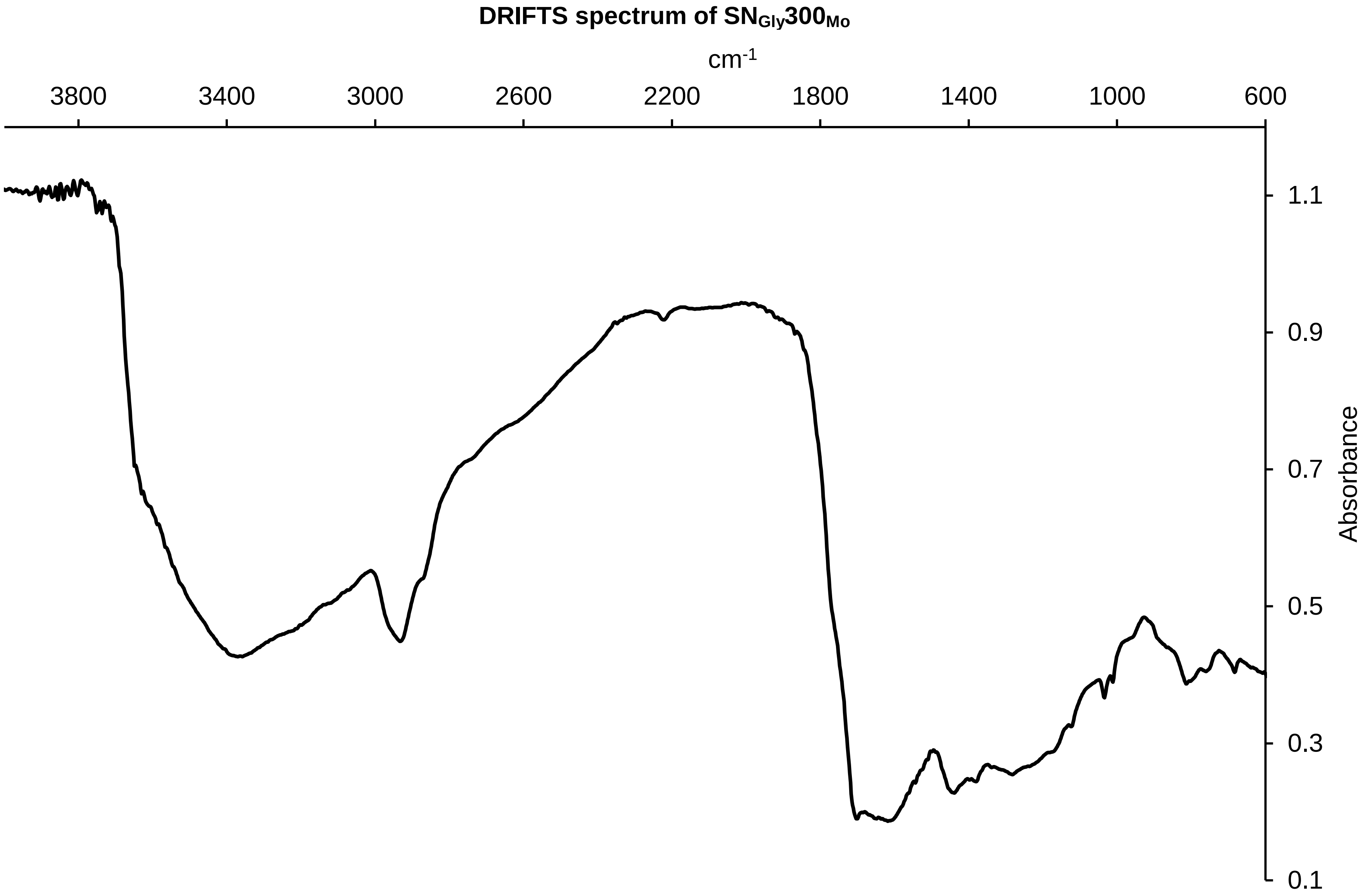
DRIFTS spectrum of $SN_{Gly}300_{Mo}$
cm-1
3800
3400
3000
2600
2200
1800
1400
1000
600
1.1
0.9
0.7
0.5
0.3
0.1
Absorbance

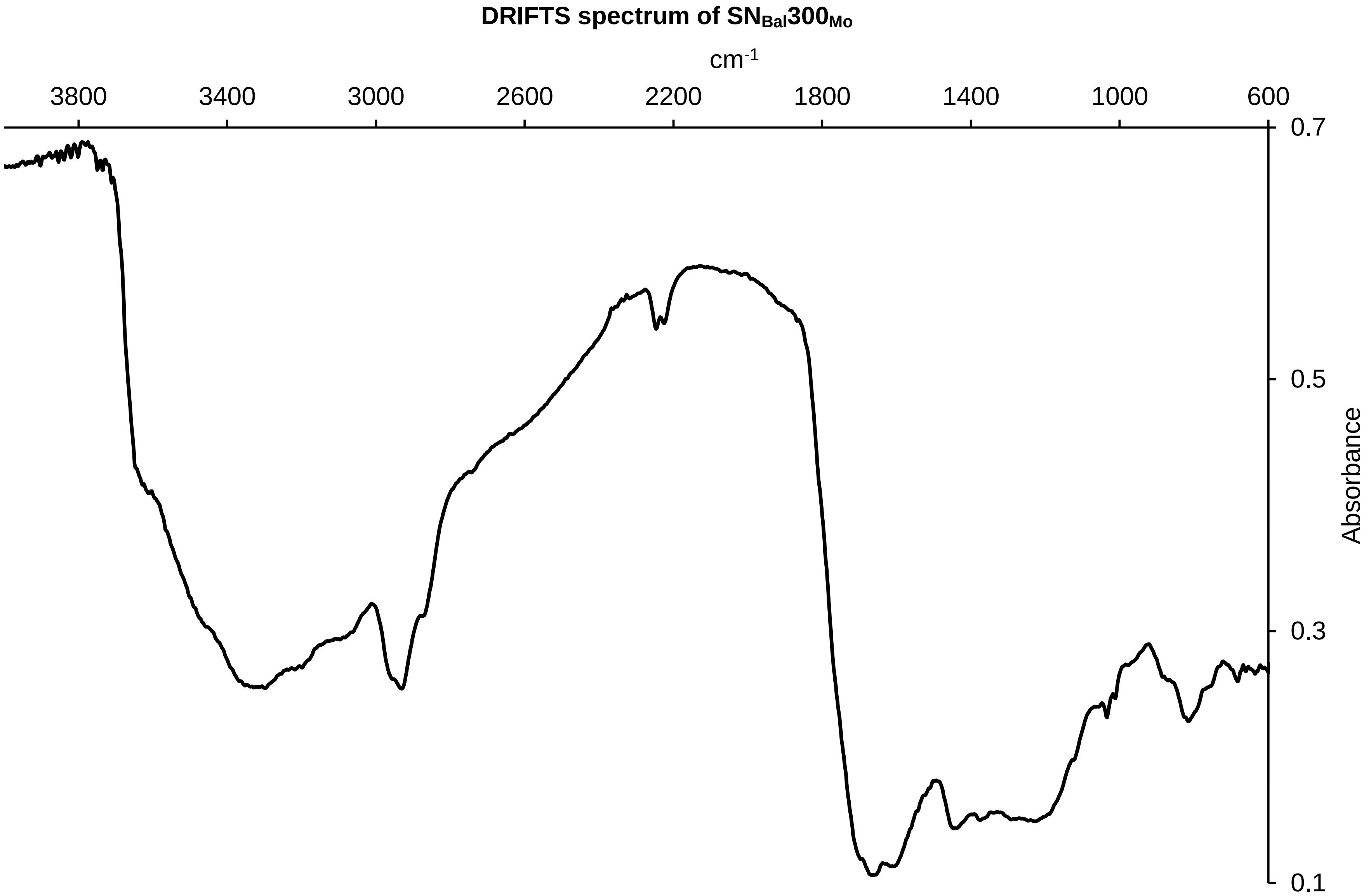

DRIFTS spectrum of SN$_{Bal}$300$_{Mo}$
cm^{-1}
3800
3400
3000
2600
2200
1800
1400
1000
600
0.7
0.5
0.3
0.1
Absorbance

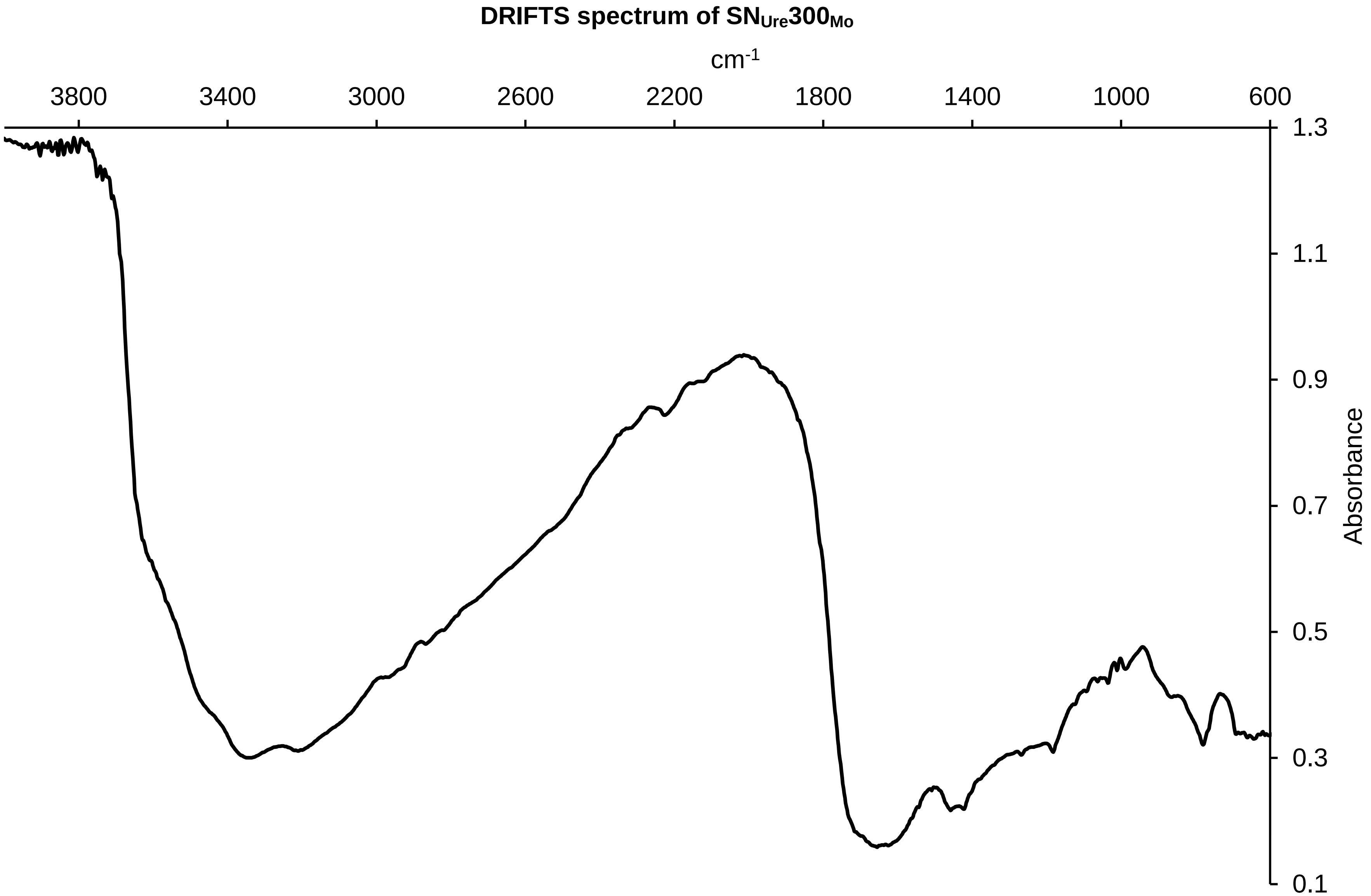
DRIFTS spectrum of SNUre300Mo
cm-1
3800
3400
3000
2600
2200
1800
1400
1000
600
1.3
1.1
0.9
0.7
0.5
0.3
0.1
Absorbance

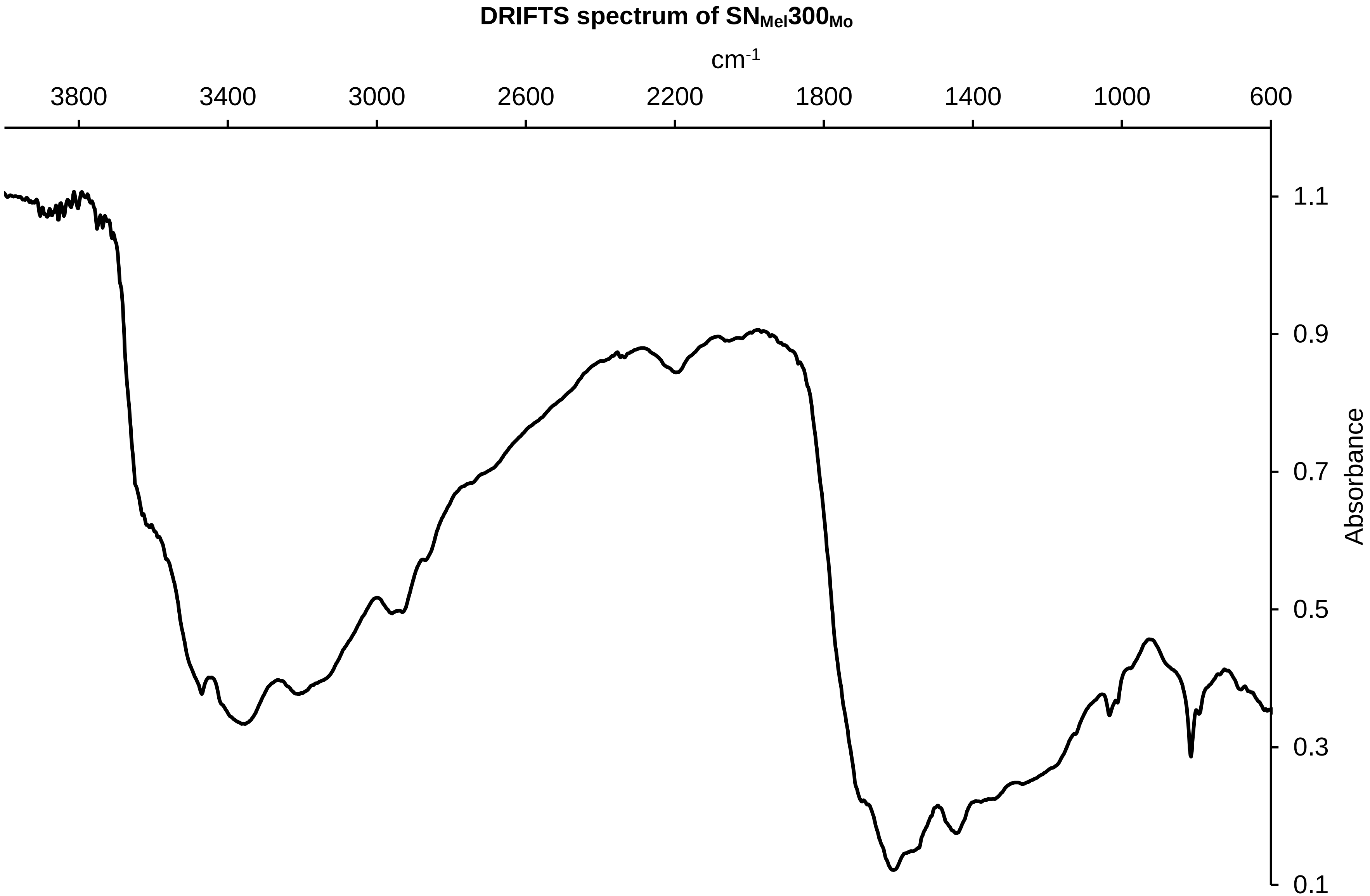
DRIFTS spectrum of SNMel300Mo
cm-1
3800
3400
3000
2600
2200
1800
1400
1000
600
1.1
0.9
0.7
0.5
0.3
0.1
Absorbance

DRIFTS spectrum of $SN_{Nic}300_{Mo}$

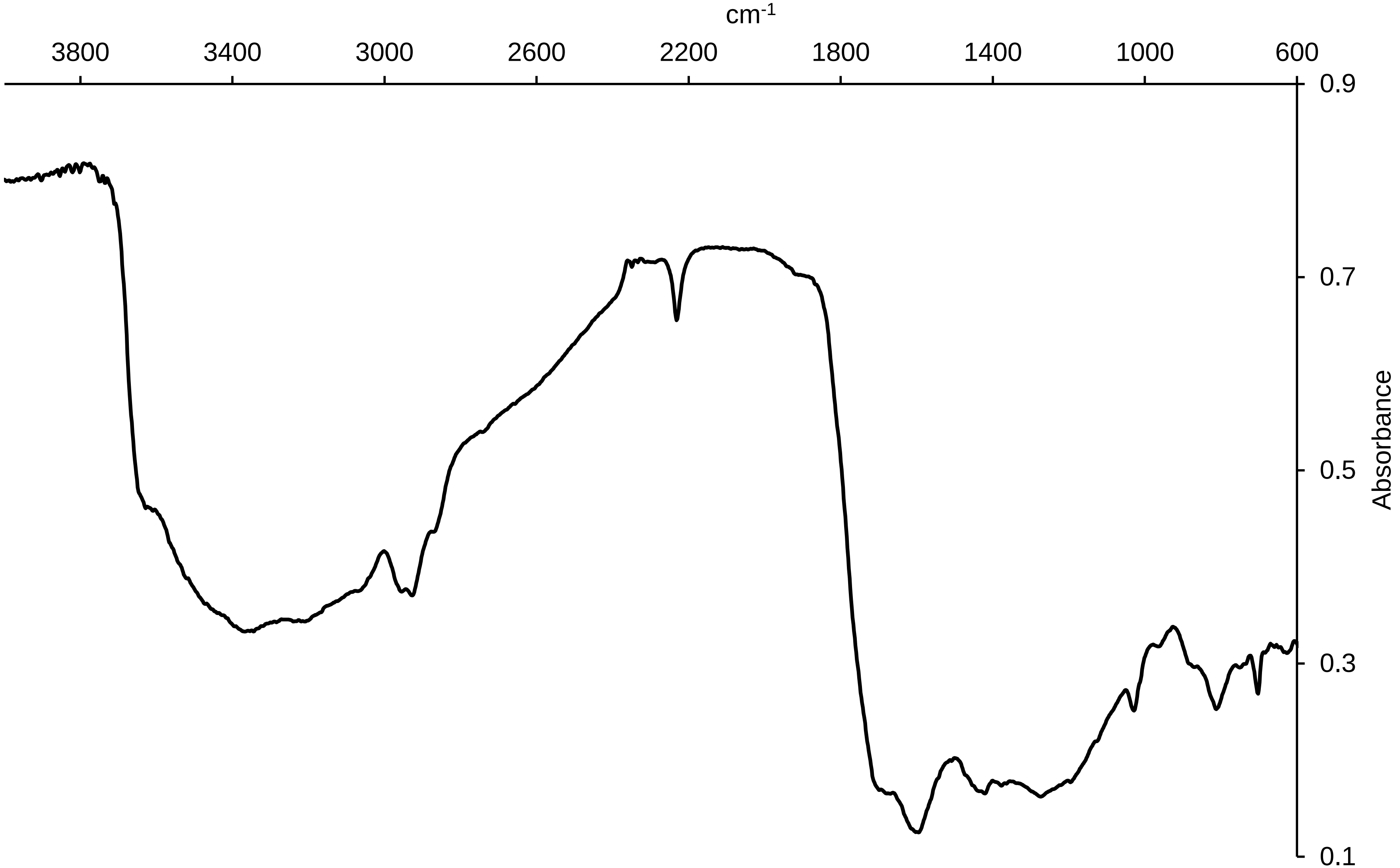

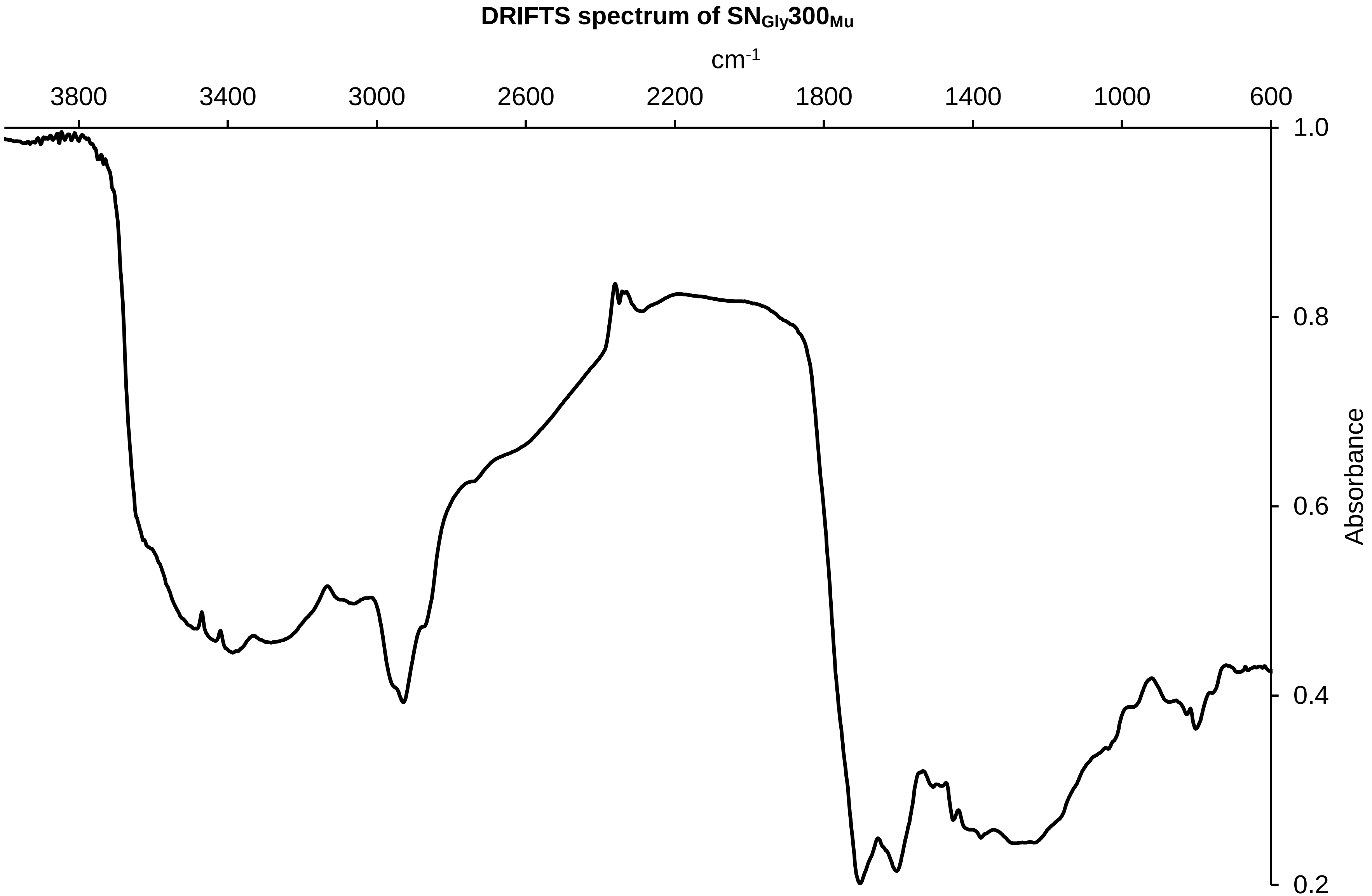
DRIFTS spectrum of $SN_{Gly}300_{Mu}$
cm-1
3800
3400
3000
2600
2200
1800
1400
1000
600
1.0
0.8
0.6
0.4
0.2
Absorbance

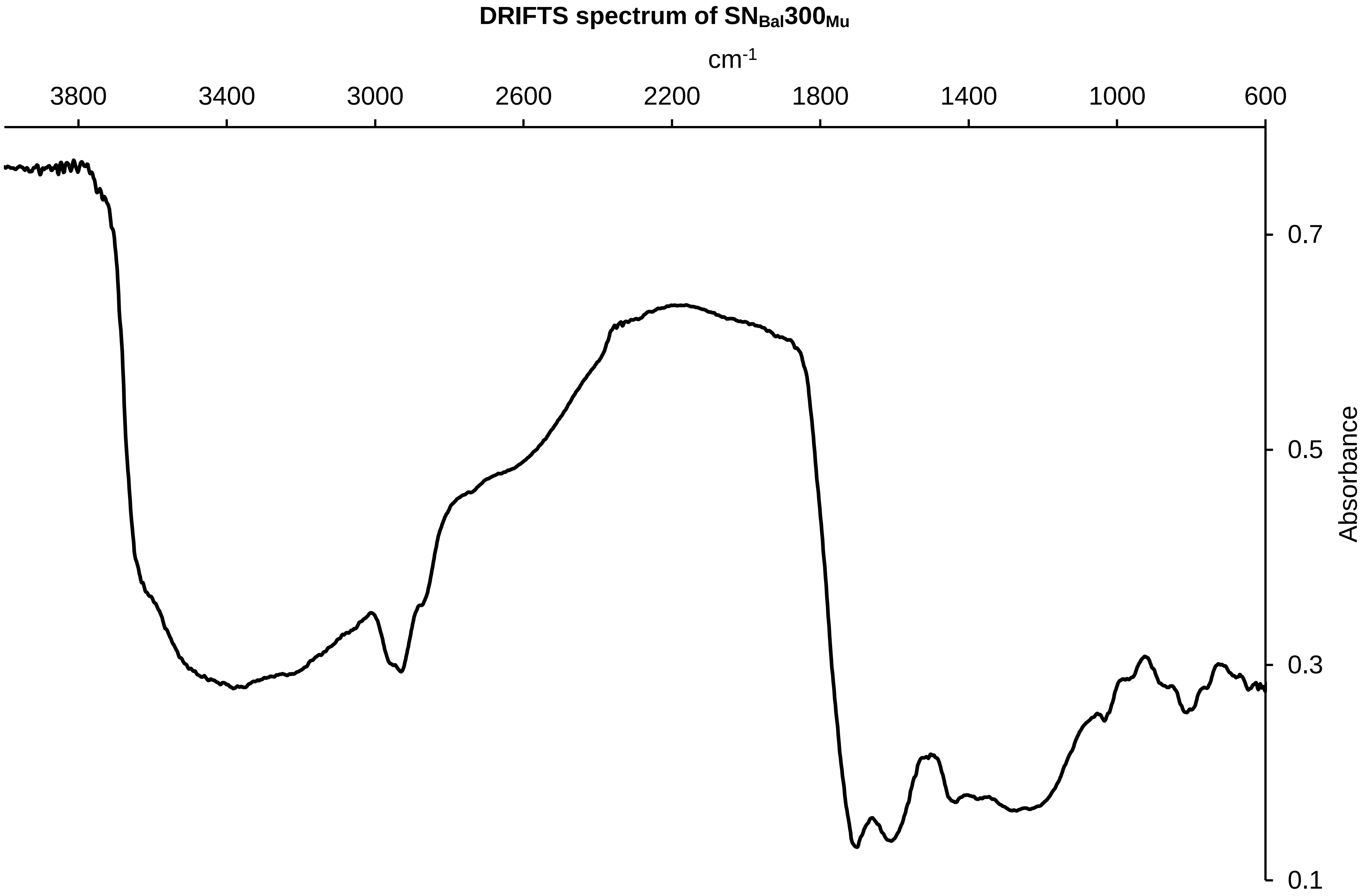
DRIFTS spectrum of $SN_{Bal}300_{Mu}$
cm-1
3800
3400
3000
2600
2200
1800
1400
1000
600
0.7
0.5
0.3
0.1
Absorbance

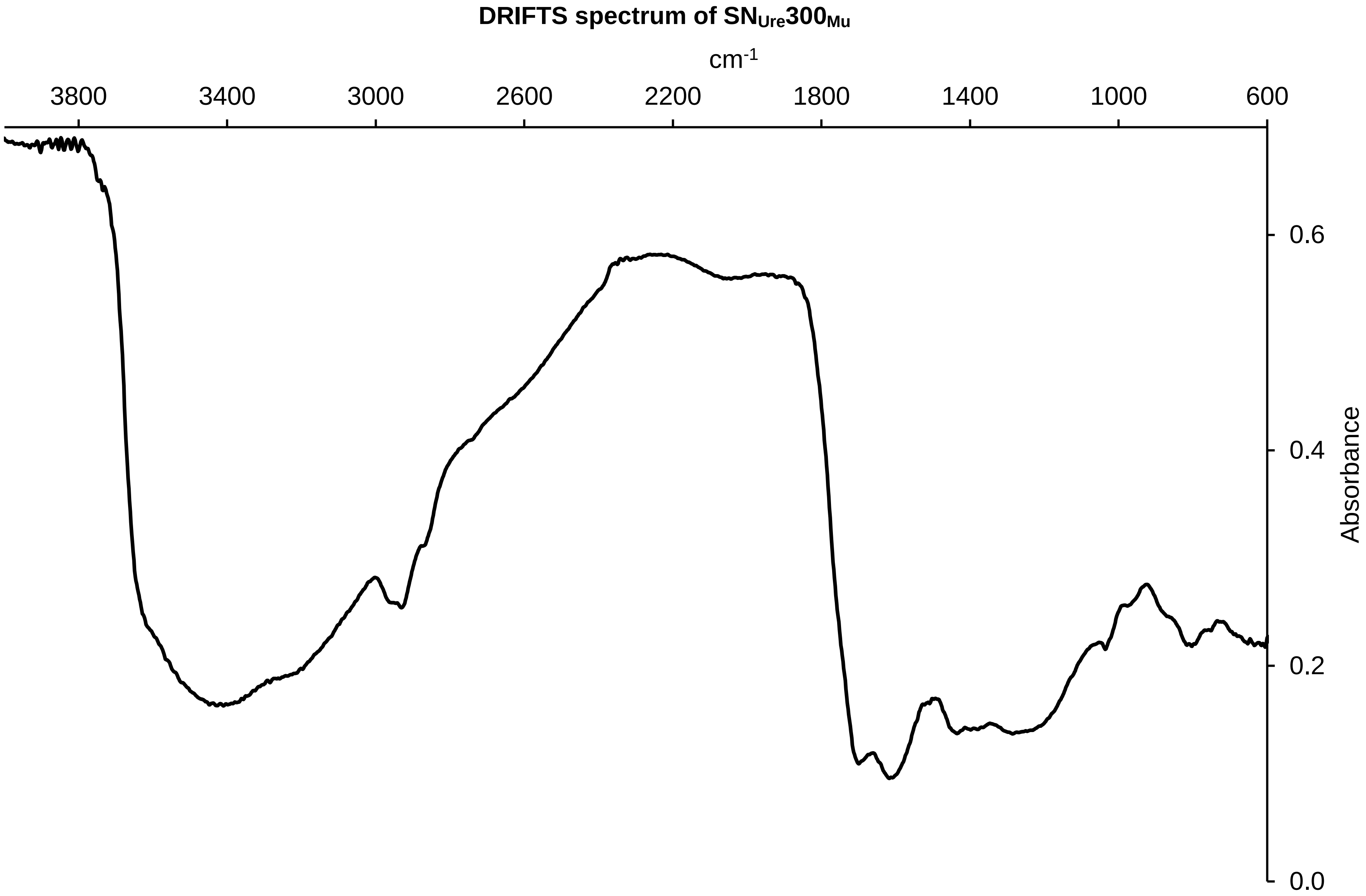

DRIFTS spectrum of SNUre300Mu
cm-1
3800
3400
3000
2600
2200
1800
1400
1000
600
0.6
0.4
0.2
0.0
Absorbance

DRIFTS spectrum of $SN_{Mel}300_{Mu}$

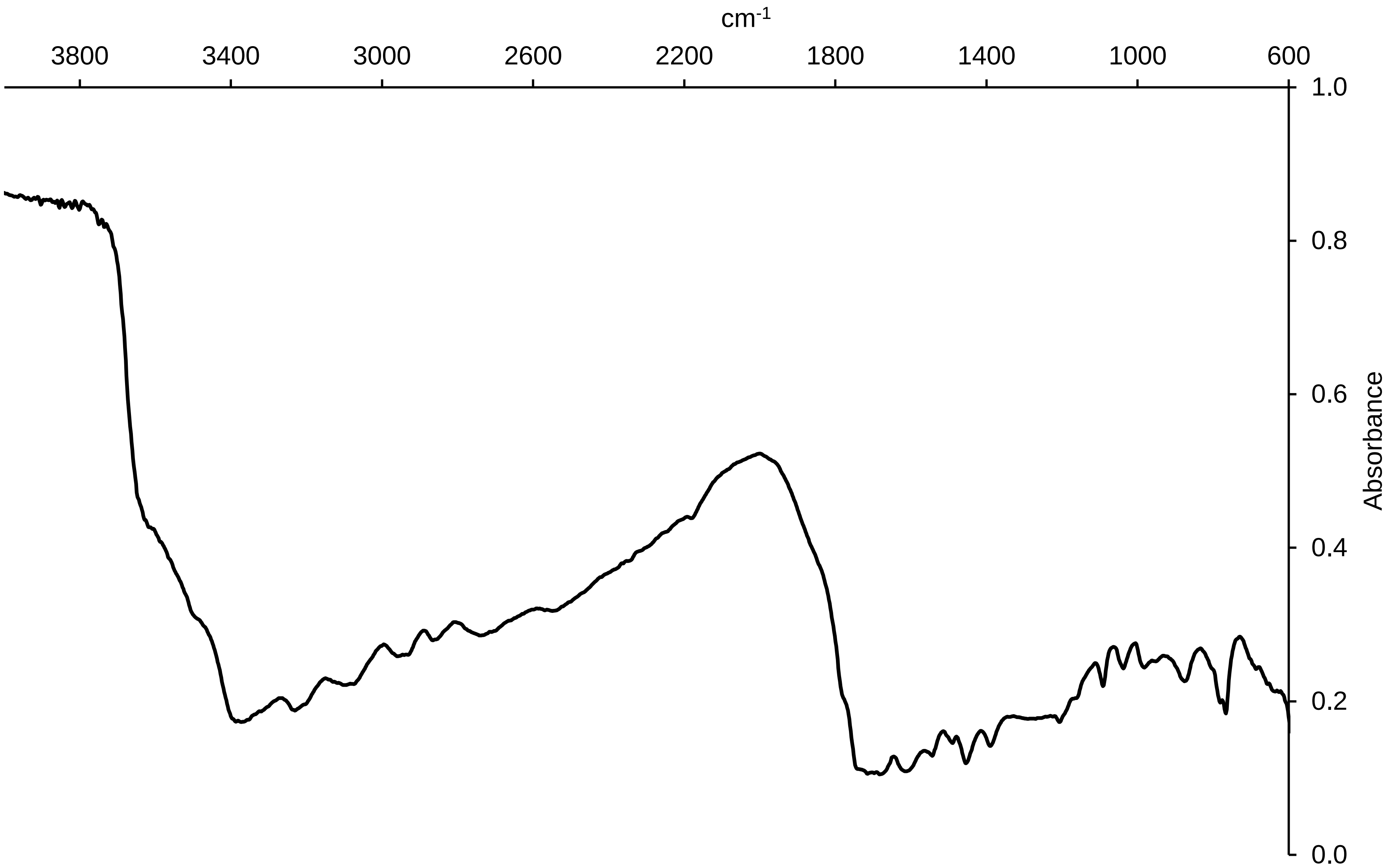

DRIFTS spectrum of $SN_{Nic}300_{Mu}$

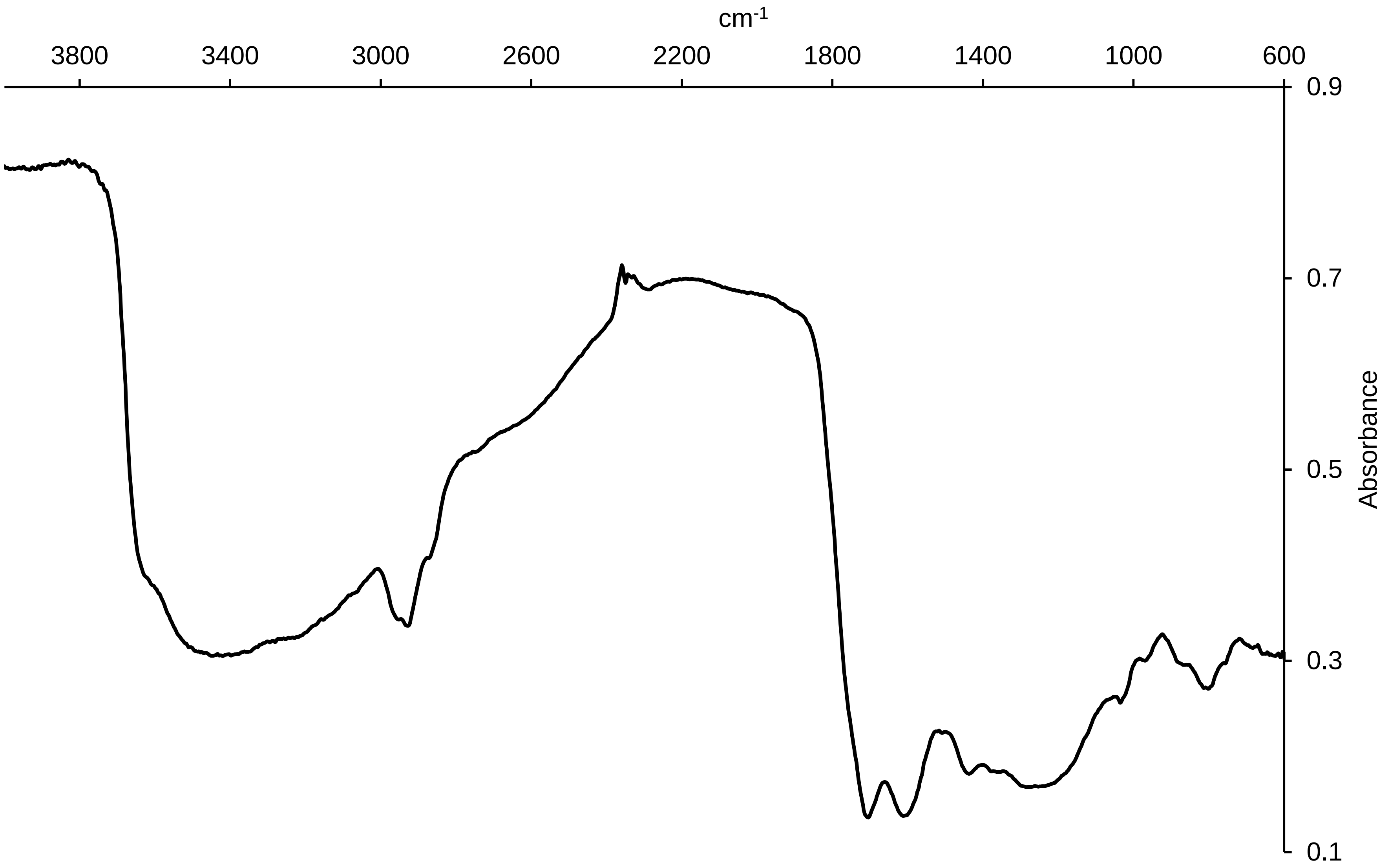

Thermogravimetric analysis of CO_2 adsorption onto S300

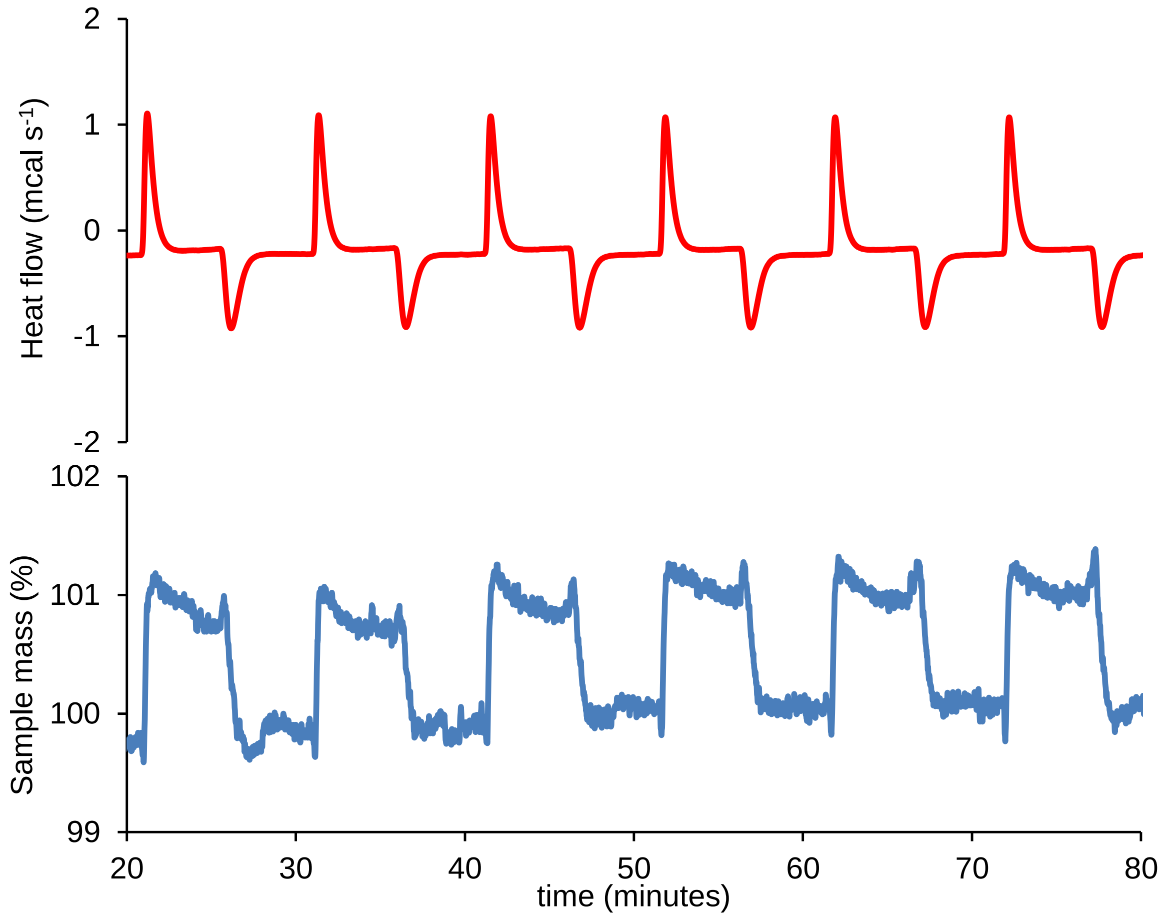

Thermogravimetric analysis of CO_2 adsorption onto S800

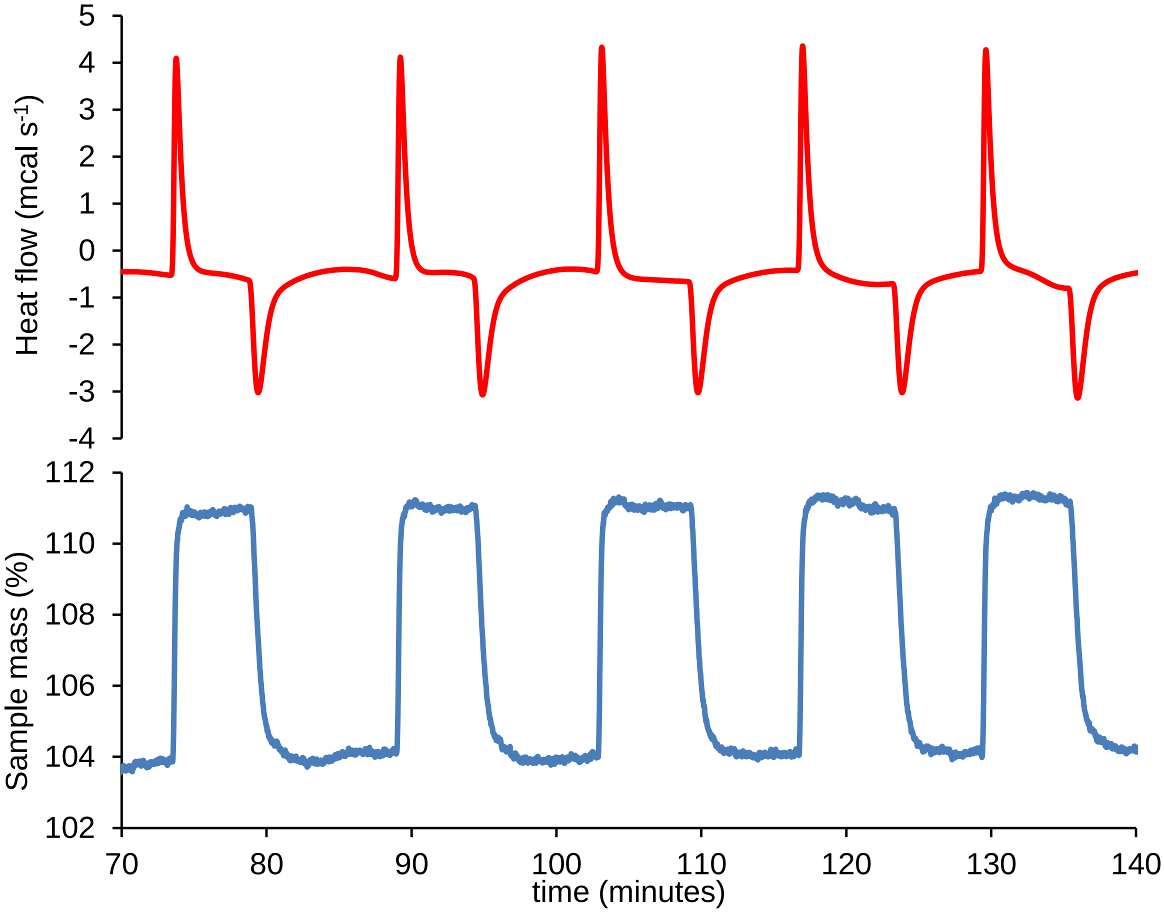

Thermogravimetric analysis of CO_2 adsorption onto $SN_{Gly}300_{Th}$

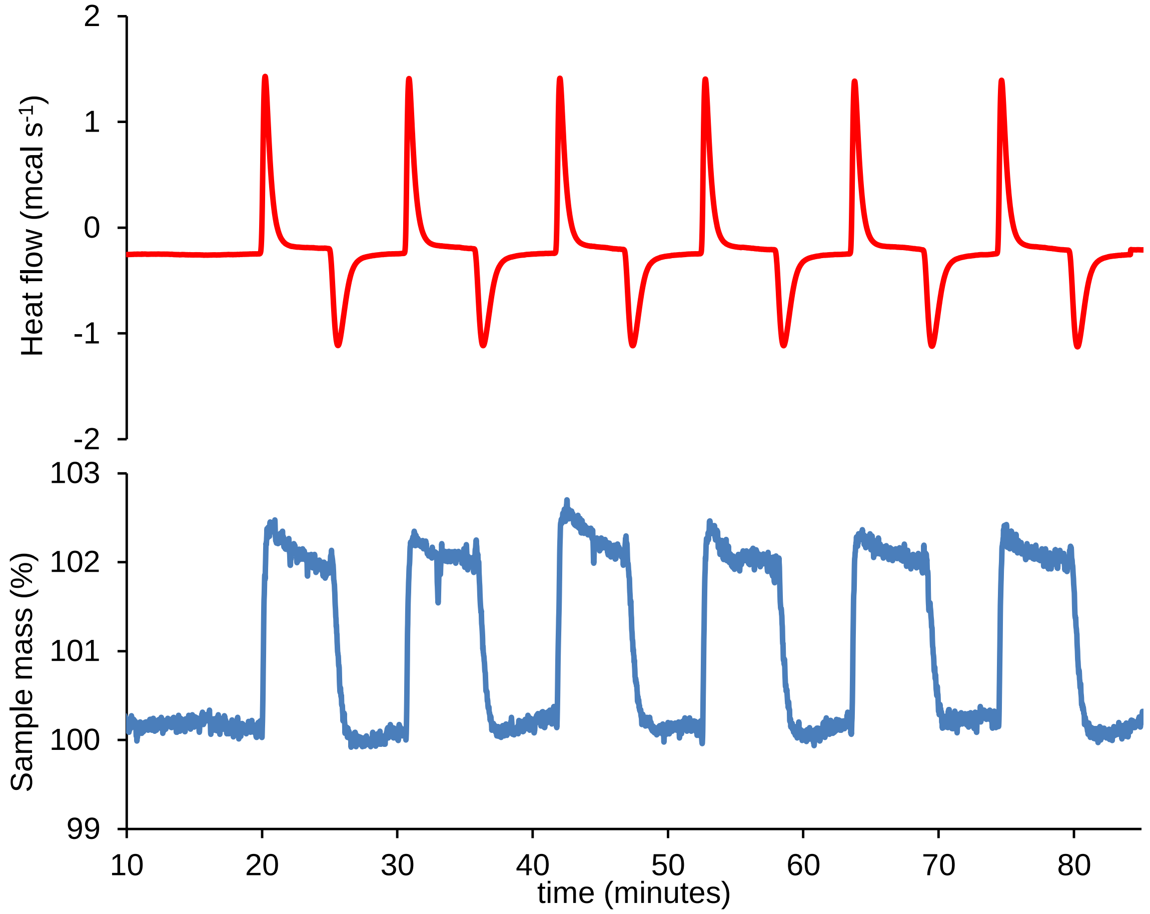

Thermogravimetric analysis of CO_2 adsorption onto $SN_{Gly}300_{Mo}$

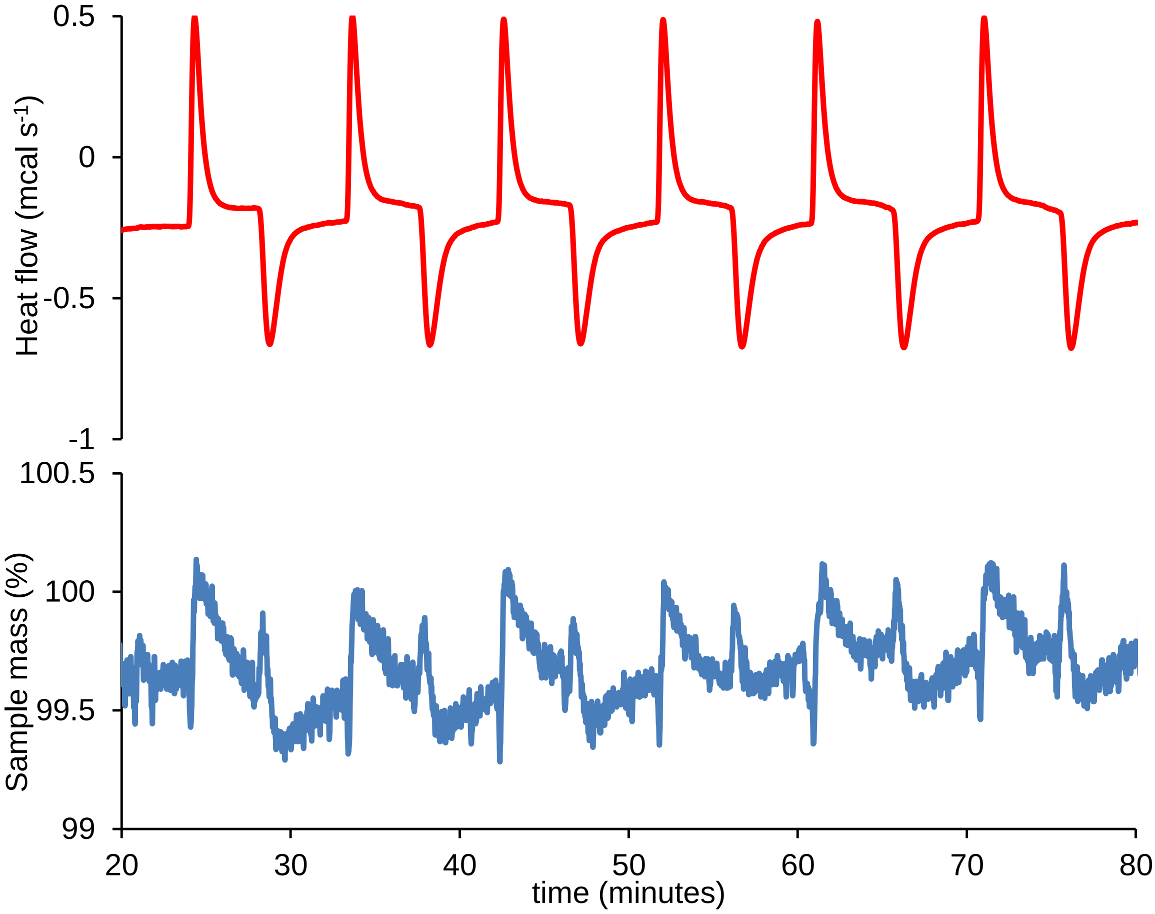

Thermogravimetric analysis of CO_2 adsorption onto $SN_{Gly}300_{Mu}$

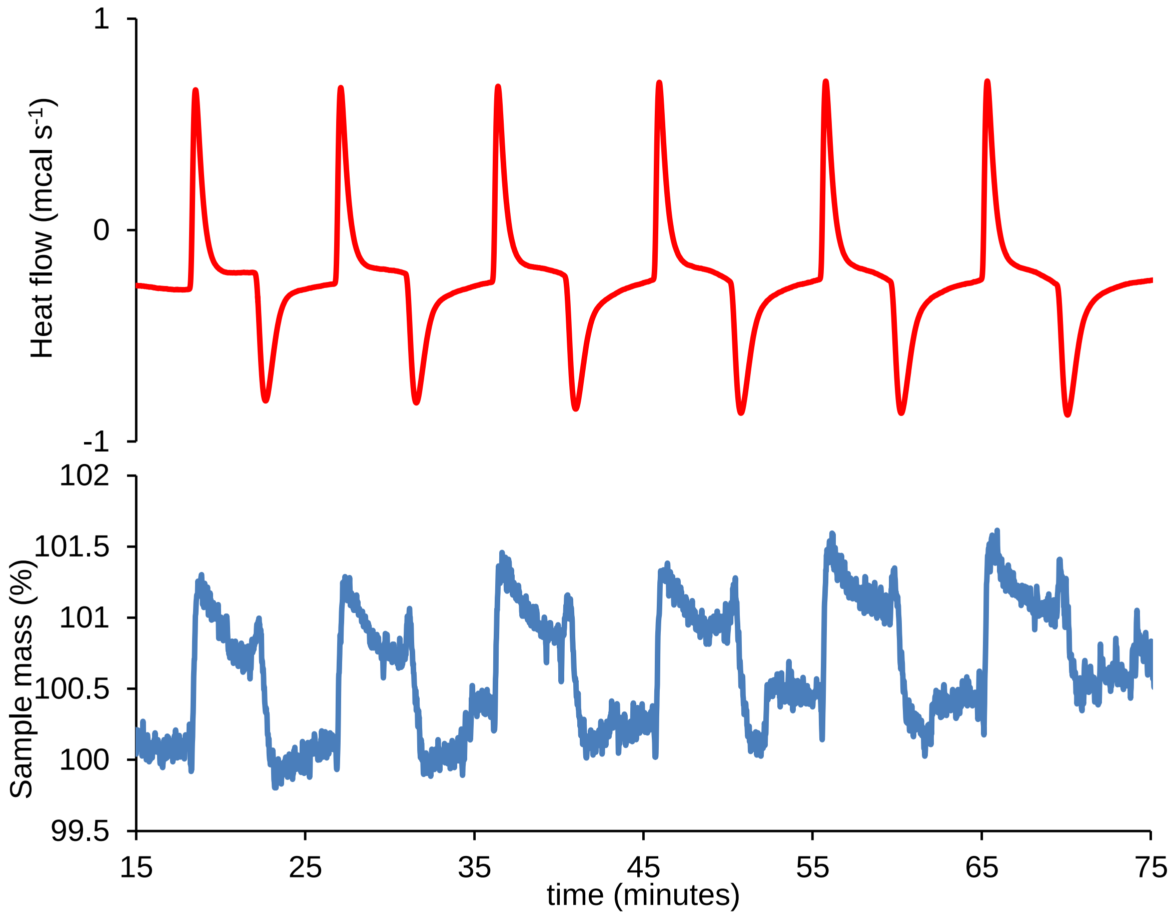

Thermogravimetric analysis of CO_2 adsorption onto $SN_{Bal}300_{Th}$

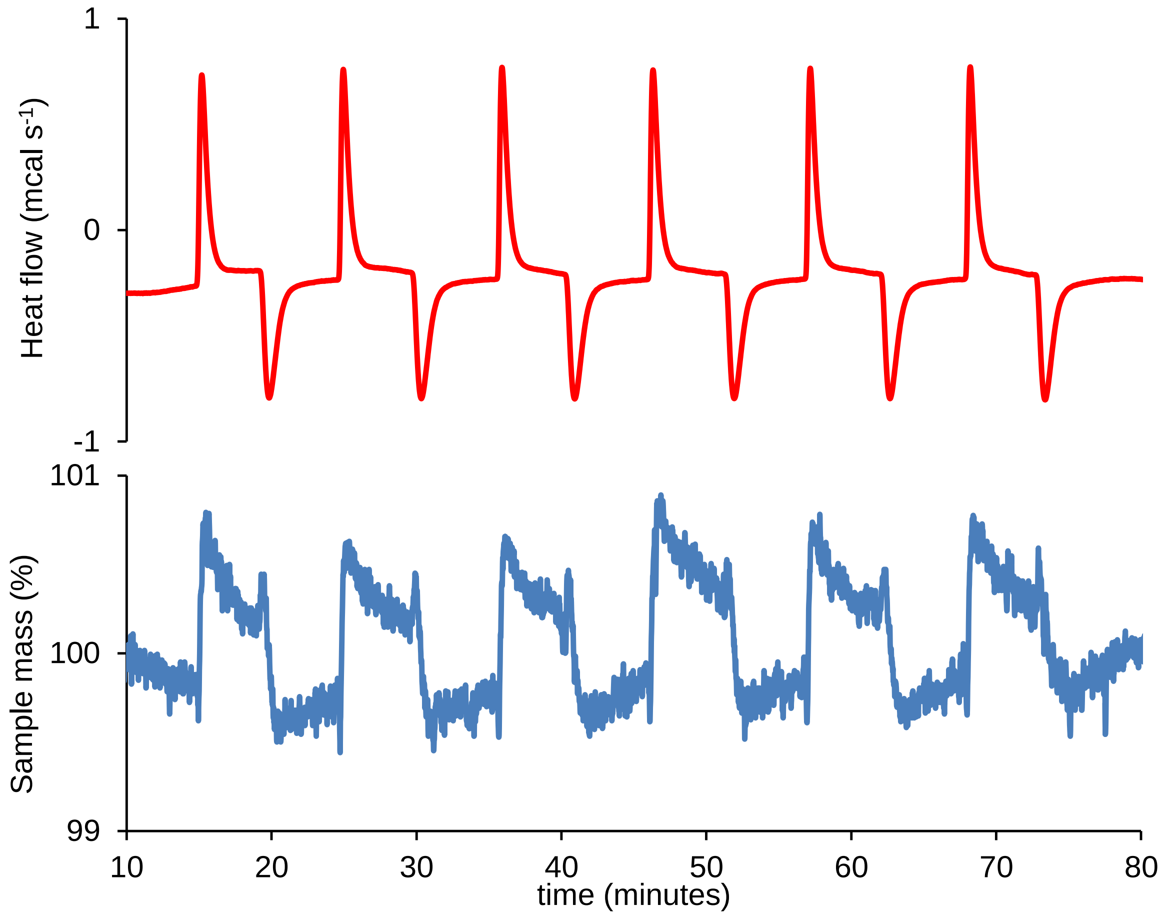

Thermogravimetric analysis of CO_2 adsorption onto $SN_{Bal}300_{Mo}$

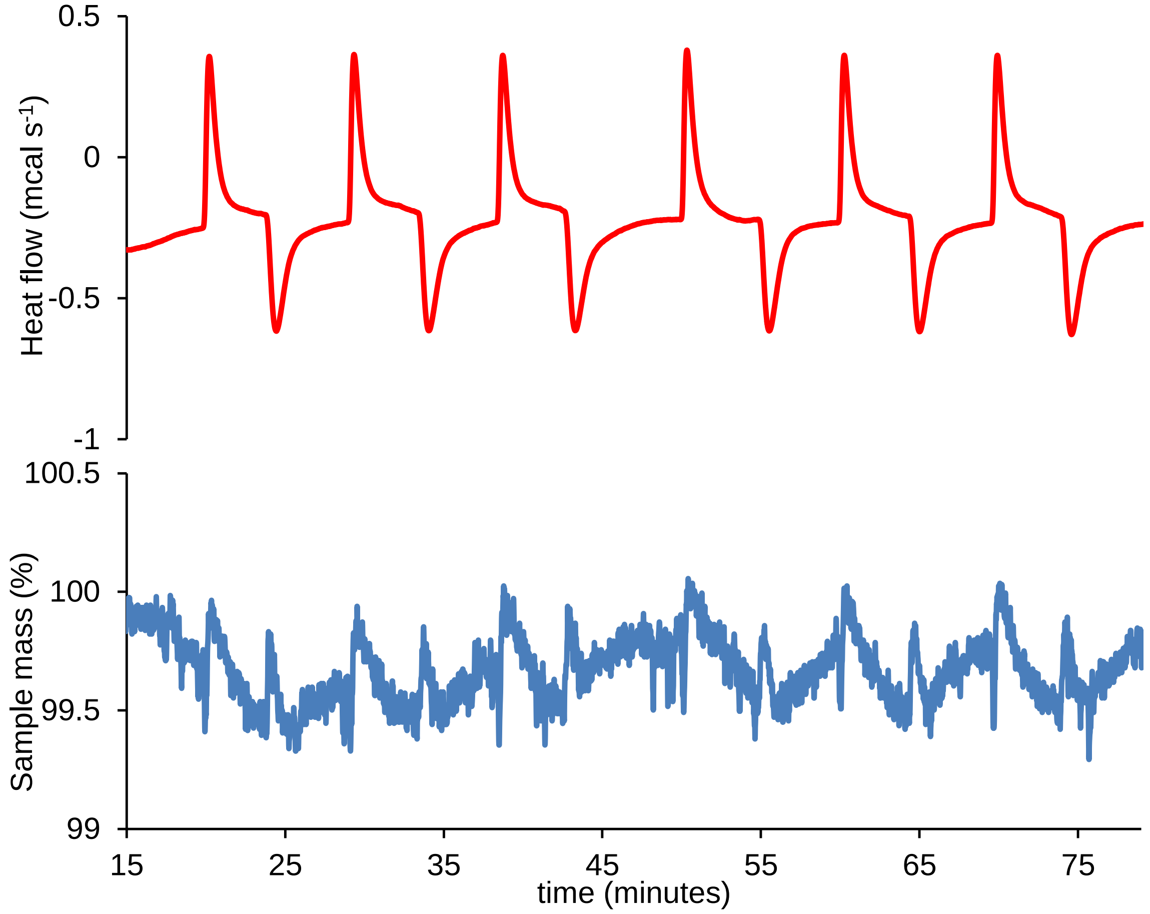

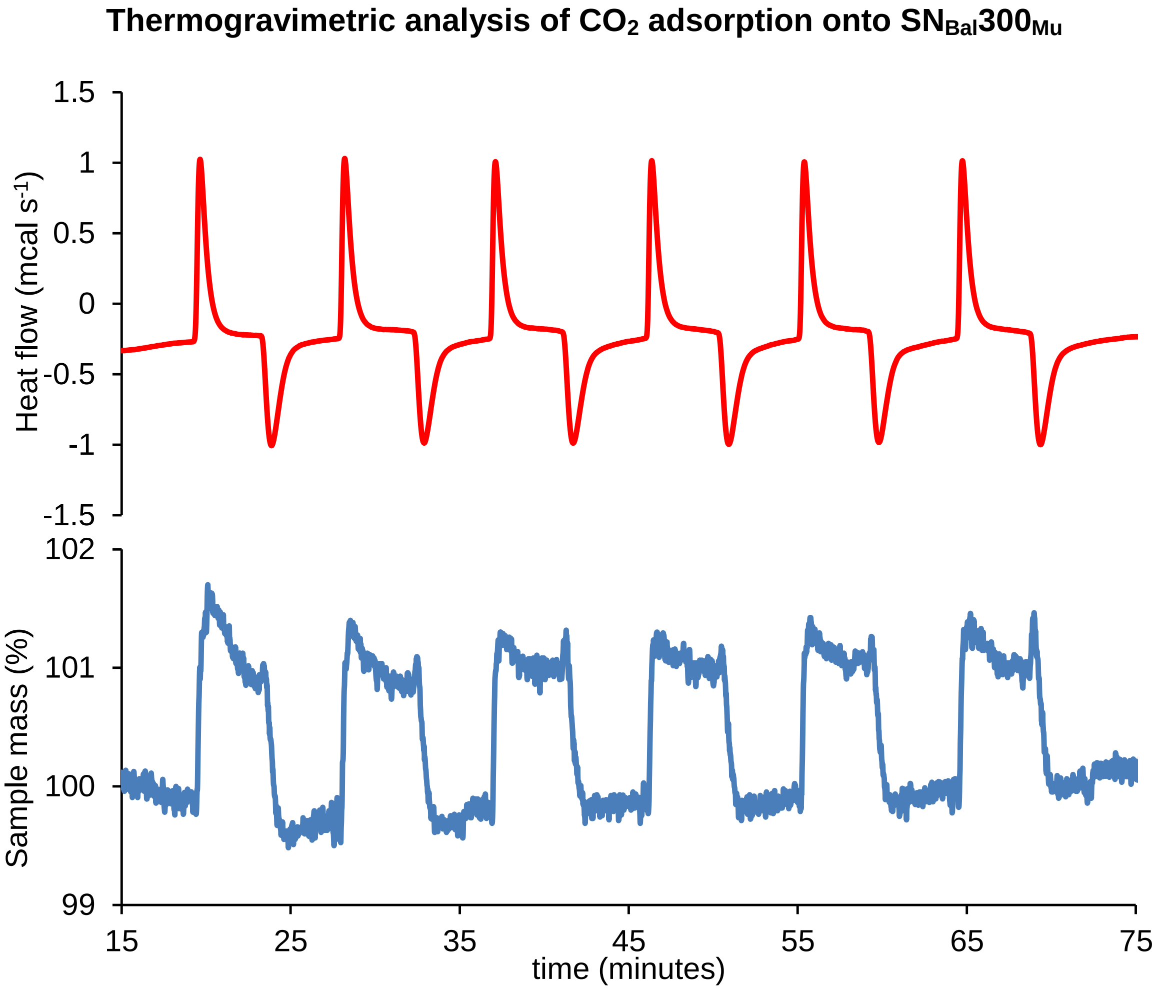

Thermogravimetric analysis of CO2 adsorption onto SNBal300Mu
Heat flow (mcal s-1)
1.5
1
0.5
0
-0.5
-1
-1.5
Sample mass (%)
102
101
100
99
15
25
35
45
55
65
75
time (minutes)

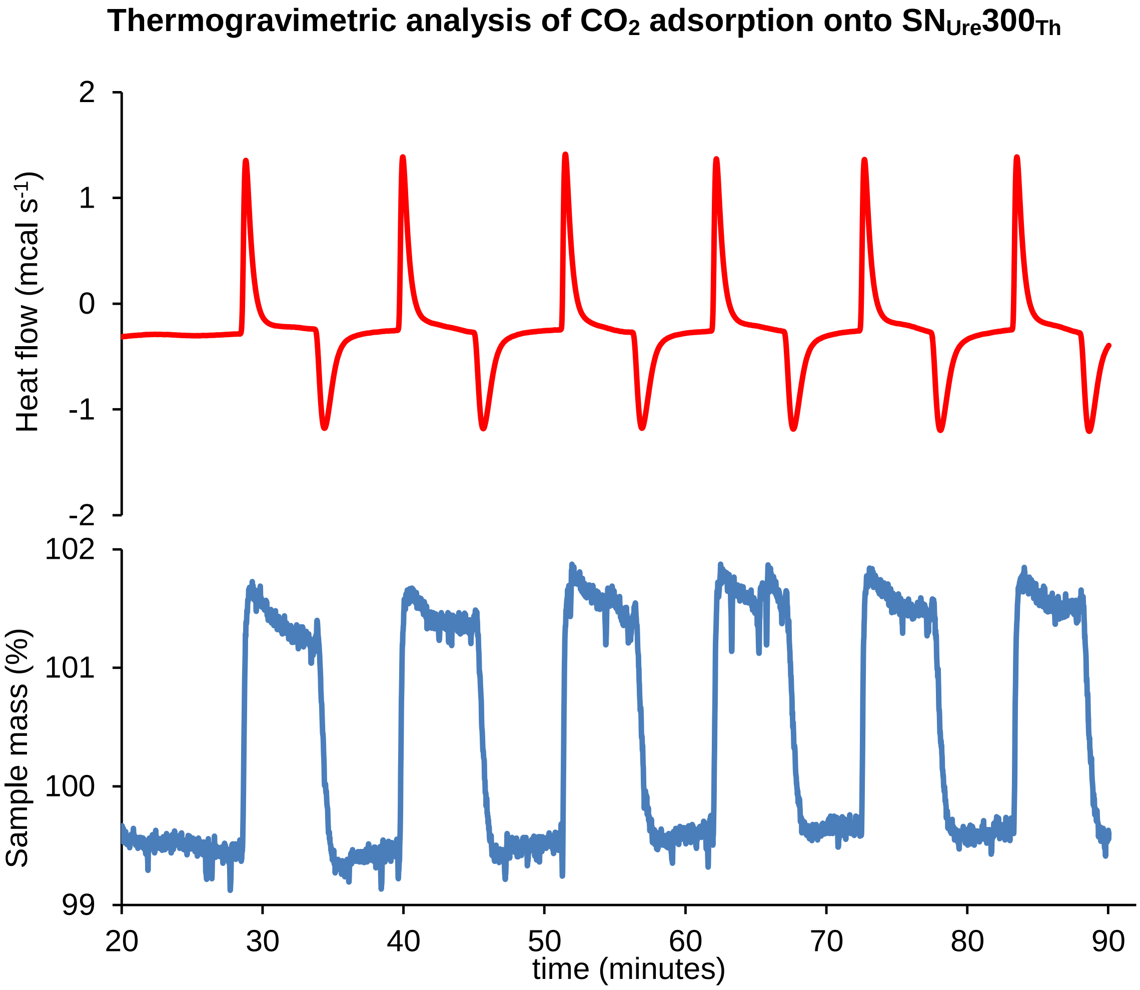
Thermogravimetric analysis of CO2 adsorption onto SNUre300Th
Heat flow (mcal s-1)
2
1
0
-1
-2
Sample mass (%)
102
101
100
99
20
30
40
50
60
70
80
90
time (minutes)

Thermogravimetric analysis of CO_2 adsorption onto $SN_{Ure}300_{Mo}$

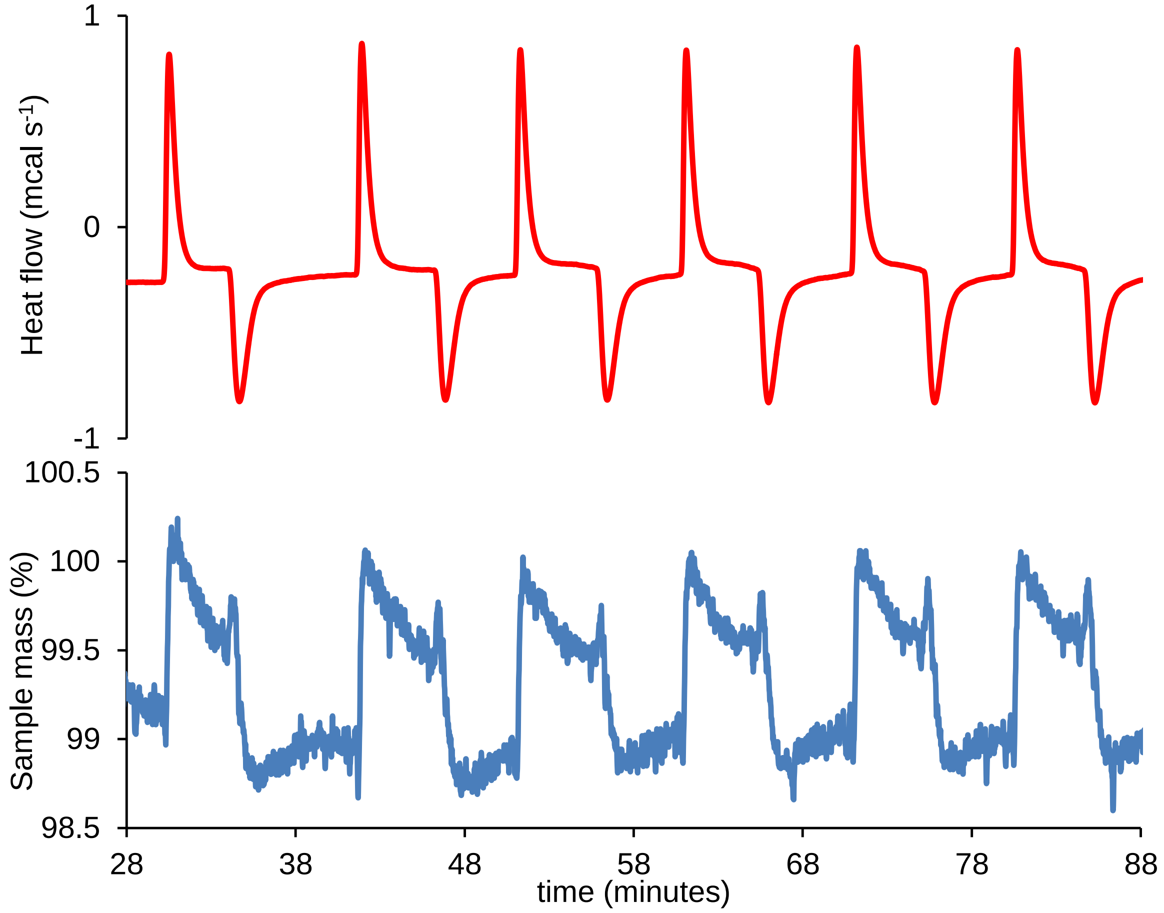

Thermogravimetric analysis of CO_2 adsorption onto $SN_{Ure}300_{Mu}$

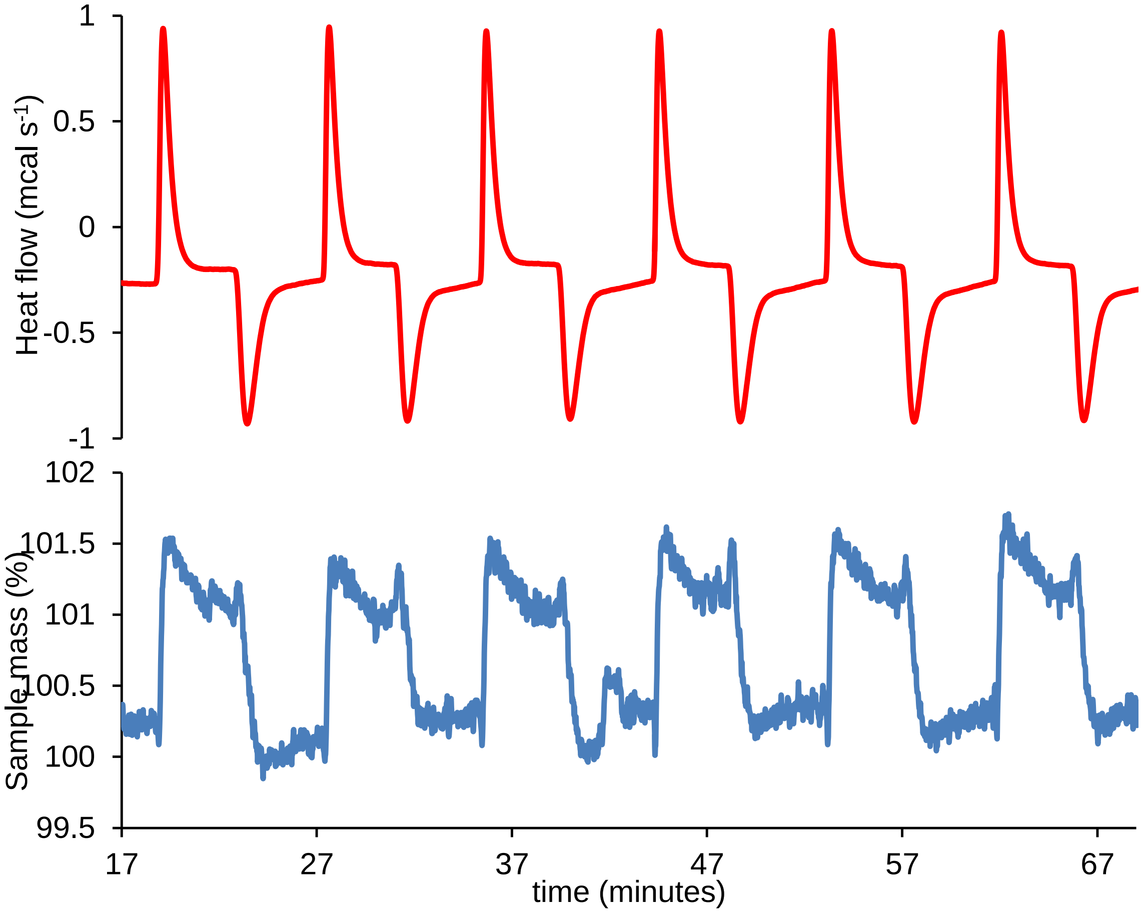

Thermogravimetric analysis of CO_2 adsorption onto $SN_{Mel}300_{Th}$

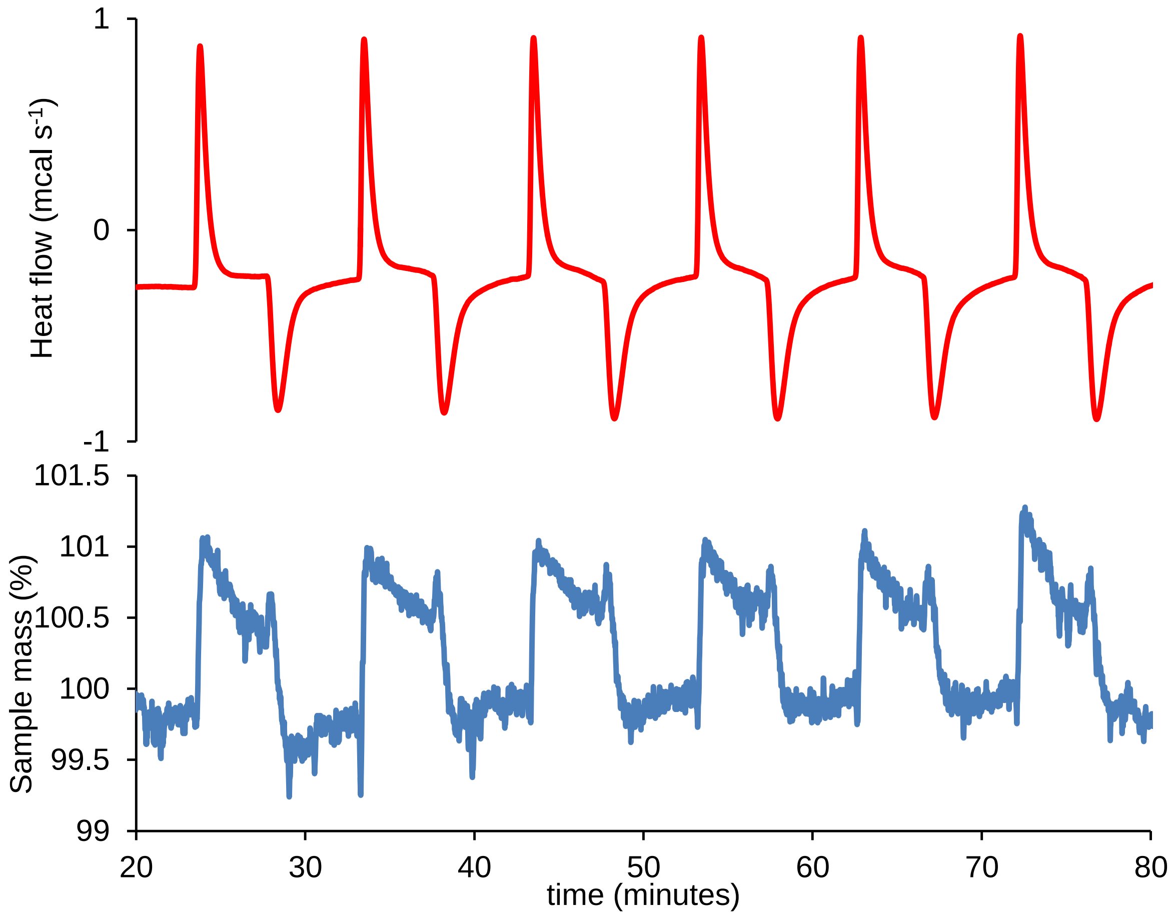

Thermogravimetric analysis of CO_2 adsorption onto $SN_{Mel}300_{Mo}$

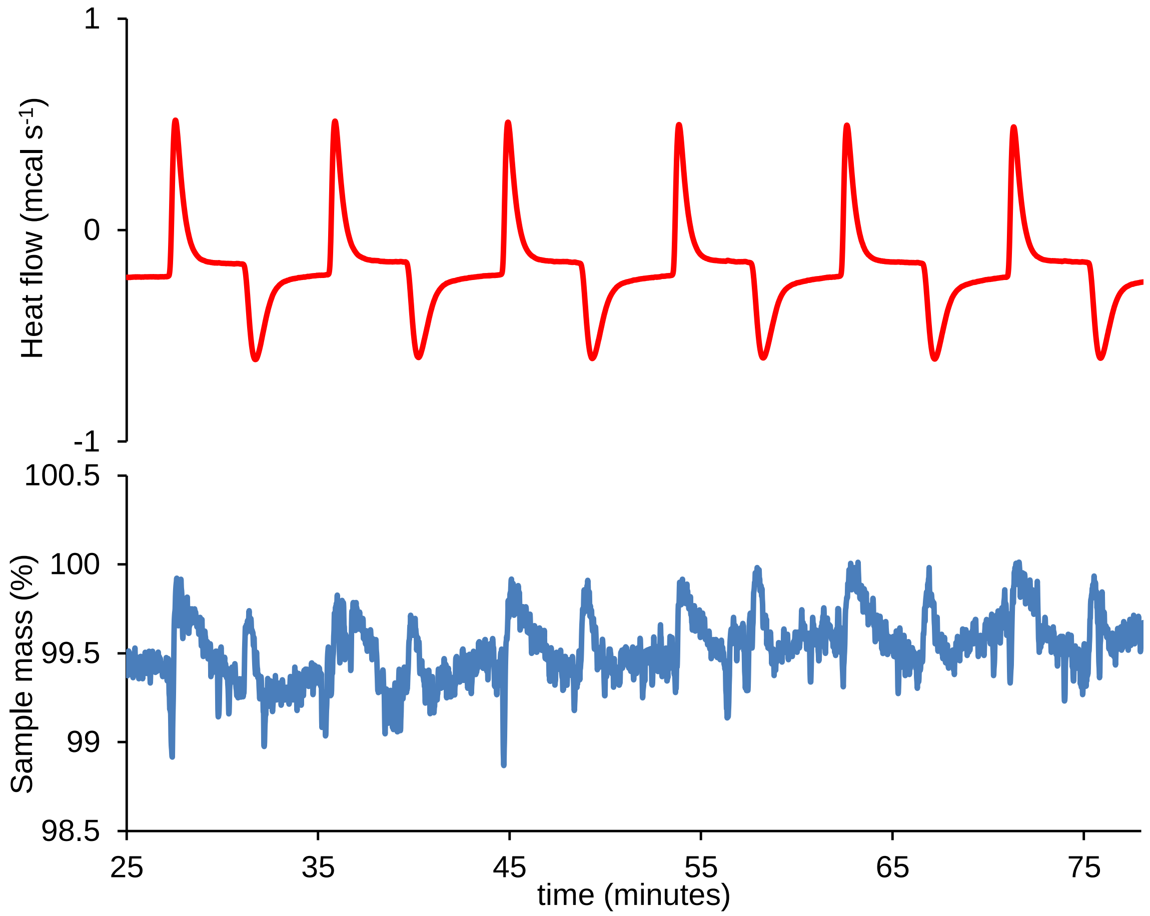

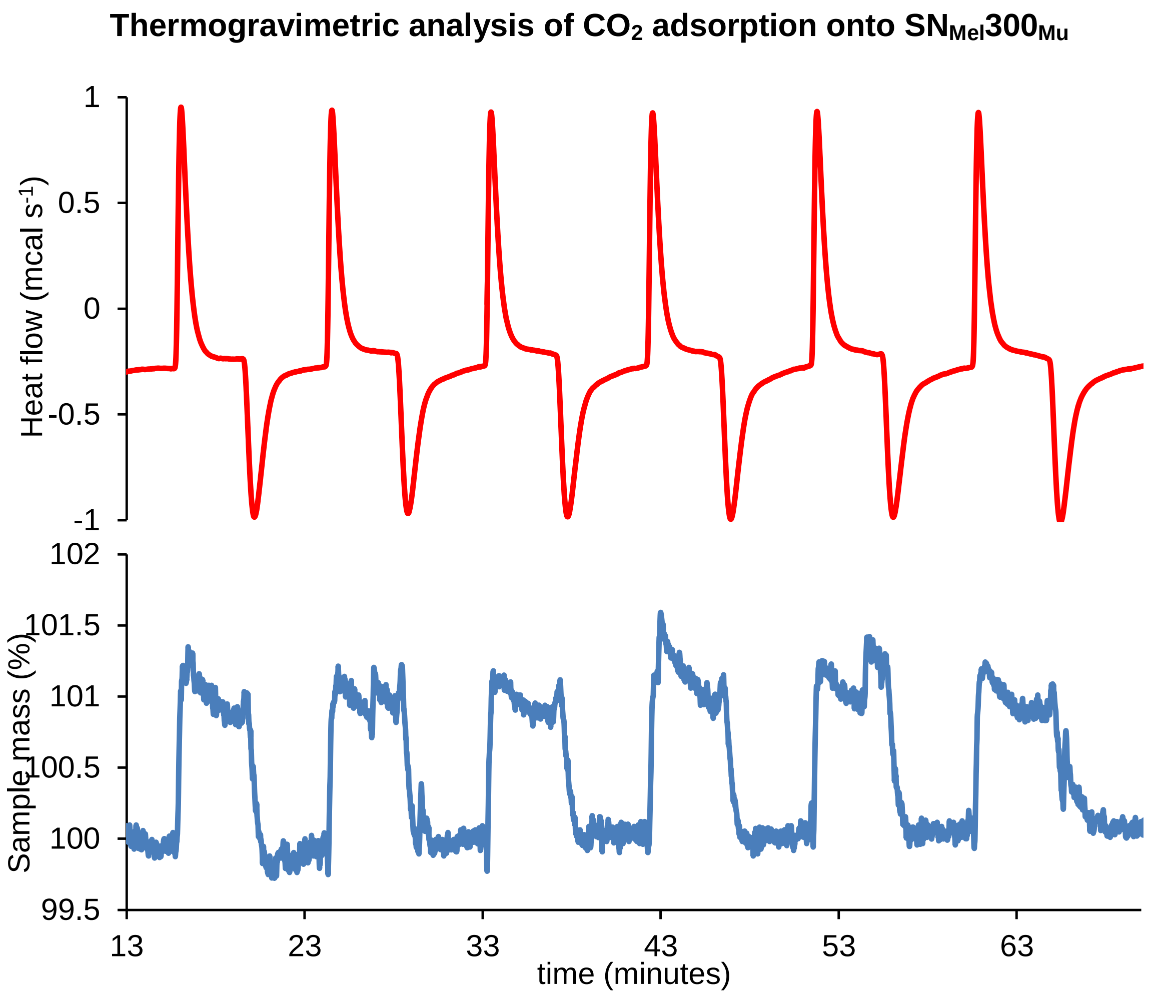
Thermogravimetric analysis of CO2 adsorption onto SNMel300Mu
Heat flow (mcal s-1)
1
0.5
0
-0.5
-1
Sample mass (%)
102
101.5
101
100.5
100
99.5
13
23
33
43
53
63
time (minutes)

Thermogravimetric analysis of CO_2 adsorption onto $SN_{Nic}300_{Th}$

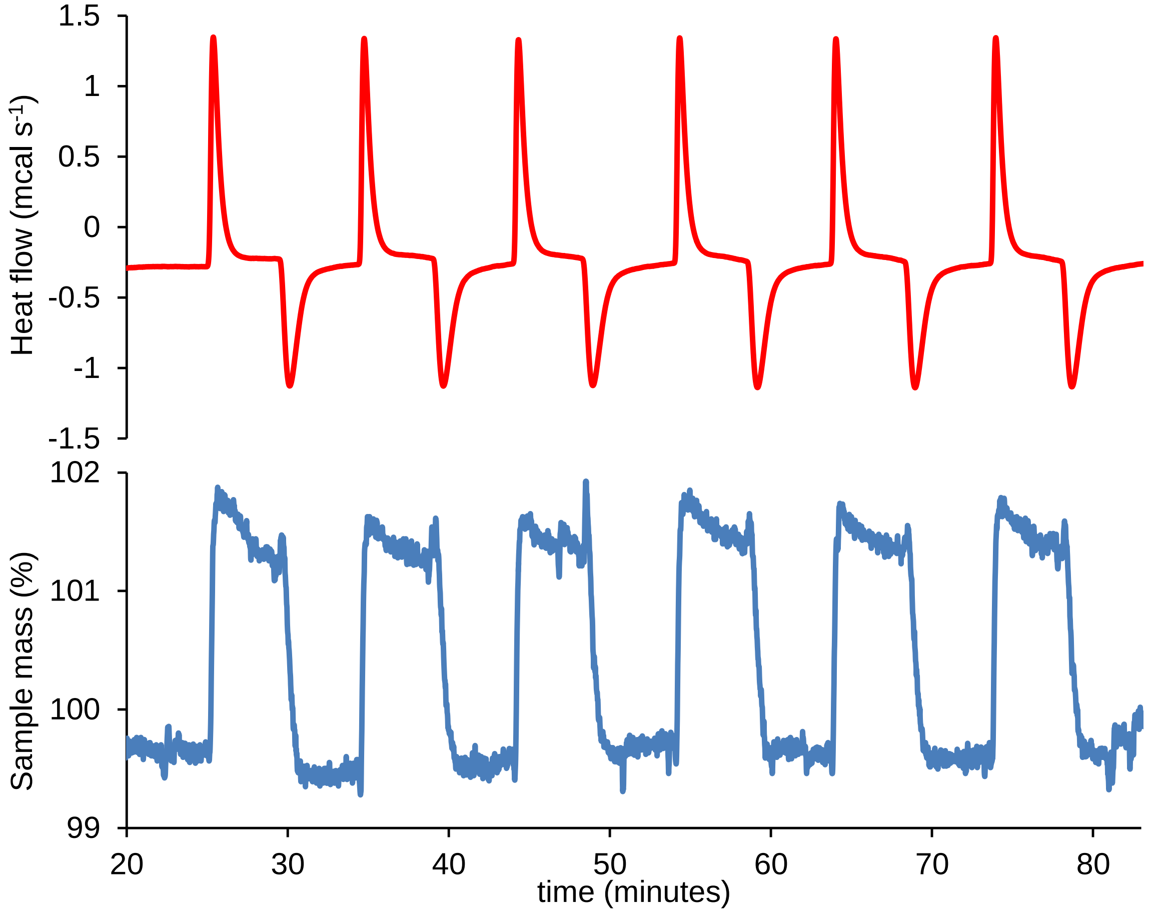

Thermogravimetric analysis of CO_2 adsorption onto $SN_{Nic}300_{Mo}$

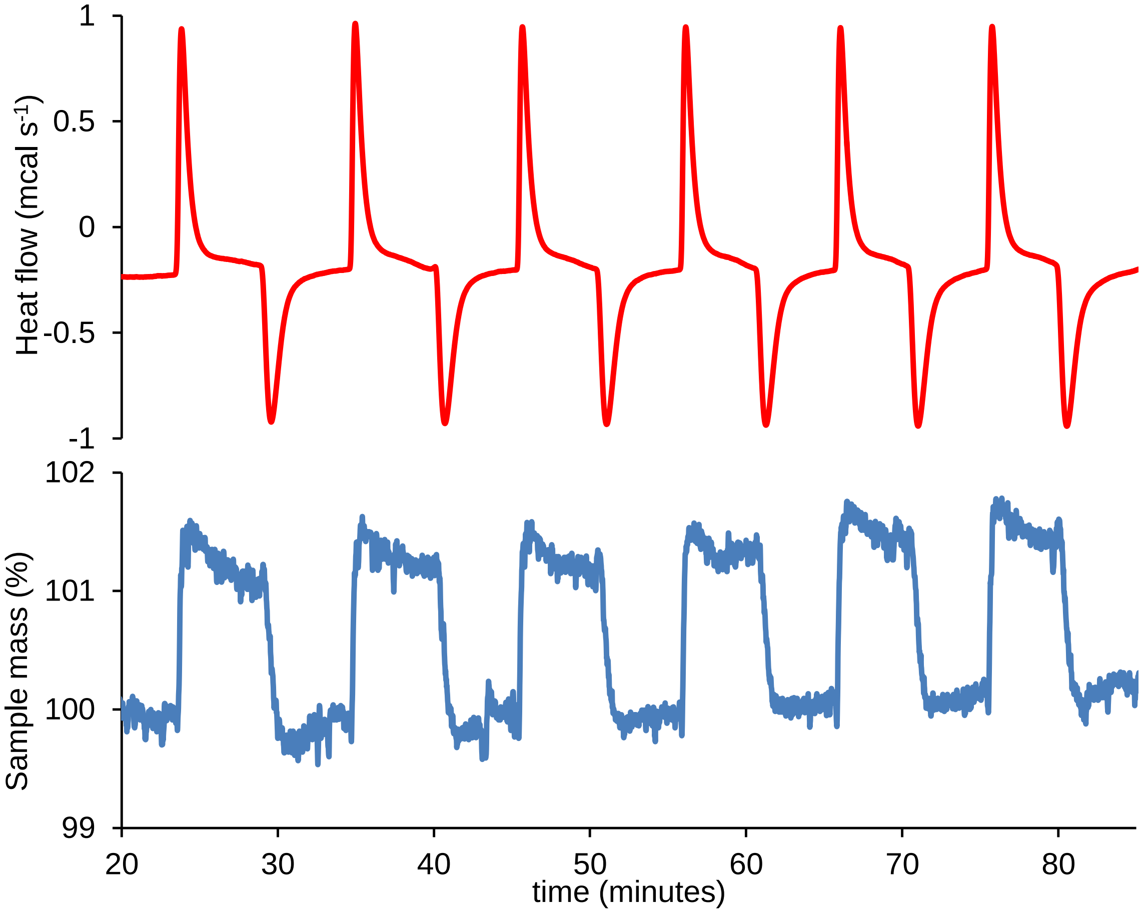

Thermogravimetric analysis of CO_2 adsorption onto $SN_{Nic}300_{Mu}$

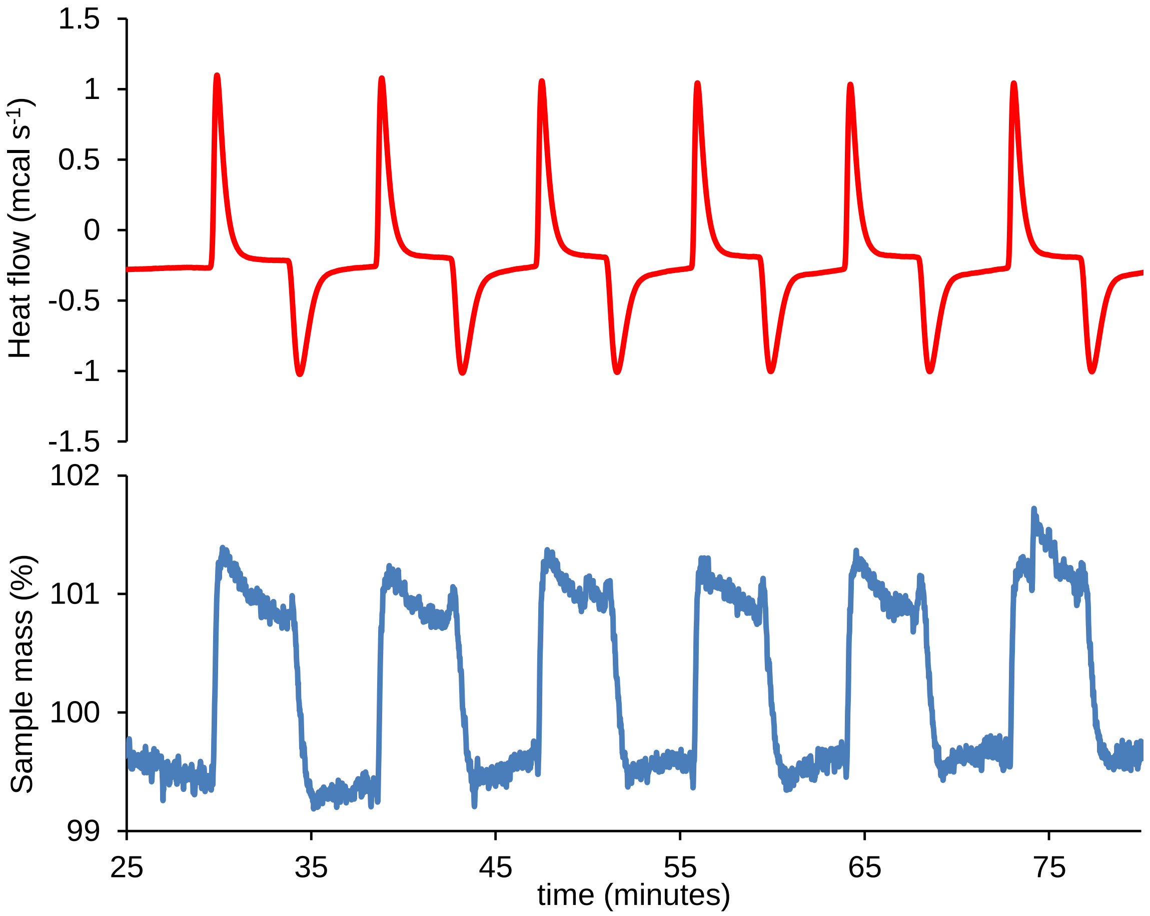

Thermogravimetric analysis of CO_2 adsorption onto $SN_{Gly}800_{Th}$

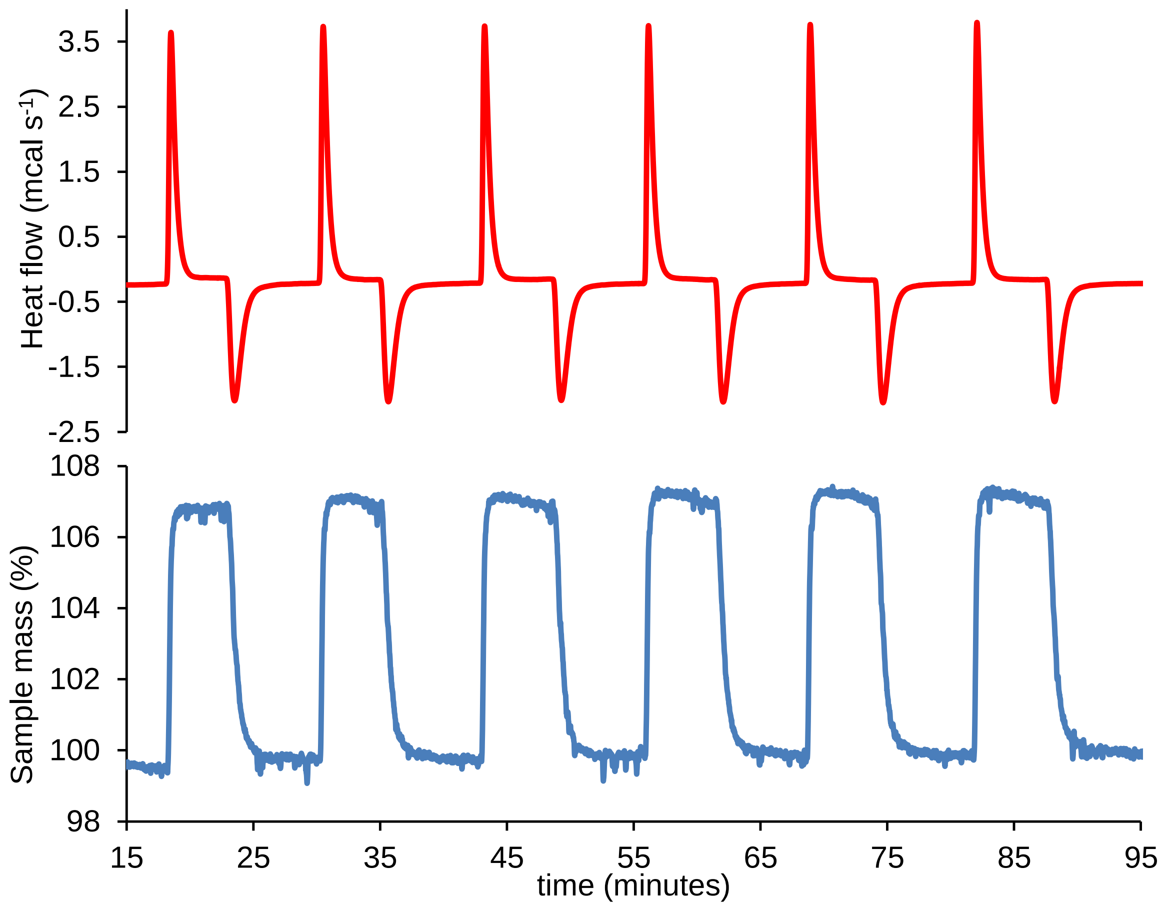

Thermogravimetric analysis of CO_2 adsorption onto $SN_{Gly}800_{Mo}$

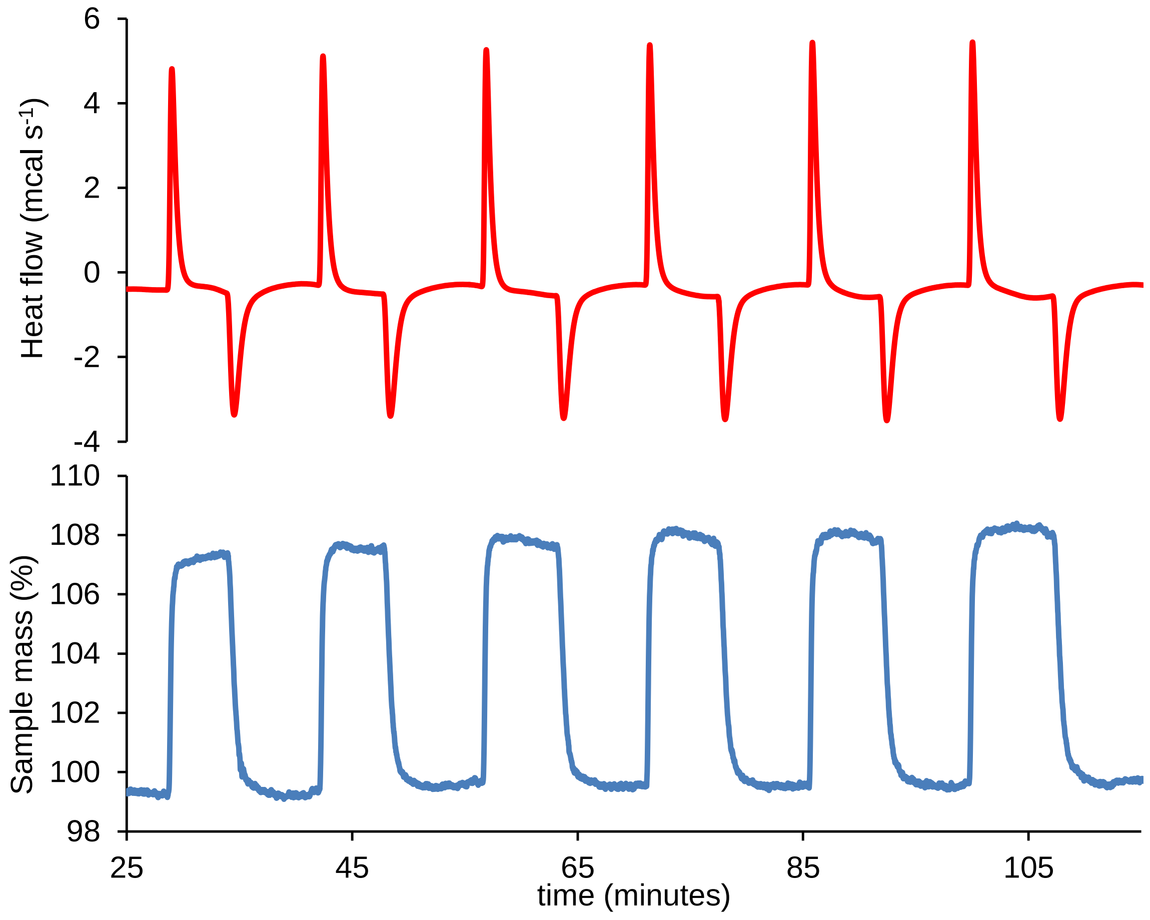

Thermogravimetric analysis of CO_2 adsorption onto $SN_{Gly}800_{Mu}$

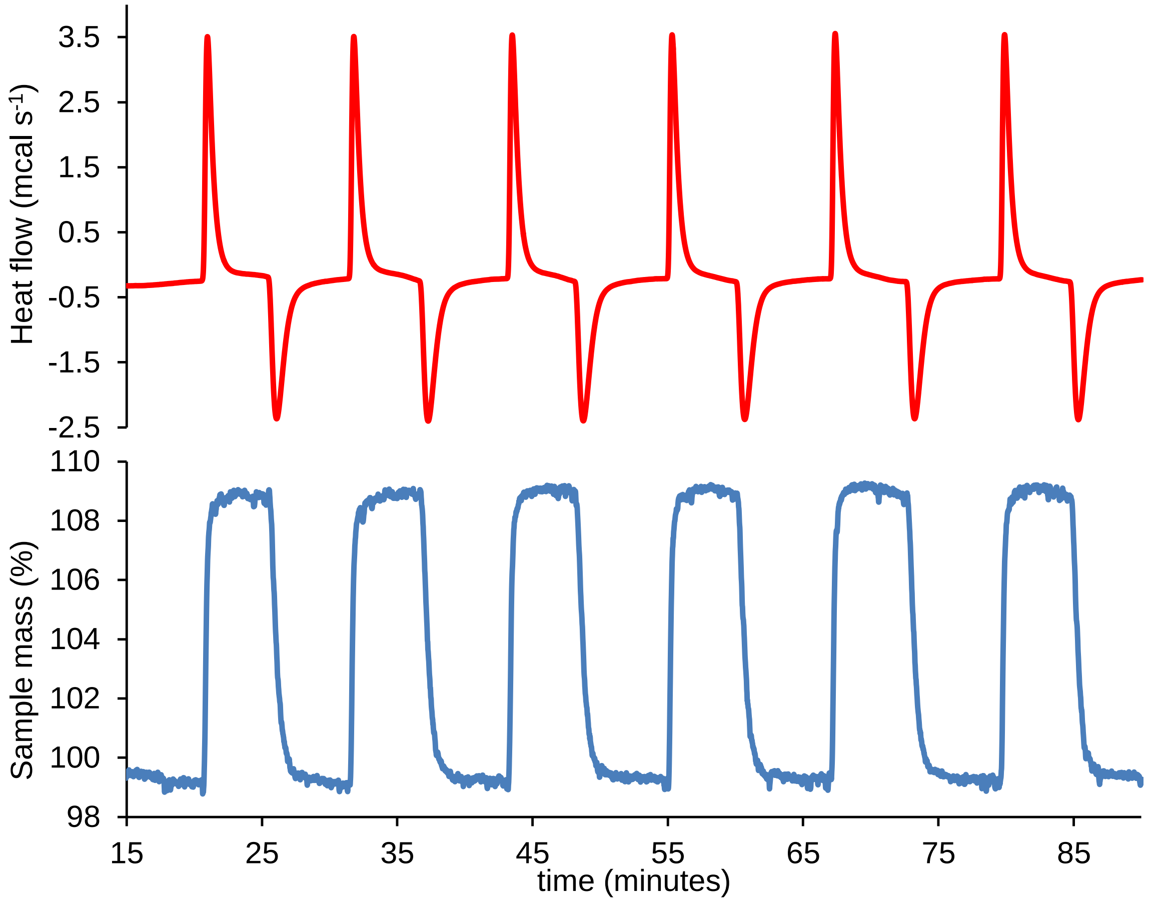

Thermogravimetric analysis of CO_2 adsorption onto $SN_{Bal}800_{Th}$

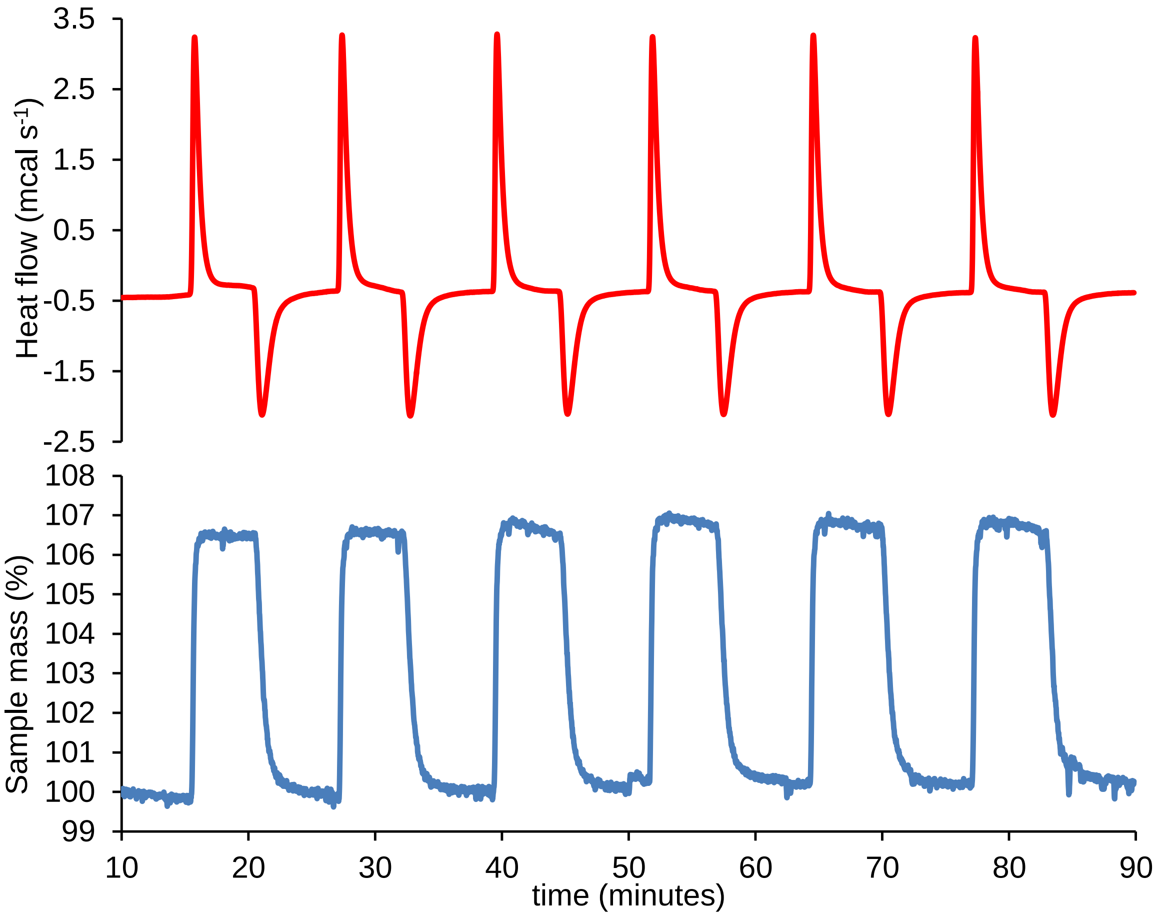

Thermogravimetric analysis of CO_2 adsorption onto $SN_{Bal}800_{Mo}$

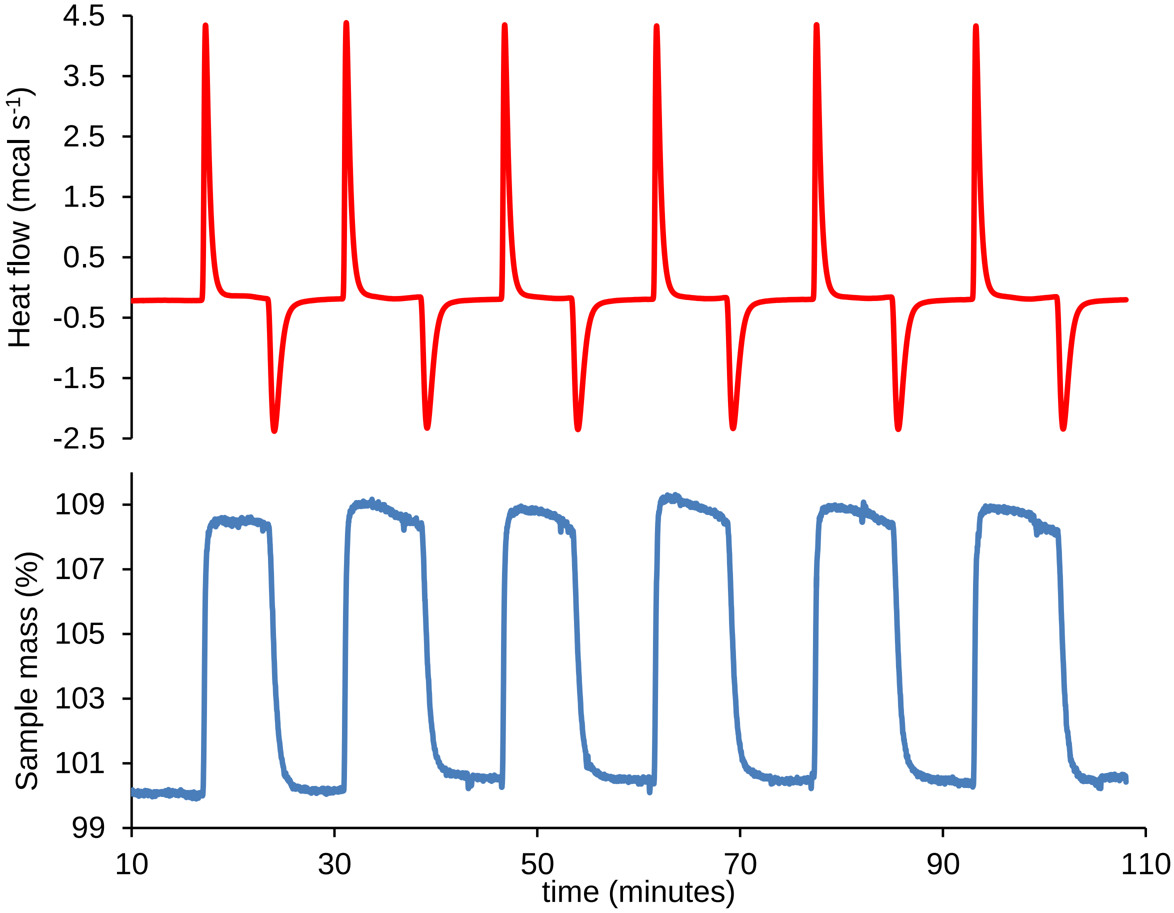

Thermogravimetric analysis of CO_2 adsorption onto $SN_{Bal}800_{Mu}$

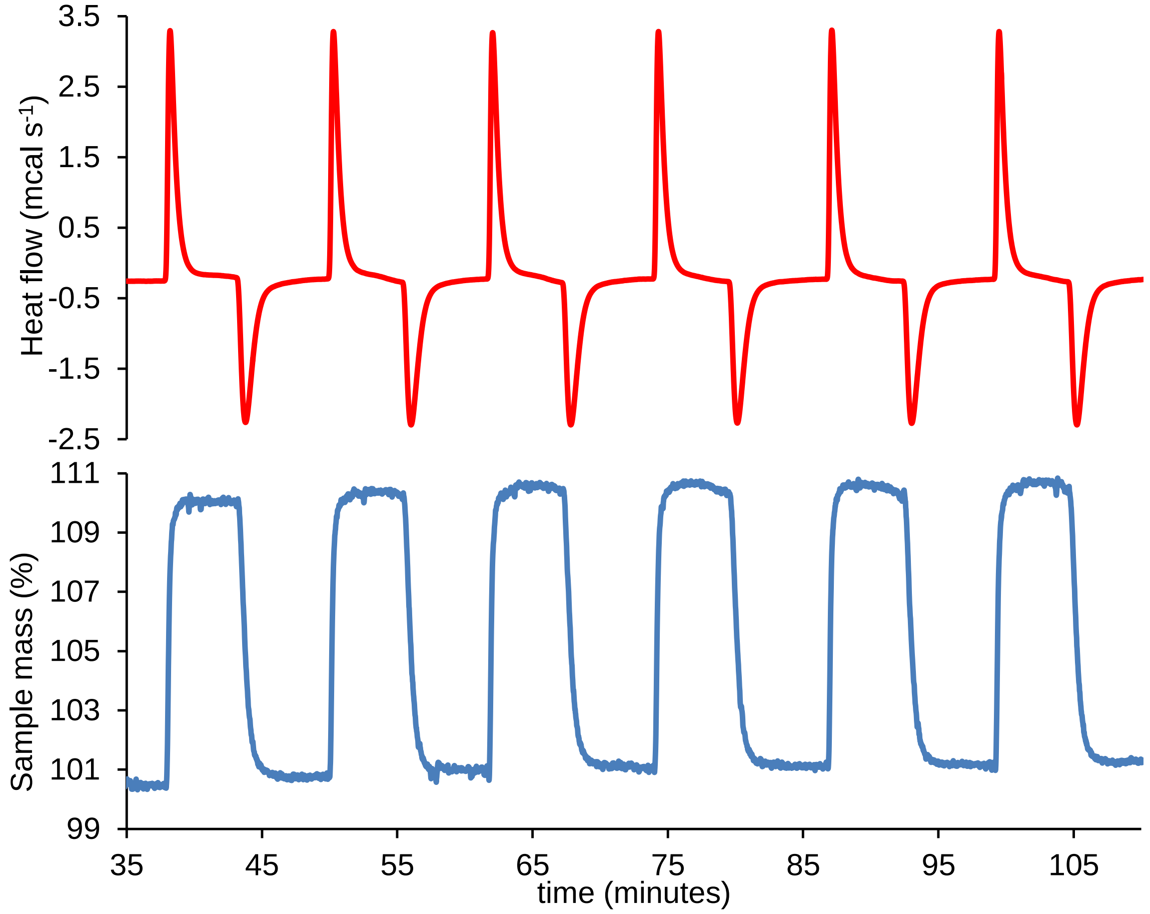

Thermogravimetric analysis of CO_2 adsorption onto $SN_{Ure}800_{Th}$

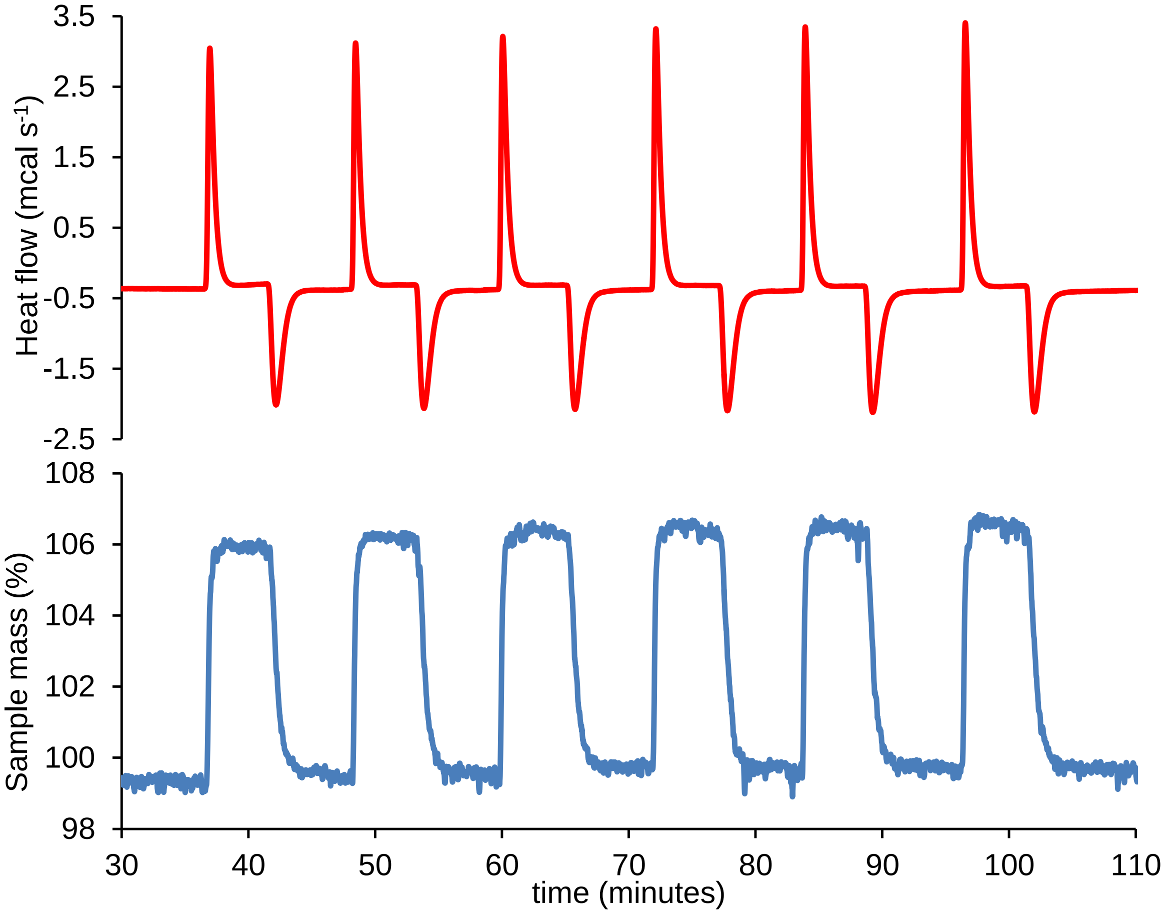

Thermogravimetric analysis of CO_2 adsorption onto $SN_{Ure}800_{Mo}$

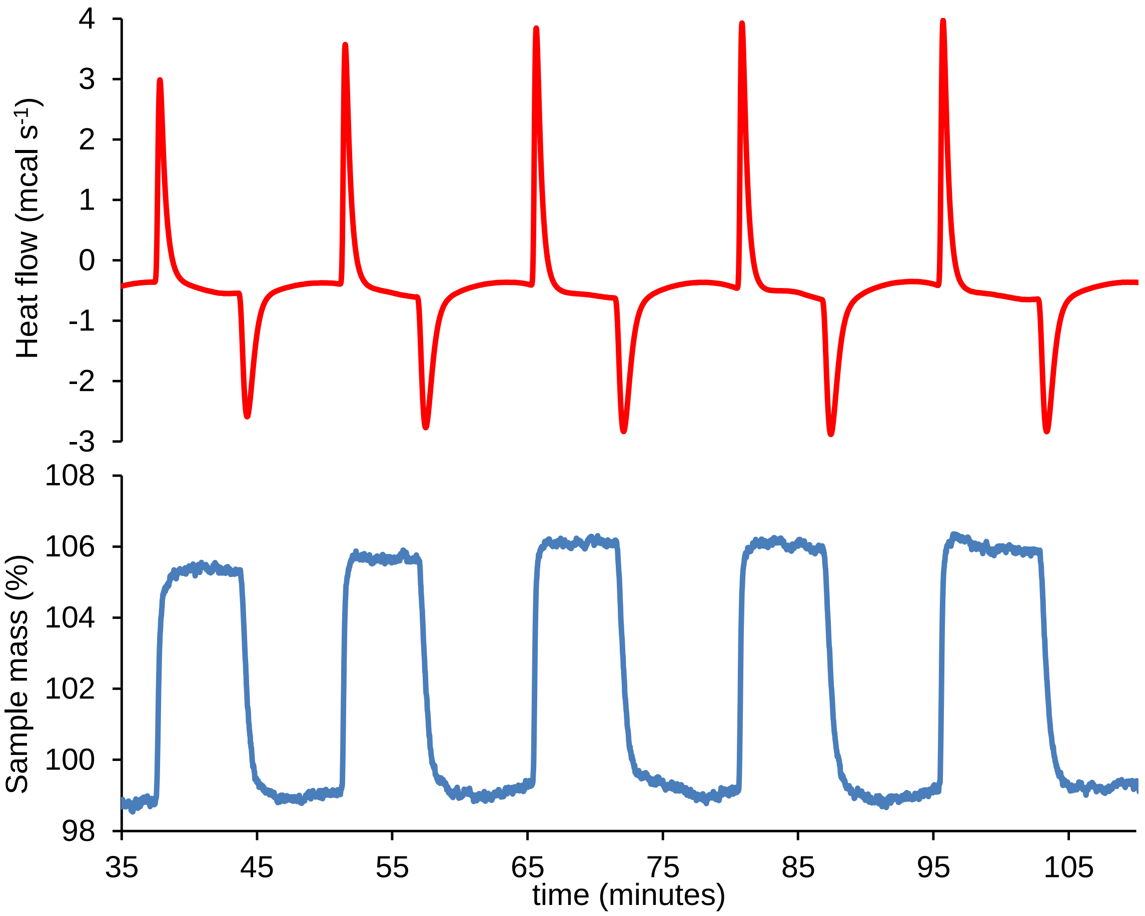

Thermogravimetric analysis of CO_2 adsorption onto $SN_{Ure}800_{Mu}$

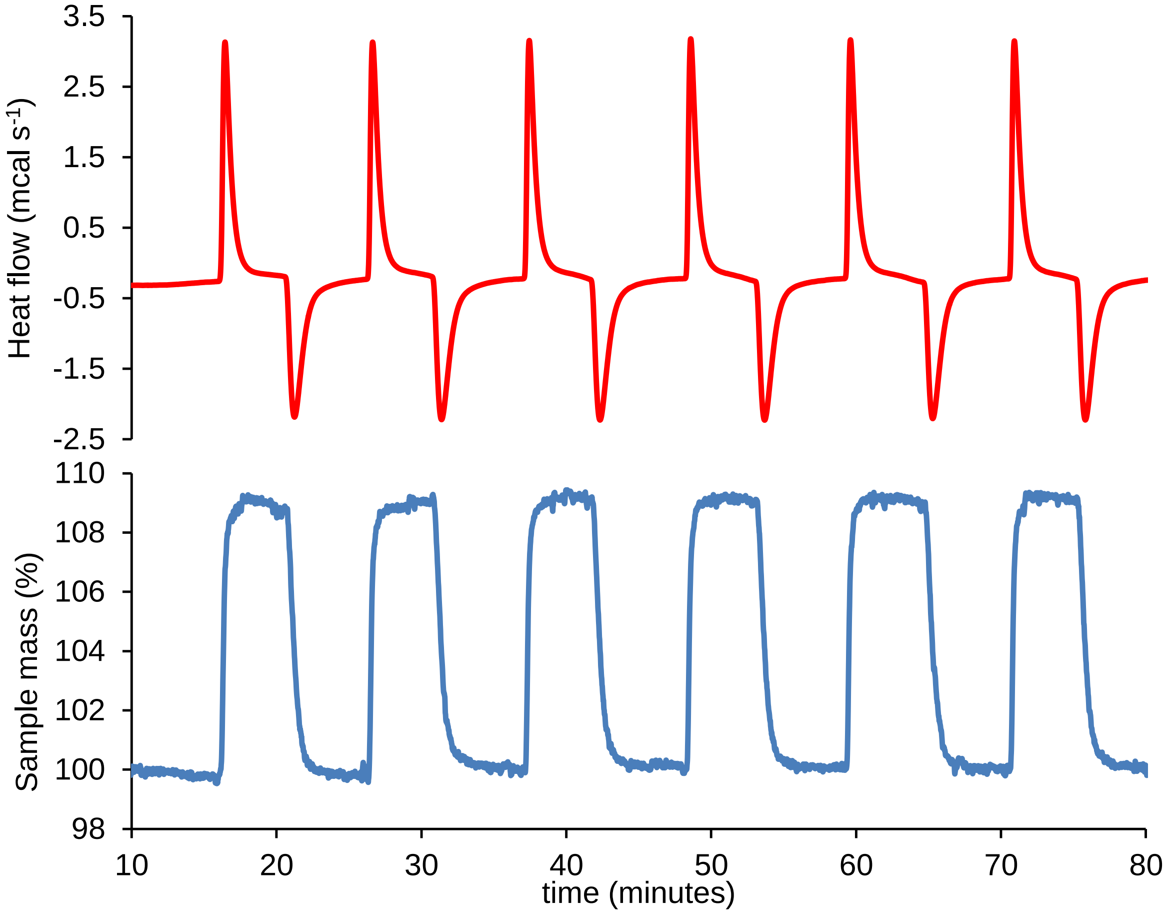

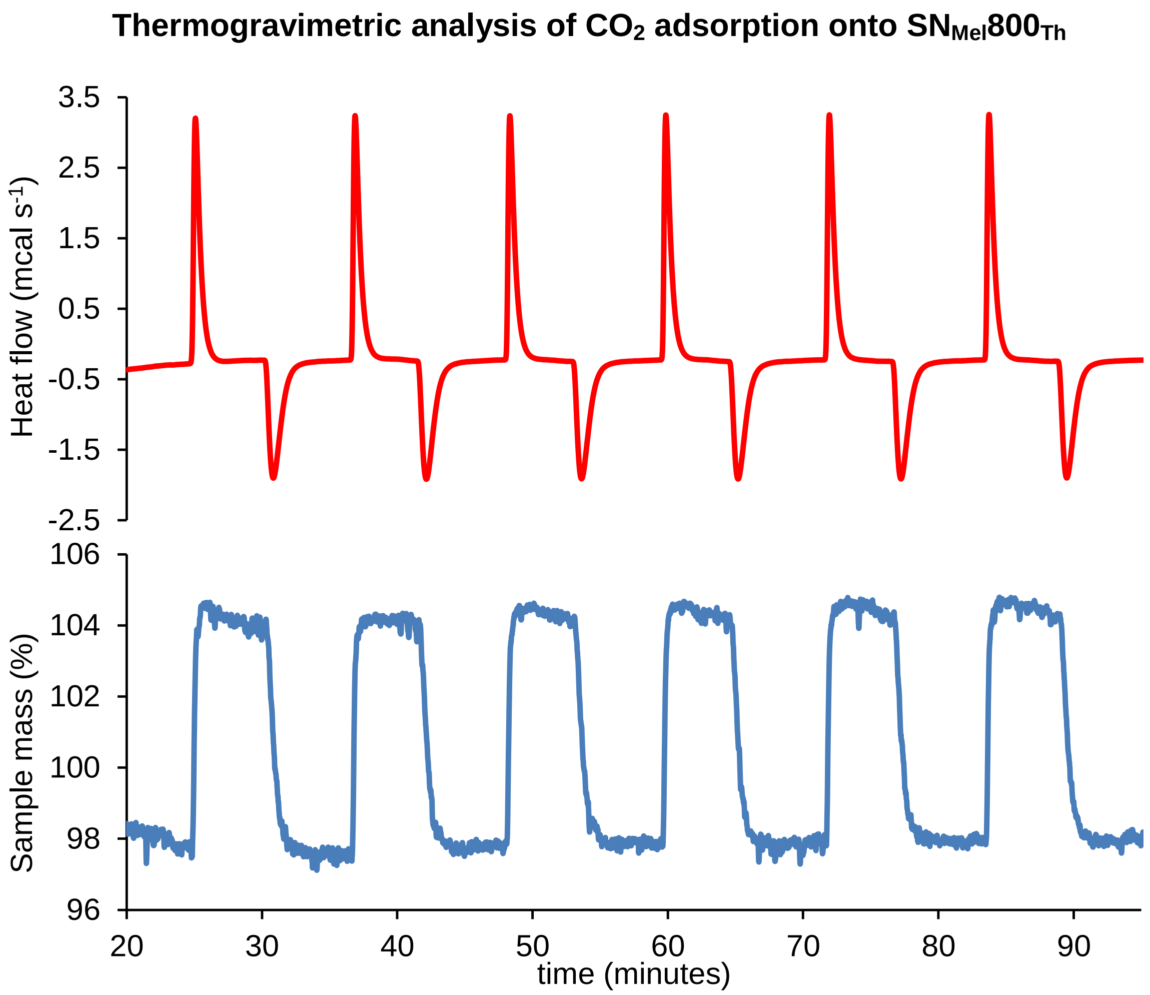
Thermogravimetric analysis of CO_2 adsorption onto $SN_{Mel}800_{Th}$
Heat flow (mcal s^{-1})
3.5
2.5
1.5
0.5
-0.5
-1.5
-2.5
Sample mass (%)
106
104
102
100
98
96
20
30
40
50
60
70
80
90
time (minutes)

Thermogravimetric analysis of CO_2 adsorption onto $SN_{Mel}800_{Mo}$

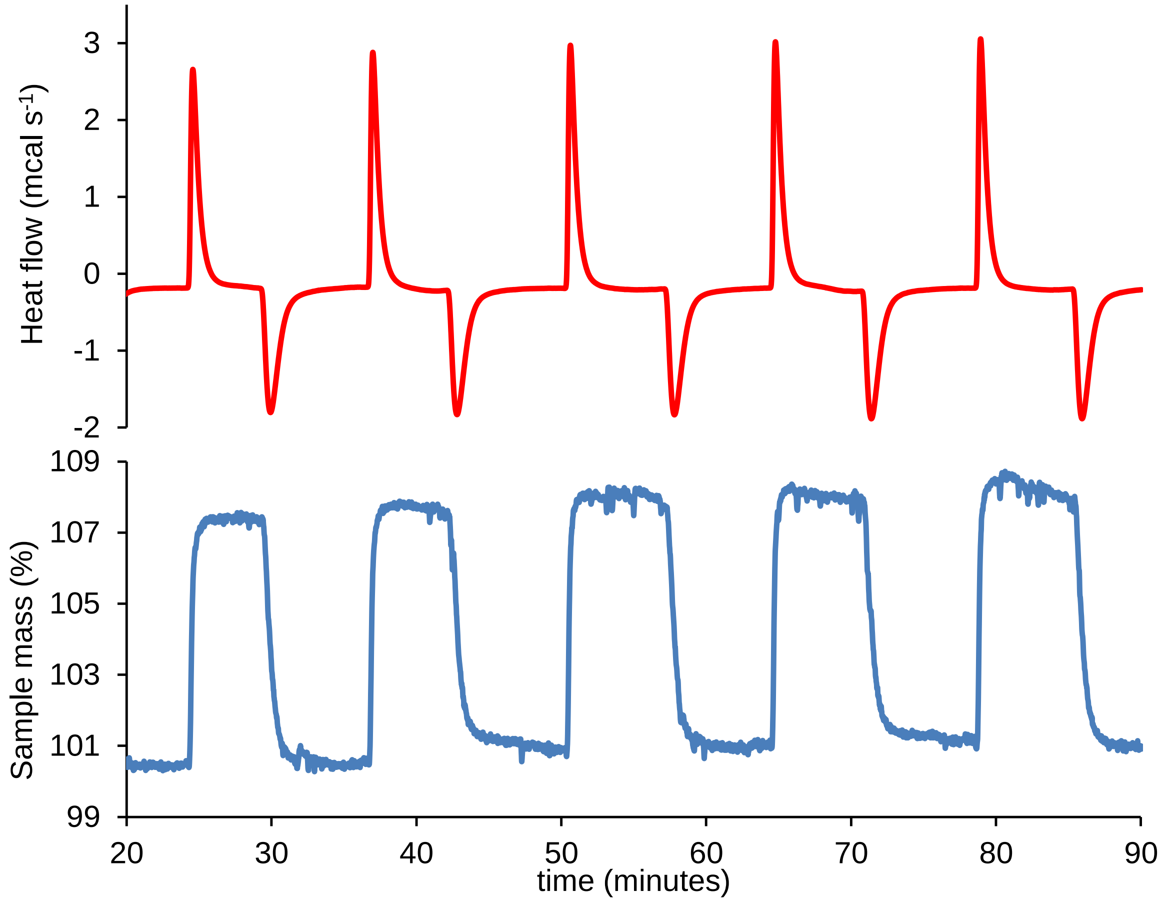

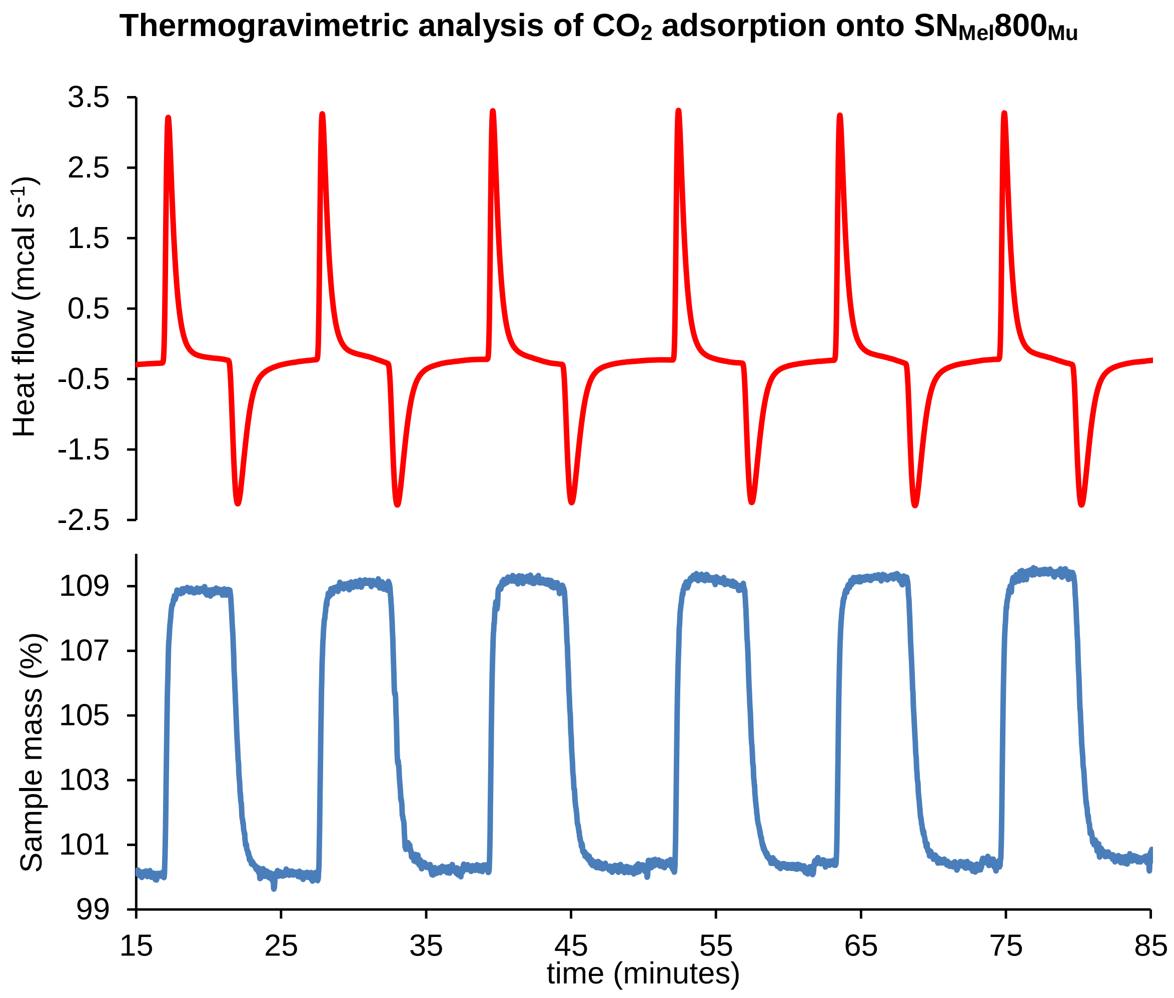
Thermogravimetric analysis of CO2 adsorption onto SNMel800Mu
Heat flow (mcal s-1)
3.5
2.5
1.5
0.5
-0.5
-1.5
-2.5
Sample mass (%)
109
107
105
103
101
99
15
25
35
45
55
65
75
85
time (minutes)

Thermogravimetric analysis of CO_2 adsorption onto $SN_{Nic}800_{Th}$

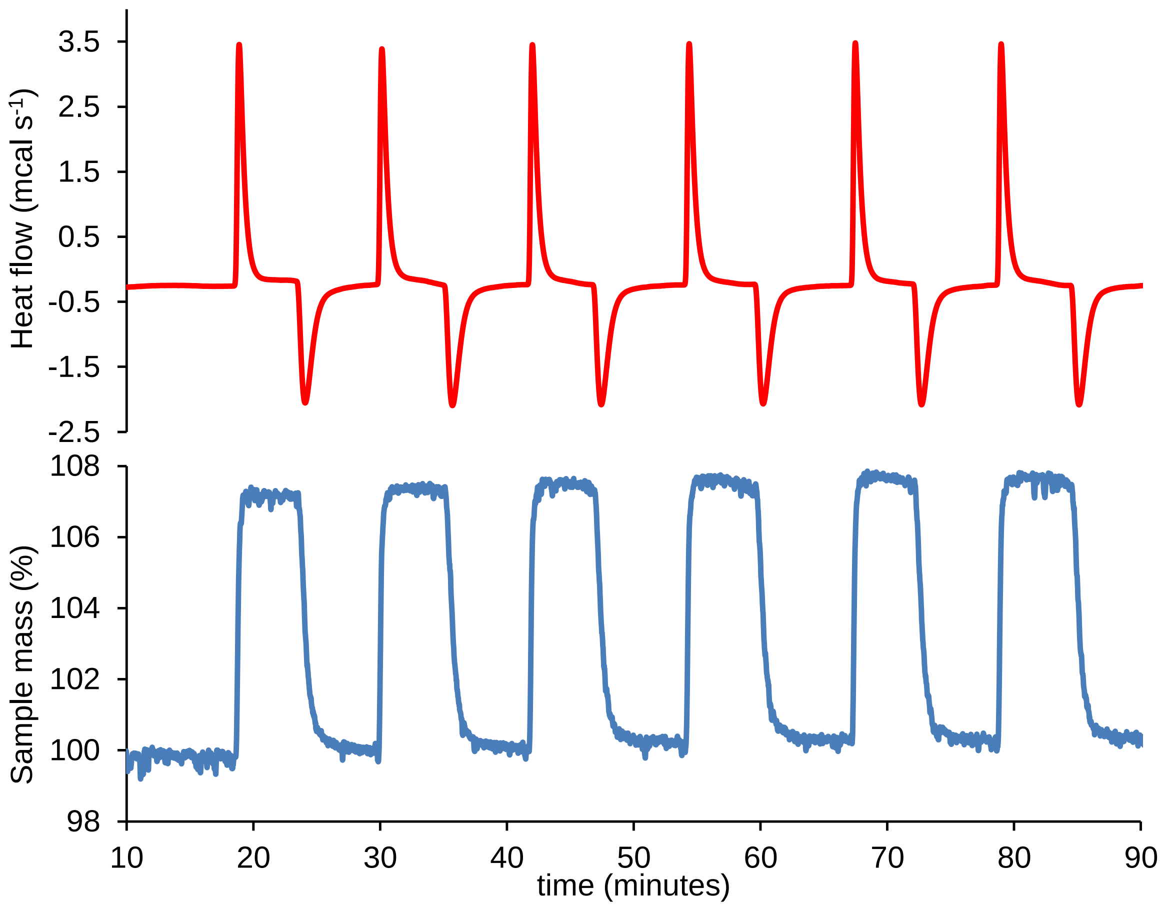

Thermogravimetric analysis of CO_2 adsorption onto $SN_{Nic}800_{Mo}$

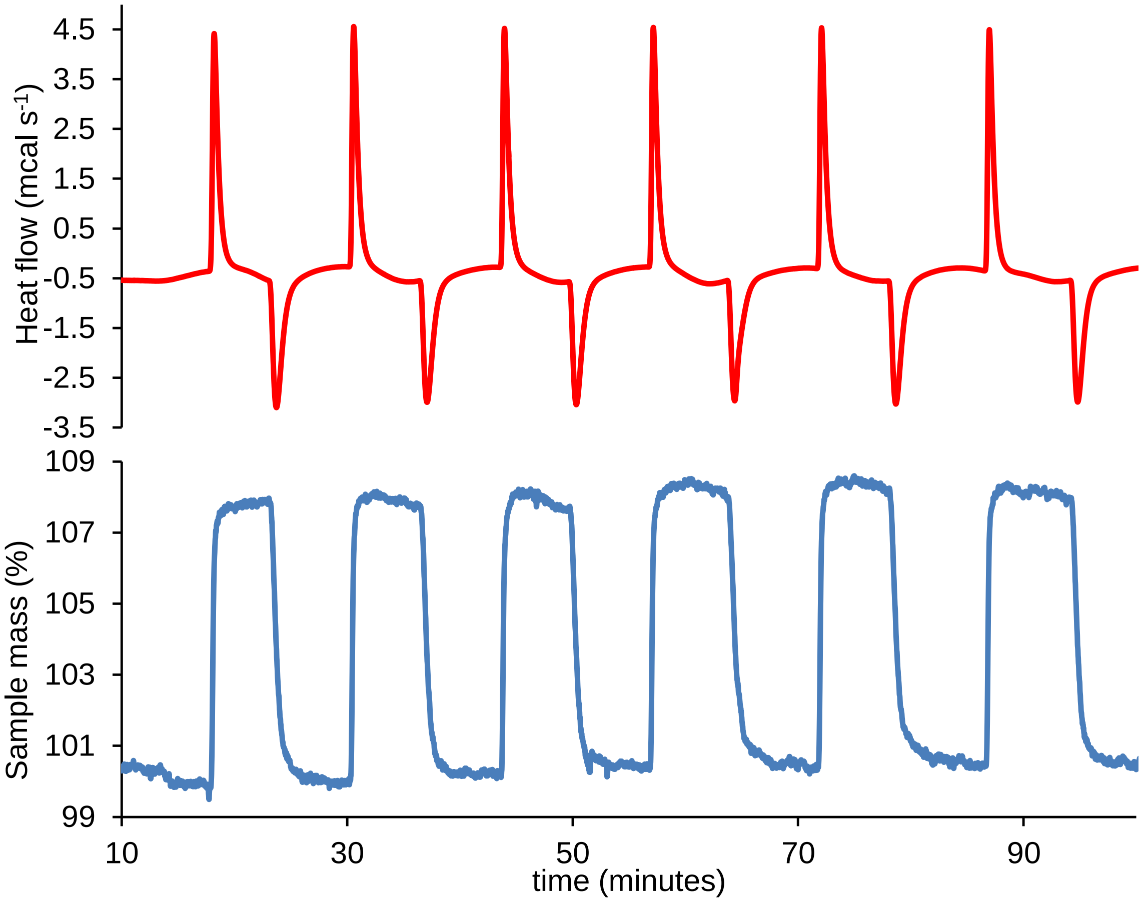

Thermogravimetric analysis of CO_2 adsorption onto $SN_{Nic}800_{Mu}$

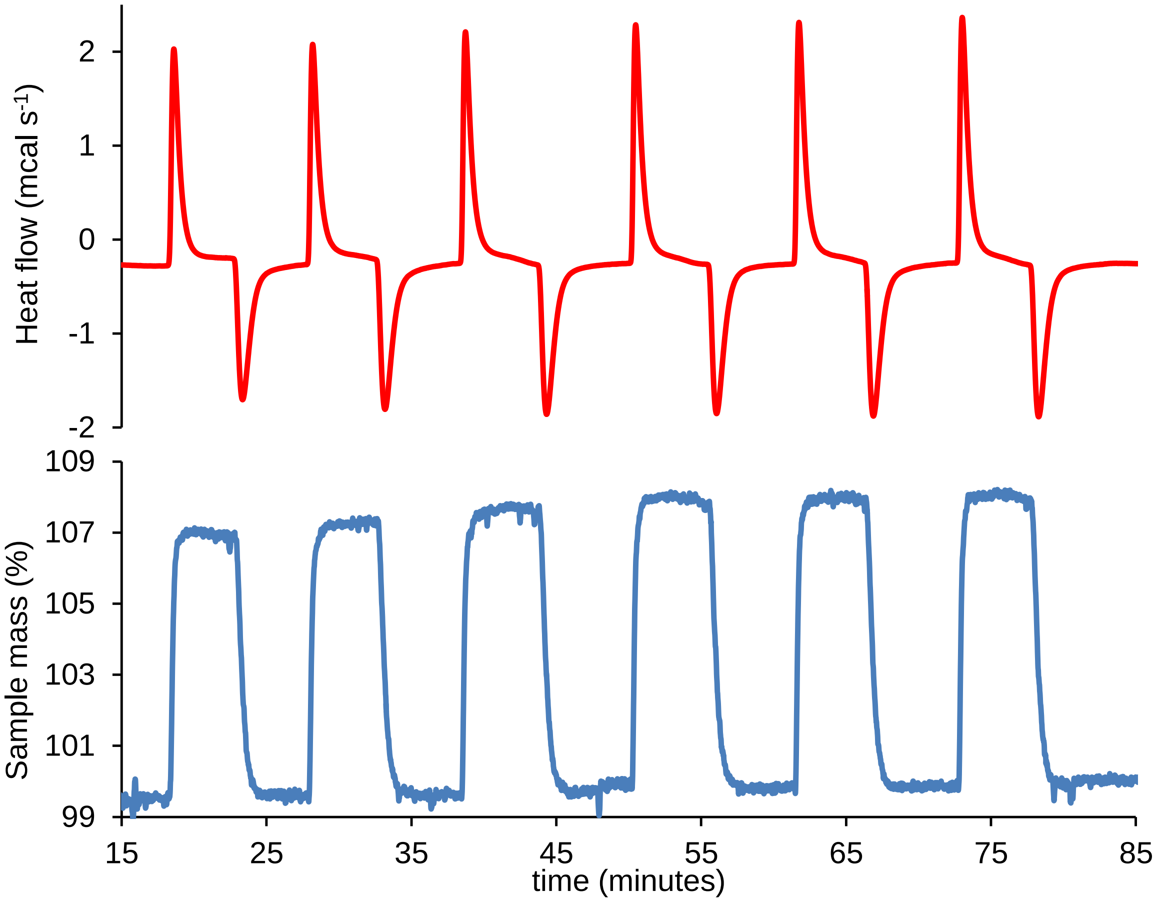

CO_2 adsorption isotherm onto S800

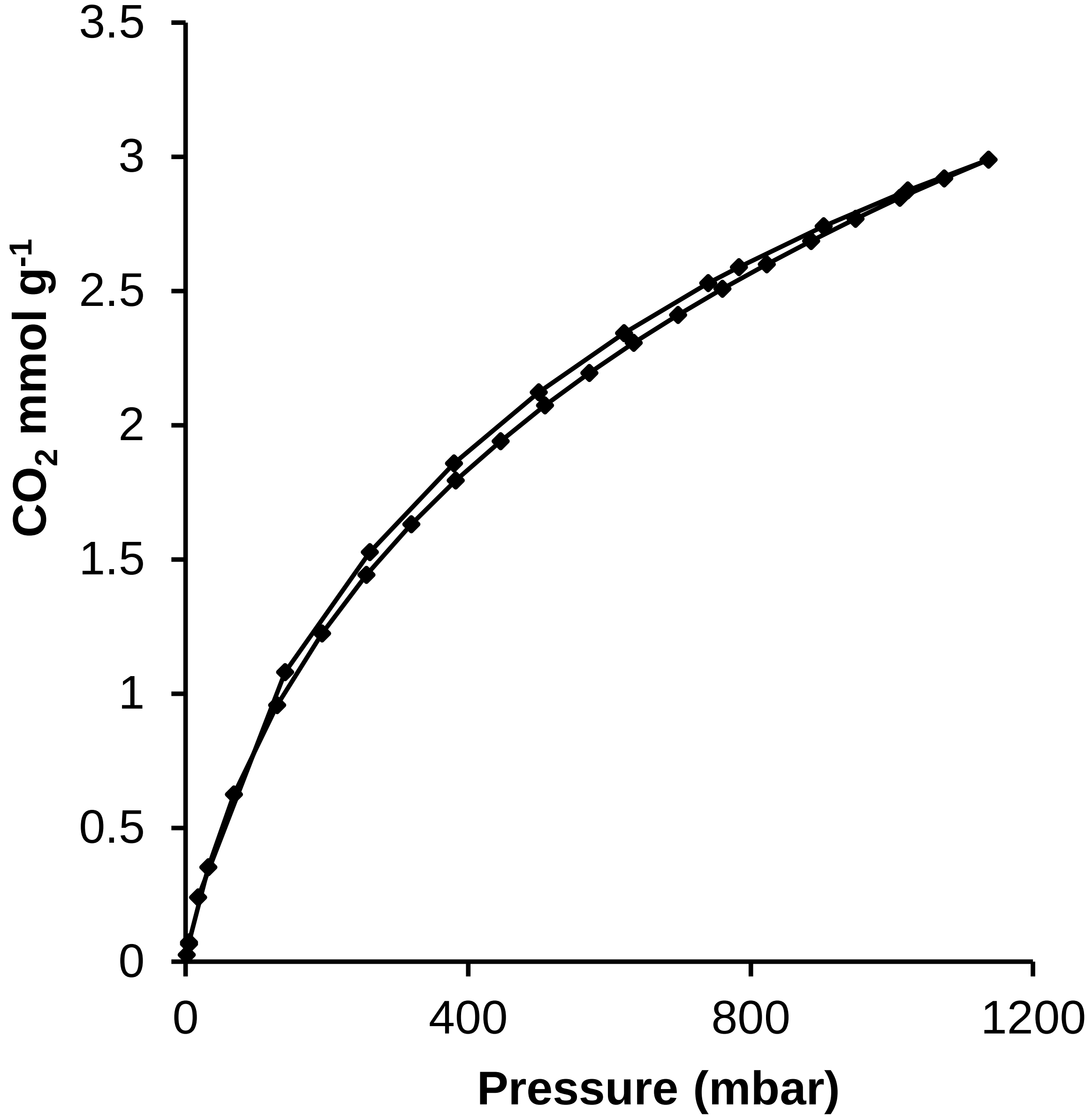

CO_2 adsorption isotherm onto $SN_{Gly}800_{Th}$

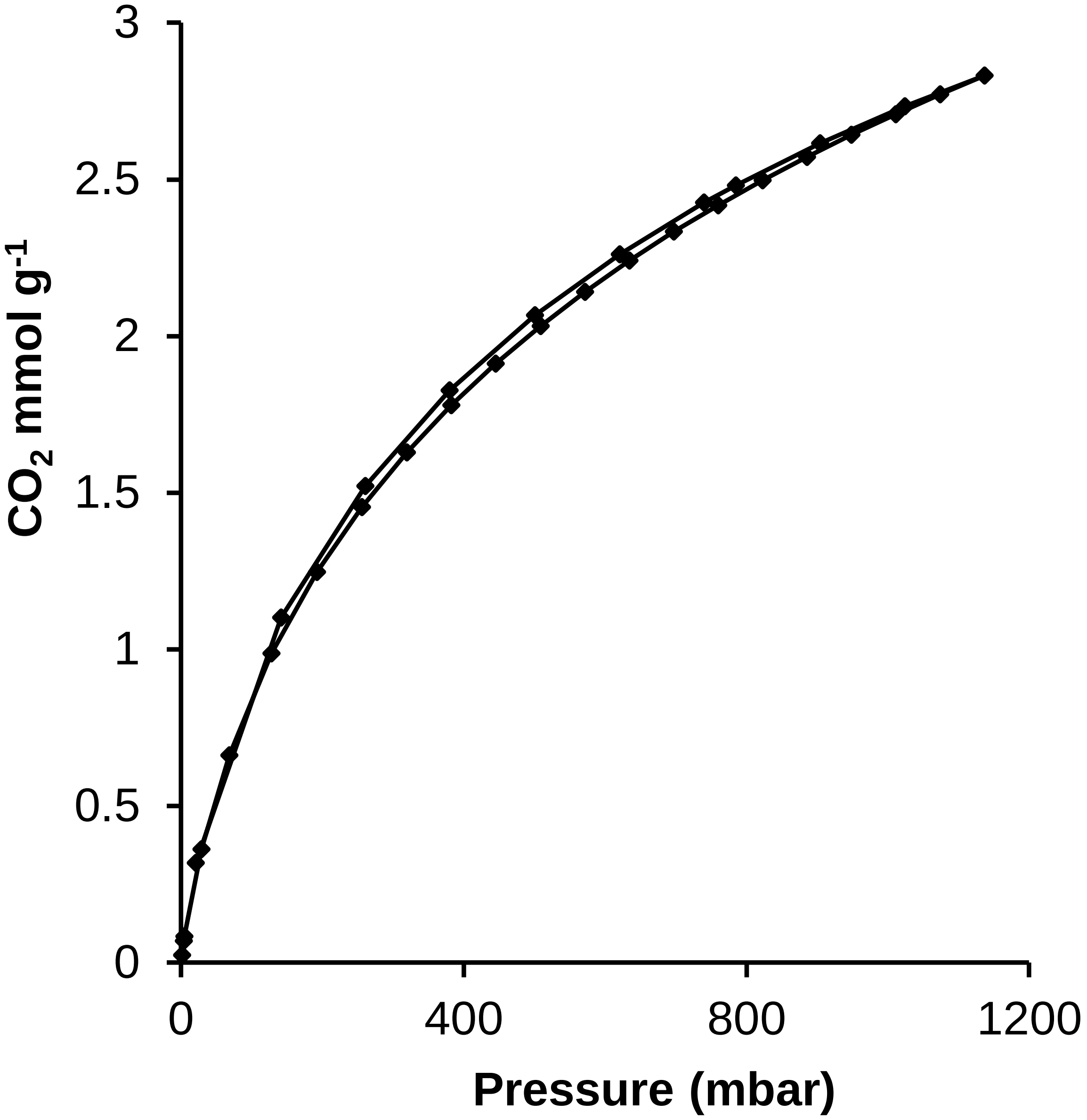

CO_2 adsorption isotherm onto $SN_{Gly}800_{Mo}$

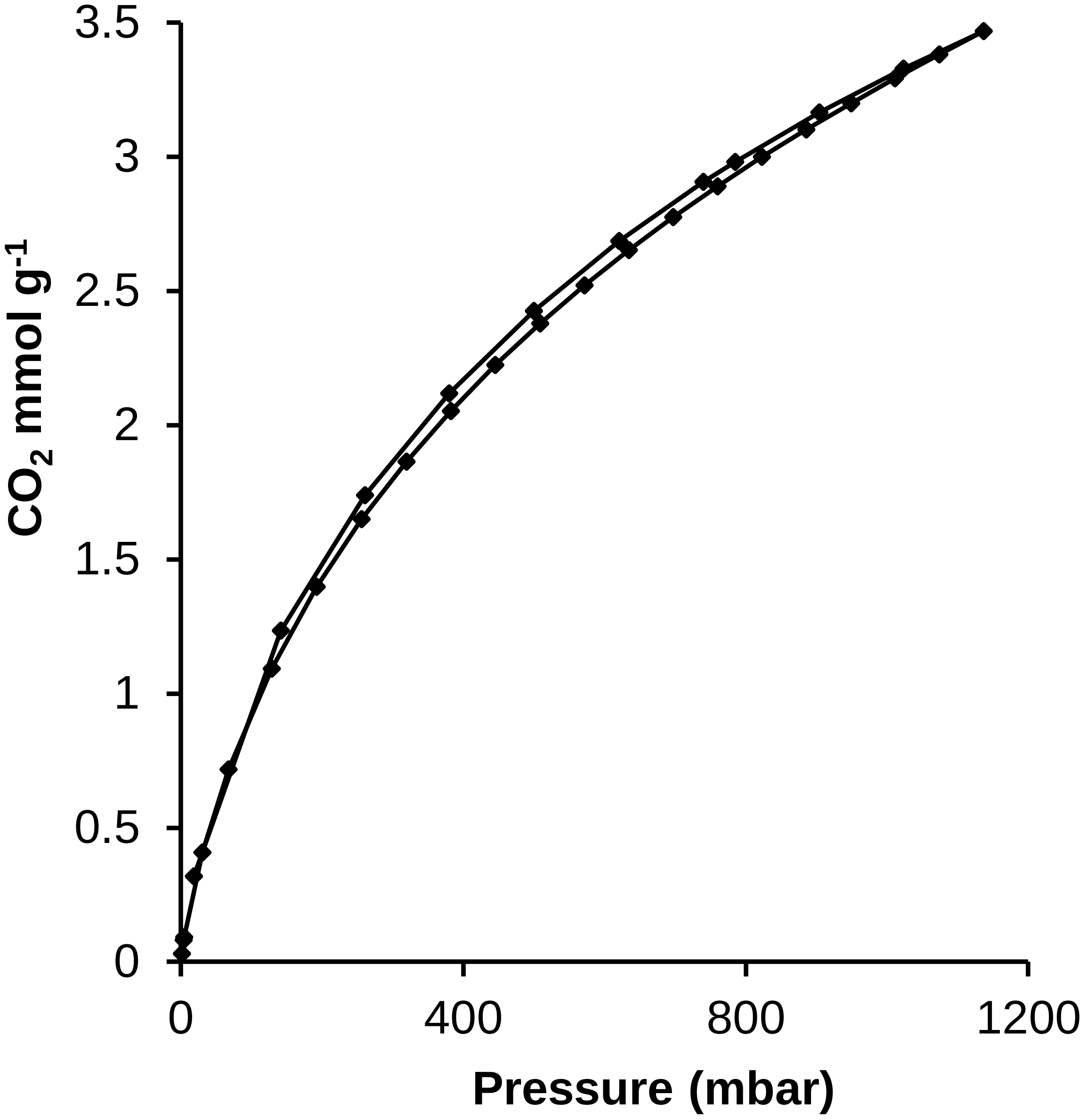

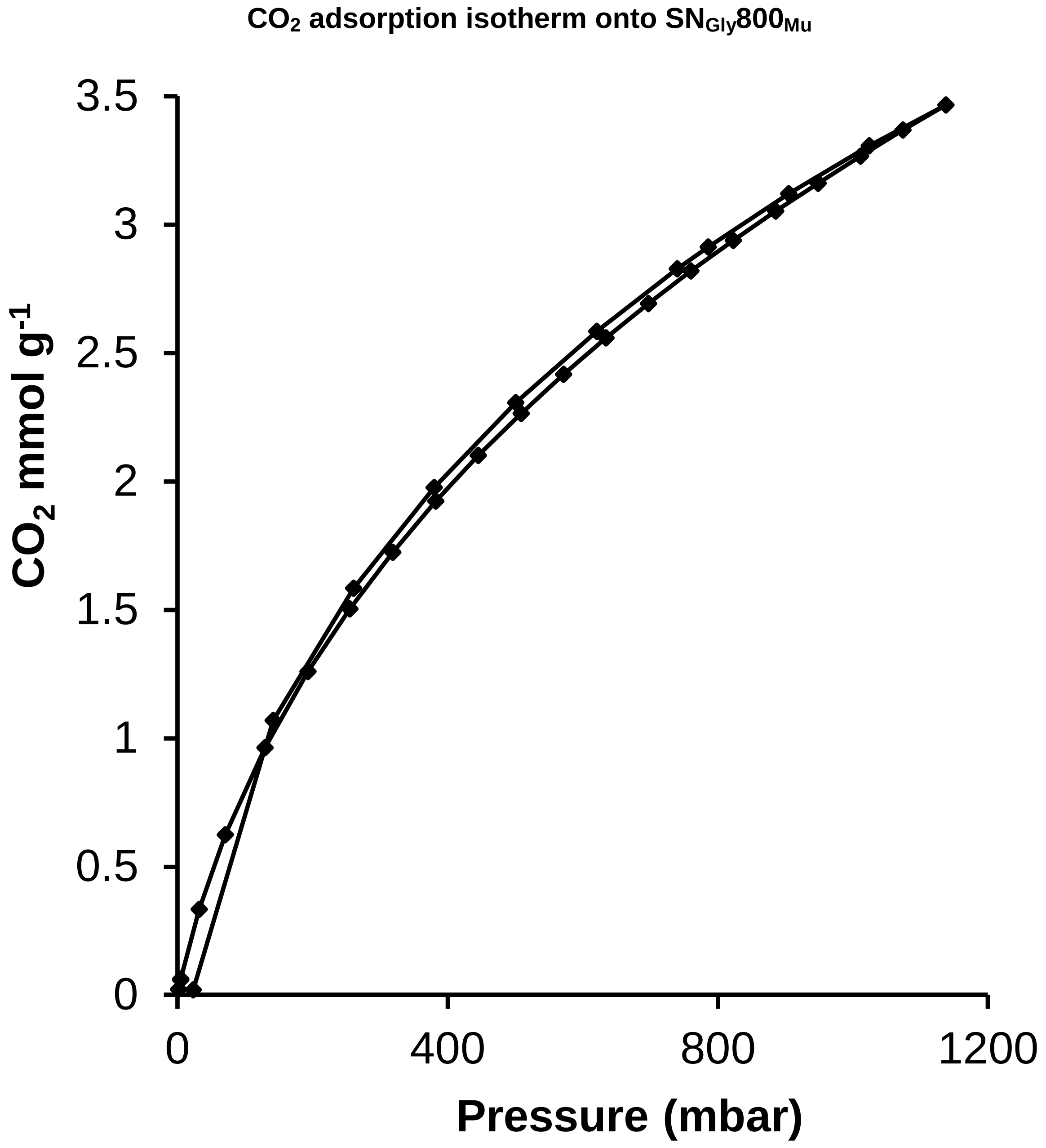
CO2 adsorption isotherm onto SNGly800Mu
CO2 mmol g-1
3.5
3
2.5
2
1.5
1
0.5
0
0
400
800
1200
Pressure (mbar)

CO_2 adsorption isotherm onto $SN_{Bal}800_{Mo}$

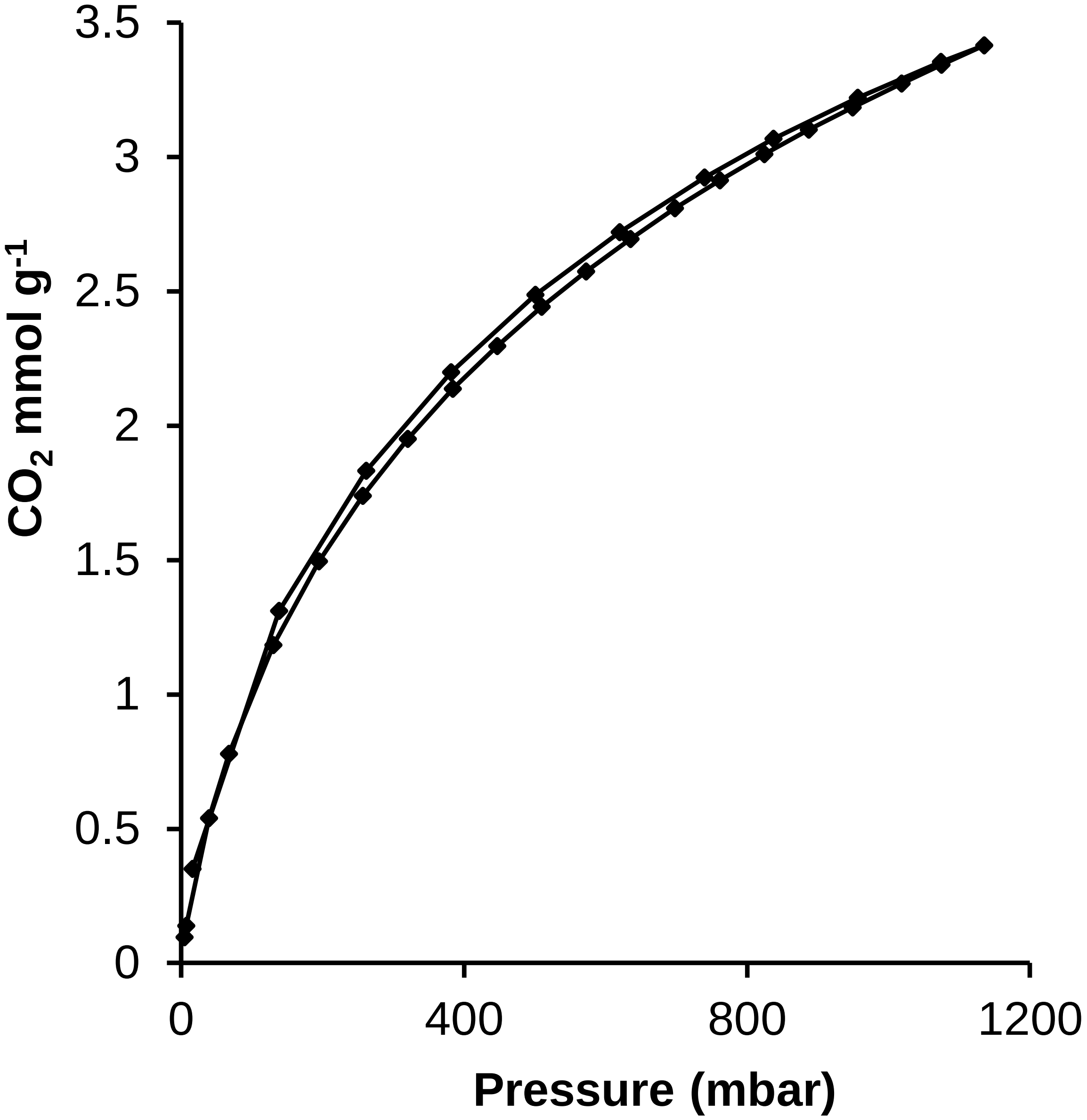

CO_2 adsorption isotherm onto $SN_{Ure}800_{Mo}$

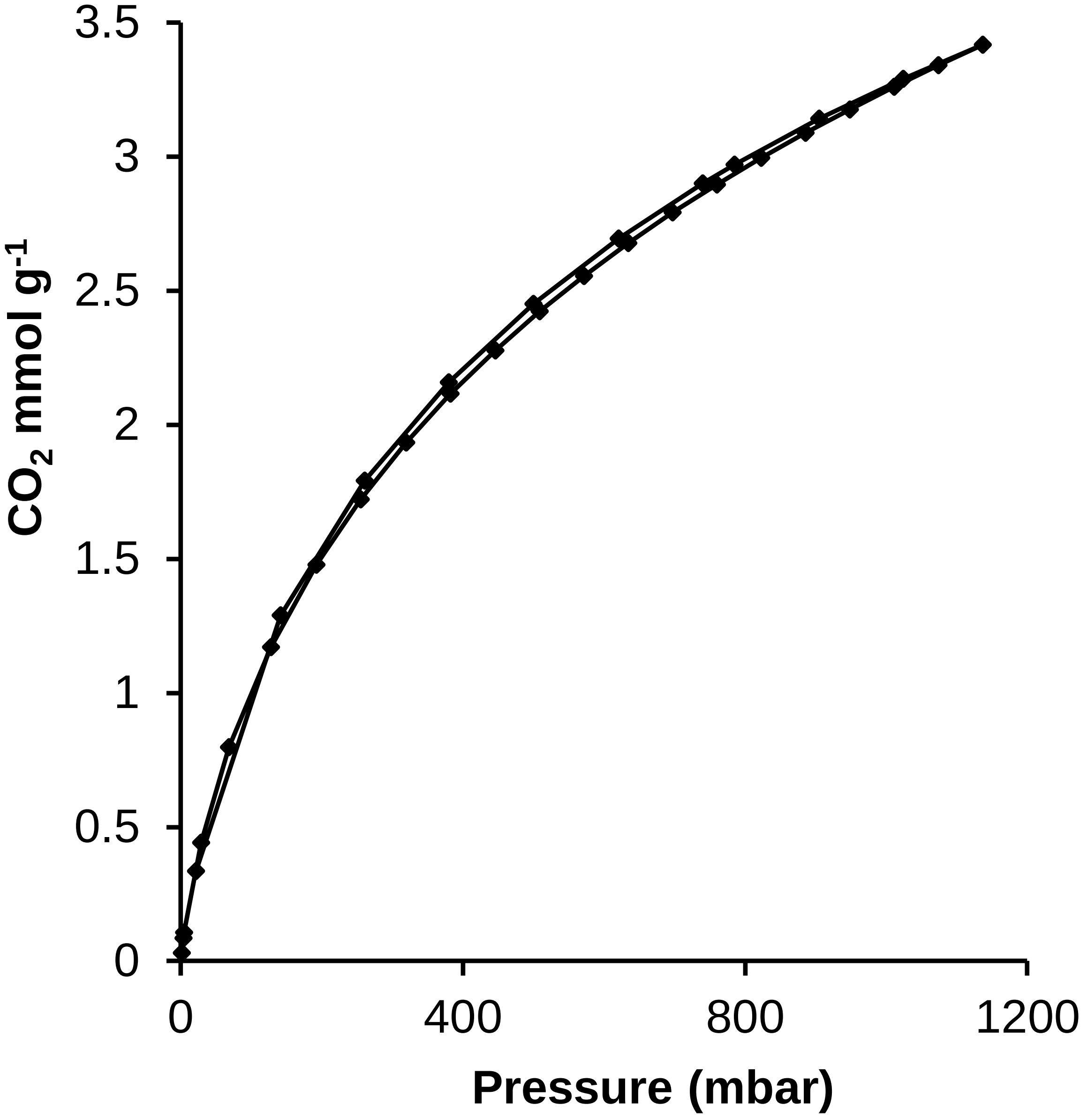

CO_2 adsorption isotherm onto $SN_{Mel}800_{Mo}$

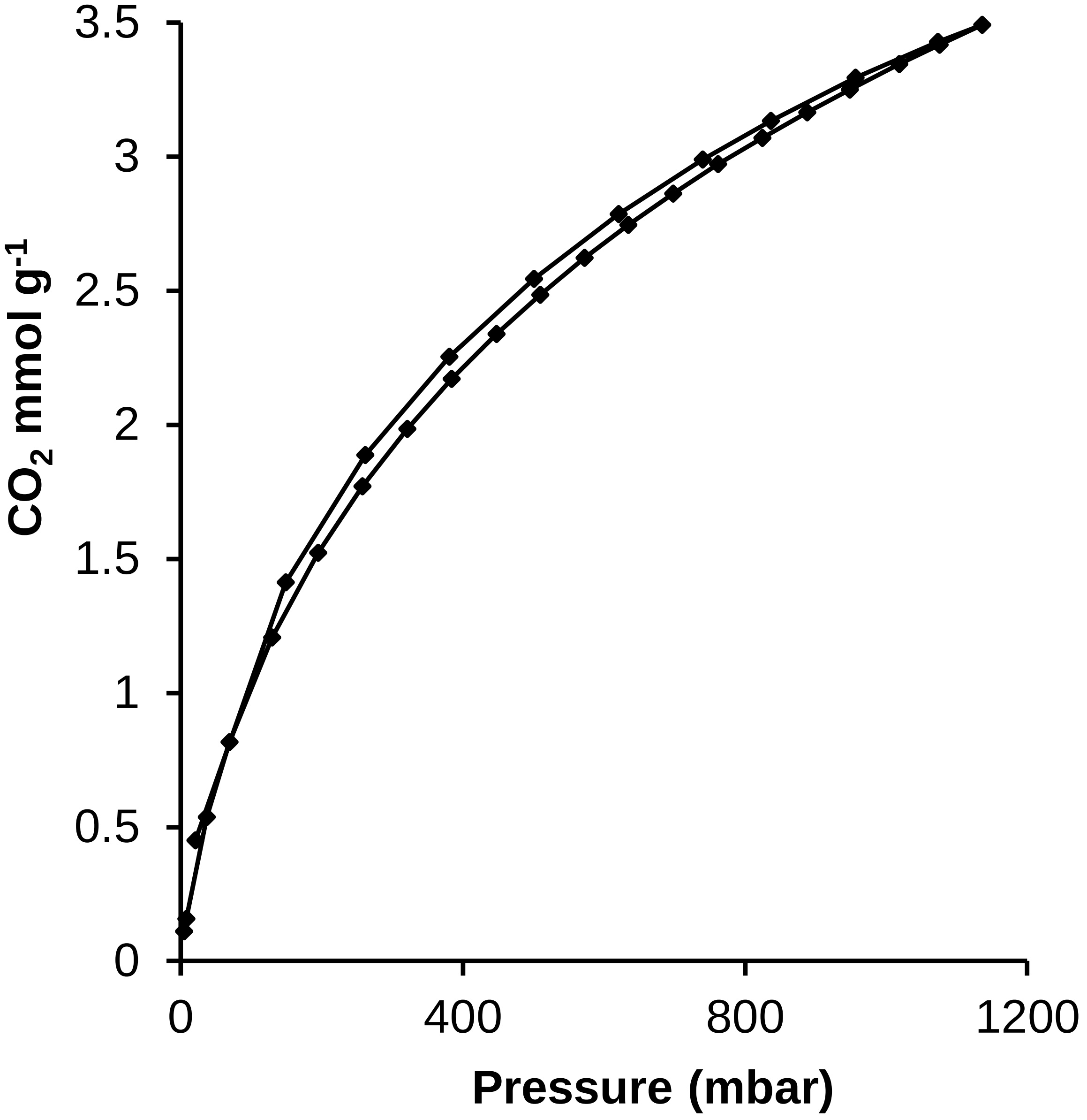

CO_2 adsorption isotherm onto $SN_{Nic}800_{Mo}$

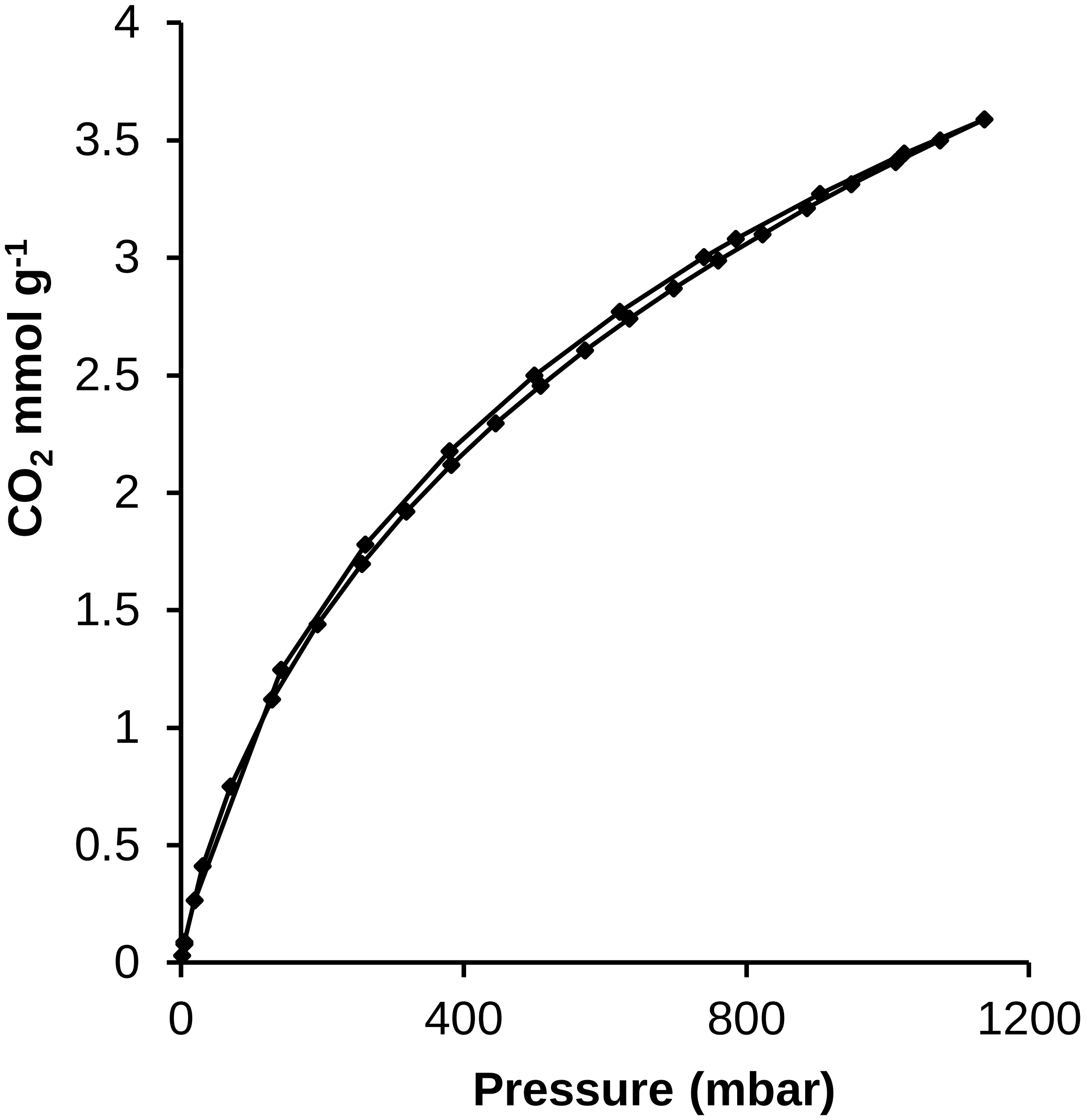

N_2 adsorption isotherm onto S800

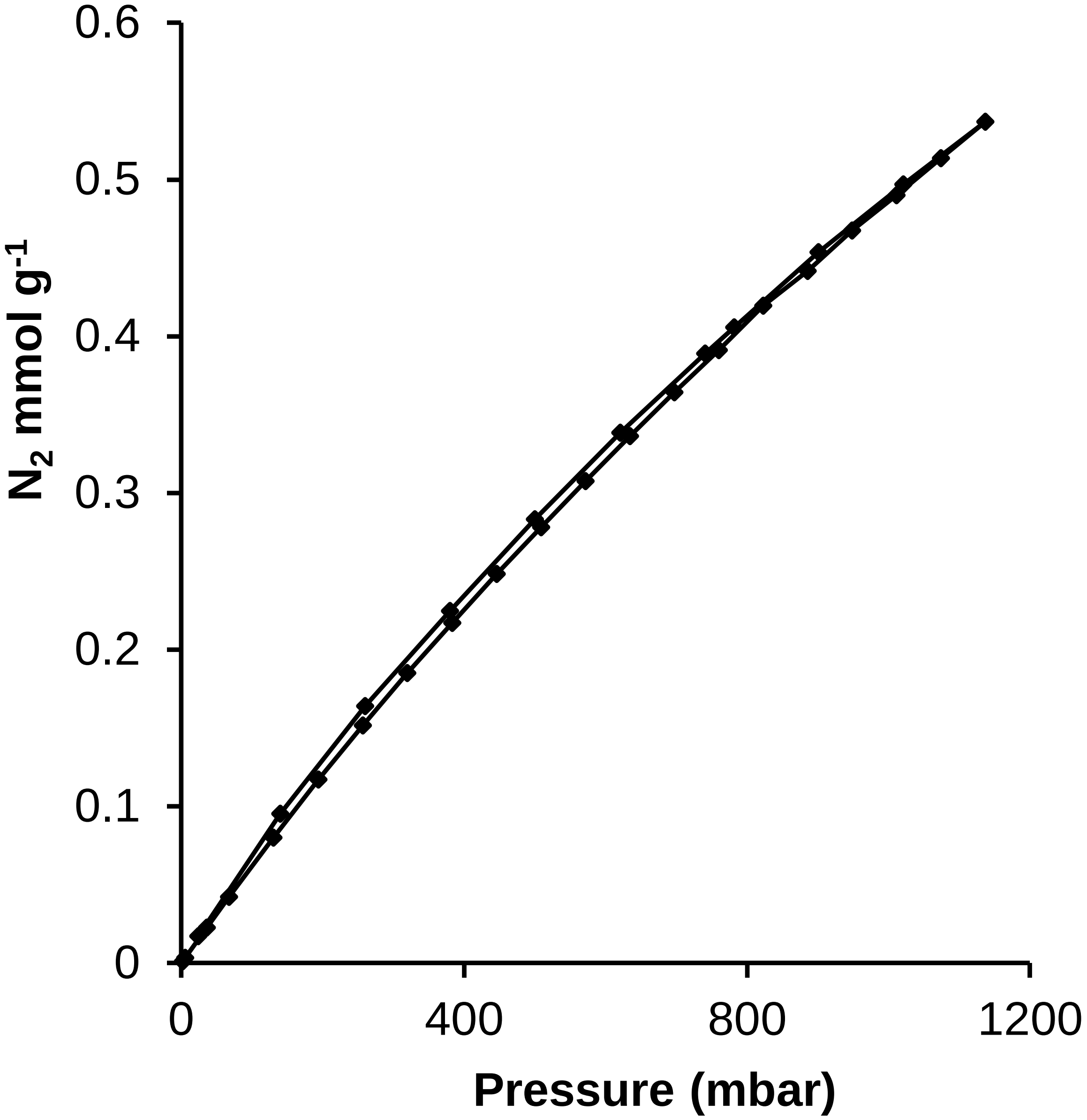

N_2 adsorption isotherm onto $SN_{Gly}800_{Th}$

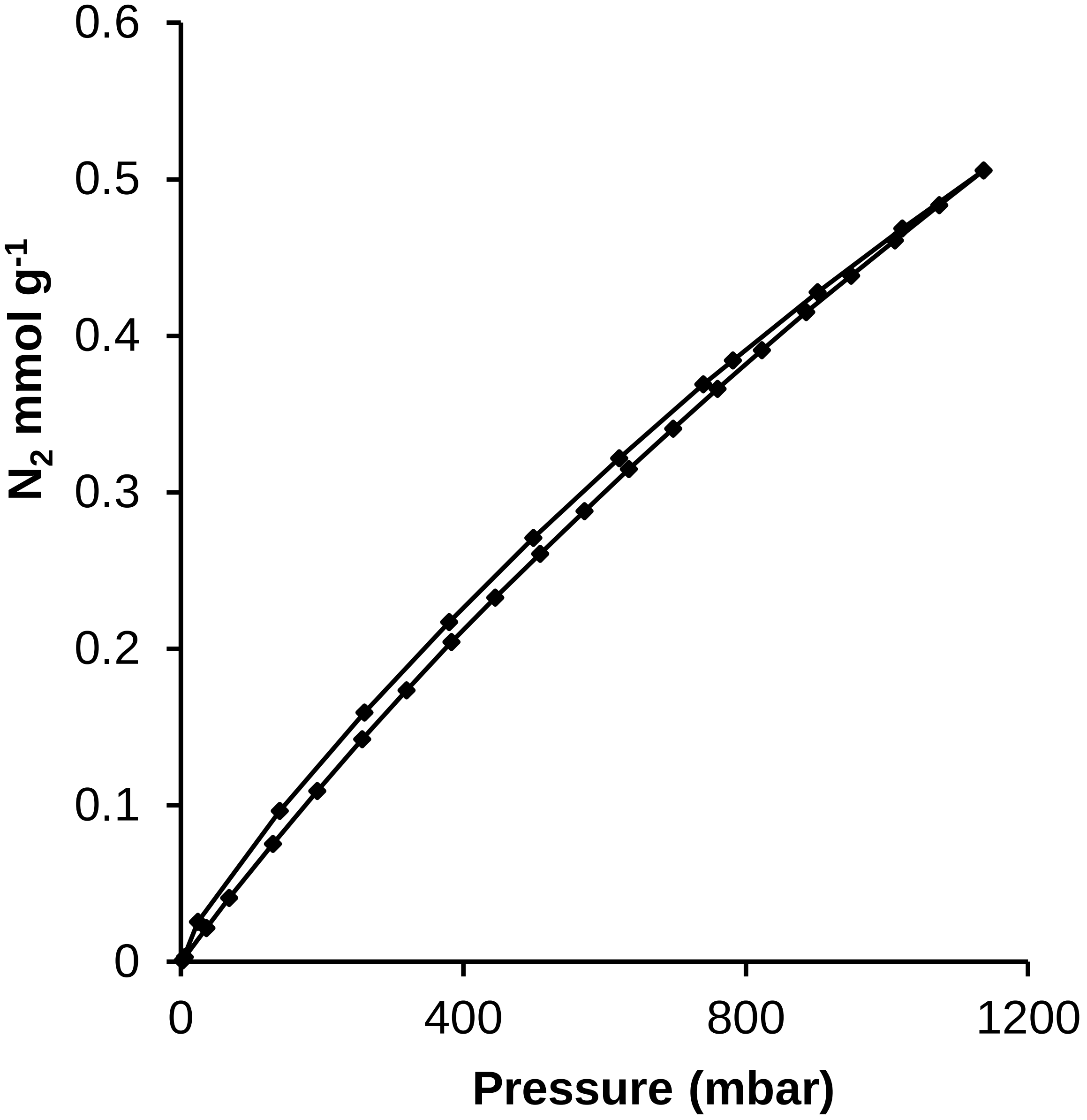

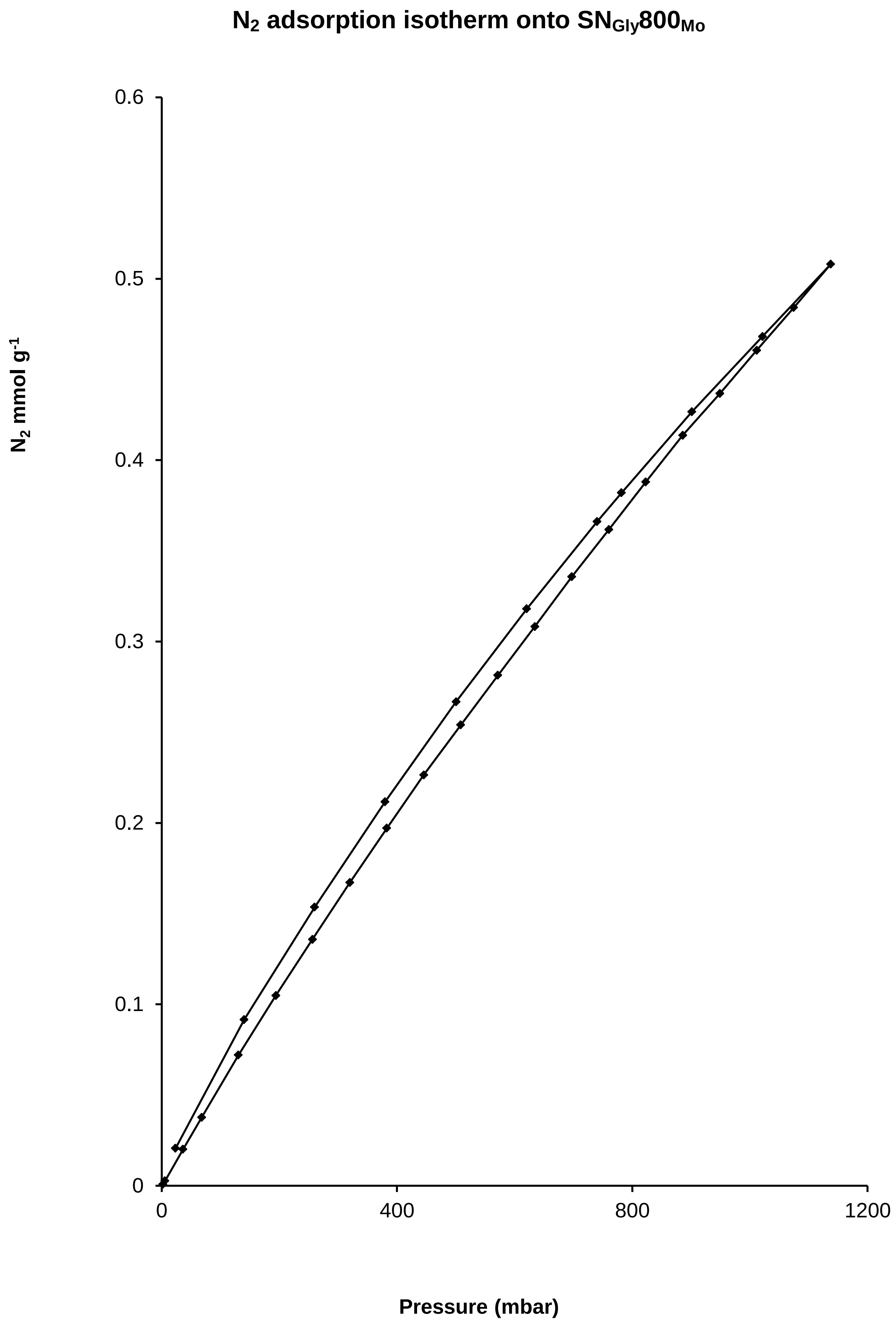
N2 adsorption isotherm onto SNGly800Mo
N2 mmol g-1
0.6
0.5
0.4
0.3
0.2
0.1
0
0
400
800
1200
Pressure (mbar)

N_2 adsorption isotherm onto $SN_{Gly}800_{Mu}$

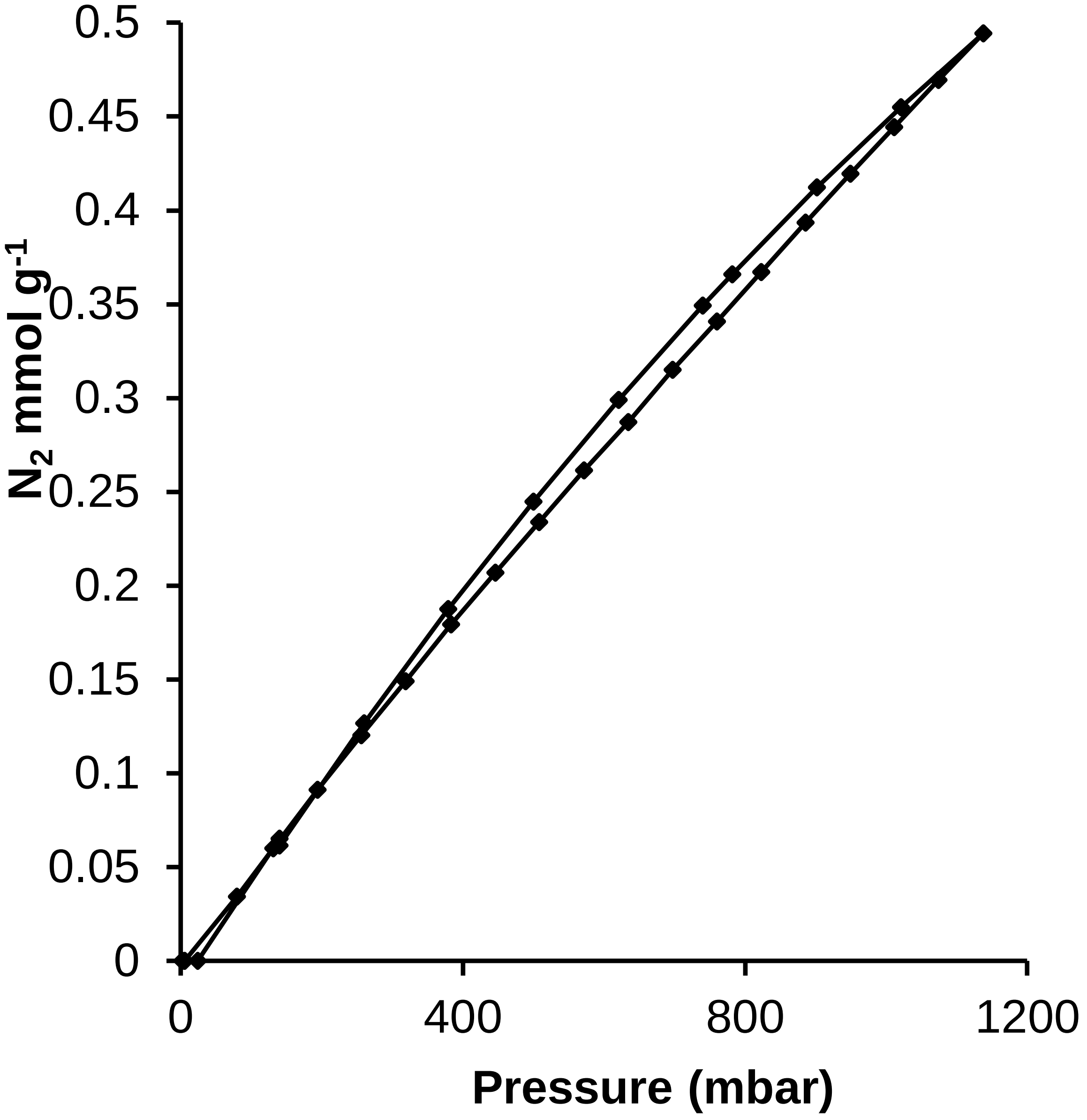

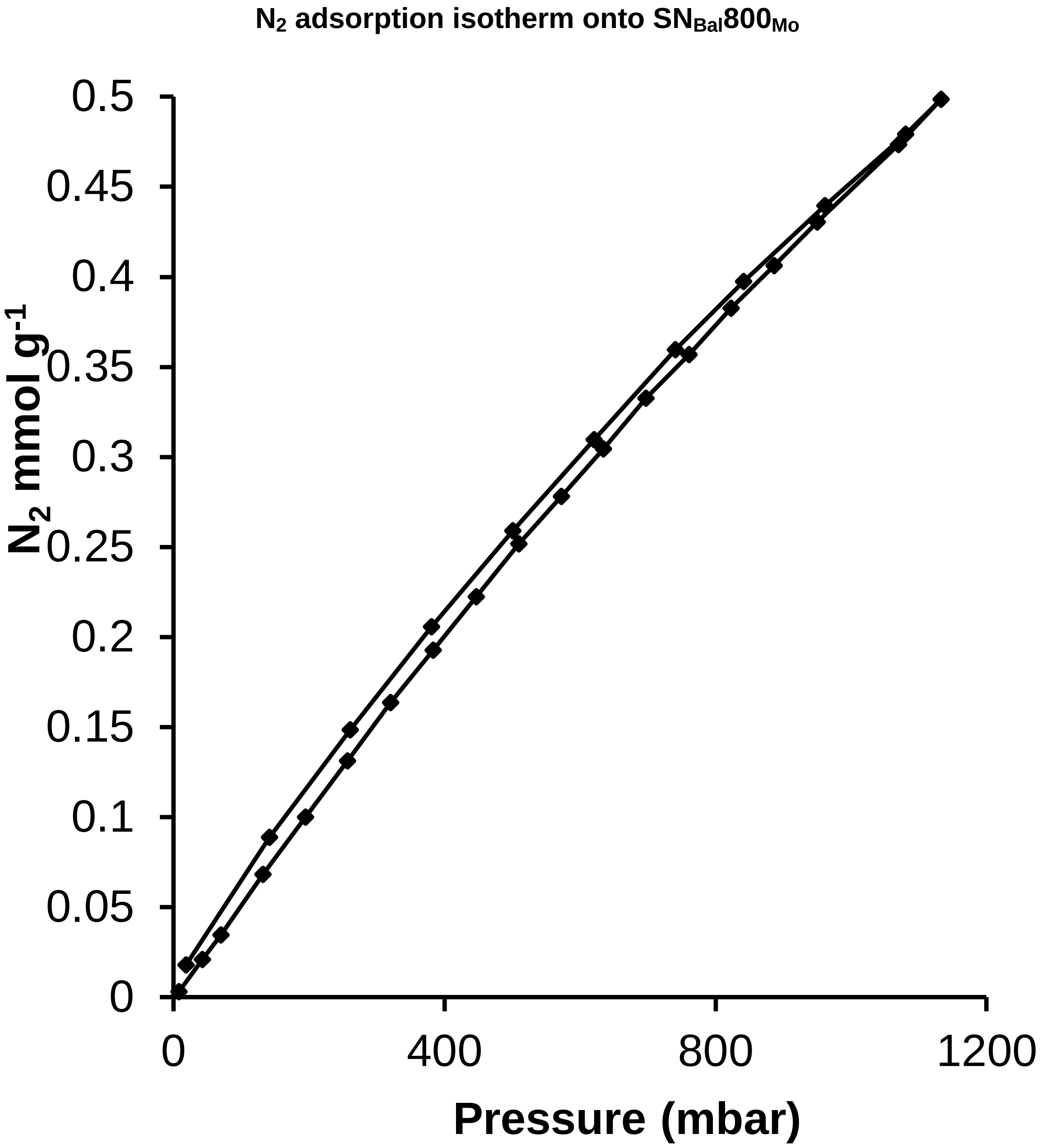
N2 adsorption isotherm onto SNBal800Mo
N2 mmol g-1
0.5
0.45
0.4
0.35
0.3
0.25
0.2
0.15
0.1
0.05
0
0
400
800
1200
Pressure (mbar)

N_2 adsorption isotherm onto $SN_{Ure}800_{Mo}$

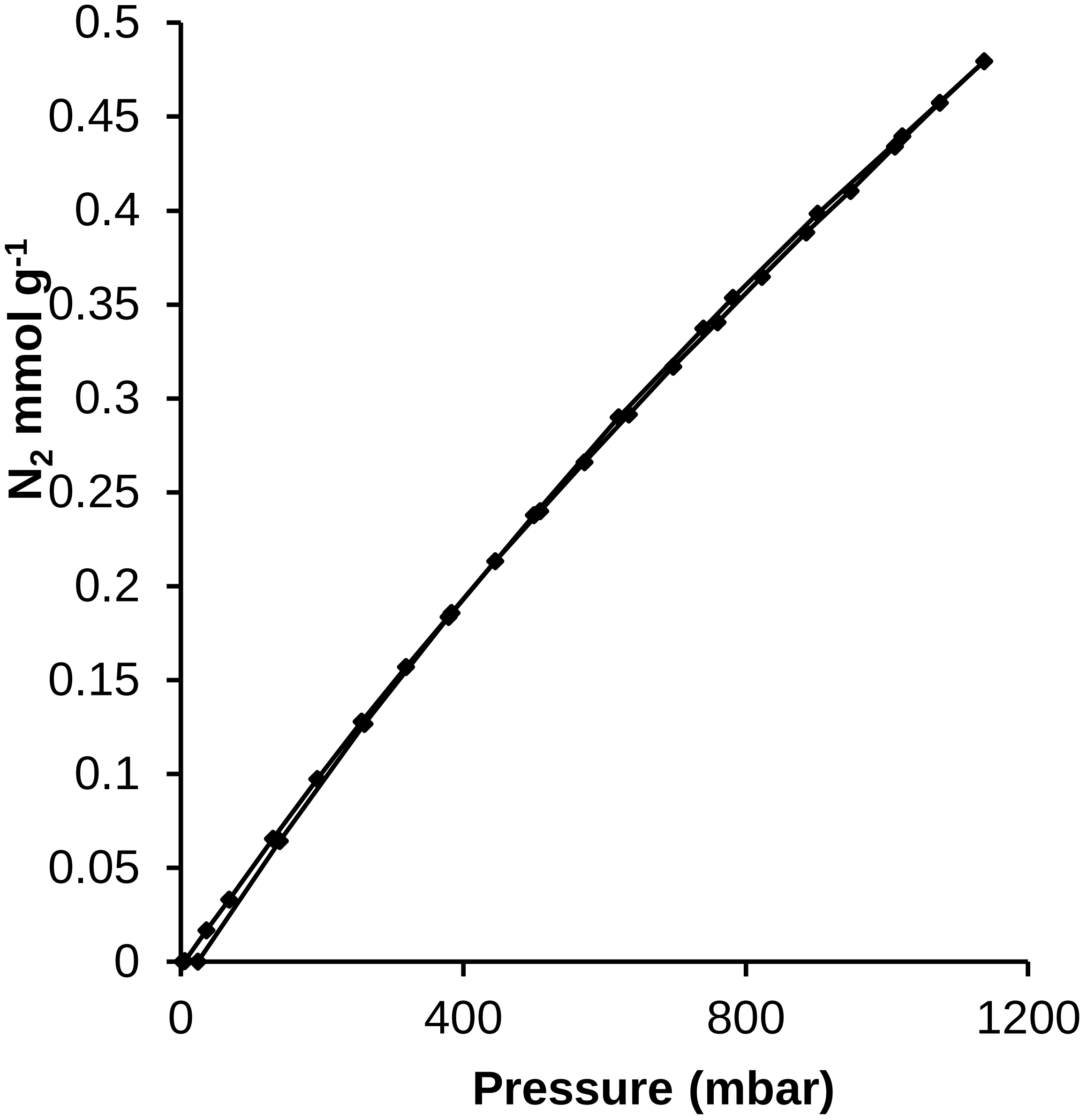

N_2 adsorption isotherm onto $SN_{Mel}800_{Mo}$

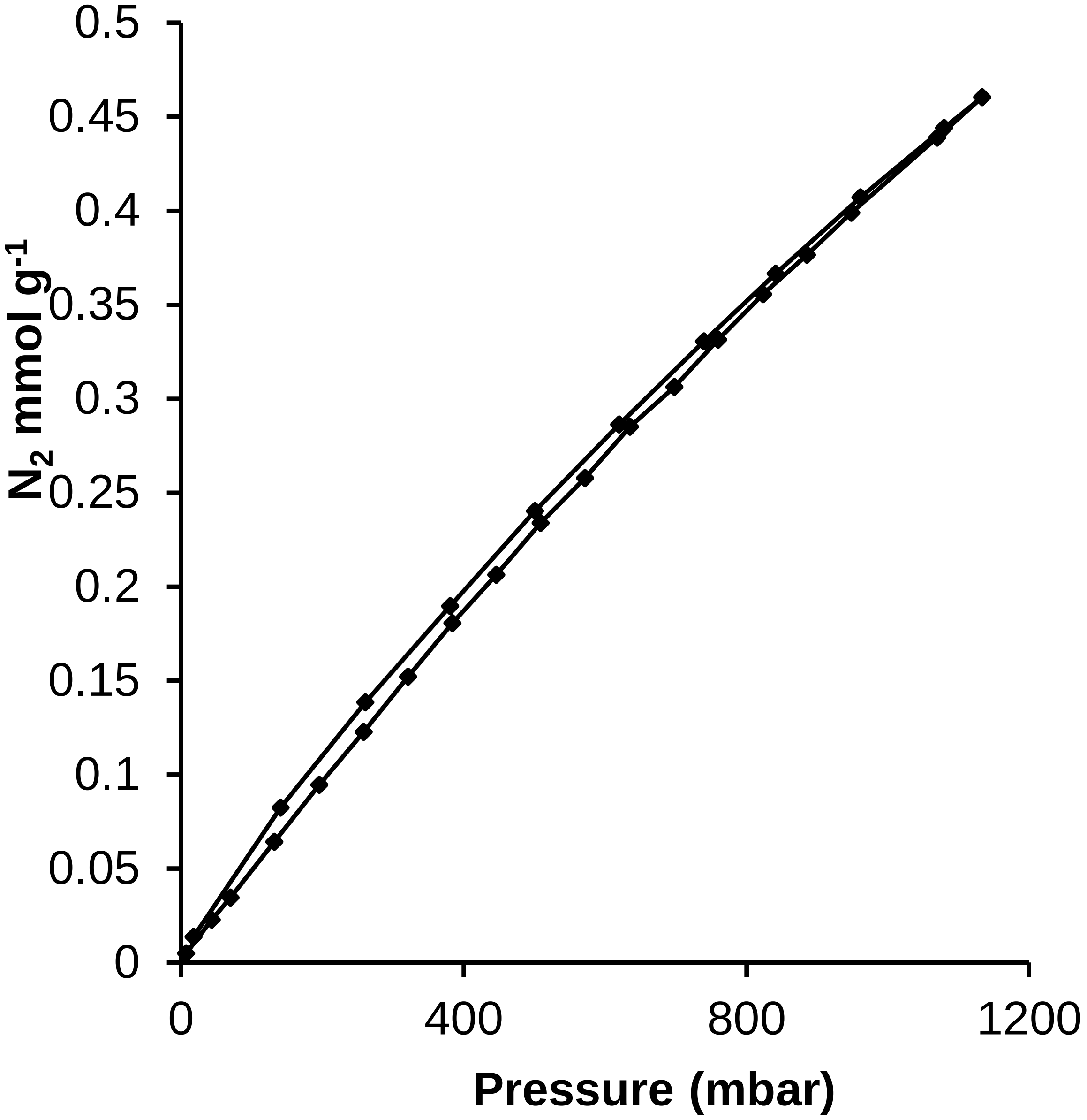

N_2 adsorption isotherm onto $SN_{Nic}800_{Mo}$

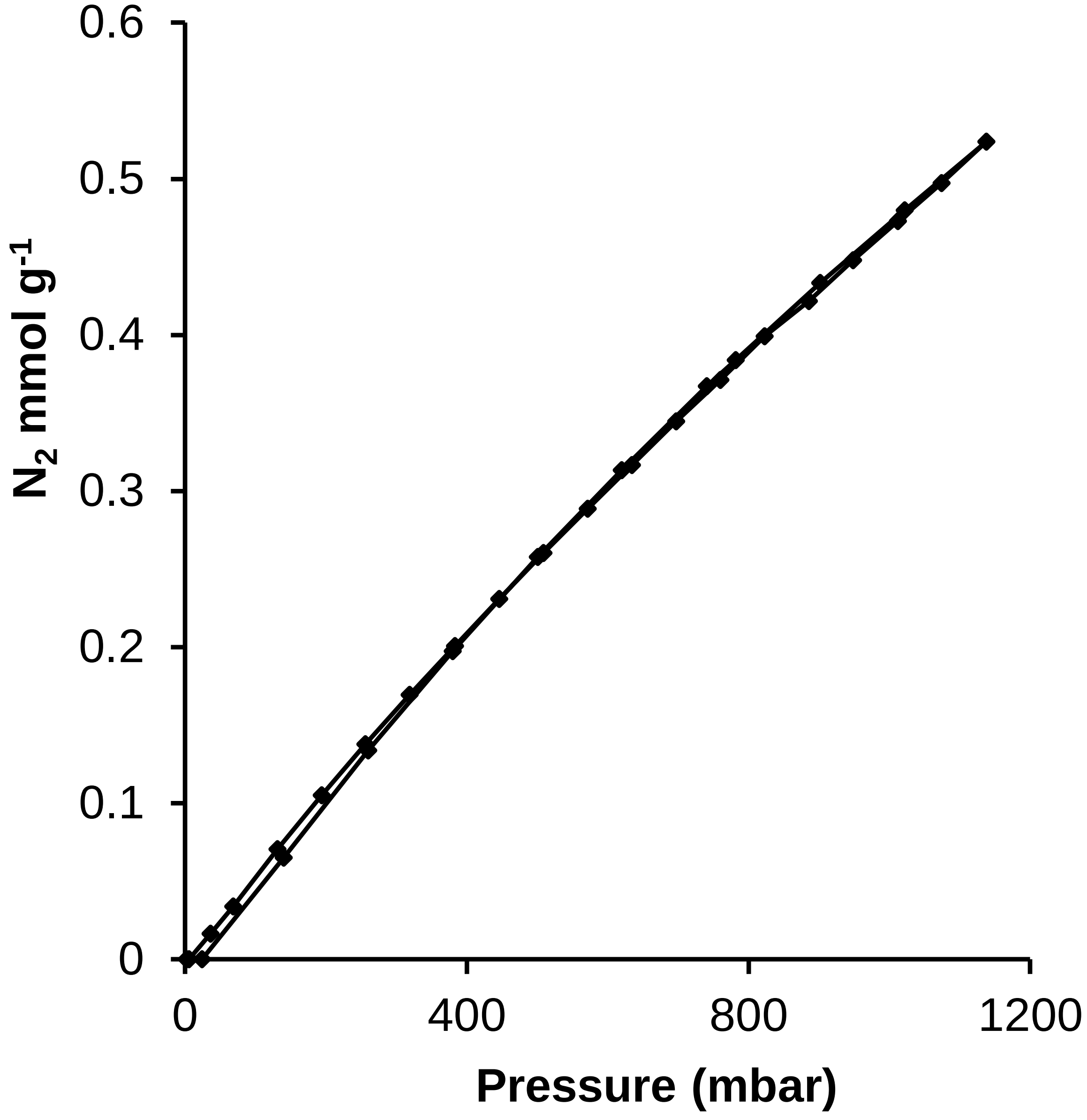

CH_4 adsorption isotherm onto S800

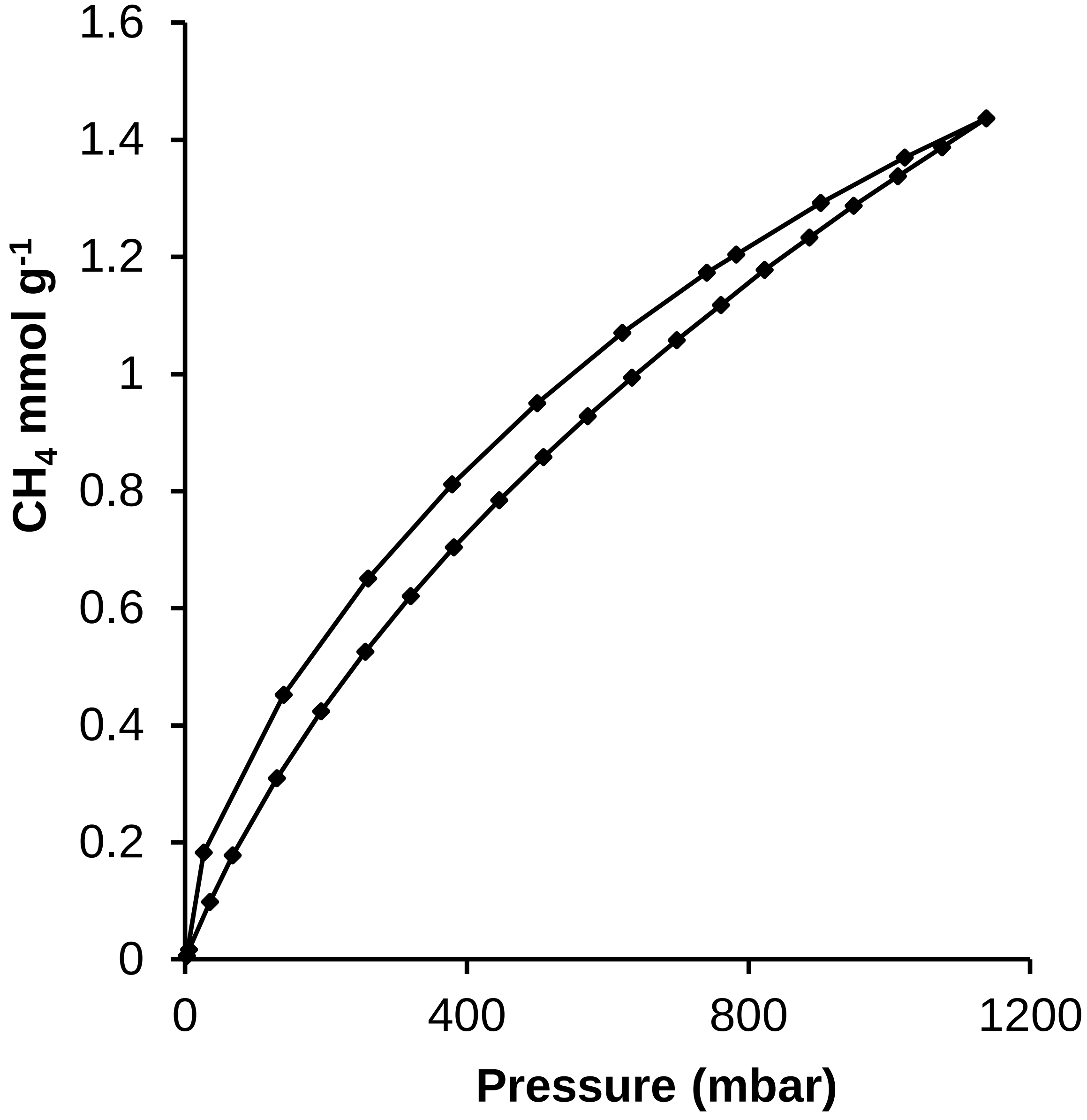

CH$_4$ adsorption isotherm onto SN$_{Gly}$800$_{Th}$

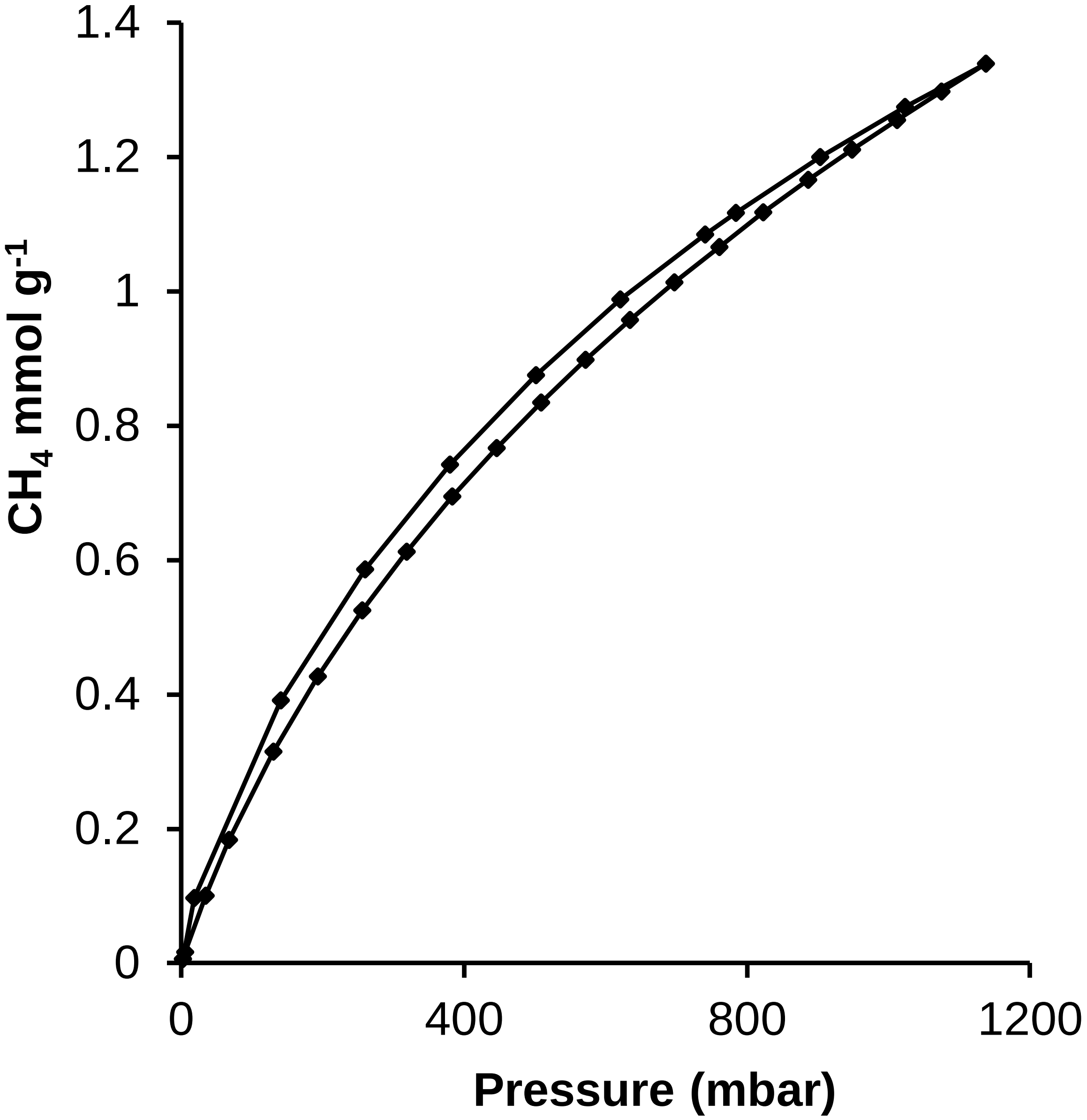

CH_4 adsorption isotherm onto $SN_{Gly}800_{Mo}$

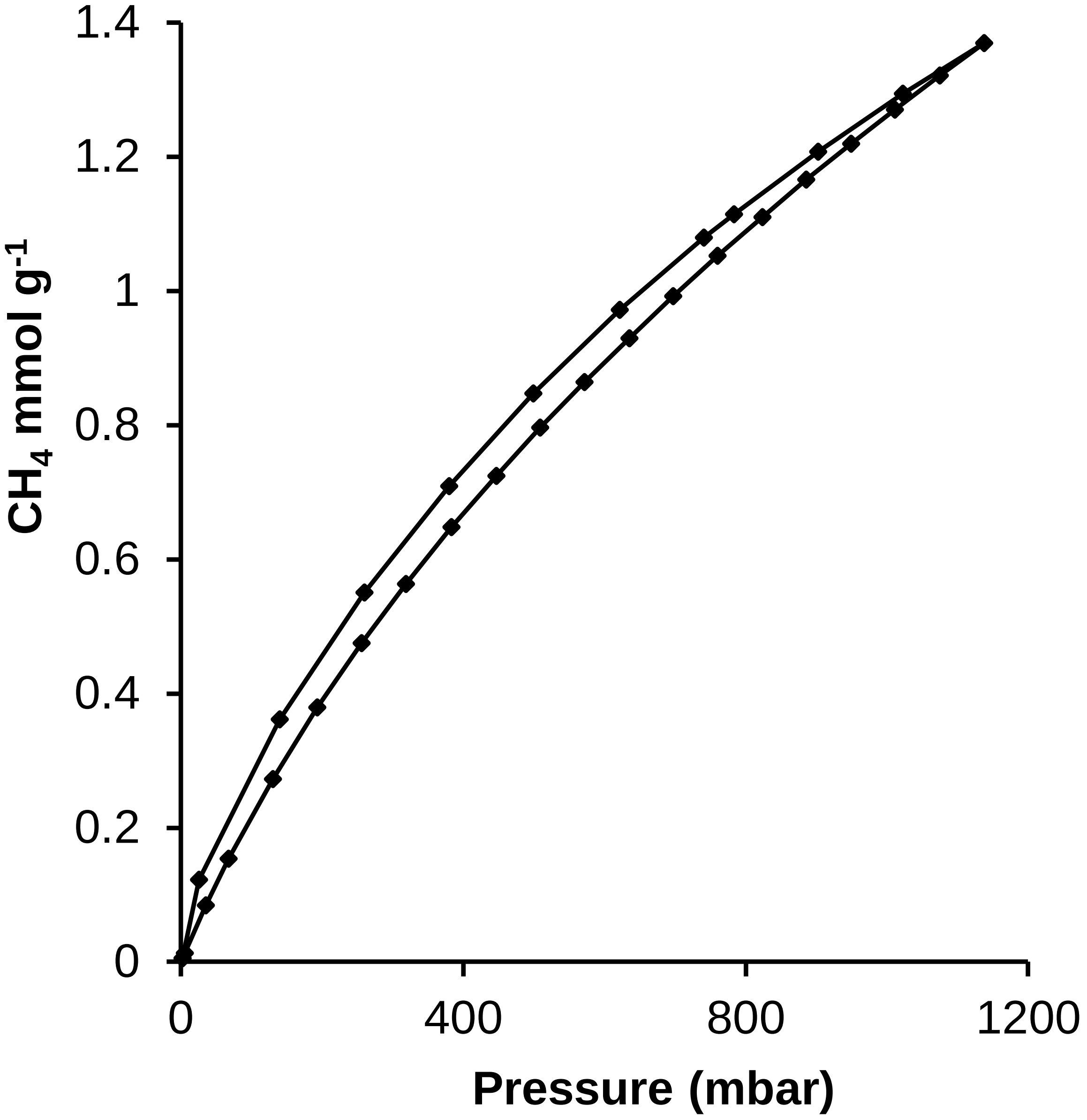

CH_4 adsorption isotherm onto $SN_{Gly}800_{Mu}$

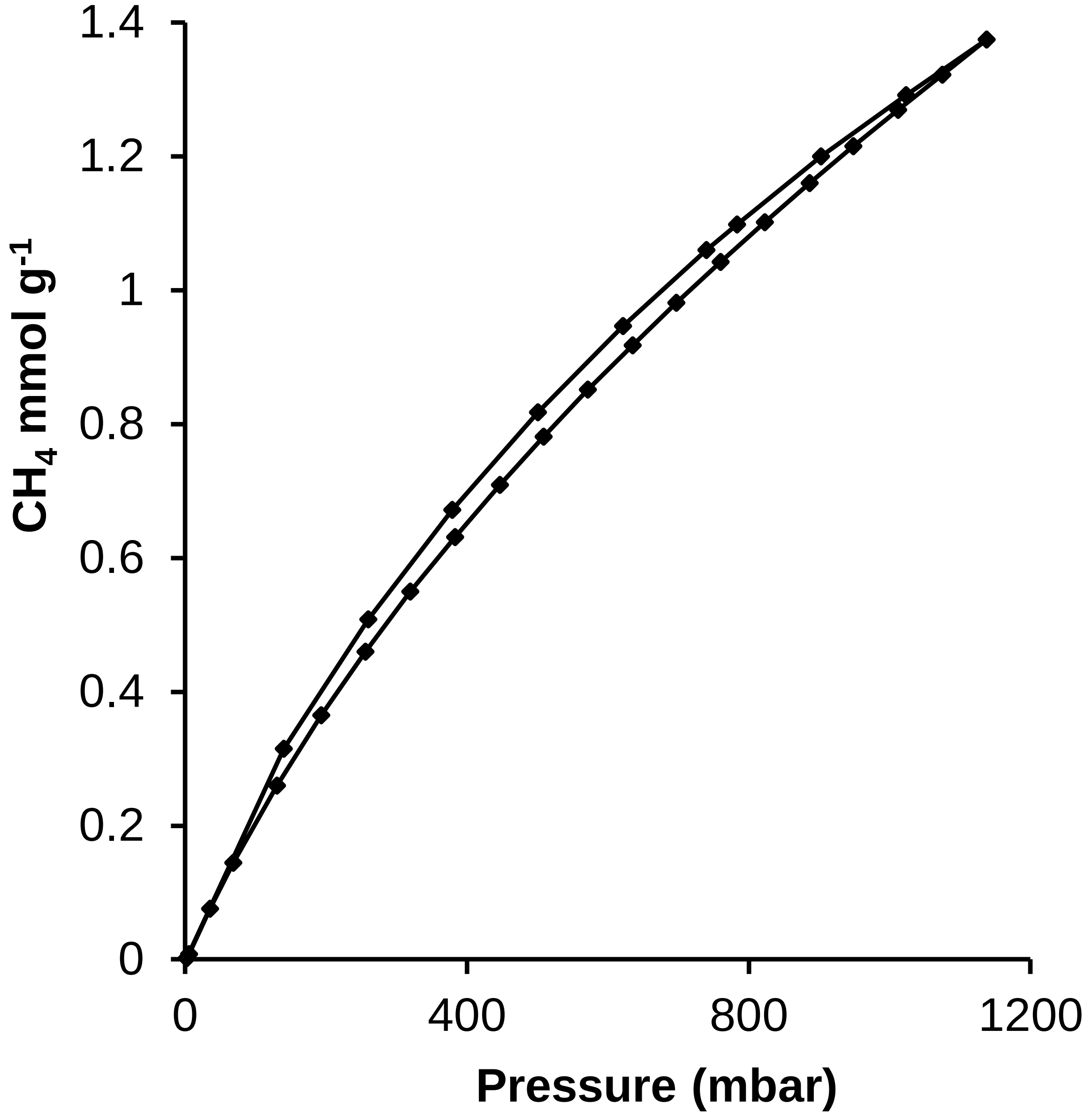

CH_4 adsorption isotherm onto $SN_{Bal}800_{Mo}$

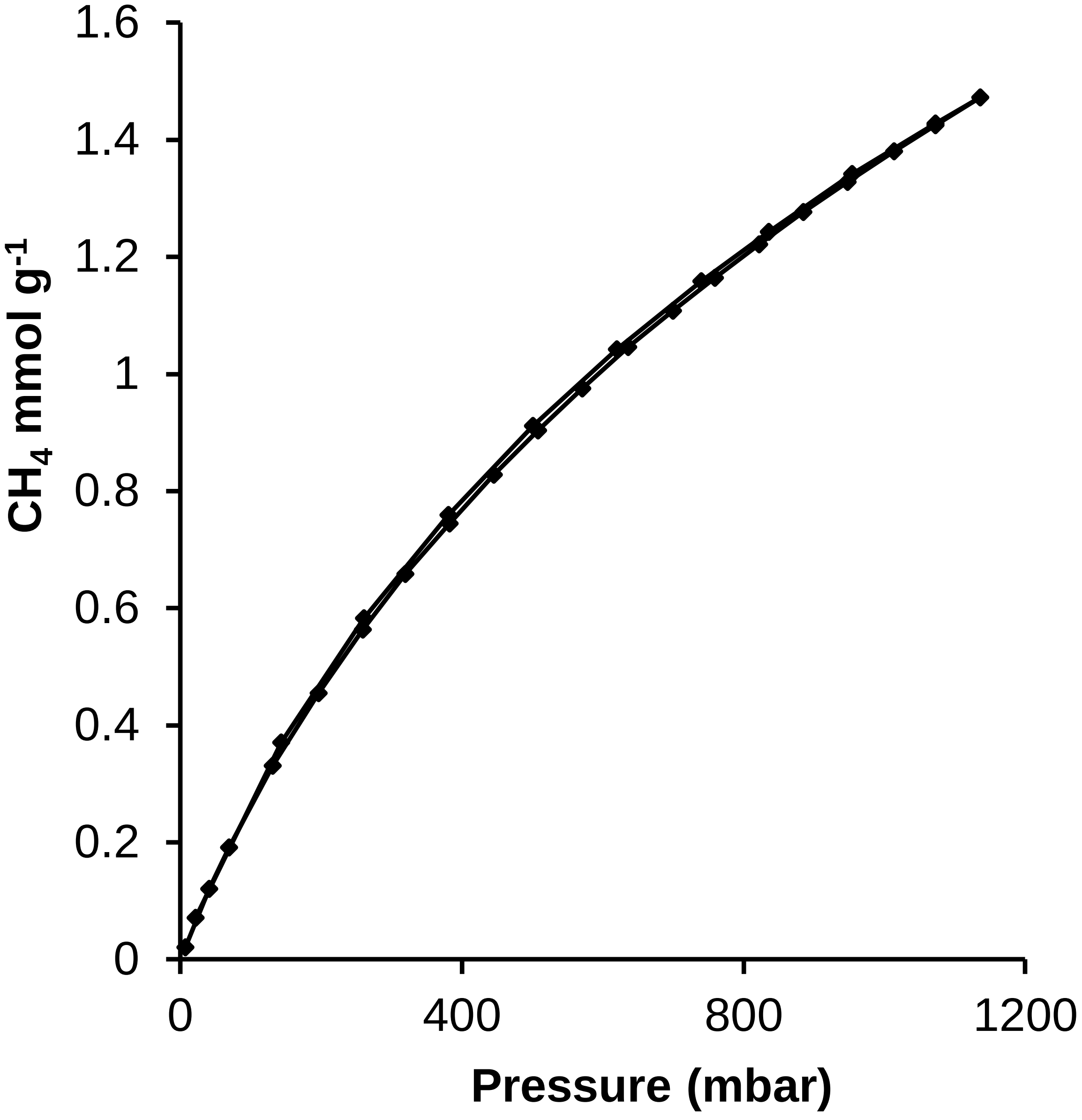

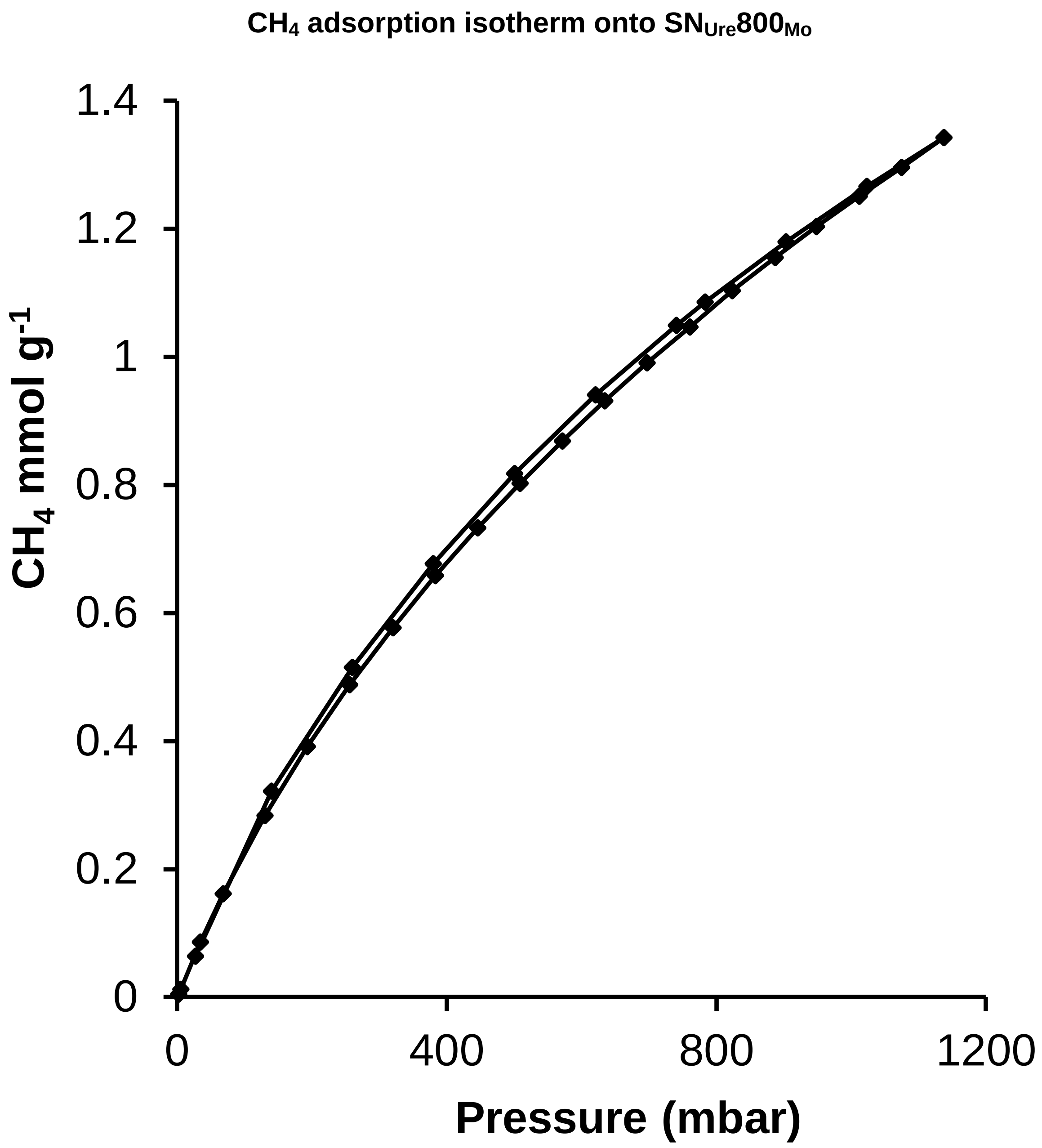
CH4 adsorption isotherm onto SNUre800Mo
1.4
1.2
1
0.8
0.6
0.4
0.2
0
CH4 mmol g-1
0
400
800
1200
Pressure (mbar)

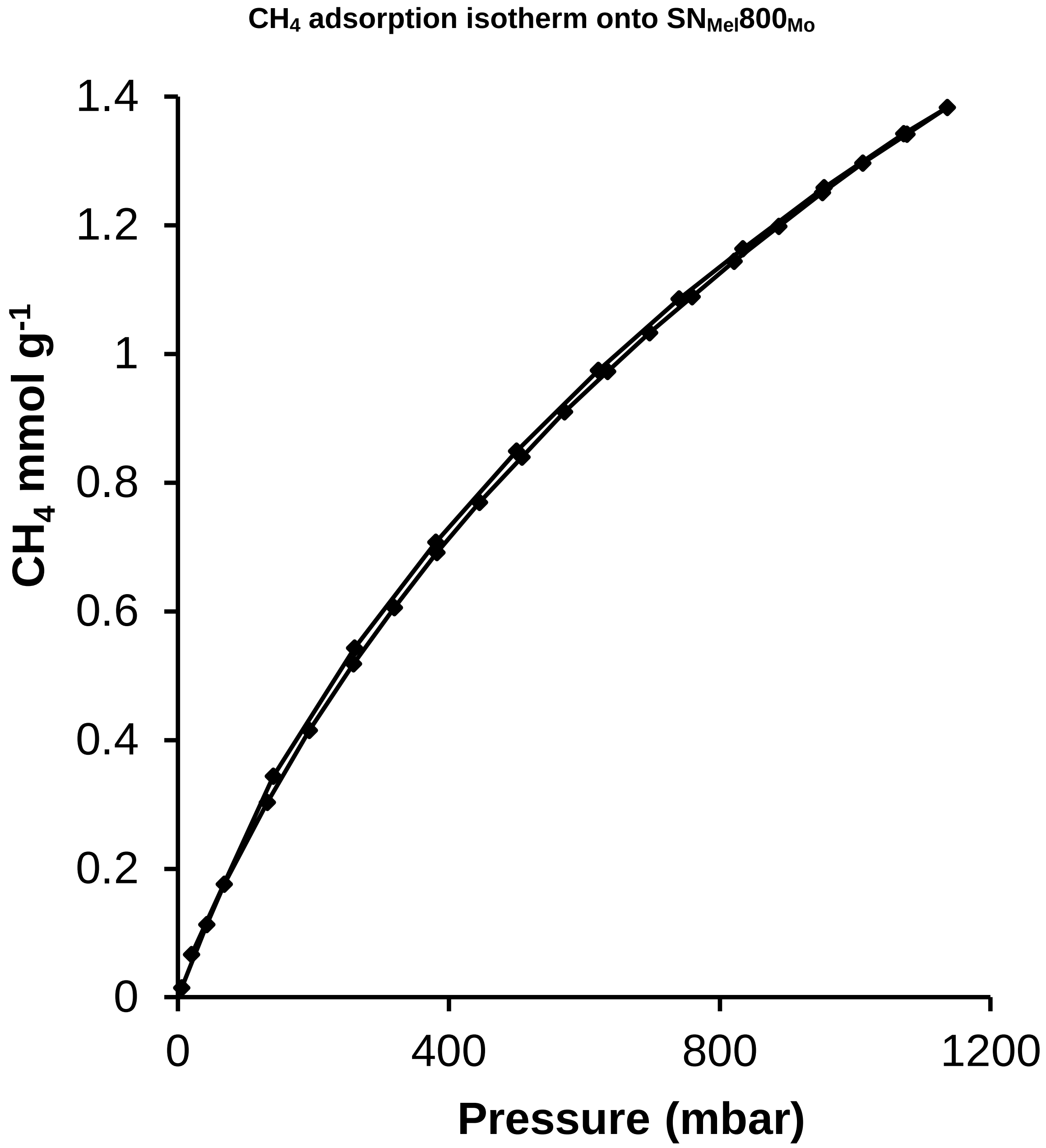
CH4 adsorption isotherm onto SNMel800Mo
1.4
1.2
1
0.8
0.6
0.4
0.2
0
CH4 mmol g-1
0
400
800
1200
Pressure (mbar)

CH$_4$ adsorption isotherm onto SN$_{Nic}$800$_{Mo}$

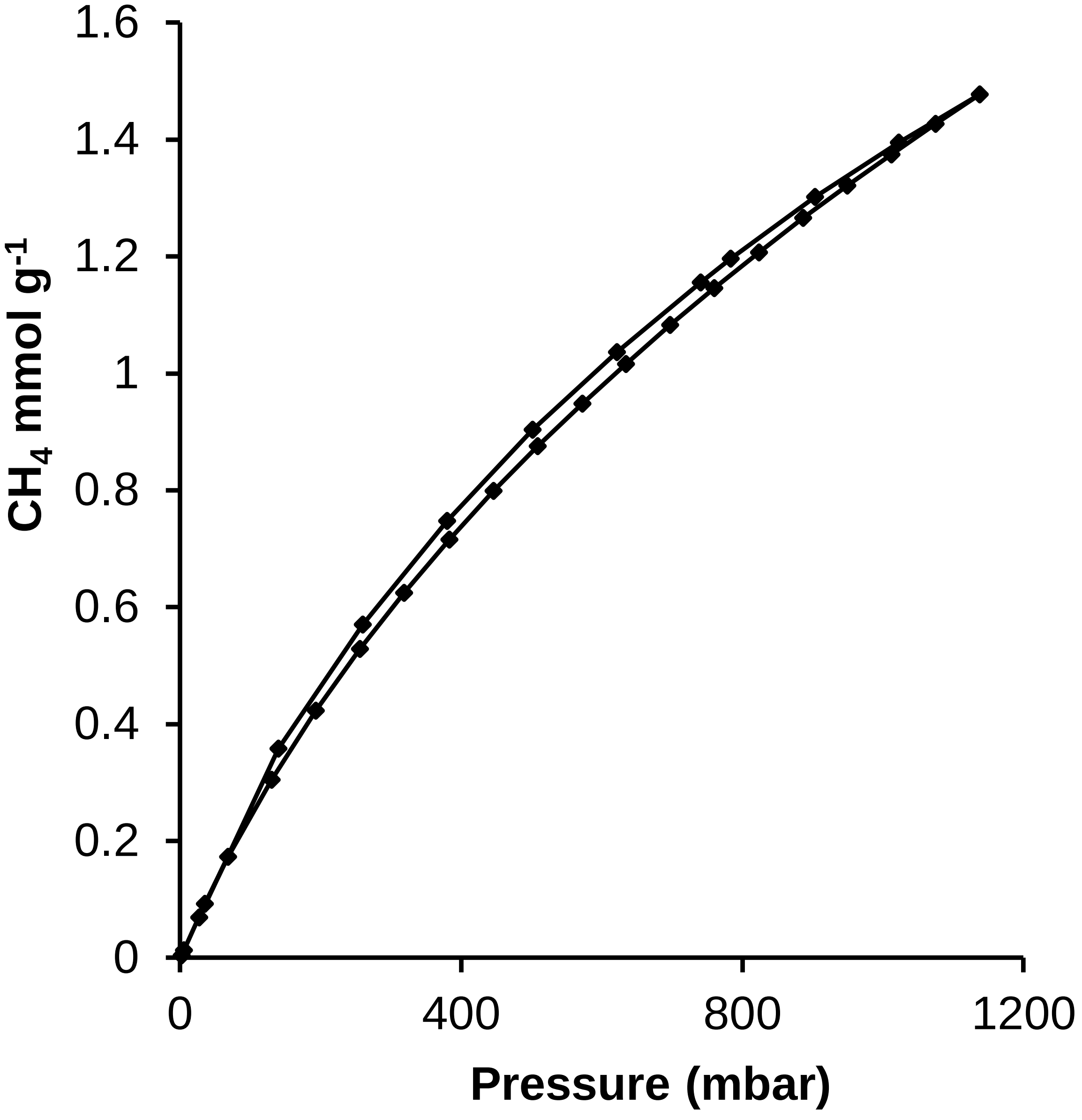

Supplement: Supplementary file 1 — Supporting Information [file CHEM-30-0-s001.pdf]
